# Identifying a Hidden Conglomerate Chiral Pool in the CSD

**DOI:** 10.1021/jacsau.2c00394

**Published:** 2022-09-23

**Authors:** Mark P. Walsh, James A. Barclay, Callum S. Begg, Jinyi Xuan, Natalie T. Johnson, Jason C. Cole, Matthew O. Kitching

**Affiliations:** ‡ Department of Chemistry Durham University, Lower Mount Joy, South Rd, DurhamDH1 3LE, United Kingdom; § Cambridge Crystallographic Data Centre, 12 Union Road, CambridgeCB2 1EZ, United Kingdom

**Keywords:** chirality, chiral pool, conglomerate crystallization, CSD, spontaneous resolution, spontaneous deracemization

## Abstract

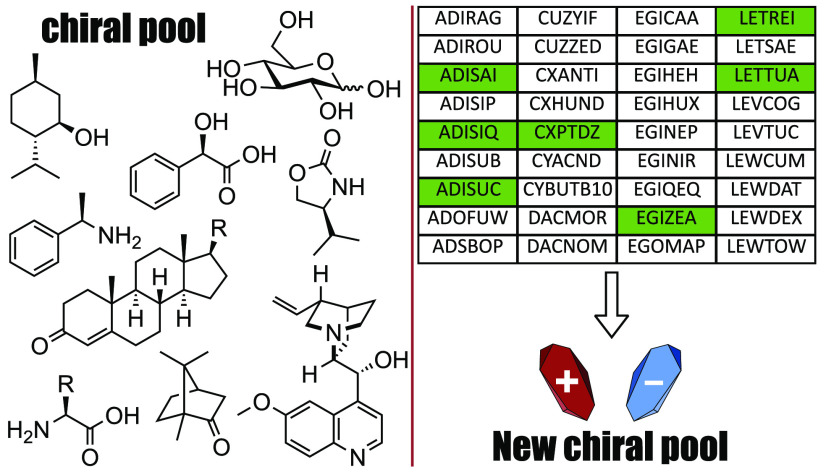

Conglomerate crystallization is the spontaneous generation of individually enantioenriched crystals from a nonenantioenriched material. This behavior is responsible for spontaneous resolution and the discovery of molecular chirality by Pasteur. The phenomenon of conglomerate crystallization of chiral organic molecules has been left largely undocumented, with no actively curated list available in the literature. While other crystallographic behaviors can be interrogated by automated searching, conglomerate crystallizations are not identified within the Cambridge Structural Database (CSD) and are therefore not accessible by conventional automated searching. By conducting a manual search of the CSD and literature, a list of over 1800 chiral species capable of conglomerate crystallization was curated by inspection of the racemic synthetic routes described in each publication. The majority of chiral conglomerate crystals are produced and published by synthetic chemists who seldom note and rarely exploit the implications this phenomenon can have on the enantiopurity of their crystalline materials. With their structures revealed, we propose that this list of compounds represents a new chiral pool which is not tied to biological sources of chirality.

## Introduction

**Figure 1:**
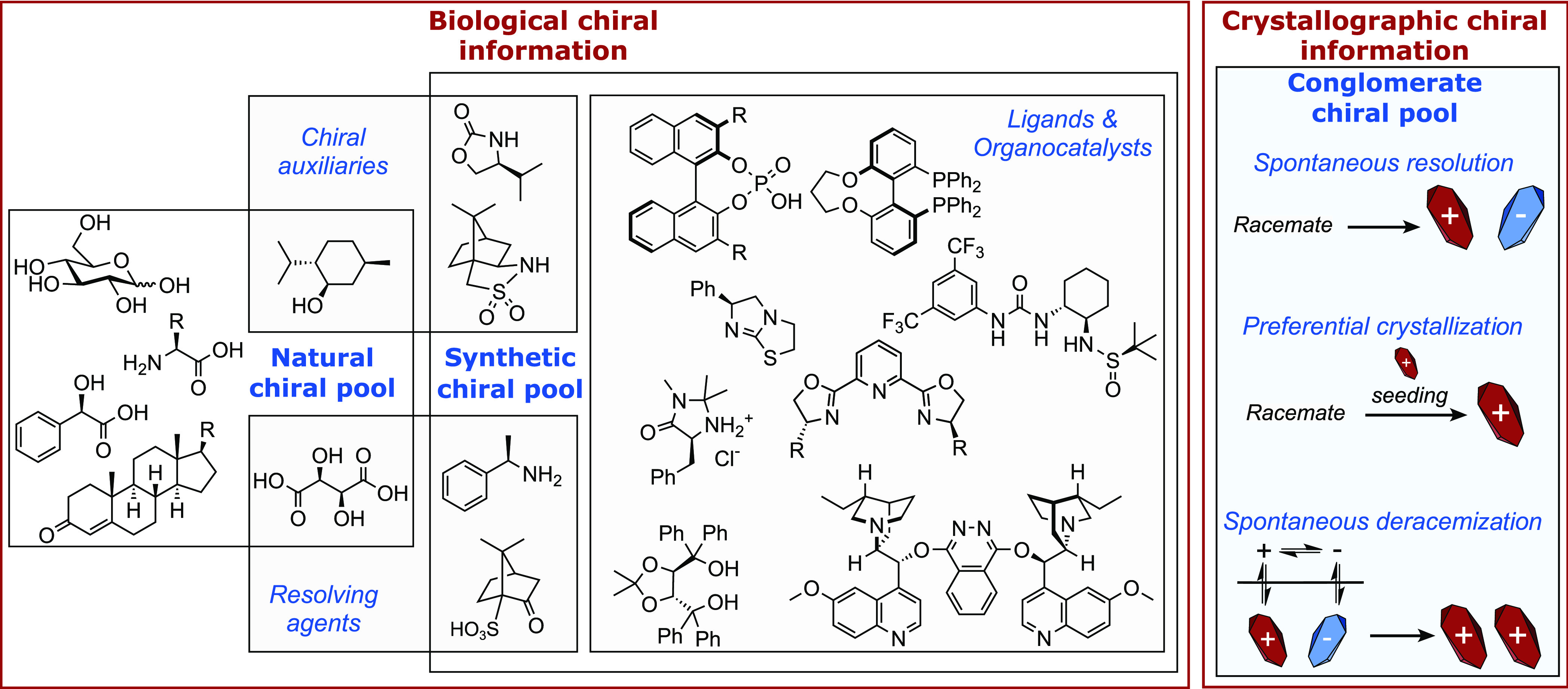
Euler diagram displaying the relationship between the natural and synthetic chiral pools, and the proposed conglomerate chiral pool.

Asymmetric synthesis fundamentally relies on the enantioenriched nature of biological systems. The natural chiral pool is fixed in size, constrained by evolutionary pressures of the organisms that produce its members, and limited in scaffold diversity. Due to the enantiopurity of biological machinery and their chemical precursors, often the resulting compounds are only naturally available in one enantiomeric form. Yet, synthetic chemists have used the chiral information handed to them by the natural world to great effect with increasing levels of stereocontrol (Figure 1).^1,2^ First, by using the natural chiral pool as a synthetic feedstock, new enriched derivatives are accessible, expanding the library of available enantioenriched materials to include a synthetic chiral pool. Exploiting this expanded library to mediate diastereoselective syntheses allows for the transfer of stereochemical information from the natural chiral pool to new, previously inaccessible stereogenic elements.^3−5^ However, this reliance on the natural chiral pool to supply chemical scaffolds can limit access to a singular enantiomeric form of a product. The solution to this problem comes with the development of resolution methods using materials derived from the natural and synthetic chiral pools, allowing for the separation of racemic non-natural scaffolds and therefore granting access to both senses of enantioenrichment of compounds not derived from the natural chiral pool. Temporary attachment of these materials and their derivatives, so-called chiral auxiliaries, to molecular frameworks allows for stereoselective transformations on substrates not part of the natural chiral pool. However, this strategy requires derivatization of the substrate molecule, installing stoichiometric amounts of chiral information in a covalent fashion. To overcome this limitation, modern asymmetric catalytic processes take these enantioenriched materials and employ them in transformations which impart stereochemical bias while only requiring substoichiometric amounts of the enriched material to be present, allowing chiral information to be amplified. Ultimately, all materials and asymmetric methods that synthetic chemists currently employ to access enantioenriched synthetic products *all rely on the chiral information originally imparted from biology*.^6^

There is an opportunity to create a chiral pool independent of biological chiral information: one which is based solely on the crystallographic properties of the material itself (Figure 1). In the case of racemic compounds, the most likely outcome from a crystallization is the formation of racemic crystals—crystals in which equal proportions of enantiomers are present. However, in a substantial number of cases, an intriguing crystallographic phenomenon can occur. Here, a racemic material can undergo spontaneous resolution with each crystal containing a single enantiomer in a process referred to as a conglomerate crystallization.^200^ This phenomenon imparts chiral information on the material and the chemist is no longer in the confines of the natural chiral pool to achieve enantioenrichment. Broadly speaking, there are three methods to exploit conglomerate crystallization for accessing enantioenriched materials. The first is to follow an unbiased spontaneous resolution protocol in which both enantiomers crystallize as discrete crystals from the mixture. This was originally described by Pasteur when he separated individual enantiomorphic crystals of sodium ammonium tartrate by hand.^7^ While this method was historically fundamental in the discovery of molecular chirality, this method is too arduous to be applicable in modern synthetic processes. The second method in which a conglomerate crystal may be exploited to produce enantioenrichment is to conduct the crystallization in the presence of seed crystals of the desired enantiomer. This seed crystal imparts a kinetic bias to coax the matching enantiomer to crystallize from solution, removing the need to manually sort the resulting crystals.^8−11^ This method, called preferential crystallization (also known as resolution by entrainment or *dédoublement par entraînment*), can be regarded as a stereoselective crystallization. The mother liquors will be left enriched in the opposite enantiomer, which can be brought to supersaturation and seeded with the opposite enantiomer. Alternating the enantiomer of the seed crystal and replenishing the mother liquor solution with racemic material allows for indefinite cycles of resolution to occur. As such this method is an attractive resolution strategy and numerous examples of this strategy have been applied in continuous crystallization modes, rendering the process incredibly efficient for large scale industrial processes requiring enantioenriched materials.^12−15^ A list of conglomerate crystals which have been resolved by preferential crystallization is available in the Supporting Information.

The third and the most recently discovered possibility is to combine a conglomerate crystallization with solution phase racemization and a symmetry breaking event. With careful control of the crystallization/racemization conditions, deracemization of the bulk material can be achieved without external chiral influences, i.e., a spontaneous asymmetric synthesis.^16−23^ The modern method to achieve a spontaneous asymmetric synthesis is by following an attrition-enhanced deracemization protocol, more commonly known as Viedma ripening.^24,25^ While the first attrition-enhanced deracemization of conglomerate crystals was performed on sodium chlorate and sodium bromate salts,^26−30^ this process has since been exploited to produce chiral organic molecules with high enantiopurity, where the chirality of the molecule is maintained upon dissolution of the crystal. The advantage in this strategy is the ability to convert the undesired enantiomer by racemization to the desired enantiomeric form, giving a theoretical maximum yield of 100%. This is in contrast to spontaneous resolution and preferential crystallization protocols which are restricted to 50% yield of a desired enantiomer. Importantly, all three strategies that use conglomerate behavior to achieve enantioenrichment rely solely on the crystallographic behavior of the material without recourse to the natural chiral pool.

Many desirable traits exist for a conglomerate chiral pool based on crystallographic chiral information as opposed to a chiral pool originating from biological sources. There is no limit to which materials could crystallize as a conglomerate, thus providing a vast range of diverse scaffolds which can be spontaneously enantioenriched. A conglomerate crystal is not dependent on a particular organism to produce an abundance of a desired compound to be economically viable. Practically speaking, both enantiomers are equally likely to crystallize (unless a specific enantiomer is deliberately biased from the crystallization), allowing access to both enantiomeric forms of the members of this chiral pool. While the synthetic chiral pool can increase in size through extended chains of resolution or enantioinduction, ultimately the original source of the chiral information remains the natural chiral pool. In contrast, conglomerate crystallization imparts enantioselectivity directly at the crystallization event, providing ever increasing access to sources of chiral information. As chemists continue to synthesize and crystallize new materials, more conglomerate crystals should be discovered every year, thus increasing the structural diversity present in this enantioenriched library. Given these advantages, why is the phenomenon of conglomerate crystallization not currently being widely exploited for augmenting our current sources of chirality and as a means to generate this new chiral pool?

The answer is the lack of documentation. The main hurdle in the adoption of this phenomenon for producing enantioenriched materials is the lack of curated knowledge of which crystals have the capacity to crystallize as conglomerates. The CSD (Cambridge Structural Database) is the largest and most widely adopted crystallographic repository service which is charged with the curation of crystallographic data produced by chemists. At the time of writing, it boasts over 1.1 million structures which can be searched and freely accessed by the community. The development of automated means to search this database with CCDC developed software (*ConQuest*) and community developed algorithms^31^ have led to new insights regarding statistical crystal behaviors.^32−34^ However, the CSD does not require conglomerate crystallization behavior to be identified in their metadata at submission, leading to a loss of this information as a search term in the database. Retrieving conglomerate crystals from this database is further frustrated by the difficulties in their prediction. While efforts to rationalize conglomerate crystallization have been conducted using crystal structure prediction,^35^ structural modifications,^36−38^ and supramolecular interactions,^39,40^ currently only direct measurements of the physical characteristics of a crystal can identify conglomerate behavior conclusively.

The typical work-flow of how X-ray crystallography samples are solved in most academic institutions is not conducive to the communication of conglomerate behavior between the synthetic chemist and the crystallographer, symptomatic of a traditional view of separated scientific disciplines. Often the synthetic chemist will supply a crystalline sample with a proposed structure and the solvent of crystallization to the collaborating crystallographer. Communication of the synthetic origin of the sample is less standardized and whether the starting materials are racemic or enriched, possibly unclear. Without this information it is impossible for the crystallographer to unambiguously identify conglomerate behavior. The sample will then be solved and returned to the synthetic chemist, who is generally interested in the connectivity of the molecule and relative stereochemistry within the crystal (unless they specifically ask for confirmation of absolute configuration). The importance of Sohncke space groups^41^ or Flack parameters^42^ in the crystallographic data has the potential to be overlooked, or at least unreported, by the synthetic community leading to the possibility of conglomerate behavior being unidentified. The CIF (crystallographic information file) is deposited in the CSD by the crystallographer and now the synthetic chemist, crystallographer and the wider chemistry community are unaware of the full crystallographic behavior of this sample. Once deposited, the conglomerate crystal can no longer be retrieved selectively without also bringing up thousands of nonconglomerate crystals which have been produced by enantioselective means. Therefore, this foundational phenomenon responsible for the discovery of molecular chirality is currently being undocumented by both the synthetic and crystallographic communities.

It is only once a phenomenon has been documented that it can be fully exploited for its true potential by members of the chemical community. The most complete list of potential conglomerate crystals was compiled by Jacques, Collet, and Wilen in their influential book published in 1981;^43^ however, the reports of the crystals contained in this list mostly predate the CSD. There is no actively curated list of chiral conglomerate crystals available in the literature. It is also understood that an automated search of the CSD to identify conglomerate crystallization cannot be achieved without prior recording of metadata; that is to say, conglomerates are hiding in plain sight within the CSD.^34^ The wealth of crystallographic information present in the CSD represents an untapped resource for confirmed conglomerate behavior. To extract this information, a manual search of crystals in the CSD would have to be conducted, which would interrogate the origin of each chiral crystal to ensure it originated from a racemic synthetic process. This requires manually examining each reported synthetic route. We sought to tap into this wealth of crystallographic and synthetic potential by conducting such a manual search of the CSD for previously unidentified conglomerate crystallizations in order to catalogue this new chiral pool.

## Results and Discussion

### Methods for Conglomerate Identification

The full list of conglomerate crystals along with their chemical structures and associated references are available in the Supporting Information. While the formation of chiral conglomerate crystals from achiral materials is also possible,^44−47^ this work focused specifically on documenting the spontaneous resolution phenomenon for chiral organic molecules which will be of interest for the synthetic community. The queries generated to conduct the search are detailed in the Experimental Section. Once a list of candidates (21,098 crystals) was generated from search queries of the CSD mediated by *ConQuest*, a manual search and interpretation of the reported syntheses for the crystals within the CSD was undertaken to identify conglomerate crystallization.

Caution had to be taken to distinguish between absolute and relative stereochemistry and the use of stereochemical notation to display perspective in compound representations. Crucially, confirming if a crystal had displayed conglomerate behavior relied on the ability to trace the stereochemical enrichment of the starting materials and rule out any use of asymmetric methodology throughout the synthesis. In cases where the synthetic route for the compound was not available, or the described synthetic route was ambiguous in stereochemical information on the precursors, these examples were omitted. As such, all structures which were only available as a *CSD Communication* were excluded as the origins of these materials was not possible to interrogate. Of course, the following assumptions had to be made while interpreting the reported syntheses and crystallizations within this list. It is assumed that the authors have reported the syntheses and the nature of the enrichment of their reagents/catalysts accurately, that the crystal structure(s) they reported indeed were crystallized from the batch of material as described and that the crystal structures themselves have been solved accurately (i.e., the space groups are correctly assigned).

### Trends in Publication and Deposition of Conglomerate Crystals

From this search, 1626 conglomerate crystal structures were identified within the CSD. A further 210 structures were compiled from literature searches from known conglomerate crystallizations and preferential crystallizations, some of which with as-yet undetermined or unreported crystal structures. A recent analysis of the CSD in 2020 by Rekis^32^ suggests that 9.5% of the chiral compounds which crystallized in Sohncke space groups would be conglomerates, giving an estimated 4281 conglomerates of chiral organic compounds hidden in the CSD. If this estimate is correct, the list curated in this work accounts for 38% of the chiral organic conglomerates currently unaccounted for in the CSD. An intriguing question arises from this search: *How many compounds which have been prepared in a nonracemic fashion, and thus were excluded from this list, would show conglomerate behavior?* This includes molecules isolated from natural sources, pharmaceuticals, ligands, organocatalysts, and peptide oligomers—most of which have only been prepared in an enantiomerically enriched form and so any conglomerate behaviors would remain obscured.

We sought to determine whether conglomerate crystallization could also be elucidated using the current methods of recording enantioenrichment of deposited crystals in *CIF dictionary* approved terms. This was achieved by conducting an internal search of the deposited crystallographic data in the CSD for the fields which can record enantioenrichment in a crystalline sample. Only 12 crystals in the CSD contained an entry for the “_chemical_enantioexcess_*” fields, none of which were conglomerate crystals (see the Supporting Information). In comparison, only 17 entries in the CSD have conglomerate behavior unofficially identified using text strings within the deposited CIF, which can be found using *ConQuest*. These results demonstrate that there is no other means to search the CSD for conglomerate crystals, due to the way that CIFs have been prepared and deposited into the CSD without any attempt to record conglomerate behavior using official *CIF dictionary* fields or unofficial text strings.

**Figure 2:**
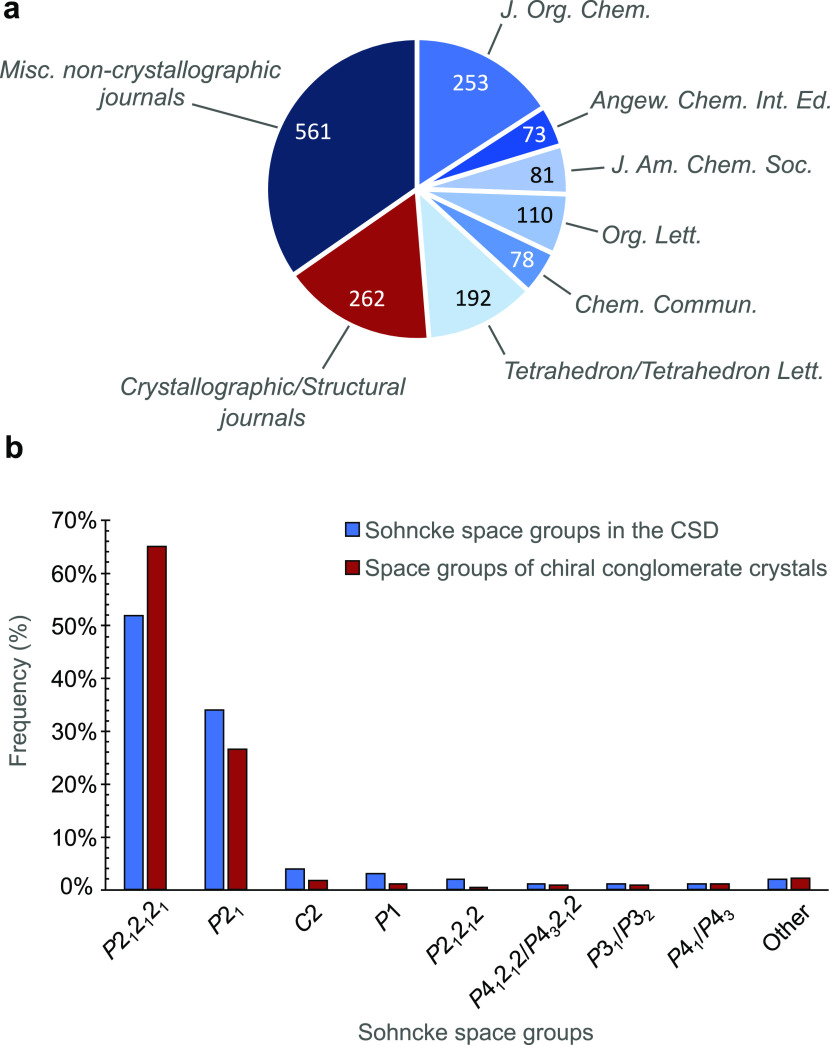
(a) Number of publications by journal which contained a conglomerate crystal identified by manual search of the CSD (*n* = 1610). (b) Comparison of the distribution of the Sohncke space groups for enantioenriched chiral materials in the CSD (*n* = 39,894, blue chart) and distribution of the space groups exhibited by chiral conglomerate crystals found by manual searching of the CSD and literature sources (*n* = 1765, red chart).

The majority of the conglomerate crystals found from our manual search of the CSD had been originally reported by synthetic groups publishing in non-crystallographic journals, reflecting the vast number of crystallographic samples produced by the synthetic community. A breakdown of the literature sources of conglomerate crystals is shown in Figure 2a. Non-crystallographic journals make up 84% of this conglomerate list. It appears that synthetic chemists publishing in *J*. *Org*. *Chem*., *Org. Lett*., *Tetrahedron*, and *Tetrahedron Lett*. are responsible for 34% of the papers containing conglomerate crystals. In almost all cases where a conglomerate appears in a synthesis focused paper, the phenomenon is not commented on in the CIF or the respective paper. Of the 1,626 conglomerates found from the manual search of the CSD, only 120 mentioned conglomerate behavior in the manuscript text.

Conglomerates have no distinguishing features in their routinely recorded crystallographic metadata which identify them from other enantioenriched compounds. A comparison of the frequency of space groups present in conglomerate crystals (*n* = 1765; red chart, manual CSD search and literature sources) and the frequency of Sohncke space groups in the CSD for enantioenriched species (*n* = 39,894; blue chart)^32^ was conducted (Figure 2b). While there is a slightly greater prevalence of *P*2_1_2_1_2_1_ within the conglomerate data set (65%) than observed in the CSD (52%), the overall trends of space group frequency of conglomerates match those observed in the CSD. The implications of this are clear: once a crystal is deposited in a crystallographic database such as the CSD, only a manual review of the synthetic route to the compound will be able to identify conglomerate behavior.

### Structural Observations in Conglomerate Behavior

**Figure 3:**
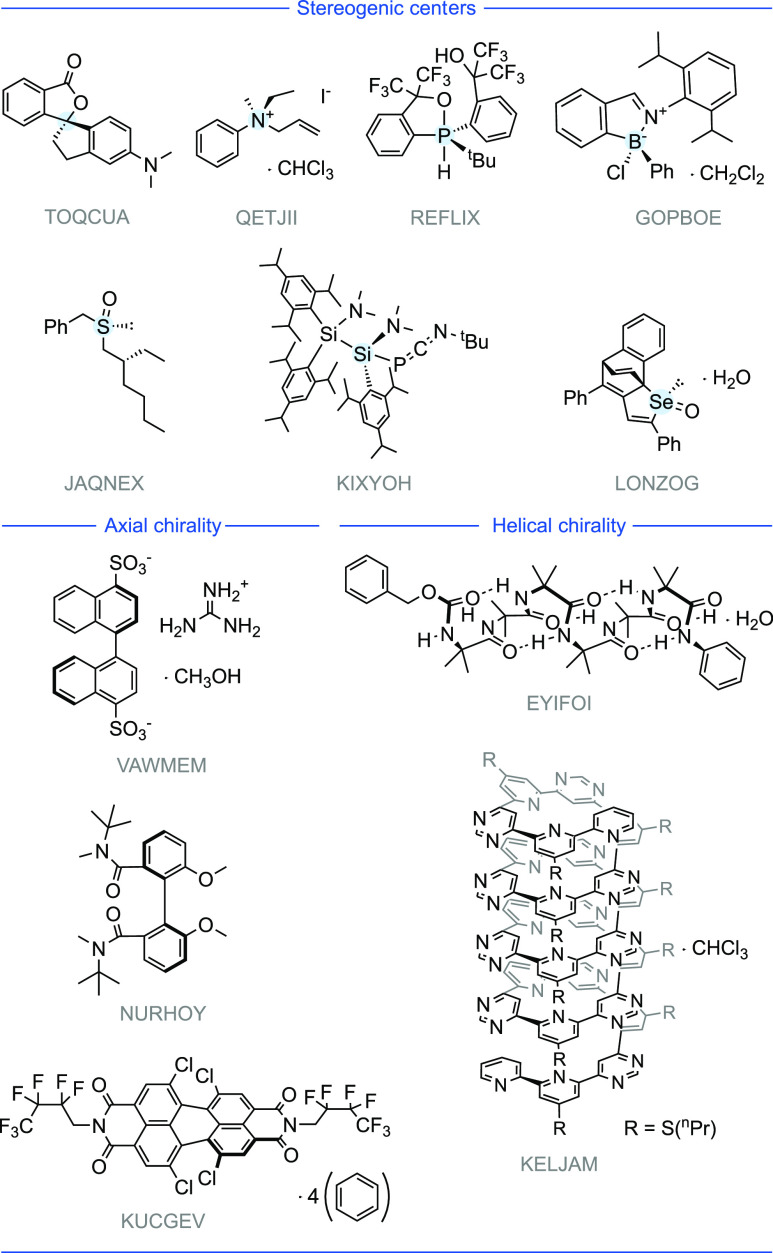
Types of stereocenters resolved by conglomerate crystallization and other chiral elements present in conglomerate crystals. The following crystal structures, labeled by their CSD Refcode, have been identified as conglomerates: TOQCUA,^48^ QETJII,^18,22^ REFLIX,^49^ GOPBOE,^50^ JAQNEX,^51^ KIXYOH,^52^ LONZOG,^53^ VAWMEM,^54^ EYIFOI,^55^ NURHOY,^56^ KUCGEV,^57^ KELJAM.^58^

Conglomerate behavior was observed in all manner of chiral compounds, with no apparent limiting factors on what structures can undergo this process. Carbon, nitrogen, phosphorus, boron, sulfur, silicon, and selenium based stereocenters were among the compounds resolved by conglomerate behavior (Figure 3). Other stereogenic elements are also possible to enrich by crystallization, including axial chirality in the form of atropisomeric (VAWMEM,^54^ NURHOY^56^) and twisted structures (KUCGEV^57^). Larger supramolecular examples also demonstrate the potential to be a conglomerate crystal, including a helical Aib_6_ foldamer (EYIFOI^55^) and a helical pyridine-pyrimidine superstructure (KELJAM^58^). These demonstrate the diversity of structures which are within this list of conglomerate crystals.

### Conglomerate Crystallization in Natural Product Synthesis

**Figure 4:**
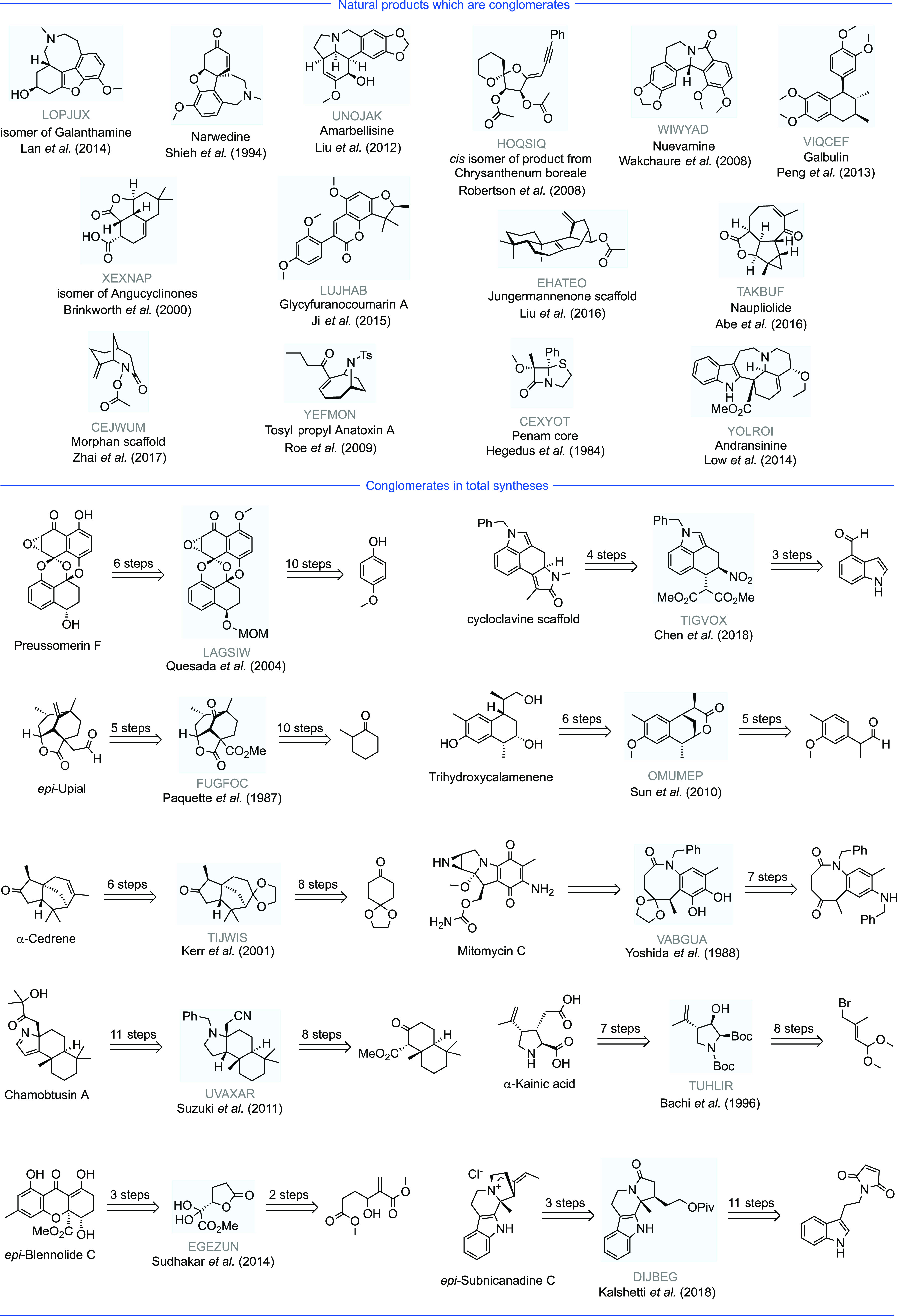

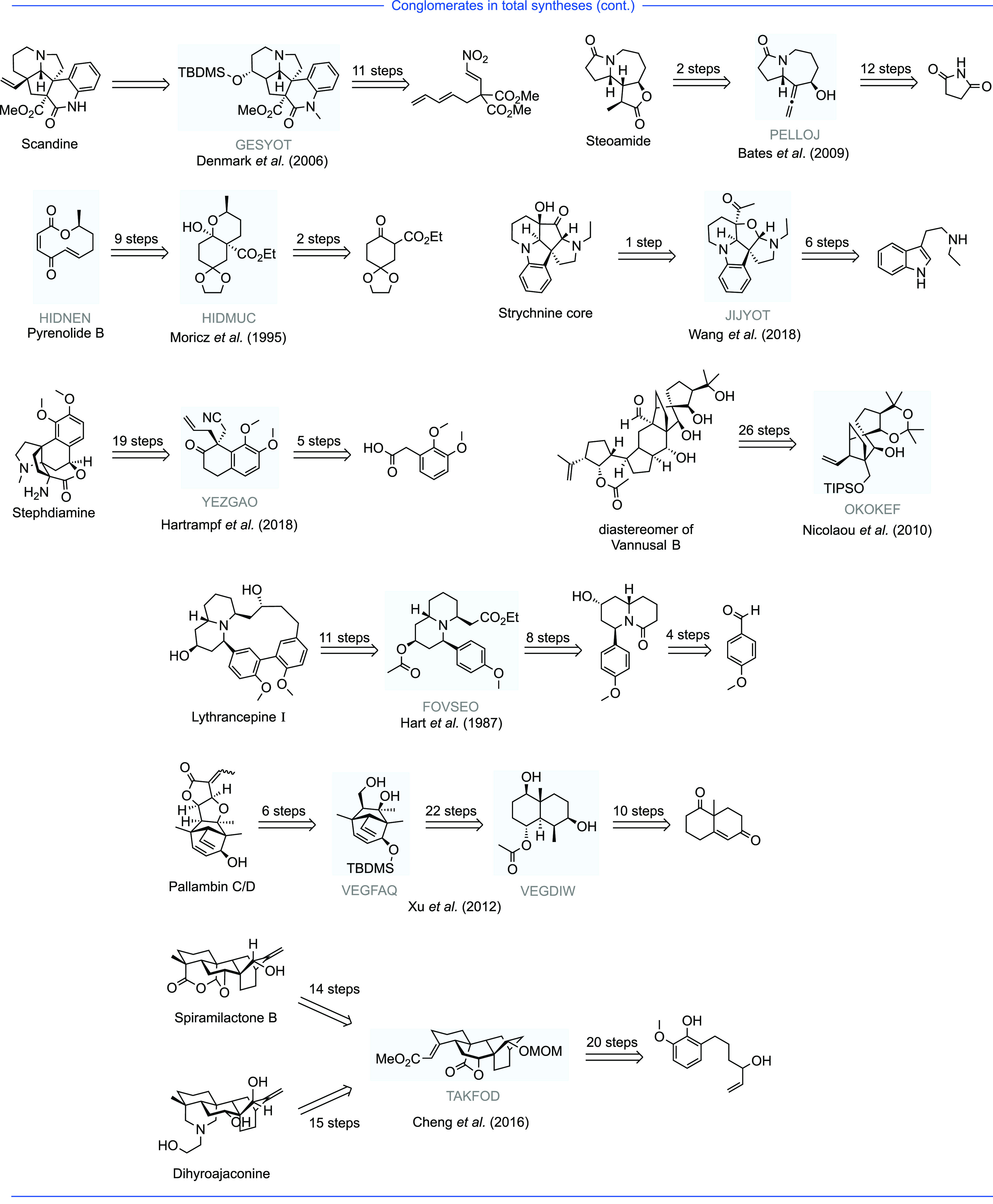
Natural products and total syntheses which contain a conglomerate crystal. The following crystal structures, labeled by their CSD Refcode, have been identified as conglomerates: LOPJUX,^61^ UNOJAK,^62^ HOQSIQ,^63^ WIWYAD,^64^ VIQCEF,^65^ XEXNAP,^66^ LUJHAB,^67^ EHATEO,^68^ TAKBUF,^69^ CEJWUM,^70^ YEFMON,^71^ CEXYOT,^72^ YOLROI,^73^ LAGSIW,^74^ TIGVOX,^75^ FUGFOC,^76^ OMUMEP,^77^ TIJWIS,^78,79^ VAGBUA,^80^ UVAXAR,^81^ TUHLIR,^82^ EGEZUN,^83^ DIJBEG,^84^ GESYOT,^85^ PELLOJ,^86^ HIDNEN,^60^ HIDMUC,^60^ JIJYOT,^87^ YEZGAO,^88^ OKOKEF,^89^ FOVSEO,^90^ VEGFAQ,^59^ VEGDIW,^59^ TAKFOD.^91^

Structural complexity is not a barrier to conglomerate behavior. Since natural product synthesis has been a core area of study for organic chemists for decades, we wished to pay special attention to conglomerate crystals discovered in this area. We have noted a number of natural products and related scaffolds that exhibit conglomerate behavior when prepared in racemic fashion and crystallized (Figure 4). Notably, in these examples, the authors rarely note that spontaneous resolution had occurred during crystallization. There were also notable examples of conglomerate crystals appearing within the synthetic routes of racemic total syntheses. For example, in the synthetic routes to Pallambin C/D^59^ and Pyrenolide B,^60^ both routes contained two structures which crystallized as conglomerates within the synthesis. This established that in some synthetic routes there can be multiple instances of conglomerate behavior. The number of observed conglomerate crystals in natural products will be underestimated in this list as it was assumed that any material extracted from a biological source would be enantioenriched and so were discounted. Synthetic chemists have also been incentivised to produce enantioselective routes to natural products, which would also obscure conglomerate crystallization. The use of a conglomerate crystallization resolution or the development of racemization conditions to allow for attrition-enhanced deracemization within these established routes would give access to enantioenriched natural products.

### Conglomerate Crystallization in Medicinal Chemistry and Crystal Engineering

**Figure 5:**
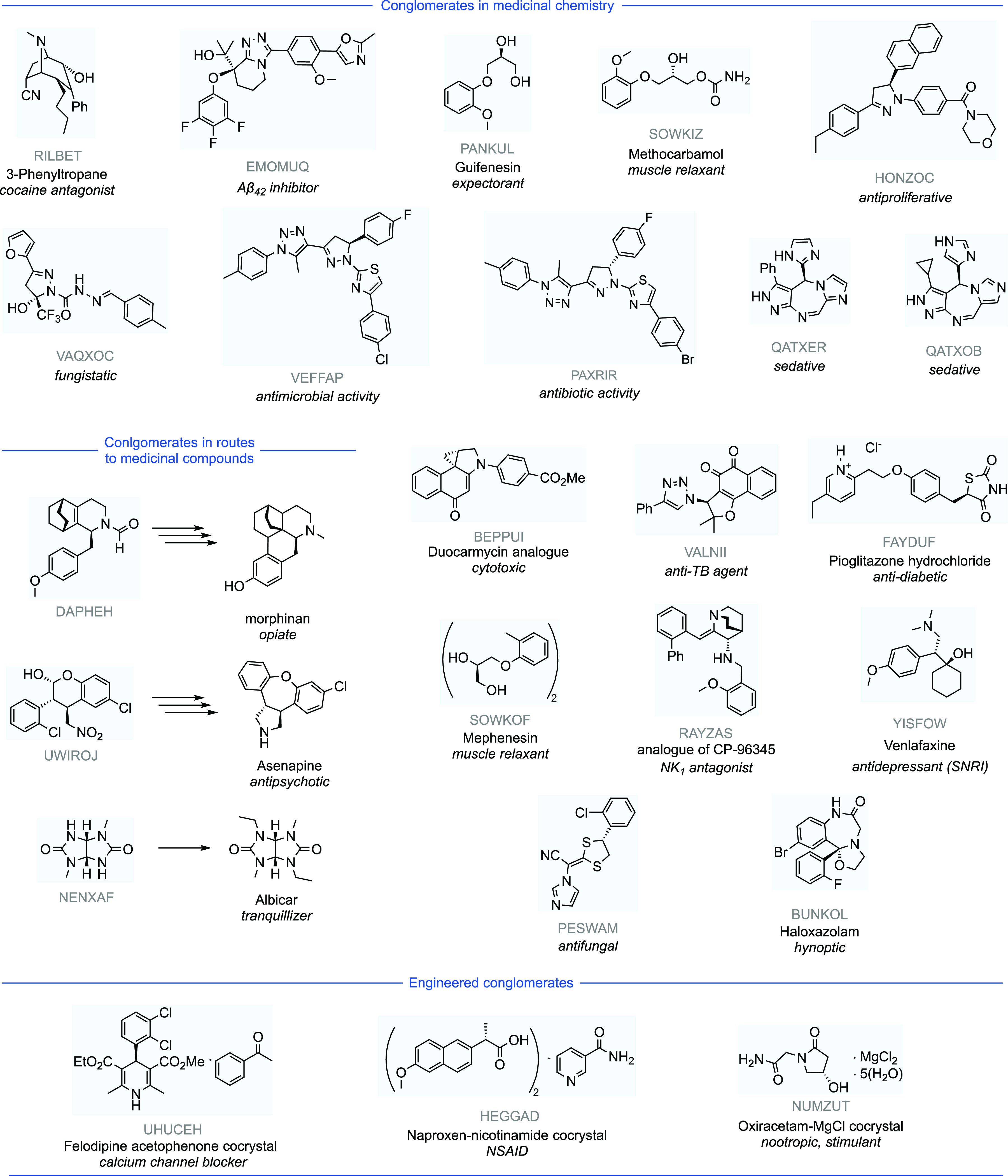
Conglomerate crystals present in medicinally relevant compounds. The following crystal structures, labeled by their CSD Refcode, were identified as conglomerates: RILBET,^103^ EMOMUQ,^104^ PANKUL,^105^ SOWKIZ,^105^ HONZOC,^106^ VAQXOC,^107^ VEFFAP,^108^ PAXRIR,^109,110^ QATXER,^111^ QATXOB,^111^ DAPHEH,^112^ BEPPUI,^113^ VALNII,^114^ FAYDUF,^115,116^ SOWKOF,^105,117^ RAYZAS,^118^ YISFOW,^119^ NENXAF,^120^ PESWAM,^121^ BUNKOL,^19,20^ UHUCEH,^95^ HEGGAD,^92,93^ NUMZUT.^94^

Conglomerate behavior is not restricted to compounds of academic interest. Materials exhibiting conglomerate behaviors with importance in medicinal chemistry were also compiled (Figure 5), as these compounds have proven industrial interest. The development of a preferential crystallization or spontaneous deracemization protocols of pharmaceuticals will be of interest because of the scalability of crystallization processes, the already present need to find and control crystal polymorphs of the target, and the possibility of removing expensive enantioenriched ligands for transition metal based catalysts from synthetic routes. Similar to the study of natural product conglomerate behavior, the position of the conglomerate can occur at any stage in the synthesis.

Crystal engineering has also been successful in producing conglomerate crystals. Exploring different crystallization methods and conditions can produce conglomerate crystals from structures which previously did not show conglomerate crystallization behaviors. This is a method to remove the probabilistic nature of conglomerate formation and allow for more control over which substrates display this behavior. The use of crystal engineering can be used to formulate cocrystallization conditions which lead to conglomerate crystal structures (HEGGAD,^92,93^ NUMZUT,^94^ UHUCEH,^95^ and others^96−99^), while retaining favorable biophysical properties. For better or worse, this may also offer a means to evergreen patents on existing pharmaceuticals if a synthetic route is altered to incorporate a conglomerate based asymmetric synthesis or if a final target itself is reformulated to become a conglomerate crystal. The choice of solvent has also been shown to control the formation of a conglomerate crystallization over a racemic crystal.^19,20,100^ The few cases of analysis of both racemic and conglomerate polymorphs of crystals are invaluable case studies for the development of methods to predict and understand conglomerate formation.^101,102^ Cases in which a conglomerate crystal formed a racemic twin are also of interest in further understanding this phenomenon and has been collated in the Supporting Information.

### Potential Applications of Conglomerate Crystallization Behavior

**Figure 6:**
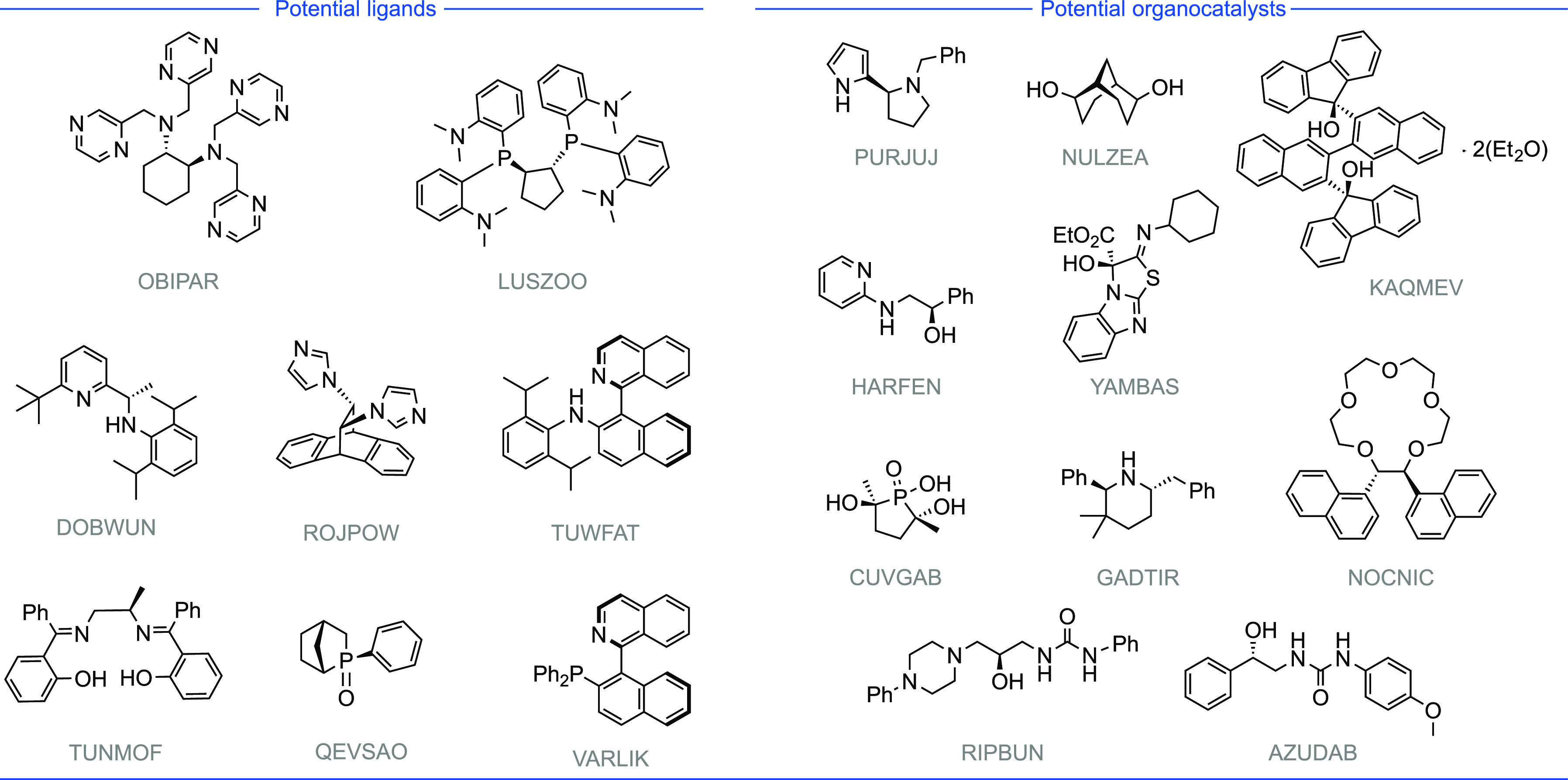
Potential ligands and organocatalysts from conglomerate crystals. The following crystal structures, labeled by their CSD Refcode, were identified as conglomerates: OBIPAR,^122^ LUZOO,^123^ PURJUJ,^135^ NULZEA,^128^ KAQMEV,^167^ DOBWUN,^126^ ROJPOW,^124^ TUWFAT,^125^ HARFEN,^134^ YAMBAS,^133^ CUVGAB,^130^ GADTIR,^168^ NOCNIC,^129^ TUNMOF,^127^ QEVSAO,^169^ VARLIK,^170^ RIPBUN,^131^ AZUDAB.^132^

From surveying the full list of conglomerate crystals, it is possible to identify structures of interest for future applications. Structures with potentially broad utility as chiral ligands and organocatalysts are shown in Figure 6. This highlights the possibility of utilizing conglomerate crystallization as a new chiral pool to provide chemists with sources of chiral information for asymmetric catalysis. Within this list are *C*_2_ symmetrical pyrimidine (OBIPAR^122^), phosphine (LUSZOO^123^), and imidazole (ROJPOW^124^) ligands, an atropisomeric quinoline (TUWFAT^125^), an α-methylpyridine (DOBWUN^126^), and a chiral salen ligand (TUNMOF^127^). Potential types of organocatalysts such as the *C*_2_ symmetrical diol (NULZEA^128^), a PTC (phase transfer catalyst) crown ether (NOCNIC^129^), a chiral phosphoric acid (CUVGAB^130^), chiral ureas (RIPBUN,^131^ AZUDAB^132^), benzotetramisole (YAMBAS^133^), amino-alcohol (HARFEN^134^), and imidazole (PURJUJ^135^) may also find use in asymmetric synthesis. These are only selected examples, and we would encourage the community to view our full list of structures to ascertain which compounds they may deem useful to their research. We acknowledge that the concepts of conglomerate crystallization, preferential crystallization and spontaneous asymmetric synthesis are not original to this paper. Research groups who are aware of the utility of conglomerate crystallization search for chiral structures which crystallize in this manner and which also contain stereocenter(s) suitable for racemization. Once these candidates are identified, spontaneous deracemization protocols have been developed, allowing for the enantioenrichment of materials without conventional forms of asymmetric synthesis. To develop such a spontaneous deracemization protocol, conglomerate crystallization conditions must be unified with racemization conditions such that both may occur simultaneously. Multiple strategies have been employed to allow for solution phase racemization while simultaneously crystallizing the target compound, including base catalysis,^136−147^ acid catalysis,^148^ reversible reactions (such as the Mannich,^149^ aldol,^150,151^ Diels–Alder,^152,153^ [2,3]-sigmatropic rearrangements,^154^ annulation^155^ reactions), Schiff base formation,^156−158^ photoracemization,^159^ and thermal racemization (such as crystallizing from a melt^160^). With this established, a chiral symmetry breaking event is introduced to allow the system to spontaneously enantioenrich. When conducted in an unbiased fashion, as with attrition/grinding^161−163^ (Viedma ripening) and ultrasound,^164^ stochastically enantioenriched material can be produced. Alternatively, these strategies can also be biased using crystal seeding^17,165^ or CPL,^166^ allowing for selection of the enantiomer to be formed from crystallization. Researchers working at the interface of crystallography and synthesis have succeeded in achieving impressive examples of spontaneous deracemization and expanding the protocols available to do so, but have been hindered by the lack of documentation of chiral conglomerate species. We present this curated list of conglomerate crystals for the benefit of both the crystallographic and synthetic communities to unlock the potential of this powerful strategy.

### Future Outlook

Questions on the utility of this curated list of conglomerate crystals may arise:

1. *Why should synthetic chemists care about a crystallographic phenomenon?* It is a phenomenon that can directly affect the enantiopurity of crystalline materials. If a recrystallization had been performed as a purification step on a racemic material which exhibited conglomerate behavior, selection of a conglomerate crystal from this material not only would give different diffraction properties in SCXRD and PXRD compared to its racemate, but also would affect the recorded melting point, IR spectra, Raman spectra, and interactions with other enantioenriched species, such as those encountered in biological and pharmaceutical studies (IC_50_, LD_50,_ protein binding, pharmacokinetics, pharmacodynamics).
2. *How are conglomerate crystals synthetically useful?* The curation of this list of conglomerates should not only aid future research on understanding this fundamental crystallization phenomenon, but also act as a potential source of chiral information for the synthetic community. By tracking materials which undergo this type of crystallization, the possibility of exploiting a powerful mode of chiral amplification can be achieved, whereby a substrate is able to bias its own enantioenrichment.

**Figure 7:**
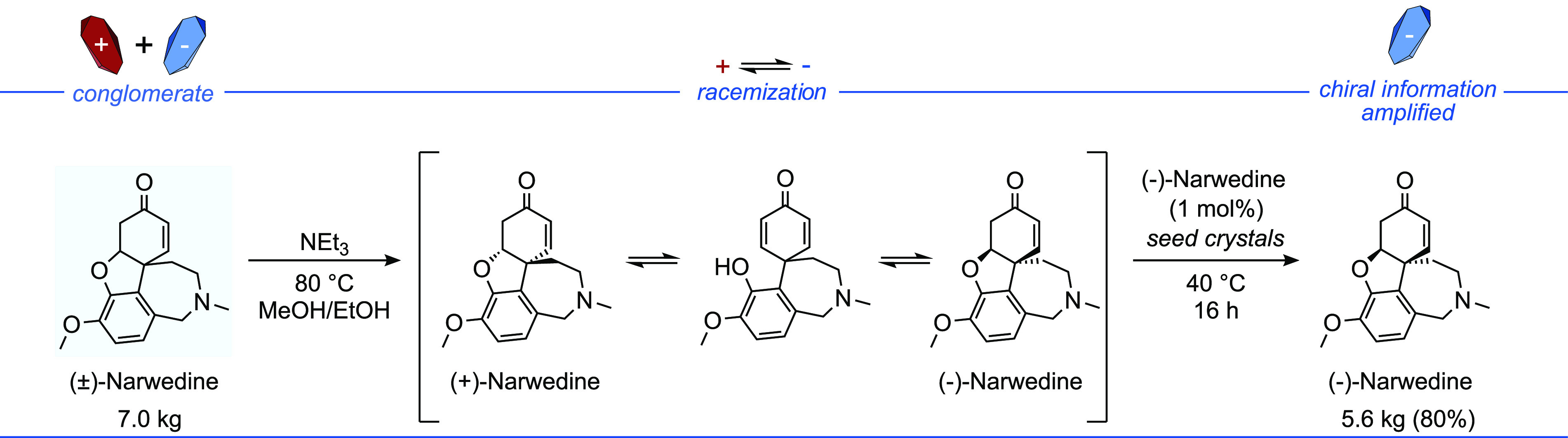
Pilot scale spontaneous deracemization of Narwedine.^17^

The exciting synthetic potential of conglomerate crystallization has been demonstrated in the case of the natural product Narwedine.^17^Figure 7 highlights the process that was developed on a pilot scale, showing this strategy in asymmetric synthesis can reliably produce desired enantioenriched materials in a cost-effective manner for industrial syntheses.^165^

**Figure 8:**
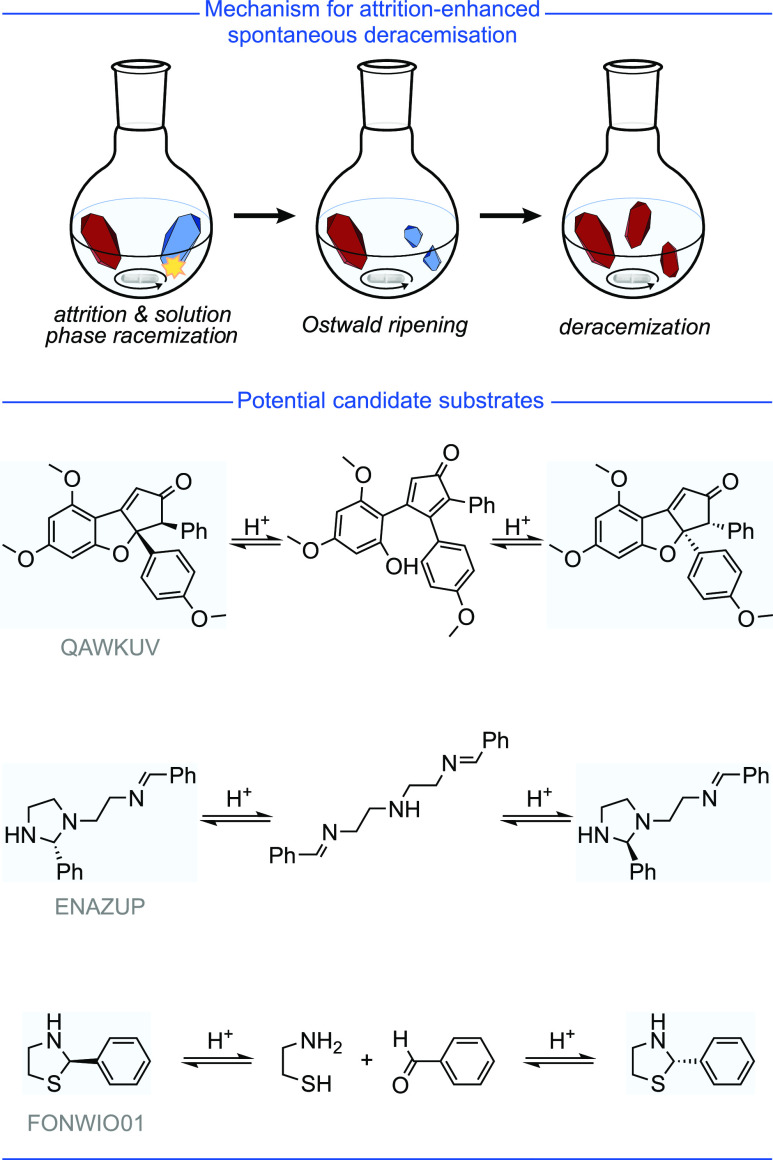
Mechanism of attrition-enhanced deracemization and hypothesized candidates.

The prospective new chiral pool of conglomerate crystals disclosed in the Supporting Information contains a huge variety of structural diversity, with each member being a potential target for spontaneous asymmetric synthesis. A selection of candidates and their hypothesized deracemization conditions are proposed within Figure 8. Armed with a full knowledge of the chemical structures of compounds able to undergo a conglomerate crystallization, we hope that the synthetic and crystallographic communities take advantage of this exciting opportunity to view the structures in this list and use their creativity to develop conditions to exploit this untapped source of chiral information.

## Conclusion

A list of over 1800 conglomerate crystals has been compiled from the CSD and literature, representing 38% of the predicted chiral conglomerate compounds contained within the CSD. Incentivizing synthetic chemists to rapidly communicate their crystal structures with a description of the synthetic procedures and reagents which produced the material—even if such crystals are considered unremarkable by the crystallographic community or the synthesis unremarkable to the synthetic community—is the best method to discover new conglomerate crystals. A simple change in the deposition process to the CSD, which could prompt the synthetic chemist/crystallographer to consider if the material originated from a racemic process, would avoid the need to conduct arduous manual searches in the future. We propose that this list of chiral conglomerate crystals could be viewed as a fundamentally new type of chiral pool; one which is not bound to biologically sourced chiral information. We hope that the curation of this list of conglomerate crystals aids the development of preferential crystallization and spontaneous deracemization protocols, while also furthering the understanding of the formation of conglomerate crystal behavior.

## Experimental Section

CSD version 5.41 (November 2019) was used for the search. Search queries were generated using *Conquest*, with the following queries chosen to try and minimize the total number of crystals to be checked while also maximizing the potential number of conglomerate candidates. Crystals must exist in Sohncke space group *AND* Z′ = 1. Crystals must *NOT* be in carbohydrate, steroid, peptide, or nucleoside/nucleotide classes. Entries must have a carbon center with C(Nonmetal)_4_*OR* H–C(Nonmetal)_3_. The main focus was put on carbon stereocenters since they make up 98% of all stereocenters within in the CSD. The search was refined such that crystals must be organic, not polymer, not salts, and single crystal only, with *R*_1_ < 0.075 and no errors. Disordered structures were allowed. It was also found that specific strings of text could be used to exclude certain natural products, including: “isolated”, “sourced from”, “extracted”, “bark”, “marine”, “sponge”, and “penicillium”. Natural products could be further filtered when sorting the resulting CSD hits by their structure names; generic naming such as “cinchonine”, “strychnine”, and “Striatin A” could be excluded due their natural sources or as targets for asymmetric total syntheses. This generated a list of 30,204 crystals as potential conglomerates. Compounds listed with known stereochemical assignments could be excluded from the list as well. Compound names with the following: (+), (−), d, l, (*R*), and (*S*), were removed from the list, as these were either sourced from the natural chiral pool or were produced from enantioselective methodologies and XRD was used for absolute configuration assignment. This left 21,098 crystals to be inspected manually.

## Notes

The chiral conglomerate crystals identified in this work have been collated as CIF and Refcode list formats (.cif,. txt,. gcd) and are freely available from the *Zenodo* data repository (10.5281/zenodo.6092203).

## Supporting Information Available

Curated list of conglomerate crystals along with their chemical structures and their associated references (PDF)

## Notes

The Python script used to extract the CIF fields information from the CIFs within the internal CSD is available from the *GitHub* repository (https://github.com/walshm78/CSD_conglomerate_search). This manuscript was originally deposited as a preprint on *ChemRxiv* (10.26434/chemrxiv-2022-3g59b).

## Abbreviations

CCDC: Cambridge Crystallographic Data Centre
CIF: crystallographic information file
CSD: Cambridge Structural Database
CPL: circularly polarized light
SCXRD: single crystal X-ray diffraction
PXRD: powder X-ray diffraction
