# Supplementary material for: Identifying a Hidden Conglomerate Chiral Pool in the CSD
Source: JACS Au. 2022 Sep 23;2(10):2235–50. doi: 10.1021/jacsau.2c00394 (PMC9597607; doi:10.1021/jacsau.2c00394)
Supplement: Supplementary file 1 — au2c00394_si_001.pdf [file au2c00394_si_001.pdf]

Supporting Information

Identifying a Hidden Conglomerate Chiral Pool in the CSD

Mark P. Walsh[a*], James A. Barclay[a†], Callum S. Begg[a†], Jinyi Xuan[a†], Natalie T. Johnson[b], Jason C. Cole[b], Matthew O. Kitching[a*]

[a] Department of Chemistry, Durham University, Lower Mount Joy, South Rd, Durham, DH1 3LE, United Kingdom.

[b] Cambridge Crystallographic Data Centre, 12 Union Road, Cambridge, CB2 1EZ, United Kingdom.

[†] These authors contributed equally to this work

* Correspondence to be sent to:

markpwalsh1@gmail.com

matthew.o.kitching@durham.ac.uk

Contents

Search parameters 3

Inaccessible journals 4

Conglomerate crystals found within the CSD 5

Conglomerate crystals found by literature search 91

Conglomerate crystals not available in the CSD 96

Crystals in the CSD which undergo racemic twinning........ 97

Internal search of the CSD 98

Conglomerate crystals confirmed by preferential crystallisation........ 99

References 101

Search parameters

CSD version 5.41 (November 2019) was used for the search. Queries were generated using *Conquest*, with the following queries chosen to try and minimise the total number of crystals to be checked while also maximising the potential number of conglomerate candidates. Crystals must exist in Sohncke space group AND Z' = 1. Must NOT be in carbohydrate, steroid, peptide or nucleoside/nucleotide classes. Must have carbon centre with C(Non-metal)$_4$ OR H-C(Non-metal)$_3$. The main focus was put on carbon stereocentres since they make up 98% of all stereocentres for chiral compounds within in the CSD.

Must be organic, no polymer, single crystal only, $R_1 < 0.075$, no errors, disorder allowed.

It was also found that specific strings of text could be used to exclude certain natural products, including: "isolated", "sourced from", "extracted", "bark", "marine", "sponge", "penicillium". Natural products could be further filtered when sorting the resulting CSD hits by their structure names; generic naming such as "cinchonine", "strychnine", "Striatin A" could be excluded due their natural sources or as targets for asymmetric total syntheses. This generated a list of 30,204 crystals as potential conglomerates.

Compounds listed with known stereochemical assignments could be excluded from the list too. Compound names with the following: (+), (-), D, L, (R) and (S), were removed from the list as these were either sourced from the natural chiral pool or were produced from enantioselective methodologies and XRD was used for absolute configuration assignment. Leaving 21,098 crystals to be sorted manually. Likewise, compounds labelled as a racemate, such as: *rac*, (±), and (D/L) were earmarked as potential conglomerate candidates for further checking.

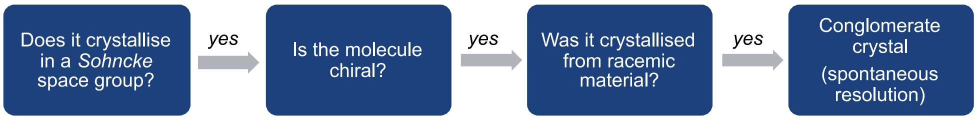

Figure 1 - Flowchart for conditions in the identification of a chiral conglomerate crystal in the CSD

Inaccessible journals

Due to lack of access/online availability, crystals sourced from the following journals could not be checked:

Jiegou Huaxue
Kristallografiya
Izvestiya Akademii Nauk SSSR Seriya Khimicheskaya
Khimiya Geterotsiklicheskikh Soedinenii
Vestnik Moskovskii Universitet
Seriia Khimiia
Zhurnal Obshchei Khimii
Sibirskogo Khimicheskaia Zhurnal
Zhongshan Daxue Xuebao Ziran Kexueban
Khimiia Prirodnykh Soedinenii
Youji Huaxue
Gazzetta Chimica Italiana
Revue Roumaine de Chimie
Izvestiya Minist.Nauki- Akademii Nauk Resp. Kazakhskoi, Seriia Khimicheskaya
Chemical Research in Chinese Universities
Izv. Jug. Cent. Krist., Ser. A
Doklady Akademii Nauk SSSR
Warasan Wichai Mahawitt.Thaksin
Xiamen Daxue Xuebao,Ziran Kexueban
Letters in Organic Chemistry
Tennen Yuki Kagobutsu Toronkai Koen Yoshishi
Recueil des Travaux Chimiques des Pays-Bas
Doklady Akademii Nauk SSSR
Revista Latinoamericana de Quimica
Collection of Czechoslovak Chemical Communications
Latv. PSR Zinat. Akad. Vestis. Khim. Ser.
Elektrokhimiya
Bioorganicheskaia Khimiia
Canadian Mineralogist
Memorias do Instituto Oswaldo Cruz
Bulletin of the Institute of Chemistry, Academia Sinica
Journal de la Societe Chimique de Tunisie
Vestnik Akademiya Nauk Respubliki Kazakhstan
Vestnik Moskovskii Universitet, Seriia Khimiia

Conglomerate crystals found within the CSD

CSD Code	Space Group	a / Å	b / Å	c / Å	α / °	β / °	γ / °	Publication Year	Reference
ABIBIY	$P2_12_12_1$	9.928	10.295	17.911	90	90	90	2011	[1]
ABIJED	$R3$	26.426	26.426	7.039	90	90	120	2016	[2]
ABIZUJ	$P2_12_12_1$	9.469	9.7437	24.586	90	90	90	2016	[3]
ABOBAX	$P3_1$	10.386	10.386	19.468	90	90	120	2016	[3]
ABOWUJ	$P2_1$	8.8361	10.252	10.586	90	90.691	90	2001	[4]
ACIFAW	$P2_1$	6.4533	8.15	20.626	90	93.939	90	2017	[5]
ACIRAG	$P2_1$	9.136	5.4923	16.728	90	94.004	90	2006	[6]
ACORER	$P2_1$	7.7576	6.007	14.757	90	93.71	90	2012	[7]
ACORUH	$P2_12_12_1$	6.355	8.01	24.798	90	90	90	2012	[7]
ACOSES	$P2_1$	8.5077	9.6154	9.324	90	116.932	90	2012	[7]
ADISAI	$P2_12_12_1$	7.1251	9.8071	25.221	90	90	90	2007	[8]
ADISIQ	$P2_12_12_1$	5.8041	8.9094	28.773	90	90	90	2007	[8]
ADISUC	$P4_3$	10.523	10.523	15.76	90	90	90	2007	[8]
AFAMID	$P2_12_12_1$	9.975	11.266	19.208	90	90	90	2002	[9]
AFIHUT	$P2_12_12_1$	11.009	11.893	15.698	90	90	90	2007	[10]
AFINOV	$P2_12_12_1$	6.875	8.2248	31.72	90	90	90	2018	[11]
AHOPUI	$P2_1$	9.3386	8.4676	12.045	90	100.4	90	2003	[12]
AJARUA	$P2_1$	7.946	9.4371	9.6309	90	100.506	90	2015	[13]
AJEREM	$P2_12_12_1$	7.439	7.522	13.369	90	90	90	2003	[14]
AKAXOZ	$P2_1$	9.811	7.659	10.687	90	114.155	90	2003	[15]
AKUWAE	$P2_12_12_1$	8.726	14.021	19.071	90	90	90	2003	[16]
ALPMIA	$P2_12_12_1$	17.422	12.777	7.564	90	90	90	1972	[17]
AMDODP10	$P2_12_12_1$	16.756	13.305	7.536	90	90	90	1980	[18]
AMEMAH	$P2_12_12_1$	6.3896	12.918	21.255	90	90	90	2011	[19]
ANOFOZ	$P2_1$	6.891	13.921	8.2	90	105.658	90	2010	[20]
ANOKUL	$P2_1$	10.443	7.4627	14.123	90	93.253	90	2013	[21]
ANOXUX	$P2_12_12_1$	10.132	11.388	12.305	90	90	90	2011	[22]
APAPOX	$P2_12_12_1$	6.0368	13.398	29.789	90	90	90	2011	[23]
APAWIY	$P2_12_12_1$	7.048	8.141	17.981	90	90	90	2010	[24]
APENTN02	$P2_12_12$	12.173	13.548	6.494	90	90	90	1999	[25]
ARAHAD	$P2_1$	10.433	13.814	10.471	90	114.652	90	2010	[26]
ASEGOW	$P2_12_12_1$	8.0275	11.093	17.321	90	90	90	2016	[27]
ASORIK	$P2_12_12_1$	10.439	11.393	19.763	90	90	90	2011	[28]
ATCTZC	$P2_12_12_1$	10.99	12.37	9.22	90	90	90	1978	[29]
AVAJEM	$P3_2$	9.4131	9.4131	9.467	90	90	120	2004	[30]
AVINID	$P2_12_12_1$	24.508	6.2205	7.2839	90	90	90	2011	[31]
AVUHEG	$P2_12_12_1$	6.8411	10.607	17.583	90	90	90	2017	[32]
AXEGOB	$R3$	19.804	19.804	6.6167	90	90	120	2016	[33]
AXIJOH	$P2_12_12_1$	5.7762	9.6032	28.672	90	90	90	2011	[34]
AXIXOV	$P2_1$	8.3353	5.9743	16.187	90	102.296	90	2011	[35]
AXOQOU	$P2_1$	8.154	7.971	12.393	90	104.93	90	2011	[36]
AXOREL	$P2_12_12_1$	6.4801	15.459	19.189	90	90	90	2011	[37]
AYAGIS	$P6_5$	9.3889	9.3889	19.095	90	90	120	2016	[38]
AZAHAL	$P2_1$	9.1	5.4032	10.452	90	108.139	90	2011	[39]
AZIDES	$P2_1$	6.0127	10.704	9.6578	90	100.24	90	2004	[40]
AZITEI	$P2_12_12_1$	5.8532	14.544	19.327	90	90	90	2004	[41]
AZOVIV	$P2_12_12_1$	9.7948	10.936	22.324	90	90	90	2011	[42]
AZUDAB	$P2_1$	6.812	8.7659	12.139	90	97.009	90	2011	[43]
AZUXUP	$P2_1$	9.014	10.242	9.289	90	108.194	90	2011	[44]

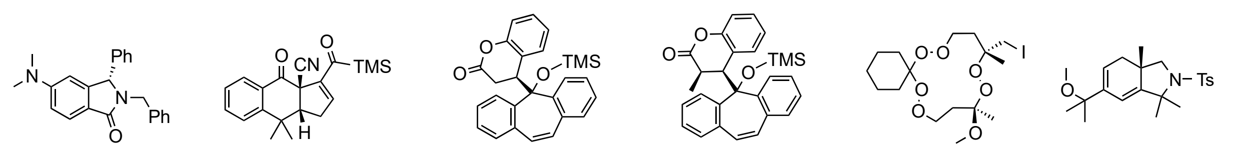

ABIBIY ABIJED ABIZUJ ABOBAX ABOWUJ ACIFAW

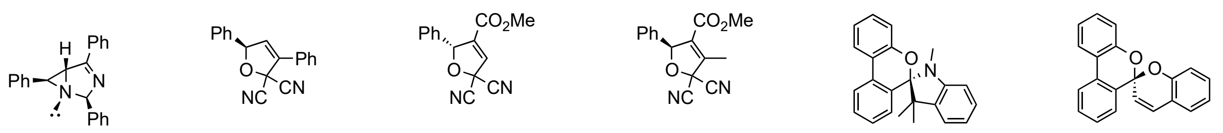

ACIRAG ACORER ACORUH ACOSES ADISAI ADISIQ

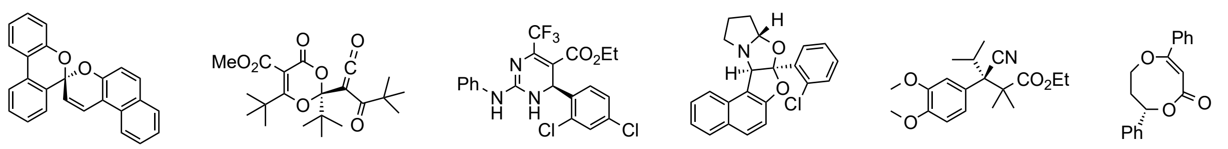

ADISUC AFAMID AFIHUT AFINOV AHOPUI AJARUA

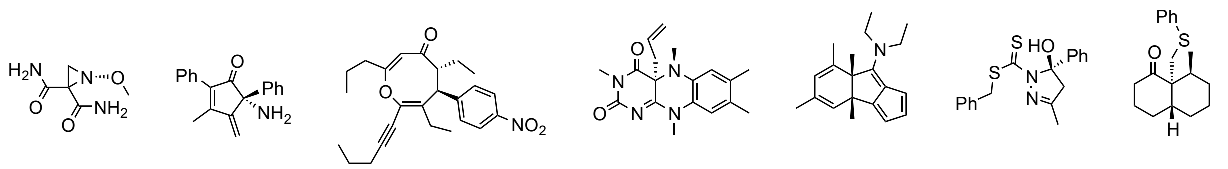

AJEREM AKAXOZ AKUWAE ALPMIA AMDODP10 AMEMAH ANOFOZ

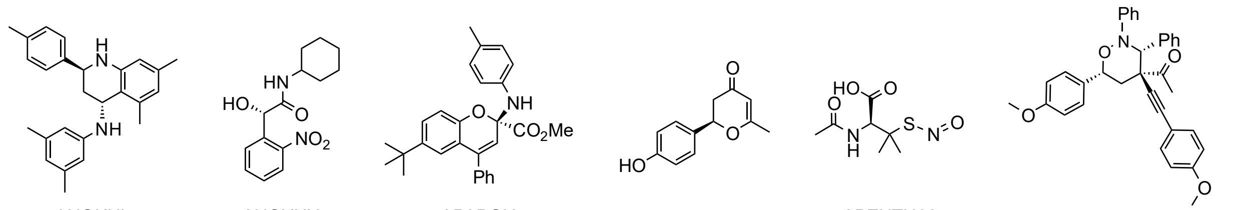

ANOKUL ANOXUX APAPOX APAWIY APENTN02 ARAHAD

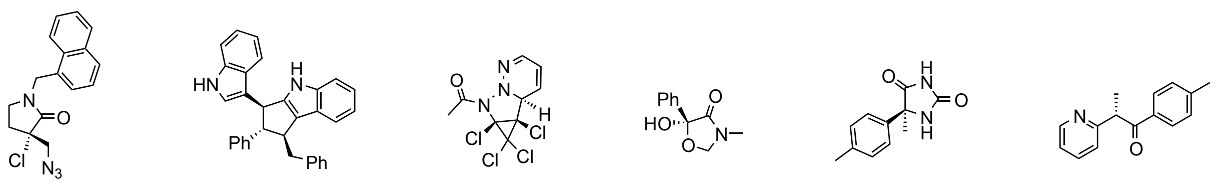

ASEGOW ASORIK ATCTZC AVAJEM AVINID AVUHEG

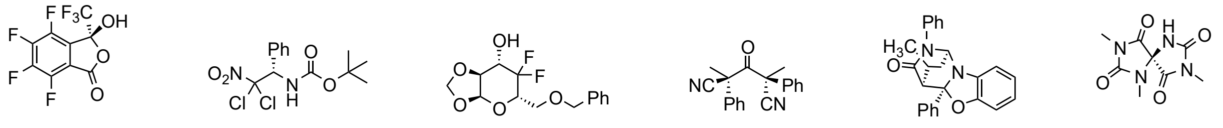

AXEGOB AXIJOH AXIXOV AXOQOU AXOREL AXOREL

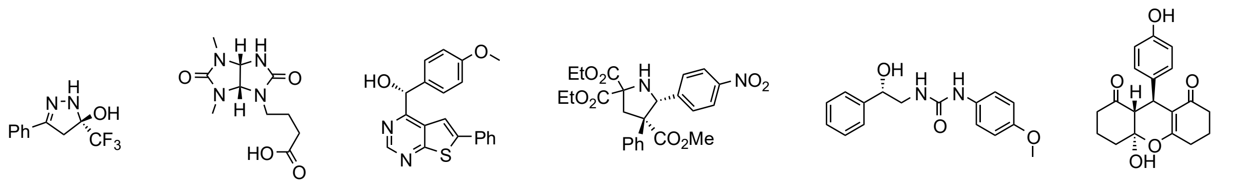

AZAHAL AZIDES AZITEI AZOVIV AZUDAB AZUXUP

CSD Code	Space Group	a / Å	b / Å	c / Å	α / °	β / °	γ / °	Publication Year	Reference
BADKOI	$P2_12_12_1$	6.778	8.5059	19.645	90	90	90	2010	45
BAFYIQ	$P2_12_12_1$	6.814	6.905	25.579	90	90	90	1998	46
BAGMAA	$P2_1$	11.734	7.397	12.645	90	107.317	90	2017	47
BAGMAY	$P2_1$	9.8688	7.0875	11.42	90	101.821	90	2002	48
BAHMUS	$P2_12_12_1$	10.094	12.845	10.776	90	90	90	1981	49
BAHNAZ	$P2_12_12_1$	10.645	15.266	8.166	90	90	90	1981	49
BAHQEG	$P2_12_12_1$	7.0306	8.0766	14.135	90	90	90	1998	50
BALLOS	$P2_1$	6.3021	15.876	9.4266	90	103.133	90	2017	51
BALSOW	$P2_1$	7.086	7.817	9.227	90	93.1	90	1981	52
BANBOJ	$P2_12_12_1$	8.03	9.784	27.646	90	90	90	2011	53
BANMEI	$P2_12_12_1$	5.857	12.388	12.633	90	90	90	1981	54
BASZAX	$P2_12_12_1$	10.665	12.506	8.5077	90	90	90	2003	55
BASZEB	$P2_12_12_1$	10.513	11.889	7.965	90	90	90	2003	55
BASZIF	$P2_12_12_1$	11.389	12.106	7.087	90	90	90	2003	55
BAVKIU	$P2_1$	5.5386	7.0291	23.255	90	93.015	90	2012	56
BAYDAI	$P2_1$	8.3826	8.2887	10.034	90	107.865	90	2012	57
BAZGEP	$P2_12_12_1$	8.453	9.46	13.44	90	90	90	2003	58
BAZREC	$P2_12_12_1$	8.2625	9.6405	17.89	90	90	90	2017	59
BAZWII	$P2_12_12_1$	6.196	19.898	13.212	90	90	90	1982	60
BCMSFA	$P2_12_12_1$	6.865	7.275	25.193	90	90	90	1980	61
BEBDET	$P2_12_12_1$	4.2571	14.425	23.481	90	90	90	2012	62
BEFCUM	$P2_1$	6.9724	8.4812	12.596	90	103.294	90	2012	63
BEFROT	$P2_12_12_1$	11.598	9.382	13.985	90	90	90	1982	64
BEJJIL	$P2_1212$	10.589	21.09	7.5101	90	90	90	2012	65
BEPHUW	$P2_12_12_1$	12.771	11.983	6.831	90	90	90	1982	66
BEPHUX	$P2_12_12_1$	12.88	11.886	6.828	90	90	90	1982	66
BEPHUY	$P2_12_12_1$	12.382	12.285	6.823	90	90	90	1982	66
BEPHUZ	$P2_12_12_1$	13.073	11.745	6.838	90	90	90	1982	66
BEPPUI	$P2_1$	8.0665	7.3516	13.338	90	102.183	90	2004	67
BEWFEP	$P2_12_12_1$	9.0449	11.435	18.083	90	90	90	2004	68
BEWMIZ	$C2$	17.922	8.4543	14.047	90	117.39	90	1998	69
BIHDUT	$P2_12_12_1$	6.5595	13.88	15.428	90	90	90	2013	70
BIRLUJ	$P2_12_12_1$	7.385	10.08	16.714	90	90	90	1982	71
BISSEE	$P2_12_12_1$	7.1064	10.316	15.973	90	90	90	2018	72
BIXLIG	$P2_12_12_1$	5.5132	9.3465	31.769	90	90	90	2018	73
BIZCIZ	$P2_12_12_1$	7.5177	12.476	16.118	90	90	90	2019	74
BOBTOD	$P2_12_12_1$	40.858	6.6959	9.2019	90	90	90	2013	75
BOCRUJ	$P2_1$	7.9875	13.69	9.6936	90	100.512	90	2019	76
BOCSOE	$P3_2$	12.851	12.851	11.785	90	90	120	2019	76
BOJPEY	$P2_12_12_1$	9.9355	14.257	19.383	90	90	90	2019	77
BOMLEW	$P2_12_12_1$	5.5015	9.707	23.399	90	90	90	2014	78
BONNOK	$P2_12_12_1$	8.3879	11.964	17.74	90	90	90	2018	79
BOPXOV	$P2_12_12_1$	5.8078	13.642	19.79	90	90	90	2014	80
BOPYAI	$P2_12_12_1$	5.678	13.848	21.308	90	90	90	2014	80
BOQKUQ	$P2_12_12_1$	7.6411	9.7095	30.167	90	90	90	2019	81
BOQREG	$P2_12_12_1$	10.047	11.306	12.199	90	90	90	2014	82
BOQYAJ	$P2_12_12_1$	9.1115	9.647	19.486	90	90	90	2014	83
BPTZTD	$P2_1$	6.991	10.497	10.452	90	98.1	90	1979	84
BUBSAT	$P2_12_12_1$	9.636	11.622	15.13	90	90	90	2009	85
BUDNUL	$P2_12_12_1$	6.898	13.722	19.927	90	90	90	2014	86
BUDTEA	$P2_12_12_1$	7.3658	14.801	17.456	90	90	90	2009	87,88
BUFLAQ	$P2_12_12_1$	9.78	14.859	20.466	90	90	90	2009	89

CSD Code	Space Group	a / Å	b / Å	c / Å	α / °	β / °	γ / °	Publication Year	Reference
BUHMOH	$P2_12_12_1$	8.9782	9.2239	13.174	90	90	90	2009	[90]
BUNKOL	$P2_12_12_1$	10.333	7.229	21.287	90	90	90	1983	[91,92]
BUNROT	$P2_12_12_1$	7.2261	13.425	18.999	90	90	90	2015	[93]
BUVGAC	$P2_1$	7.636	6.117	9.257	90	93.691	90	2014	[94]
BUVHEG	$P2_12_12_1$	9.6305	10.334	17.818	90	90	90	2010	[95]
BUWBIG	$P2_12_12_1$	5.9332	10.02	20.968	90	90	90	2015	[96]
BUZLIR	$P2_1$	7.445	10.865	13.339	90	100.69	90	2001	[97]

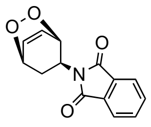
BADKOI
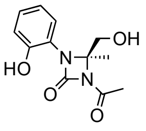
BAFYIQ
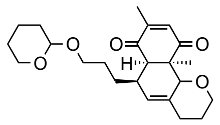
BAGMAA
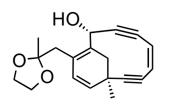
BAGMAY
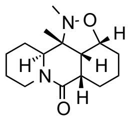
BAHMUS
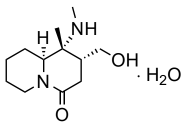
BAHNAZ
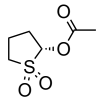
BAHQEG
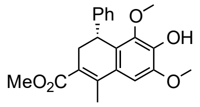
BALLOS
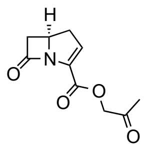
BALSOW
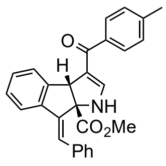
BANBOJ
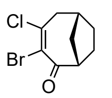
BANMEI
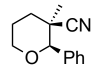
BASZAX
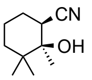
BASZEB
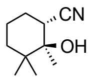
BASZIF
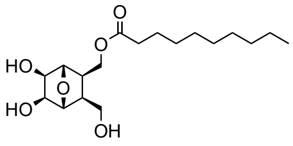
BAVKIU
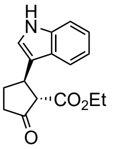
BAYDAI
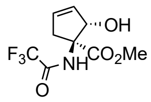
BAZGEP
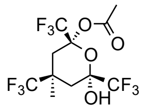
BAZREC
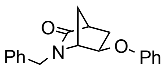
BAZWII
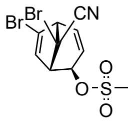
BCHMSHFA
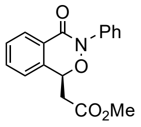
BEBDET
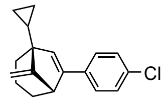
BEFCUM
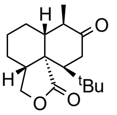
BEFROT
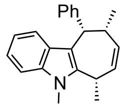
BEJJIL
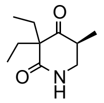
BEPHUW/BEPHUZ
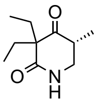
BEPHUX/BEPHUY
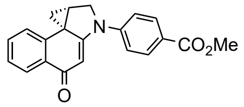
BEPPUI
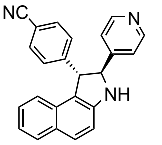
BEWFEP
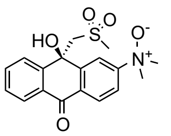
BEWMIZ
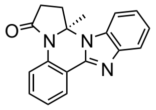
BIHDUT
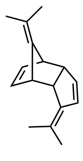
BIRLUJ
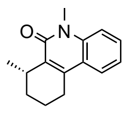
BISSEE
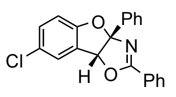
BIXLIG
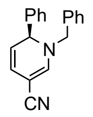
BIZCIZ
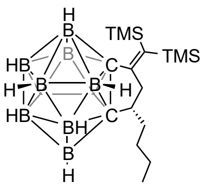
BOBTOD
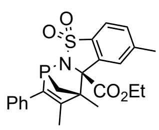
BOCRUJ
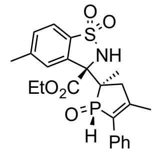
BOCSOE
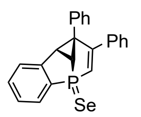
BOJPEY
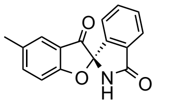
BOMLEW
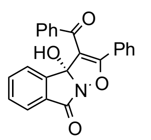
BONNOK

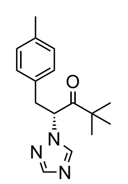

BOPXOV

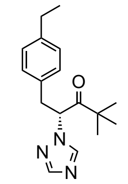

BOPYAI

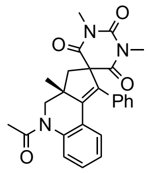

BOQKUQ

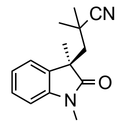

BOQREG

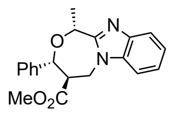

BOQYAJ

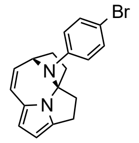

BPTZTD

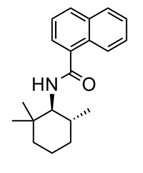

BUBSAT

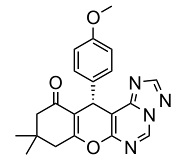

BUDNUL

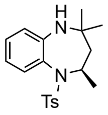

BUDTEA

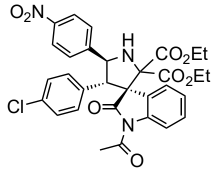

BUFLAQ

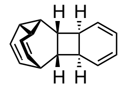

BUHMOH

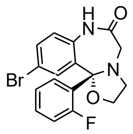

BUNKOL

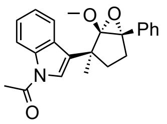

BUNROT

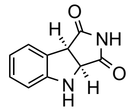

BUVGAC

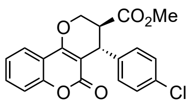

BUVHEG

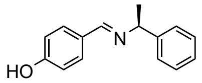

BUWBIG

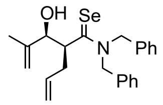

BUZLIR

CSD Code	Space Group	a / Å	b / Å	c / Å	α / °	β / °	γ / °	Publication Year	Reference
CAJCIZ	$P2_12_12_1$	12.964	14.814	10.273	90	90	90	1983	98
CALSUG	$P2_1$	9.9447	9.7882	14.404	90	99.979	90	2017	99
CANJOQ	$P2_12_12_1$	5.799	14.745	23.711	90	90	90	1983	100
CAPVEX	$P2_12_12_1$	10.263	16.29	18.859	90	90	90	2017	101
CAVGIQ	$C2$	16.412	5.944	14.762	90	95.14	90	2005	102
CAWNIZ	$P2_1$	7.9538	7.3938	14.442	90	91.551	90	2012	103
CAZRAY	$P2_1$	10.673	8.677	12.475	90	90.403	90	2012	104
CEBLAW	$P2_12_12_1$	12.109	22.916	7.678	90	90	90	1983	105
CEBNIG	$P2_12_12_1$	9.954	11.177	7.107	90	90	90	1983	106
CECTEL	$P2_12_12_1$	7.3176	13.931	16.375	90	90	90	2012	107
CEDZUH	$P2_1$	9.088	13.637	10.917	90	92.543	90	2005	108
CEFKIH	$P2_12_12_1$	8.843	10.396	21.687	90	90	90	1984	109
CEGLOS	$P2_1$	11.869	10.467	13.409	90	105.094	90	2017	110
CEHGOO	$P6_1$	9.8322	9.8322	47.953	90	90	120	2017	111
CEJWUM	$P2_1$	7.4542	7.4534	9.975	90	101.466	90	2017	112
CELNUC	$P2_12_12_1$	34.069	7.039	7.093	90	90	90	1984	113
CELPAK	$P2_12_12_1$	20.091	9.187	9.331	90	90	90	1984	113
CEMIND	$P4_1$	11.493	11.493	11.013	90	90	90	1980	114
CEMJAF	$P2_12_12_1$	7.9	14.751	18.956	90	90	90	1999	115
CEQPOE	$P2_12_12_1$	12.096	15.231	8.4361	90	90	90	2005	116
CEQYAA	$P2_1$	10.534	9.49	13.066	90	95.457	90	2012	117
CESPUM	$P2_12_12_1$	7.9691	10.742	29.44	90	90	90	2006	118
CEWCEP	$P2_12_12_1$	4.8838	15.157	17.658	90	90	90	2017	119
CEXYOT	$P2_12_12_1$	6.887	13.042	13.257	90	90	90	1984	120
CIBGAX	$P2_12_12_1$	7.8795	10.879	19.733	90	90	90	2013	121
CIBKUT	$P2_1$	9.635	8.5322	14.163	90	100.111	90	1999	122
CICSOX	$C2$	23.614	5.698	14.232	90	96.557	90	2007	123
CIKNEP	$P2_12_12_1$	5.916	11.585	21.986	90	90	90	1984	124
CIKNES	$P2_12_12_1$	8.1939	11.162	14.544	90	90	90	2018	125
CIPPUO	$P2_1$	9.3753	7.8455	10.809	90	113.902	90	2013	126
CIPREA	$P2_12_12_1$	6.1571	7.7921	31.207	90	90	90	2013	127
CIQCAG	$P2_1$	6.015	15.293	7.479	90	99.93	90	1999	128
CISJAP	$P2_1$	6.238	10.321	7.796	90	91.79	90	1984	129
CITMAT	$P4_1212$	10.542	10.542	25.817	90	90	90	1984	130
COBQES	$P2_12_12_1$	10.751	11.169	11.938	90	90	90	2019	131
COCBOM	$P2_12_12_1$	7.339	9.993	20.287	90	90	90	2008	132
COJXUX	$P2_12_12_1$	9.6058	15.773	17.665	90	90	90	2019	133
COQLUP	$P2_12_12_1$	9.304	14.95	15.712	90	90	90	1997	134
COVJOO	$P2_1$	6.708	8.388	12.407	90	103.322	90	2014	135
CPHINO	$P2_12_12_1$	10.698	10.252	15.259	90	90	90	1972	136
CPMOIC10	$P2_12_12_1$	13.21	13.8	7.33	90	90	90	1969	137
CUBSID	$P2_1$	12.626	5.511	13.381	90	107.086	90	2014	138
CUPGAW	$P6_5$	10.306	10.306	33.251	90	90	120	2009	139
CUPWOZ	$P2_12_12_1$	9.34	8.34	16.342	90	90	90	1985	140
CURJAB	$P2_12_12_1$	7.5812	13.699	15.145	90	90	90	2008	141
CURTAM	$P2_12_12_1$	8.6906	11.953	13.546	90	90	90	2015	142
CUVGAB	$P2_12_12_1$	6.743	10.419	12.406	90	90	90	1985	143
CUVJOT	$P2_1$	8.6005	9.9189	11.375	90	91.36	90	2010	144
CXPTDZ	$P2_12_12_1$	7.9596	27.763	6.4878	90	90	90	1979	145

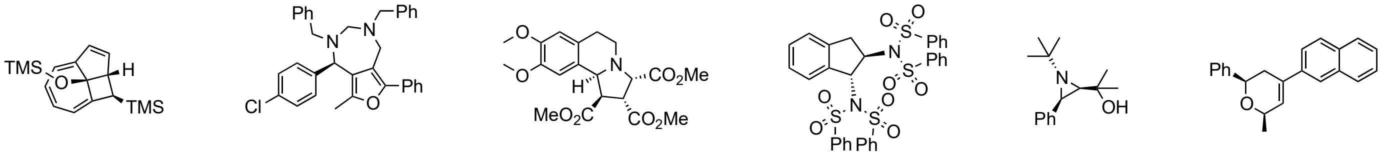

CAJCIZ CALSUG CANJOQ CAPVEX CAVGIQ CAWNIZ

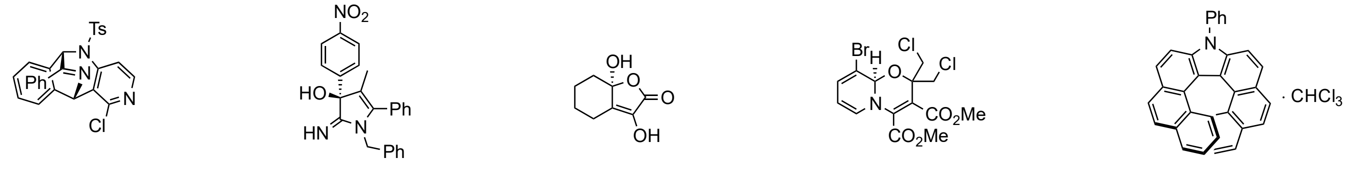

CAZRAY CEBLAW CEBNIG CECTEL CEDZUH

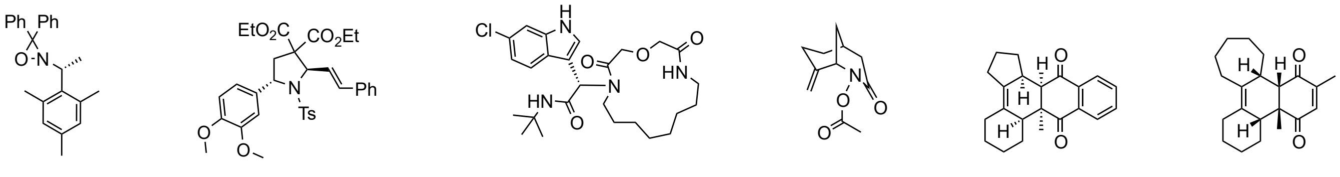

CEFKIH CEGLOS CEHGOO CEJWUM CELNUC CELPAK

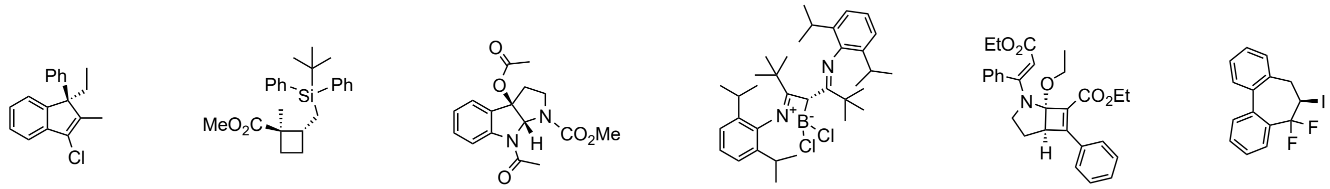

CEMIND CEMJAF CEQPOE CEQYAA CESPUM CEWCEP

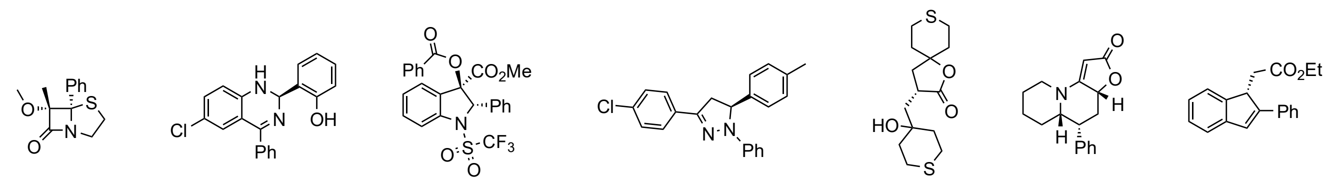

CEXYOT CIBGAX CIBKUT CICSOX CIKNEP CIKNES CIPPUO

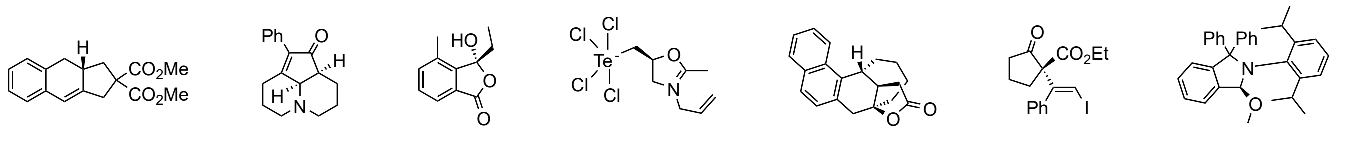

CIPREA CIQCAG CISJAP CITMAT COBQES COCBOM COJXUX

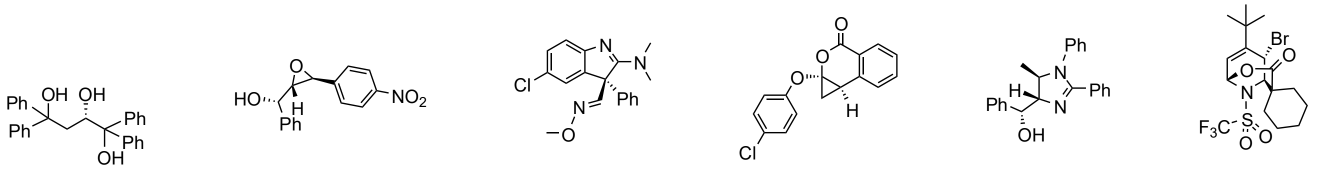

COQLUP COVJOO CPHINO CPMOIC10 CUBSID CUPGAW

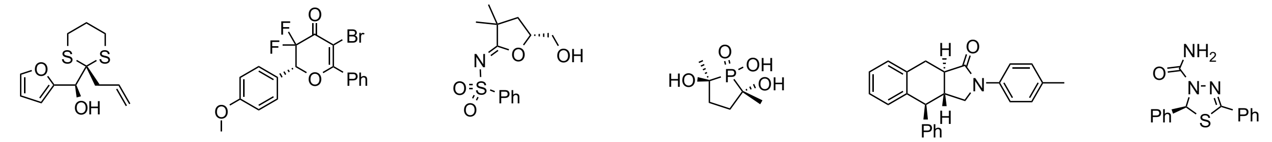

CUPWOZ CURJAB CURTAM CUVGAB CUVJOT CXPTDZ

CSD Code	Space Group	a / Å	b / Å	c / Å	α / °	β / °	γ / °	Publication Year	Reference
DACPII	$P2_1$	10.602	7.3935	10.975	90	102.931	90	2011	146
DACVOU	$P2_12_12_1$	10.445	14.899	16.596	90	90	90	2011	147
DADRAD	$P2_1$	5.8422	8.1742	16.949	90	90	90	2011	148
DAJLEG	$P2_12_12_1$	11.21	13.483	16.459	90	90	90	2004	149
DALSIS	$P2_12_12_1$	17.48	10.99	13.87	90	90	90	1985	150
DAPHEH	$P2_1$	10.933	7.855	10.185	90	102.74	90	1984	151
DASDAD	$P2_12_12_1$	6.1898	13.115	18.506	90	90	90	2005	152
DAXKAP	$P2_12_12_1$	5.068	18.168	10.128	90	90	90	2005	153
DAYTUV	$P2_12_12_1$	9.8509	12.127	14.113	90	90	90	2017	154
DAYYEJ	$P2_1$	6.2543	14.792	9.4637	90	101.721	90	2012	155
DEBKUT	$P2_12_12_1$	7.4193	12.114	17.167	90	90	90	2017	156
DEBMUS	$P2_12_12_1$	12.165	9.964	17.433	90	90	90	1985	157
DEBPAB	$P2_12_12_1$	5.939	14.181	15.323	90	90	90	1985	158
DECKUR	$P2_12_12_1$	7.396	8.107	17.049	90	90	90	1985	159
DEDLIH	$P2_1$	6.982	26.504	8.962	90	116.77	90	1985	160
DEFDIB	$P2_12_12_1$	8.747	8.787	18.173	90	90	90	1985	161,162
DEFXER	$P2_12_12_1$	13.898	7.285	20.704	90	90	90	1985	163
DEGFAZ	$P2_12_12_1$	6.609	11.695	31.395	90	90	90	2017	164
DEJPIR	$P2_12_12_1$	9.741	15.636	17.567	90	90	90	1984	165
DENHAF	$P2_12_12_1$	11.587	14.003	18.056	90	90	90	1985	166
DEPKAL	$P2_12_12_1$	12.205	13.677	17.907	90	90	90	2006	167,168
DEQNOF	$P2_12_12_1$	8.2626	10.718	22.223	90	90	90	2018	169
DERDOU	$C2$	22.973	7.806	12.919	90	91.33	90	2006	170
DESNUL	$P1$	5.4982	7.7104	13.452	82.25	82.83	78.55	2006	171
DESVIG	$P2_12_12_1$	13.802	12.966	8.02	90	90	90	1985	172
DIBFOJ	$P2_12_12_1$	7.872	12.015	12.25	90	90	90	1985	173
DICFOK	$P2_12_12_1$	9.12	15.573	16.676	90	90	90	1985	174
DICMIM	$P2_12_12_1$	9.295	9.482	11.842	90	90	90	2007	175
DIDGUS	$P2_12_12_1$	8.587	11.131	17.118	90	90	90	1985	176
DIGGAE	$P2_1$	5.571	8.229	13.979	90	92.93	90	2017	177
DIHMIR	$P2_12_12_1$	8.5489	12.954	14.829	90	90	90	2007	178
DIHNUD10	$P2_12_12_1$	8.543	10.273	22.812	90	90	90	1987	179
DIHWAT	$P2_1$	9.7085	17.605	10.186	90	113.078	90	2007	180
DIJBEG	$P2_12_12_1$	8.1549	14.391	16.503	90	90	90	2018	181
DIJSIA	$P2_1$	7.759	11.01	9.47	90	111.56	90	2014	182
DILCEG10	$P2_12_12_1$	16.725	12.833	5.939	90	90	90	1986	183
DILKOA	$P2_12_12_1$	6.5665	11.191	22.42	90	90	90	2013	184
DINQID	$P2_1$	6.6658	13.085	7.7113	90	112.529	90	2018	185
DINQUP	$P2_1$	6.6119	12.826	7.7086	90	111.978	90	2018	185
DIPRAX	$P2_12_12_1$	7.7924	12.316	21.732	90	90	90	2013	186
DIPSUT	$P2_1$	5.665	15.58	9.823	90	104.36	90	2018	187
DITMAU	$P2_12_12_1$	6.484	8.502	26.136	90	90	90	1997	188
DIVNAX	$P2_12_12_1$	10.052	12.493	7.992	90	90	90	1986	189
DIWDIW	$C2$	22.764	5.7299	13.316	90	93.101	90	1986	190
DIYYOA	$P2_12_12_1$	5.3281	8.3677	28.943	90	90	90	2008	191
DMUVIC	$P2_12_12_1$	6.16	12.639	12.754	90	90	90	1973	192
DOBFOR	$P2_1$	6.275	11.416	13.018	90	93.845	90	2013	193
DOBHUY	$P2_12_12_1$	6.0708	12.896	22.705	90	90	90	2008	194
DOBWUN	$P4_1$	10.018	10.018	21.453	90	90	90	2008	195
DOGQOF	$P2_1$	7.343	9.018	9.989	90	97.26	90	1999	196
DOGTIC	$P2_12_12_1$	6.603	9.841	10.704	90	90	90	1999	197
DOGVIH	$P2_12_12_1$	11.021	13.606	17.727	90	90	90	2019	198

CSD Code	Space Group	a / Å	b / Å	c / Å	α / °	β / °	γ / °	Publication Year	Reference
DOHWAY	$P2_12_12_1$	5.056	29.019	8.825	90	90	90	1986	[199]
DOJGOB	$P2_12_12_1$	9.4165	9.9677	17.869	90	90	90	2019	[200]
DOKPAW	$P2_1$	6.2534	7.7581	15.535	90	91.251	90	2014	[201]
DOLQAW	$P6_1$	10.014	10.014	34.556	90	90	120	1998	[202]
DONGUI	$P2_12_12_1$	9.066	9.841	17.138	90	90	90	1999	[203]
DORVOX	$P2_12_12_1$	7.5468	9.0436	22.319	90	90	90	2013	[204]
DOTKOM	$P2_1$	10.077	9.228	10.177	90	113.06	90	1986	[205]
DOTMAA	$P2_1$	11.936	6.795	12.005	90	99.92	90	1986	[206]
DOTREJ	$P6_1$	9.385	9.385	19.06	90	90	120	1986	[207]
DOXNUZ	$P2_12_12_1$	7.971	10.676	14.597	90	90	90	1986	[208]
DUFKIY	$P2_12_12_1$	11.58	23.009	8.6162	90	90	90	1986	[209]
DUGYOT	$P2_1$	8.1777	15.479	8.7929	90	110.629	90	2000	[210]
DUJFOE	$P2_12_12_1$	6.6289	10.035	23.203	90	90	90	2009	[211]
DUNFAV	$P2_12_12_1$	11.093	12.712	15.671	90	90	90	2015	[212]
DUPLIL	$P2_1$	9.2351	5.3558	12.909	90	104.113	90	2015	[213]
DUSCUR	$P2_12_12_1$	5.8134	10.721	19.809	90	90	90	2015	[214]
DUTSES	$P2_12_12_1$	10.469	10.515	16.831	90	90	90	2014	[215]
DUVDII	$P2_12_12_1$	5.885	7.5965	28.273	90	90	90	2010	[216]
DUVFOP10	$P2_12_12_1$	6.599	12.028	20.198	90	90	90	1986	[217]
DUVVIZ	$P2_1$	7.087	16.387	10.193	90	91.44	90	1986	[218]
DUXGAF	$P2_12_12_1$	4.997	10.061	24.039	90	90	90	2010	[219]
DUXHIP	$P2_12_12_1$	8.669	14.207	16.694	90	90	90	2016	[220]
DUXHOV	$P2_12_12_1$	8.2095	14.422	17.137	90	90	90	2016	[220]

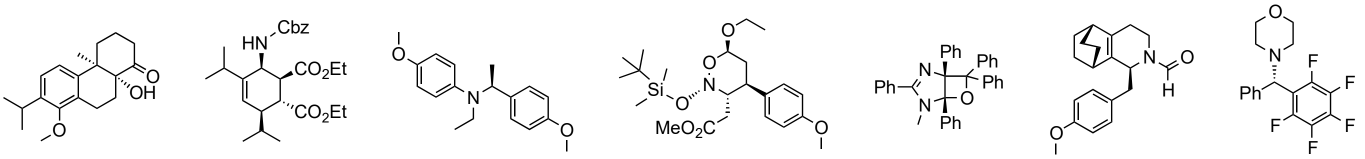

DACPII DACVOU DADRAD DAJLEG DALSIS DAPHEH DASDAD

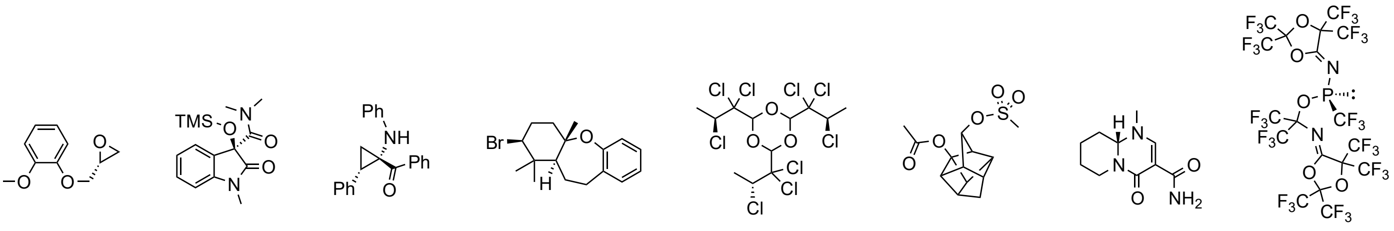

DAXKAP DAYTUV DAYYEJ DEBKUT DEBMUS DEBPAB DECKUR DEDLIH

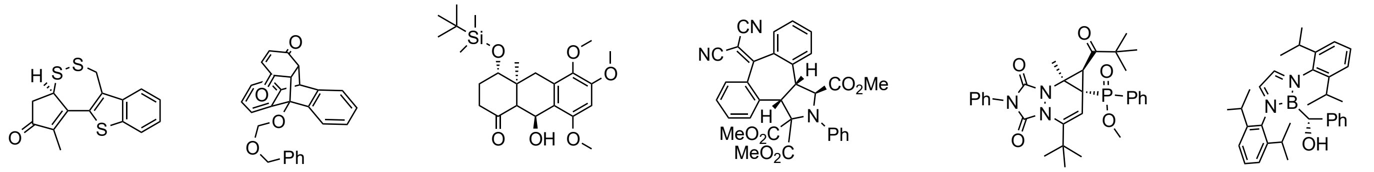

DEFDIB DEFXER DEGFAZ DEJPIR DENHAF DEPKAL

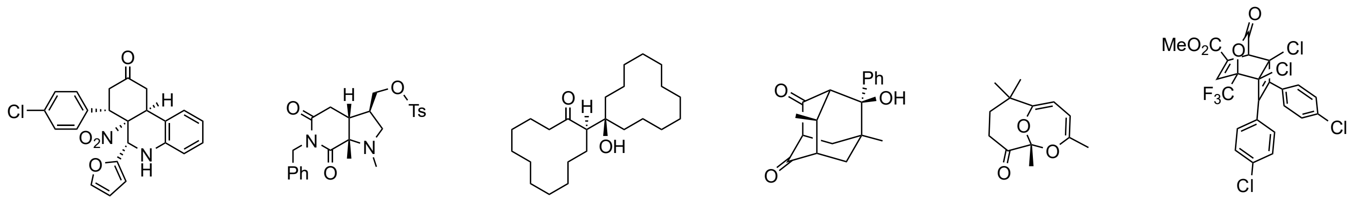

DEQNOF DERDOU DESNUL DESVIG DIBFOJ DICFOK

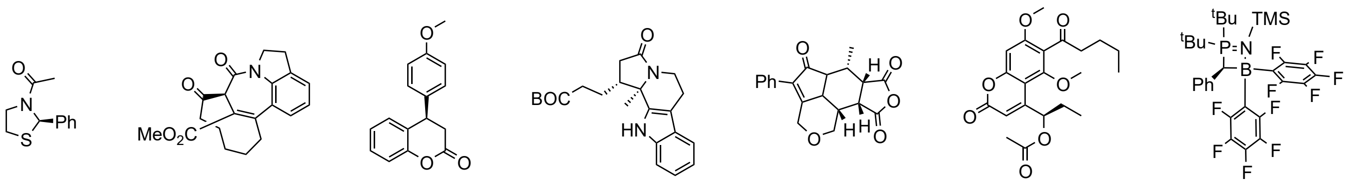

DICMIM DIDGUS DIGGAE DIJBEG DIHMIR DIHNUD10 DIHWAT

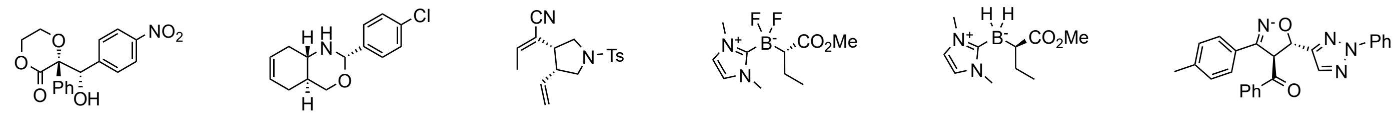

DIJSIA DILCEG10 DILKOA DINQID DINQUP DIPRAX

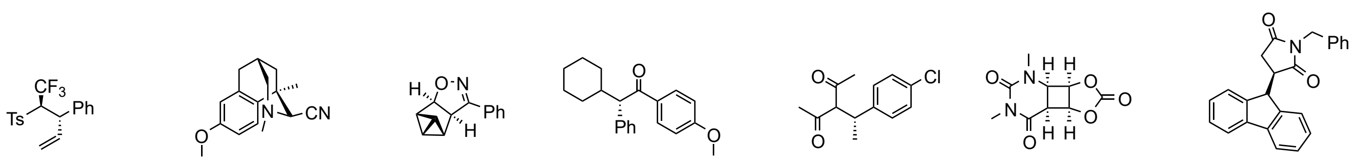

DIPSUT DITMAU DIVNAX DIWDIW DIYYOA DMUVIC DOBFOR

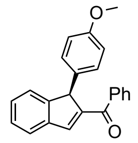

DOBHUY

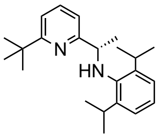

DOBWUN

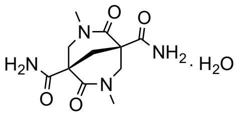

DOGQOF

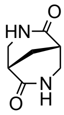

DOGTIC

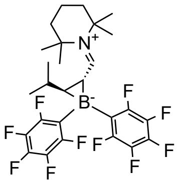

DOGVIH

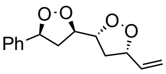

DOHWAY

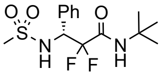

DOJGOB

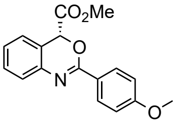

DOKPAW

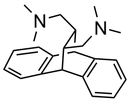

DOLQAW

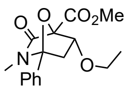

DONGUI

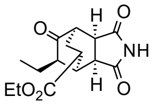

DORVOX

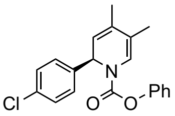

DOTKOM

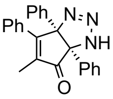

DOTMAA

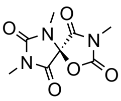

DOTREJ

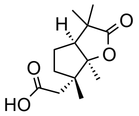

DOXNUZ

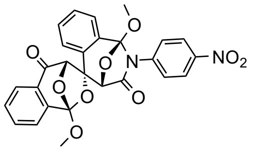

DUFKIY

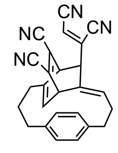

DUGYOT

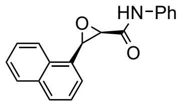

DUJFOE

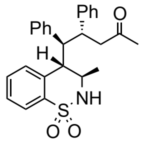

DUNFAV

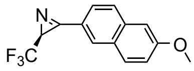

DUPLIL

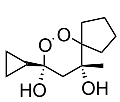

DUSCUR

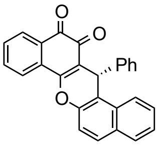

DUTSES

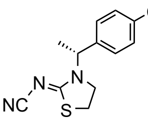

DUVDII

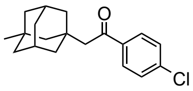

DUVFOP10

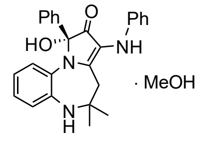

DUVVIZ

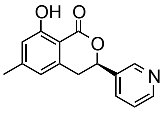

DUXGAF

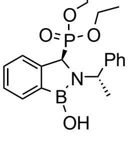

DUXHIP

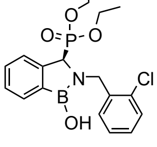

DUXHOV

CSD Code	Space Group	a / Å	b / Å	c / Å	α / °	β / °	γ / °	Publication Year	Reference
EABCEP	$P2_1$	7.515	5.1048	12.144	90	107.02	90	1992	[221]
EACHOF	$P2_12_12_1$	6.521	10.022	19.132	90	90	90	1993	[222]
EBAPUT	$P2_12_12_1$	8.053	10.635	14.657	90	90	90	2004	[223]
EBISEO	$P2_12_12_1$	10.478	13.061	13.561	90	90	90	2004	[224]
ECUPOK	$P2_1$	9.264	11.632	10.797	90	91.753	90	2017	[225]
EDAHAT	$P2_12_12_1$	10.542	12.466	13.862	90	90	90	2007	[226]
EDAQOS	$P2_12_12_1$	10.856	11.023	14.115	90	90	90	2017	[227]
EFEMIM	$P2_12_12_1$	6.486	17.747	19.257	90	90	90	2008	[228]
EGEZUN	$P2_12_12_1$	6.7699	8.2068	15.1	90	90	90	2014	[229]
EGIZEA	$P2_12_12_1$	7.9458	9.34	12.556	90	90	90	2008	[230]
EGOTIG	$P2_12_12_1$	6.8	11.461	12.833	90	90	90	2019	[231]
EGOVAZ	$P2_12_12_1$	5.2734	14.976	18.606	90	90	90	2014	[232]
EHABOH	$P2_12_12_1$	8.2526	9.1103	21.065	90	90	90	2019	[233]
EHATEO	$P2_1$	7.0843	18.153	7.4623	90	106.341	90	2016	[234]
EJUHOG	$P2_12_12_1$	5.734	8.987	15.909	90	90	90	2003	[235]
EJUQUX	$P2_12_12_1$	10.392	10.771	14.881	90	90	90	2016	[236]
EKANIN	$P2_1$	8.0652	8.7724	16.248	90	99.004	90	2003	[237]
ELOWUZ	$P2_12_12_1$	9.011	11.244	18.96	90	90	90	2015	[238]
ELUVUD	$P2_12_12_1$	8.3995	12.264	17.383	90	90	90	2010	[239]
EMEVAU	$P2_1$	9.104	7.481	13.577	90	104.216	90	2010	[240]
EMIZOP	$P1$	8.459	10.881	16.243	83.67	85.8	78.9	2003	[241]
EMOMUQ	$P6_122$	13.48	13.48	46.226	90	90	120	2016	[242]
ENAZUP	$P2_1$	8.947	5.515	16.088	90	98.93	90	2011	[243]
ENEFEI	$P2_12_12_1$	8.7632	9.695	27.381	90	90	90	2003	[244]
EQELOD	$P2_1$	6.0369	15.253	9.07	90	107.568	90	2016	[245]
EQOLAX	$P2_12_12_1$	7.979	9.742	14.053	90	90	90	2003	[246]
ERITII	$P2_12_12_1$	6.683	9.166	12.696	90	90	90	2004	[247]
ERIWOR	$P2_12_12_1$	8.427	10.2	11.163	90	90	90	2004	[248]
ESOKOO	$P2_12_12_1$	5.2177	13.903	21.121	90	90	90	2015	[249]
EWEHUK	$P2_12_12_1$	8.8027	9.9264	13.709	90	90	90	2011	[250]
EYABAH	$P2_12_12_1$	8.6141	10.721	13.421	90	90	90	2003	[251,252]
EYABEL	$P2_12_12_1$	10.483	11.541	12.533	90	90	90	2003	[251]
EYABIP	$P2_12_12_1$	10.468	10.549	13.582	90	90	90	2003	[251]
EYIFOI	$P2_1$	9.092	16.052	14.968	90	100.93	90	2011	[253]
EYIJUR	$P2_12_12_1$	4.8848	8.0205	19.558	90	90	90	2004	[254]
EZAMUO	$C2$	18.97	6.1032	12.078	90	95.162	90	2011	[255]
EZEPIJ	$P2_12_12_1$	6.2562	8.6921	17.392	90	90	90	2011	[256]
EZULOB	$P2_12_12_1$	8.3573	12.208	16.021	90	90	90	2010	[257]

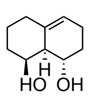
EABCEP
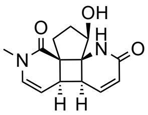
EACHOF
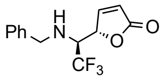
EBAPUT
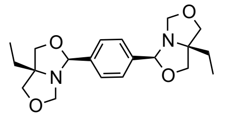
EBISEO
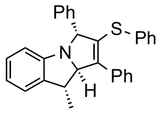
ECUPOK

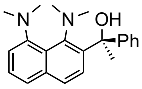
EDAHAT
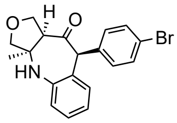
EDAQOS
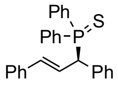
EFEMIM
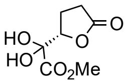
EGEZUN
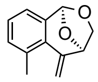
EGIZEA
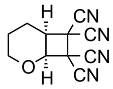
EGOTIG

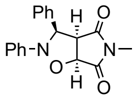
EGOVAZ
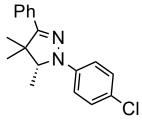
EHABOH
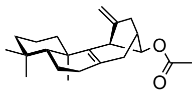
EHATEO
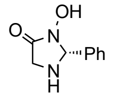
EJUHOG
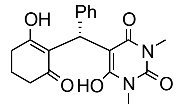
EJUQUX
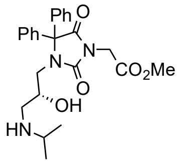
EKANIN

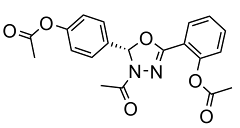
ELOWUZ
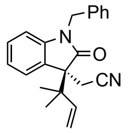
ELUVUD
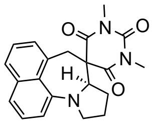
EMEVAU
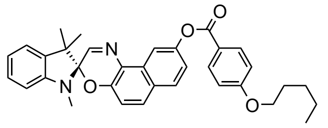
EMIZOP
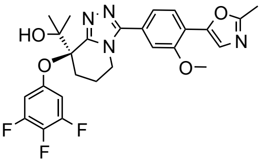
EMOMUQ

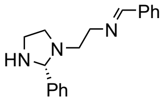
ENAZUP
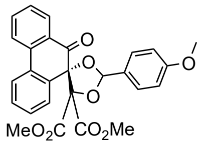
ENEFEI
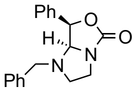
EQELOD
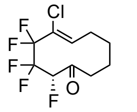
EQOLAX
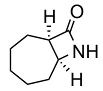
ERIVIM
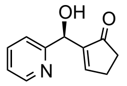
ERIWOR

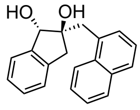
ESOKOO
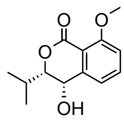
EWEHUK
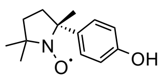
EYABAH
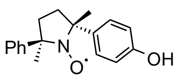
EYABEL
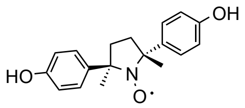
EYABIP

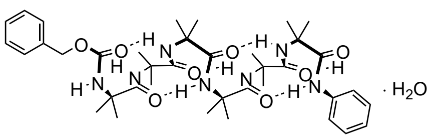
EYIFOI
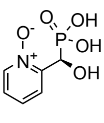
EYIJUR
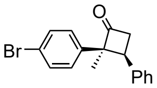
EZAMUO
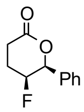
EZEPIJ
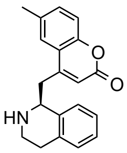
EZULOB

CSD Code	Space Group	a / Å	b / Å	c / Å	α / °	β / °	γ / °	Publication Year	Reference
FACXOY	$P2_12_12_1$	8.7615	9.7378	26.004	90	90	90	2010	258
FADWIT	$P2_12_12_1$	10.163	10.476	15.398	90	90	90	2012	259
FAFJAX	$P2_1$	10.507	6.383	15.587	90	101.52	90	1985	260
FALCEA	$P2_12_12_1$	26.207	13.828	9.259	90	90	90	1986	261
FALMUB	$P2_12_12_1$	8.5785	14.193	19.42	90	90	90	2004	262
FALPUD	$P2_12_12_1$	27.987	10.596	6.291	90	90	90	1986	263
FAMGUY	$P2_12_12_1$	8.7225	10.2	22.663	90	90	90	2017	264
FAMHAC	$P2_12_12_1$	11.654	13.301	7.193	90	90	90	1986	265
FARBIK	$P2_12_12_1$	7.5069	9.0899	13.765	90	90	90	2004	266
FASKUH	$P2_12_12_1$	8.317	13.668	21.026	90	90	90	2011	267
FAVBAH	$P2_12_12_1$	8.8893	14.433	15.441	90	90	90	2012	268
FAWNAV	$P2_12_12_1$	8.1071	9.3064	22.248	90	90	90	2017	269
FAWNEZ	$P2_1$	5.4384	7.1721	22.371	90	94.674	90	2017	269
FAXMIA	$P2_12_12_1$	10.584	10.621	14.778	90	90	90	1986	270
FAZYUD	$P2_12_12_1$	7.631	8.0161	16.235	90	90	90	2017	271
FEBJAY	$P2_12_12_1$	6.4273	15.664	18.992	90	90	90	2005	272
FECQAF	$P2_12_12$	14.919	17.888	5.238	90	90	90	1995	273
FEDQOV	$P2_12_12_1$	6.2771	9.792	19.073	90	90	90	2005	274
FEFMAF	$P2_12_12_1$	9.955	6.9722	14.673	90	90	90	2005	275
FEGZEW	$P2_12_12_1$	7.59	9.26	18.292	90	90	90	1987	276
FEHQEP	$P2_12_12_1$	5.6377	15.429	16.311	90	90	90	2004	277
FEPBAD	$P2_12_12_1$	6.942	12.421	13.114	90	90	90	1986	278
FEPTID	$P2_12_12_1$	9.574	22.453	6.661	90	90	90	1987	279
FEQBIP	$P2_1$	8.9283	5.4619	11.969	90	96.363	90	2017	280
FESCEM	$P2_12_12_1$	7.418	9.071	12.466	90	90	90	2005	281
FETMUM	$P2_12_12_1$	7.811	10.958	18.785	90	90	90	1987	282
FEWPUV	$P2_12_12_1$	8.8186	13.585	15.848	90	90	90	2018	283
FEXDET	$P2_12_12_1$	5.2087	10.676	31.392	90	90	90	2013	284
FEZWOZ	$P2_1$	8.241	5.45	10.32	90	92.32	90	2016	285
FICKEK	$P2_12_12_1$	10.378	11.467	13.112	90	90	90	2018	286
FIDPEQ	$P2_1$	11.118	6.2556	13.398	90	111.128	90	2018	287
FIFSOF	$P2_12_12_1$	8.4661	14.435	18.448	90	90	90	2018	288
FIKXOP	$P2_12_12_1$	11.57	13.01	16.385	90	90	90	2018	289
FINDOW	$P2_12_12_1$	10.113	10.206	29.277	90	90	90	2005	290
FIQZEN	$P2_1$	8.9888	10.158	9.5695	90	99.935	90	2018	291
FIVRUX	$P2_12_12_1$	7.068	10.857	20.343	90	90	90	1986	292
FIWNAC	$P2_1$	12.246	6.725	14.229	90	92.04	90	2014	293
FIYPIO	$P2_1$	9.1283	7.1019	10.576	90	113.669	90	2013	294
FIZHIF	$P2_12_12_1$	7.969	16.371	20.334	90	90	90	1987	295
FIZLIM	$P2_1$	9.6919	7.449	17.983	90	91.664	90	2018	296
FIZPUA	$P2_12_12_1$	9.0758	9.4879	11.738	90	90	90	2005	297
FIZQIQ	$P2_12_12_1$	4.2026	9.1884	28.27	90	90	90	2014	298
FIZQOX	$P2_1$	8.201	8.359	15.89	90	102.628	90	2019	299
FIZTEQ	$P2_12_12_1$	8.3242	12.107	20.597	90	90	90	2018	300
FOKKEY	$P2_12_12_1$	10.707	11.468	13.31	90	90	90	2019	301
FOLQOO	$P2_12_12_1$	5.4272	8.1299	29.841	90	90	90	2014	302
FOLQUT	$P2_12_12_1$	8.656	16.866	7.8378	90	90	90	2005	303
FOLQUU	$P2_12_12_1$	5.3299	7.8346	26.19	90	90	90	2014	302
FONWIO01	$P2_12_12_1$	18.013	8.299	5.577	90	90	90	1987	304
FOTNOT	$P2_1$	6.0935	8.9206	14.327	90	100.014	90	2014	305
FOVSAK	$P2_12_12_1$	5.873	16.483	17.546	90	90	90	1987	306
FOVSEO	$P2_1$	11.305	7.722	13.566	90	113.3	90	1987	307

CSD Code	Space Group	a / Å	b / Å	c / Å	α / °	β / °	γ / °	Publication Year	Reference
FOXCEB	$P2_1$	7.5622	10.637	11.15	90	100.373	90	2009	[308]
FOYDED	$P4_3$	10.492	10.492	12.925	90	90	90	2008	[309]
FOZFEF	$P2_1$	7.378	9.899	10.456	90	108	90	1986	[310]
FOZHIN	$P2_1$	5.662	16.271	10.324	90	97.696	90	2015	[311]
FUCJUI	$P2_12_12_1$	8.331	12.142	13.967	90	90	90	1987	[312]
FUFLAT	$P2_12_12_1$	12.326	19.182	7.227	90	90	90	1987	[313]
FUGFOC	$P2_12_12_1$	7.076	8.348	22.907	90	90	90	1987	[314]
FUHMOL	$P2_12_12_1$	9.474	15.057	15.182	90	90	90	2009	[315]
FUHNAY	$P2_1$	8.832	5.2321	16.588	90	102.005	90	2009	[316]
FUHZEN	$P2_12_12_1$	10.783	6.868	12.183	90	90	90	1987	[317]
FUJPOP	$P2_12_12_1$	8.644	9.749	11.461	90	90	90	1987	[318]
FULZIV	$P2_12_12_1$	8.982	10.08	10.739	90	90	90	1987	[319]
FUMMEG	$P2_1$	8.9411	11.072	10.937	90	99.81	90	2010	[320]
FUMMOQ	$P2_12_12_1$	9.5924	12.991	15.022	90	90	90	2010	[320]
FUVKIQ	$P2_12_12_1$	11.381	19.11	9.982	90	90	90	1987	[321]
FUWLOZ	$P2_12_12_1$	7.1099	10.297	13.07	90	90	90	2010	[322]
FUXZEF	$P4_3$	14.911	14.911	9.1435	90	90	90	2015	[323]
FUYLES	$P2_12_12_1$	7.885	14.769	16.212	90	90	90	2015	[324]
FUYMET	$P2_12_12_1$	11.688	14.889	16.832	90	90	90	2015	[325]
FUZKUH	$P3_2$	9.6842	9.6842	16.744	90	90	120	2010	[326]

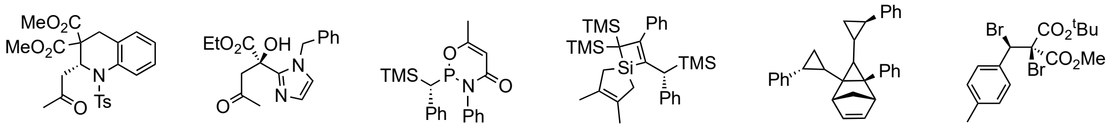

FACXOY FADWIT FAFJAX FALCEA FALMUB FALPUD

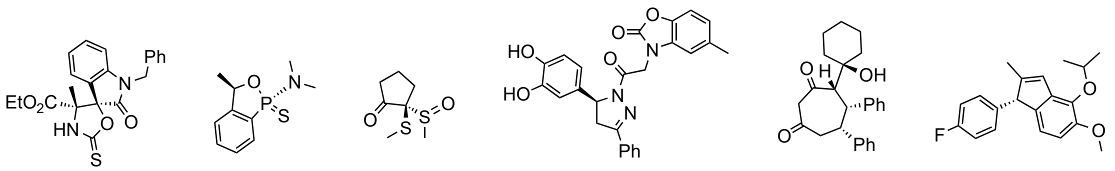

FAMGUY FAMHAC FARBIK FASKUH FAVBAH FAWNAV

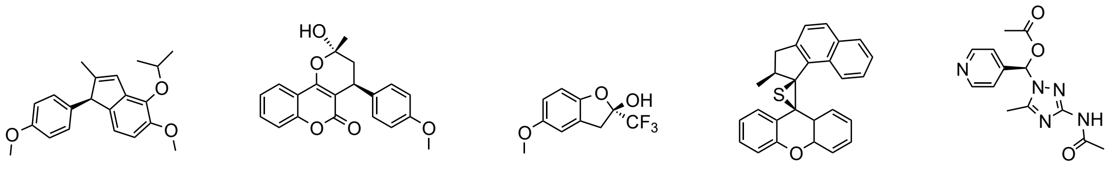

FAWNEZ FAXMIA FAZYUD FEBJAY FECQAF

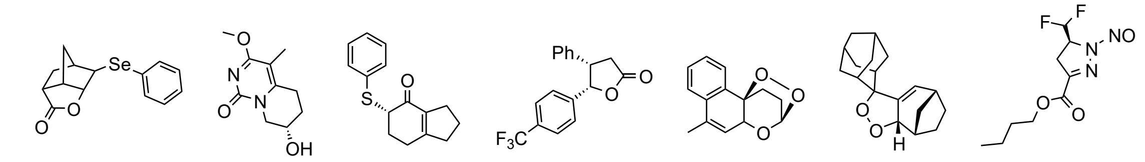

FEDQOV FEFMAF FEGZEW FEHQEP FEPBAD FEPTID FEQBIP

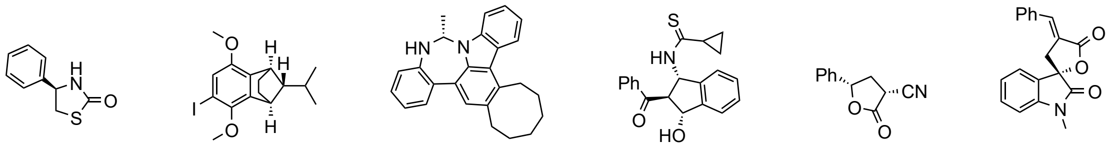

FESCEM FETMUM FEWPUV FEXDET FEZWOZ FICKEK

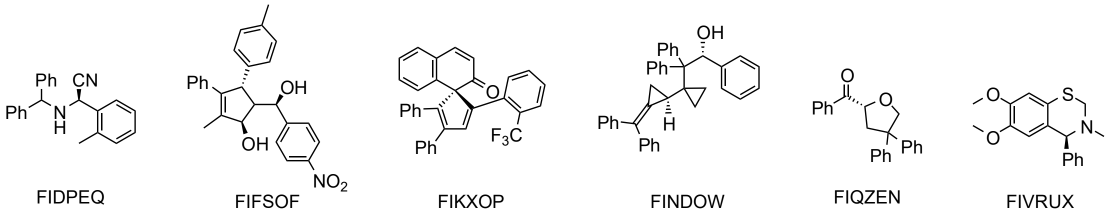

FIDPEQ FIFSOF FIKXOP FINDOW FIQZEN FIVRUX

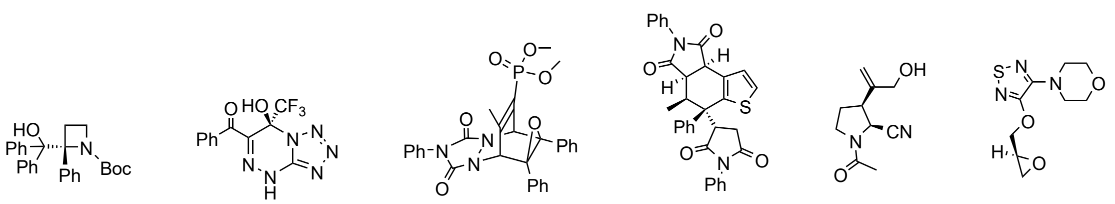

FIWNAC FIYPIO FIZHIF FIZLIM FIZPUA FIZQIQ

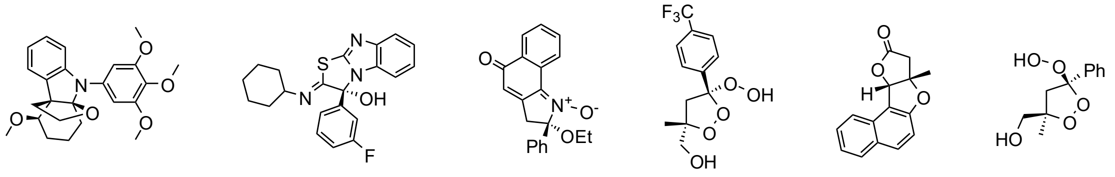

FIZQOX FIZTEQ FOKKEY FOLQOO FOLQUT FOLQUU

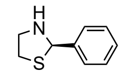

FONWIO01

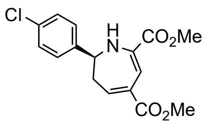

FOTNOT

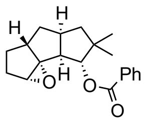

FOVSAK

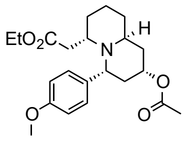

FOVSEO

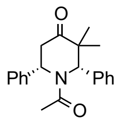

FOXCEB

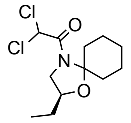

FOYDED

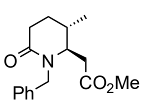

FOZFEF

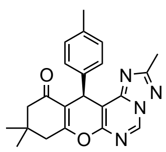

FOZHIN

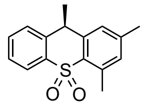

FUCJUI

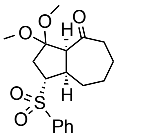

FUFLAT

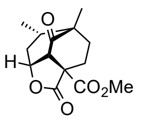

FUGFOC

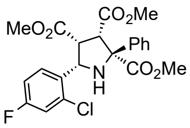

FUHMOL

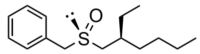

FUHNAY

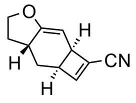

FUHZEN

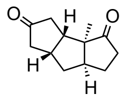

FUJPOP

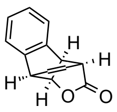

FULZIV

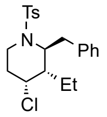

FUMMEG

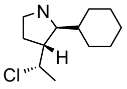

FUMMOQ

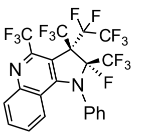

FUVKIQ

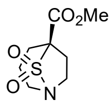

FUWLOZ

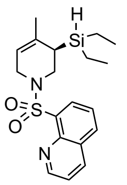

FUXZEF

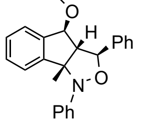

FUYLES

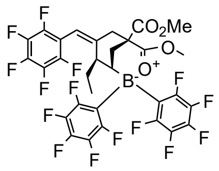

FUYMET

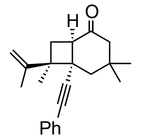

FUZKUH

CSD Code	Space Group	a / Å	b / Å	c / Å	α / °	β / °	γ / °	Publication Year	Reference
GABDAQ	$P2_12_12_1$	9.6625	12.859	19.525	90	90	90	2010	[327]
GABFOF	$P2_12_12_1$	8.694	12.995	15.514	90	90	90	2003	[328]
GACGIC	$P2_12_12_1$	6.3179	9.4245	16.735	90	90	90	2010	[329]
GACHAU	$P2_1$	8.6281	14.181	12.91	90	108.939	90	2003	[330]
GADPEG	$P2_12_12_1$	6.363	8.181	9.56	90	90	90	1988	[331]
GADTIR	$P2_12_12_1$	6.0705	13.727	19.378	90	90	90	2015	[332]
GADVAJ	$P2_12_12_1$	7.829	13.874	14.079	90	90	90	2003	[333]
GAFJIG	$P2_12_12_1$	14.967	21.816	8.075	90	90	90	1987	[334]
GAFMOR	$P2_1$	8.7652	7.9751	11.816	90	93.663	90	2010	[335]
GAFYIY	$P2_12_12_1$	7.974	14.147	16.341	90	90	90	2015	[336]
GAHGOM	$P2_12_12_1$	6.2622	13.68	15.513	90	90	90	2004	[337]
GASRUO	$P2_12_12_1$	9.5419	10.831	13.295	90	90	90	2005	[338]
GASZEH	$P2_12_12_1$	9.294	15.894	16.704	90	90	90	2011	[339]
GAWGOD	$P2_1$	8.0521	10.418	10.027	90	96.73	90	2017	[340]
GAZNEC	$P2_12_12_1$	6.9909	8.0891	33.009	90	90	90	2012	[341]
GAZNOM	$P2_12_12_1$	8.1311	11.36	22.161	90	90	90	2012	[341]
GEDWIV	$P2_12_12_1$	6.221	12.571	17.303	90	90	90	1988	[342]
GEFHOQ	$P2_1$	5.919	16.642	9.017	90	93.04	90	2012	[343]
GEHVUM	$P2_12_12_1$	10.932	12.065	12.169	90	90	90	2012	[344]
GEPTAY	$P2_12_12_1$	10.856	11.111	17.56	90	90	90	2012	[345]
GEQXIM	$P2_12_12_1$	7.8713	12.687	21.042	90	90	90	2017	[346]
GESYOT	$P2_12_12_1$	8.257	14.628	21.499	90	90	90	2006	[347]
GEVBEQ	$P2_12_12_1$	5.9368	15.404	18.531	90	90	90	2012	[348]
GEVGOF	$P2_12_12_1$	8.77	10.742	15.212	90	90	90	2012	[348]
GEYMEE	$P2_12_12_1$	9.767	10.497	15.894	90	90	90	2013	[349]
GEZWOA	$P2_12_12_1$	5.8015	7.985	22.001	90	90	90	2018	[350]
GIBVIX	$P2_12_12_1$	8.7252	11.502	13.944	90	90	90	2007	[351]
GIDHAD	$P2_12_12_1$	9.437	13.583	17.778	90	90	90	2007	[352]
GIDHIL	$P2_12_12_1$	9.6914	15.319	15.42	90	90	90	2007	[352]
GIGYEA	$P2_1$	9.06	5.859	11.91	90	102.15	90	1987	[353]
GIMVED	$P2_1$	6.798	8.733	12.486	90	91.63	90	1988	[354]
GIMXEH	$P2_12_12_1$	7.5436	14.541	19.762	90	90	90	2013	[355]
GINYUY	$P2_12_12_1$	8.6388	11.02	20.821	90	90	90	2007	[356]
GIQFUJ	$P2_12_12_1$	5.8983	16.394	20.127	90	90	90	2013	[357]
GIRBAN	$P2_12_12_1$	5.605	7.129	24.013	90	90	90	2018	[358]
GITWIS	$P2_1$	8.4173	8.6126	9.9289	90	104.531	90	2019	[359]
GIYQIP	$P2_12_12_1$	7.1009	8.107	45.591	90	90	90	2007	[360]
GOCZEG	$P2_1$	7.485	10.857	7.623	90	114.93	90	2018	[361]
GODLET01	$P2_12_12_1$	7.8363	16.438	16.69	90	90	90	2019	[362]
GODQAU	$P2_12_12_1$	5.0789	13.815	16.38	90	90	90	2019	[363]
GOGNAS	$P2_12_12_1$	9.7437	9.9574	17.451	90	90	90	2008	[364]
GOJRIJ	$P2_12_12_1$	7.6297	9.5304	25.273	90	90	90	2019	[365]
GOKKEX	$P2_1$	8.5101	10.53	9.8836	90	114.034	90	2007	[366]
GOLXIR	$P2_12_12_1$	6.5738	9.2172	35.512	90	90	90	2019	[367]
GOMGUK	$P2_12_12_1$	9.5497	11.21	11.546	90	90	90	1999	[368]
GOMHAR	$P2_12_12_1$	8.0072	11.393	13.622	90	90	90	1999	[368]
GONDIW	$P2_12_12_1$	9.5464	10.693	16.945	90	90	90	1998	[369]
GOPBOE	$P2_12_12_1$	10.359	13.026	18.498	90	90	90	2014	[370]
GORWEP	$P2_12_12_1$	11.403	17.345	19.568	90	90	90	1999	[371]
GOVKAF	$P2_12_12_1$	6.8744	8.6699	15.729	90	90	90	2016	[372]
GUBGEP	$P2_12_12$	11.069	25.113	7.8901	90	90	90	1999	[373]
GUCJUK	$C2$	28.32	4.7341	6.94	90	101.082	90	2009	[374]

CSD Code	Space Group	a / Å	b / Å	c / Å	α / °	β / °	γ / °	Publication Year	Reference
GUDSEF	$P2_12_12_1$	5.9724	14.897	16.486	90	90	90	2015	[375]
GUHKEB	$P2_12_12_1$	7.3039	13.118	19.337	90	90	90	2014	[376]
GUKCOE	$P2_12_12_1$	10.309	12.446	16.055	90	90	90	2000	[377]
GULHIF	$P4_3$	15.966	15.966	11.127	90	90	90	2009	[378]
GUMBAR	$R3$	23.056	23.056	7.325	90	90	120	2000	[379]
GUMBEV	$R3$	22.864	22.864	7.229	90	90	120	2000	[379]
GUMRUC	$P2_12_12_1$	6.6916	13.121	24.345	90	90	90	2009	[380]
GUMTEO	$P2_12_12_1$	7.6507	10.325	28.522	90	90	90	2009	[381]
GUNHAA	$P2_12_12_1$	7.2823	8.0024	24.632	90	90	90	2008	[382]
GUPCOL	$P2_1$	10.228	18.585	11.729	90	106.401	90	2005	[383]
GUPDAY	$P2_12_12_1$	11.985	18.924	19.595	90	90	90	2005	[383]
GURPEQ	$P2_12_12_1$	12.429	22.99	23.405	90	90	90	2014	[384]
GURROA	$P3_221$	6.0988	6.0988	31.485	90	90	120	2002	[385]
GUZMIY	$P2_12_12_1$	8.3863	13.161	14.465	90	90	90	2010	[386]

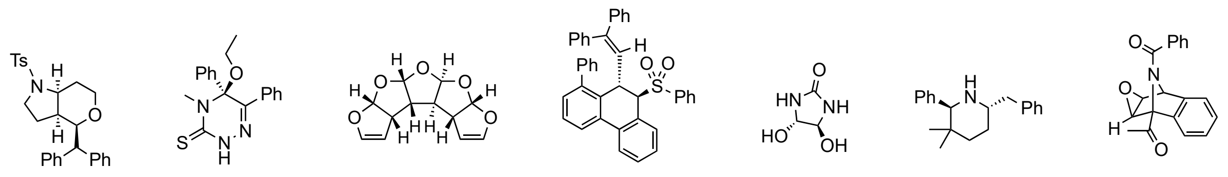

GABDAQ GABFOF GACGIC GACHAU GADPEG GADTIR GADVAJ

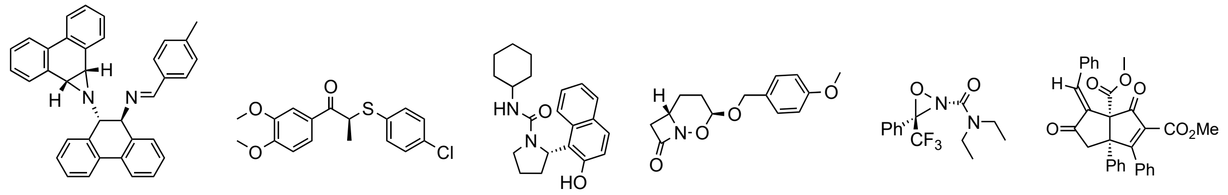

GAFJIG GAFMOR GAFYIY GAHGOM GASRUO GASZEH

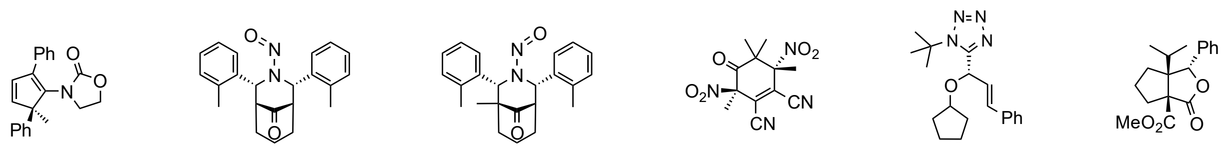

GAWGOD GAZNEC GAZNOM GEDWIV GEFHOQ GEHVUM

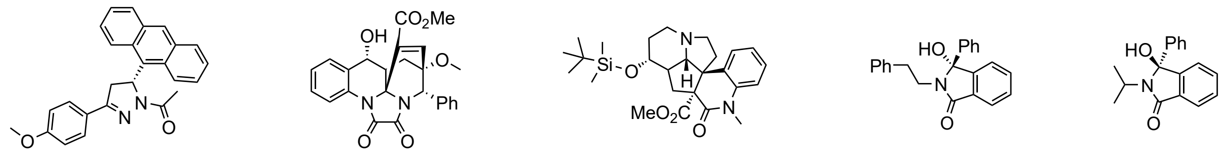

GEPTAY GEQXIM GESYOT GEVBEQ GEVGOF

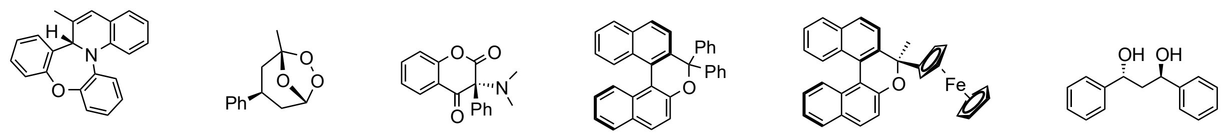

GEYMEE GEZWOA GIBVIX GIDHAD GIDHIL GIGYEA

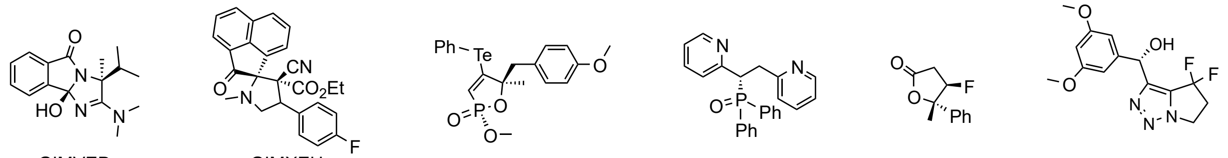

GIMVED GIMXEH GINYUY GIQFUJ GIRBAN GITWIS

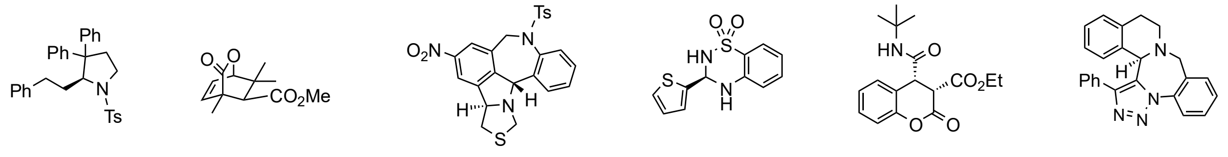

GIYQIP GOCZEG GODLET01 GODQAU GOGNAS GOJRIJ

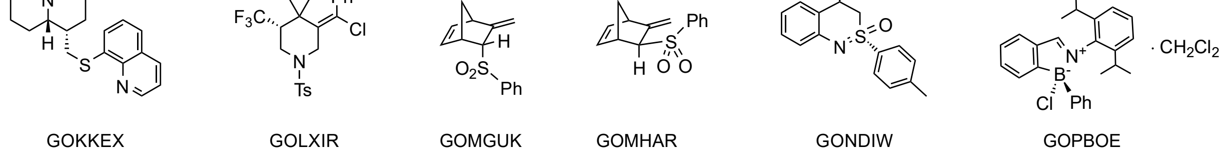

GOKKEX GOLXIR GOMGUK GOMHAR GONDIW GOPBOE

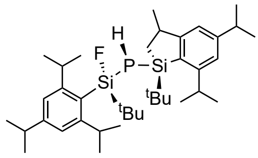

GORWEP

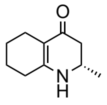

GOVKAF

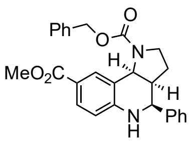

GUBGEP

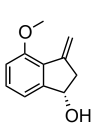

GUCJUK

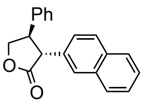

GUDSEF

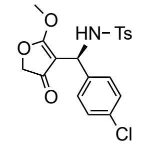

GUHKEB

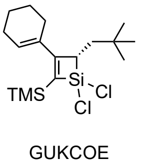

GUKCOE

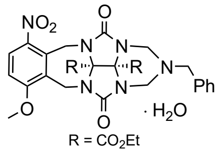

GULHIF

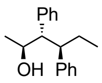

GUMBAR

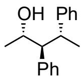

GUMBEV

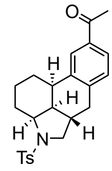

GUMRUC

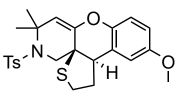

GUMTEO

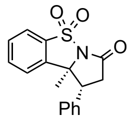

GUNHAA

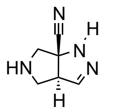

GURROA

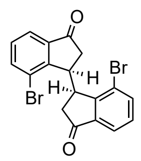

GUZMIY

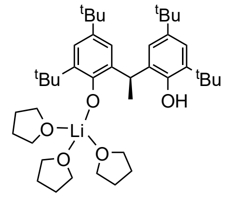

GUPCOL

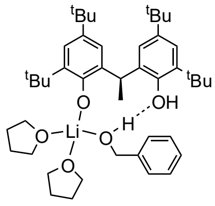

GUPDAY

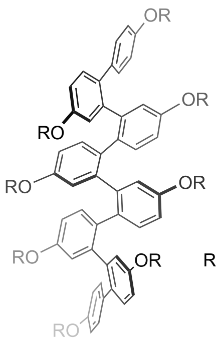

R = -SO_2-CF_3

GURPEQ

CSD Code	Space Group	a / Å	b / Å	c / Å	α / °	β / °	γ / °	Publication Year	Reference
HABXIT	$P2_12_12_1$	7.7379	12.248	17.757	90	90	90	2008	387
HAHMUB	$P2_12_12_1$	9.1302	11.005	13.371	90	90	90	2012	388
HAKLAI	$P2_12_12_1$	5.8098	10.849	19.378	90	90	90	2006	389
HALMAJ	$P2_12_12_1$	7.582	9.269	10.682	90	90	90	2004	390
HALPIT	$P2_12_12_1$	10.42	17.652	9.697	90	90	90	1993	391
HALYAW	$P2_1$	5.1041	7.7519	14.797	90	97.088	90	2011	392
HANBUV	$P2_12_12_1$	8.3735	12.156	15.483	90	90	90	2011	393
HANGIO	$P2_12_12_1$	8.1037	10.127	12.519	90	90	90	2011	394
HAQXOO	$P2_1$	8.585	7.368	14.067	90	95.35	90	2009	395
HARFEN	$P2_12_12_1$	6.048	7.315	24.334	90	90	90	2012	396
HARTIG	$P2_1$	7.944	18.203	8.111	90	109.195	90	2017	397
HASLEU	$P2_12_12_1$	5.216	10.846	26.57	90	90	90	2012	398
HASLIY	$P2_12_12_1$	6.5219	8.5138	17.335	90	90	90	2012	398
HASXAC	$P2_12_12_1$	8.6491	9.9416	24.689	90	90	90	2012	399
HAWPON	$P2_12_12_1$	9.6403	16.115	16.121	90	90	90	2017	400
HAXCAK	$P2_1$	9.791	7.129	10.428	90	91.84	90	1993	401
HAXWUY	$P2_12_12_1$	6.817	13.252	14.118	90	90	90	1993	402
HAZRUY	$P2_12_12_1$	5.631	7.857	41.046	90	90	90	2017	403
HEGHAC	$P2_12_12_1$	10.458	18.887	10.079	90	90	90	1994	404
HEJLEP	$P2_12_12_1$	5.523	10.232	22.007	90	90	90	2012	405
HEKBAA	$P2_12_12_1$	8.523	11.122	13.689	90	90	90	1994	406
HELTAT	$P2_1$	9.555	10.88	14.887	90	100.79	90	1994	407
HEMWIF	$P2_12_12_1$	13.073	13.568	9.397	90	90	90	1994	408
HEPQIF	$P2_12_12_1$	8.1382	11.224	19.464	90	90	90	2018	409
HERCUF	$P2_1$	10.166	6.943	19.088	90	101.036	90	2018	410
HERKIZ	$P2_12_12_1$	6.323	9.076	26.251	90	90	90	2006	411
HESVUW	$P2_1$	10.041	6.207	11.892	90	103.47	90	1994	412
HEWBAM	$P2_12_12_1$	11.277	22.094	9.119	90	90	90	1994	413
HEXEST	$P2_12_12_1$	6.521	7.724	34.089	90	90	90	1981	414
HEXGIA	$P2_12_12_1$	9.096	9.488	20.746	90	90	90	1998	415
HEXHOK	$P2_12_12_1$	5.7386	9.5416	18.314	90	90	90	2018	416
HEXQAD01	$P2_12_12_1$	4.6812	9.2627	23.407	90	90	90	2008	417
HIBZAT	$P2_12_12_1$	9.958	9.961	12.978	90	90	90	1995	418
HIDFOS	$P2_12_12_1$	7.4539	7.8553	17.229	90	90	90	2018	419
HIDMUC	$P2_1$	8.129	11.858	8.6895	90	112.431	90	1995	420
HIDNEN	$P2_12_12_1$	7.617	18.76	6.655	90	90	90	1995	420
HIGHIP	$P2_12_12_1$	9.4523	11.667	16.014	90	90	90	2007	421
HIJREA	$P2_12_12_1$	6.2744	14.563	15.649	90	90	90	2019	422
HIPTUW	$P2_1$	9.463	12.981	11	90	91.119	90	2007	423
HISFIZ	$P2_12_12$	13.702	23.168	8.5695	90	90	90	2007	424
HITPIJ	$P2_12_12_1$	7.249	16.064	25.347	90	90	90	1998	425
HIWPIP	$P2_12_12_1$	5.5306	7.8574	24.719	90	90	90	2019	426
HIYFIH	$P2_12_12_1$	6.3372	9.2748	30.538	90	90	90	2019	427
HMDNOA	$P2_12_12_1$	13.111	11.738	8.11	90	90	90	1980	428
HMDNOB	$P2_12_12_1$	13.125	11.571	8.206	90	90	90	1980	428
HMPYQU	$P2_12_12_1$	9.766	10.77	12.71	90	90	90	1980	429
HOBYUT	$P2_12_12_1$	8.547	11.428	18.288	90	90	90	2008	430
HOCDAF	$P2_12_12_1$	10.07	16.489	18.229	90	90	90	2008	431
HOCKUH	$P2_12_12_1$	9.4416	9.8267	28.428	90	90	90	2014	432
HOCNET	$P2_12_12_1$	6.8975	8.1786	19.802	90	90	90	2008	433
HODZIM	$P2_12_12_1$	8.0077	9.6919	12.115	90	90	90	2019	434
HOFXIK	$P2_1$	5.4172	8.0164	16.231	90	96.745	90	2008	435

CSD Code	Space Group	a / Å	b / Å	c / Å	α / °	β / °	γ / °	Publication Year	Reference
HOFXUW	$P2_12_12_1$	7.4328	12.375	14.918	90	90	90	2008	[436]
HOFYAD	$P2_12_12_1$	9.6174	11.558	13.465	90	90	90	2008	[436]
HOHHES	$P2_12_12_1$	7.743	9.106	22.312	90	90	90	2008	[437]
HOHPIE	$P2_12_12_1$	8.329	13.107	14.814	90	90	90	2008	[438]
HOHXUX	$P2_1$	6.1632	16.972	8.6866	90	96.681	90	1999	[439]
HOJSOQ	$C2$	20.83	5.635	12.122	90	108.75	90	2014	[440]
HOMCIX	$P6_5$	11.265	11.265	21.23	90	90	120	2014	[441]
HOMKIF	$P2_12_12_1$	9.4591	13.114	13.833	90	90	90	2014	[442]
HONTIN	$P2_12_12_1$	6.1993	20.503	21.694	90	90	90	1998	[443]
HONXIR	$P2_12_12_1$	7.784	8.311	22.615	90	90	90	1999	[444]
HONZOC	$P2_1$	12.859	8.8437	13.157	90	118.365	90	2019	[445]
HOPHOL	$P2_12_12_1$	5	14	20	90	90	90	2013	[446]
HOPJIH	$P2_12_12_1$	7.569	12.618	16.578	90	90	90	2013	[447]
HOPNIK	$P2_1$	10.749	5.085	15.865	90	103.529	90	2009	[448]
HOQSIQ	$P2_12_12_1$	8.4336	10.453	21.561	90	90	90	2008	[449]
HOVYAT	$P2_12_12_1$	9.6931	9.8584	11.925	90	90	90	2009	[450]
HPRIND	$P2_12_12_1$	9.141	8.67	18.538	90	90	90	1980	[451]
HUDDAL	$P2_12_12_1$	8.3897	11.268	11.463	90	90	90	2001	[452]
HUHKOM	$P2_12_12_1$	5.837	14.149	17.65	90	90	90	2015	[453]
HUNDEZ	$P2_12_12_1$	10.43	13.453	16.702	90	90	90	2002	[454]
HURYUQ	$P1$	5.8748	6.6516	7.3831	67.621	84.526	66.767	2015	[455]
HUTNIU	$P2_1$	6.3808	17.138	7.7629	90	93.772	90	2010	[456]
HUVVEA	$P2_12_12_1$	5.5355	8.2984	31.406	90	90	90	2010	[457]
HUWLEQ	$P2_12_12_1$	8.2895	19.855	6.0825	90	90	90	2003	[458]
HUXJEQ	$P2_12_12_1$	8.8552	9.5107	20.672	90	90	90	2010	[459]
HUZWAC	$P1$	5.9629	9.6761	9.6943	83.833	82.215	89.828	2015	[460]

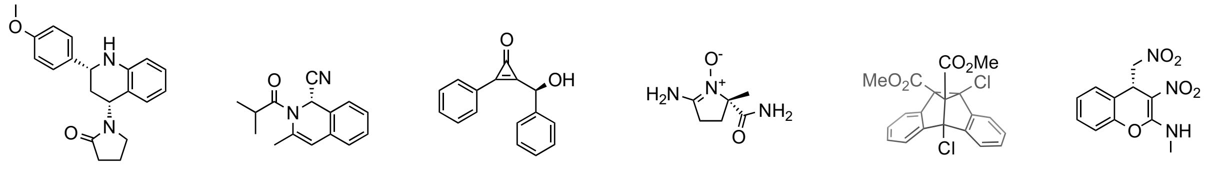

HABXIT HAHMUB HAKLAI HALMAJ HALPIT HALYAW

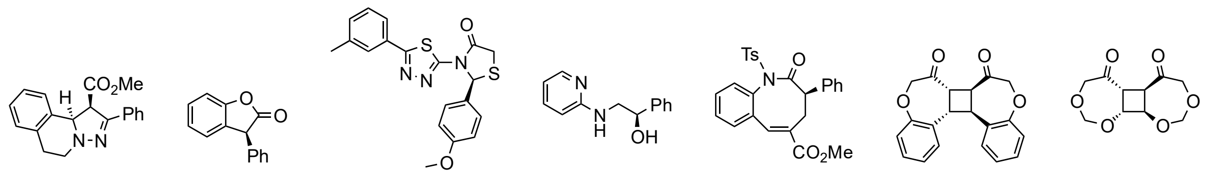

HANBUV HANGIO HAQXOO HARFEN HARTIG HASLEU HASLIY

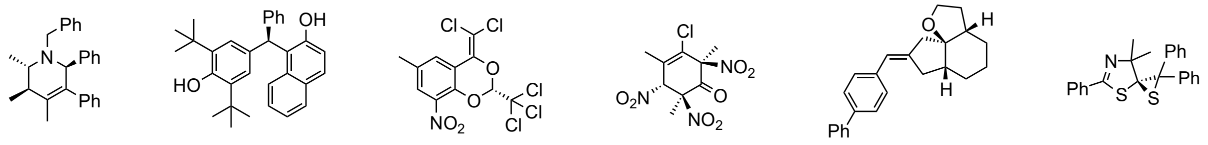

HASXAC HAWPON HAXCAK HAXWUY HAZRUY HAZRUY

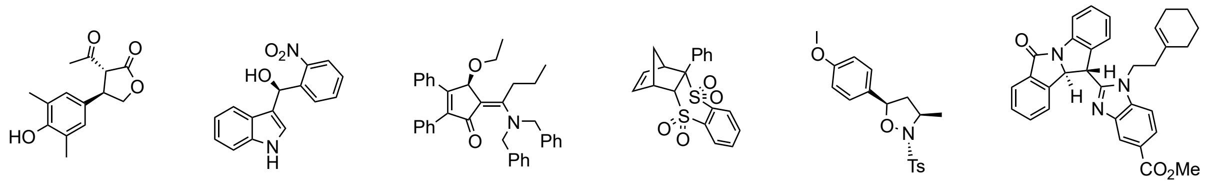

HEJLEP HEKBAA HELTAT HEMWIF HEPQIF HERCUF

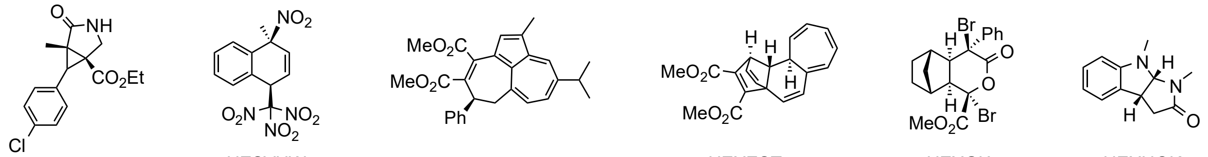

HERKIZ HESVUW HEWBAM HEXEST HEXGIA HEXHOK

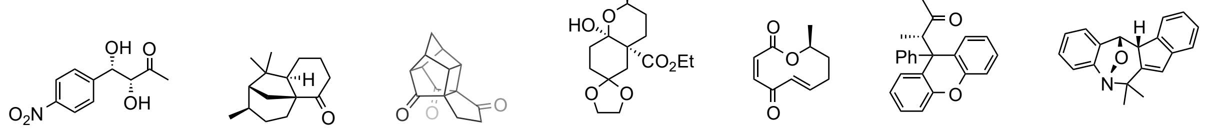

HEXQAD01 HIBZAT HIDFOS HIDMUC HIDNEN HIGHIP HIJREA

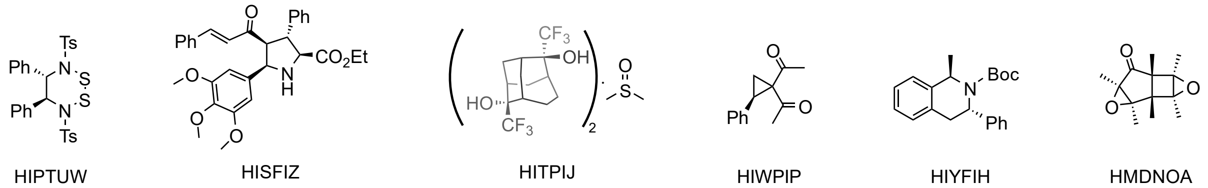

HIPTUW HISFIZ HITPIJ HIWPIP HIYFIH HMDNOA

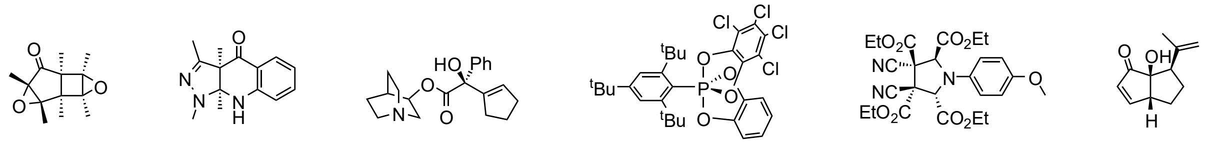

HMDNOB HMPYQU HOBYUT HOCDAF HOCKUH HOCNET

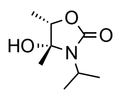
HODZIM

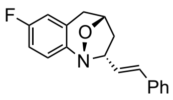
HOFXIK

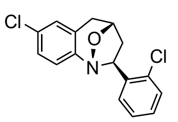
HOFXUW

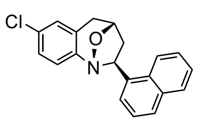
HOFXUW

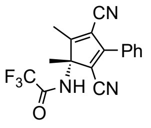
HOHHES

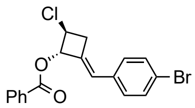
HOHPIE

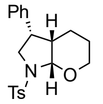
HOHXUX

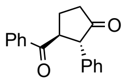
HOJSOQ

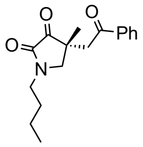
HOMCIX

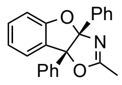
HOMKIF

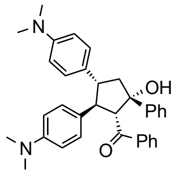
HONTIN

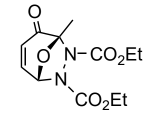
HONXIR

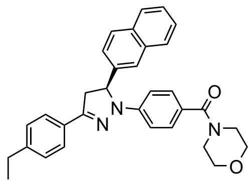
HONZOC

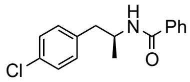
HOPHOL

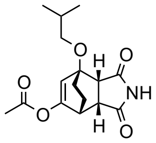
HOPJIH

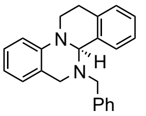
HOPNIK

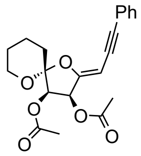
HOQSIQ

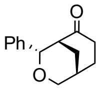
HOVYAT

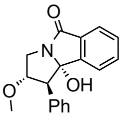
HPRIND

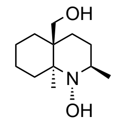
HUDDAL

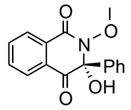
HUHKOM

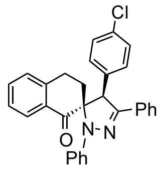
HUNDEZ

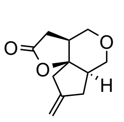
HURYUQ

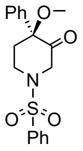
HUTNIU

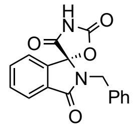
HUVVEA

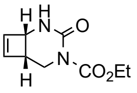
HUWLEQ

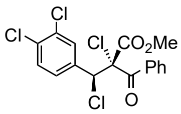
HUXJEQ

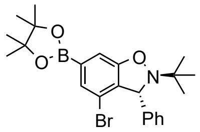
HUZWAC

CSD Code	Space Group	a / Å	b / Å	c / Å	α / °	β / °	γ / °	Publication Year	Reference
IBAJON	$P2_1$	8.8392	12.746	9.5979	90	99.664	90	2016	461
IBIYOK	$P2_12_12_1$	4.6736	17.606	19.17	90	90	90	2017	462
IBORAT	$P2_12_12_1$	7.3492	11	18.093	90	90	90	2007	463
ICEBIB	$P2_12_12_1$	9.1047	10.75	26.495	90	90	90	2000	464
ICOGUC	$P2_12_12_1$	6.116	12.66	16.38	90	90	90	2001	465
ICUSEF	$P2_12_12_1$	9.5729	10.351	15.169	90	90	90	2006	466
ICUSOP	$P2_12_12_1$	6.7139	7.4777	29.86	90	90	90	2006	466
IDABUL	$P2_12_12_1$	14.858	14.905	11.273	90	90	90	2006	467
IDAZES	$P2_12_12_1$	7.856	14.382	18.746	90	90	90	2001	468
IDELUB	$P2_1$	9.114	8.76	11.937	90	98.233	90	2018	469
IDOQUP	$P2_12_12_1$	8.4421	10.55	20.68	90	90	90	2013	470
IDUHIY	$P2_1$	7.853	16.234	10.534	90	93.12	90	2002	471
IFEJAE	$P2_12_12_1$	6.499	13.533	15.91	90	90	90	2002	472
IFIDOQ	$P2_12_12_1$	7.962	8.249	18.931	90	90	90	2002	473
IFUWAK	$P2_12_12_1$	5.0522	8.4389	21.723	90	90	90	2018	474
IHURIO	$P2_1$	8.698	5.5239	12.259	90	107.7	90	2015	475
IJUCOG	$P2_12_12_1$	12.686	13.673	15.278	90	90	90	2011	476
IKONED	$P2_12_12_1$	8.2974	13.684	15.775	90	90	90	2016	477
IMICAI	$P2_12_12_1$	9.5947	9.6887	11.319	90	90	90	2003	478
IMIPAX	$P2_12_12_1$	5.6672	14.727	22.855	90	90	90	2016	479
IMUQIR	$P2_12_12_1$	7.1904	11.345	24.359	90	90	90	2011	480
INENIY	$P2_1$	10.533	5.145	13.607	90	106.018	90	2003	481
INITUW	$P2_1$	11.722	6.0787	12.996	90	114.44	90	2016	482
INUKUY	$P2_12_12_1$	6.8896	14.746	30.691	90	90	90	2011	483,484
IPAMIX	$P2_12_12_1$	5.1576	11.222	21.303	90	90	90	2016	485
IPAPUL	$P2_12_12_1$	5.5698	16.002	16.311	90	90	90	2011	486
IPEXEG	$P2_12_12_1$	9.0053	12.194	21.174	90	90	90	2003	487
IPMUDS	$P2_12_12_1$	10.751	25.028	7.107	90	90	90	1973	488
IPUCUS	$P2_1$	7.2228	7.6519	10.57	90	95.512	90	2011	489
IPUDAZ	$P2_1$	5.8204	8.6505	11.438	90	100.784	90	2011	489
IPUHUY	$P2_12_12_1$	7.6054	11.951	17.019	90	90	90	2013	490
IPUMAJ	$P2_12_12_1$	8.698	9.5228	20.257	90	90	90	2009	491
IQACUZ	$P2_12_12_1$	7.1897	10.065	29.034	90	90	90	2010	492
IQADEK	$P2_12_12_1$	8.1254	13.476	21.652	90	90	90	2010	492
IQAFIQ	$P2_12_12_1$	4.7205	11.961	24.114	90	90	90	2010	493
IQOGUS	$P2_12_12_1$	8.2754	12.082	13.332	90	90	90	2016	494
IRANAR	$P2_12_12_1$	11.108	11.532	12.152	90	90	90	2011	495
IRUDOQ	$P2_12_12_1$	5.9339	12.305	21.713	90	90	90	2016	496
ISIPEH	$P2_12_12_1$	10.987	11.662	20.9	90	90	90	2016	497
ISOCUQ	$P2_12_12_1$	8.0525	10.875	16.216	90	90	90	2016	498
ISUMOA	$P2_1$	11.771	5.5329	12.706	90	115.831	90	2016	499
ISUMUG	$P2_1$	11.641	5.5396	12.781	90	115.94	90	2016	499
ISUPAN	$P2_12_12_1$	6.4796	17.968	16.059	90	90	90	2004	500
ISUXEA	$P2_12_12_1$	7.145	9.7128	11.446	90	90	90	2011	501
ITAKIX	$P1$	6.2686	8.196	6.084	111.81	101.01	110.62	2004	502–504
ITAWUW	$P2_1$	9.3853	11.87	12.071	90	95.56	90	2011	505
ITOHEG	$P2_12_12_1$	8.5662	10.764	14.342	90	90	90	2016	506
IVENAZ	$P2_12_12_1$	8.9435	10.815	19.188	90	90	90	2007	507
IVOCUR	$P2_12_12_1$	6.075	19.042	20.889	90	90	90	2002	508
IVOPOY	$C2$	17.365	6.852	12.991	90	99.854	90	2004	509
IWUQIC	$P2_12_12_1$	9.5802	13.211	19.031	90	90	90	2016	510
IWUZOR	$P2_12_12_1$	7.7399	11.983	15.86	90	90	90	2016	511
IZEBIY	$P2_12_12_1$	6.13	12.95	23.68	90	90	90	2004	512
IZIKUX	$P2_12_12_1$	8.264	11.829	12.102	90	90	90	2004	513
IZIQIT	$P2_12_12_1$	5.7857	10.276	22.093	90	90	90	2016	514
IZUYAD	$P2_12_12_1$	8.6317	14.882	17.572	90	90	90	2004	515,516

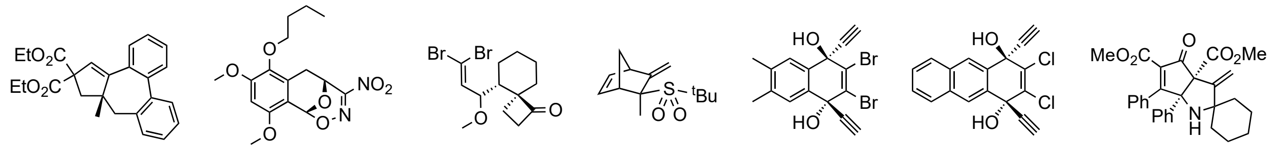

IBAJON IBIYOK IBORAT ICOGUC ICUSEF ICUSOP IDABUL

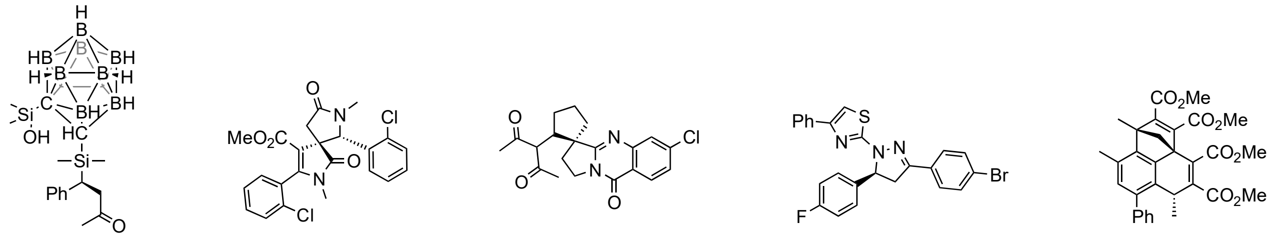

ICEBIB IDAZES IDELUB IDOMOF IDUHIY

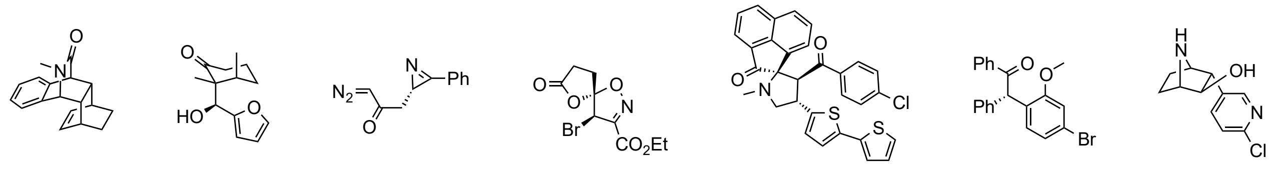

IFEJAE IFIDOQ IFUWAK IHURIO IJUCOG IKONED IMICAI

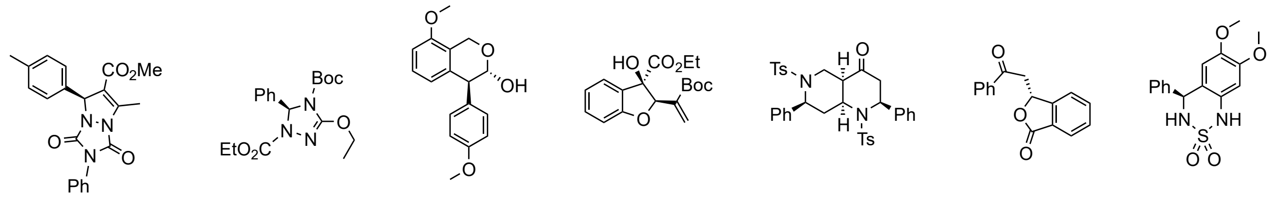

IMIPAX IMUQIR INENIY INITUW INUKUY IPAMIX IPAPUL

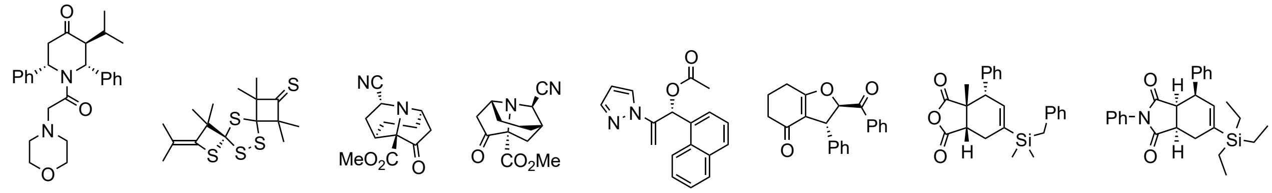

IPEXEG IPMUDS IPUCUS IPUDAZ IPUHUY IPUMAJ IQACUZ IQADEK

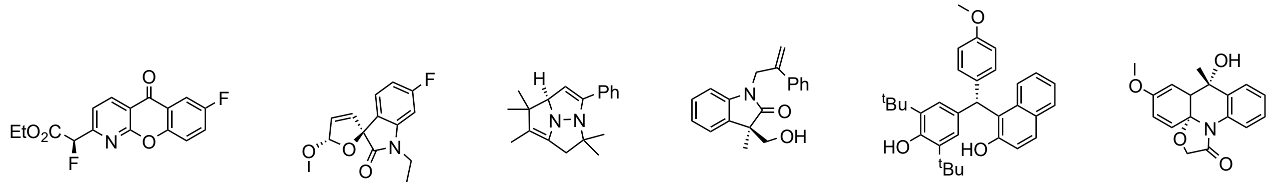

IQAFIQ IQOGUS IRANAR IRUDOQ ISIPEH ISOCUQ

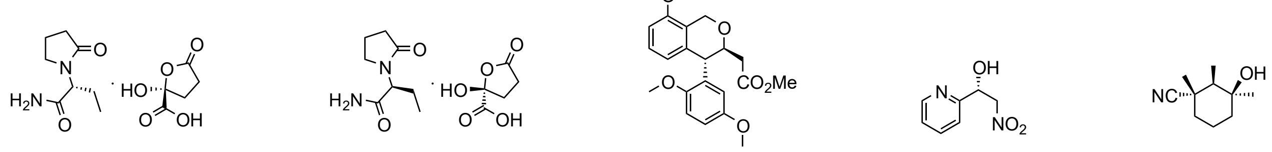

ISUMOA ISUMUG ISUPAN ISUXEA ITAKIX

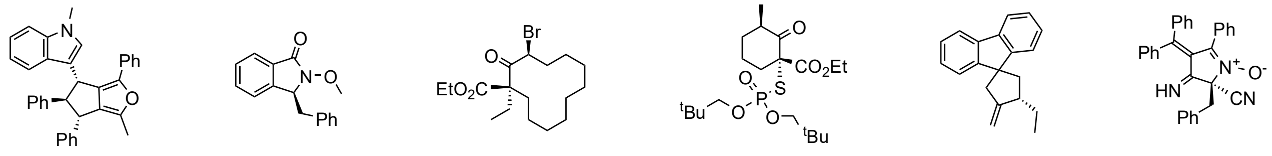

ITAWUW ITOHEG IVENAZ IVOCUR IVOPOY IWUQIC

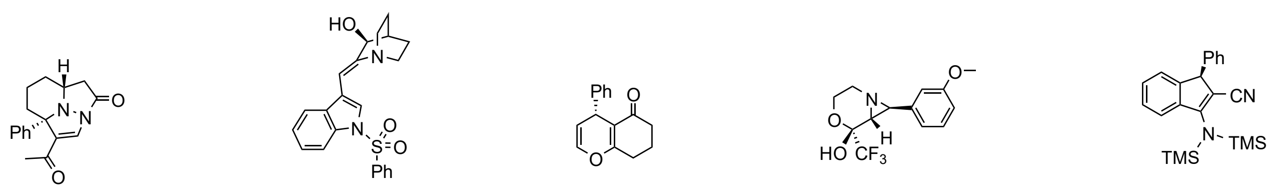

IWUZOR IZEBIY IZIKUX IZIQIT IZUYAD

CSD Code	Space Group	a / Å	b / Å	c / Å	α / °	β / °	γ / °	Publication Year	Reference
JABJEC	$P2_12_12_1$	5.047	6.583	35.061	90	90	90	2001	[517]
JABPUY	$P2_12_12_1$	5.6681	7.3025	21.099	90	90	90	2002	[518]
JABQIN	$P2_1$	10.037	4.493	12.149	90	107.257	90	2002	[518]
JACVIV	$P2_12_12_1$	11.198	13.537	13.635	90	90	90	2015	[519]
JADTAJ	$P2_12_12_1$	6.045	9.837	19.487	90	90	90	1989	[520]
JAFCIC	$P2_1$	12.844	8.396	5.351	90	94.3	90	1989	[521]
JAMGEJ	$P2_1$	10.59	5.84	11.17	90	109.8	90	1989	[522]
JAQGUE	$P6_5$	21.12	21.12	6.7753	90	90	120	2005	[523]
JAQNEX	$P2_1$	8.812	5.223	16.563	90	102.058	90	2009	[524]
JATLAR	$P2_12_12_1$	6.762	6.581	33.26	90	90	90	1989	[525]
JATVOP	$P2_12_12_1$	7.886	9.522	13.626	90	90	90	1989	[526]
JATXIL	$P2_12_12_1$	8.101	10.377	17.338	90	90	90	1989	[527]
JAVZUC	$P4_32_12$	11.704	11.704	28.975	90	90	90	2005	[528]
JAWKIC	$P2_12_12_1$	7.1237	11.333	17.328	90	90	90	2005	[529]
JEBRUD	$P2_1$	12.599	8.965	10.268	90	97.622	90	1989	[530]
JEFPEP	$P2_12_12_1$	8.18	11.348	13.996	90	90	90	1990	[531]
JEJSUN	$P2_12_12_1$	12.807	14.518	8.7113	90	90	90	2005	[532]
JESHOF	$P2_12_12_1$	4.2445	7.834	26.989	90	90	90	2006	[533]
JESHUK	$P2_12_12_1$	7.639	17.262	14.385	90	90	90	1990	[534]
JESPUS	$P2_1$	9.9985	5.9278	10.088	90	108.09	90	1990	[535]
JEXBIY	$P3_1$	8.701	8.701	11.671	90	90	120	2006	[536]
JIDGIM	$P2_12_12_1$	7.856	12.823	15.82	90	90	90	1990	[537]
JIJYOT	$P2_12_12_1$	8.09	8.792	24.87	90	90	90	2018	[538]
JILNIB	$P2_12_12_1$	7.769	10.191	29.594	90	90	90	1991	[539]
JILNOH	$P2_12_12_1$	7.696	10.441	30.13	90	90	90	1991	[539]
JIMHIX	$P2_12_12_1$	6.247	9.076	29.473	90	90	90	2007	[540]
JINBOX	$P2_12_12_1$	9.962	12.576	11.766	90	90	90	1991	[541]
JIPWOV	$P2_12_12_1$	8.1386	10.733	15.6	90	90	90	2007	[542]
JIQJOI	$P2_1$	7.602	9.84	10.411	90	103.07	90	1998	[543]
JIQKAV	$P2_12_12_1$	9.327	13.178	13.216	90	90	90	1998	[543]
JISVUC	$P2_12_12_1$	22.447	11.937	8.601	90	90	90	1991	[544]
JODQOI	$P2_12_12_1$	5.096	13.187	16.359	90	90	90	1996	[545]
JOGYEL	$P2_1$	11.084	9.0833	14.403	90	95.981	90	2014	[546]
JOHKIB	$P2_1$	6.377	12.363	6.4861	90	113.896	90	2008	[547]
JOHSAC	$P2_12_12$	12.309	23.558	7.8737	90	90	90	2014	[548]
JOKKUQ	$P3_2$	9.999	9.999	11.728	90	90	120	2008	[549]
JOPLEF	$P2_12_12_1$	9.99	14.406	18.106	90	90	90	1992	[550]
JORFEB	$P1$	6.442	8.083	9.231	63.436	82.195	83.721	1992	[551]
JOTKAE	$P2_12_12_1$	6.589	7.003	18.436	90	90	90	1992	[552]
JOXTEV	$P2_12_12_1$	11.625	16.666	9.78	90	90	90	1992	[553]
JUFDET	$P2_12_12_1$	8.546	11.391	13.348	90	90	90	1997	[554]
JUGTIQ	$P2_12_12_1$	5.7035	7.8753	45.629	90	90	90	2015	[555]
JUKJIK	$P2_1$	12.828	5.2901	14.448	90	97.477	90	2015	[556]
JUNRAN	$P2_12_12_1$	5.2106	12.434	33.325	90	90	90	2015	[557]
JUQLOW	$P2_1$	7.9421	14.695	8.5892	90	107.985	90	1999	[558]
JUWZUW	$P2_1$	6.222	7.469	15.502	90	99.02	90	1992	[559]

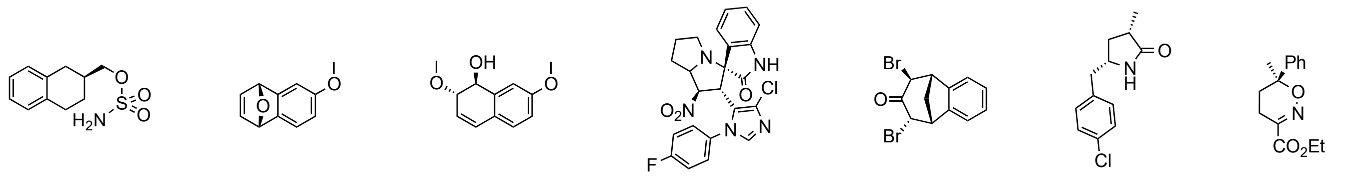

JABJEC JABPUY JABQIN JACVIV JADTAJ JAFCIC JAMGEJ

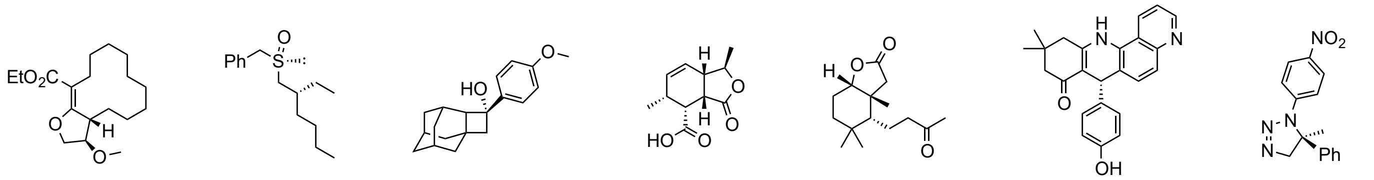

JAQGUE JAQNEX JATLAR JATVOP JATXIL JAVZUC JAWKIC

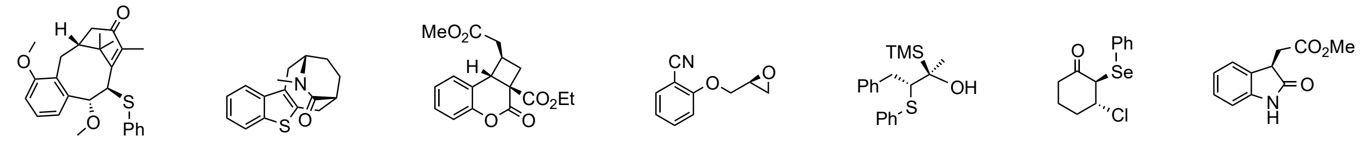

JEBRUD JEFPEP JEJSUN JESHOF JESHUK JESPUS JEXBIY

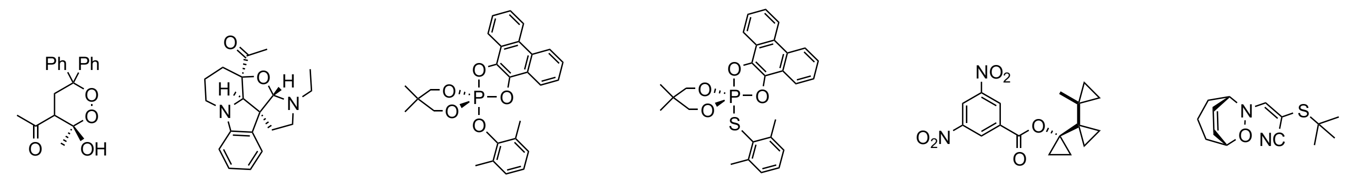

JIDGIM JIJYOT JILNIB JILNOH JIMHIX JINBOX

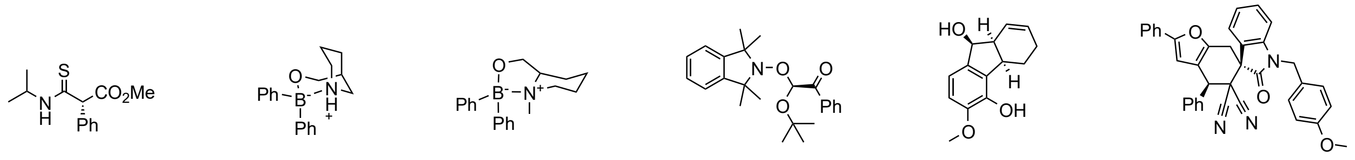

JIPWOV JIQJOI JIQKAV JISVUC JODQOI JOGYEL

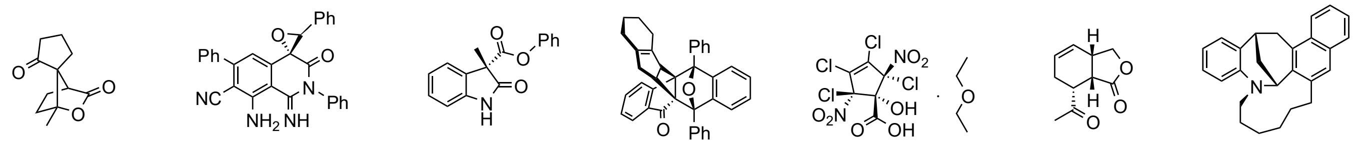

JOHKIB JOHSAC JOKKUQ JOPLEF JORFEB JOTKAE JOXTEV

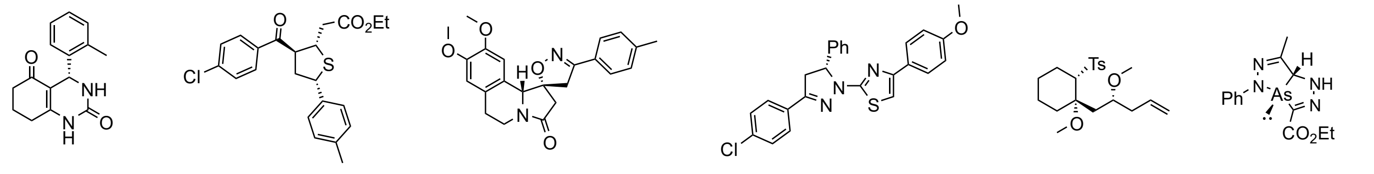

JUFDET JUGTIQ JUKJIK JUNRAN JUQLOW JUWZUW

CSD Code	Space Group	a / Å	b / Å	c / Å	α / °	β / °	γ / °	Publication Year	Reference
KABMUX	$P2_12_12_1$	7.7318	11.065	19.725	90	90	90	2010	560
KACLOP	$P2_12_12_1$	10.79	10.889	12.442	90	90	90	1989	561
KACLOQ	$P2_12_12_1$	8.677	11.408	21.372	90	90	90	2003	562
KAGTIV	$P2_12_12_1$	8.824	9.417	29.073	90	90	90	1989	563
KAHPAN	$P2_1$	8.912	5.8904	18.411	90	102.422	90	2016	564
KAJQET	$P2_12_12_1$	9.61	11.493	12.112	90	90	90	2010	565
KAKDUV	$P2_1$	7.443	16.387	7.813	90	110.33	90	1988	566
KALZIH	$P2_12_12_1$	6.5473	10.862	18.606	90	90	90	2005	567
KAMNUH	$P2_12_12_1$	9.708	12.14	12.437	90	90	90	1989	568
KAQFEP	$P2_12_12_1$	7.9672	12.027	21.151	90	90	90	2012	569
KAQMEV	$P2_12_12_1$	11.397	18.642	19.787	90	90	90	2005	570
KARGOZ	$P2_1$	8.267	10.699	9.039	90	113.67	90	1989	571
KATBAI	$P2_1$	8.831	7.21	13.207	90	105.77	90	1989	572
KATNIE	$P2_1$	11.235	7.9046	14.06	90	94.892	90	2011	573
KATRAA	$P2_1$	10.083	14.168	10.255	90	114.729	90	2012	574
KAWNUU	$P4_3$	16.181	16.181	12.989	90	90	90	2017	575
KAXSIM	$P2_12_12_1$	6.604	15.346	19.811	90	90	90	2005	576
KAYMOM	$P2_1$	10.278	6.467	11.972	90	95.46	90	1990	577
KAZHUO	$P2_1$	8.339	10.917	9.866	90	113.92	90	1989	578
KEBSES	$P2_1$	9.1353	14.149	11.24	90	108.677	90	2017	579
KEDWUL	$P2_1$	8.109	12.057	8.671	90	104.22	90	1989	580
KEFBOP	$P2_12_12_1$	7.7814	14.611	19.834	90	90	90	2017	581
KEFLIR	$P2_12_12_1$	9.8568	11.95	17.3	90	90	90	2002	582
KEGBIH	$P2_1$	8.213	10.841	8.234	90	95.57	90	1989	583
KEGQUL	$P2_1$	9.4312	11.673	14.725	90	100.851	90	2017	584
KEKTOK	$P2_1$	10.087	5.4955	14.843	90	97.321	90	2006	585
KELBOV	$P2_12_12_1$	7.0773	8.1344	21.313	90	90	90	2017	586
KELJAM	$P2_1$	15.565	24.386	15.634	90	109.24	90	1999	587
KENPUR	$P2_12_12_1$	8.7468	12.105	14.474	90	90	90	2017	588
KEPHIY	$P2_12_12_1$	7.8321	15.876	21.612	90	90	90	2009	589
KEPKUN	$P2_12_12_1$	6.5491	15.726	16.139	90	90	90	2012	590
KEPTIL	$P2_12_12_1$	7.4013	11.185	27.578	90	90	90	2017	591
KEPTOR	$P2_1$	10.513	7.9062	13.598	90	105.832	90	2017	591
KEWMII	$P2_12_12_1$	12.783	12.752	10.544	90	90	90	1990	592
KEXRAI	$P2_1$	10.376	7.2478	10.455	90	109.713	90	2013	593
KEZYAP	$P6_1$	21.402	21.402	9.018	90	90	120	1990	594
KIBPUJ	$P2_12_12_1$	8.8358	13.074	16.772	90	90	90	2017	595
KICGAE	$P2_12_12_1$	7.253	12.756	13.199	90	90	90	1990	596
KIDDAC	$P2_12_12_1$	6.564	11.067	16.375	90	90	90	1990	597
KIGJES	$P2_1$	5.0179	9.1748	14.144	90	100.168	90	2018	598
KIGJOC	$P2_12_12_1$	5.8087	15.607	16.721	90	90	90	2018	598
KIJZIP	$P2_12_12_1$	10.027	12.78	25.956	90	90	90	2018	599
KIMKUO	$P2_12_12_1$	5.8953	10.579	12.004	90	90	90	2011	600
KIQXEN	$P3_221$	13.91	13.91	20.341	90	90	120	1999	601
KIQZEP	$P4_1$	12.644	12.644	13.65	90	90	90	2000	602
KIRGAV	$P2_12_12_1$	10.096	11.421	20.313	90	90	90	2013	603
KIRMAB	$P2_12_12_1$	11.527	14.946	18.061	90	90	90	2013	604
KIWXAR	$P2_12_12_1$	10.379	17.822	19.778	90	90	90	2013	605
KIXYOH	$P2_1$	12.264	15.988	13.652	90	91.927	90	2014	606
KIYNUD	$C2$	29.151	6.4003	7.3763	90	93.113	90	2014	607
KOBDIO	$P2_12_12_1$	10.833	13.229	18.195	90	90	90	1991	608
KOBQEX	$P2_12_12_1$	11.166	13.423	10.878	90	90	90	2000	609

CSD Code	Space Group	a / Å	b / Å	c / Å	α / °	β / °	γ / °	Publication Year	Reference
KOCWEE	$P2_12_12_1$	10.913	18.603	10.231	90	90	90	1991	[610]
KOFVEJ	$P2_12_12_1$	7.6275	11.254	16.026	90	90	90	2019	[611]
KOJGOH	$P2_12_12_1$	7.1815	8.0509	15.957	90	90	90	2014	[612]
KOJHEY	$P2_1$	9.8857	7.1169	10.74	90	93.306	90	2014	[612]
KOJNIG	$P2_12_12_1$	9.71	15.452	6.488	90	90	90	1991	[613]
KOKVOY	$P2_12_12_1$	5.7659	10.679	29.497	90	90	90	2019	[614]
KOLSUB	$P2_12_12_1$	6.4937	12.44	20.999	90	90	90	2014	[615]
KOQNIP	$P2_12_12_1$	6.0842	16.149	17.314	90	90	90	2014	[616]
KORDAY01	$P2_12_12_1$	6.7978	7.3605	29.386	90	90	90	2015	[617]
KOSPOZ	$P2_12_12_1$	7.0804	12.724	15.486	90	90	90	2014	[618]
KOSQIU	$P2_1$	9.422	6.079	15.628	90	101.009	90	2014	[618]
KOSWEW	$P2_12_12_1$	9.6641	11.731	21.73	90	90	90	2014	[619]
KOTGIJ	$P2_12_12_1$	6.0219	14.875	15.188	90	90	90	1992	[620]
KOTGIL	$P2_12_12_1$	9.45	13.149	16.973	90	90	90	2014	[618]
KOVWUN01	$P2_1$	8.031	8.16	12.323	90	92.64	90	2000	[621]
KUCGEV	$C2$	27.065	7.0704	26.322	90	114.671	90	2009	[622]
KUCGIZ	$C2$	27.857	7.1869	13.008	90	115.185	90	2009	[622]
KUHTOW	$P2_1$	7.4947	10.954	9.8199	90	98.99	90	1992	[623]
KUPYUT	$\mathrm{P22_12_1}$	6.033	11.629	12.501	90	90	90	1991	[624]
KURDUX	$P2_1$	10.052	5.299	12.976	90	91.1	90	2010	[625]
KURGAG	$P2_1$	10.475	5.4759	10.963	90	105.791	90	2010	[626]
KUSJUE	$P2_1$	6.8977	7.9761	13.068	90	99.194	90	2010	[627]
KUSKAL	$P2_12_12_1$	8.8337	9.9476	18.93	90	90	90	2010	[627]
KUSKEP	$P2_12_12_1$	8.5503	9.658	18.273	90	90	90	2010	[628]
KUSLAN	$P2_12_12_1$	9.4325	15.016	15.207	90	90	90	2015	[629]
KUTMES	$P2_12_12_1$	8.675	12.028	19.221	90	90	90	2010	[630]
KUVNUK	$P2_12_12_1$	9.419	11.3	13.844	90	90	90	1992	[631]
KUWLOE	$P2_1$	5.7533	7.4213	16.616	90	93.258	90	2010	[632]
KUXBOV	$P2_12_12_1$	6.6905	15.379	17.049	90	90	90	2010	[633]
KUXJET	$P2_12_12_1$	9.1278	13.996	16.078	90	90	90	2010	[634]
KUXJOE	$P2_12_12_1$	8.4025	8.9518	22.406	90	90	90	2015	[635]
KUXZIO	$P2_12_12_1$	8.0828	8.9952	21.181	90	90	90	2014	[636]
KUZYUA	$P2_12_12_1$	5.459	11.141	21.844	90	90	90	2010	[637]

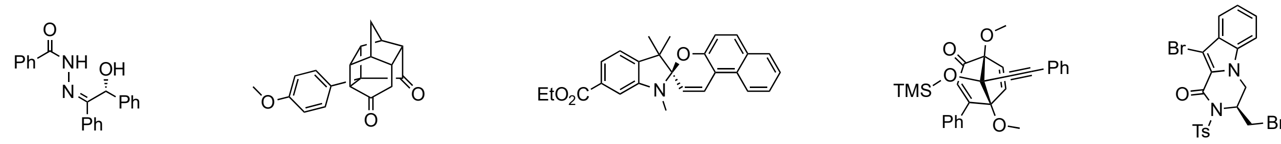

KABMUX KACLOP KACLOQ KAGTIV KAHPAN

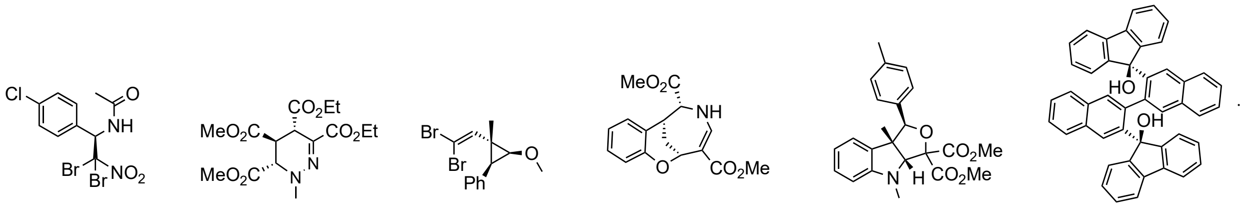

KAJQET KAKDUV KALZIH KAMNUH KAQFEP KAQMEV

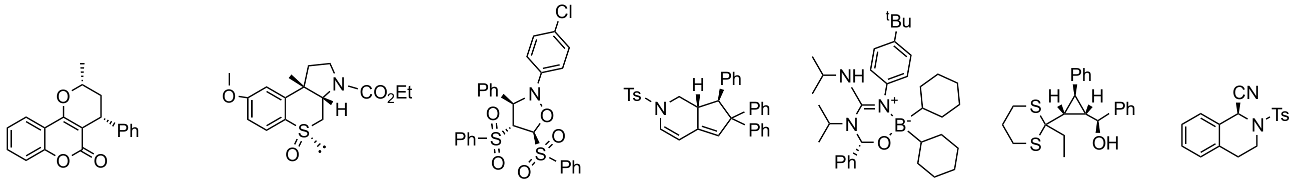

KARGOZ KATBAI KATNIE KATRAA KAWNUU KAXSIM KAYMOM

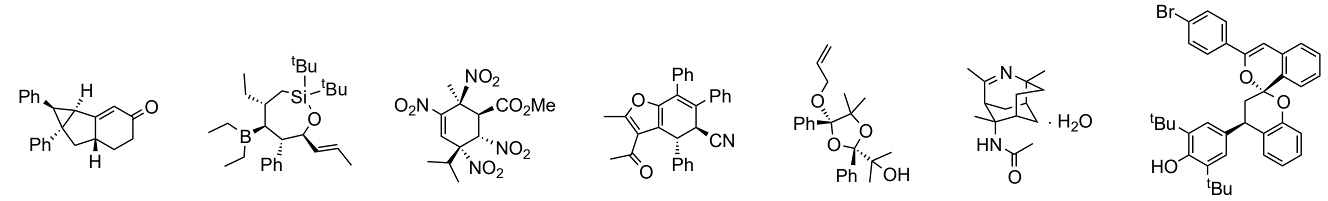

KAZHUO KEBSES KEDWUL KEFBOP KEFLIR KEGBIH KEGQUL

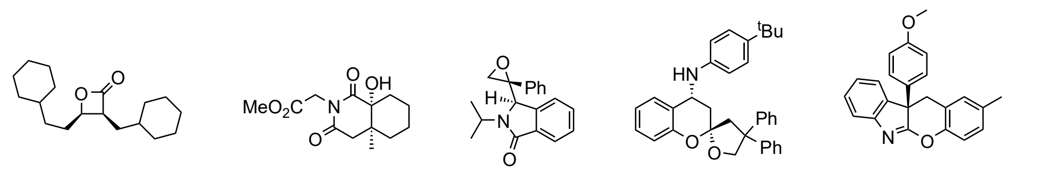

KEKTOK KELBOV KENPUR KEPHIY KEPKUN

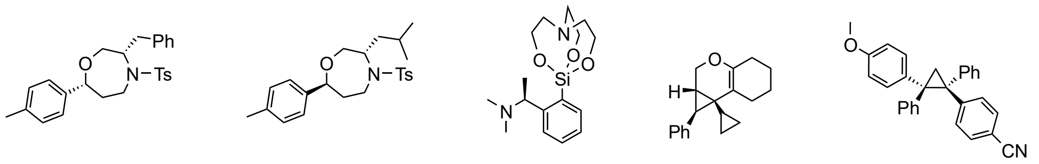

KEPTIL KEPTOR KEWMII KEXRAI KEZYAP

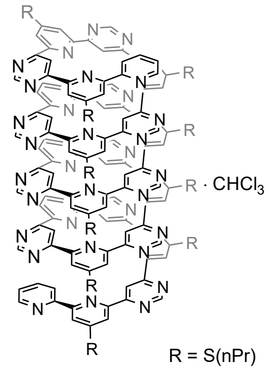

KELJAM

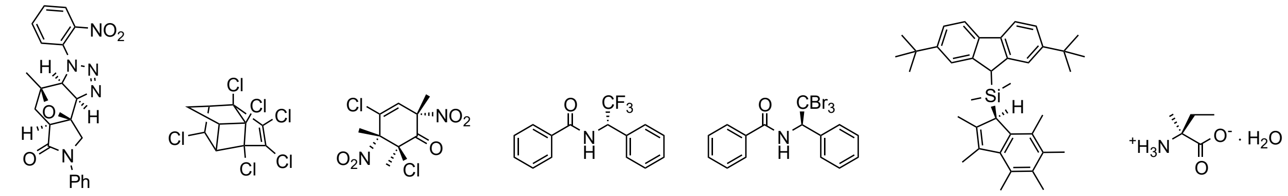

KIBPUJ KICGAE KIDDAC KIGJES KIGJOC KIJZIP KIMKUO

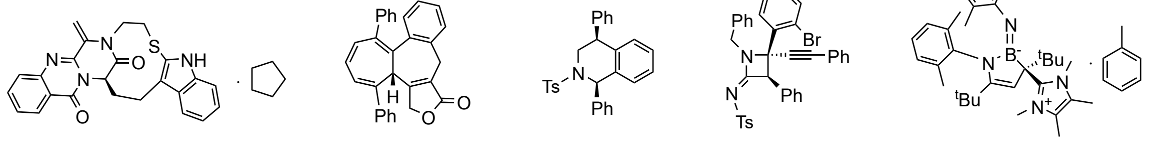

KIQXEN KIQZEP KIRGAV KIRMAB KIWXAR

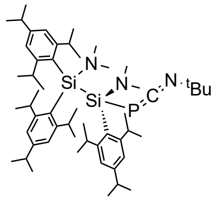
KIXYOH

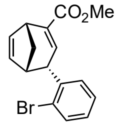
KIYNUD

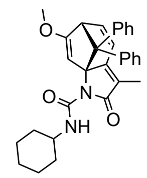
KOBDIO

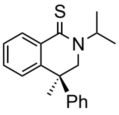
KOBQEX

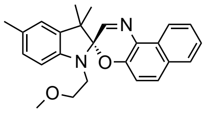
KOCWEE

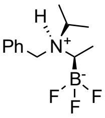
KOFVEJ

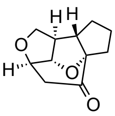
KOJGOH

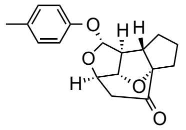
KOJHEY

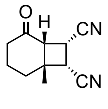
KOJNIG

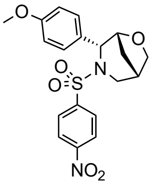
KOKVOY

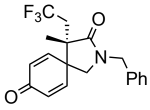
KOLSUB

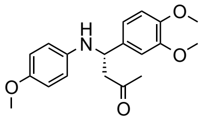
KOQNIP

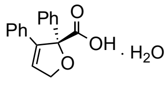
KORDAY01

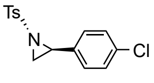
KOSPOZ

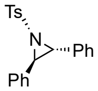
KOSQIU

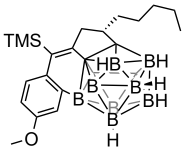
KOSWEW

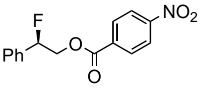
KOTGIJ

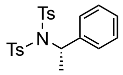
KOTGIL

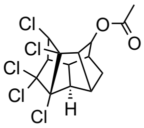
KOVWUN01

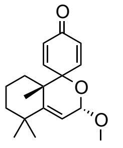
KUHTOW

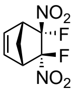
KUPYUT

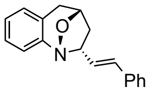
KURDUX

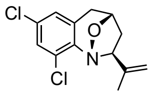
KURGAG

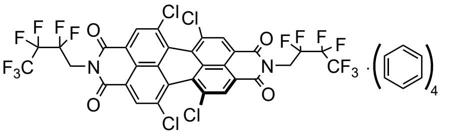
KUCGEV

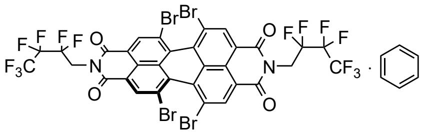
KUCGIZ

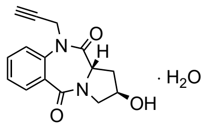
KUSJUE

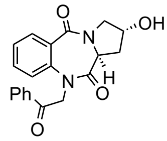
KUSKAL

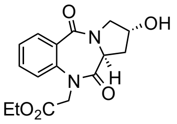
KUSKEP

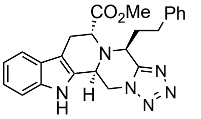
KUSLAN

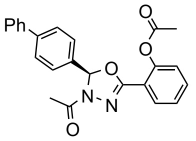
KUTMES

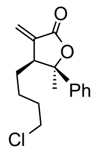
KUVNUK

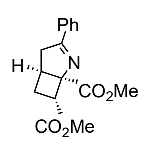
KUWLOE

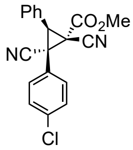
KUXBOV

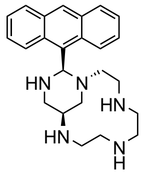
KUXJET

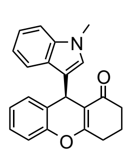
KUXJOE

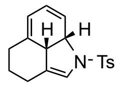
KUXZIO

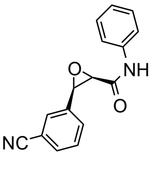
KUZYUA

CSD Code	Space Group	a / Å	b / Å	c / Å	α / °	β / °	γ / °	Publication Year	Reference
LABJUT	$P2_12_12_1$	15.991	25.458	6.0858	90	90	90	1998	[638]
LAGSIW	$P2_1$	10.575	7.6628	12.022	90	103.94	90	2004	[639]
LAHCEE	$P2_1$	5.8622	17.456	11.936	90	103.83	90	2010	[640]
LAHKUA	$P2_12_12_1$	8.288	13.254	16.193	90	90	90	1993	[641]
LAKHAG	$P2_12_12_1$	6.369	14.923	16.301	90	90	90	1993	[642]
LAKQEV	$P2_1$	9.201	7.605	12.505	90	110.476	90	2010	[643]
LALWIE	$P2_1$	8.573	10.628	6.544	90	105.55	90	1993	[644]
LAMPEV	$P2_12_12_1$	6.0476	10.18	32.026	90	90	90	2005	[645]
LANJOC	$P2_12_12_1$	5.1573	13.4	16.647	90	90	90	2017	[646]
LAQGEQ	$P2_1$	7.872	7.923	9.822	90	98.156	90	2005	[647]
LAQYAF	$P2_1$	7.995	12.695	14.1	90	103.65	90	2012	[648]
LARKUN	$P2_1$	10.815	6.3799	17.985	90	91.439	90	2017	[649]
LAVLEZ	$P2_1$	7.5589	9.3566	9.4213	90	96.521	90	1999	[650]
LAWJAX	$P2_12_12_1$	8.8272	10.132	19.563	90	90	90	2016	[651]
LAWZAL	$P2_1$	9.579	5.8454	17.241	90	93.52	90	2006	[652]
LAYHOI	$P2_12_12_1$	14.337	12.697	10.822	90	90	90	1993	[653]
LAYRUA	$P2_12_12_1$	6.689	12.251	18.106	90	90	90	2012	[654]
LECHAF	$P2_12_12_1$	9.8236	11.813	19.802	90	90	90	2017	[655]
LEFDAD	$P2_12_12_1$	8.472	10.304	25.877	90	90	90	2012	[656]
LEFSEW	$P2_12_12_1$	7.5547	11.879	22.11	90	90	90	2012	[657]
LEJFIP	$P2_12_12_1$	11.622	17.259	9.371	90	90	90	1994	[658]
LENXIN	$P2_12_12_1$	6.282	11.482	19.687	90	90	90	2012	[659]
LEQSIJ	$P2_12_12_1$	5.97	8.569	19.982	90	90	90	1998	[660]
LETREI	$P2_1$	8.545	8.875	9.964	90	113.978	90	2006	[661]
LETTUA	$C2$	15.898	5.7353	15.816	90	90.979	90	2006	[662]
LEWWUG	$P2_12_12_1$	5.717	8.6358	20.329	90	90	90	2005	[663]
LEXNAE	$P2_12_12_1$	10.682	11.154	15.43	90	90	90	2007	[664]
LICYUR	$P1$	4.6064	7.5393	16.106	91.613	96.45	98.508	1994	[665]
LIDYEC	$P2_12_12_1$	5.601	10.224	12.233	90	90	90	1994	[666]
LIJVUW	$P2_12_12_1$	5.7114	15.989	16.101	90	90	90	2007	[667]
LIKSUU	$P2_1$	12.347	5.4389	12.475	90	95.172	90	2007	[668]
LIMGOG	$P2_12_12_1$	5.1242	18.410	24.547	90	90	90	2018	[669]
LIMHAT	$P2_12_12_1$	8.885	15.643	15.771	90	90	90	2018	[669]
LIMJEY	$P2_1$	8.587	8.304	13.962	90	94.883	90	2013	[670]
LINMED	$P2_12_12_1$	5.7505	9.7182	32.097	90	90	90	2018	[671]
LINMIH	$P2_12_12_1$	5.7022	9.6372	31.486	90	90	90	2018	[671]
LIPHUN01	$P2_1$	5.895	8.771	8.769	90	91.98	90	2004	[672]
LIPMOP	$P2_12_12_1$	4.6994	11.171	25.75	90	90	90	2018	[673]
LIRROV	$P2_1$	10.227	12.263	17.169	90	94.869	90	2013	[674]
LISGOM	$P2_12_12_1$	9.6919	10.988	18.127	90	90	90	2018	[675]
LISVUG	$P2_1$	8.8514	5.9553	18.398	90	97.931	90	2013	[676]
LISWAN	$P2_1$	9.7696	5.2297	19.019	90	96.09	90	2013	[676]
LISWER	$P2_12_12_1$	5.6127	16.985	18.795	90	90	90	2013	[676]
LITMAD	$P2_12_12_1$	6.276	13.602	21.238	90	90	90	2007	[677]
LIVROA	$P2_12_12_1$	5.2857	9.201	25.718	90	90	90	2019	[678]
LIWTAN	$P2_12_12_1$	8.637	10.878	18.003	90	90	90	2008	[679]
LIYSAP	$P4_32_12$	10.058	10.058	36.133	90	90	90	2014	[680]
LIYZUP	$P2_12_12_1$	8.0598	14.871	18.048	90	90	90	2008	[681]
LIZXAV	$P1$	5.29	6.432	12.429	86.64	88.36	65.8	2014	[682]
LOBPUQ	$P2_12_12_1$	8.197	12.019	18.445	90	90	90	2019	[683]
LOBQEY	$P2_1$	7.1046	7.2949	13.314	90	93.036	90	2000	[684]
LOCFEO	$P2_12_12_1$	8.293	8.772	17.562	90	90	90	2000	[685]

CSD Code	Space Group	a / Å	b / Å	c / Å	α / °	β / °	γ / °	Publication Year	Reference
LODLOH	$P2_12_12_1$	9.327	10.719	12.261	90	90	90	2014	[686]
LODSEE	$P2_12_12_1$	5.6981	8.6185	32.841	90	90	90	2014	[687]
LONDAW	$P2_12_12_1$	5.8281	11.079	20.877	90	90	90	2019	[688]
LONVOC	$P2_12_12_1$	5.6924	13.477	18.557	90	90	90	2019	[689]
LONZOG	$P2_1$	9.1234	12.75	9.6022	90	116.321	90	2019	[690]
LOPJUX	$P2_12_12_1$	6.226	8.3026	28.059	90	90	90	2014	[691]
LOPLOU	$P2_12_12_1$	8.701	9.584	24.807	90	90	90	2019	[692]
LOQCOM	$P2_12_12_1$	9.0961	14.569	16.156	90	90	90	2019	[693]
LORZEX	$P2_12_12_1$	10.615	14.277	7.838	90	90	90	2000	[694]
LOVNEP	$P2_12_12_1$	9.999	14.127	19.022	90	90	90	2000	[695]
LUBNEB	$C2$	18.368	6.662	13.031	90	100.56	90	2002	[696]
LUCXOY	$P2_12_12_1$	7.4929	9.8033	23.509	90	90	90	2014	[697]
LUGXUG	$P2_1$	7.2775	8.1705	15.67	90	102.537	90	2002	[698]
LUHHEC	$C2$	25.134	9.5269	16.899	90	128.434	90	2009	[699]
LUHVIV	$P2_1$	8.1413	13.747	10.606	90	108.05	90	2015	[700]
LUJFUT	$P2_12_12_1$	6.1259	13.308	14.863	90	90	90	2015	[701]
LUMLUB	$P2_12_12_1$	8.8471	12.554	14.41	90	90	90	2009	[702]
LUPBII	$P2_12_12_1$	6.1103	13.388	20.371	90	90	90	2009	[703]
LUPBON	$P2_1$	8.972	6.264	12.19	90	101	90	2003	[704]
LUQJUE	$P2_12_12_1$	9.7402	11.076	23.916	90	90	90	2015	[705]
LUQSEW	$P2_12_12_1$	9.6761	10.434	14.268	90	90	90	2009	[706]
LUQZIG	$P2_1$	8.8742	10.398	10.169	90	103.437	90	2002	[707]
LUSZOO	$P2_12_12_1$	11.976	14.916	19.812	90	90	90	2002	[708]
LUTMUK	$P2_12_12_1$	5.6631	12.126	14.353	90	90	90	2015	[709]
LUZCAK	$P2_1$	8.5879	10.73	8.8188	90	117.872	90	2003	[710]
LUZGIX	$P2_12_12_1$	8.6371	10.592	21.629	90	90	90	2010	[711]

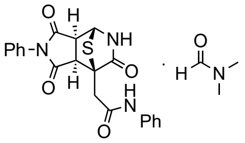
LABJUT
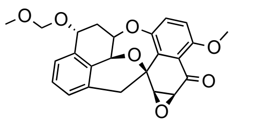
LAGSIW
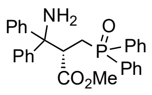
LAHCEE
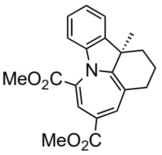
LAHKUA
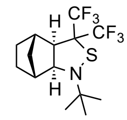
LAKHAG
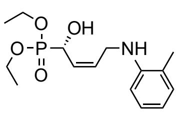
LAKQEV
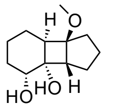
LALWIE
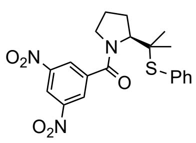
LAMPEV
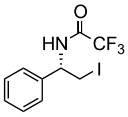
LANJOC
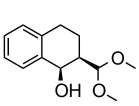
LAQGEQ
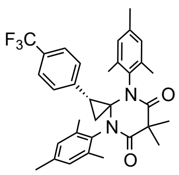
LAQYAF
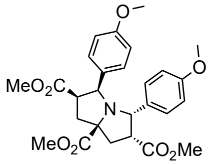
LARKUN
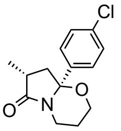
LAVLEZ
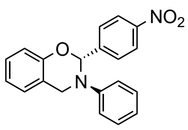
LAWJAX
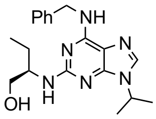
LAWZAL
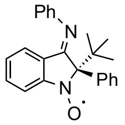
LAYHOI
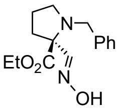
LAYRUA
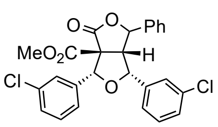
LECHAF
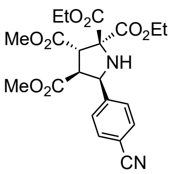
LEFDAD
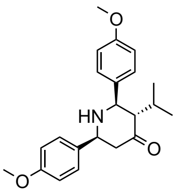
LEFSEW
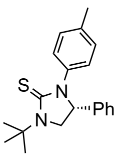
LEJFIP
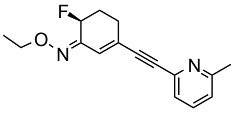
LENXIN
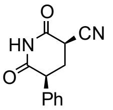
LEQSIJ
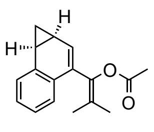
LETREI
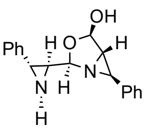
LETTUA
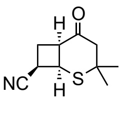
LEWWUG
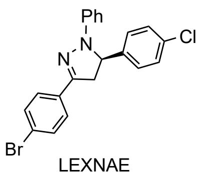
LEXNAE
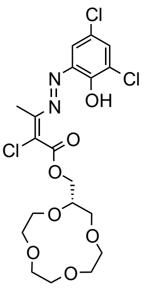
LICYUR
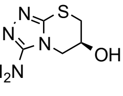
LIDYEC
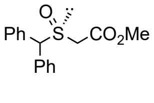
LIJVUW
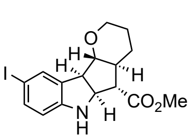
LIKSUU
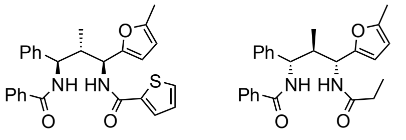
LIMGOG LIMHAT
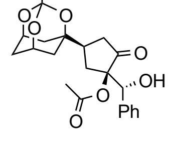
LIMJEY
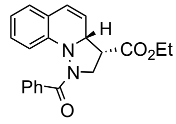
LINMED
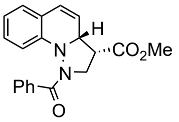
LINMIH
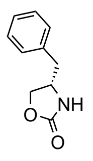
LIPHUN01
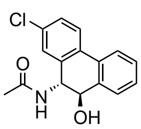
LIPMOP
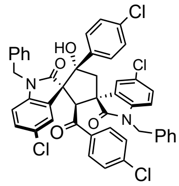
LIRROV
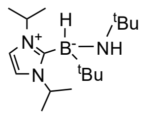
LISGOM
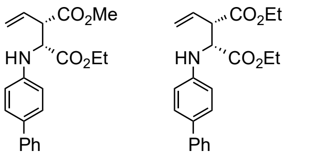
LISVUG LISWAN

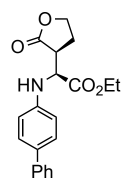

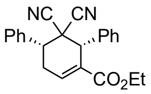

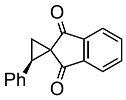

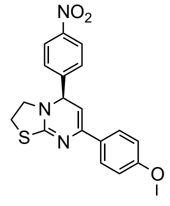

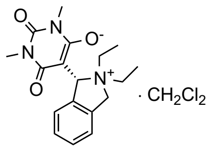

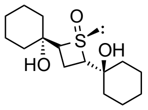

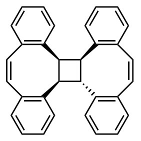

LISWER LITMAD LIVROA LIWTAN LIYSAP LIZXAV LIZYUP

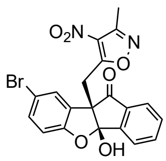

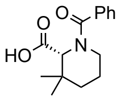

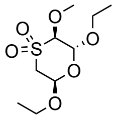

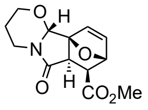

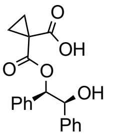

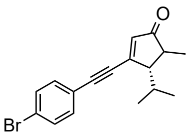

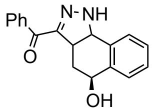

LOBPUQ LOBQEY LOCFEO LODLOH LODSEE LONDAW LONVOC

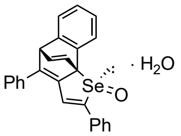

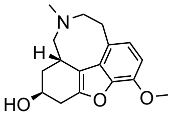

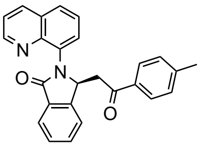

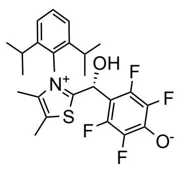

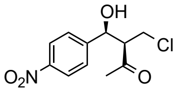

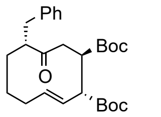

LONZOG LOPJUX LOPLOU LOQCOM LORZEX LOVNEP

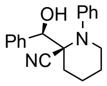

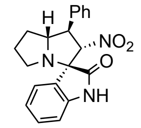

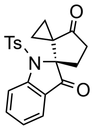

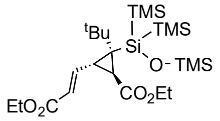

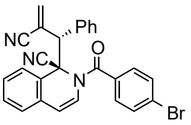

LUBNEB LUCXOY LUGXUG LUHHEC LUHVIV

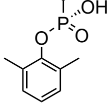

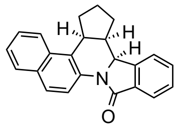

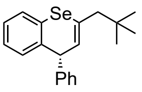

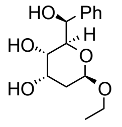

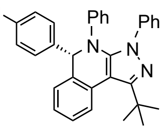

LUJFUT LUMLUB LUPBII LUPBON LUQJUE

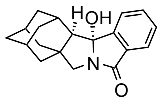

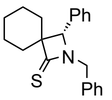

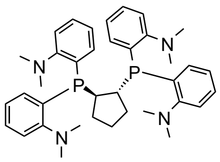

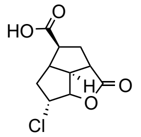

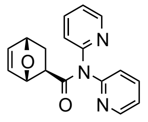

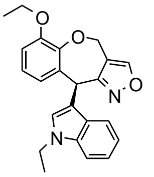

LUQSEW LUQZIG LUSZOO LUTMUK LUZCAK LUZGIX

CSD Code	Space Group	a / Å	b / Å	c / Å	α / °	β / °	γ / °	Publication Year	Reference
MACSEQ	$P2_1$	12.393	5.8186	14.691	90	111.21	90	2010	712
MAFVUL	$P2_1$	9.0495	5.5777	12.801	90	105.279	90	2003	713
MAGRAP	$P2_12_12_1$	11.001	11.945	20.385	90	90	90	2010	714
MALSOH	$P2_12_12_1$	6.735	10.529	17.909	90	90	90	2000	715
MAMHAJ	$P2_12_12_1$	9.525	14.705	24.448	90	90	90	2000	716
MANFEN	$P2_12_12_1$	6.3805	16.94	17.43	90	90	90	2005	717
MAPXUZ	$P2_12_12_1$	7.738	11.656	14.09	90	90	90	2017	718
MAQMUP	$P2_12_12_1$	7.6363	8.2848	27.736	90	90	90	2017	719
MAQNAW	$P2_1$	14.221	10.214	15.384	90	115.536	90	2017	719
MAVLOK	$P2_12_12_1$	11.25	17.59	10.69	90	90	90	1999	720
MAVLUQ	$P2_12_12_1$	9.984	24.19	7.27	90	90	90	1999	720
MAXPEH	$P2_1$	8.26	17.21	8.341	90	105.36	90	2005	721
MAYCAS	$P2_12_12_1$	10.4	25	8.29	90	90	90	2009	722
MAYFEA	$P2_12_12_1$	5.8945	15.279	15.385	90	90	90	2017	723
MAZXER	$P2_1$	5.5803	11.574	13.142	90	98.66	90	2006	724
MBABIR	$P2_12_12_1$	8.73	30.094	5.95	90	90	90	1978	725
MCHTBI	$P2_1$	8.929	15.612	5.813	90	93.05	90	1979	726
MCPXZN10	$P2_12_12_1$	9.01	11.113	12.15	90	90	90	1979	727
MCTHPY10	$P2_1$	11.36	8.052	11.77	90	99.86	90	1972	728
MEBGIK	$P2_12_12_1$	8.1881	12.713	14.239	90	90	90	2006	729
MEBHEH	$P2_12_12_1$	9.961	10.677	14.073	90	90	90	2006	730
MEBMIP	$P2_12_12_1$	18.243	9.947	9.685	90	90	90	1999	731
MEHNAP	$P2_12_12_1$	7.7404	10.023	20.562	90	90	90	2006	732
MEHPIY	$P2_1$	10.255	7.1019	13.085	90	93.787	90	1999	733
MEJFEM	$P2_1$	7.8265	7.7477	10.926	90	92.497	90	1999	734
MEJMIY	$P2_12_12_1$	9.676	12.826	17.434	90	90	90	2006	735
MEJTUS	$P2_12_12_1$	5.551	11.084	24.666	90	90	90	2012	736
MELBUB	$P2_1$	6.22	10.057	14.276	90	95.969	90	2006	737
MELKAP	$P1$	6.068	7.8228	10.863	73.813	83.34	81.93	2000	738
MEMQON	$P2_1$	6.3211	8.5298	20.397	90	90.395	90	2017	739
MEMTUW	$P2_1$	11.01	9.1251	11.497	90	91.296	90	2017	740
MEPZTC	$P2_1$	11.249	7.498	10.272	90	90.91	90	1976	741
MESHUO	$P2_12_12_1$	5.71	9.925	16.153	90	90	90	2006	742
MEWPUB	$P2_1$	9.4059	6.5009	12.492	90	105.914	90	2013	743
MEZKUZ	$P2_1$	7.5236	9.5911	8.5262	90	105.191	90	2012	744
MEZPEO	$P2_1$	8.189	7.185	11.259	90	93.555	90	2012	745
MFSUDT	$P2_12_12_1$	9.052	10.439	19.561	90	90	90	1979	746
MICHIP	$P2_12_12_1$	15.012	17.109	19.453	90	90	90	2001	747
MIFKER	$P2_1$	9.98	13.182	13.318	90	103.26	90	2002	748
MIFVOM	$P2_12_12_1$	6.1961	13.613	15.842	90	90	90	2002	749
MIGBAI	$P2_12_12_1$	7.9398	12.128	22.14	90	90	90	2018	750
MIHRAY	$P2_12_12_1$	12.095	13.613	17.988	90	90	90	2014	751
MIJDIT	$P2_12_12_1$	7.4451	14.812	16.314	90	90	90	2007	752
MIJKEY	$P2_12_12_1$	5.6951	15.752	21.571	90	90	90	2018	753
MIJPUT	$P2_12_12_1$	11.118	11.273	18.127	90	90	90	2018	753
MIJQAA	$P2_1$	9.596	12.151	10.024	90	103.007	90	2018	753
MILWAG	$P2_1$	9.795	8.637	9.836	90	103.874	90	2007	754
MIMLIE	$P2_12_12_1$	6.425	12.313	21.84	90	90	90	2007	755
MIMREG	$P2_12_12_1$	8.521	9.176	17.747	90	90	90	2007	754
MINHEX	$P2_12_12_1$	8.2484	10.778	13.784	90	90	90	2007	756
MIQQIN	$P2_12_12_1$	9.932	11.444	20.64	90	90	90	2007	757
MISQUA	$P2_12_12_1$	7.62	9.01	15.94	90	90	90	2002	758

CSD Code	Space Group	a / Å	b / Å	c / Å	α / °	β / °	γ / °	Publication Year	Reference
MITXAO	$P2_12_12_1$	4.662	12.888	28.008	90	90	90	2002	[759]
MIVZOH	$P2_1$	10.334	11.604	10.833	90	102.721	90	2008	[760]
MOCDAK	$P2_12_12_1$	6.6794	9.7679	20.085	90	90	90	2008	[761]
MODSEG	$P2_1$	8.029	8	10.088	90	108.606	90	2019	[762]
MOFSUW	$P2_12_12_1$	7.171	11.742	14.709	90	90	90	2008	[763]
MONFIE	$P2_12_12_1$	7.16	9.656	13.74	90	90	90	2002	[764]
MOTKOX	$P2_12_12_1$	5.455	11.14	22.564	90	90	90	2015	[765]
MOVFOT	$P2_1$	7.1324	7.417	18.265	90	101.203	90	2009	[766]
MOVHEL	$P2_1$	7.8658	8.6374	15.382	90	99.533	90	2009	[766]
MOVJUD	$P2_1$	8.2702	7.8909	10.76	90	95.892	90	2008	[767]
MOYHUD	$P2_12_12_1$	6.528	33.655	5.965	90	90	90	2002	[768]
MPATHA	$P2_1$	12.238	6.66	9.034	90	96.4	90	1980	[769]
MUBKEA	$P2_1$	5.98	5.196	21.503	90	96.105	90	2009	[770]
MUDDIY	$P2_1$	5.8358	9.285	9.343	90	96.96	90	2002	[771]
MUJKUZ	$P2_12_12_1$	10.754	11.235	13.407	90	90	90	2015	[772]
MUJWAR01	$P2_1$	9.1662	5.7246	13.918	90	90.153	90	2015	[773]
MUMDOP	$P2_12_12_1$	7.4464	12.118	17.213	90	90	90	2015	[774]
MUMXEY	$P2_12_12_1$	10.562	11.613	15.592	90	90	90	2009	[775]
MUSDOT	$P2_12_12_1$	13.8	16.213	19.088	90	90	90	2003	[776]
MUZVOT	$P2_12_12_1$	5.4192	8.4005	26.631	90	90	90	2010	[777]
MXPOXZ10	$P2_1$	13.364	5.763	14.599	90	111.17	90	1983	[778]
MXTDOB	$P2_1$	12.211	6.371	8.796	90	111.65	90	1979	[779]

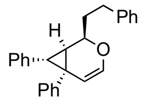

MACSEQ

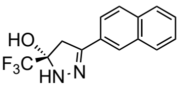

MAFVUL

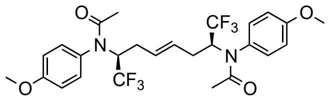

MAGRAP

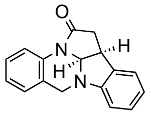

MALSOH

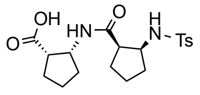

MANFEN

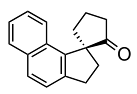

MAPXUZ

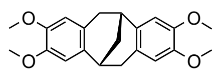

MAQMUP

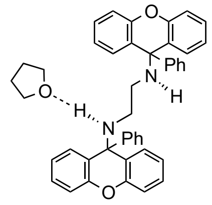

MAMHAJ

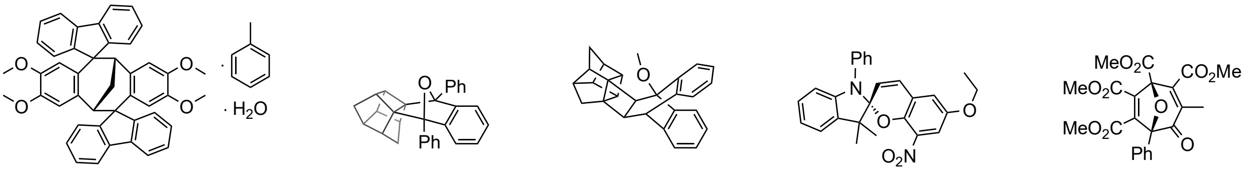

MAQNAW MAVLOK MAVLUQ MAXPEH MAYCAS

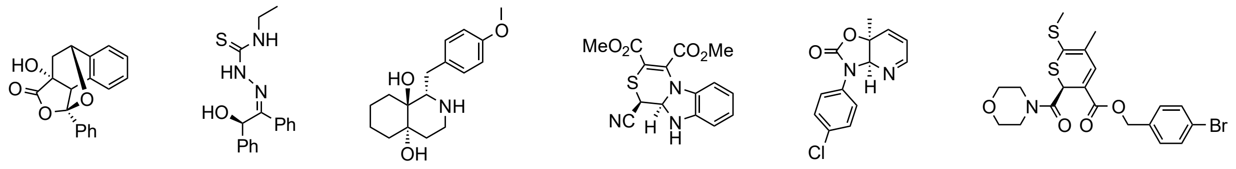

MAYFEA MAZXER MBABIR MCHTBI MCPXZN10 MCTHPY10

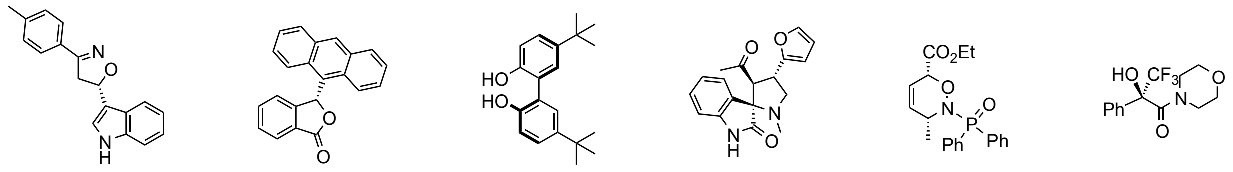

MEBGIK MEBHEH MEBMIP MEHNAP MEHPIY MEJFEM

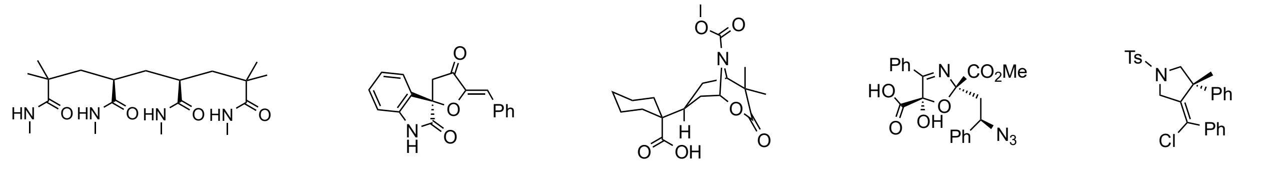

MEJMIY MEJTUS MELBUB MELKAP MEMQON

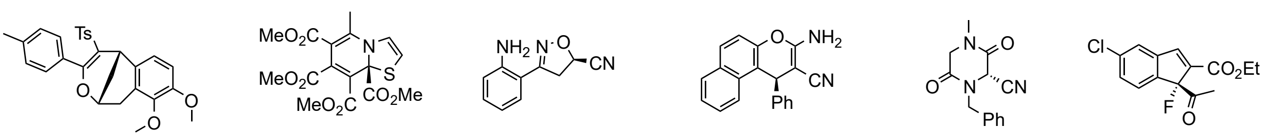

MEMTUW MEPZTC MESHUO MEWPUB/MUJWAR01 MEZKUZ MEZPEO

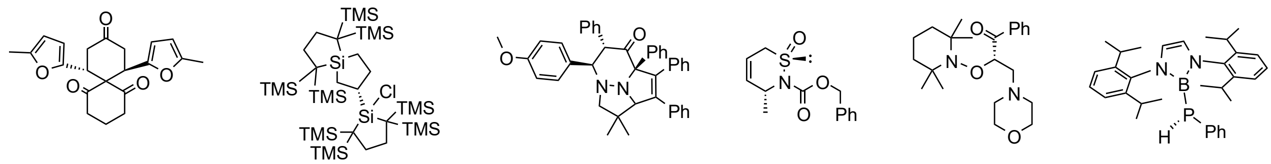

MFSUDT MICHIP MIFKER MIFVOM MIGBAI MIHRAY

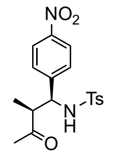
MIJDIT

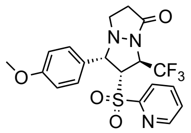
MIJKEY

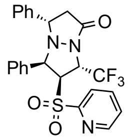
MIJPUT

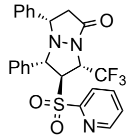
MIJQAA

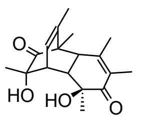
MILWAG

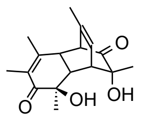
MIMREG

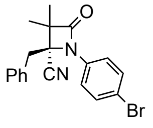
MIMLIE

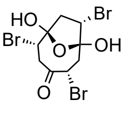
MINHEX

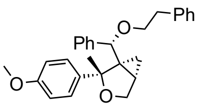
MIQQIN

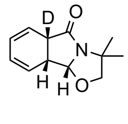
MISQUA

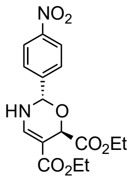
MITXAO

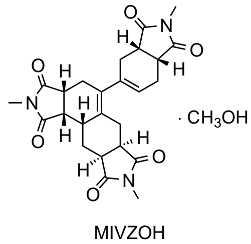
MIVZOH

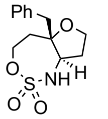
MOCDAK

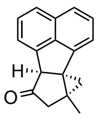
MODSEG

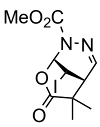
MOFSUW

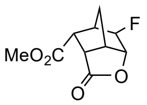
MONFIE

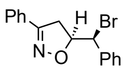
MOTKOX

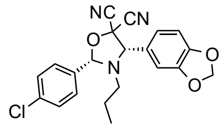
MOVFOT

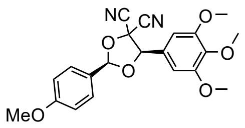
MOVHEL

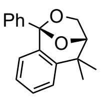
MOVJUD

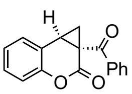
MOYHUD

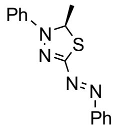
MPATHA

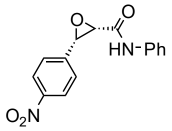
MUBKEA

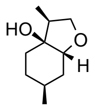
MUDDIY

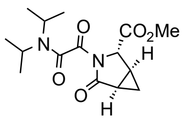
MUJKUZ

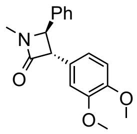
MUMDOP

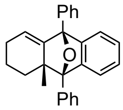
MUMXEY

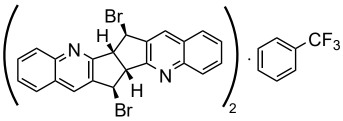
MUSDOT

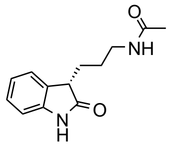
MUZVOT

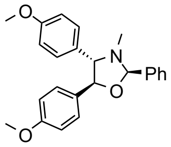
MXPOXZ10

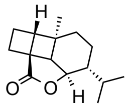
MXTDOB

CSD Code	Space Group	a / Å	b / Å	c / Å	α / °	β / °	γ / °	Publication Year	Reference
NAJFEJ	$P2_12_12_1$	9.4897	10.516	24.582	90	90	90	1996	780
NAPCEP	$P2_12_12_1$	7.6551	11.731	23.996	90	90	90	2016	781
NAPNUO	$P2_12_12_1$	4.6278	10.575	27.488	90	90	90	2005	782
NAWBOF	$P2_1$	5.8766	7.9602	16.648	90	94.4336	90	2017	783
NAWBUL	$P2_12_12_1$	8.256	12.073	15.125	90	90	90	2017	783
NAWYIV	$P1$	8.2762	9.5852	12.597	82.36	80.27	77.15	2011	784
NAXTUD	$P2_1$	9.56	9.3119	10.968	90	111.27	90	2012	785
NAYWIT	$P2_1$	10.892	8.687	13.621	90	109.15	90	1997	786
NEBMAK	$P2_1$	7.314	12.361	10.212	90	110.02	90	2012	787
NECFEJ	$P2_1$	5.868	19.876	7.5288	90	92.133	90	2017	788
NEFQAS	$P2_12_12_1$	8.288	10.858	19.168	90	90	90	2012	789
NEJTAX	$P2_12_12_1$	10.748	16.23	9.765	90	90	90	1997	790
NEJZOR	$P2_12_12_1$	10.153	17.535	17.751	90	90	90	1997	791
NELNUQ	$P2_12_12_1$	5.7561	10.135	20.636	90	90	90	2017	792
NEMQOO	$P2_1$	5.8384	7.951	18.153	90	97.022	90	2013	793
NENKEX	$P2_1$	4.0243	14.312	11.111	90	94.002	90	2006	794
NENXAF	$P2_12_12_1$	4.4209	7.95	22.473	90	90	90	2001	795
NERSAH	$I2$	13.539	5.6134	19.199	90	107.79	90	2017	796
NESDAT	$P2_1$	5.774	14.657	12.222	90	101.02	90	2017	797
NESGUQ	$P2_12_12_1$	5.1885	8.975	25.944	90	90	90	2017	798
NESLUS	$P2_12_12_1$	9.433	9.561	38.142	90	90	90	1997	799
NETHAY	$C2$	30.091	6.1991	12.79	90	101.201	90	2017	800
NEXNEK	$P2_12_12_1$	7.106	12.491	23.387	90	90	90	2006	801
NIGQIE	$P2_12_12_1$	5.968	12.151	19.327	90	90	90	2007	802
NIHKAR	$P2_12_12_1$	9.3604	9.9285	17.935	90	90	90	2007	803
NIJMAW	$P2_12_12_1$	7.0401	9.8225	22.412	90	90	90	2013	804
NIJPIF	$P2_12_12_1$	11.082	15.518	8.7868	90	90	90	1997	805
NIKYEM	$P2_12_12_1$	5.9299	8.176	27.17	90	90	90	2007	806
NILXAH	$P2_12_12_1$	12.021	20.149	6.655	90	90	90	1998	807
NILYOY	$P2_12_12_1$	8.3228	11.055	21.599	90	90	90	2013	808
NIMLUR	$C2$	25.453	12.571	17.357	90	131.308	90	2007	809
NIPLOQ	$P2_1$	10.273	7.9593	10.591	90	113.069	90	2018	810
NIQHED	$P2_1$	10.801	7.1714	12.542	90	101.366	90	2018	811
NISNAE	$P2_1$	4.006	11.489	7.347	90	97.5	90	1998	812
NIVMUB	$P2_1$	7.9098	16.815	8.1247	90	91.953	90	2008	813
NIVYOJ	$P2_12_12_1$	8.1053	10.435	12.4	90	90	90	2019	814
NIWQEQ	$P2_12_12_1$	7.564	14.549	19.397	90	90	90	2008	815
NIYSIA	$P1$	7.9305	8.026	10.937	73.997	75.084	62.95	2019	816
NIZSOH	$P2_1$	9.9964	10.129	10.117	90	97.676	90	2019	817
NOBKIB	$P2_12_12_1$	9.365	9.465	18.699	90	90	90	2019	818
NOBLAR	$P2_12_12_1$	11.078	14.57	14.596	90	90	90	1998	819
NOCNIC	$C2$	22.236	7.574	15.041	90	95.13	90	1998	820
NOFQEH	$P2_12_12_1$	8.8858	11.097	19.011	90	90	90	2019	821
NOGLIH	$P2_12_12_1$	7.08	8.7733	28.321	90	90	90	2019	822
NOHDAS	$P2_12_12_1$	9.3042	11.117	15.169	90	90	90	2019	823
NOHSEK	$P2_1$	7.618	5.9682	14.103	90	90.846	90	2014	824
NOHZOB	$P2_1$	10.276	8.6059	10.538	90	118.016	90	2014	825
NONDAW	$P2_12_12_1$	10.629	12.942	21.764	90	90	90	2008	826
NONFRA	$P2_12_12_1$	8.439	7.423	18.032	90	90	90	1980	827
NOPLAG	$P2_1$	12.655	6.4943	15.007	90	109.347	90	2008	828
NOPMEL	$P2_12_12_1$	8.3315	13.992	18.291	90	90	90	2008	829
NORJEK	$P2_12_12_1$	5.796	9.924	20.209	90	90	90	2009	830

CSD Code	Space Group	a / Å	b / Å	c / Å	α / °	β / °	γ / °	Publication Year	Reference
NOSXOI	$P2_12_12_1$	6.923	8.291	21.551	90	90	90	1998	[831]
NOSXUO	$P2_12_12_1$	8.662	9.551	17.649	90	90	90	1998	[831]
NOTHEL	$P2_1$	6.8289	13.514	12.142	90	106.143	90	2015	[832]
NUBPAD	$P2_12_12_1$	10.438	19.318	22.887	90	90	90	2015	[833]
NUCYUG	$P2_12_12_1$	5.8601	11.043	24.76	90	90	90	2009	[834]
NUFLOQ	$P2_1$	9.2273	10.87	10.451	90	110.996	90	2009	[835]
NUGWAO	$P2_12_12_1$	5.609	9.854	27.272	90	90	90	2009	[836]
NUKHIM	$P2_1$	5.549	8.7759	19.443	90	92.815	90	2015	[837]
NUKZUQ	$P2_12_12_1$	5.8678	15.063	25.104	90	90	90	2015	[838]
NULZEA	$P4_12_12$	9.891	9.891	17.167	90	90	90	2009	[839]
NUMRET	$P4_1$	10.6	10.6	14.863	90	90	90	2009	[840]
NUNNOZ	$P2_1$	8.664	10.64	8.822	90	108.38	90	1998	[841]
NURFEM	$P2_12_12_1$	5.5714	11.018	18.753	90	90	90	2010	[842,843]
NURHOY	$P2_1$	10.556	10.498	11.426	90	103.691	90	2010	[844]
NURHUE	$P2_12_12_1$	8.5061	12.797	19.474	90	90	90	2010	[844]
NURYEE	$P2_12_12_1$	12.888	16.806	10.156	90	90	90	1998	[845]
NUTSEA	$P2_12_12_1$	11.072	11.493	18.908	90	90	90	1998	[846]
NUWKUM	$P2_12_12_1$	9.3902	10.694	18.817	90	90	90	2009	[847]
NUWNOI	$P2_1$	10.375	6.482	12.292	90	102.304	90	1997	[848]
NUWNUP	$P2_1$	11.714	9.0109	11.85	90	102.577	90	2009	[849]
NUYSOR	$P2_12_12_1$	7.7273	11.351	16.776	90	90	90	2015	[850]

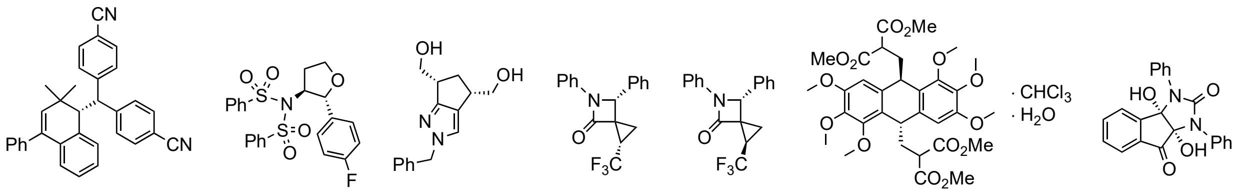

NAJFEJ NAPCEP NAPNUO NAWBOF NAWBUL NAWYIV NAXTUD

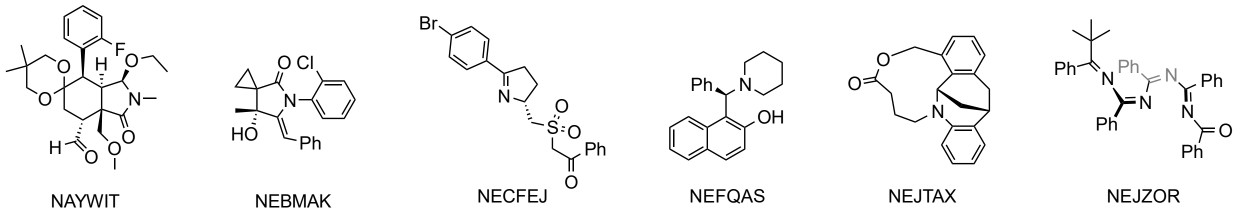

NAYWIT NEBMAK NECFEJ NEFQAS NEJTAX NEJZOR

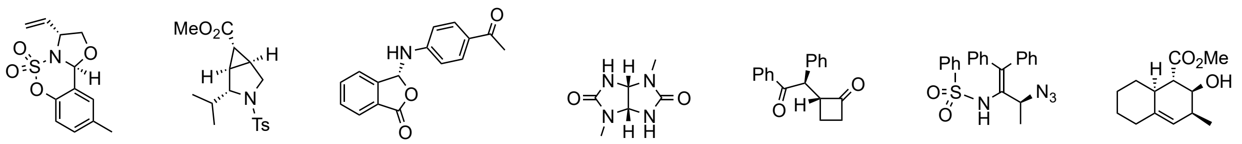

NELNUQ NEMQOO NENKEX NENXAF NERSAH NESDAT NESGUQ

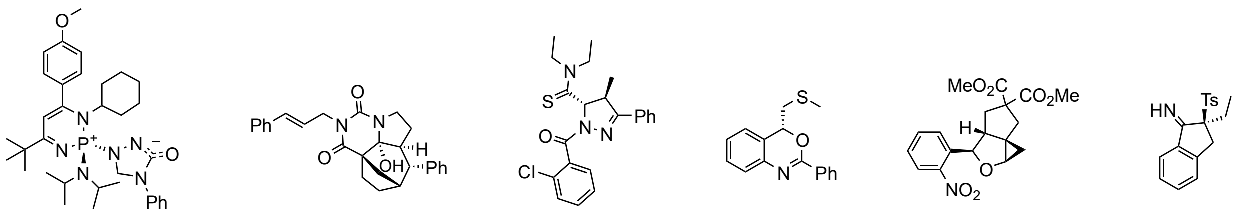

NESLUS NETHAY NEXNEK NIGQIE NIHKAR NIJMAW

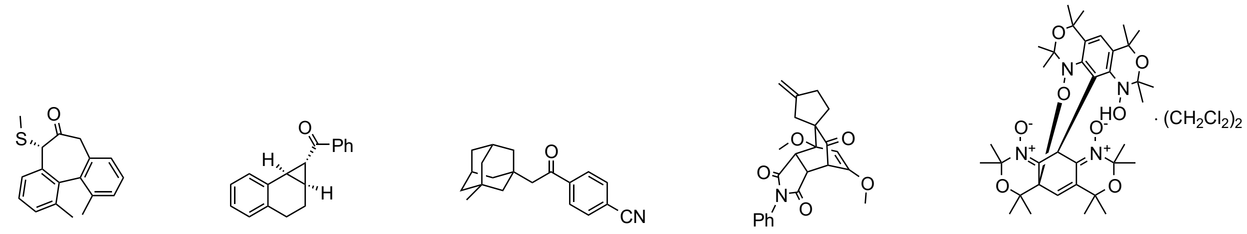

NIJPIF NIKYEM NILXAH NILYOY NIMLUR

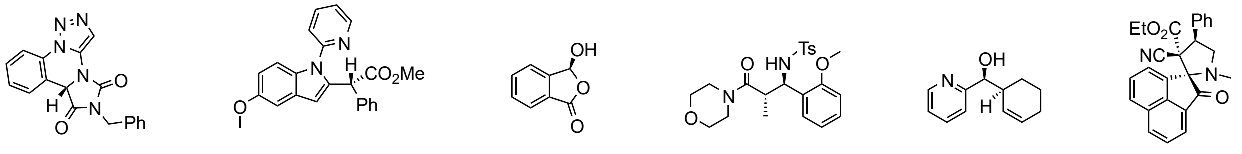

NIPLOQ NIQHED NISNAE NIVMUB NIVYOJ NIWQEQ

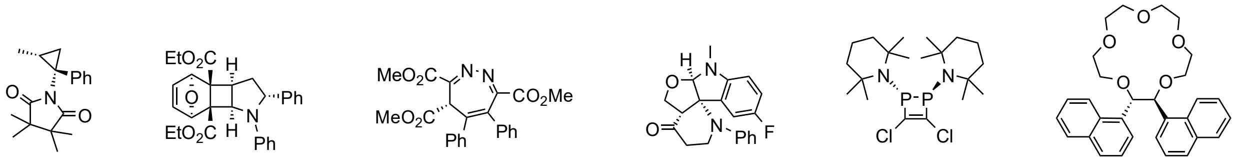

NIWTOF NIYSIA NIZSOH NOBKIB NOBLAR NOCNIC

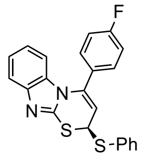

NOFQEH

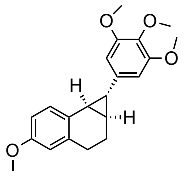

NOGLIH

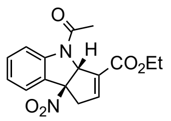

NOHDAS

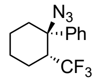

NOHSEK

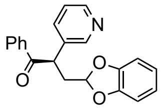

NOHZOB

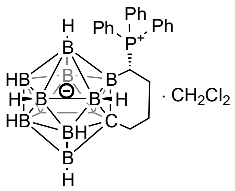

NONDAW

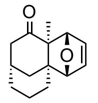

NONFRA

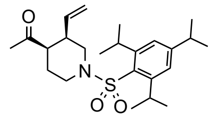

NOPLAG

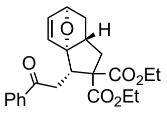

NOPMEL

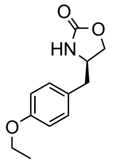

NORJEK

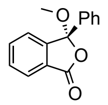

NOSXOI

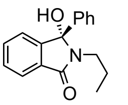

NOSXUO

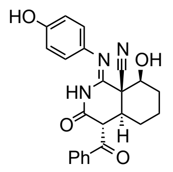

NOTHEL

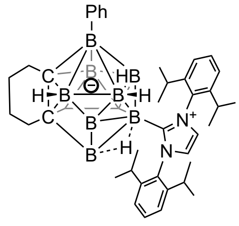

NUBPAD

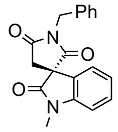

NUCYUG

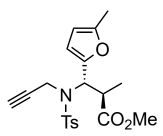

NUFLOQ

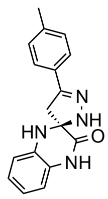

NUGWAO

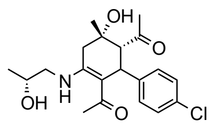

NUKHIM

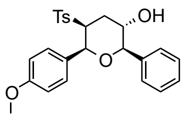

NUKZUQ

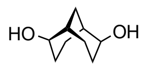

NULZEA

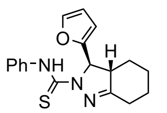

NUMRET

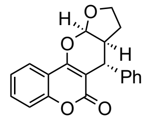

NUNNOZ

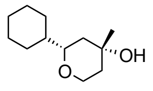

NURFEM

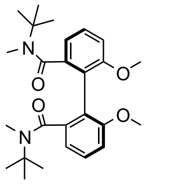

NURHOY

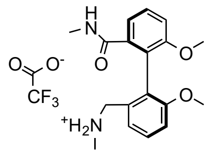

NURHUE

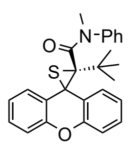

NURYEE

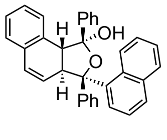

NUTSEA

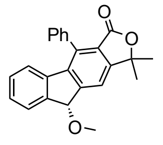

NUWKUM

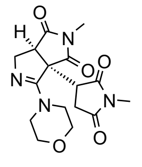

NUWNOI

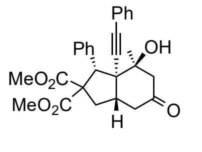

NUWNUP

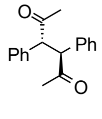

NUYSOR

CSD Code	Space Group	a / Å	b / Å	c / Å	α / °	β / °	γ / °	Publication Year	Reference
OBIPAR	$C2$	22.928	8.3801	12.929	90	91.893	90	2004	851
OCELOZ	$C2$	10.495	6.283	19.247	90	97.029	90	2011	852
ODOVUZ	$P2_1$	7.7229	11.844	12.022	90	100.828	90	2007	853
OFAPUG	$P2_1$	11.527	6.0597	12.781	90	104.32	90	2002	854
OFETAV	$P2_1$	8.2626	12.979	10.325	90	107.622	90	2007	853
OFUZUM	$P2_1$	9.2228	7.4537	16.957	90	96.151	90	2013	855
OGETOM	$P2_12_12_1$	10.058	11.341	17.361	90	90	90	2018	856
OGODEV	$P2_12_12_1$	9.833	10.073	23.348	90	90	90	2015	857
OGUZOH	$P2_12_12_1$	7.8538	14.061	16.416	90	90	90	2015	858
OHADAC	$P2_12_12_1$	8.0291	12.978	20.36	90	90	90	2002	859
OHADEG	$P2_12_12_1$	8.0658	12.969	21.015	90	90	90	2002	859
OHAWOJ	$P2_1$	11.856	6.471	12.272	90	94.886	90	2002	860
OHNODC	$P2_1$	10.992	5.647	8.597	90	103	90	1981	861
OHOVOY	$P2_12_12_1$	8.4903	9.6656	18.663	90	90	90	2015	862
OICMTH	$P2_1$	8.324	10.982	7.271	90	93.12	90	1971	863
OJEGIU	$P2_12_12_1$	11.069	14.681	13.91	90	90	90	2009	864
OKAFEL	$P2_12_12_1$	5.254	12.698	17.938	90	90	90	2002	865
OKAWED	$P2_12_12_1$	8.8087	8.8087	12.066	90	90	90	2011	866
OKAYUV	$P2_12_12_1$	8.155	9.8788	16.812	90	90	90	2011	867
OKOKEF	$P2_12_12_1$	8.6041	18.186	19.188	90	90	90	2010	868
OKOTEP	$P2_1$	8.431	12.486	10.251	90	90.558	90	2016	869
OKUCAY	$P2_1$	9.5203	7.1738	11.291	90	92.251	90	2003	870
OKUCEC	$P2_12_12_1$	6.1964	13.703	16.864	90	90	90	2003	870
OLESAA	$P2_12_12_1$	5.5701	6.8642	32.453	90	90	90	2011	871
OLESOO	$P2_12_12_1$	5.577	15.715	18.402	90	90	90	2011	872
OMEHOE	$P2_12_12_1$	6.1344	14.69	14.987	90	90	90	2010	873
OMEMOJ	$P2_1$	11.304	7.763	13.644	90	99.568	90	2010	874
OMENEA	$P2_1$	10.781	7.655	15.097	90	104.28	90	2010	874
OMENUQ	$P2_12_12_1$	7.801	12.547	25.089	90	90	90	2010	874
OMINEE	$P2_12_12_1$	5.5473	18.097	18.114	90	90	90	2010	875
OMORUF	$P2_1$	9.4625	6.1999	15.917	90	107.15	90	2016	876
OMUMEP	$P2_12_12_1$	7.521	9.936	18.87	90	90	90	2010	877
OMUYOM	$P2_1$	11.667	5.7483	14.77	90	103.841	90	2016	878
ONILIH	$P1$	6.5748	8.8652	10.763	69.163	82.406	82.35	2010	879
OPEGUN	$P2_1$	12.255	4.962	15.283	90	109.783	90	2015	880
OPEXUE	$P2_1$	8.6937	8.2505	9.8923	90	96.8229	90	2016	881
OPIHEC	$P2_1$	12.263	7.2788	12.339	90	104.51	90	2016	882
OPOHAE	$P2_12_12_1$	6.1199	10.713	23.819	90	90	90	2014	883
OPOZAW	$P2_12_12_1$	8.2715	10.427	19.179	90	90	90	2013	884
OQATOQ	$P2_12_12_1$	11.359	18.503	20.27	90	90	90	2011	885
OQOHOT	$P2_1$	5.7189	8.7327	14.379	90	97.71	90	2016	886
OQOZOL	$P2_1$	9.5951	4.8146	15.262	90	101.578	90	2010	887
OREPAD	$P2_12_12_1$	6.8534	9.8732	27.541	90	90	90	2011	888
ORIFEC	$P2_12_12_1$	9.53	17.096	19.184	90	90	90	2016	889
OSEFOJ	$P2_1$	10.023	7.569	13.421	90	108.949	90	2016	890
OSUSOL	$P2_12_12_1$	9.7747	11.045	24.484	90	90	90	2011	891
OTEREM	$P2_12_12_1$	5.5419	8.9082	26.856	90	90	90	2016	892
OTOHIP	$P2_12_12_1$	6.947	14.989	18.031	90	90	90	2011	893
OTOLIT	$P2_12_12_1$	8.6992	12.056	19.17	90	90	90	2010	894
OVAZUI	$P2_12_12_1$	7.7393	10.418	19.775	90	90	90	2016	895
OVEBEY	$P2_12_12_1$	7.5591	10.272	21.727	90	90	90	2016	895
OWOGOY	$P2_12_12_1$	5.6457	17.515	18.278	90	90	90	2016	896

CSD Code	Space Group	a / Å	b / Å	c / Å	α / °	β / °	γ / °	Publication Year	Reference
OXAZLB	$P2_12_12_1$	9.424	12.774	16.552	90	90	90	1981	[897]
OXECOL	$P2_12_12_1$	7.961	11.086	13.374	90	90	90	2016	[898]
OXUCER	$P2_12_12_1$	6.9578	11.041	24.685	90	90	90	2016	[899]
OZUPUW	$P2_12_12_1$	6.1949	10.967	23.453	90	90	90	2016	[900]

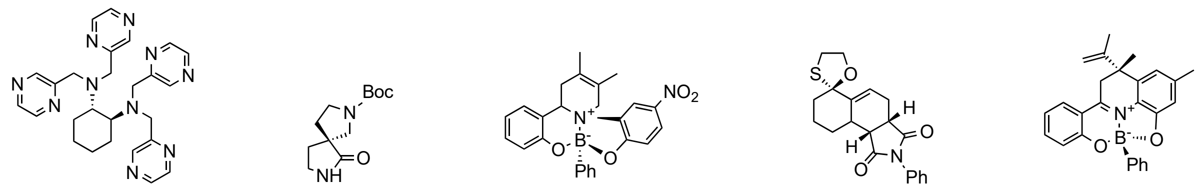

OBIPAR OCELOZ ODOVUZ OFAPUG OFETAV

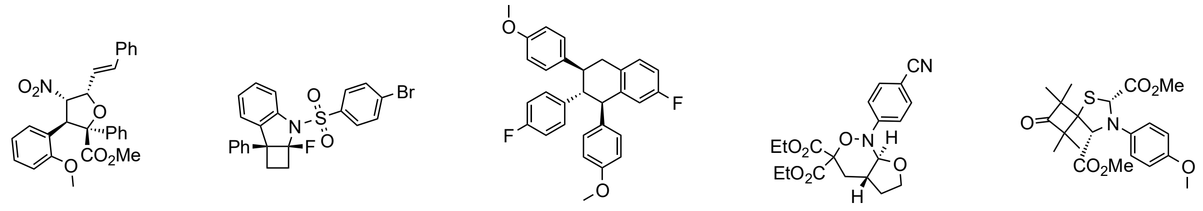

OFUZUM OGETOM OGODEV OGUZOH OHADAC

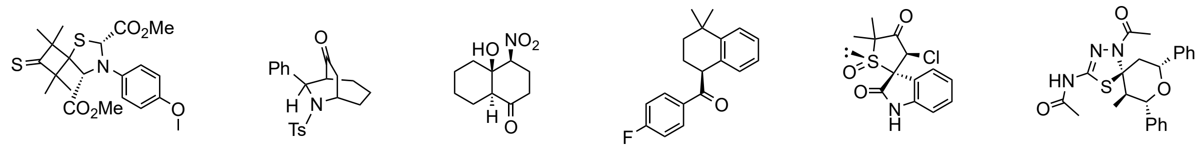

OHADEG OHAWOJ OHNODC OHOVOY OICMTH OJEGIU

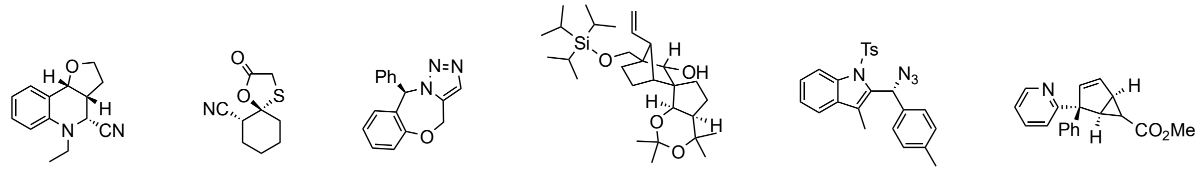

OKAFEL OKAWED OKAYUV OKOKEF OKOTEP OKUCAY

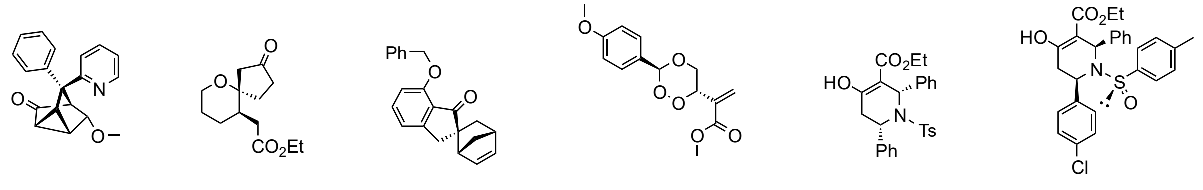

OKUCEC OLESAA OLESOO OMEHOE OMEMOJ OMENEA

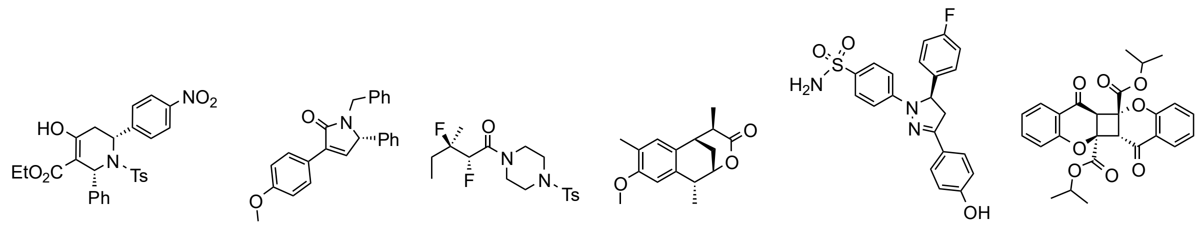

OMENUQ OMINEE OMORUF OMUMEP OMUYOM ONILIH

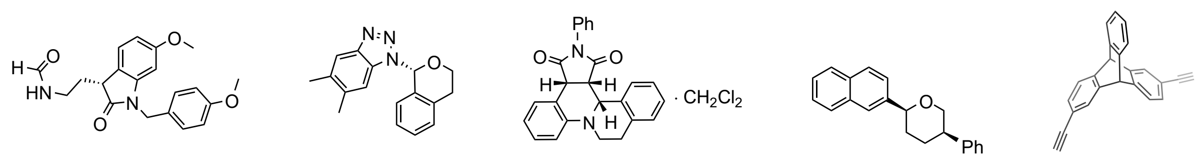

OPEGUN OPEXUE OPIHEC OPOHAE OPOZAW

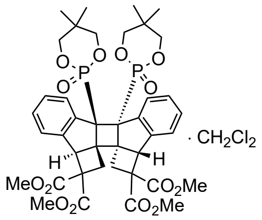

OQATOQ

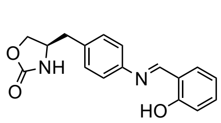

OQOHOT

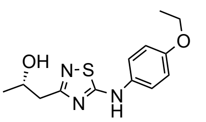

OQOZOL

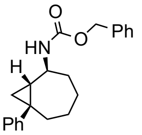

OREPAD

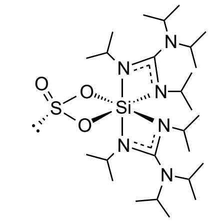

ORIFEC

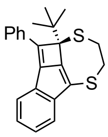

OSEFOJ

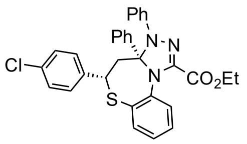

OSUSOL

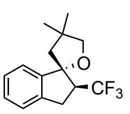

OTEREM

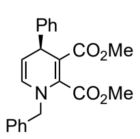

OTOHIP

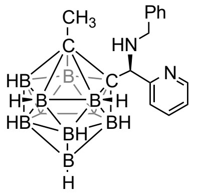

OTOLIT

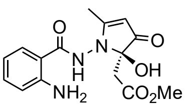

OVAZUI

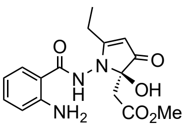

OVEBEY

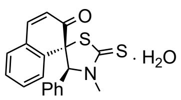

OWOGOY

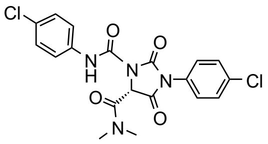

OXAZLB

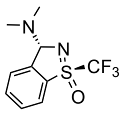

OXECOL

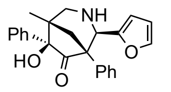

OXUCER

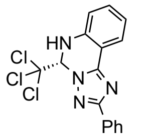

OZUPUW

CSD Code	Space Group	a / Å	b / Å	c / Å	α / °	β / °	γ / °	Publication Year	Reference
PABGAC	$P2_12_12_1$	5.673	9.768	22.126	90	90	90	2010	901
PACBEA	$P2_12_12_1$	6.396	10.321	17.513	90	90	90	1992	902
PACTIZ	$P4_1$	15.275	15.275	13.74	90	90	90	2015	903
PADVEV	$P2_12_12_1$	11.35	22.223	5.1067	90	90	90	1992	904
PAFBIH	$P2_12_12_1$	11.135	22.122	8.089	90	90	90	1992	905
PAHGOW	$P2_12_12_1$	6.5442	9.7896	21.292	90	90	90	2011	906
PAJPAR	$P2_12_12_1$	17.23	17.65	5.721	90	90	90	1992	907
PAJRIE	$C2$	25.305	5.8855	11.941	90	110.07	90	2017	908
PAKMIA	$P2_12_12_1$	6.1724	17.084	18.357	90	90	90	2012	909
PANKUL	$P2_12_12_1$	4.9836	7.6562	25.698	90	90	90	2005	910
PAPCAL	$P2_12_12_1$	7.4218	8.7401	17.287	90	90	90	2005	911
PAPWOS	$P2_1$	7.188	6.323	16.482	90	91.21	90	1992	912
PATFUO	$P2_1$	6.0965	7.8294	17.927	90	96.951	90	2017	913
PATVAK	$P2_1$	9.1845	6.2326	15.953	90	104.976	90	2017	914
PAVZOD	$P2_12_12_1$	5.377	12.598	15.601	90	90	90	2012	915
PAWFOK	$P2_12_12_1$	5.927	11.241	14.9	90	90	90	2012	916
PAWNUZ	$P2_12_12_1$	7.1051	12.329	21.593	90	90	90	2017	917
PAWSIP	$P2_12_12_1$	7.188	12.602	14.559	90	90	90	1997	918
PAXRIR	$P2_12_12_1$	11.348	14.055	15.954	90	90	90	2012	919,920
PAXVIV	$P2_12_12_1$	8.855	12.827	22.432	90	90	90	2012	921
PAZSER	$P2_12_12_1$	7.866	12.253	13.298	90	90	90	2017	922
PEFGUF	$P2_12_12_1$	9.3811	11.214	15.274	90	90	90	2017	923
PEGYOR	$P2_12_12_1$	6.0048	15.757	15.819	90	90	90	2013	924
PELLOJ	$P2_1$	7.1792	10.475	7.4867	90	115.163	90	2009	925
PEMGEW	$P2_12_12_1$	5.3916	9.4754	19.052	90	90	90	2017	926
PEMWIO	$P2_1$	7.9222	11.653	10.953	90	91.454	90	2006	927
PEMWOU	$P1$	8.0265	10.297	10.322	62.490	70.797	81.071	2006	927
PEMWUA	$P2_12_12_1$	6.5480	8.4752	24.468	90	90	90	2006	927
PEPPEG	$P2_1$	6.0006	8.0097	14.479	90	90.68	90	2006	928
PEPXOZ	$P2_12_12_1$	8.84	11.216	15.684	90	90	90	2013	929
PEQFIA	$P2_1$	8.229	5.155	13.389	90	98.68	90	1997	930
PERWOZ	$P2_12_12_1$	5.5779	8.1397	38.894	90	90	90	2006	931
PERYER	$P2_12_12_1$	7.2168	13.155	14.900	90	90	90	2006	932
PESWAM	$P2_1$	5.569	9.512	13.372	90	95.05	90	2006	933
PEVNIN	$P2_12_12_1$	8.661	12.132	14.011	90	90	90	1993	934
PICFAL	$P2_12_12_1$	10.966	11.143	17.306	90	90	90	2018	935
PIFLIC	$P2_12_12_1$	9.8569	11.593	21.895	90	90	90	2018	936
PIHPEE	$P2_12_12_1$	9.1557	11.86	21.294	90	90	90	2018	937
PIJSAF	$P2_12_12_1$	9.4213	9.6086	17.135	90	90	90	2018	938
PIKRAC	$P2_1$	11.124	6.286	11.856	90	103.84	90	1993	939
PIMXIU	$P2_1$	6.0044	10.653	10.296	90	94.784	90	2013	940
PIMYAN	$P2_1$	6.3415	16.423	6.6602	90	98.19	90	2013	940
PIQHAA	$P2_12_12_1$	6.6387	10.102	15.661	90	90	90	2013	941
PIQQUE	$P2_1$	6.471	22.927	7.36	90	115.977	90	2018	942
PIRPOV	$P3_1$	17.274	17.274	6.166	90	90	120	1994	943
PIWVIC	$P2_12_12_1$	10.331	10.646	14.088	90	90	90	2014	944
PIYZEE	$P2_1$	5.645	7.855	18.421	90	95.363	90	2014	945
PODJIC	$P2_12_12_1$	9.2887	11.181	18.055	90	90	90	2008	946
POFLII	$P2_12_12_1$	7.9579	12.902	14.844	90	90	90	2019	947
POHHUR	$P2_12_12_1$	8.6589	10.179	15.977	90	90	90	2014	948
POKJEF	$P2_12_12_1$	6.7333	8.0319	26.078	90	90	90	1994	949
POLJEF	$P2_12_12_1$	10.174	11.981	14.184	90	90	90	1994	950

CSD Code	Space Group	a / Å	b / Å	c / Å	α / °	β / °	γ / °	Publication Year	Reference
POLJOP	$P2_1$	8.691	8.451	10.468	90	111.97	90	1994	[951]
PONTAO01	$C222_1$	9.7978	26.393	19.907	90	90	90	2017	[952]
POPDEE	$P2_12_12_1$	17.409	4.9166	19.27	90	90	90	2008	[953]
POSDAF	$P2_1$	7.7857	9.2744	13.665	90	93.49	90	2012	[954]
POSLEQ	$P2_12_12_1$	5.5998	12.645	18.051	90	90	90	2014	[955]
POXVUV	$P2_12_12_1$	7.9707	8.873	16.273	90	90	90	2015	[956]
POZHOC	$P2_12_12_1$	7.6139	12.742	24.148	90	90	90	2009	[957]
PSIHPD	$P2_12_12_1$	9.509	17.394	17.545	90	90	90	1979	[958]
PTSANO	$P2_12_12_1$	7.652	8.311	25.486	90	90	90	1980	[959]
PULNIV	$P2_12_12_1$	9.398	9.776	24.059	90	90	90	2015	[960]
PUQPIA	$P2_1$	8.727	9.162	10.205	90	111.04	90	1998	[961]
PURJUJ	$P2_12_12_1$	5.537	8.819	25.832	90	90	90	2015	[962]
PUTFUF	$P2_12_12_1$	6.065	7.674	21.728	90	90	90	1998	[963]
PUTHUH	$P2_12_12_1$	13.067	13.094	8.299	90	90	90	1998	[964]
PUZVAJ	$P2_1$	11.623	5.2394	14.288	90	100.456	90	2016	[965]

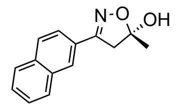
PABGAC

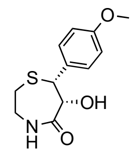
PACBEA

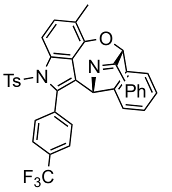
PACTIZ

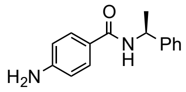
PADVEV

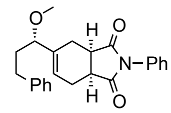
PAFBIH

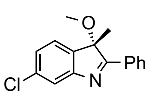
PAHGOW

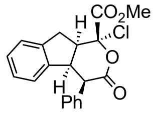
PAJPAR

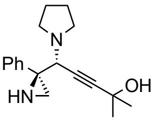
PAJRIE

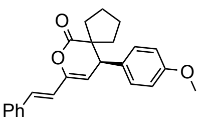
PAKMIA

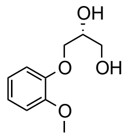
PANKUL

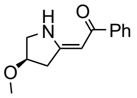
PAPCAL

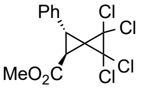
PAPWOS

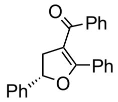
PATFUO

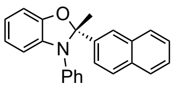
PATVAK

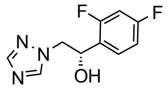
PAVZOD

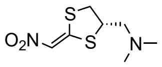
PAWFOK

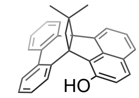
PAWNUZ

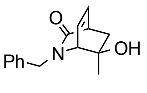
PAWSIP

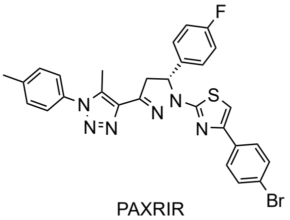
PAXRIR

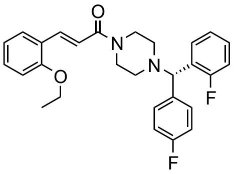
PAXVIV

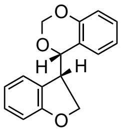
PAZSER

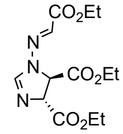
PEFGUF

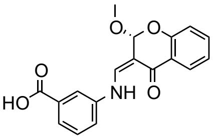
PEGYOR

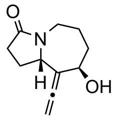
PELLOJ

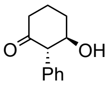
PEMGEW

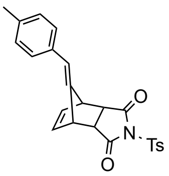
PEMWIO

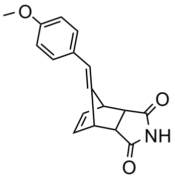
PEMWOU

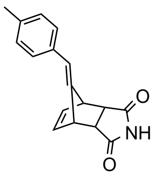
PEMWUA

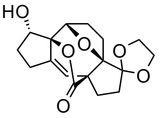
PEPPEG

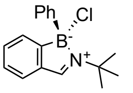
PEPXOZ

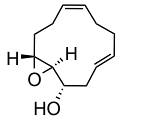
PEQFIA

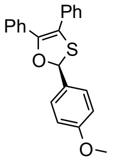
PERWOZ

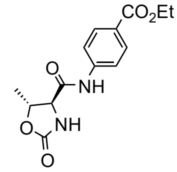
PERYER

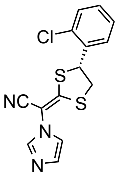
PESWAM

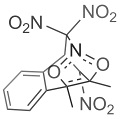
PEVNIN

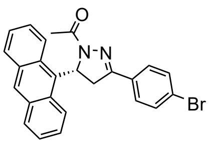
PICFAL

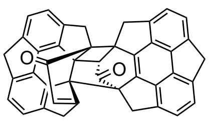
PIFLIC

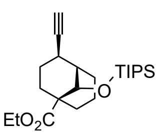
PIHPEE

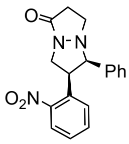
PIJSAF

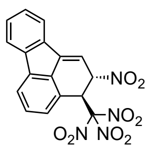

PIKRAC

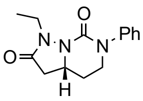

PIMXIU

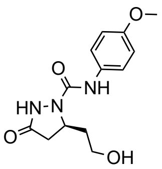

PIMYAN

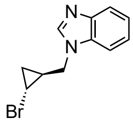

PIQHAA

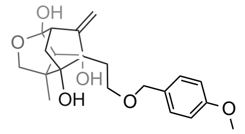

PIQQUE

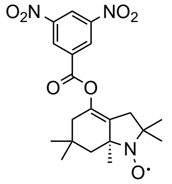

PIRPOV

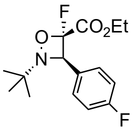

PIWVIC

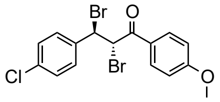

PIYZEE

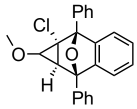

PODJIC

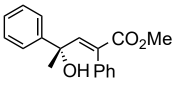

POFLII

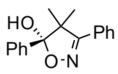

POHHUR

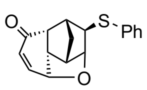

POKJEF

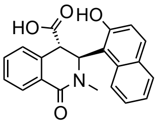

POLJEF

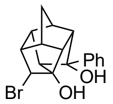

POLJOP

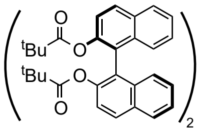

PONTAO01

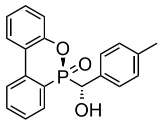

POPDEE

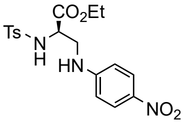

POSDAF

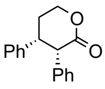

POSLEQ

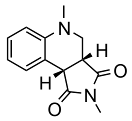

POXVUV

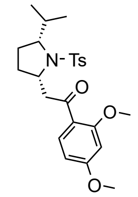

POZHOC

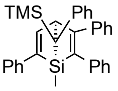

PSIHPD

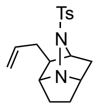

PTSANO

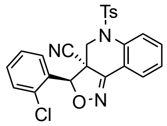

PULNIV

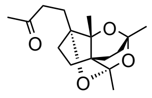

PUQPIA

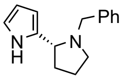

PURJUJ

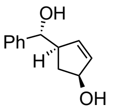

PUTFUF

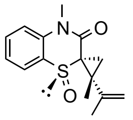

PUTHUH

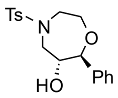

PUZVAJ

CSD Code	Space Group	a / Å	b / Å	c / Å	α / °	β / °	γ / °	Publication Year	Reference
QACXEY	$P2_12_12_1$	9.0387	9.7306	13.171	90	90	90	2002	[966]
QADGOS	$P2_1$	6.5608	15.754	7.9593	90	97.185	90	2003	[967]
QAGRIZ	$P2_12_12_1$	12.179	20.214	7.613	90	90	90	1999	[968]
QAJMEU	$P2_1$	7.548	13.129	11.868	90	105.03	90	2004	[969]
QAJZIL	$P2_12_12_1$	8.66	13.115	23.23	90	90	90	2004	[970]
QAMJOG	$P4_32_12$	10.255	10.255	30.57	90	90	90	2007	[971]
QANGAP	$P2_12_12_1$	9.66	14.819	17.582	90	90	90	2012	[972]
QANSIK	$P2_1$	10.683	9.4028	11.613	90	113.503	90	2017	[973]
QAPROR	$P2_12_12_1$	8.755	16.754	16.818	90	90	90	2017	[974]
QAPZIS	$P2_12_12_1$	4.7416	9.6905	28.978	90	90	90	2012	[975]
QAQWUB	$P2_12_12_1$	10.473	10.904	13.042	90	90	90	2005	[976]
QARFIA	$P2_12_12_1$	5.9944	12.742	20.146	90	90	90	2012	[977]
QATXER	$P1$	6.6729	7.4096	7.9216	69.598	80.621	78.419	2017	[978]
QATXOB	$P2_1$	8.0302	7.8521	10.379	90	93.925	90	2017	[978]
QATZAN	$P2_12_12_1$	14.615	15.929	8.391	90	90	90	2005	[979]
QAVBOH	$P2_12_12_1$	10.796	11.062	17.442	90	90	90	2017	[980]
QAWKUV	$P2_12_12_1$	10.895	12.141	15.792	90	90	90	2005	[981]
QEBFIM	$P2_12_12_1$	7.908	21.361	5.9	90	90	90	2000	[982]
QECYIG	$P2_12_12_1$	8.49	9.358	20.262	90	90	90	2000	[983]
QEFWED	$P2_12_12_1$	6.845	11.2	23.219	90	90	90	1998	[984]
QEHKIX	$P2_1$	6.707	10.355	8.795	90	109.23	90	2001	[985]
QEMTUA	$P2_12_12_1$	5.8489	15.163	18.617	90	90	90	2018	[986]
QEQMIJ	$P2_1$	9.214	10.191	9.606	90	111.68	90	2006	[987]
QERQOU	$P2_12_12_1$	6.3359	17.341	18.407	90	90	90	2006	[988]
QEVSAO	$P2_12_12_1$	8.6681	10.398	11.412	90	90	90	2018	[989]
QEZVAV	$P2_12_12_1$	10.376	10.82	10.899	90	90	90	2018	[990]
QIBCOU	$P2_1$	8.9503	9.6053	9.5069	90	107.63	90	2007	[991]
QIBQEY	$P2_12_12_1$	9.1967	13.528	17.333	90	90	90	2005	[992]
QICDIR	$P2_12_12_1$	8.7385	11.447	15.196	90	90	90	2012	[993]
QIHDUG	$P2_12_12_1$	12.214	13.305	9.083	90	90	90	2001	[994]
QIHSOP	$P2_1$	6.279	9.308	10.219	90	90	90	2001	[995]
QILTIR	$P2_1$	9.836	6.0097	14.856	90	102.362	90	2018	[996]
QILWAL	$P2_12_12_1$	9.0603	10.111	24.204	90	90	90	2013	[997]
QIMRUB	$P2_1$	8.42	5.718	12.43	90	101.626	90	2013	[998]
QIMSAI	$P4_1$	11.53	11.53	10.844	90	90	90	2013	[998]
QIPSUF	$P3_2$	9.277	9.277	34.749	90	90	120	2013	[999]
QIQMEI	$P2_12_12_1$	6.522	7.463	29.342	90	90	90	2001	[1000]
QIQTEP	$P2_12_12_1$	16.09	16.19	8.298	90	90	90	2000	[1001]
QITJEI	$P2_12_12_1$	11.51	11.42	9.9	90	90	90	2000	[1002]
QOCLUR	$P2_12_12_1$	6.8549	11.448	13.137	90	90	90	2014	[1003]
QOCMAY	$P2_12_12_1$	6.2528	10.758	15.927	90	90	90	2014	[1003]
QOCNUR	$P2_1$	9.272	11.433	14.02	90	106.38	90	2001	[1004]
QOGGOK	$P2_12_12_1$	9.4335	12.569	16.477	90	90	90	2013	[1005]
QOHRUB01	$P2_1$	10.768	6.2844	10.869	90	100.21	90	2012	[1006,1007]
QOQLAK	$P2_12_12_1$	9.3034	12.907	20	90	90	90	2009	[1008]
QOQWOJ	$P2_12_12_1$	9.5827	10.296	17.554	90	90	90	2009	[1009]
QOQWUP	$P2_12_12_1$	9.532	10.469	18.008	90	90	90	2009	[1009]
QORBEF	$P2_12_12_1$	5.632	10.187	18.636	90	90	90	2009	[1010]
QOXWOQ	$P2_12_12_1$	6.916	10.499	21.773	90	90	90	2009	[1011]
QUCREN	$P3_2$	10.18	10.18	10.954	90	90	120	2015	[1012]
QUCVUG	$P2_12_12_1$	6.3186	14.436	18.614	90	90	90	2009	[1013]
QUKPOD	$P2_12_12_1$	8.4667	9.3336	12.037	90	90	90	2015	[1014]

CSD Code	Space Group	a / Å	b / Å	c / Å	α / °	β / °	γ / °	Publication Year	Reference
QUMZUT	$P2_1$	8.9285	5.9015	15.595	90	93.391	90	2001	[1015]
QURTIG	$P2_12_12_1$	8.277	9.723	16.5	90	90	90	2001	[1016]
QUTFOA	$P2_12_12_1$	7.4302	11.98	12.979	90	90	90	2001	[1017]
QUXGOH	$P2_12_12_1$	6.1362	8.956	31.549	90	90	90	2015	[1018]
QUZBUK	$P2_1$	8.5299	9.3529	11.881	90	103.128	90	2015	[1019]
QUZFOI	$P2_1$	8.5947	9.7536	11.95	90	101.773	90	2015	[1019]

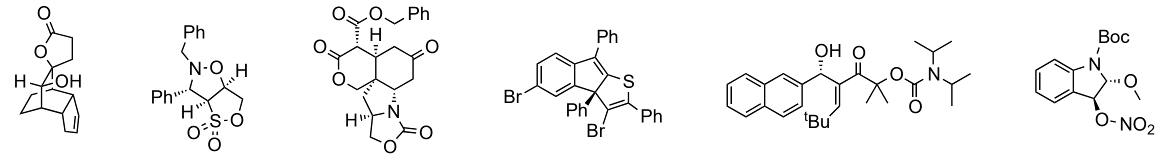

QACXEY QADGOS QAGRIZ QAJMEU QAJZIL QAMJOG

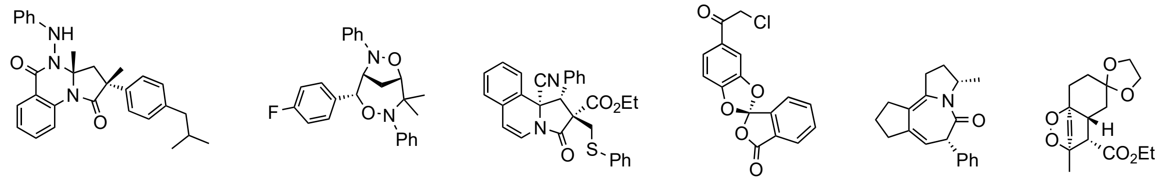

QANGAP QANSIK QAPROR QAPZIS QAQWUB QARFIA

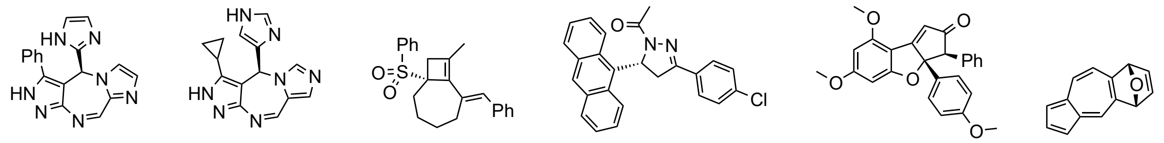

QATXER QATXOB QATZAN QAVBOH QAWKUV QEBFIM

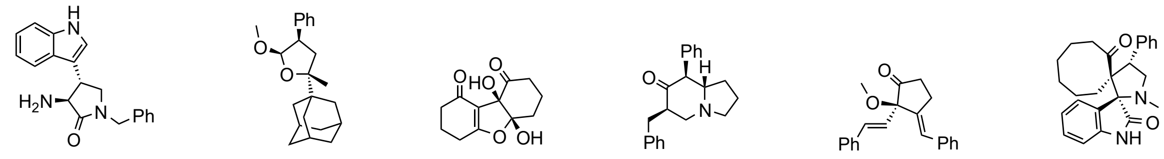

QECYIG QEFWED QEHKIX QEMTUA QEQMIJ QERQOU

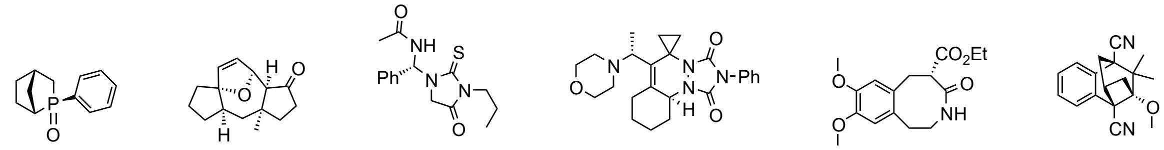

QEVSAO QEZVAV QIBCOU QIBQEY QICDIR QIHDUG

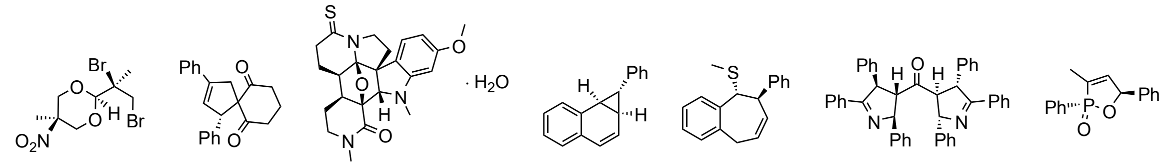

QIHSOP QILTIR QILWAL QIMRUB QIMSAI QIPSUF QIQMEI

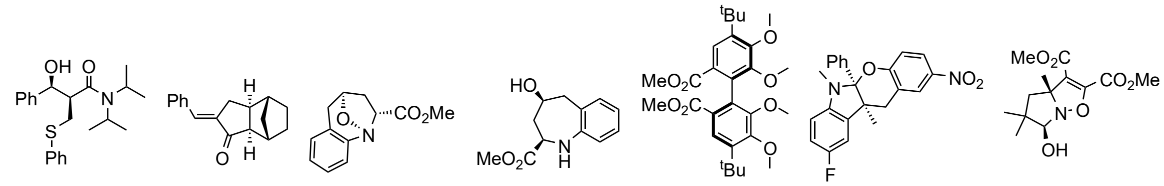

QIQTEP QITJEI QOCLUR QOCMAY QOCNUR QOGGOK QOHRUB01

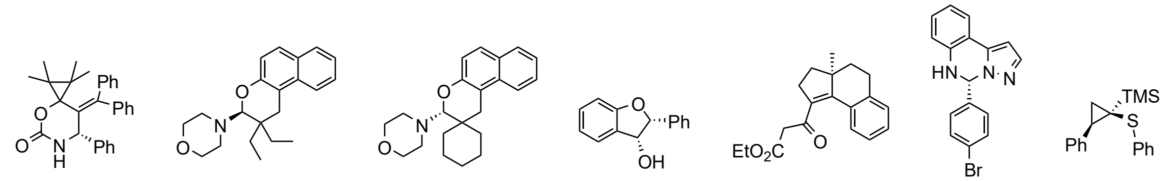

QOQLAK QOQWOJ QOQWUP QORBEF QOXWOQ QUCREN QUCVUG

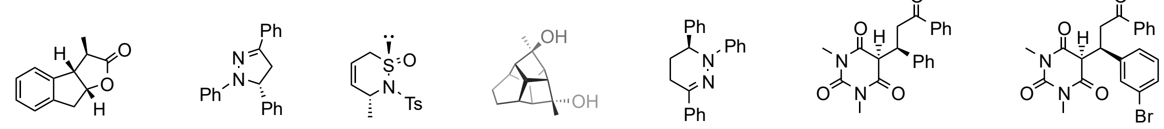

QUKPOD QUMZUT QURTIG QUTFOA QUXGOH QUZBUK QUZFOI

CSD Code	Space Group	a / Å	b / Å	c / Å	α / °	β / °	γ / °	Publication Year	Reference
RABZUP	$P2_12_12_1$	8.447	11.097	14.108	90	90	90	1995	[1020]
RACBIG	$P2_12_12_1$	8.457	12.089	15.275	90	90	90	1994	[1021]
RAFPUJ	$P2_1$	10.457	10.427	11.982	90	108.89	90	1997	[1022]
RAJZEK	$P2_12_12_1$	5.8714	13.929	22.747	90	90	90	2017	[1023]
RAKWEH	$P2_12_12_1$	5.8788	8.1919	25.521	90	90	90	2011	[1024]
RARMII	$P2_12_12_1$	6.9445	11.372	17.438	90	90	90	2012	[1025]
RAWNOU	$P2_12_12_1$	8.5429	11.857	18.454	90	90	90	2012	[1026]
RAXPOY	$P2_12_12_1$	10.761	12.645	15.125	90	90	90	2017	[1027]
RAXQUD	$P2_12_12_1$	6.424	11.67	18.045	90	90	90	2005	[1028]
RAYZAS	$P2_12_12_1$	10.328	13.182	16.469	90	90	90	1996	[1029]
RAZDEB	$P2_1$	9.281	11.154	10.465	90	94.62	90	1996	[1030]
REBNOD	$P2_12_12_1$	5.294	10.197	19.812	90	90	90	2012	[1031]
REBTUO	$P2_1$	8.207	4.857	12.615	90	93.1	90	2006	[1032]
REDCEJ	$P2_12_12_1$	11.681	19.082	6.506	90	90	90	2006	[1033]
REFLIX	$P2_12_12_1$	14.027	16.421	10.704	90	90	90	1996	[1034]
REMQAE	$P2_12_12_1$	5.608	8.703	20.091	90	90	90	2017	[1035]
REMVUD	$P2_12_12_1$	5.5974	19.005	17.254	90	90	90	2017	[1036]
REQYER	$P2_12_12_1$	6.937	7.996	12.91	90	90	90	1997	[1037]
RERLIL	$P2_12_12_1$	9.829	9.8991	16.372	90	90	90	2013	[1038]
RERNUZ	$P2_1$	10.133	8.776	16.458	90	92.6	90	2013	[1039]
RESGII	$P2_1$	10.409	6.2017	15.424	90	92.398	90	2018	[1040]
RESRIS	$P2_1$	12.463	8.235	12.658	90	114.56	90	2013	[1041]
REVGEF	$P2_12_12_1$	24.29	10.813	8.2719	90	90	90	2006	[1042]
REYZEA	$P2_12_12_1$	6.037	20.049	14.867	90	90	90	1997	[1043]
RICCAI	$P2_12_12_1$	8.3272	10.025	19.173	90	90	90	2007	[1044]
RICRIE	$P1$	6.9014	7.6944	8.3578	65	82	65	2001	[1045]
RICXIM	$P2_12_12_1$	6.371	9.26	22.629	90	90	90	2013	[1046]
RIFJEY	$P2_12_12_1$	9.3423	13.223	14.653	90	90	90	2018	[1047]
RIJGAS	$P2_12_12_1$	5.45	14.58	14.802	90	90	90	1996	[1048]
RILBET	$P2_12_12_1$	10.472	19.127	8.594	90	90	90	1997	[1049]
RILXIV	$P2_12_12_1$	5.7199	10.088	22.966	90	90	90	2013	[1050]
RIMBIB	$P2_12_12_1$	10.086	15.32	18.346	90	90	90	2017	[1051]
RINLAB	$P2_12_12_1$	7.664	10.354	11.838	90	90	90	1997	[1052]
RIPBUN	$P2_12_12_1$	4.787	9.461	40.809	90	90	90	1996	[1053]
RIPSOB	$P2_1$	9.9844	6.1453	15.226	90	104.406	90	2018	[1054]
RIRJAD	$P2_1$	10.31	12.524	10.978	90	114.61	90	1996	[1055]
RIWBIK	$P2_12_12_1$	9.176	11.499	14.733	90	90	90	2014	[1056]
RIWDOS	$P4_32_12$	10.342	10.342	25.123	90	90	90	2014	[1056]
RIXGEN	$P2_1$	10.175	11.032	11.672	90	102.926	90	2018	[1057]
RIZCOT	$P2_12_12_1$	8.863	9.759	20.071	90	90	90	2008	[1058]
ROBTEJ	$P2_12_12_1$	5.6284	15.592	18.829	90	90	90	2014	[1059]
ROCXIT	$P2_1$	9.4601	6.1264	16.668	90	90.894	90	2019	[1060]
ROGMUY	$P2_12_12_1$	9.468	9.704	17.245	90	90	90	2019	[1061]
ROGVOZ	$P2_12_12_1$	7.4686	16.501	18.259	90	90	90	2008	[1062]
ROJPOW	$P2_1$	9.43	9.3303	9.6272	90	90.282	90	2008	[1063]
ROKWOF	$P2_12_12_1$	4.931	16.1	19.376	90	90	90	2014	[1064]
ROMFAD	$P4_3$	10.655	10.655	15.113	90	90	90	2019	[1065]
ROMFOR	$P2_12_12_1$	5.6073	16.731	16.921	90	90	90	2019	[1065]
ROPNAL	$P2_12_12_1$	6.4973	12.871	15.643	90	90	90	2001	[1066]
ROSBAE	$P2_12_12_1$	6.1169	7.6024	31.316	90	90	90	2014	[1067]
ROSMUK	$P2_12_12_1$	5.9266	7.4195	29.122	90	90	90	2019	[1068]
ROTLOE	$P2_12_12_1$	6.1557	9.8405	16.063	90	90	90	2019	[1069]

CSD Code	Space Group	a / Å	b / Å	c / Å	α / °	β / °	γ / °	Publication Year	Reference
ROTNAS	$P2_12_12_1$	8.6617	9.6496	22.599	90	90	90	2019	[1069]
ROVBIQ	$P2_12_12_1$	6.2462	7.5477	31.123	90	90	90	2019	[1070]
ROVMAS	$P2_1$	6.462	21.405	11.722	90	105.001	90	2014	[1071]
ROYMUP	$P2_1$	6.2588	14.004	8.5176	90	101.959	90	2015	[1072]
RUDQIS	$P2_12_12_1$	7.2551	9.233	21.918	90	90	90	2014	[1073]
RUJMAL	$P2_12_12_1$	6.717	10.037	20.429	90	90	90	2009	[1074]
RUJSAR	$P2_12_12_1$	5.9793	11.737	22.565	90	90	90	2009	[1075]
RUJVOH	$P2_12_12_1$	9.4477	14.272	16.526	90	90	90	1997	[1076]
RUPFEO	$P3_2$	12.133	12.133	5.804	90	90	120	2009	[1077]
RUQFIS	$P2_12_12_1$	7.789	29.326	5.313	90	90	90	1997	[1078]
RUTYIO	$P2_1$	9.4793	10.384	11.594	90	91.408	90	1997	[1079]
RUWHIB	$P2_12_12_1$	6.434	12.378	23.51	90	90	90	2010	[1080]
RUWLUQ	$P2_12_12_1$	9.818	11.191	11.79	90	90	90	1997	[1081]
RUXJUR	$P2_12_12_1$	10.042	17.602	18.655	90	90	90	2015	[1082]

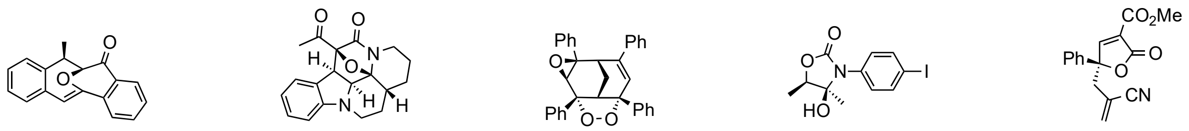

RABZUP RACBIG RAFPUJ RAKWEH RARMII

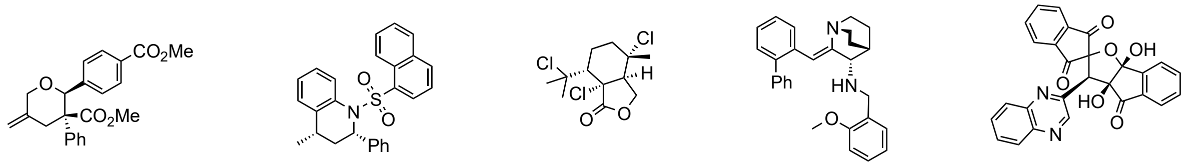

RAWNOU RAXPOY RAXQUD RAYZAS RAZDEB

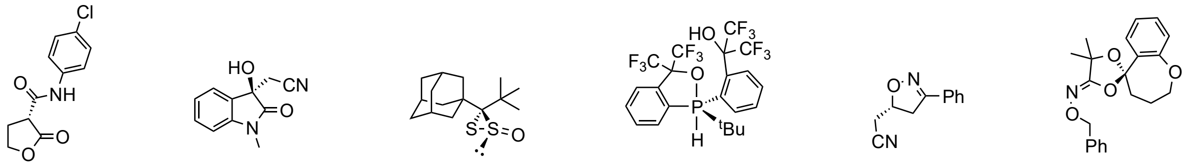

REBNOD REBTUO REDCEJ REFLIX REMQAE REMVUD

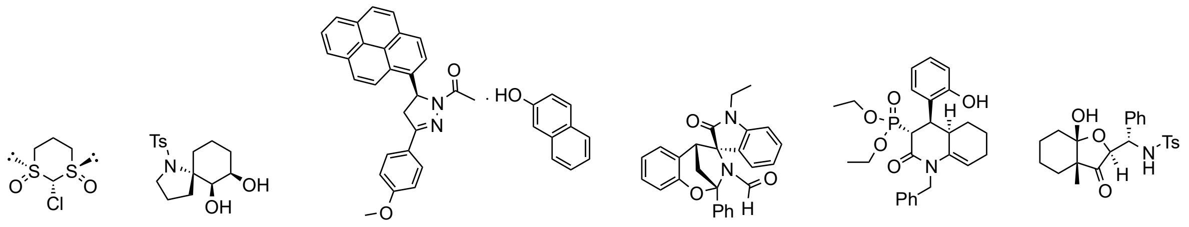

REQYER RERLIL RERNUZ RESGII RESRIS REVGEF

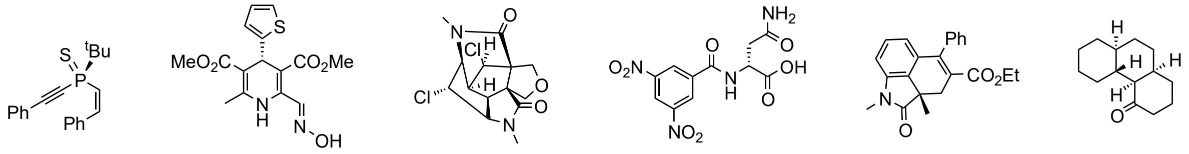

REYZEA RICCAI RICRIE RICXIM RIFJEY RIJGAS

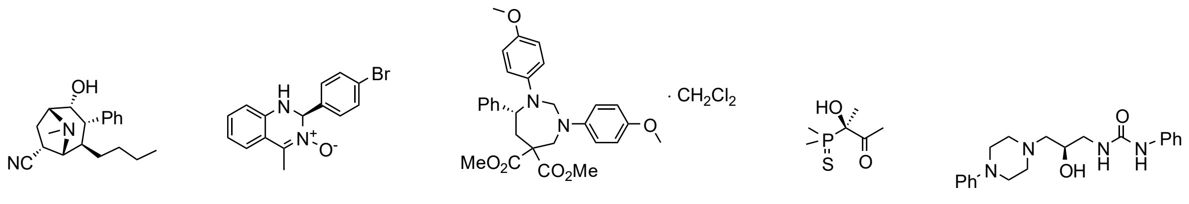

RILBET RILXIV RIMBIB RINLAB RIPBUN

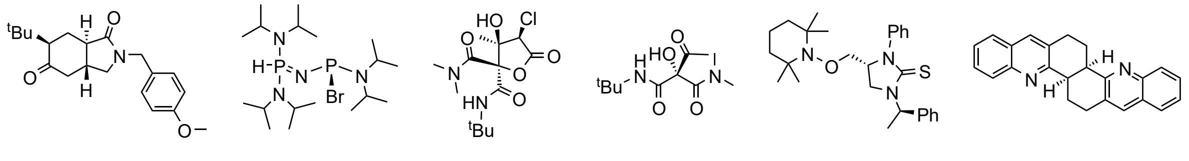

RIPSOB RIRJAD RIWBIK RIWDOS RIXGEN RIZCOT

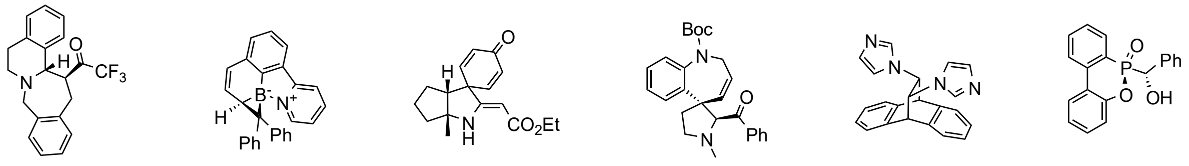

ROBTEJ ROCXIT ROGMUY ROGVOZ ROJPOW ROKWOF

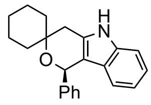

ROMFAD

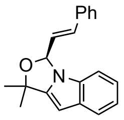

ROMFOR

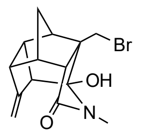

ROPNAL

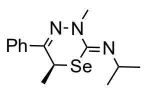

ROSBAE

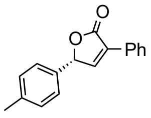

ROSMUK

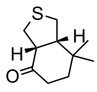

ROTLOE

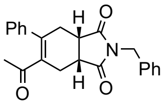

ROTNAS

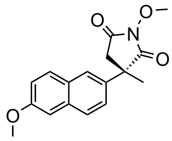

ROVBIQ

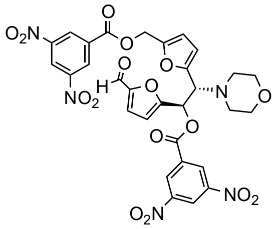

ROVMAS

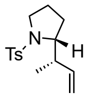

ROYMUP

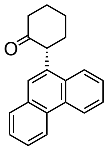

RUDQIS

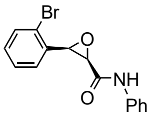

RUJMAL

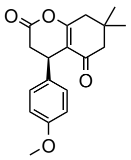

RUJSAR

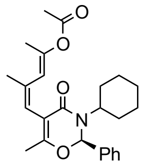

RUJVOH

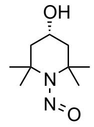

RUPFEO

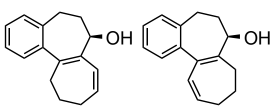

RUQFIS

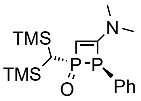

RUTYIO

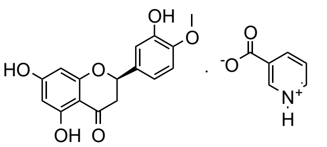

RUWHIB

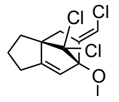

RUWLUQ

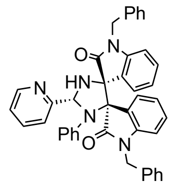

RUXJUR

CSD Code	Space Group	a / Å	b / Å	c / Å	α / °	β / °	γ / °	Publication Year	Reference
SAGQIC	$P2_1$	7.122	5.469	12.981	90	98.13	90	2012	1083
SAKCEP	$P2_1$	7.1675	13.137	10.007	90	101.075	90	2017	1084
SALMOI	$P2_12_12_1$	5.1862	12.839	17.14	90	90	90	2005	1085
SALROM	$P2_12_12_1$	7.74	12.186	23.86	90	90	90	1989	1086
SALWOR	$P2_12_12_1$	5.178	11.763	19.249	90	90	90	1989	1087
SANVEJ	$P4_3$	8.2117	8.2117	27.44	90	90	90	2005	1088
SAPKAY	$P2_1$	11.256	6.3122	18.973	90	98.717	90	2013	1089
SAPMAY	$P2_12_12_1$	7.82	7.911	28.766	90	90	90	2005	1090
SAPMAZ	$C2$	12.332	11.416	12.947	90	96.198	90	2011	1091
SAPQUX	$P2_12_12_1$	9.1625	9.861	13.968	90	90	90	2012	1092
SAPSEI	$P2_12_12_1$	11.139	19.073	7.848	90	90	90	2004	1093
SAPTUY	$P2_1$	7.271	7.081	18.33	90	95.83	90	1989	1094
SASBIZ	$P2_12_12_1$	5.8929	11.533	29.228	90	90	90	2012	1095
SASDIZ	$P2_12_12_1$	12.467	14.924	22.58	90	90	90	1989	1096
SASHAX	$P2_1$	6.5119	7.3096	16.603	90	93.825	90	2011	1097
SATZOC	$P2_1$	7.225	11.735	7.77	90	112.07	90	1989	1098
SAVCOH	$P2_1$	11.086	8.4961	6.8763	90	91.118	90	1989	1099
SAVHIJ	$P2_12_12_1$	9.0757	10.462	26.245	90	90	90	2017	1100
SAYTOB	$P2_12_12_1$	9.469	9.652	13.949	90	90	90	1988	1101
SECNET	$P2_1$	5.603	7.606	13.964	90	99.88	90	1989	1102
SECYAA	$P2_12_12_1$	9.702	16.234	16.455	90	90	90	1989	1103
SEDMAR	$P2_12_12_1$	5.701	12.485	26.736	90	90	90	2012	1104
SEKLEZ	$P2_12_12_1$	10.69	10.96	10.507	90	90	90	1990	1105
SEKLID	$P2_1$	11.084	13.524	6.304	90	104.8	90	1990	1105
SELGUN	$P2_1$	7.717	10.035	9.992	90	102.451	90	2012	1106
SELZIU	$P2_1$	9.6233	5.3529	13.578	90	91.032	90	2012	1107
SELZOA	$P2_1$	5.5018	10.356	13.085	90	91.64	90	2012	1107
SEMJIE	$P2_12_12_1$	8.1328	9.4467	20.376	90	90	90	2006	1108
SEMZAO	$P2_12_12_1$	9.5716	11.688	18.183	90	90	90	2017	1109
SEPPIP	$P2_12_12_1$	6.5617	7.0318	24.2	90	90	90	2017	1110
SEPXES	$P2_12_12_1$	6.0202	9.5695	28.79	90	90	90	2013	1111
SESSIT	$P2_1$	8.1504	8.4608	9.6959	90	98.036	90	2006	1112
SETREO	$P2_12_12_1$	8.547	9.788	28.068	90	90	90	1990	1113
SICQAY	$P2_12_12_1$	8.392	14.05	19.993	90	90	90	2013	1114
SIGJIB	$P2_12_12_1$	10.385	15.256	8.727	90	90	90	1990	1115
SIGQAD	$P4_1$	8.1375	8.1375	22.67	90	90	90	2018	1116
SIGTEK	$P2_1$	10.729	5.8516	12.815	90	114.59	90	2019	1117
SIJSUC	$P2_12_12_1$	5.1418	8.3699	25.903	90	90	90	2018	1118
SIKHEZ	$P2_12_12_1$	6.425	9.418	19.485	90	90	90	1990	1119
SILJEC	$P2_12_12_1$	7.114	12.251	12.752	90	90	90	1990	1120
SINWOB	$P2_12_12_1$	8.841	10.988	11.912	90	90	90	1986	1121
SIPCEZ	$P2_1$	6.5089	14.972	11.088	90	105.395	90	1997	1122
SIQFIK	$P2_12_12_1$	7.7693	12.582	15.723	90	90	90	2018	1123
SIQXAU	$P2_12_12_1$	6.4112	8.4234	27.491	90	90	90	2018	1124
SIRTIZ	$P2_1$	8.717	10.391	11.596	90	101.797	90	2019	1125
SIVHOU	$P2_12_12_1$	16.921	7.699	6.251	90	90	90	1991	1126
SIVMOB	$P2_1$	10.093	6.337	12.831	90	100.192	90	2014	1127
SIXLAP	$P2_12_12_1$	10.126	12.341	19.425	90	90	90	2019	1128
SIZBAF	$P2_12_12_1$	9.253	11.551	12.831	90	90	90	2007	1129
SIZFEM	$P4_1$	10.722	10.722	16.917	90	90	90	1991	1130
SIZMOG	$P2_12_12_1$	6.4521	10.109	21.654	90	90	90	2019	1131
SOCDOD	$P2_12_12_1$	11.798	14.115	11.108	90	90	90	1998	1132

CSD Code	Space Group	a / Å	b / Å	c / Å	α / °	β / °	γ / °	Publication Year	Reference
SOFHAW	$P2_12_12_1$	8.519	12.409	14.772	90	90	90	1991	[1133]
SOJWAQ	$P2_12_12_1$	8.791	9.756	19.194	90	90	90	2008	[1134]
SOJXIZ	$P2_12_12_1$	6.2729	11.291	12.464	90	90	90	2008	[1135]
SOKTUH	$P2_12_12_1$	25.598	12.846	8.732	90	90	90	1991	[1136]
SOLRUJ	$P2_12_12_1$	6.7377	9.7118	29.733	90	90	90	2019	[1137]
SOMDII	$P2_1$	5.709	15.564	9.005	90	95.57	90	2008	[1134]
SOMRAN	$P2_12_12_1$	11.5	14.877	8.102	90	90	90	1991	[1138]
SORTUO	$P2_12_12_1$	9.392	11.447	12.914	90	90	90	1991	[1139]
SOTXAA01	$P2_12_12_1$	12.25	10.969	13.343	90	90	90	1998	[1140]
SOVCIS	$C2$	22.14	6.8047	14.236	90	100.71	90	2019	[1141]
SOVKIZ	$P2_1$	5.717	7.812	12.901	90	93.75	90	2014	[1142]
SOWKIZ	$P2_1$	7.5500	4.9283	15.390	90	94.798	90	2009	[910]
SOWKOF	$P2_1$	13.013	4.8558	16.371	90	109.235	90	2011	[1143]
SUCPYR10	$P2_12_12_1$	7.775	14.974	7.73	90	90	90	1976	[1144]
SUDTOC	$P2_12_12_1$	8.6959	10.222	14.583	90	90	90	2015	[1145]
SUFKEL	$P2_12_12_1$	8.9396	16.447	17.519	90	90	90	2015	[1146]
SUGBIH	$P2_1$	8.0631	6.911	12.33	90	92.691	90	2014	[1147]
SUGVOH	$P2_12_12_1$	8.8242	11.09	19.493	90	90	90	2015	[1148]
SUHTOG	$P1$	8.0316	9.266	9.7678	110.12	113.018	94.668	2014	[1149]
SUJTAU	$P2_1$	10.43	6.0297	12.714	90	108.918	90	2015	[1150]
SUQDEO	$P1$	6.1179	7.734	9.9532	72.868	79.475	81.828	2008	[1151]
SURFUH	$P2_12_12_1$	7.4202	10.764	19.946	90	90	90	2009	[1152]
SURVUY	$P2_12_12_1$	10.673	10.993	12.81	90	90	90	2004	[1153]
SUSCOA	$P2_12_12_1$	10.593	12.629	18.741	90	90	90	2011	[1154]
SUTDIW	$P2_1$	7.077	8.79	14.988	90	100.256	90	2015	[1155]
SUWQUX	$P2_12_12_1$	5.7885	15.144	23.744	90	90	90	2010	[1156]
SUXHIE	$P2_12_12_1$	9.002	13.225	17.293	90	90	90	2016	[1157]
SUZLOO	$P2_12_12_1$	13.304	15.256	7.2256	90	90	90	2001	[1158]
SUZMEG	$P2_12_12_1$	5.9179	13.917	20.405	90	90	90	2010	[1159]

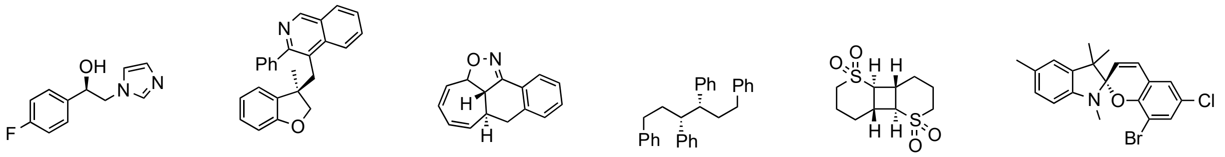

SAGQIC SAKCEP SALMOI SALROM SALWOR SANVEJ

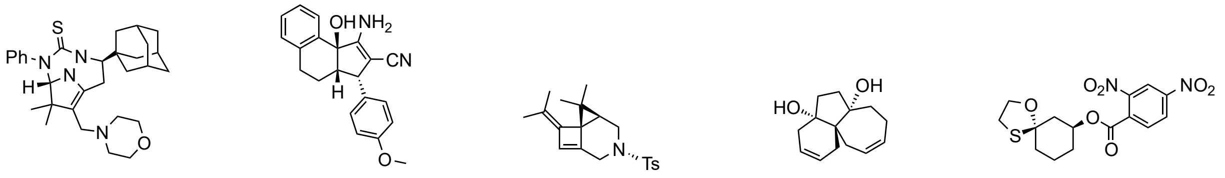

SAPKAY SAPMAY SAPMAZ SAPQUX SAPSEI

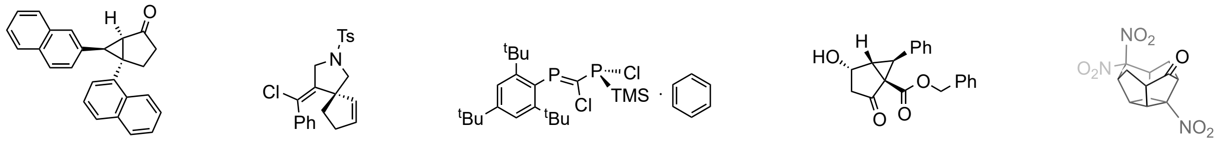

SAPTUY SASBIZ SASDIZ SASHAX SATZOC

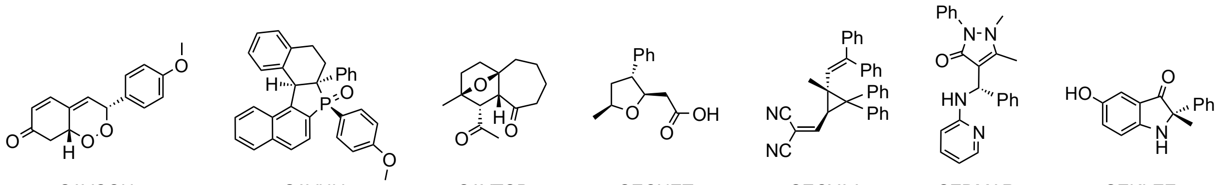

SAVCOH SAVHIJ SAYTOB SECNET SECYAA SEDMAR SEKLEZ

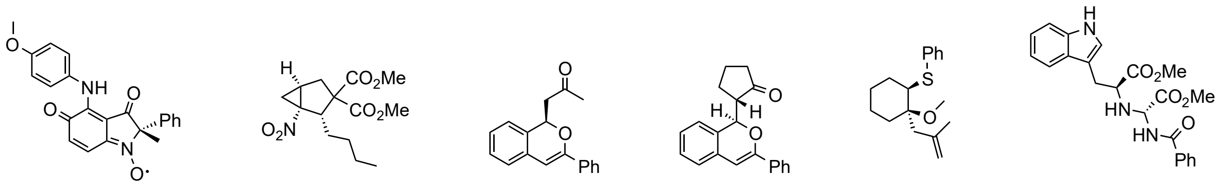

SEKLID SELGUN SELZIU SELZOA SEMJIE SEMZAO

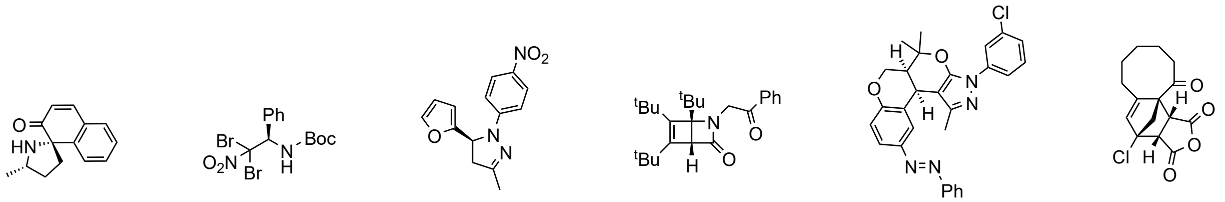

SEPPIP SEPXES SESSIT SETREO SICQAY SIGJIB

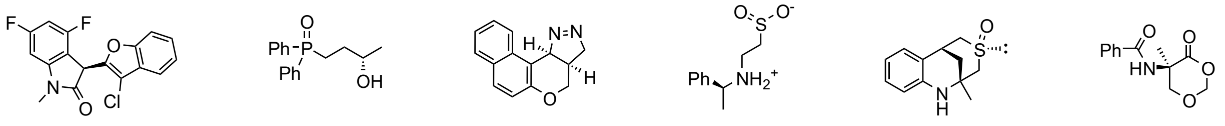

SIGQAD SIGTEK SIJSUC SIKHEZ SILJEC SINWOB

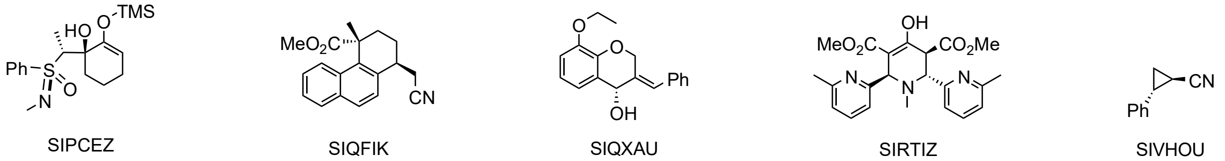

SIPCEZ SIQFIK SIQXAU SIRTIZ SIVHOU

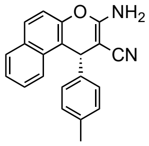

SIVMOB

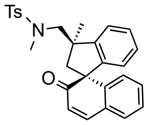

SIXLAP

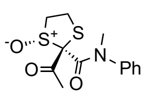

SIZBAF

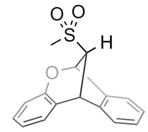

SIZMOG

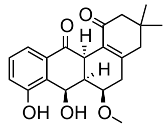

SOCDOD

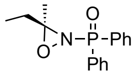

SOFHAW

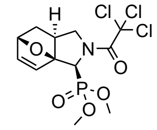

SOJWAQ

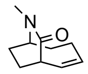

SOJXIZ

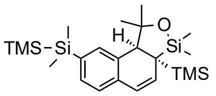

SOKTUH

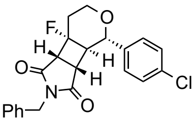

SOLRUJ

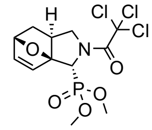

SOMDII

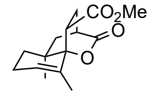

SOMRAN

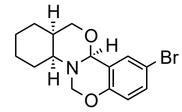

SORTUO

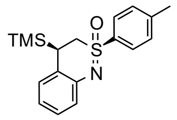

SOTXAA01

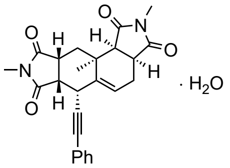

SOVCIS

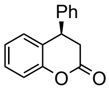

SOVKIZ

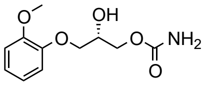

SOWKIZ

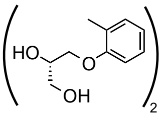

SOWKOF

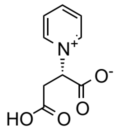

SUCPYR10

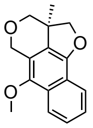

SUDTOC

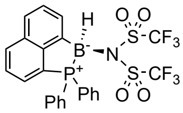

SUFKEL

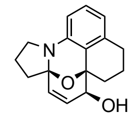

SUGBIH

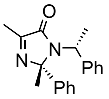

SUGVOH

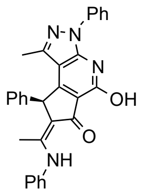

SUHTOG

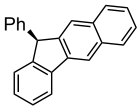

SUJTAU

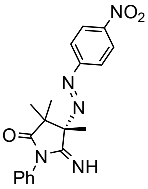

SUQDEO

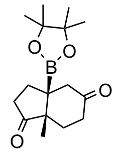

SURFUH

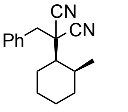

SURVUY

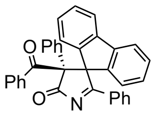

SUSCOA

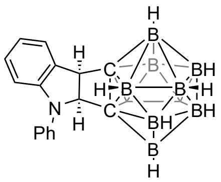

SUTDIW

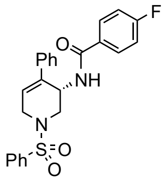

SUWQUX

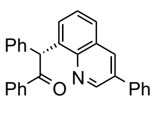

SUXHIE

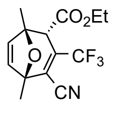

SUZLOO

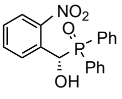

SUZMEG

CSD Code	Space Group	a / Å	b / Å	c / Å	α / °	β / °	γ / °	Publication Year	Reference
TABVEA	$P2_1$	7.306	11.03	10.145	90	94.146	90	2016	[1160]
TADNET	$P2_12_12_1$	10.991	13.005	20.055	90	90	90	2010	[1161]
TADRIA	$P2_12_12_1$	8.8291	9.8819	18.205	90	90	90	2003	[1162]
TAFZOS	$P2_1$	9.0877	15.044	9.2994	90	111.757	90	2016	[1163]
TAGGEP	$P2_12_12_1$	6.0327	14.27	16.473	90	90	90	2010	[1164]
TAGGEQ	$P2_12_12_1$	9.4078	9.4698	26.64	90	90	90	2016	[1165]
TAGGIT	$P2_12_12_1$	4.0331	15.775	22.125	90	90	90	2010	[1164]
TAGGOA	$P2_12_12_1$	5.6186	8.359	37.132	90	90	90	2016	[1165]
TAHCOU	$P2_12_12_1$	13.028	13.021	14.252	90	90	90	1991	[1166]
TAJFES	$P2_1$	9.5259	9.8506	10.346	90	108.316	90	2016	[1167]
TAJLAR	$P2_12_12_1$	9.411	12.194	16.412	90	90	90	1991	[1168]
TAKBUF	$P2_1$	9.471	5.1141	12.778	90	95.046	90	2016	[1169]
TAKFOD	$P2_12_12_1$	6.9218	11.389	25.067	90	90	90	2016	[1170]
TAKGIV	$P2_12_12_1$	10.893	28.072	10.596	90	90	90	1990	[1171]
TAMPII	$P2_1$	13.589	21.416	16.083	90	90.573	90	2011	[1172]
TANVIQ	$P2_12_12_1$	6.0741	14.962	16.218	90	90	90	2017	[1173]
TAQHIE	$P2_1$	11.241	6.823	11.298	90	117.314	90	2012	[1174]
TATJAZ	$P2_12_12_1$	9.442	10.733	18.333	90	90	90	1996	[1175]
TAVBOK	$P2_1$	13.577	7.8653	14.909	90	108.082	90	2017	[1176]
TAZKUB	$P2_12_12_1$	8.2718	10.086	16.593	90	90	90	2006	[1177]
TBCNON	$P2_12_12_1$	11.693	6.4303	10.462	90	90	90	1972	[1178]
TECPAV	$P2_12_12_1$	6.8321	11.623	17.82	90	90	90	2017	[1179]
TEFVAB	$P2_12_12_1$	7.041	7.252	26.363	90	90	90	1996	[1180]
TEFYUZ	$P2_12_12_1$	15.422	16.648	6.804	90	90	90	2006	[504]
TEJPII	$P2_12_12_1$	5.8236	7.982	24.523	90	90	90	2006	[1181]
TENJEE	$P2_12_12_1$	7.3042	10.834	18.159	90	90	90	2017	[1182]
TERBUN	$P2_1$	9.149	10.325	10.308	90	115.68	90	1996	[1183]
TESNEL	$P2_12_12_1$	6.5521	10.563	14.078	90	90	90	2006	[1184]
TESPEN	$P2_12_12_1$	7.5202	7.8968	17.309	90	90	90	2006	[1184]
TEVBAZ	$P2_1$	7.5389	9.8159	9.8152	90	95.303	90	2013	[1185]
TEVJIN	$P2_12_12_1$	8.2271	13.563	13.765	90	90	90	1995	[1186]
TEWFOQ	$P2_12_12_1$	10.291	16.521	8.631	90	90	90	1996	[1187]
TEZDOS	$P2_12_12_1$	11.129	13.919	16.987	90	90	90	2007	[1188]
TEZKOA	$P2_12_12_1$	8.2442	13.672	20.987	90	90	90	2013	[1189]
THZOLB	$P2_12_12_1$	13.695	10.581	11.261	90	90	90	1981	[1190]
TIBGOA	$P2_12_12_1$	9.592	14.983	16.214	90	90	90	2001	[1191]
TIBGUG	$P2_1$	6.031	13.94	8.556	90	100.354	90	1996	[1192,1193]
TICHOC	$P2_12_12_1$	15.962	5.218	9.613	90	90	90	1996	[1194]
TIDLIC	$P2_12_12_1$	9.0712	11.824	31.201	90	90	90	2007	[1195]
TIDTAC	$P2_12_12_1$	6.592	10.908	15.132	90	90	90	2007	[1196]
TIDVAE	$P6_1$	23.622	23.622	4.7492	90	90	120	2007	[1197]
TIGVOX	$P2_12_12_1$	9.6235	13.355	16.794	90	90	90	2018	[1198]
TIHJUR	$P2_1$	12.813	6.246	15.497	90	110.299	90	2012	[1199]
TIHKOM	$P2_1$	12.578	6.194	14.866	90	108.503	90	2012	[1199]
TIJWIS	$P2_1$	8.328	8.393	10.212	90	106.696	90	2001	[1200,1201]
TIMRUE	$P2_12_12_1$	7.8181	12.613	21.222	90	90	90	2013	[1202]
TIMTUE	$P2_12_12_1$	5.675	17.185	18.397	90	90	90	1996	[1203]
TITVUN	$P2_12_12_1$	7.095	10.208	22.222	90	90	90	1995	[1204]
TIVHUC	$P2_1$	10.648	8.5764	12.78	90	113.614	90	2007	[1205]
TIXPOG	$P2_1$	8.4041	7.7989	11.97	90	103.336	90	2008	[1206]
TIYSIF	$P2_12_12_1$	9.519	9.886	25.98	90	90	90	2013	[1207]
TIZGAN	$P2_12_12_1$	10.104	10.416	12.689	90	90	90	2019	[1208]

CSD Code	Space Group	a / Å	b / Å	c / Å	α / °	β / °	γ / °	Publication Year	Reference
TIZMAT	$P2_1$	10.424	7.8757	12.046	90	103.775	90	2019	[1209]
TMPHTB	$P3_1$	15.756	15.756	5.596	90	90	120	1980	[1210]
TOCHUR	$P2_12_12_1$	6.8989	11.941	18.966	90	90	90	2019	[1211]
TODVEP	$P2_12_12_1$	6.5803	6.6436	32.419	90	90	90	2014	[1212]
TOHJOP	$P2_1$	8.137	12.363	10.537	90	98.77	90	1997	[1213]
TOHMIP	$P2_12_12_1$	8.0548	12.219	16.53	90	90	90	2019	[1214]
TOJBID	$P2_12_12_1$	13.156	10.255	9.631	90	90	90	1996	[1215]
TOPMUH	$P2_1$	8.9537	11.937	11.689	90	100.554	90	2008	[1216]
TOPQOF	$P2_12_12_1$	7.221	10.099	20.92	90	90	90	2008	[1217]
TOQCUA	$P2_1$	7.8987	6.2421	14.872	90	103.53	90	2019	[1218]
TOSTOK	$P2_12_12$	15.61	18.634	7.5672	90	90	90	1996	[1219]
TOVMOG	$P2_12_12_1$	7.916	13.044	18.443	90	90	90	1996	[1220]
TOVNUP	$P2_12_12_1$	12.855	11.294	8.2943	90	90	90	2015	[1221]
TOVZOV	$P2_12_12_1$	7.985	10.282	12.55	90	90	90	2015	[1221]
TOZBIV	$P3_2$	17.867	17.867	5.329	90	90	120	2015	[1222]
TOZYOY	$P2_12_12_1$	6.7599	8.3411	14.101	90	90	90	2015	[1223]
TUHLIR	$P2_12_12_1$	9.169	11.977	17.595	90	90	90	1996	[1224]
TULPUN	$P2_12_12_1$	8.1544	9.1587	16.57	90	90	90	2015	[1225]
TUMJIW	$P2_12_12_1$	8.6617	10.028	13.347	90	90	90	2015	[1226]
TUNCEM	$P2_12_12_1$	11.041	13.009	16.225	90	90	90	2015	[1227]
TUNMOF	$C2$	18.177	7.9808	16.035	90	92.703	90	2010	[1228]
TUNRIE	$P2_12_12_1$	10.582	11.212	15.457	90	90	90	2010	[1229,1230]
TUPMAT	$P2_12_12_1$	6.0171	15.312	18.149	90	90	90	2010	[1231]
TUQBAJ	$P2_12_12_1$	7.8893	11.77	17.439	90	90	90	2010	[1232]
TUSROP	$P2_1$	8.5135	21.504	9.8635	90	110.542	90	2010	[1233]
TUSVEK	$P2_12_12_1$	7.6242	11.63	18.443	90	90	90	2015	[1234]
TUWFAT	$P2_12_12_1$	9.0996	11.215	23.974	90	90	90	2010	[1235]

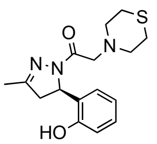
TABVEA

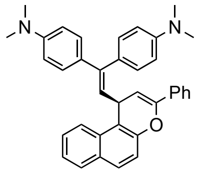
TADNET

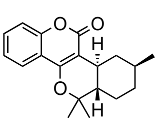
TADRIA

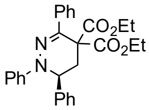
TAFZOS

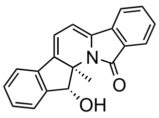
TAGGEP

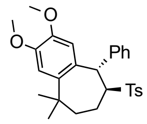
TAGGEQ

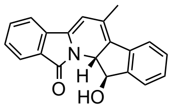
TAGGIT

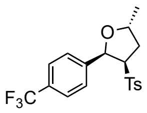
TAGGOA

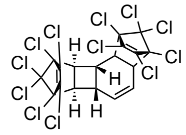
TAHCOU

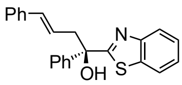
TAJFES

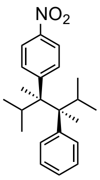
TAJLAR

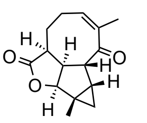
TAKBUF

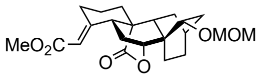
TAKFOD

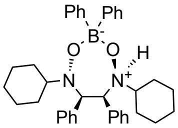
TAKGIV

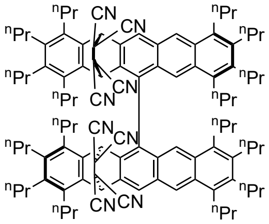
TAMPII

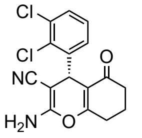
TANVIQ

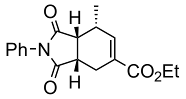
TAQHIE

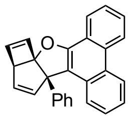
TATJAZ

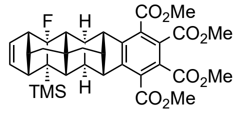
TAVBOK

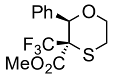
TAZKUB

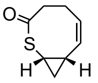
TBCNON

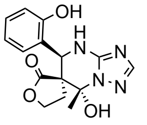
TECPAV

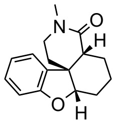
TEFVAB

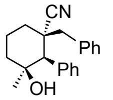
TEFYUZ

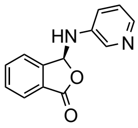
TEJPII

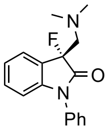
TENJEE

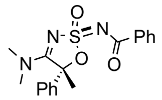
TERBUN

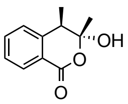
TESNEL

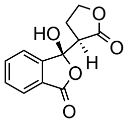
TESPEN

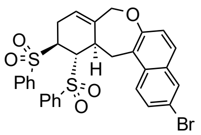
TETYIB

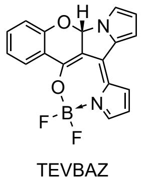
TEVBAZ

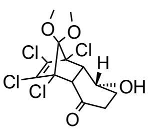
TEVJIN

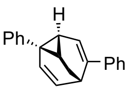
TEWFOQ

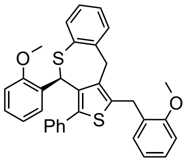
TEZDOS

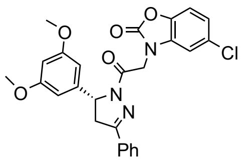
TEZKOA

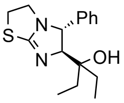
THZOLB

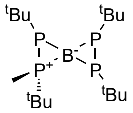
TIBGOA

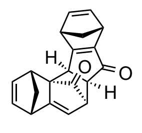
TIBGUG

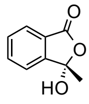
TICHOC

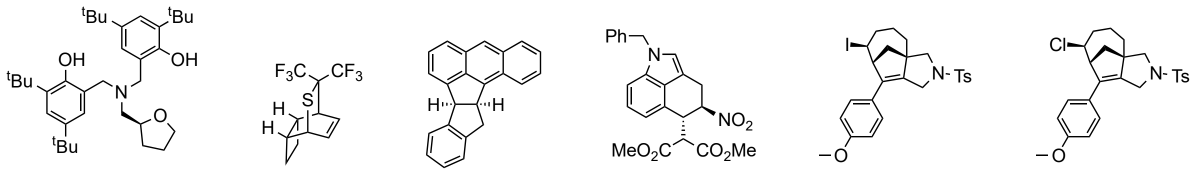

TIDLIC TIDTAC TIDVAE TIGVOX TIHJUR TIHKOM

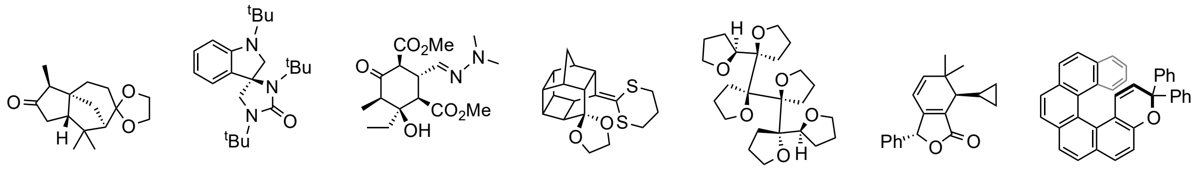

TIJWIS TIMRUE TIMTUE TITVUN TIVHUC TIXPOG TIYSIF

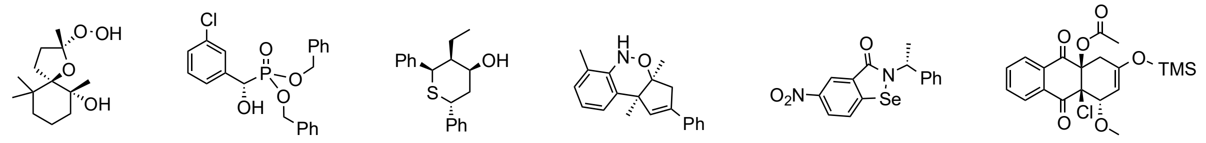

TIZGAN TIZMAT TMPHTB TOCHUR TODVEP TOHJOP

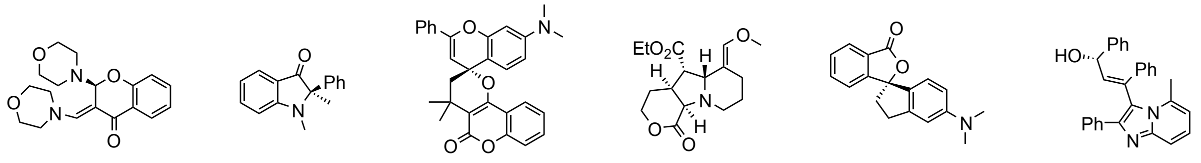

TOHMIP TOJBID TOPMUH TOPQOF TOQCUA TOSTOK

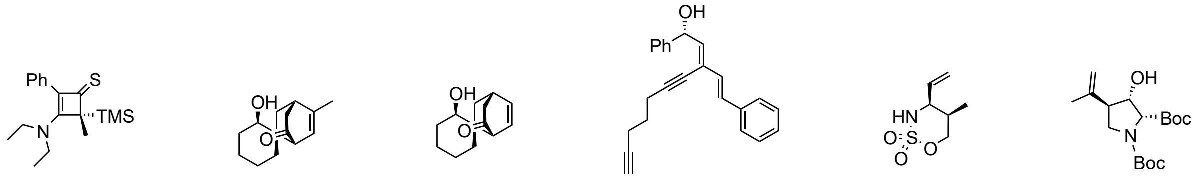

TOVMOG TOVNUP TOVZOV TOZBIV TOZYOY TUHLIR

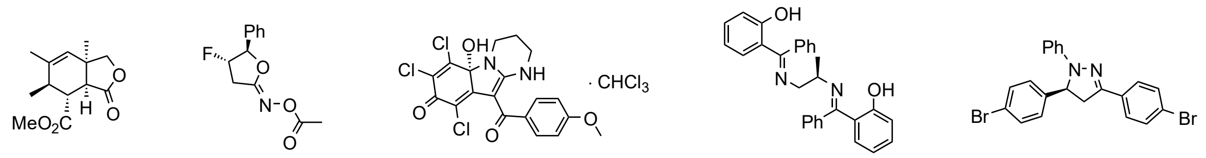

TULPUN TUMJIW TUNCEM TUNMOF TUNRIE

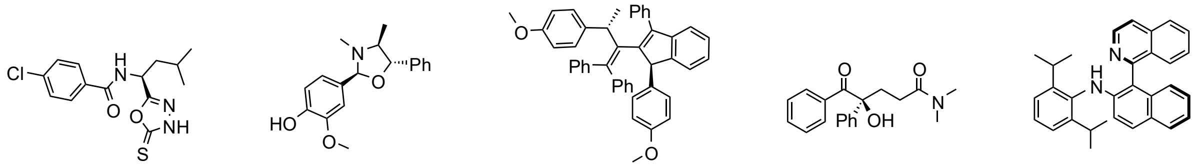

TUPMAT TUQBAJ TUSROP TUSVEK TUWFAT

CSD Code	Space Group	a / Å	b / Å	c / Å	α / °	β / °	γ / °	Publication Year	Reference
UBAVAW	$P2_12_12_1$	11.432	11.794	19.677	90	90	90	2011	1236
UBIJIY	$P2_12_12_1$	11.165	11.255	10.054	90	90	90	2001	1237
UCIPAY	$P2_1$	7.5332	9.7075	8.7091	90	109.469	90	2006	1238
UCUCEC	$P2_12_12_1$	9.184	9.445	26.482	90	90	90	2011	1239
UDAHIS	$P2_12_12_1$	8.0247	9.3837	21.631	90	90	90	2011	1240
UDIMIF	$P2_12_12_1$	7.6095	8.7618	22.784	90	90	90	2012	1241
UFANOF	$P2_12_12_1$	9.147	9.6695	14.872	90	90	90	2004	1242
UFEPUR	$P2_1$	8.51	6.8849	9.445	90	95.993	90	2008	1243
UFUGAG	$P2_1$	6.649	12.867	9.6024	90	91.4588	90	2018	1244
UGUHUZ	$P2_12_12_1$	5.788	17.499	19.463	90	90	90	2002	1245
UHACIP	$P2_12_12_1$	7.578	8.978	12.248	90	90	90	2002	1246
UHASIF	$P2_12_12_1$	10.478	10.684	17.44	90	90	90	2002	1247
UHASOL	$P2_1$	8.3194	10.738	11.011	90	101.232	90	2002	1247
UHAWUV	$P2_12_12_1$	10.129	10.428	13.195	90	90	90	2002	1248
UHUCEH	$P2_12_12_1$	7.394	11.499	29.003	90	90	90	2015	1249
UJEYEN	$P2_12_12_1$	7.8299	10.284	17.242	90	90	90	2003	1250
UJIVIU	$P2_12_12_1$	11.602	12.822	13.428	90	90	90	2016	1251
UKUTOJ	$P2_12_12_1$	7.469	8.452	31.817	90	90	90	2003	1252
ULAKAT	$P2_12_12_1$	9.661	10.475	12.72	90	90	90	2003	1253
ULAWUB	$P2_1$	5.1263	8.6668	11.257	90	91.76	90	2015	1254
ULEBOE	$P2_12_12_1$	10.622	10.706	18.665	90	90	90	2016	1255
ULEKED	$P2_1$	10.417	7.846	15.614	90	109.024	90	2016	1256
ULEROT	$P2_12_12_1$	10.844	11.431	19.27	90	90	90	2011	1257
ULIXIX	$P2_1$	5.3978	14.004	11.449	90	96.5658	90	2010	1258
UMEPUX	$C2$	16.731	5.8	14.801	90	108.49	90	2003	1259
UNERIQ	$P2_12_12_1$	7.768	15.017	18.503	90	90	90	2016	1260
UNOCIJ	$P2_12_12_1$	7.8787	12.446	24.501	90	90	90	2004	1261
UNOJAK	$P2_12_12_1$	9.1314	12.473	12.871	90	90	90	2013	1262
UPAZAN	$P3_1$	9.56	9.56	11.257	90	90	120	2011	1263
UPEPIP	$P2_12_12_1$	7.749	11.197	14.083	90	90	90	2010	1264
UPOFAI	$P2_1$	10.351	7.7827	11.328	90	103.741	90	2016	1265
UQAZUI	$P2_12_12_1$	8.2763	10.963	24.066	90	90	90	2011	1266
UQIDUV	$P2_12_12_1$	12.463	14.005	14.187	90	90	90	2016	1267
UQIFAD	$P2_12_12_1$	8.0478	18.796	19.243	90	90	90	2016	1267
URADUN	$P2_1$	11.388	11.458	13.489	90	109.752	90	2011	1268
URAPIN	$P2_12_12_1$	7.7712	10.841	18.976	90	90	90	2011	1269
URERUG	$P3_2$	10.857	10.857	11.492	90	90	120	2017	1270
USONUM	$P2_12_12_1$	11.081	12.439	12.544	90	90	90	2010	1271
UTIFAG	$P2_12_12_1$	10.326	15.021	16.203	90	90	90	2016	1272
UTIFOU	$P2_12_12_1$	7.941	11.929	13.923	90	90	90	2016	1272
UVAQEP	$P2_12_12_1$	7.8972	15.021	16.979	90	90	90	2016	1273
UVATIV	$C2$	17.796	6.252	20.307	90	90.601	90	2011	1274
UVAXAR	$P2_12_12_1$	8.0477	9.343	27.225	90	90	90	2011	1275
UVIRAT	$P2_12_12_1$	9.242	12.376	18.093	90	90	90	2011	1276
UVOTUV	$P2_1$	9.5306	15.631	10.528	90	104.282	90	2011	1277
UVUQIM	$P2_12_12_1$	5.68	8.405	21.474	90	90	90	2010	1278
UWIROJ	$P2_12_12_1$	5.882	14.797	17.754	90	90	90	2016	1279
UWUQAG	$P2_1$	9.0852	8.3949	11.196	90	106.71	90	2016	1280
UXINUM	$P2_1$	13.092	5.674	14.417	90	106.217	90	2016	1281
UXIPAT	$P2_12_12_1$	13.032	14.817	17.543	90	90	90	2010	1282
UXISEA	$P2_12_12_1$	5.9163	11.863	26.721	90	90	90	2011	1283
UXODAN	$P2_12_12_1$	8.6924	9.9931	22.187	90	90	90	2011	1284
UYIJAO	$P2_1$	8.4105	5.6321	10.022	90	107.78	90	2011	1285

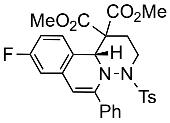
UBAVAW

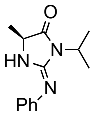
UBIJIY

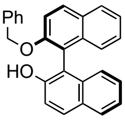
UBULUB

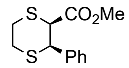
UCIPAY

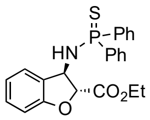
UCUCEC

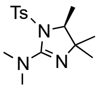
UDAHIS

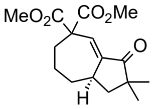
UDIMIF

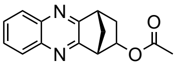
UFANOF

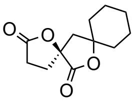
UFEPUR

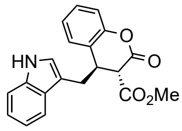
UFUGAG

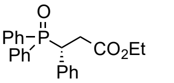
UGUHUZ

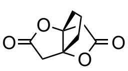
UHACIP

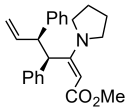
UHASIF

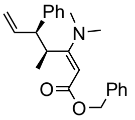
UHASOL

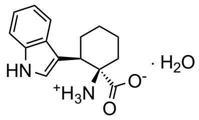
UHAWUV

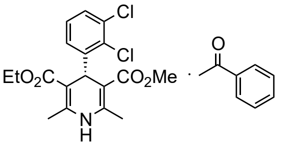
UHUCEH

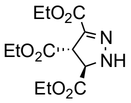
UJEYEN

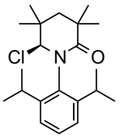
UJIVIU

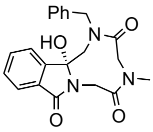
UKUTOJ

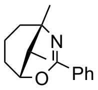
ULAKAT

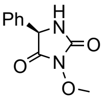
ULAWUB

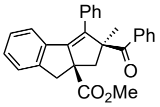
ULEBOE

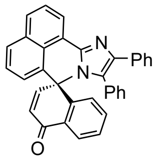
ULEKED

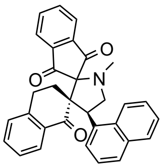
ULEROT

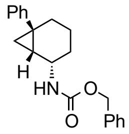
ULIXIX

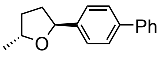
UMEPUX

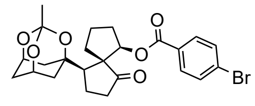
UNERIQ

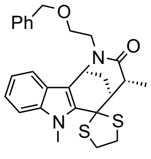
UNOCIJ

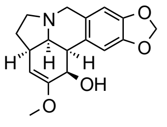
UNOJAK

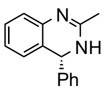
UPAZAN

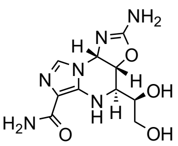
UPEPIP

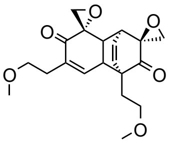
UPOFAI

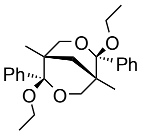
UQAZUI

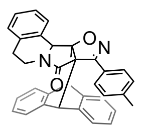
UQIDUV

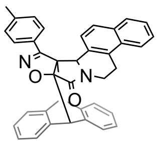
UQIFAD

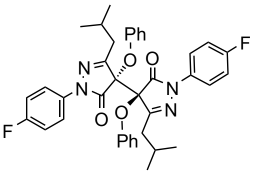
URADUN

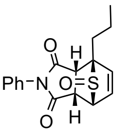
URAPIN

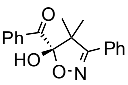
URERUG

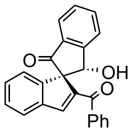
USONUM

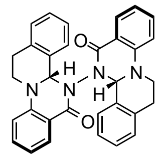
UTIFAG

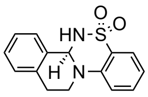

UTIFOU

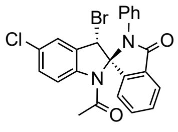

UVAQEP

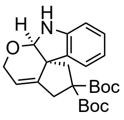

UVATIV

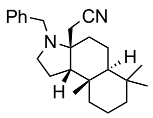

UVAXAR

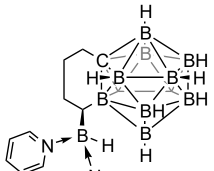

UVIRAT

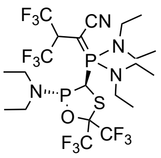

UVOTUV

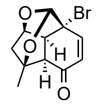

UVUQIM

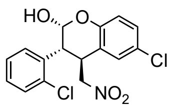

UWIROJ

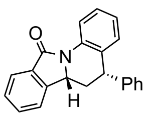

UWUQAG

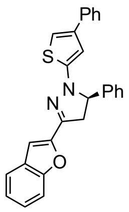

UXINUM

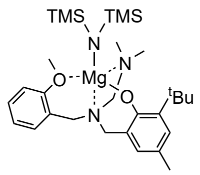

UXIPAT

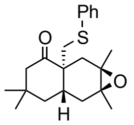

UXISEA

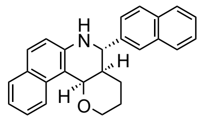

UXODAN

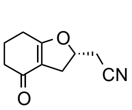

UYIJAO

CSD Code	Space Group	a / Å	b / Å	c / Å	α / °	β / °	γ / °	Publication Year	Reference
VABGUA	$P2_12_12_1$	11.239	17.932	9.424	90	90	90	1988	1286
VACVUQ	$P2_12_12_1$	8.117	13.209	14.477	90	90	90	1998	1287
VAGVEG	$P2_1$	5.4973	8.3407	11.846	90	90.166	90	2010	1288
VALNII	$P2_12_12_1$	5.41	12.669	25.696	90	90	90	2015	1289
VALNUT	$P2_12_12_1$	7.939	13.091	16.816	90	90	90	2011	1290
VAMROS	$P2_1$	6.1017	8.0077	21.337	90	93.404	90	2012	1291
VANTOT	$P2_1$	12.52	14.224	13.701	90	114.59	90	1989	1292
VAQNOS	$P2_12_12_1$	7.547	24.407	6.067	90	90	90	2012	1293
VAQXOC	$P2_1$	6.057	9.272	15.708	90	99.941	90	2012	1294
VAQYET	$P2_1$	8.6585	8.5691	9.4347	90	93.358	90	2012	1295
VARLIK	$P2_1$	9.3582	11.232	11.065	90	94.223	90	2003	1296
VARSOZ	$P2_12_12_1$	9.7095	13.75	14.304	90	90	90	2017	1297
VASTOB	$P2_12_12_1$	8.3892	15.839	18.308	90	90	90	2017	1298
VAVZEX	$P2_12_12_1$	8.23	9.34	12.6	90	90	90	1989	1299
VAWQUG	$P2_12_12_1$	15.252	22.075	5.6408	90	90	90	2004	1300
VAXHUZ	$P2_12_12_1$	8.3225	10.151	22.155	90	90	90	2012	1301
VAXJUB	$P2_12_12_1$	5.6799	13.596	15.013	90	90	90	2012	1302
VAXLEN	$P2_1$	6.7204	8.6319	13.131	90	90.268	90	2012	1303
VAXLUD	$P2_12_12_1$	14.536	12.816	8.453	90	90	90	2012	1303
VAXMIS	$P2_1$	7.487	6.6303	13.487	90	105.544	90	2012	1303
VAYPAN	$P2_1$	6.1243	14.424	10.767	90	104.165	90	2005	1304
VAYQAQ	$I4$	22.607	22.607	6.0161	90	90	90	2018	1305
VAZNAL	$P2_12_12_1$	16.417	16.012	7.215	90	90	90	1989	1306
VEBTIG	$P2_12_12_1$	7.4291	8.8854	11.796	90	90	90	2005	1307
VEDZIN	$P2_12_12_1$	9.463	13.243	14.938	90	90	90	1989	1308
VEFFAP	$P2_12_12_1$	11.434	14.113	16.065	90	90	90	2012	1309
VEFXEL	$P2_12_12_1$	5.637	10.231	24.765	90	90	90	2012	1310
VEFYEM	$P2_12_12_1$	8.1778	8.9545	21.73	90	90	90	2012	1311
VEGDIW	$P3_2$	14.419	14.419	6.0541	90	90	120	2012	1312
VEGFAQ	$P2_1$	11.629	7.7435	12.297	90	90.516	90	2012	1312
VEHVEM	$P2_1$	8.0405	11.877	10.592	90	100.232	90	2017	1313
VELCOE	$P2_12_12_1$	5.0335	16.059	21.414	90	90	90	1990	1314
VELGUQ	$P2_12_12_1$	10.533	12.448	13.668	90	90	90	2012	1315
VELVEN	$P2_12_12_1$	10.432	10.742	19.819	90	90	90	1990	1316
VEMHIH	$P2_12_12_1$	7.1591	10.236	16.713	90	90	90	2017	1317
VENJUU	$P2_12_12_1$	8.869	10.552	17.902	90	90	90	2006	1318
VEPWOD	$P2_12_12_1$	8.209	10.253	12.101	90	90	90	2006	1319
VEQBEB	$P2_12_12_1$	7.2945	14.207	17.459	90	90	90	2017	1320
VEQDAZ	$R3$	20.404	20.404	13.662	90	90	120	2017	1320
VEQDIH	$P2_12_12_1$	9.8687	12.284	15.482	90	90	90	2017	1320
VEQXEV	$P2_12_12_1$	9.1596	11.398	12.233	90	90	90	2006	1321
VERJUX	$P2_1$	12.026	7.199	13.326	90	96.33	90	1990	1322
VESMOW	$P2_12_12_1$	10.237	11.013	14.289	90	90	90	2000	1323
VESVOF	$P2_1$	10.383	9.893	13.212	90	98.41	90	2006	1324
VEVKAK	$P2_12_12_1$	8.1706	12.286	23.69	90	90	90	2012	1325
VEZMOF	$P2_1$	8.5012	5.8031	13.167	90	103.618	90	2018	1326
VEZMUL	$P2_12_12_1$	5.4221	9.3338	25.191	90	90	90	2018	1326
VEZNAS	$P2_12_12_1$	9.6647	10.325	13.709	90	90	90	2018	1326
VICJAV	$P2_12_12_1$	9.947	15.743	18.185	90	90	90	2018	1327
VICPEE	$P2_1$	8.6438	7.4285	8.7806	90	94.451	90	2013	1328
VIDHAS	$P2_1$	7.9522	5.898	10.554	90	110.003	90	2007	1329
VIHBOE	$P2_12_12_1$	6.3779	11.151	22.536	90	90	90	2007	1330

CSD Code	Space Group	a / Å	b / Å	c / Å	α / °	β / °	γ / °	Publication Year	Reference
VIHCAR	$P2_12_12_1$	9.9745	11.205	12.151	90	90	90	2007	[1330]
VIJJEF	$P2_12_12_1$	5.35	14.41	24.847	90	90	90	2013	[1331]
VIKSEN	$P2_12_12_1$	9.413	11.967	21.721	90	90	90	1991	[1332]
VILBUN	$P2_12_12_1$	8.52	26.15	5.06	90	90	90	1991	[1333]
VILLAG	$P2_1$	10.389	5.5083	14.009	90	105.836	90	2018	[1334]
VIMLEL	$P6_1$	20.689	20.689	7.342	90	90	120	2018	[1335]
VINREQ	$P2_12_12_1$	6.6705	10.944	13.394	90	90	90	2007	[1336]
VINTUI	$P2_12_12_1$	7.6737	9.123	13.496	90	90	90	2003	[1337]
VIPGEI	$P2_12_12_1$	6.8789	8.0525	17.389	90	90	90	2013	[1338]
VIQCEF	$P2_1$	5.4244	11.419	15.941	90	99.108	90	2013	[1339]
VISBIJ	$P2_12_12_1$	11.175	11.302	14.146	90	90	90	2007	[1340]
VISFIN	$P2_1$	10.465	7.4563	13.87	90	101.473	90	2007	[1341]
VISGAG	$P2_12_12_1$	8.4962	9.3365	23.868	90	90	90	2007	[1341]
VIWREZ	$P2_12_12_1$	8.618	12.824	15.465	90	90	90	2007	[1342]
VIWTAZ	$P2_12_12_1$	7.2868	10.851	18.495	90	90	90	2019	[1343]
VIXYEJ	$P2_1$	9.5963	8.9633	13.404	90	91.801	90	2019	[1344]
VIYVEH	$P2_12_12_1$	11.459	12.08	13.655	90	90	90	2019	[1345]
VOHHUV	$P2_12_12_1$	5.26	13.053	17.08	90	90	90	1990	[1346]
VOLFEH	$P2_12_12_1$	9.625	11.067	26.676	90	90	90	1991	[1347]
VOLRET	$P2_1$	8.479	16.21	9.4367	90	109.342	90	1991	[1348]
VOLXID	$P2_12_12_1$	5.845	16.557	18.783	90	90	90	1991	[1349]
VOMXED	$P2_12_12_1$	7.2816	12.341	21.075	90	90	90	2019	[1350]
VONCAC	$P2_12_12_1$	9.038	9.488	12.294	90	90	90	1991	[1351]
VONQUN	$P2_12_12_1$	10.488	15.165	17.863	90	90	90	2011	[1352]
VORSUT	$P2_12_12_1$	5.3084	14.029	25.162	90	90	90	2019	[1353]
VOSCUD	$P2_12_12_1$	6.5107	9.4968	17.237	90	90	90	2013	[1354]
VOTTED	$P2_1$	9.759	8.068	10.696	90	106.48	90	1991	[1355]
VOTYOS	$P2_12_12_1$	5.925	15.014	17.081	90	90	90	1992	[1356]
VOVHUJ	$P2_12_12_1$	5.828	11.82	15	90	90	90	1992	[1357]
VUJVAX	$P2_12_12_1$	9.1	9.678	17.36	90	90	90	1992	[1358]
VUMSEC	$P2_12_12_1$	7.9642	11.428	13.761	90	90	90	2009	[1359]
VUNTAB	$P2_12_12_1$	10.094	11.275	17.835	90	90	90	2015	[1360]
VUQZIS	$P2_12_12_1$	8.033	10.739	18.778	90	90	90	2015	[1361]
VURXIR	$P2_12_12_1$	8.8259	14.64	19.755	90	90	90	2016	[1362]
VUSFAS	$P2_12_12_1$	9.872	12.077	16.125	90	90	90	2015	[1363]
VUXBEX	$P2_12_12_1$	9.221	10.655	32.257	90	90	90	2015	[1364]
VUZGAZ	$P2_12_12_1$	7.0739	8.2861	25.046	90	90	90	2010	[1365]

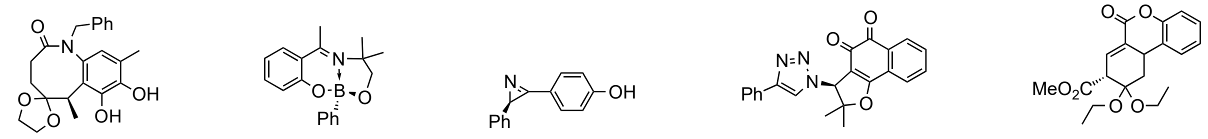

VABGUA VACVUQ VAGVEG VALNII VALNUT

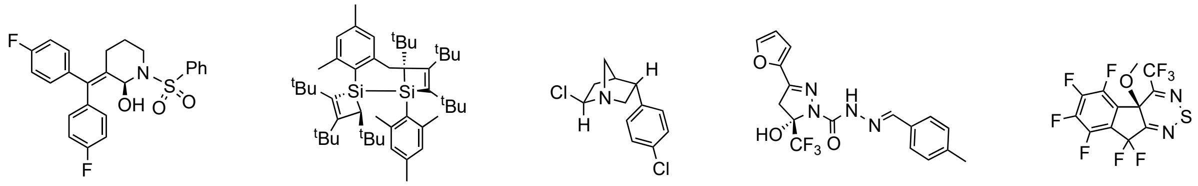

VAMROS VANTOT VAQNOS VAQXOC VAQYET

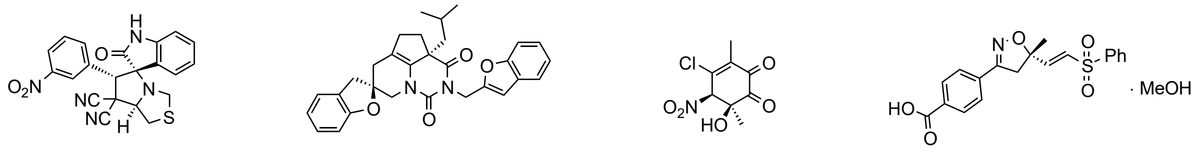

VARSOZ VASTOB VAVZEX VAWQUG

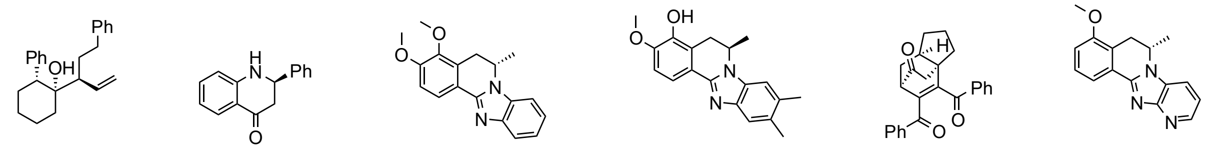

VAXHUZ VAXJUB VAXLEN VAXLUD VAYPAN VAXMIS

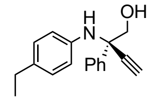
VAYQAQ

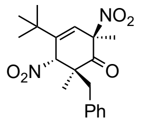
VAZNAL

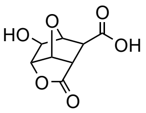
VEBTIG

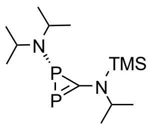
VEDZIN

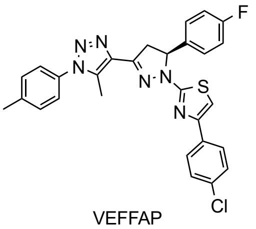
VEFFAP

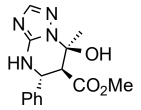
VEFXEL

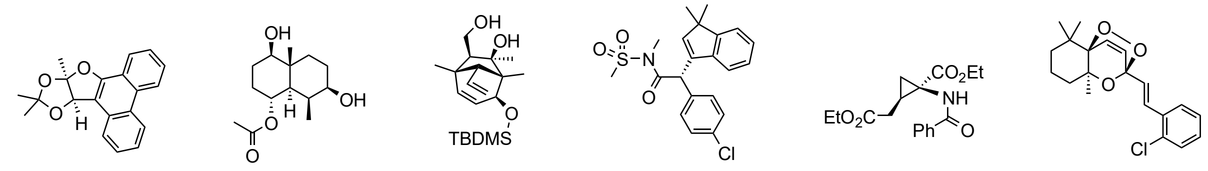

VEFYEM VEGDIW VEGFAQ VEHVEM VELCOE VELGUQ

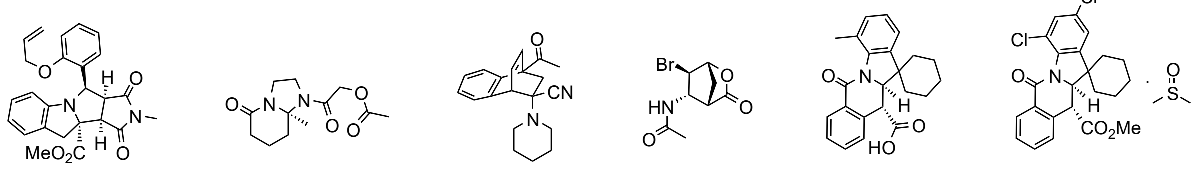

VELVEN VEMHIH VENJUU VEPWOD VEQBEB VEQDAZ

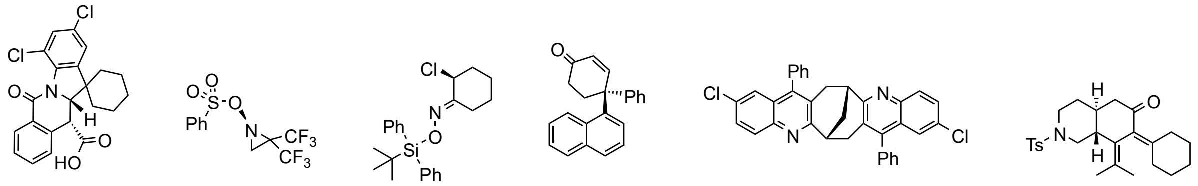

VEQDIH VEQXEV VERJUX VESMOW VESVOF VEVKAK

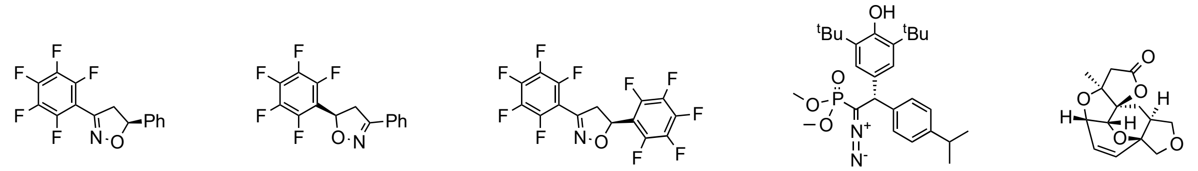

VEZMOF VEZMUL VEZNAS VICJAV VICPEE

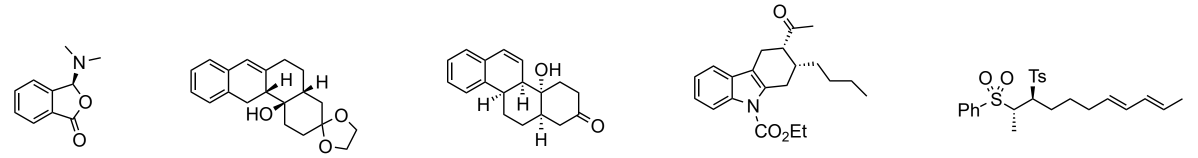

VIDHAS VIHBOE VIHCAR VIJJEF VIKSEN

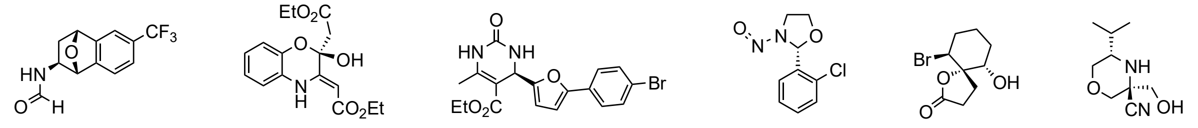

VILBUN VILLAG VIMLEL VINREQ VINTUI VIPGEI

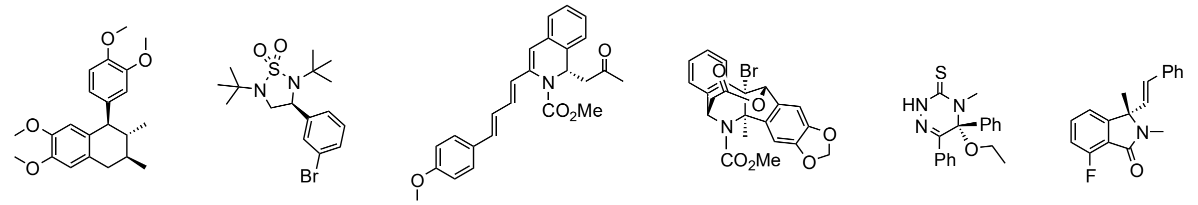

VIQCEF VISBIJ VISFIN VISGAG VIWREZ VIWTAZ

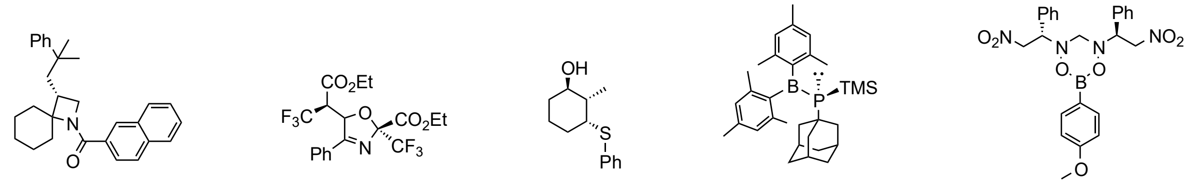

VIXYEJ VIYVEH VOHHUV VOLFEH VOLRET

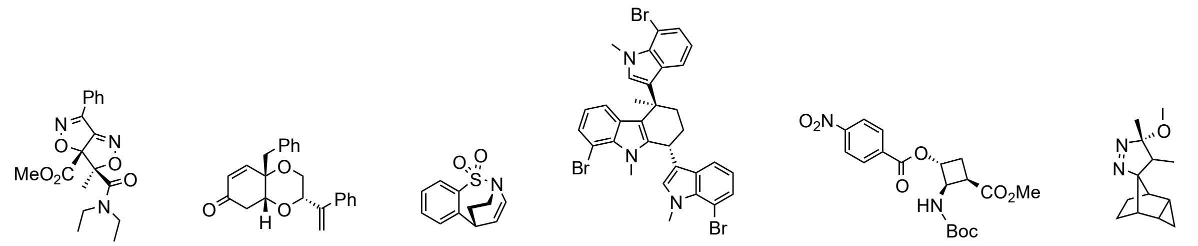

VOLXID VOMXED VONCAC VONQUN VORSUT VOSCUD

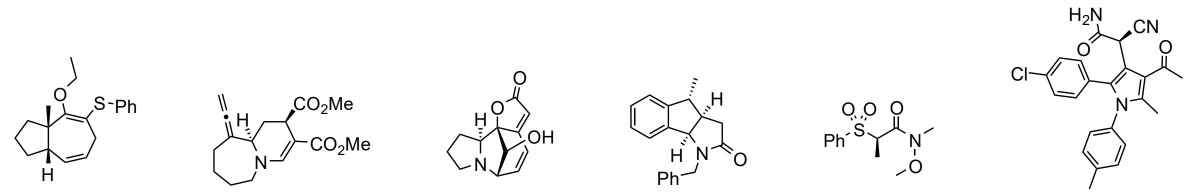

VOTTED VOTYOS VOVHUJ VUJVAX VUMSEC VUNTAB

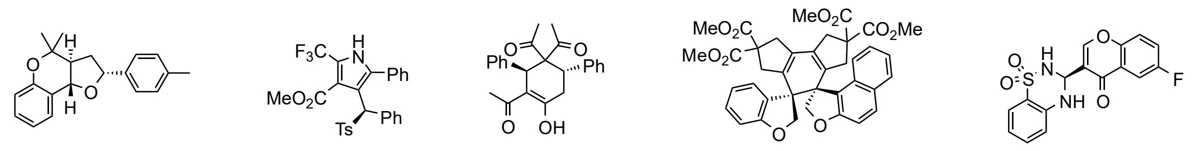

VUQZIS VURXIR VUSFAS VUXBEX VUZGAZ

CSD Code	Space Group	a / Å	b / Å	c / Å	α / °	β / °	γ / °	Publication Year	Reference
WACDEM	$P2_12_12_1$	5.9383	19.552	20.799	90	90	90	2016	1366
WAFGAO	$P2_1$	9.223	10.866	9.4767	90	115.29	90	2016	1367
WAFGUI	$P2_1$	8.7713	8.2519	12.829	90	104.429	90	2016	1367
WAFJAR	$P2_12_12_1$	7.6862	10.5	28.451	90	90	90	2016	1367
WAHZIP	$P2_12_12_1$	6.2803	13.385	20.519	90	90	90	2004	1368
WAHZUB	$P2_1$	11.079	7.588	11.595	90	110.306	90	2004	1368
WAKSOS	$P2_12_12_1$	5.6474	10.77	20.601	90	90	90	2010	1369
WAKSUY	$P2_12_12_1$	6.0556	15.165	16.454	90	90	90	2010	1369
WARGAA	$P2_12_12_1$	5.1817	17.718	18.732	90	90	90	2017	1370
WARTOB	$P2_12_12_1$	11.844	11.91	13.458	90	90	90	2017	1371
WEDGEU	$P2_12_12_1$	5.1674	11.234	21.321	90	90	90	2017	1372
WEDHIW	$P2_12_12_1$	10.869	16.352	8.006	90	90	90	1993	1373
WEFLOJ	$P2_12_12_1$	14.473	19.323	8.732	90	90	90	2006	1374
WEFLUP	$P2_12_12_1$	8.743	14.459	19.084	90	90	90	2006	1374
WEJBOC	$P2_12_12_1$	8.1	31.729	7.848	90	90	90	1994	1375
WEKFOH	$P2_12_12_1$	12.404	21.552	5.579	90	90	90	1994	1376
WEKHEA01	$P2_12_12_1$	5.785	11.268	20.673	90	90	90	2005	1377
WEMMIK	$P2_1$	10.394	20.309	11.363	90	97.23	90	1994	1378
WENQUD	$P2_1$	6.8796	18.353	7.5914	90	92.61	90	2013	1379
WEPHAA	$P2_12_12_1$	9.124	10.952	20.074	90	90	90	1994	1380
WEVBAB	$P2_12_12_1$	8.4093	12.309	17.135	90	90	90	2006	1381
WEZDUD	$P2_12_12_1$	8.8464	10.423	12.837	90	90	90	2018	1382
WEZTED	$P2_1$	6.587	10.692	11.061	90	93.961	90	2018	1383
WICBIW	$P2_12_12_1$	12.746	15.999	24.859	90	90	90	2018	1384
WICLAV	$P2_1$	10.467	15.729	5.662	90	101.2	90	1994	1385
WICZAL	$P2_1$	10.53	12.636	10.725	90	92.865	90	2013	1386
WIDCOB	$P2_12_12_1$	6.685	10.079	18.582	90	90	90	1994	1387
WIDJAX01	$P2_12_12_1$	8.1519	11.276	17.456	90	90	90	2018	1388
WIFZUH	$P2_12_12_1$	6.6498	8.4274	15.494	90	90	90	2007	1389
WIJCEA	$P2_1$	9.2784	14.172	9.8379	90	114.077	90	2018	1390
WIKBIB	$P2_12_12_1$	5.9478	6.7797	24.348	90	90	90	1994	1391
WILZEX	$P2_12_12_1$	5.1005	9.8564	29.147	90	90	90	2007	1392
WISCAE	$P2_12_12_1$	7.5254	10.721	18.042	90	90	90	2013	1393
WISYUU	$P2_1$	11.136	6.875	15.243	90	104.49	90	2013	1394
WIWWIK	$P2_12_12_1$	9.1769	10.289	14.62	90	90	90	2014	1395
WIWYAD	$P2_12_12_1$	10.682	11.597	12.74	90	90	90	2008	1396
WOBGOK	$P2_12_12_1$	8.3235	10.954	13.899	90	90	90	2008	1397
WOBRUB	$P2_12_12_1$	6.2225	14.026	21.046	90	90	90	2008	1398
WOCWAM	$P2_1$	9.684	5.2868	12.821	90	106.47	90	2000	1399
WODSIT	$P2_12_12_1$	10.307	12.023	15.822	90	90	90	2014	1400
WOFKAG	$P2_1$	6.4762	7.8291	18.92	90	92.615	90	2019	1401
WOJSEW	$P2_12_12_1$	7.8437	11.915	20.157	90	90	90	2019	1402
WOLGAH	$P2_12_12_1$	6.898	7.368	27.395	90	90	90	2014	1403
WOLGEL	$P2_12_12_1$	6.26	11.78	16.144	90	90	90	2014	1403
WOLGOV	$P2_12_12_1$	8.029	8.293	13.453	90	90	90	2014	1403
WOLHIQ	$P2_1$	7.606	6.726	11.088	90	100.6	90	2014	1403
WOMDAF	$P2_12_12_1$	7.7028	13.522	23.808	90	90	90	2014	1404
WOMDEK	$P2_1$	10.383	7.335	11.756	90	92.267	90	2019	1405
WOMLAL	$P4_1212$	9.21	9.21	37.021	90	90	90	1999	1406
WONNIX	$P2_12_12_1$	9.068	11.551	27.114	90	90	90	2008	1407
WOPCIP	$P2_12_12_1$	10.962	11.277	12.202	90	90	90	2015	1408
WOSHIV	$P2_12_12_1$	10.282	18.946	7.9101	90	90	90	2000	1409

CSD Code	Space Group	a / Å	b / Å	c / Å	α / °	β / °	γ / °	Publication Year	Reference
WOSYAE	$P2_12_12_1$	8.787	12.329	18.789	90	90	90	2000	[1410]
WUBFOO	$P2_12_12_1$	8.1766	8.8854	23.089	90	90	90	2002	[1411]
WUCBIF	$P2_1$	10.125	10.212	10.928	90	96.22	90	2002	[1412]
WUHGIP	$P2_12_12_1$	7.434	9.856	29.368	90	90	90	2002	[1413]
WURJOK	$P2_12_12_1$	8.292	9.959	16.982	90	90	90	2013	[1414]
WUSKAX	$P2_12_12_1$	7.575	12.048	16.684	90	90	90	2010	[1415]
WUTPOQ	$P2_12_12_1$	12.425	21.493	5.9427	90	90	90	2003	[1416]

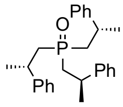
WACDEM
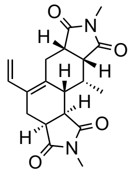
WAFGAO
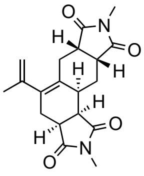
WAFGUI
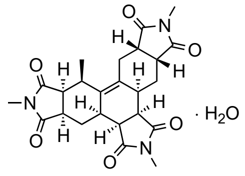
WAFJAR
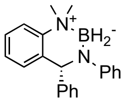
WAHZIP
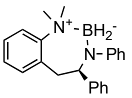
WAHZUB
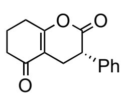
WAKSOS
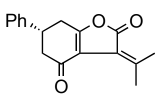
WAKSUY
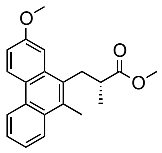
WARGAA
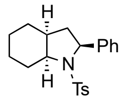
WARTOB
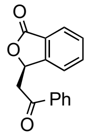
WEDGEU
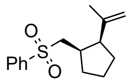
WEDHIW
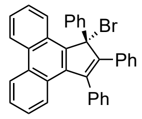
WEFLOJ
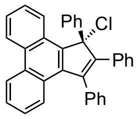
WEFLUP
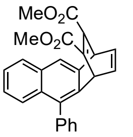
WEJBOC
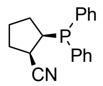
WEKFOH
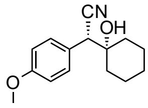
WEKHEA01
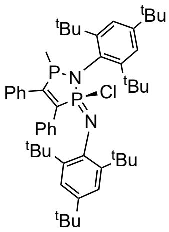
WEMMIK
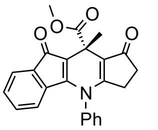
WENQUD
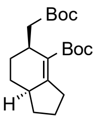
WEPHAA
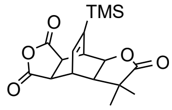
WEVBAB
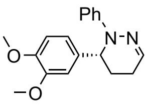
WEZTED
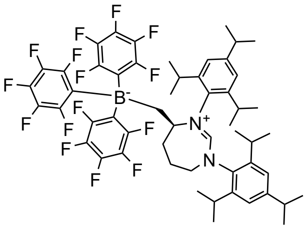
WICBIW
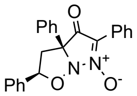
WICLAV
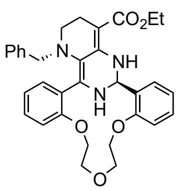
WICZAL
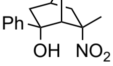
WIDCOB
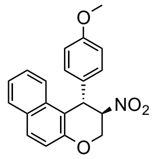
WIDJAX01
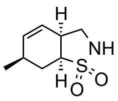
WIFZUH
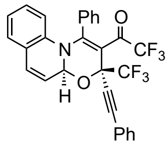
WIJCEA
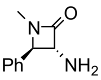
WIKBIB
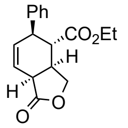
WILZEX
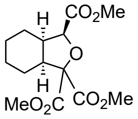
WISCAE
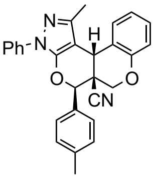
WISYUU
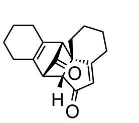
WIWWIK
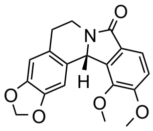
WIWYAD
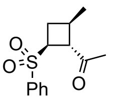
WOBGOK
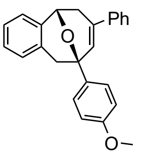
WOBRUB
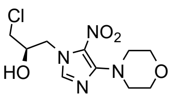
WOCWAM
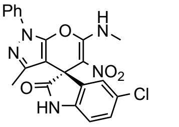
WODSIT
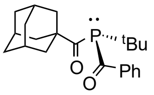
WOFKAG

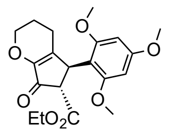

WOJSEW

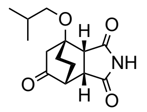

WOLGAH

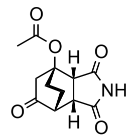

WOLGEL

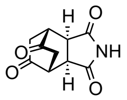

WOLGOV

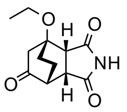

WOLHIQ

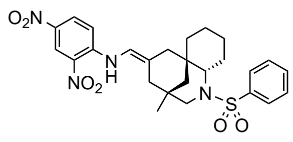

WOMDAF

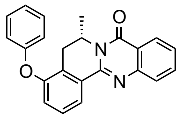

WOMDEK

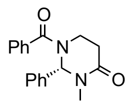

WOMLAL

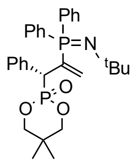

WONNIX

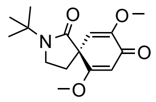

WOPCIP

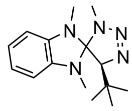

WOSHIV

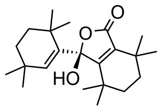

WOSYAE

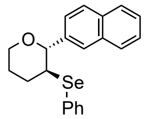

WUBFOO

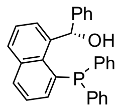

WUCBIF

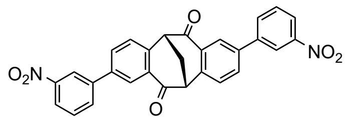

WUHGIP

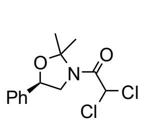

WURJOK

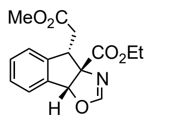

WUSKAX

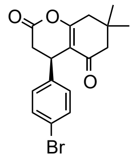

WUTPOQ

CSD Code	Space Group	a / Å	b / Å	c / Å	α / °	β / °	γ / °	Publication Year	Reference
XADZIM	$P2_12_12_1$	8.4042	11.936	21.361	90	90	90	2004	[1417]
XAGCEN	$P2_12_12_1$	8.57	9.459	12.232	90	90	90	2000	[1418]
XAGSAA	$P2_1$	8.107	6.179	15.753	90	92.74	90	2004	[1419]
XAHQAB	$P2_12_12_1$	10.006	14.676	19.773	90	90	90	2016	[1420]
XAJNOM	$P2_12_12_1$	4.8338	11.587	25.819	90	90	90	2004	[1421]
XALKEC	$P2_1$	8.676	12.879	9.4794	90	99.158	90	2012	[1422]
XARBAV	$P2_12_12_1$	7.6148	8.5592	9.3406	90	90	90	2012	[1423]
XATSAM	$P2_1$	5.88	6.483	16.927	90	91.2	90	2000	[1424]
XATYUM	$P2_12_12_1$	4.698	12.653	14.808	90	90	90	2000	[1425]
XAXTEX	$P2_12_12_1$	6.4478	7.9827	35.414	90	90	90	2012	[1426]
XAYDOS	$P2_1$	9.068	12.326	9.6447	90	117.87	90	2012	[1427]
XEJVEQ	$P2_1$	7.9678	10.943	15.044	90	95.755	90	2017	[1428]
XENLAE	$P2_12_12_1$	4.7156	9.3166	31.065	90	90	90	2006	[1429]
XENSAL	$P2_12_12_1$	6.5756	9.3484	11.749	90	90	90	2006	[1430]
XEQROA	$P2_12_12_1$	9.8576	17.821	19.603	90	90	90	1999	[1431]
XERGAF	$P2_12_12_1$	7.5161	8.9972	21.41	90	90	90	2017	[1432]
XETKIR	$P2_12_12_1$	5.8107	13.664	15.451	90	90	90	2006	[1433]
XEVLAO	$P2_1$	6.0568	8.6108	12.439	90	102.292	90	2018	[1434]
XEVLOC	$P2_1$	6.412	13.228	7.46	90	90.017	90	2018	[1434]
XEWVIH	$P2_12_12_1$	9.455	19.2	11.799	90	90	90	2018	[1435]
XEXNAP	$P2_12_12_1$	8.23	12.074	12.438	90	90	90	2000	[1436]
XIDHAU	$C2$	19.795	6.222	12.671	90	119.601	90	2007	[1437]
XIDZIW	$P2_1$	10.032	5.6962	14.973	90	97.189	90	2018	[1438]
XIPHAG	$P2_12_12_1$	6.3792	9.5181	22.695	90	90	90	2007	[1439,1440]
XIQXIG	$P2_12_12_1$	9.8745	18.241	23.774	90	90	90	2012	[1441]
XITWOP	$P2_12_12_1$	12.833	15.27	22.031	90	90	90	2019	[1442]
XIWVAD01	$P2_12_12_1$	7.9152	9.8145	15.322	90	90	90	2019	[1443]
XIYBIQ	$P4_3$	9.4397	9.4397	18.552	90	90	90	2002	[1444]
XOCYUJ	$P2_1$	7.689	6.524	9.087	90	101.48	90	2000	[1445]
XODBEX	$P2_12_12_1$	7.734	9.15	19.125	90	90	90	2000	[1446]
XOPNEV	$P2_12_12_1$	6.463	7.338	18.659	90	90	90	2002	[1447]
XOPNIZ	$P2_12_12_1$	6.7148	10.753	11.513	90	90	90	2002	[1447]
XOPZOR	$P2_12_12_1$	14.61	18.83	9.292	90	90	90	2001	[1448]
XOSJEU	$P2_12_12_1$	6.4817	11.87	21.43	90	90	90	2002	[1449]
XOSZAI	$P2_12_12_1$	10.247	10.461	13.544	90	90	90	2015	[1450]
XOVJUP	$P2_1$	6.6848	11.868	11.085	90	101.183	90	2015	[1451]
XUJLAR	$P2_12_12_1$	9.3692	12.201	13.577	90	90	90	2014	[1452]
XUPNED	$P2_12_12_1$	9.2832	11.256	11.695	90	90	90	2015	[1453]
XURDAR	$P2_1$	8.8436	12.76	9.619	90	111.761	90	2016	[1454]
XUYTEQ	$P2_12_12_1$	5.691	6.902	34.044	90	90	90	2003	[1455]
XUZXUM	$P2_12_12_1$	6.946	8.9632	10.352	90	90	90	2010	[1456]

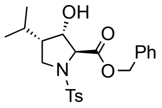
XADZIM

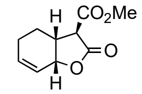
XAGCEN

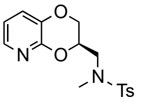
XAGSAA

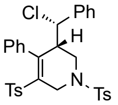
XAHQAB

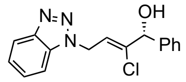
XAJNOM

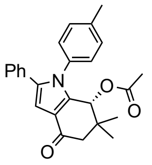
XALKEC

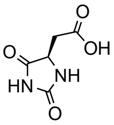
XARBAV

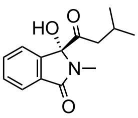
XATSAM

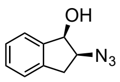
XATYUM

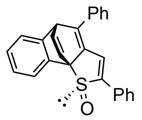
XAYDOS

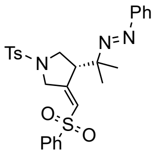
XEJVEQ

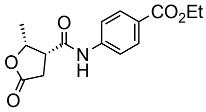
XENLAE

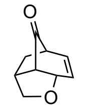
XENSAL

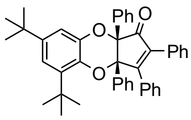
XEQROA

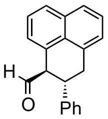
XERGAF

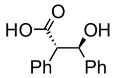
XETKIR

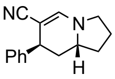
XEVLAO

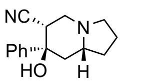
XEVLOC

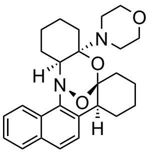
XEWVIH

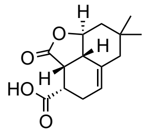
XEXNAP

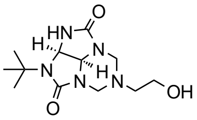
XIDHAU

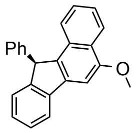
XIDZIW

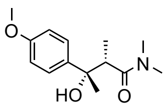
XIPHAG

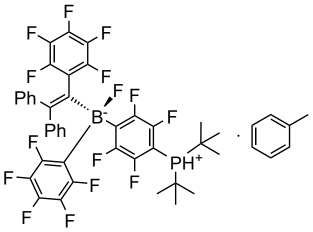
XIQXIG

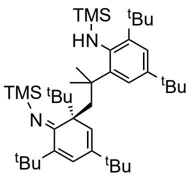
XITWOP

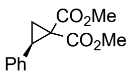
XIWVAD01

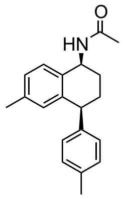
XIYBIQ

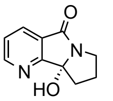
XOCYUJ

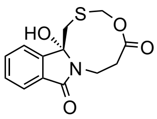
XODBEX

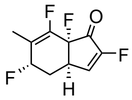
XOPNEV

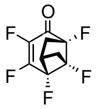
XOPNIZ

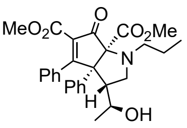
XOPZOR

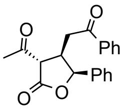
XOSJEU

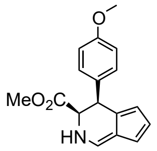
XOSZAI

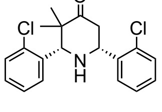
XOVJUP

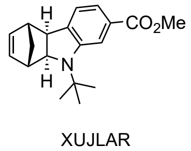
XUJLAR

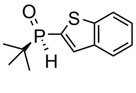
XUPNED

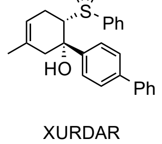
XURDAR

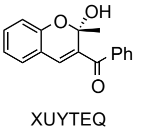
XUYTEQ

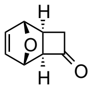
XUZXUM

CSD Code	Space Group	a / Å	b / Å	c / Å	α / °	β / °	γ / °	Publication Year	Reference
YACQAV	$P2_1$	8.0436	5.7529	15.487	90	90.757	90	2004	[1457]
YAJGOH	$P2_1$	6.0712	13.434	10.082	90	96.882	90	2011	[1458]
YALYOB	$P2_12_12_1$	8.5437	15.436	17.795	90	90	90	2010	[1459]
YAMBAS	$P2_12_12_1$	8.5073	10.295	20.661	90	90	90	2016	[1460]
YAMGEA	$P2_1$	8.1967	9.8216	9.2868	90	101.543	90	2011	[1461]
YAQWOE	$P2_1$	11.077	5.958	12.778	90	103.792	90	2011	[1462]
YAQYOH	$P6_1$	21.104	21.104	10.499	90	90	120	2017	[1463]
YAZQUO	$P2_1$	8.811	7.488	13.687	90	107.942	90	2017	[1464]
YEFMON	$P2_12_12_1$	8.3478	10.642	19.54	90	90	90	2009	[1465]
YEHSUC	$P2_12_12_1$	8.0221	10.018	22.589	90	90	90	2017	[1466]
YEKVOB	$P2_12_12_1$	6.0119	9.8632	21.278	90	90	90	2012	[1467]
YEPMOV	$P2_1$	8.74	10.66	10.565	90	111.49	90	1994	[1468]
YEZGAO	$P2_12_12_1$	7.6671	8.3382	23.437	90	90	90	2018	[1469]
YIBCIX	$P2_12_12_1$	9.4122	9.7013	15.144	90	90	90	2013	[1470]
YIBGOF	$P2_12_12_1$	8.181	9.25	14.085	90	90	90	1994	[1471]
YIHFOL	$P2_1$	8.3758	5.7112	10.699	90	90.428	90	2007	[1472]
YILNOY	$P2_12_12_1$	6.6135	8.0429	13.136	90	90	90	2013	[1473]
YISCIP	$P2_12_12_1$	9.497	10.58	13.75	90	90	90	2018	[1474]
YISFOW	$P2_12_12_1$	8.2239	8.8594	22.272	90	90	90	2008	[1475]
YIZSEG	$P2_12_12_1$	7.34	8.883	23.766	90	90	90	2008	[1476]
YODKUY	$P2_1$	5.972	8.5153	14.827	90	97.223	90	2008	[1477,1478]
YODTAO	$P2_1$	6.203	15.275	9.51	90	104.293	90	2013	[1479]
YOFVIB	$P2_12_12_1$	5.5757	8.7855	30.824	90	90	90	2019	[1480]
YOJLAL	$P4_1$	10.332	10.332	12.643	90	90	90	2007	[1481]
YOLROI	$P2_1$	8.4914	9.1548	12.479	90	95.838	90	2014	[1482]
YORBUF	$P2_12_12_1$	4.4361	14.128	20.646	90	90	90	2019	[1483]
YORSEF	$P2_12_12_1$	7.6193	10.971	11.858	90	90	90	2014	[1484]
YOSBUD	$P2_12_12_1$	9.495	27.003	8.165	90	90	90	1995	[1485]
YOWHEZ	$P2_12_12_1$	10.528	10.736	15.596	90	90	90	2015	[1486]
YUBQOD	$P2_12_12_1$	11.335	14.198	14.777	90	90	90	2015	[1487]
YUCSAR	$P2_1$	8.8558	7.3585	9.9622	90	101.62	90	2009	[1488]
YUDKAK	$P2_12_12_1$	7.5305	10.816	18.289	90	90	90	2009	[1489]
YUDMAL	$P2_12_12_1$	5.552	9.054	20.745	90	90	90	1995	[1490]
YUJBAG	$P2_12_12_1$	10.731	18.016	8.347	90	90	90	1995	[1491]
YULTOP	$P2_12_12_1$	6.2054	10.304	21.808	90	90	90	2009	[1492]
YUTFIC	$P2_1$	6.172	13.074	13.194	90	90.72	90	1995	[1493]
YUTFOI	$P2_1$	9.752	6.471	12.898	90	91.58	90	1995	[1493]

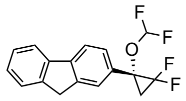

YACQAV

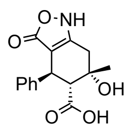

YAJGOH

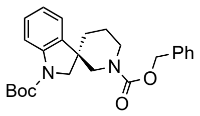

YALYOB

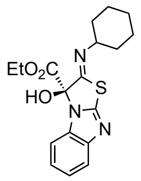

YAMBAS

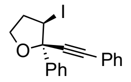

YAMGEA

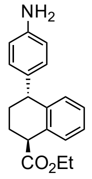

YAQWOE

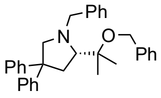

YAQYOH

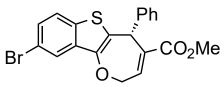

YAZQUO

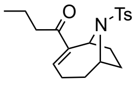

YEFMON

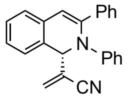

YEHSUC

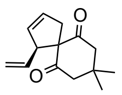

YEKVOB

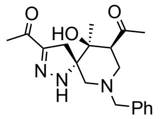

YEPMOV

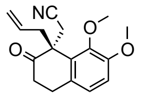

YEZGAO

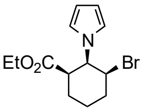

YIBCIX

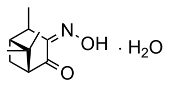

YIBGOF

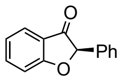

YIHFOL

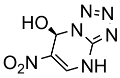

YILNOY

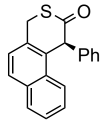

YISCIP

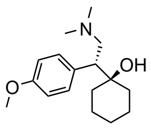

YISFOW

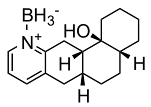

YIZSEG

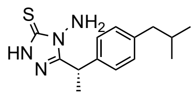

YODKUY

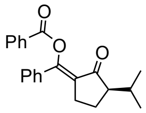

YODTAO

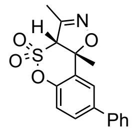

YOFVIB

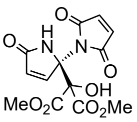

YOJLAL

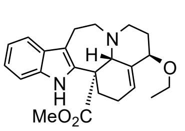

YOLROI

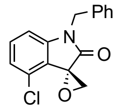

YORBUF

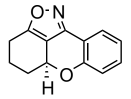

YORSEF

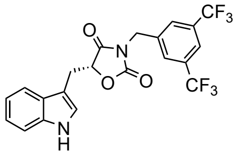

YOSBUD

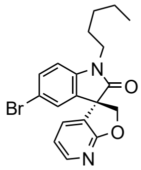

YOWHEZ

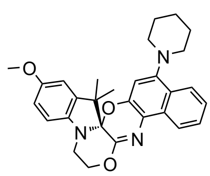

YUBQOD

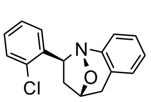

YUCSAR

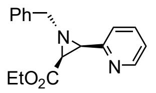

YUDKAK

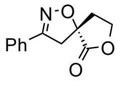

YUDMAL

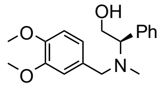

YUJBAG

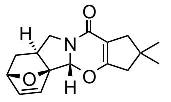

YULTOP

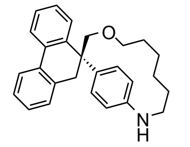

YUTFIC

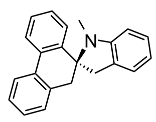

YUTFOI

CSD Code	Space Group	a / Å	b / Å	c / Å	α / °	β / °	γ / °	Publication Year	Reference
ZADMOG	$P2_12_12_1$	12.776	17.2	7.4384	90	90	90	1995	1494
ZAGVOV	$P2_1$	9.064	9.689	10.074	90	114.841	90	2015	1495
ZAJGAV	$P2_12_12_1$	8.0323	11.044	21.437	90	90	90	2017	1496
ZANDEX	$P2_12_12_1$	10.635	13.199	20.643	90	90	90	1995	1497
ZARQOA	$P2_12_12_1$	7.4412	10.655	17.446	90	90	90	2012	1498
ZAVZAX	$P1$	5.953	7.459	9.409	69.11	82.09	89.74	1995	1499
ZAYLAM	$P2_12_12_1$	9.526	10.194	16.391	90	90	90	1995	1500
ZEDLAV	$P2_12_12_1$	9.334	20.525	7.708	90	90	90	1994	1501
ZEFPAD	$P2_12_12_1$	5.0482	9.422	10.25	90	90	90	2012	1502
ZEFPUX	$P2_12_12_1$	5.058	5.222	16.054	90	90	90	2012	1502
ZEHCUN	$P2_12_12_1$	12.067	17.883	26.759	90	90	90	2017	1503
ZELVEU	$P2_1$	6.7838	11.793	12.664	90	104.498	90	2017	1504
ZEMBEB	$P2_12_12_1$	8.9233	10.977	15.592	90	90	90	2017	1505
ZERWUQ	$P2_1$	11.844	9.2994	15.71	90	103.174	90	2012	1506
ZESWOL	$P2_12_12_1$	9.5405	12.192	15.639	90	90	90	2013	1507
ZEWCUC	$P2_12_12_1$	5.6334	17.083	20.646	90	90	90	2018	1508
ZEWHUG	$P2_12_12_1$	6.3845	13.615	22.618	90	90	90	2013	1509
ZEWPIA	$P2_12_12_1$	6.873	12.929	14.988	90	90	90	1995	1510
ZEZROL	$P2_12_12_1$	8.633	21.147	8.561	90	90	90	1995	1511
ZIGYIX	$P2_1$	9.972	8.859	13.96	90	94.01	90	1995	1512
ZIKSER	$P2_12_12_1$	10.001	20.603	8.601	90	90	90	1995	1513
ZILCEC	$P2_1$	7.346	5.87	15.959	90	99.64	90	1995	1514
ZILCIG	$P2_12_12_1$	6.022	7.319	32.279	90	90	90	1995	1514
ZIMWEA	$P2_12_12_1$	6.1623	15.622	21.728	90	90	90	2018	1515
ZIVNOH	$P2_1$	6.798	13.675	9.722	90	99.72	90	1996	1516
ZIVSEC	$P2_12_12_1$	16.305	10.366	6.9	90	90	90	1996	1517
ZOBYEW	$P2_12_12_1$	7.9717	11.17	14.28	90	90	90	2014	1518
ZOCCIF	$P2_12_12_1$	10.67	14.524	16.368	90	90	90	2013	1519
ZOCLEK	$P2_12_12_1$	9.8678	11.051	18.812	90	90	90	2014	1520
ZOFKEN	$P2_12_12_1$	6.5618	7.9332	21.013	90	90	90	2019	1521
ZOMCEM	$P2_1$	5.1491	19.66	7.3046	90	98.612	90	2019	1522
ZOSROQ	$P2_12_12_1$	8.9323	12.078	13.107	90	90	90	2014	1523
ZOVFEV	$P2_12_12_1$	12.756	18.748	6.621	90	90	90	1995	1524
ZOWCOD	$P2_1$	5.9956	12.692	9.7791	90	98.813	90	1996	1525
ZOZWES	$P2_12_12_1$	10.079	11.335	12.235	90	90	90	2015	1526
ZOZZUJ	$P2_12_12_1$	5.809	15.514	13.883	90	90	90	1996	1527
ZUCZUS	$P2_1$	13.111	7.68	11.09	90	103.961	90	1996	1528
ZUDGOU	$P2_12_12_1$	6.786	11.817	16.18	90	90	90	1996	1529
ZUGQOH	$P2_12_12_1$	8.321	12.411	17.008	90	90	90	1996	1530
ZUMKAT	$P2_12_12_1$	7.191	8.713	14.101	90	90	90	1996	1531
ZUTFEB	$P2_1$	7.8821	11.618	10.827	90	111.059	90	2013	1532
ZUVZUN	$P2_1$	11.378	5.7382	13.148	90	114.27	90	2013	1533
ZUWTOA	$P2_12_12_1$	7.9188	10.815	11.261	90	90	90	1995	1534
ZUXYEY	$P2_12_12_1$	8.939	10.588	13.799	90	90	90	2015	1535
ZUXYIC	$P2_12_12_1$	9.051	10.638	14.281	90	90	90	2015	1535

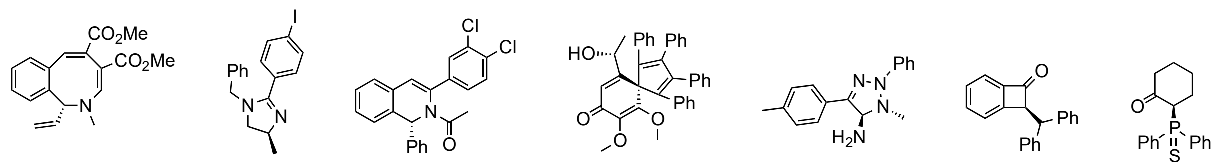

ZADMOG ZAGVOV ZAJGAV ZANDEX ZARQOA ZAVZAX ZAYLAM

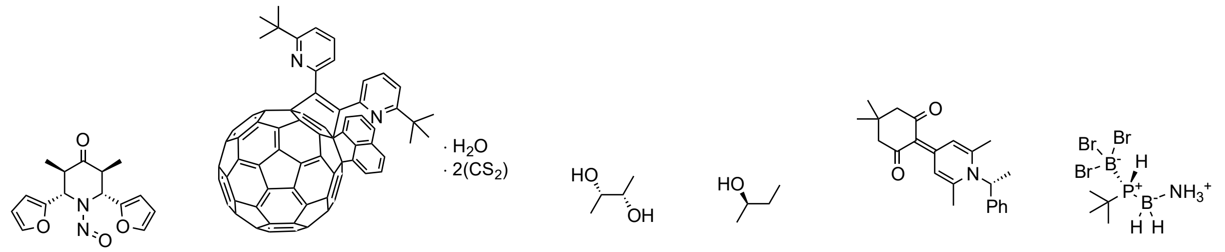

ZEDLAV ZEHCUN ZEFPAD ZEFPUX ZELVEU ZEMBEB

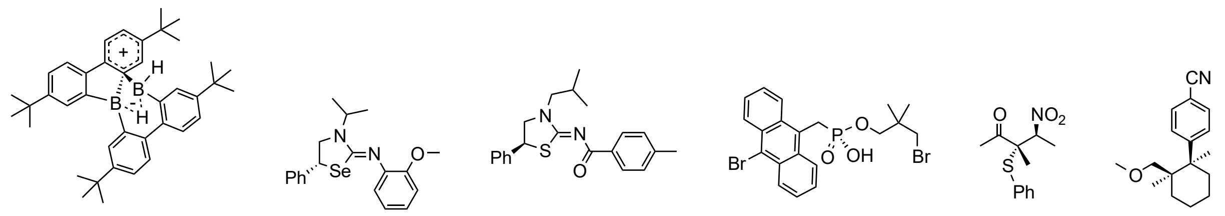

ZERWUQ ZESWOL ZEWCUC ZEWHUG ZEWPIA ZEZROL

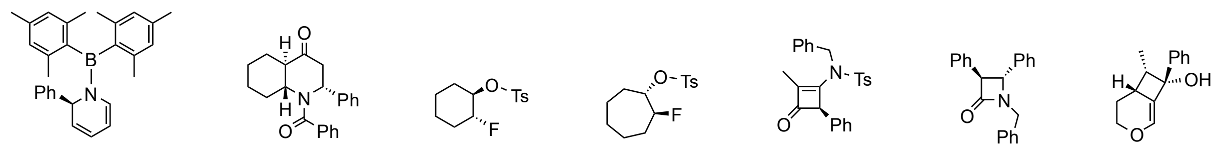

ZIGYIX ZIKSER ZILCEC ZILCIG ZIMWEA ZIVNOH ZIVSEC

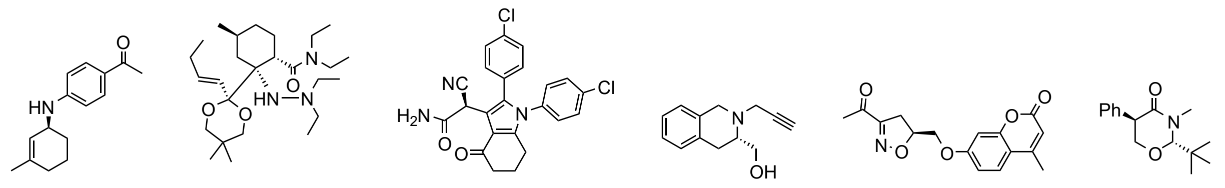

ZOBYEW ZOCCIF ZOCLEK ZOFKEN ZOMCEM ZOSROQ

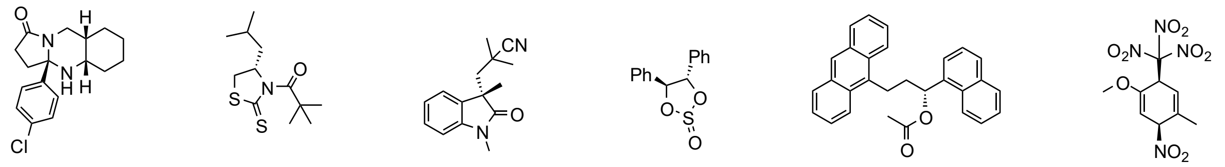

ZOVFEV ZOWCOD ZOZWES ZOZZUJ ZUCZUS ZUDGOU

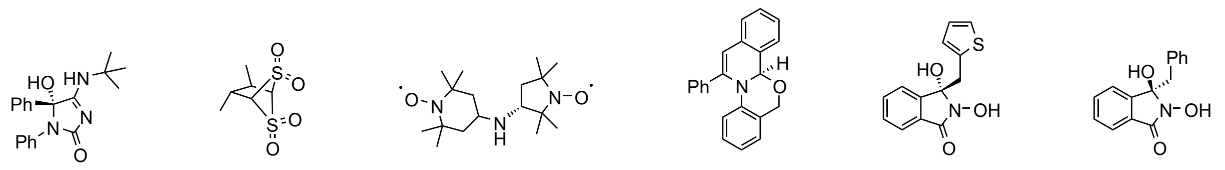

ZUGQOH ZUMKAT ZUTFEB ZUVZUN ZUXYEY ZUXYIC

Conglomerate crystals found by literature search

CSD Code	Space Group	a / Å	b / Å	c / Å	α / °	β / °	γ / °	Publication Year	Reference
ABAFAL	$P1$	5.8601	8.1656	16.715	97.332	97.876	91.229	2004	1536
AMETMA	$P2_12_12_1$	24.73	9.81	4.88	90	90	90	1971	1537
AVENAT	$P2_1$	10.1348	9.0044	10.187	90	91.337	90	2021	1538
AVOROV	$P2_12_12_1$	6.1403	17.442	18.062	90	90	90	2021	1539
AVORUB	$P2_12_12_1$	6.2115	17.488	18.342	90	90	90	2021	1539
AVOSIQ	$P2_12_12_1$	6.1039	6.5048	26.064	90	90	90	2021	1539
BAXPIA	$C2$	27.453	7.1250	13.017	90	104.47	90	2003	1540
BAXPOG	$C2$	27.787	7.3490	13.383	90	104.89	90	2003	1540
BEPSIA	$P2_12_12_1$	13.509	24.364	26.380	90	90	90	2013	1541
BICZET	$P2_12_12_1$	9.000	14.151	16.681	90	90	90	2004	1542,1543
BOHTIB	$P2_1$	10.776	7.241	13.028	90	95.84	90	1999	1544
BOPTAE	$P2_1$	11.282	6.515	12.958	90	98.767	90	2019	1545
BOPTEI	$P2_1$	11.435	6.4322	12.819	90	104.40	90	2019	1545
BOPTIM	$P2_1$	11.355	6.5917	13.076	90	97.120	90	2019	1545
BOPTUY	$P2_12_12_1$	7.0043	16.476	17.303	90	90	90	2019	1545
BOPVEK	$P2_12_12_1$	7.1595	16.639	17.369	90	90	90	2019	1545
CIJRAP	$P2_12_12_1$	8.3100	10.926	25.694	90	90	90	2006	1546
CUQGOK	$P2_1$	9.5339	12.880	9.005	90	100.52	90	1999	1547
DAHSUA01	$P2_12_12_1$	5.9552	11.992	18.811	90	90	90	2021	1548
DBRDOX	$P2_12_12_1$	5.680	7.590	15.820	90	90	90	1963	1549
DCACNA	$P2_12_12$	7.79	7.74	8.38	90	90	90	1972	1550
DEFQAG01	$P2_12_12_1$	6.3842	9.4106	26.866	90	90	90	2003	14
DHPROA	$P4_12_12$	5.607	5.607	28.753	90	90	90	1974	1551
DOSBUL	$P6_5$	13.849	13.849	22.225	90	90	120	2019	1552
DOSCOG	$P6_1$	13.875	13.847	22.239	90	90	120	2019	1552
EDIKOS	$P2_1$	9.8813	18.133	11.477	90	103.33	90	2007	1553
EJUFOE	$P6_1$	7.4471	7.4471	52.900	90	90	120	2003	1554
EKIGUD	$P2_12_12_1$	10.490	10.541	13.322	90	90	90	2021	1555
FAYDUF	$P2_1$	10.069	9.4318	10.175	90	95.178	90	2005	1556,1557
FEDYAR	$P2_12_12_1$	7.4315	10.843	12.674	90	90	90	2017	1558
FIQNIE	$P2_1$	9.5288	8.2186	11.473	90	108.52	90	2013	1559
FUSJAE	$P2_12_12_1$	9.368	26.703	6.043	90	90	90	1987	1560
FUSJEI	$P2_12_12_1$	13.868	20.457	6.471	90	90	90	1987	1560
GASCEK	$P2_1$	8.0612	7.4986	13.891	90	105.99	90	2012	1561,1562
GEJXEZ	$P3_121$	15.257	15.257	21.418	90	90	120	2006	1563
GEJXID	$P3_221$	14.147	14.147	23.316	90	90	120	2006	1563
GEPRID	$P3_121$	14.165	14.165	23.145	90	90	120	2006	1563
GEPROJ	$P3_221$	15.355	15.355	21.479	90	90	120	2006	1563
HEGGAD	$P2_12_12_1$	5.9633	15.128	32.541	90	90	90	2012	1564,1565
HEZXAM	$P2_12_12_1$	6.0584	8.0933	21.915	90	90	90	2007	1566
HIRYIS	$P4_12_12$	8.299	8.299	25.826	90	90	90	2013	1567
HIRYOY	$P4_32_12$	8.3066	8.3066	25.829	90	90	90	2013	1567
HIWHON	$P2_1$	5.8761	20.905	16.212	90	98.169	90	2019	1568
HOSKAC	$P2_12_12_1$	9.7300	12.372	13.365	90	90	90	2009	1569
HOXZAW	$P2_12_12_1$	10.647	12.387	18.395	90	90	90	2009	1570
HUDNIE	$P2_12_12_1$	10.652	12.387	18.413	90	90	90	2009	1570
IVUSEY	$P2_1$	12.824	7.7239	18.065	90	104.56	90	2011	1571
JORJEF02	$P2_12_12_1$	8.937	12.021	19.771	90	90	90	1992	1572
LABWEQ	$P2_1$	11.207	7.078	13.436	90	93.968	90	1999	1573
LECFOP	$P4_3$	17.796	17.796	12.384	90	90	90	2006	1574
LIDCOT	$P2_1$	15.055	5.2629	15.138	90	116.99	90	2018	1575
LIDCUZ	$P2_1$	15.033	5.2605	15.157	90	116.95	90	2018	1575
LTHREO	$P2_12_12_1$	13.611	7.738	5.142	90	90	90	1950	1576

CSD Code	Space Group	a / Å	b / Å	c / Å	α / °	β / °	γ / °	Publication Year	Reference
LUTQUP	$P2_12_12_1$	7.5425	13.671	20.401	90	90	90	2020	[1577]
LUTRAW	$P2_1$	11.449	5.7017	14.095	90	107.69	90	2020	[1577]
LUTREA	$P2_1$	11.775	5.7683	13.210	90	105.95	90	2020	[1577]
MAGSAQ	$P4_3$	7.6223	7.6223	23.186	90	90	90	2010	[1578,1579]
MHCEPO	$P2_12_12_1$	8.96	18.60	5.80	90	90	90	1969	[1580]
MONQIQ	$P3_221$	9.6710	9.6710	22.674	90	90	90	2009	[1581]
MSUFAC	$P2_12_12_1$	7.832	8.234	5.360	90	90	90	1969	[1582,1583]
MTYROS	$P2_1$	5.956	5.2992	13.866	90	104.36	90	1974	[1584]
MUTBEJ	$P2_12_12_1$	4.900	7.586	25.89	90	90	90	2010	[1585]
MUTBIN	$P2_12_12_1$	4.853	7.838	27.210	90	90	90	2010	[1585]
MUTBOT	$P2_12_12_1$	4.873	7.669	26.510	90	90	90	2010	[1585]
NUMZUT	$P2_1$	7.2526	11.663	9.3657	90	94.231	90	2020	[1586]
OCODOD	$P2_12_12_1$	5.436	8.208	25.760	90	90	90	2021	[1587]
OCOFIZ	$P2_1$	8.084	13.535	13.535	90	106.92	90	2021	[1587]
OFUQEO	$P2_12_12_1$	5.9134	7.7193	31.935	90	90	90	2018	[1588]
OFUQIS	$P2_12_12_1$	5.9479	12.365	12.365	90	90	90	2018	[1588]
OFUQOY	$P2_12_12_1$	6.4325	15.152	15.740	90	90	90	2018	[1588]
OGUCOJ	$P2_1$	10.920	8.1845	18.107	90	106.74	90	2009	[1589]
OVIDUT	$P2_1$	8.0959	7.5319	13.899	90	105.90	90	2011	[1590]
OXIDAD	$P2_12_12_1$	6.5571	8.028	24.199	90	90	90	2021	[1591]
OXIGIO	$P2_1$	9.0259	8.4265	8.9633	90	95.811	90	2021	[1591]
OXIGUA	$P2_12_12_1$	6.1701	9.4160	21.474	90	90	90	2021	[1591]
PALNEX	$P4_32_12$	11.402	11.402	43.218	90	90	90	2012	[1592]
PEGGAJ	$C2$	23.418	13.518	24.598	90	108.52	90	1993	[1593]
PLACTA01	$P2_12_12_1$	5.8389	8.3195	16.969	90	90	90	2011	[1594]
PUMILC02	$P2_1$	8.592	7.574	11.612	90	109.86	90	2001	[1595,1596]
QETJII	$P2_12_12_1$	9.8591	12.605	13.763	90	90	90	2001	[1597,1598]
QQQAUJ05	$P2_1$	8.7829	5.9985	31.017	90	96.952	90	2014	[1599]
QQQGXG	$P2_12_12_1$	12.75	9.87	8.27	90	90	90	1974	[1600]
QUZYES	$P4_32_12$	8.8893	8.8893	45.353	90	90	90	2020	[1601]
QUZYOC	$P2_12_12_1$	5.442	8.871	28.748	90	90	90	2020	[1602]
QUZYUI	$P2_12_12_1$	5.6145	8.7362	30.511	90	90	90	2020	[1602]
QUZZAP	$P2_12_12_1$	5.4955	8.8034	29.525	90	90	90	2020	[1602]
SEYPUH	$P2_12_12_1$	5.799	13.552	19.654	90	90	90	1990	[1603]
SNBMXZ	$P2_12_12_1$	12.279	9.755	8.829	90	90	90	1976	[1604]
SUCDUR	$P2_1$	10.823	6.6783	12.818	90	108.89	90	2015	[1605]
TCLCYH	$P2_12_12$	7.60	7.54	7.72	90	90	90	1949	[1606]
TEXBUV	$P4_32_12$	8.3080	8.3080	40.738	90	90	90	2013	[1607]
UCUSAN	$P4_1$	17.808	17.808	12.400	90	90	90	2006	[1574]
UTEFIL	$P2_12_12_1$	5.0750	10.279	19.507	90	90	90	2021	[1608]
UTEFUX	$P2_12_12_1$	5.0440	10.440	19.180	90	90	90	2021	[1608]
UTEFUX01	$P2_1$	10.838	5.0955	10.128	90	99.926	90	2021	[1608]
UTEGAE	$P2_1$	4.9282	8.7117	17.839	90	97.491	90	2021	[1608]
UTEGEI	$P2_1$	12.996	4.9826	14.385	90	91.383	90	2021	[1608]
UTEGEI01	$P2_12_12_1$	10.262	19.125	5.0900	90	90	90	2021	[1608]
UTEHIN	$P2_1$	13.251	4.9950	15.815	90	94.094	90	2021	[1608]
UTEHIN01	$P2_12_12_1$	22.583	10.088	5.0328	90	90	90	2021	[1608]
UTEZOL	$P2_12_12_1$	8.7643	10.729	15.195	90	90	90	2021	[1609]
VAWMEM	$C2$	13.105	7.1018	14.644	90	94.154	90	2005	[1610]
XECTIL	$P2_1$	12.831	5.037	17.50	90	101.81	90	2017	[1611]
YIDKOO	$P2_1$	4.9300	7.480	15.899	90	94.656	90	2018	[1612]
YUPZOB	$P2_12_12_1$	5.521	8.801	30.85	90	90	90	2020	[1602]
YUPZUH	$P2_12_12_1$	5.4469	8.8456	31.639	90	90	90	2020	[1602]
YUQBAQ	$P2_1$	8.8108	5.9955	15.702	90	97.468	90	2020	[1602]
YUQBEU	$P2_12_12_1$	5.5067	8.7964	33.354	90	90	90	2020	[1602]
YUQBIY	$P4_1$	8.906	8.906	46.052	90	90	90	2020	[1601]

CSD Code	Space Group	a / Å	b / Å	c / Å	α / °	β / °	γ / °	Publication Year	Reference
ZIMGIO	$P2_12_12_1$	6.7516	11.948	15.630	90	90	90	2018	[1613,1614]
ZUQXIV	$P2_12_12_1$	5.50	10.4	19.7	90	90	90	2020	[1615]
ZUSLEE	$P2_12_12_1$	7.572	34.424	6.321	90	90	90	1996	[1616]
ZUSLOO	$P2_12_12_1$	9.376	28.353	6.076	90	90	90	1996	[1616]
ZUSLUU	$P2_1$	13.326	6.172	11.885	90	114.90	90	1996	[1616]
ZUSMIJ	$P2_1$	19.838	6.356	7.032	90	90.14	90	1996	[1616]
ZUSMUV	$P2_12_12_1$	7.182	32.302	6.256	90	90	90	1996	[1616]
ZUSNEG	$P2_12_12_1$	10.088	23.117	5.972	90	90	90	1996	[1616]
ZUSNOQ	$P2_12_12_1$	14.901	18.377	5.970	90	90	90	1996	[1616]
ZUSPEI	$P2_12_12_1$	13.659	20.024	6.357	90	90	90	1996	[1616]
ZZZUFW	$P2_12_12$	7.90	7.98	7.89	90	90	90	1939	[1617]

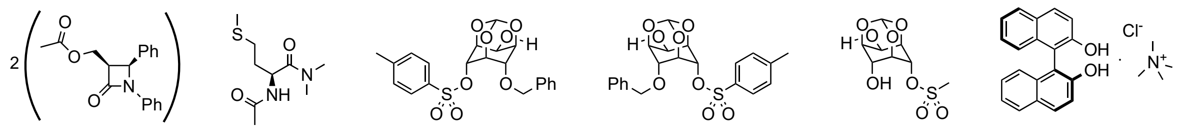

ABAFAL AMETMA AVOROV AVORUB AVOSIQ BICZET

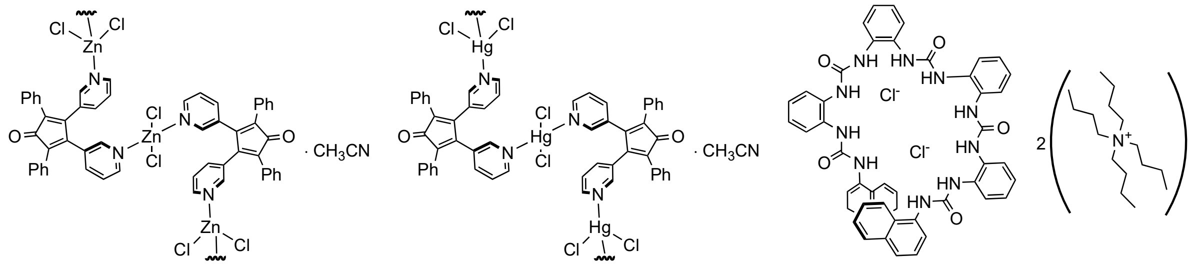

BAXPIA BAXPOG BEPSIA

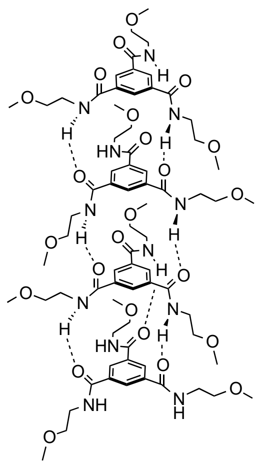

BOHTIB

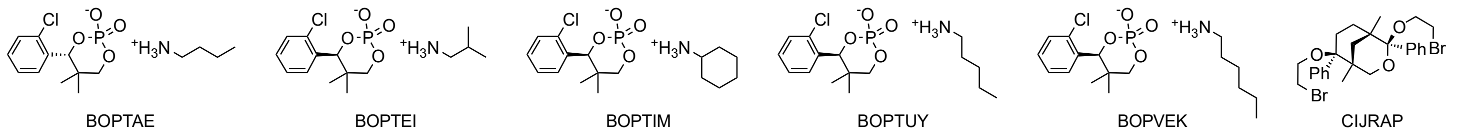

BOPTAE BOPTEI BOPTIM BOPTUY BOPVEK CIJRAP

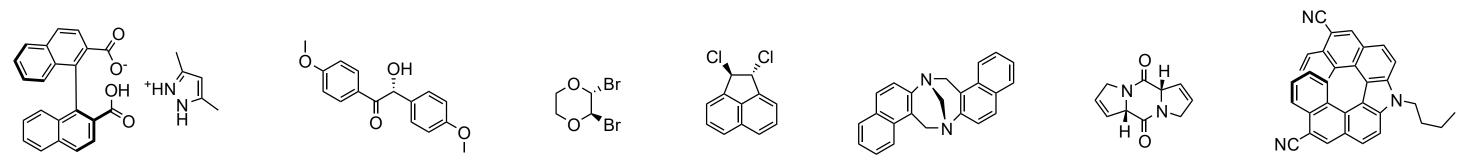

CUQGOK DAHSUA01 DBRDOX DCACNA DEFQAG01 DHPROA DOSBUL

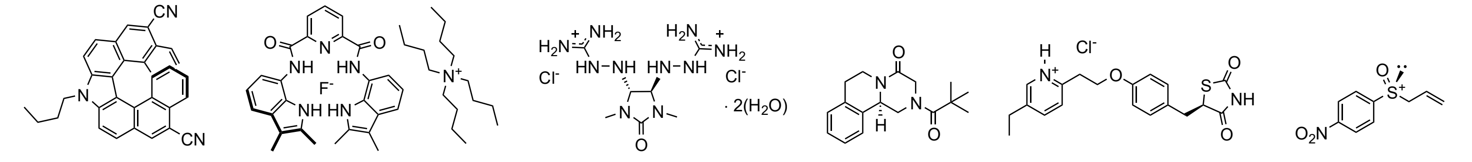

DOSCOG EDIKOS EJUFOE EKIGUD FAYDUF FEDYAR

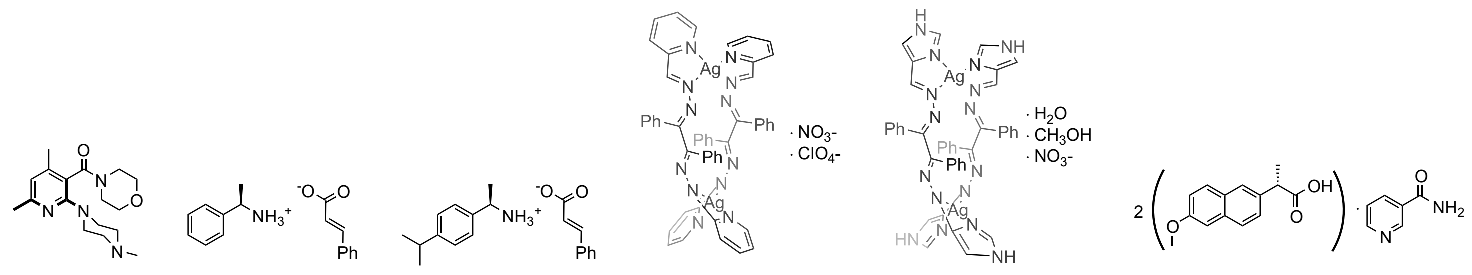

FIQNIE FUSJAE FUSJEI GEJXEZ/GEPROJ GEJXID/GEPRID HEGGAD

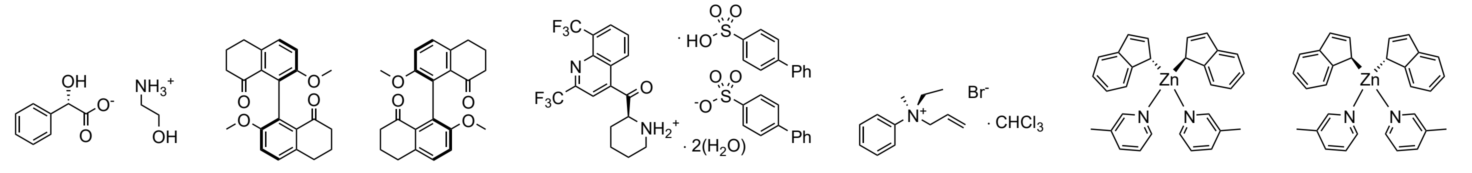

HEZXAM HIRYIS HIRYOY HIWHON HOSKAC HOXZAW HUDNIE

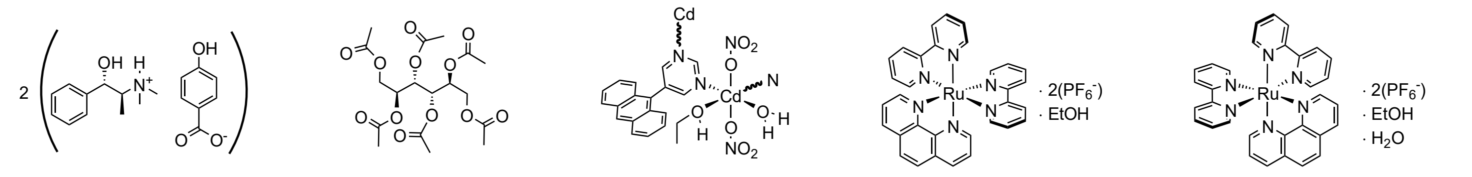

IVUSEY JORJEF02 LABWEQ LECFOP UCUSAN

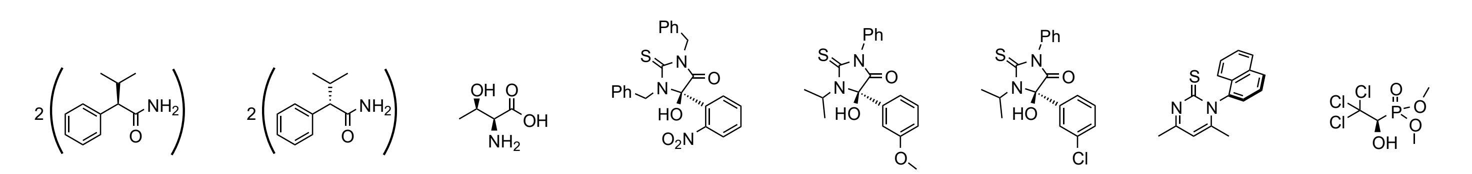

LIDCOT LIDCUZ LTHREO LUTQUP LUTRAW LUTREA MAGSAQ MHCEPO

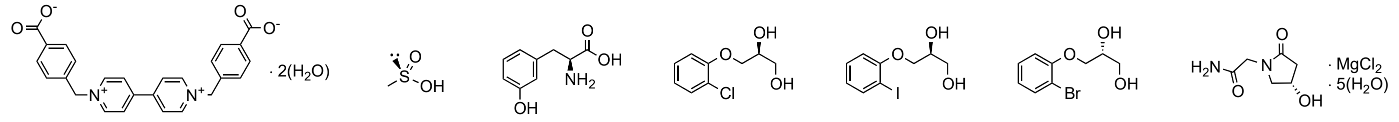

MONQIQ MSUFAC MTYROS MUTBEJ MUTBIN MUTBOT NUMZUT

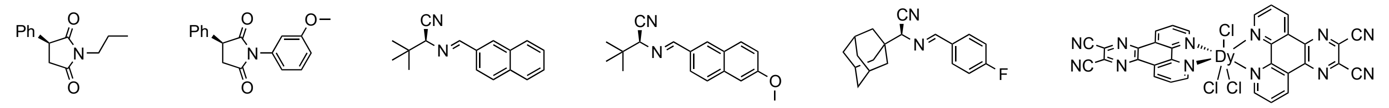

OCODOD OCOFIZ OFUQEO OFUQIS OFUQOY OGUCOJ

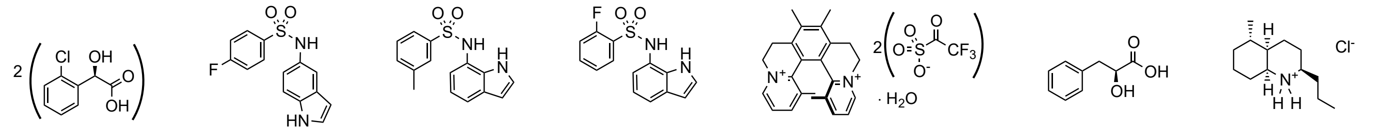

OVIDUT OXIDAD OXIGIO OXIGUA PALNEX PLACTA01 PUMILC02

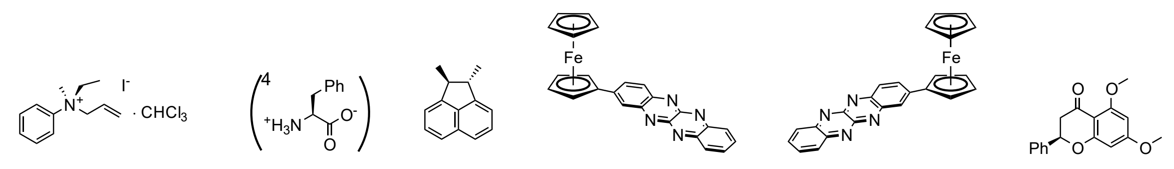

QETJII QQQAUJ05 QQQGXG QUZYES YUQBIY QUZYOC

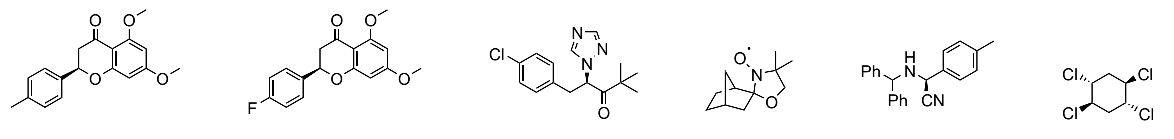

QUZYUI QUZZAP SEYPUH SNBMXZ SUCDUR TCLCYH

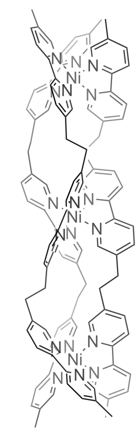

PEGGAJ

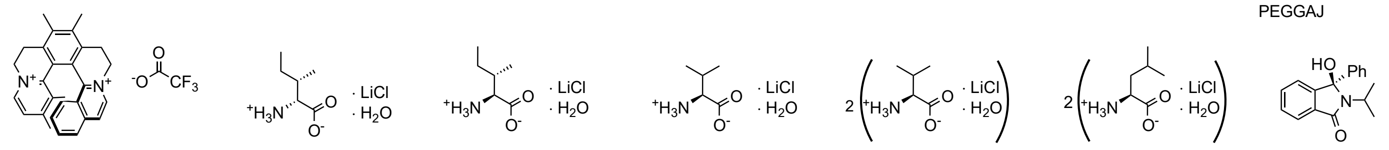

TEXBUV UTEFIL UTEFUX / UTEFUX01 UTEGAE UTEGEI / UTEGEI01 UTEHIN / UTEHIN01 UTEZOL

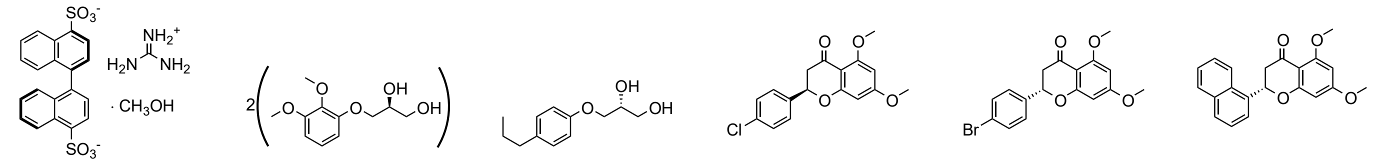

VAWMEM XECTIL YIDKOO YUPZOB YUPZUH YUQBAQ

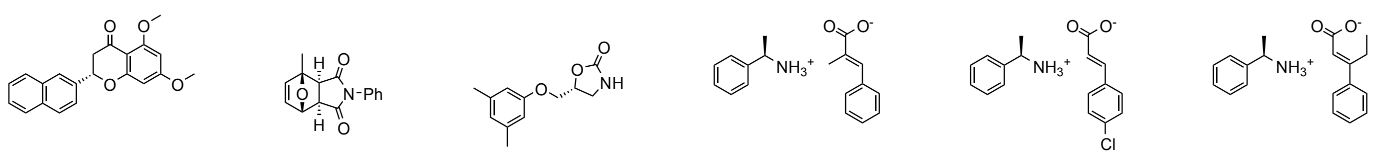

YUQBEU ZIMGIO ZUQXIV ZUSLEE ZUSLOO ZUSLUU

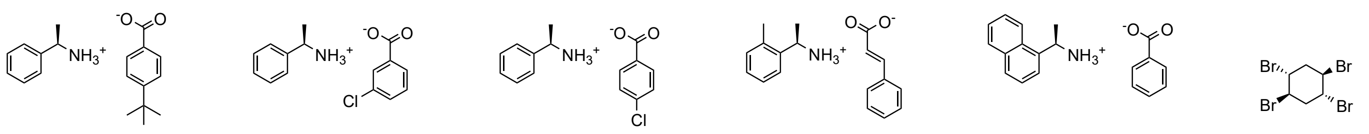

ZUSMIJ ZUSMUV ZUSNEG ZUSNOQ ZUSPEI ZZZUFW

Conglomerate crystals not available in the CSD

Confirmed by crystallography but not in the CSD

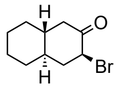

a

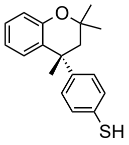

b

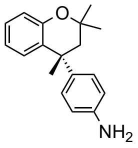

c

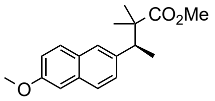

d

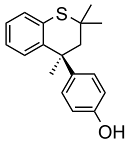

e

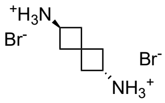

f

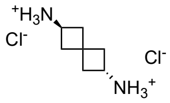

g

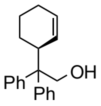

h

Structure	Reference
**a**	1618
**b**	1619
**c**	1619
**d**	1620
**e**	1621
**f**	1622
**g**	1622
**h**	1623

A comprehensive list of additional conglomerates detected by alternative means (IR spectroscopy, optical rotation, and binary phase diagrams) is described by Jacques, Collet, and Wilen.[1624]

Crystals in the CSD which undergo racemic twinning

CSD Code	Space Group	a / Å	b / Å	c / Å	α / °	β / °	γ / °	Publication Year	Reference
ADUQOF	$P2_12_12_1$	7.98	7.219	12.818	90	90	90	2001	[1625]
AJURON	$P2_12_12_1$	6.08	11.792	15.073	90	90	90	2010	[1626]
AQOXOT	$P2_12_12_1$	17.871	25.474	10.069	90	90	90	2003	[1627]
BAKLUX	$P2_12_12_1$	7.445	12.465	16.095	90	90	90	2017	[1628]
BISXUZ	$P2_12_12_1$	10.987	11.76	12.441	90	90	90	2018	[1629]
CICTAK	$P2_12_12_1$	10.207	10.643	15.587	90	90	90	2007	[1630]
HILGOB	$P6_5$	26.79	26.79	5.9921	90	90	120	2018	[1631]
HOFXEG	$P2_1$	10.579	5.3448	12.873	90	90.204	90	2008	[435]
HUKZET	$P2_12_12_1$	6.317	12.442	21.957	90	90	90	2009	[1632]
KIKDIS	$P4_1212$	7.9963	7.9963	25.793	90	90	90	2007	[1633]
KURHUB	$P2_12_12_1$	5.5214	10.298	22.9	90	90	90	2010	[1634]
KUSPAQ	$P2_1$	4.2981	12.995	15.728	90	96.493	90	2010	[1635]
LAPYOS	$P2_12_12_1$	5.0213	15.683	18.637	90	90	90	2012	[1636]
LEXBEY	$P2_1$	7.3454	6.2161	10.797	90	108.14	90	2018	[1637]
LUBFAR	$P2_12_12_1$	6.7174	16.822	21.769	90	90	90	2015	[1638]
LUJHAB	$P2_12_12_1$	9.3393	19.75	9.6197	90	90	90	2015	[1639]
MARBAL	$P2_12_12_1$	5.4961	10.233	22.726	90	90	90	2017	[1640]
MAYPIB	$P2_12_12_1$	8.792	17.037	14.493	90	90	90	1976	[1641]
MERKUS	$P2_1$	5.5702	6.3387	11.793	90	101.67	90	2017	[1642]
NIWTOF	$P2_1$	7.7498	11.179	9.345	90	95.75	90	2019	[1643]
PEQLIJ	$C2$	10.207	6.3761	23.227	90	101.45	90	2018	[1644]
RAJZIO	$P2_1$	5.8136	15.285	11.833	90	94.654	90	2017	[1023]
REBBOQ	$P2_12_12_1$	6.2089	14.639	18.596	90	90	90	2006	[1645]
RULMOC	$P2_12_12_1$	18.169	13.324	14.28	90	90	90	2015	[1646]
TAVFIG	$P2_12_12_1$	7.8186	12.882	13.76	90	90	90	2005	[1647]
TEQZOH	$P2_12_12_1$	5.3885	14.607	21	90	90	90	2017	[1648t]
UCUDAZ	$P2_12_12_1$	5.6623	10.078	16.324	90	90	90	2010	[1649]
UREYOG	$P2_1$	6.0775	8.8106	12.579	90	99.904	90	2011	[1650]
VUGVIE	$P2_1$	10.056	6.172	12.751	90	99.641	90	2015	[1651]
XENSOB	$P2_1$	12.813	7.0224	12.817	90	117.21	90	2017	[1652]
YIRWOM	$P2_12_12_1$	9.0943	10.917	18.066	90	90	90	2008	[1653]

Internal search of the CSD

An internal search of CIF information in the CSD was undertaken using a python script (Made available here: https://github.com/walshm78/CSD_conglomerate_search).

The information in the chemical_enantioexcess_* fields (_chemical_enantioexcess_bulk, _chemical_enantioexcess_bulk_technique, _chemical_enantioexcess_crystal, _chemical_enantioexcess_crystal_technique; as identified in the IUCr Core CIF Dictionary, https://www.iucr.org/__data/iucr/cifdic_html/1/cif_core.dic/index.html) from stored CIF files at the CCDC was extracted using the CSD Python API. CIFs that contained information in this field were identified.

The total output of the search yielded the following entries:

CSD Code	Deposition number	_chemical_enantioexcess_bulk	_chemical_enantioexcess_bulk_technique	_chemical_enantioexcess_crystal	_chemical_enantioexcess_crystal_technique
JUBBAM	1962550	0	N/A	0	N/A
VELQEK	876278	N/A	N/A	0	N/A
VELQIO	884436	N/A	N/A	0	N/A
PEXBEB	905798	N/A	N/A	1	N/A
BEXTAB01	905799	N/A	N/A	0	N/A
BEXTAB	915064	N/A	N/A	0	N/A
KUJCAV	945842	0	N/A	0	N/A
XEFROR	774553	0	N/A	0	N/A
LESCOE	796981	N/A	N/A	0	N/A
SUKQAS	843532	0	N/A	0	N/A
VACNIZ	856484	N/A	N/A	0	N/A
GUHQEG	717504	0	N/A	0	N/A

- Only 12 entries contained one of the _chemical_enantioexcess_* fields.
- Of these 12 entries, only 1 (PEXBEB), has the required values in its CIF to mark it as a potential conglomerate.
- Upon inspection, the PEXBEB crystal was deemed to *not* be a conglomerate crystal.

Therefore, we concluded that even with the ability to access the internal search parameters which are not made publicly available by the CCDC, it is not possible to conduct an automated search for conglomerates due to a lack of metadata recorded within the deposited CIFs.

Conglomerate crystals confirmed by preferential crystallisation

Resolved by Preferential Crystallisation (Entrainment)

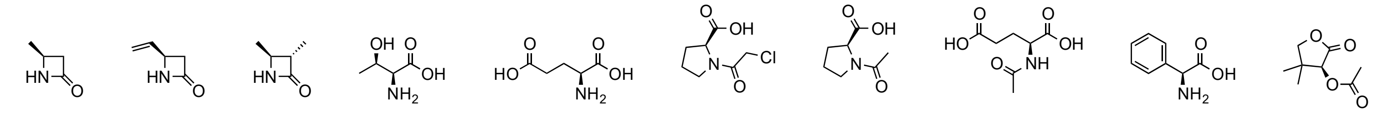

I II III IV V VI VII VIII IX X

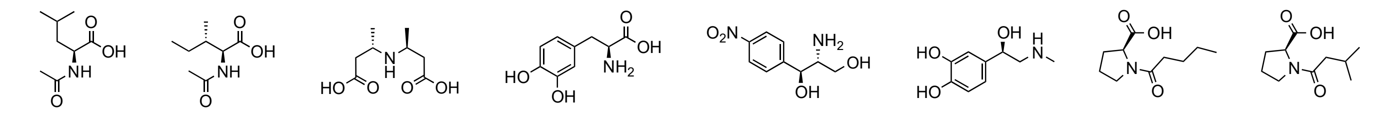

XI XII XIII XIV XV XVI XVII XVIII

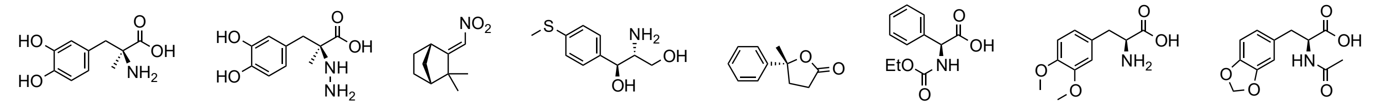

XIX XX XXI XXII XXIII XXIV XXV XXVI

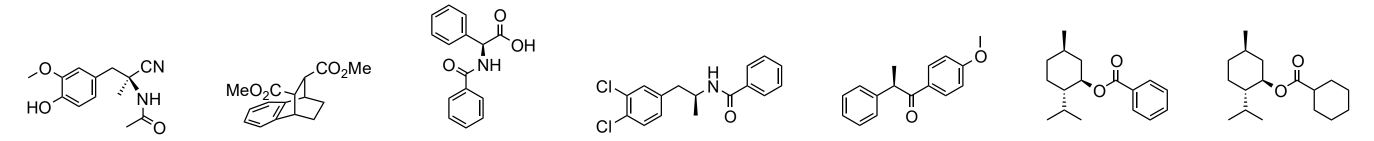

XXVII XXVIII XXIX XXX XXXI XXXII XXXIII

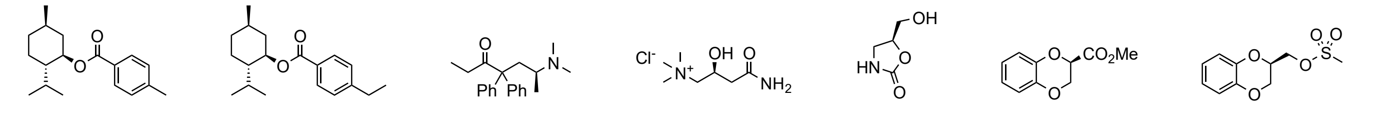

XXXIV XXXV XXXVI XXXVII XXXVIII XXXIX XL

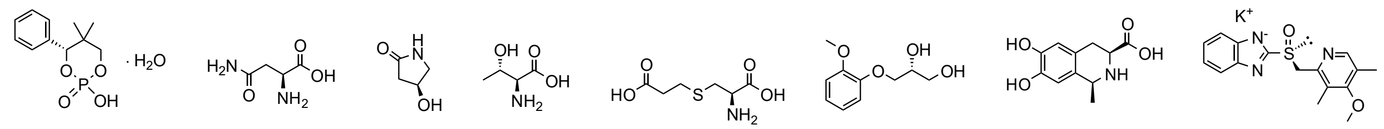

XLI XLII XLIII XLIV XLV XLVI XLVII XLVIII

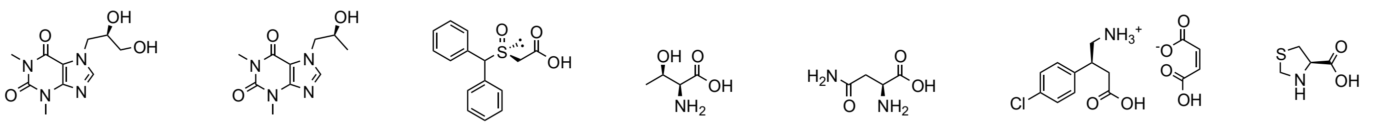

XLIX L LI LII LIII LIV LV

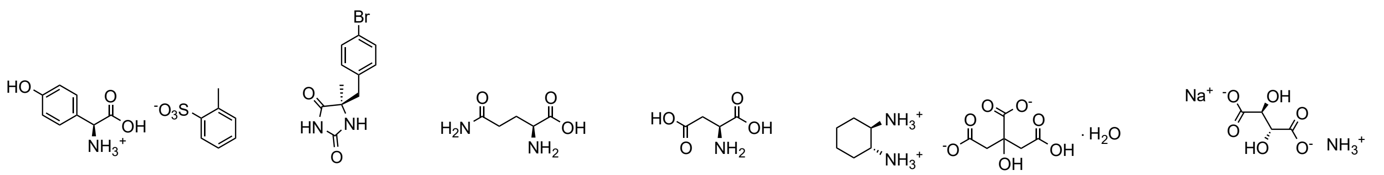

LVI LVII LVIII LIX LX LXI

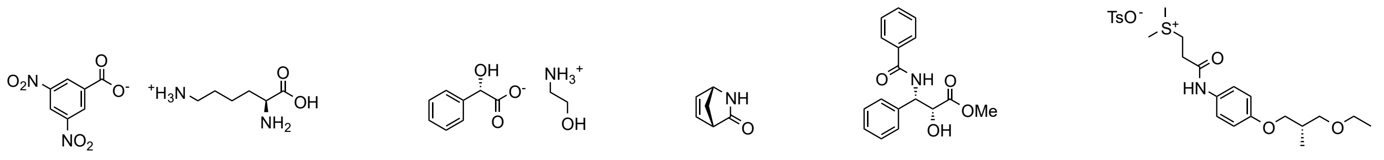

LXII LXIII LXIV LXV LXVI

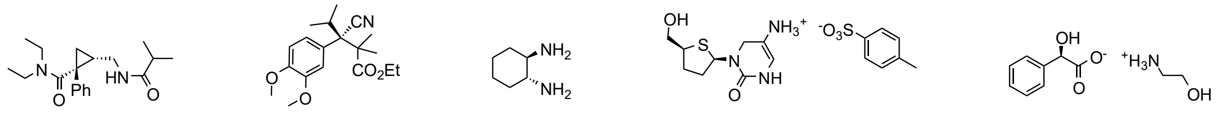

LXVII LXVIII LXIX LXX LXXI

Entry	Reference	Entry	Reference	Entry	Reference
**I**	1654	**XXIV**	1655	**XLVII**	1656
**II**	1654	**XXV**	1657	**XLVIII**	1658
**III**	1654	**XXVI**	1657	**XLIX**	1659
**IV**	1660,1661	**XXVII**	1662	**L**	1663
**V**	1664–1666	**XXVIII**	1667	**LI**	1668
**VI**	1669,1670	**XXIX**	1671	**LII**	1672
**VII**	1670	**XXX**	1673	**LIII**	1666,1674
**VIII**	1675	**XXXI**	1676,1677	**LIV**	1678
**IX**	1679	**XXXII**	1680	**LV**	1681
**X**	1682	**XXXIII**	1680	**LVI**	1683
**XI**	1676,1684	**XXXIV**	1680	**LVII**	1685
**XII**	1684	**XXXV**	1680	**LVIII**	1666
**XIII**	1686	**XXXVI**	1687	**LIX**	1666
**XIV**	1688	**XXXVII**	1689	**LX**	1690
**XV**	1691	**XXXVIII**	1692	**LXI**	1693
**XVI**	1694	**XXXIX**	1695	**LXII**	1696
**XVII**	1669,1670	**XL**	1695	**LXIII**	1566
**XVIII**	1669,1670	**XLI**	1697	**LXIV**	1698
**XIX**	1677	**XLII**	1699	**LXV**	1700
**XX**	1701	**XLIII**	1702	**LXVI**	1703
**XXI**	1704	**XLIV**	1705	**LXVII**	1706
**XXII**	1707	**XLV**	1708	**LXVIII**	1709
**XXIII**	1710	**XLVI**	1711,1712	**LXIX**	1713
				**LXX**	1714,1715
				**LXXI**	1716

Salts and complexes of amino acids which have undergone preferential crystallisation such as: alanine, serine, valine, proline, glutamic acid, leucine, lysine, histidine, phenylglycine, phenylalanine, and tryptophan; are described by Jacques , Collet, Brienne and Wilen[1624,1717] and references therein.

References

(1) Shacklady-Mcatee, D. M.; Dasgupta, S.; Watson, M. P. Nickel(0)-Catalyzed Cyclization of N -Benzoylaminals for Isoindolinone Synthesis. *Org. Lett.* **2011**, *13* (13), 3490–3493. https://doi.org/10.1021/ol201248c.

(2) Huang, J. K.; Wong, Y. C.; Kao, T. T.; Tseng, C. T.; Shia, K. S. Cobalt(II)-Catalyzed Aerobic Oxidation of Terminal-Capped Alkynyl α-Cyano Alkanone Systems. An Oxygen-Mediated Radical Chain Reaction. *J. Org. Chem.* **2016**, *81* (22), 10759–10768. https://doi.org/10.1021/acs.joc.6b01837.

(3) Kise, N.; Hamada, Y.; Sakurai, T. Electroreductive Intermolecular Coupling of Coumarins with Benzophenones: Synthesis of 4-(2-Hydroxyphenyl)-5,5-Diaryl-γ-Butyrolactones, 2-(2,2-Diaryl-2,3-Dihydrobenzofuran-3-Yl)Acetic Acids, and 4-(Diarylmethyl)Coumarins. *J. Org. Chem.* **2016**, *81* (22), 11043–11056. https://doi.org/10.1021/acs.joc.6b02056.

(4) McCullough, K. J.; Ito, T.; Tokuyasu, T.; Masuyama, A.; Nojima, M. The Synthesis and Crystal Structure Analysis of Novel Macrocyclic Peroxides. *Tetrahedron Lett.* **2001**, *42* (32), 5529–5532. https://doi.org/10.1016/S0040-4039(01)01015-2.

(5) Liu, R.; Giordano, L.; Tenaglia, A. Ruthenium-Catalyzed [2+2+2] Cycloaddition of 1,6-Enynes and Unactivated Alkynes: Access to Ring-Fused Cyclohexadienes. *Chem. Asian J.* **2017**, *12* (17), 2245–2257. https://doi.org/10.1002/asia.201700642.

(6) Bruno, G.; Nicolò, F.; Rotondo, A.; Risitano, F.; Grassi, G.; Foti, F. Structure Investigation of Bridgehead Aziridine: Synthesis, Theoretical, and Crystallographic Study of 2,4,6-Triphenyl-1,3-Diazabicyclo[3.1.0]Hex-3-Ene. *Helv. Chim. Acta* **2006**, *89* (2), 190–200. https://doi.org/10.1002/hlca.200690021.

(7) Bentabed-Ababsa, G.; Hamza-Reguig, S.; Derdour, A.; Domingo, L. R.; Sáez, J. A.; Roisnel, T.; Dorcet, V.; Nassar, E.; Mongin, F. Experimental and Theoretical Study of the [3 + 2] Cycloaddition of Carbonyl Ylides with Alkynes. *Org. Biomol. Chem.* **2012**, *10* (42), 8434–8444. https://doi.org/10.1039/c2ob26442k.

(8) Wang, B.; Li, M.; Xu, S.; Song, H.; Wang, B. A General Synthetic Route to Dibenzospiropyrans and Dinaphthospiropyrans from Dibenzofuran and Dinaphthofuran. *Synthesis* **2007**, No. 11, 1643–1648. https://doi.org/10.1055/s-2007-966061.

(9) Wallfisch, B. C.; Belaj, F.; Wentrup, C.; Kappe, C. O.; Kollenz, G. Synthesis and Chemical Reactivity of Methoxycarbonyl-1,3-Dioxinyl(Pivaloyl)Ketene—a Persistent α-Oxoketene. *J. Chem. Soc. Perkin 1* **2002**, *2* (5), 599–605. https://doi.org/10.1039/b111143d.

(10) Yu, C. X.; Lei, S.; Yao, C. S.; Tu, S. J. Ethyl 2-Anilino-4-(2,4-Dichloro-Phen-Yl)-6-Trifluoro-Methyl-3,4-Dihydro-Pyrimidine-5-Carboxyl-Ate. *Acta Cryst. E* **2007**, *63* (9), o3804–o3804. https://doi.org/10.1107/S1600536807037786.

(11) Kumar, B.; Battini, N.; Ahmed, Q. N.; Ali, A.; Gupta, V. K. X-Ray Study of 7a-(2-Chlorophenyl)-7a,8a,9,10,11,12ahexadronaptho[1′,2′:4,5]Furo[3,2-d]Pyrrolo[2,1-b]Oxazole and 2-(4-Fluorophenyl)-2-Hydroxynaptho[2,1-b]Furan-1(2H)-One. *Crystallogr. Reports* **2018**, *63* (3), 382–387. https://doi.org/10.1134/S1063774518030045.

(12) Roux, M. V.; Jiménez, P.; Vacas, A.; Cano, F. H.; Apreda-Rojas, M. del C.; Ros, F. A Compact and Lipophilic Enantiomer Showing a Bilayer Crystal Structure - Free Energy of Spontaneous Resolution of the Racemic Compound into the Crystalline Enantiomers. *Eur. J. Org. Chem.* **2003**, *2003* (11), 2084–2091. https://doi.org/10.1002/ejoc.200200315.

(13) Karad, S. N.; Chung, W. K.; Liu, R. S. Gold-Catalyzed Formal [4π + 2π]-Cycloadditions of Propiolate Derivatives with Unactivated Nitriles. *Chem. Sci.* **2015**, *6* (10), 5964–5968. https://doi.org/10.1039/c5sc01950h.

(14) Kostyanovsky, R. G.; Kostyanovsky, V. R.; Gul'nara, K. K.; Lyssenko, K. A. Asymmetric Three-Coordinated Nitrogen Compounds: Spontaneous Resolution and Absolute Asymmetric Synthesis. *Mendeleev Commun.* **2003**, *13* (3), 111–113. https://doi.org/10.1070/MC2003v013n03ABEH001807.

(15) Chabane, H.; Meth-Cohn, O.; Rees, C. W.; White, A. J. P.; Williams, D. J. Cycloadditions of N=S Functions to Cyclopentadienones. *Tetrahedron Lett.* **2003**, *44* (35), 6709–6711. https://doi.org/10.1016/S0040-4039(03)01646-0.

(16) Rosas, N.; Sharma, P.; Alvarez, C.; Gómez, E.; Gutiérrez, Y.; Méndez, M.; Toscano, R. A.; Maldonado, L. A. A Novel Method for the Synthesis of 5,6-Dihydro-4H-Oxocin-4-Ones: 6-Endo-Dig versus 8-Endo-Dig Cyclizations. *Tetrahedron Lett.* **2003**, *44* (43), 8019–8022. https://doi.org/10.1016/j.tetlet.2003.08.104.

(17) Jefcoate, C. R.; Ghisla, S.; Hemmerich, P. Studies in the Flavin Series. Part XVI. Alkylation and Rearrangement

Reactions of Dihydroalloxazines. *J. Chem. Soc. C Org. Chem.* **1971**, *43* (0), 1689–1694. https://doi.org/10.1039/J39710001689.

(18) Lindner, H. J.; Kitschke, B.; Hafner, K.; Ude, W. Structure of a 1:1 Adduct of 4,6,8-Trimethylazulene and 1-(Diethylamino)Propyne. *Acta Cryst. B* **1980**, *36* (3), 758–759. https://doi.org/10.1107/s0567740880004438.

(19) De Sousa, G. F.; Gatto, C. C.; Resck, I. S.; Deflon, V. M. Synthesis, Spectroscopic Studies and X-Ray Crystal Structures of New Pyrazoline and Pyrazole Derivatives. *J. Chem. Crystallogr.* **2011**, *41* (3), 401–408. https://doi.org/10.1007/s10870-010-9896-2.

(20) Lee, J. H.; Zhang, Y.; Danishefsky, S. J. A Straightforward Route to Functionalized Trans-Diels-Alder Motifs. *J. Am. Chem. Soc.* **2010**, *132* (41), 14330–14333. https://doi.org/10.1021/ja1073855.

(21) Ribelles, P.; Sridharan, V.; Villacampa, M.; Ramos, M. T.; Menéndez, J. C. Diastereoselective, Multicomponent Access to Trans-2-Aryl-4-Arylamino-1,2, 3,4-Tetrahydroquinolines via an AA′BC Sequential Four-Component Reaction and Their Application to 2-Arylquinoline Synthesis. *Org. Biomol. Chem.* **2013**, *11* (4), 569–579. https://doi.org/10.1039/c2ob26754c.

(22) Ramazani, A.; Mahyari, A.; Lashgari, H.; Ślepokura, K.; Lis, T. Silica Nanoparticles as a Highly Efficient Catalyst for the One-Pot Synthesis of 2-Hydroxyacetamide Derivatives from Isocyanides and Electron-Poor Aromatic Aldehydes. *Helv. Chim. Acta* **2011**, *94* (4), 611–622. https://doi.org/10.1002/hlca.201000280.

(23) Wu, Y. C.; Li, H. J.; Liu, L.; Liu, Z.; Wang, D.; Chen, Y. J. Cascade Reaction of β,γ-Unsaturated α-Ketoesters with Phenols in Trityl Chloride/TFA System. Highly Selective Synthesis of 4-Aryl-2H-Chromenes and Their Applications. *Org. Biomol. Chem.* **2011**, *9* (8), 2868–2877. https://doi.org/10.1039/c0ob01143f.

(24) Khera, R. A.; Ahmad, R.; Ullah, I.; Abid, O. U. R.; Fatunsin, O.; Sher, M.; Villinger, A.; Langer, P. Cyclization vs. Elimination Reactions of 5-Aryl-5-Hydroxy 1,3-Diones: One-Pot Synthesis of 2-Aryl-2,3-Dihydro-4H-Pyran-4-Ones. *Helv. Chim. Acta* **2010**, *93* (9), 1705–1715. https://doi.org/10.1002/hlca.201000015.

(25) Arulsamy, N.; Bohle, D. S.; Butt, J. A.; Irvine, G. J.; Jordan, P. A.; Sagan, E. Interrelationships between Conformational Dynamics and the Redox Chemistry of S-Nitrosothiols. *J. Am. Chem. Soc.* **1999**, *121* (30), 7115–7123. https://doi.org/10.1021/ja9901314.

(26) Zhang, Y.; Liu, F.; Zhang, J. Catalytic Regioselective Control in the Diastereoselective 1,3-Dipolar Cycloaddition Reactions of 1-(1-Alkynyl)Cyclopropyl Ketones with Nitrones. *Chem. Eur. J.* **2010**, *16* (21), 6146–6150. https://doi.org/10.1002/chem.200903342.

(27) Chen, L.; Xing, H.; Zhang, H.; Jiang, Z. X.; Yang, Z. Copper-Catalyzed Intermolecular Chloroazidation of α,β-Unsaturated Amides. *Org. Biomol. Chem.* **2016**, *14* (31), 7463–7467. https://doi.org/10.1039/c6ob01352j.

(28) McNulty, J.; McLeod, D. Discovery of an Acid-Promoted [3+2] Cyclodimerization of 3-Vinylindoles and the Development of a General Lewis Acid Catalyzed Process. *Synlett* **2011**, No. 5, 717–721. https://doi.org/10.1055/s-0030-1259688.

(29) Ohsawa, A.; Wada, I.; Igeta, H.; Akimoto, T.; Tsuji, A.; Iitaka, Y. 1,3-Dipolar Cycloaddition of Pyridazinium N-Ylides with Perhalocycloalkenes. *Tetrahedron Lett.* **1978**, *19* (43), 4121–4124. https://doi.org/10.1016/S0040-4039(01)95159-7.

(30) Wang, R.; Chen, C.; Duesler, E.; Mariano, P. S.; Yoon, U. C. β-Lactam-Forming Photochemical Reactions of N-Trimethylsilylmethyl- and N-Tributylstannylmethyl-Substituted α-Ketoamides. *J. Org. Chem.* **2004**, *69* (4), 1215–1220. https://doi.org/10.1021/jo030343q.

(31) Amharar, Y.; Petit, S.; Sanselme, M.; Cartigny, Y.; Petit, M. N.; Coquerel, G. Crystal Structures, Dehydration Mechanism, and Chiral Discrimination in the Solid State of a Hydantoin Derivative. *Cryst. Growth Des.* **2011**, *11* (6), 2453–2462. https://doi.org/10.1021/cg200243y.

(32) Bai, J.; Wang, P.; Cao, W.; Chen, X. Tautomer-Selective Derivatives of Enolate, Ketone and Enaminone by Addition Reaction of Picolyl-Type Anions with Nitriles. *J. Mol. Struct.* **2017**, *1128*, 645–652. https://doi.org/10.1016/j.molstruc.2016.09.038.

(33) Rybalova, T. V.; Gatilov, Y. V.; Zonov, Y. V.; Karpov, V. M. Supramolecular Architecture of Crystals of Perfluorinated 3-Alkylphthalides. *J. Struct. Chem.* **2016**, *57* (4), 777–783. https://doi.org/10.1134/S0022476616040223.

(34) Mei, H.; Han, J.; Li, G.; Pan, Y. KOH-Catalyzed Highly Efficient Aminohalogenation of β-Nitrostyrenes with t-Butyl N,N-Dichlorocarbamate as Nitrogen/Halogen Source. *RSC Adv.* **2011**, *1* (3), 429–433. https://doi.org/10.1039/c1ra00174d.

(35) Giuffredi, G. T.; Bernet, B.; Gouverneur, V. De Novo Synthesis of Racemic 4-Deoxy-4,4-Difluoro- and 2,4-Dideoxy-2,4,4- Trifluorohexosides. *Eur. J. Org. Chem.* **2011**, *2011* (20–21), 3825–3836.

https://doi.org/10.1002/ejoc.201100565.

(36) Shiraki, S.; Natarajan, A.; Garcia-Garibay, M. A. The Synthesis and Stereospecific Solid-State Photodecarbonylation of Hexasubstituted Meso- and d,l-Ketones. *Photochem. Photobiol. Sci.* **2011**, *10* (9), 1480–1487. https://doi.org/10.1039/c1pp05080j.

(37) El Asri, Z.; Génisson, Y.; Guillen, F.; Baslé, O.; Isambert, N.; Del Mar Sanchez Duque, M.; Ladeira, S.; Rodriguez, J.; Constantieux, T.; Plaquevent, J. C. Multicomponent Reactions in Ionic Liquids: Convenient and Ecocompatible Access to the 2,6-DABCO Core. *Green Chem.* **2011**, *13* (9), 2549–2552. https://doi.org/10.1039/c1gc15635g.

(38) Quintanilla, G.; Usarralde, Á.; Pérez, I.; Gargiulo, M. L.; Yakupoglu, G.; Martín, A.; Barba, F. Anodic Oxidation of Caffeine and Theophylline in Glacial Acetic Acid. *ChemistrySelect* **2016**, *1* (3), 414–416. https://doi.org/10.1002/slct.201500047.

(39) Asiri, A. M.; Al-Youbi, A. O.; Faidallah, H. M.; Ng, S. W. 5-Hydroxy-3-Phenyl-5-Trifluoromethyl-4,5-Dihydro-1H-Pyrazole. *Acta Cryst. E* **2011**, *67* (9), o2442–o2442. https://doi.org/10.1107/S160053681103368X.

(40) Lyssenko, K. A.; Golovanov, D. G.; Kravchenko, A. N.; Chikunov, I. E.; Lebedev, O. V.; Makhova, N. N. New Conglomerate in the Series of Glycolurils. *Mendeleev Commun.* **2004**, *14* (3), 105–107. https://doi.org/10.1070/MC2004v014n03ABEH001886.

(41) Therkelsen, F. D.; Rottländer, M.; Thorup, N.; Pedersen, E. B. 4-Metalated Condensed Pyrimidines: Their Preparation and Reaction with Aldehydes under Barbier-Type Conditions. *Org. Lett.* **2004**, *6* (12), 1991–1994. https://doi.org/10.1021/ol049432v.

(42) He, L. 2,2-Diethyl 4-Methyl 5-(4-Nitrophenyl)-4-Phenylpyrrolidine-2,2,4- Tricarboxylate. *Acta Cryst. E* **2011**, *67* (10), o2593–o2593. https://doi.org/10.1107/S1600536811036038.

(43) Choi, H.; Shim, Y. S.; Lee, S. C.; Kang, S. K.; Sung, C. K. 1-(2-Hydroxy-2-Phenylethyl)-3-(4-Meth-Oxyphenyl)Urea. *Acta Cryst. E* **2011**, *67* (10), o2632–o2632. https://doi.org/10.1107/S1600536811036464.

(44) Wang, L.; Lu, W.; Yang, Y.; Zhu, Y. 4a-Hydroxy-9-(4-Hydroxyphenyl)-4,4a,5,6,9,9a-Hexahydro-3H-Xanthene-1,8(2H, 7H)-Dione. *Acta Cryst. E* **2011**, *67* (10), o2751–o2751. https://doi.org/10.1107/S1600536811038335.

(45) Sar, A.; Lindeman, S.; Donaldson, W. A. De Novo Synthesis of Polyhydroxyl Aminocyclohexanes. *Org. Biomol. Chem.* **2010**, *8* (17), 3908–3917. https://doi.org/10.1039/c004730a.

(46) Chandrakala, P. S.; Katz, A. K.; Carrell, H. L.; Sailaja, P. R.; Podile, A. R.; Nangia, A.; Desiraju, G. R. Synthesis, X-Ray Crystal Structures and Biological Evaluation of Some Mono- and Bi-Cyclic 1,3-Diazetidin-2-Ones: Non-Natural β-Lactam Analogues. *J. Chem. Soc. Perkin Trans. 1* **1998**, No. 16, 2597–2608. https://doi.org/10.1039/a802438c.

(47) Mohan Raj, R.; Balasubramanian, K. K.; Easwaramoorthy, D. Diels-Alder Trapping of in Situ Generated Dienes from 3,4-Dihydro-2H-Pyran with p-Quinone Catalysed by p-Toluenesulfonic Acid. *Org. Biomol. Chem.* **2017**, *15* (5), 1115–1121. https://doi.org/10.1039/C6OB02006B.

(48) Semmelhack, M. F.; Sarpong, R.; Bergman, J.; Ho, D. M. Evaluation of Alkene Isomerization as a Trigger for Enediyne Activation. *Tetrahedron Lett.* **2002**, *43* (4), 541–544. https://doi.org/10.1016/S0040-4039(01)02139-6.

(49) Brambilla, R.; Friary, R.; Ganguly, A.; Puar, M. S.; Sunday, B. R.; Wright, J. J.; Onan, K. D.; Mcphail, A. T. Intramolecular Cycloadditions of Nitrones Joined by Amides to Olefins. *Tetrahedron* **1981**, *37* (21), 3615–3625. https://doi.org/10.1016/S0040-4020(01)98890-8.

(50) Fernández, T.; Suárez, D.; Sordo, J. A.; Monnat, F.; Roversi, E.; De Castro, A. E.; Schenk, K.; Vogel, P. Competition between Hetero-Diels-Alder and Cheletropic Addition of Sulfur Dioxide. Theoretical and Experimental Substituent Effects on the Relative Stability of 3,6-Dihydro-1,2-Oxathiin-2-Oxides (Sultines) and 2,5-Dihydrothiophene-1,1-Dioxides (Sulfolene. *J. Org. Chem.* **1998**, *63* (25), 9490–9499. https://doi.org/10.1021/jo981679g.

(51) Luo, Y. C.; Ma, H.; Hu, X. Q.; Xu, P. F. Sc(OTf)3 Catalyzed [4 + 2]-Annulation Reaction between Electron-Rich Phenols and Donor-Acceptor Cyclopropanes: Synthesis of Polysubstituted Dihydronaphthols. *J. Org. Chem.* **2017**, *82* (2), 1013–1023. https://doi.org/10.1021/acs.joc.6b02566.

(52) Pfaendler, H. R.; Gosteli, J.; Woodward, R. B.; Ribs, G. Structure, Reactivity, and Biological Activity of Strained Bicyclic β-Lactams. *J. Am. Chem. Soc.* **1981**, *103* (15), 4526–4531. https://doi.org/10.1021/ja00405a039.

(53) Zheng, D.; Li, S.; Luo, Y.; Wu, J. An Efficient Route to Tetrahydroindeno[2,1-b]Pyrroles via a Base-Promoted Reaction of (E)-2-Alkynylphenylchalcone with 2-Isocyanoacetate. *Org. Lett.* **2011**, *13* (24), 6402–6405. https://doi.org/10.1021/ol202708f.

(54) Kraus, W.; Patzelt, H.; Sadlo, H.; Sawitzki, G.; Schwinger, G. Darstellung Und Röntgenstrukturanalyse von

Bicyclo-[4.2.1]Non-3-en-2-on-Derivaten. *Liebigs Ann. der Chemie* **1981**, *1981* (10), 1826–1837. https://doi.org/10.1002/jlac.198119811010.

(55) Fleming, F. F.; Gudipati, V.; Steward, O. W. ω-Halonitriles: Domino Cyclizations to Oxa- and Carbocyclic Nitriles. *J. Org. Chem.* **2003**, *68* (10), 3943–3946. https://doi.org/10.1021/jo026874g.

(56) Ganesan, M.; Muraleedharan, K. M. Oxanorbornane-Based Amphiphilic Systems: Design, Synthesis and Material Properties. *RSC Adv.* **2012**, *2* (10), 4048–4051. https://doi.org/10.1039/c2ra01198k.

(57) Leskinen, M. V.; Yip, K. T.; Valkonen, A.; Pihko, P. M. Palladium-Catalyzed Dehydrogenative β′-Functionalization of β-Keto Esters with Indoles at Room Temperature. *J. Am. Chem. Soc.* **2012**, *134* (13), 5750–5753. https://doi.org/10.1021/ja300684r.

(58) Kummeter, M.; Kazmaier, U. Synthesis of Polyhydroxylated Aminocyclopentanes. *Eur. J. Org. Chem.* **2003**, *2003* (17), 3325–3329. https://doi.org/10.1002/ejoc.200200703.

(59) Silvestri, A. P.; Dawson, P. E. Base-Catalyzed Diastereoselective Trimerization of Trifluoroacetone. *Org. Biomol. Chem.* **2017**, *15* (24), 5131–5134. https://doi.org/10.1039/c7ob01094j.

(60) Ammon, H. L.; Mazzocchi, P. H.; Liu, L.; Colicelli, E. C.; Doherty, R.; Stewart, J. M. The Structures of Five Bicyclic Lactams: 2-Benzyl-3-Oxo-2-Azabicyclo[2.2.1]Hept-6-Exo-Yl p-Toluenesulfonate, 2-Benzyl-6-Exo-Phenoxy-2-Azabicyclo[2.2.1]Heptan-3-One, 2-Benzyl-6-Exo-(2-Iodo-4-Methylphenoxy)-2-Azabicyclo[2.2.1]Heptan-3-One, 2-Benzyl-6-Exo-(2. *Acta Cryst. B* **1982**, *38* (2), 540–547. https://doi.org/10.1107/s0567740882003343.

(61) Mehta, G.; Sambasxva Rao, K.; Chander Suri, S.; Stanley Cameron, T.; Chan, C. Cubanes Uncaged: Novel Garbonium Ion Rearrangements of Pentacyclo-[4.3.0.02,5.03,8.04,7]Nonan-9-Ones (Homocubanones) to Bicyclo[3.2.1]Octa-2,6-Diene and Tetracyclo[3.3.0.02,6.03,8]Octane Ring Systems. *J. Chem. Soc. - Ser. Chem. Commun.* **1980**, *8* (14), 650–652. https://doi.org/10.1039/C39800000650.

(62) Sun, Z. X.; Cheng, Y. N-Heterocyclic Carbene-Catalyzed Cascade Annulation Reaction of o-Vinylarylaldehydes with Nitrosoarenes: One-Step Assembly of Functionalized 2,3-Benzoxazin-4-Ones. *Org. Biomol. Chem.* **2012**, *10* (20), 4088–4094. https://doi.org/10.1039/c2ob25137j.

(63) Barluenga, J.; Sigüeiro, R.; Vicente, R.; Ballesteros, A.; Tomás, M.; Rodríguez, M. A. Gold-Catalyzed Functionalization of Unactivated C(Sp 3)-H Bonds by Hydride Transfer Facilitated by Alkynylspirocyclopropanes. *Angew. Chem. Int. Ed.* **2012**, *51* (41), 10377–10381. https://doi.org/10.1002/anie.201205051.

(64) Rubin, B.; Danoff, T.; Brooks, L. 10α-Tert-Butyl-3,3aα,4,5,6,6aα,7,8,9,10-Decahydro-7α-Methyl-1H-Naphtho[1,8a-c]Furan-1,8-Dione. *Acta Cryst. B* **1982**, *38* (3), 1033–1035. https://doi.org/10.1107/s0567740882004877.

(65) Han, X.; Li, H.; Hughes, R. P.; Wu, J. Gallium(III)-Catalyzed Three-Component (4+3) Cycloaddition Reactions. *Angew. Chem. Int. Ed.* **2012**, *51* (41), 10390–10393. https://doi.org/10.1002/anie.201205238.

(66) Oberhänsli, W. E. Die Spontane Spaltung von 3,3-Diäthyl-5-methylpiperidin-2,4-dion(Methyprylon) in Die Optischen Isomeren. Eine Rötgenstrukturanalyse Eines Isodimorphen Systems von Mischkristallen. *Helv. Chim. Acta* **1982**, *65* (3), 924–933. https://doi.org/10.1002/hlca.19820650325.

(67) Parrish, J. P.; Trzupek, J. D.; Hughes, T. V.; Hwang, I.; Boger, D. L. Synthesis and Evaluation of N-Aryl and N-Alkenyl CBI Derivatives. *Bioorganic Med. Chem.* **2004**, *12* (22), 5845–5856. https://doi.org/10.1016/j.bmc.2004.08.032.

(68) Zhao, H.; Li, Y. H.; Wang, X. Sen; Qu, Z. R.; Wang, L. Z.; Xiong, R. G.; Abrahams, B. F.; Xue, Z. Noncentrosymmetric Organic Solids with Very Strong Harmonic Generation Response. *Chem. Eur. J.* **2004**, *10* (10), 2386–2390. https://doi.org/10.1002/chem.200305425.

(69) Mąkosza, M.; Nizamov, S.; Urbańczyk-Lipkowska, Z. Reactions of Chloromethyl Aryl Sulfones Carbanions with Anthraquinone Derivatives. *Tetrahedron* **1998**, *54* (22), 6147–6158. https://doi.org/10.1016/S0040-4020(98)00307-X.

(70) Ji, X.; Zhou, Y.; Wang, J.; Zhao, L.; Jiang, H.; Liu, H. Au(I)/Ag(I)-Catalyzed Cascade Approach for the Synthesis of Benzo[4,5]Imidazo[1,2-c]Pyrrolo[1,2-a]Quinazolinones. *J. Org. Chem.* **2013**, *78* (9), 4312–4318. https://doi.org/10.1021/jo400228g.

(71) Koch, O.; Edelmann, F.; Lubke, B.; Behrens, U. Übergangsmetall-Fulven-Komplexe, XX. Carbonylmetallkomplexe Mit Dimeren Fulvenliganden. *Chem. Ber.* **1982**, *115* (9), 3049–3062. https://doi.org/10.1002/cber.19821150911.

(72) Xu, D.; Kaiser, F.; Li, H.; Reich, R. M.; Guo, H.; Kühn, F. E. Highly Selective AlCl 3 Initiated Intramolecular α-Alkylation of α,β-Unsaturated Lactams and Lactones. *Org. Biomol. Chem.* **2019**, *17* (1), 49–52. https://doi.org/10.1039/c8ob02961j.

(73) Li, M.; Wang, J. H.; Li, W.; Wen, L. R. Metal-Free Direct Construction of 2-(Oxazol-5-Yl)Phenols from N-Phenoxyamides and Alkynylbenziodoxolones via Sequential [3,3]-Rearrangement/Cyclization. *Org. Lett.* **2018**, *20* (23), 7694–7698. https://doi.org/10.1021/acs.orglett.8b03427.

(74) Chen, S. J.; Zhu, H.; Zhang, M. M.; Xu, W. W.; Wang, Y. C.; Zhang, Z. F. Crystal Structure of 1-Benzyl-3-Cyano-6-Phenyl-1,2-Dihydropyridine, C 19 H 16 N 2. *Zeitschrift fur Krist. New Cryst. Struct.* **2019**, *234* (3), 519–520. https://doi.org/10.1515/ncrs-2018-0516.

(75) Quan, Y.; Zhang, J.; Xie, Z. Three-Component [2+2+1] Cross-Cyclotrimerization of Carboryne, Unactivated Alkene, and Trimethylsilylalkyne Co-Mediated by Zr and Ni. *J. Am. Chem. Soc.* **2013**, *135* (50), 18742–18745. https://doi.org/10.1021/ja410233e.

(76) Wonneberger, P.; König, N.; Kraft, F. B.; Sárosi, M. B.; Hey-Hawkins, E. Access to 1-Phospha-2-Azanorbornenes by Phospha-Aza-Diels–Alder Reactions. *Angew. Chem. Int. Ed.* **2019**, *58* (10), 3208–3211. https://doi.org/10.1002/anie.201811673.

(77) Rigo, M.; Habraken, E. R. M.; Bhattacharyya, K.; Weber, M.; Ehlers, A. W.; Mézailles, N.; Slootweg, J. C.; Müller, C. Phosphinine-Based Ligands in Gold-Catalyzed Reactions. *Chem. Eur. J.* **2019**, *25* (37), 8769–8779. https://doi.org/10.1002/chem.201900938.

(78) Kundu, A.; Pathak, S.; Debnath, K.; Pramanik, A. Facile Synthesis of 3H,3′H-Spiro[Benzofuran-2,1′-Isoindole]-3,3′-Diones Using Monobromomalononitrile (MBM) as an Efficient Organo-Brominating Agent. *Tetrahedron Lett.* **2014**, *55* (29), 3960–3968. https://doi.org/10.1016/j.tetlet.2014.04.027.

(79) Lang, X. D.; He, L. N. Integration of Co2 Reduction with Subsequent Carbonylation: Towards Extending Chemical Utilization of Co2. *ChemSusChem* **2018**, *11* (13), 2062-2067. https://doi.org/10.1002/cssc.201800902.

(80) Clevers, S.; Rougeot, C.; Simon, F.; Sanselme, M.; Dupray, V.; Coquerel, G. Detection of Order-Disorder Transition in Organic Solids by Using Temperature Resolved Second Harmonic Generation (TR-SHG). *J. Mol. Struct.* **2014**, *1078*, 61–67. https://doi.org/10.1016/j.molstruc.2014.04.007.

(81) Sun, K.; Si, Y. F.; Chen, X. L.; Lv, Q. Y.; Jiang, N.; Wang, S. S.; Peng, Y. Y.; Qu, L. B.; Yu, B. Silver-Catalyzed Radical Cascade Cyclization of Unactivated Alkenes towards Cyclopenta[c]Quinolines. *Adv. Synth. Catal.* **2019**, *361* (19), 4483–4488. https://doi.org/10.1002/adsc.201900691.

(82) Wei, W.; Wen, J.; Yang, D.; Guo, M.; Tian, L.; You, J.; Wang, H. Copper-Catalyzed Cyanoalkylarylation of Activated Alkenes with AIBN: A Convenient and Efficient Approach to Cyano-Containing Oxindoles. *RSC Adv.* **2014**, *4* (89), 48535–48538. https://doi.org/10.1039/c4ra09022e.

(83) Govindaraj, J.; Raja, R.; Suresh, M.; Raghunathan, R.; Subbiahpandi, A. Crystal Structures of Methyl 3-Phenyl-4,5-Dihydro-1H,3H-Benzo[4,5]Imidazo[2,1-c][1,4]Oxazepine-4-Carboxylate and Methyl 1-Methyl-3-Phenyl-4,5-Dihydro-1H,3H-Benzo[4,5]Imidazo[2,1-c][1,4]Oxazepine-4-Carboxylate. *Acta Cryst. E* **2014**, *70* (11), 316–318. https://doi.org/10.1107/S1600536814021655.

(84) Paudler, W. W.; Mahaffey, R. L.; Atwood, J. L. Novel Rearrangement of a[2.2](2,5)Pyrrolophane. *J. Org. Chem.* **1979**, *44* (14), 2498–2499. https://doi.org/10.1021/jo01328a036.

(85) Bexrud, J. A.; Eisenberger, P.; Leitch, D. C.; Payne, P. R.; Schafer, L. L. Selective C-H Activation α to Primary Amines. Bridging Metallaaziridines for Catalytic, Intramolecular α-Alkylation. *J. Am. Chem. Soc.* **2009**, *131* (6), 2116–2118. https://doi.org/10.1021/ja808862w.

(86) Wang, Z. X.; Lia, B.; Xing, Z.; Chen, L. Z.; Han, G. F. Synthesis of Novel 9,9-Dimethyl-8,12-Dihydro-9H-Chromeno[3,2-e] [1,2,4] Triazolo[1,5-c]Pyrimidin-11(10H)-One Derivatives. *J. Chem. Res.* **2014**, *38* (8), 480–485. https://doi.org/10.3184/174751914X14053476598397.

(87) Ponnuswamy, S.; Murugadoss, R.; Jeyaraman, R.; Thiruvalluvar, A.; Parthasarathy, V. Stereochemistry of N-Acyltetrahydro-1,5-Benzodiazepines Using NMR Spectra, X-Ray Crystallography and Semiempirical MO Calculations. *Indian J. Chem. - Sect. B Org. Med. Chem.* **2006**, *45* (9), 2059–2070.

(88) Ravichandran, K.; Sathiyaraj, K.; Ilango, S. S.; Ponnuswamy, S.; Ponnuswamy, M. N. 2,2,4-Trimethyl-5-(4-Tolyl-Sulfon-Yl)-2,3,4,5-Tetra-Hydro-1H-1, 5-Benzo-Diazepine. *Acta Cryst. E* **2009**, *65* (10), o2363–o2364. https://doi.org/10.1107/S1600536809034837.

(89) He, L. Diethyl 1-Acetyl-4′-(4-Chloro-Phen-Yl)-5′-(4-Nitro-Phen-Yl)-2- Oxospiro-[Indoline-3,3′-Pyrrolidine]-2′,2′-Dicarboxyl-Ate. *Acta Cryst. E* **2009**, *65* (10), o2388–o2388. https://doi.org/10.1107/S160053680903551X.

(90) Siegwarth, J.; Bornhöft, J.; Näther, C.; Herges, R. Chiral Crystallization: Freezing a Rapid Cope Rearrangement in the Solid State. *Org. Lett.* **2009**, *11* (15), 3450–3452. https://doi.org/10.1021/ol901326p.

(91) Okada, Y.; Takebayashi, T.; Hashimoto, M.; Kasuga, S.; Sato, S.; Tamura, C. Formation of Optically Active Compounds under Achiral Synthetic Conditions. *J. Chem. Soc. Chem. Commun.* **1983**, No. 14, 784–785.

https://doi.org/10.1039/c39830000784.

(92) Okada, Y.; Takebayashi, T.; Sato, S. Asymmetric Transformation: III: Crystal Properties and Structures of a 1,4-Benzodiazepinooxa-Zole Derivative. *Chem. Pharm. Bull.* **1989**, *37* (1), 5–8. https://doi.org/10.1248/cpb.37.5.

(93) Dange, N. S.; Stepherson, J. R.; Ayala, C. E.; Fronczek, F. R.; Kartika, R. Cooperative Benzylic-Oxyallylic Stabilized Cations: Regioselective Construction of α-Quaternary Centers in Ketone-Derived Compounds. *Chem. Sci.* **2015**, *6* (11), 6312–6319. https://doi.org/10.1039/c5sc01914a.

(94) Al-Jalal, N. A.; Ibrahim, M. R.; Al-Awadi, N. A.; Elnagdi, M. H.; Ibrahim, Y. A. Photochemistry of Benzotriazoles: Generation of 1,3-Diradicals and Intermolecular Cycloaddition as a New Route toward Indoles and Dihydropyrrolo[3,4-b]Indoles. *Molecules* **2014**, *19* (12), 20695–20708. https://doi.org/10.3390/molecules191220695.

(95) Ge, S. Q.; Yang, X.; Wu, B.; Xia, M. Base-Dependent Cascade Synthesis of Novel Pyrano[3,2-c]Coumarin Derivatives from Baylis-Hillman Bromide. *Synth. Commun.* **2010**, *40* (7), 1009–1021. https://doi.org/10.1080/00397910903029933.

(96) Khalaji, A. D.; Gholinejad, M.; Rad, S. M.; Grivani, G.; Fejfarova, K.; Dusek, M. Synthesis, Characterization, Crystal Structure and Theoretical Studies of New Chiral Schiff Base (E)-4-Hydroxy[(1-Phenylethyl)Iminomethyl]Benzyne. *Res. Chem. Intermed.* **2015**, *41* (3), 1635–1645. https://doi.org/10.1007/s11164-013-1299-5.

(97) Murai, T.; Suzuki, A.; Kato, S. Aldol-Type Condensation Reactions of Lithium Eneselenolates Generated from Selenoamides with Aldehydes. *J. Chem. Soc. Perkin 1* **2001**, *1* (20), 2711–2716. https://doi.org/10.1039/b100198l.

(98) Fujise, Y.; Morishima, T.; Namiwa, K.; Shiokawa, T.; Fukazawa, Y.; Itô, S. Formation and Rearrangement of Tricyclo[7.1.1.04,10undeca-2,4,6,8-Tetraen-10-Ol, Novel Pathway from Bicyclo[4.4.1]Undeca-1(10),2,4,8-Tetraen-11-One to 4-Methylazulene. *Tetrahedron Lett.* **1983**, *24* (39), 4261–4264. https://doi.org/10.1016/S0040-4039(00)88316-1.

(99) Liu, S.; Yang, P.; Peng, S.; Zhu, C.; Cao, S.; Li, J.; Sun, J. Gold-Catalyzed Sequential Annulations towards 3,4-Fused Bi/Tri-Cyclic Furans Involving a [3+2+2]-Cycloaddition. *Chem. Commun.* **2017**, *53* (6), 1152–1155. https://doi.org/10.1039/c6cc09154g.

(100) Tóth, G.; Frank, J.; Bende, Z.; Weber, L.; Simon, K. 1,3-Dipolar Cycloaddition Reaction of 3,4-Dihydro-6,7-Dimethoxyisoquinoline Ylide with Olefins. *J. Chem. Soc. Perkin Trans. 1* **1983**, 1961–1966. https://doi.org/10.1039/p19830001961.

(101) Weng, S. S.; Hsieh, K. Y.; Zeng, Z. J.; Zhang, J. W. Synergistic Copper-TEMPO Catalysis of Intermolecular Vicinal Diamination of Styrenes. *Tetrahedron Lett.* **2017**, *58* (7), 670–673. https://doi.org/10.1016/j.tetlet.2017.01.015.

(102) Bilke, J. L.; Dzuganova, M.; Fröhlich, R.; Würthwein, E. U. Diastereoselective One-Step Synthesis of Functionalized Cis-Aziridinyl Alcohols from Oxiranyl Carbaldimines. *Org. Lett.* **2005**, *7* (15), 3267–3270. https://doi.org/10.1021/ol051090l.

(103) Fujiwara, K.; Kurahashi, T.; Matsubara, S. Cationic Iron(III) Porphyrin-Catalyzed [4 + 2] Cycloaddition of Unactivated Aldehydes with Simple Dienes. *J. Am. Chem. Soc.* **2012**, *134* (12), 5512–5515. https://doi.org/10.1021/ja300790x.

(104) Jiang, L.; Yu, X.; Fang, B.; Wu, J. Silver Triflate-Catalyzed Tandem Reaction of N′-(2-Alkynylbenzylidene)Hydrazide with Pyridyne. *Org. Biomol. Chem.* **2012**, *10* (40), 8102–8107. https://doi.org/10.1039/c2ob26379c.

(105) Sakamoto, M.; Akiyama, Y.; Furumi, N.; Ishii, K.; Tomimatsu, Y.; Date, T. Reaction of N-(1-Phenylalkylidene)Benzylamines with Benzoyl Chlorides. *Chem. Pharm. Bull.* **1983**, *31* (8), 2623–2631.

(106) Sheldrick, W. S.; Trowitzsch, W. Stereochemistry and Tautomerism of Amino Acid Antagonists: α,γ-Diketo Acids and Esters and Their Cyclisation Products. *Zeitschrift fur Naturforsch. - Sect. B J. Chem. Sci.* **1983**, *38* (2), 220–225. https://doi.org/10.1515/znb-1983-0219.

(107) Asghari, S.; Habibi, A. K. One Pot Three-Component Regioselective and Diastereoselective Synthesis of Halogenated Pyrido[2,1-b][1,3]Oxazines. *Tetrahedron* **2012**, *68* (43), 8890–8898. https://doi.org/10.1016/j.tet.2012.08.038.

(108) Nakano, K.; Hidehira, Y.; Takahashi, K.; Hiyama, T.; Nozaki, K. Stereospecific Synthesis of Hetero[7]Helicenes by Pd-Catalyzed Double N-Arylation and Intramolecular O-Arylation. *Angew. Chem. Int. Ed.* **2005**, *44* (43), 7136–7138. https://doi.org/10.1002/anie.200502855.

(109) Boyd, D. R.; McCombe, K. M.; Hamor, T. A.; Jennings, W. B.; Wilson, V. E. Dynamic Stereochemistry of Imines and Derivatives. Part 16. Conformation and Stereodynamics of Oxaziridines, Nitrones, and Imines Containing the N-(1-Mesitylethyl) or (1-Pentamethylphenylethyl) Group; a Nuclear Magnetic Resonance and x-Ray Crystallographi. *J. Chem. Soc. Perkin Trans. 2* **1984**, No. 1, 95–100. https://doi.org/10.1039/P29840000095.

(110) Verma, K.; Banerjee, P. Lewis Acid Catalyzed Formal [3+2] Cycloaddition of Donor-Acceptor Cyclopropanes and 1-Azadienes: Synthesis of Imine Functionalized Cyclopentanes and Pyrrolidine Derivatives. *Adv. Synth. Catal.* **2017**, *359* (21), 3848–3854. https://doi.org/10.1002/adsc.201700744.

(111) Madhavachary, R.; Abdelraheem, E. M. M.; Rossetti, A.; Twarda-Clapa, A.; Musielak, B.; Kurpiewska, K.; Kalinowska-Tłuścik, J.; Holak, T. A.; Dömling, A. Two-Step Synthesis of Complex Artificial Macrocyclic Compounds. *Angew. Chem. Int. Ed.* **2017**, *56* (36), 10725–10729. https://doi.org/10.1002/anie.201704426.

(112) Zhai, L.; Tian, X.; Wang, C.; Cui, Q.; Li, W.; Huang, S. H.; Yu, Z. X.; Hong, R. Construction of Morphan Derivatives by Nitroso–Ene Cyclization: Mechanistic Insight and Total Synthesis of (±)-Kopsone. *Angew. Chem. Int. Ed.* **2017**, *56* (38), 11599–11603. https://doi.org/10.1002/anie.201706018.

(113) Kaftory, M.; Weisz, A. Structures of Various Adducts between 2,5-Dimethyl-1,4-Benzoquinone or 2-Methyl-1,4-Naphthoquinone and 1,1'-Bicycloalkenyls. *Acta Cryst. C* **1984**, *40* (3), 456–464. https://doi.org/10.1107/s0108270184004509.

(114) Deppisch, B.; Vittinghoff, K. 3-Chloro-1-Ethyl-2-Methyl-1-Phenylindene. *Acta Cryst. B* **1980**, *36* (12), 3191–3193. https://doi.org/10.1107/s0567740880011272.

(115) Knölker, H. J.; Baum, G.; Schmitt, O.; Wanzl, G. Stereoselective Total Synthesis of (±)-Fragranol by TiCl4 Promoted [2 + 2] Cycloaddition of Allyl-Tert-Butyldiphenylsilane and Methyl Methacrylate. *Chem. Commun.* **1999**, *1* (17), 1737–1738. https://doi.org/10.1039/a905019a.

(116) Iwaki, T.; Yamada, F.; Funaki, S.; Somei, M. Three New Synthetic Methods for the 1,2,3,3a,8,8a-Hexa-Hydropyrrolo[2,3-b] Indoles Having an Alkoxy Group at the 3a-Position. *Heterocycles* **2005**, *65* (8), 1811–1815. https://doi.org/10.3987/COM-05-10443.

(117) Xie, L.; Hu, H.; Cui, C. Cyclopropanation and Isomerization Reactions of β-Diketiminato Boron Complexes. *Organometallics* **2012**, *31* (12), 4405–4408. https://doi.org/10.1021/om300337r.

(118) Zheng, Z.; Yu, Z.; Wang, L.; He, W.; Liu, Z.; Xiuwen Han. Biscarbene Complexes from the Reactions of O-Ethyl Lactim and 1-Alkynyl Fischer Carbene Complexes of Chromium and Tungsten. *J. Organomet. Chem.* **2006**, *691* (23), 5007–5015. https://doi.org/10.1016/j.jorganchem.2006.08.056.

(119) Fujita, T.; Kinoshita, R.; Takanohashi, T.; Suzuki, N.; Ichikawa, J. Ring-Size-Selective Construction of Fluorine-Containing Carbocycles via Intramolecular Iodoarylation of 1,1-Difluoro-1-Alkenes. *Beilstein J. Org. Chem.* **2017**, *13* (1), 2682–2689. https://doi.org/10.3762/bjoc.13.266.

(120) Hegedus, L. S.; McGuire, M. A.; Schultze, L. M.; Yijun, C.; Anderson, O. P. Reaction of Chromium Carbene Complexes with Imines. Synthesis of β-Lactams. *J. Am. Chem. Soc.* **1984**, *106* (9), 2680–2687. https://doi.org/10.1021/ja00321a032.

(121) Panja, S. K.; Saha, S. Recyclable, Magnetic Ionic Liquid Bmim[FeCl4]-Catalyzed, Multicomponent, Solvent-Free, Green Synthesis of Quinazolines. *RSC Adv.* **2013**, *3* (34), 14495–14500. https://doi.org/10.1039/c3ra42039f.

(122) Seiler, M.; Schumacher, A.; Lindemann, U.; Barbosa, F.; Giese, B. Diastereoselective Photocyclization to Dihydroindolinols. *Synlett* **1999**, No. 10, 1588–1590. https://doi.org/10.1055/s-1999-2902.

(123) Guo, H. M.; Jian, F. F.; Zhao, P. S.; Huang, B. Y.; Lin, C. H. 3-(4-Chlorophenyl)-5-(4-Methylphenyl)-1-Phenyl-2-Pyrazoline. *Acta Cryst. E* **2007**, *63* (5), o2622–o2622. https://doi.org/10.1107/S1600536807018867.

(124) Satyamurthy, N.; Berlin, K. D.; Powell, D. R.; Helm, D. Van Der. Synthesis of 3-Methylene-1-Oxa-8-Heteraspiro[4.5]Decan-2-Ones and 1-Oxa-8-Heteraspiro[4.5]Decan-2-Ones Novel Formation and Crystal Structure of 3-[(4-Hydroxy-4-Thianyl)Methyl] - 1-Oxa-8-Thiaspiro[4.5]Decan-2-One. *Phosphorus Sulfur Relat. Elem.* **1984**, *19* (2), 137–153. https://doi.org/10.1080/03086648408077573.

(125) Sun, W.; Wilson, D. C.; Light, M. E.; Harrowven, D. C. A Thermally Induced Hydride Transfer from an Amine to an Allene Triggers an Annulation Reaction, Giving Dihydrofuropyridinones. *Org. Lett.* **2018**, *20* (14), 4346–4349. https://doi.org/10.1021/acs.orglett.8b01792.

(126) Mondal, S.; Mohamed, R. K.; Manoharan, M.; Phan, H.; Alabugin, I. V. Drawing from a Pool of Radicals for the Design of Selective Enyne Cyclizations. *Org. Lett.* **2013**, *15* (22), 5650–5653. https://doi.org/10.1021/ol4028072.

(127) Wang, Z.; Wang, Z.; Ren, J. Lewis Acids Promoted Formal Intramolecular [3 + 2] Parallel and Cross-Cycloadditions of Cyclopropane 1,1-Diesters with Allenes. *Org. Lett.* **2013**, *15* (22), 5682–5685.

https://doi.org/10.1021/ol402662j.

(128) Rudler, H.; Parlier, A.; Bezennine-Lafollee, S.; Vaissermann, J. Synthesis of Functionalized Quinolizines and Homoquinolizines from N-Alkynylaminocarbene Complexes of Chromium Intramolecular C–H Activation. *Eur. J. Org. Chem.* **1999**, No. 11, 2825–2833.

(129) Rudinger-Adler, E.; Hostettmann, M.; Gramlich, V.; Petter, W. Synthese Und Kristallstruktur Der Hydroxy-lactone von 6-Methyl-2-propionyl- Und 3-Methyl-2-propionylbenzoesäure. *Helv. Chim. Acta* **1984**, *67* (3), 743–747. https://doi.org/10.1002/hlca.19840670314.

(130) Bergman, J.; Sidén, J.; Maartmann-Moe, K. Structure Elucidation of a Zwitterionic 2-Oxazoline Obtained by Cyclofunctionalization of n-Acetyldiallylamine with Tellurium Tetrachloride. *Tetrahedron* **1984**, *40* (9), 1607–1610. https://doi.org/10.1016/S0040-4020(01)91812-5.

(131) Borade, B. R.; Nomula, R.; Gonnade, R. G.; Kontham, R. Fe(III)-Catalyzed Diastereoselective Friedel-Crafts Alkylation-Hemiketalization-Lactonization Cascade for the Synthesis of Polycyclic Bridged 2-Chromanol Lactones. *Org. Lett.* **2019**, *21* (8), 2629–2633. https://doi.org/10.1021/acs.orglett.9b00614.

(132) Tsuji, H.; Fujimoto, T.; Endo, K.; Nakamura, M.; Nakamura, E. Stereoselective Synthesis of Trisubstituted E-Iodoalkenes by Indium-Catalyzed Syn-Addition of 1,3-Dicarbonyi Compounds to 1-Iodoaikynes. *Org. Lett.* **2008**, *10* (6), 1219–1221. https://doi.org/10.1021/ol800105r.

(133) Lorkowski, J.; Krahfuß, M.; Kubicki, M.; Radius, U.; Pietraszuk, C. Intramolecular Ring-Expansion Reaction (RER) and Intermolecular Coordination of In Situ Generated Cyclic (Amino)(Aryl)Carbenes (CAArCs). *Chem. Eur. J.* **2019**, *25* (48), 11365–11374. https://doi.org/10.1002/chem.201902630.

(134) Weber, E.; Zaumüller, A.; Seichter, W.; Czugler, M. Triol Crystalline Hosts Derived from Malic Acid. Synthesis, Inclusion Formation and x-Ray Crystal Structures of a Free Host and Its Inclusion Compound with Ethanol (1:1). *Supramol. Chem.* **1997**, *8* (4), 351–359. https://doi.org/10.1080/10610279708034954.

(135) Obregón-Mendoza, M. A.; Escobedo-Martínez, C.; Lozada, M. C.; Gnecco, D.; Soriano-García, M.; Enríquez, R. G. Investigation of Three Diasteromeric Chalcone Epoxides Derivatives by Nmr Spectroscopy and X-Ray Crystallography. *J. Chem. Crystallogr.* **2014**, *44* (10), 512–519. https://doi.org/10.1007/s10870-014-0544-0.

(136) Gilman, N. W.; Blount, J. F.; Sternbach, L. H. Quinazolines and 1,4-Benzodiazepines. LIV.1 the Base-Catalyzed Rearrangement of 2-Dimethylamino-5-Phenyl-7-Chloro-3H-1,4-Benzodiazepine 4-Oxide. *J. Org. Chem.* **1972**, *37* (21), 3201–3206. https://doi.org/10.1021/jo00986a001.

(137) Guggenberger, L. J.; Jacobson, R. A. The Crystal Structure of a Benzocyclopropapyran. *Acta Cryst. B* **1969**, *25* (5), 888–894. https://doi.org/10.1107/s0567740869003189.

(138) Chen, H.; Kaga, A.; Chiba, S. Diastereoselective Aminooxygenation and Diamination of Alkenes with Amidines by Hypervalent Iodine(III) Reagents. *Org. Lett.* **2014**, *16* (23), 6136–6139. https://doi.org/10.1021/ol503000c.

(139) Parlier, A.; Kadouri-Puchot, C.; Beaupierre, S.; Jarosz, N.; Rudler, H.; Hamon, L.; Herson, P.; Daran, J. C. An Intramolecular Journey of a Carboxyl Group around 1,2-Dihydropyridines: Multisite δ- versus γ-Lactonization Reactions. *Tetrahedron Lett.* **2009**, *50* (52), 7274–7279. https://doi.org/10.1016/j.tetlet.2009.10.038.

(140) Sternbach, D. D.; Rossana, D. M.; Onan, K. D. Intramolecular Diels-Alder Reactions with Furan: The Gem-Dialkyl Effect Revisited. *Tetrahedron Lett.* **1985**, *26* (5), 591–594. https://doi.org/10.1016/S0040-4039(00)89155-8.

(141) Schuler, M.; Silva, F.; Bobbio, C.; Tessier, A.; Gouverneur, V. Gold(I)-Catalyzed Alkoxyhalogenation of β-Hydroxy-α,α- Difluoroynones. *Angew. Chem. Int. Ed.* **2008**, *47* (41), 7927–7930. https://doi.org/10.1002/anie.200802162.

(142) Liu, G. Q.; Li, L.; Duan, L.; Li, Y. M. MCPBA-Mediated Metal-Free Intramolecular Aminohydroxylation and Dioxygenation of Unfunctionalized Olefins. *RSC Adv.* **2015**, *5* (75), 61137–61143. https://doi.org/10.1039/c5ra09024e.

(143) Gałdecki, Z.; Bartczak, T. J.; Wolf, W. M.; Krawczyk, H.; Majewski, P. Structure of 1,2,5-Trihydroxy-2,5-Dimethylphospholane 1-Oxide, C6H13O4P. *Acta Cryst. C* **1985**, *41* (5), 732–734. https://doi.org/10.1107/s0108270185005297.

(144) Hu, Y.; Qu, Y.; Wu, F.; Gui, J.; Wei, Y.; Hu, Q.; Wang, S. Tuned C-H Functionalization to Construct Aza-Podophyllotoxin/Aza- Conidendrin Derivatives by Means of Domino Cyclization. *Chem. Asian J.* **2010**, *5* (2), 309–314. https://doi.org/10.1002/asia.200900307.

(145) Flowers, W. T.; Moss, S. F.; Robinson, J. F.; Taylor, D. R.; Tipping, A. E.; Haley, M. J. Unexpected Formation of Amidino-Thiadiazolines by Reaction of a Chlorodiazabutadiene with Thioureas. *J. Chem. Soc. Chem. Commun.* **1979**, No. 4, 149–151. https://doi.org/10.1039/C39790000149.

(146) Goncalves, S.; Nicolas, M.; Maillos, P.; Baati, R. Cationic Cyclization of Keto-Epoxides Mediated by Zirconium(IV)

Tetrachloride: Diastereoselective Synthesis of Cis-Decalinols. *Tetrahedron* **2011**, *67* (43), 8373–8382. https://doi.org/10.1016/j.tet.2011.08.050.

(147) Fichtler, R.; Neudörfl, J. M.; Jacobi Von Wangelin, A. Practical Three-Component Synthesis of Crowded Arenes with Donor-Acceptor Substitution. *Org. Biomol. Chem.* **2011**, *9* (20), 7224–7236. https://doi.org/10.1039/c1ob05984j.

(148) Murali, A.; Puppala, M.; Varghese, B.; Baskaran, S. A Lewis Acid Mediated Schmidt Reaction of Benzylic Azide: Synthesis of Sterically Crowded Aromatic Tertiary Amines. *Eur. J. Org. Chem.* **2011**, *2011* (27), 5297–5302. https://doi.org/10.1002/ejoc.201100674.

(149) Smirnov, V. O.; Ioffe, S. L.; Tishkov, A. A.; Khomutova, Y. A.; Nesterov, I. D.; Antipin, M. Y.; Smit, W. A.; Tartakovsky, V. A. New C-C Coupling Reaction of Cyclic Nitronates with Carbon Nucleophiles. Umpolung of the Conventional Reactivity of Nitronates. *J. Org. Chem.* **2004**, *69* (24), 8485–8488. https://doi.org/10.1021/jo048944k.

(150) Ito, Y.; Ji-Ben, M.; Suzuki, S.; Kusunaga, Y.; Matsuura, T.; Fukuyama, K. Efficient Photochemical Oxetane Formation from 1-Methyl-2,4,5-Triphenylimidazole and Benzophenones. *Tetrahedron Lett.* **1985**, *26* (17), 2093–2096. https://doi.org/10.1016/S0040-4039(00)94787-7.

(151) Roche, V. F.; Roche, E. B.; Nagel, D. L.; Mcphail, A. T. Studies in the Synthesis of C Ring Bridged Morphinans. 2.1 The Synthesis and Structural Verification of a Novel 3, Llc-Ethano-10-Hydroxy-6-Methyl-l, 2, 3, 3a, Llb, Llc-Hexahydroaporphine. *J. Org. Chem.* **1984**, *49* (21), 3881–3887. https://doi.org/10.1021/jo00195a002.

(152) Dilman, A. D.; Belyakov, P. A.; Korlyukov, A. A.; Struchkova, M. I.; Tartakovsky, V. A. Synthesis of Pentafluorophenylmethylamines via Silicon Mannich Reaction. *Org. Lett.* **2005**, *7* (14), 2913–2915. https://doi.org/10.1021/ol050845l.

(153) Bredikhin, A. A.; Strunskaya, E. I.; Zakharychev, D. V.; Krivolapov, D. B.; Litvinov, I. A.; Bredikhina, Z. A. Solid State Properties of 1,2-Epoxy-3-(2-Methoxyphenyloxy)-Propane - Valuable Intermediate in Non-Racemic Drug Synthesis. *Tetrahedron Asymmetry* **2005**, *16* (20), 3361–3366. https://doi.org/10.1016/j.tetasy.2005.09.013.

(154) Liang, J. Y.; Wang, H.; Yang, Y. L.; Shen, S. J.; Chen, J. X. Addition of Carbamoylsilane to Isatins: Highly Efficient Synthesis of 3-Hydroxy-3-Aminocarbonyl-2-Oxindoles Derivatives. *Tetrahedron Lett.* **2017**, *58* (27), 2636–2639. https://doi.org/10.1016/j.tetlet.2017.05.051.

(155) Mao, Z.; Qu, H.; Zhao, Y.; Lin, X. A General Access to 1,1-Cyclopropane Aminoketones and Their Conversion into 2-Benzoyl Quinolines. *Chem. Commun.* **2012**, *48* (79), 9927–9929. https://doi.org/10.1039/c2cc35235d.

(156) Grayfer, T. D.; Retailleau, P.; Dodd, R. H.; Dubois, J.; Cariou, K. Chemodivergent, Tunable, and Selective Iodine(III)-Mediated Bromo-Functionalizations of Polyprenoids. *Org. Lett.* **2017**, *19* (18), 4766–4769. https://doi.org/10.1021/acs.orglett.7b02125.

(157) Archer, S. J.; Irving, H. M. N. H.; Koch, K. R.; Nassimbeni, L. R. Isomerism in Parabutylchloral; x-Ray Crystallographic Determinations of 2,4,6-Tris(1′,1′,2′-Trichloropropyl)-1,3,5-Trioxanes. *J. Crystallogr. Spectrosc. Res.* **1985**, *15* (4), 333–349. https://doi.org/10.1007/BF01160726.

(158) Klunder, A. J. H.; de Valk, W. C. G. M.; Verlaak, J. M. J.; Schellekens, J. W. M.; Noordik, J. H.; Parthasarathi, V.; Zwanenburg, B. Nucleophilic Eliminative Ring Fission of Bridgehead Substituted 1,3-Bishomocubyl Acetates. *Tetrahedron* **1985**, *41* (5), 963–973. https://doi.org/10.1016/S0040-4020(01)96416-6.

(159) Hermecz, I.; Kajtár, M.; Simon, K.; Breining, T.; Surján, P. R.; Tóth, G.; Mészáros, Z. Nitrogen Bridgehead Compounds. 48.1 Synthesis and Stereochemistry of 4-Oxo-1,6,7,8,9,9a-Hexahydro-4H-Pyrido[1,2-a]Pyrimidine-3-Carboxamides. *J. Org. Chem.* **1985**, *50* (16), 2918–2925. https://doi.org/10.1021/jo00216a022.

(160) Roesky, H. W.; Lucas, J.; Weber, K. -L; Djarrah, H.; Egert, E.; Noltemeyer, M.; Sheldrick, G. M. Reaktionen von Hexafluoraceton Mit Nitrilen Der V. Und VI. Hauptgruppe. *Chem. Ber.* **1985**, *118* (6), 2396–2406. https://doi.org/10.1002/cber.19851180619.

(161) Alex, G.; Srinivasan, S.; Ramadas, S. R.; Appa Rao, J. Structure of an Epipolysulfide: 3a,6-Dihydro-1-Methylcyclopenta[6,7][1,2]Dithiepino[5,4-b][1]Benzothiophen-2(3H)-One. *Acta Cryst. C* **1985**, *41* (10), 1463–1465. https://doi.org/10.1107/s0108270185008174.

(162) Ramadas, S. R.; Chenchaiah, P. C.; Rao, J. A.; Kumaresan, S. Approaches to the Synthesis of 12-Thiasteroids: Synthesis of B-nor-6,12-Bisthiaestra-1,3,5(10),8,14-Pentaen-17-One. *Tetrahedron Lett.* **1983**, *24* (48), 5403–5406. https://doi.org/10.1016/S0040-4039(00)87880-6.

(163) Bharadwaj, P.; Potenza, J. A.; Ornaf, R. M.; Rodriques, K. E.; Knapp, S.; Lalancette, R. A. Structure of Cis-9-[(Benzyloxy)Methoxy]-4a,9,9a,10-Tetrahydro-9,10-o-Benzenoanthracene-1,4-Dione, a Sterically Congested 2-Ene-1,4-Dione. *Acta Cryst. C* **1985**, *41* (10), 1520–1522. https://doi.org/10.1107/s0108270185008393.

(164) Yang, B.; Lin, K.; Shi, Y.; Gao, S. Ti(Oi-Pr)4-Promoted Photoenolization Diels-Alder Reaction to Construct Polycyclic Rings and Its Synthetic Applications. *Nat. Commun.* **2017**, *8* (1), 1–10. https://doi.org/10.1038/s41467-017-00440-8.

(165) Fichou, D.; Tonnard, F.; Toupet, L.; Carrié, R. Regioselectivite de La Cycloaddition Dipolaire-1,3. Reactions de Dibenzo-2,3:6,7 Heptafulvenes Avec Quelques Dipoles. *Tetrahedron* **1984**, *40* (24), 5121–5133. https://doi.org/10.1016/S0040-4020(01)91261-X.

(166) Hoffmann, K. -L; Maas, G.; Regitz, M. Carbene, 30. Metallkatalysierte Zersetzung von 4-Diazomethyl-4H-pyranen – Ein Neuer Zugang in Das Oxepin-System. *Chem. Ber.* **1985**, *118* (9), 3700–3713. https://doi.org/10.1002/cber.19851180923.

(167) Segawa, Y.; Yamashita, M.; Nozaki, K. Boryllithium: Isolation, Characterization, and Reactivity as a Boryl Anion. *Science* **2006**, *314* (5796), 113–115. https://doi.org/10.1126/science.1131914.

(168) Segawa, Y.; Suzuki, Y.; Yamashita, M.; Nozaki, K. Chemistry of Boryllithium: Synthesis, Structure, and Reactivity. *J. Am. Chem. Soc.* **2008**, *130* (47), 16069–16079. https://doi.org/10.1021/ja8057919.

(169) Feng, C.; Li, Y.; Xu, Q.; Pan, L.; Liu, Q.; Xu, X. Triple Nucleophilic Attack of Nitromethane on (2-Iminoaryl)Divinyl Ketones: A Domino Synthetic Strategy for Hexahydrophenanthridinones. *J. Org. Chem.* **2018**, *83* (3), 1232–1240. https://doi.org/10.1021/acs.joc.7b02759.

(170) Cheng, S. G.; Hourcade, S.; Ferdenzi, A.; Chiaroni, A.; Mons, S.; Delpech, B.; Marazano, C. A Biogenetically Based Strategy towards the Polycyclic Core Skeleton of Sarain A. *Eur. J. Org. Chem.* **2006**, *2006* (18), 4106–4114. https://doi.org/10.1002/ejoc.200600293.

(171) Wang, M. A.; Tu, G. Z.; Ma, Z. C.; Zhang, N.; Wang, D. Q. Synthesis and Structural Characteristics of Two Novel Bicyclododecyl Derivatives. *Chinese J. Chem.* **2006**, *24* (2), 205–209. https://doi.org/10.1002/cjoc.200690039.

(172) Hickmott, P. W.; Ahmed, M. G.; Ahmed, S. A.; Wood, S.; Kapon, M. Enamine Chemistry. Part 29. Synthesis of Adamantane Derivatives from α, β-Unsaturated Acid Chlorides and 4,4-Disubstituted Cyclohexanone Enamines. Multiple [3,3] Sigmatropic Rearrangement Transition State Stereochemistry. X-Ray Analysis. *J. Chem. Soc. Perkin Trans. 1* **1985**, No. 0, 2559–2571. https://doi.org/10.1039/p19850002559.

(173) Jørgensen, F. S.; Gajhede, M.; Frei, B. Cyclic Acetals. Structural Analysis of 1,3-Dioxepine and Related Compounds. *Helv. Chim. Acta* **1985**, *68* (8), 2148–2157. https://doi.org/10.1002/hlca.19850680809.

(174) Martin, P.; Streith, J.; Rihs, G.; Winkler, T.; Belluš, D. A New Electrophilic 2-Pyrone Bearing a CF3- Group, Its Preparation and Its [4+2] Cycloaddition Reactions. *Tetrahedron Lett.* **1985**, *26* (33), 3947–3950. https://doi.org/10.1016/S0040-4039(00)98694-5.

(175) Chandrasekhar, S.; Chopra, D.; Gopalaiah, K.; Guru Row, T. N. The Generalized Anomeric Effect in the 1,3-Thiazolidines: Evidence for Both Sulphur and Nitrogen as Electron Donors. Crystal Structures of Various N-Acylthiazolidines Including Mercury(II) Complexes. Possible Relevance to Penicillin Action. *J. Mol. Struct.* **2007**, *837* (1–3), 118–131. https://doi.org/10.1016/j.molstruc.2006.10.034.

(176) Letcher, R. M.; Lai, T. F. The X-Ray Molecular Structures of Methyl 4,5,7,8,9,10,11,12,13,14-Decahydro-7,9-Dioxo-8,15-Methenopyrrolo[3,2,1 -Op] [1] Benzaracyclododecine-16-Carboxylate, Methyl (2)-1′,2′,4′,5′-Tetra Hydro-2,4′-Dioxospiro(Cycloheptane-1,6′-[6H]Pyrrolo[3,2,1 -Ij]Quinol. *J. Chem. Soc. Perkin Trans. 1* **1985**, No. 0, 1921–1926. https://doi.org/10.1039/P19850001921.

(177) Vaz, W. F.; Custodio, J. M. F.; Rodrigues, N. M. N.; Santin, L. G.; Oliveira, S. S.; Gargano, R.; Osório, F. A. P.; Aquino, G. L. B.; Camargo, A. J.; Oliveira, M. S.; Napolitano, H. B. A Novel Dihydrocoumarin under Experimental and Theoretical Characterization. *J. Mol. Model.* **2017**, *23* (11), 1–13. https://doi.org/10.1007/s00894-017-3485-7.

(178) Soo, Y. C.; Sang, I. L.; Kang, H. P.; Young, K. C. Sequential Diels-Alder and Cobalt Octacarbonyl Catalyzed Pauson-Khand Reactions in the Formation of Polycyclic Enones. *Synlett* **2007**, *2007* (12), 1857–1862. https://doi.org/10.1055/s-2007-984511.

(179) Begley, M. J.; Crombie, L.; Jones, R. C. F.; Palmer, C. J. Synthesis of the Mammea Coumarins. Part 4. Stereochemical and Regiochemical Studies, and Synthesis of (-)-Mammea B/BB. *J. Chem. Soc. Perkin Trans. 1* **1987**, No. 0, 353–357. https://doi.org/10.1039/p19870000353.

(180) Alhomaidan, O.; Hollink, E.; Stephan, D. W. Main Group Heterocycles from Lithiated Phosphinimines. *Organometallics* **2007**, *26* (12), 3041–3048. https://doi.org/10.1021/om070154o.

(181) Kalshetti, M. G.; Argade, N. P. Diastereoselective Synthesis of (±)- Epi-Subincanadine C. *ACS Omega* **2018**, *3* (5), 5308–5316. https://doi.org/10.1021/acsomega.8b00587.

(182) Guo, X.; Liu, W.; Hu, W. A Facile Access to Polyfunctional Oxygen-Containing Heterocycles via Intramolecularly

Formed Protic Oxonium Ylide Trapping Processes. *Chem. Asian J.* **2014**, *9* (1), 117–120. https://doi.org/10.1002/asia.201301115.

(183) Argay, G.; Kálmán, A.; Ribár, B.; Bernáth, G. Stereochemical Studies. 115. Structure of Trans-2-(P-chlorophenyl)-1,2,4a,5,8,8a-hexahydro-4H-3,1-benzoxazine. *Acta Cryst. C* **1986**, *42* (12), 1884–1886. https://doi.org/10.1107/S0108270186090157.

(184) Amako, Y.; Hori, H.; Arai, S.; Nishida, A. Regioselective Hydronickelation of Allenes and Its Application to the Hydrocyanative Carbocyclization Reaction of Allene-Ynes and Bis-Allenes. *J. Org. Chem.* **2013**, *78* (21), 10763–10775. https://doi.org/10.1021/jo401758v.

(185) Radcliffe, J. E.; Fasano, V.; Adams, R. W.; You, P.; Ingleson, M. J. Reductive α-Borylation of α,β-Unsaturated Esters Using NHC-BH3 Activated by I2 as a Metal-Free Route to α-Boryl Esters. *Chem. Sci.* **2019**, *10* (5), 1434–1441. https://doi.org/10.1039/c8sc04305a.

(186) Zeng, Y.; Liu, F. 1,3-Dipolar Cycloaddition in the Synthesis of Novel Isoxazoline/Pyrazole Derivatives Bearing 1,2,3-Triazoles Moiety. *J. Heterocycl. Chem.* **2013**, *50* (3), 696–702. https://doi.org/10.1002/jhet.1527.

(187) Zhang, X.; Liu, Z.; Yang, X.; Dong, Y.; Virelli, M.; Zanoni, G.; Anderson, E. A.; Bi, X. Use of Trifluoroacetaldehyde N-Tfsylhydrazone as a Trifluorodiazoethane Surrogate and Its Synthetic Applications. *Nat. Commun.* **2019**, *10* (1), 1–9. https://doi.org/10.1038/s41467-018-08253-z.

(188) Ye, B.; Yao, Z. J.; Burke, T. R. Synthesis of a New Tyrosine Analogue Having X1 and X2 Angles Constrained to Values Observed for an SH2 Domain-Bound Phosphotyrosyl Residue. *J. Org. Chem.* **1997**, *62* (16), 5428–5431. https://doi.org/10.1021/jo9700787.

(189) Christl, M.; Mattauch, B.; Irngartinger, H.; Goldmann, A. Additionen von Benzvalen an Nitriloxide. Eine Synthese Für Benzvalen-3-carbonitril. *Chem. Ber.* **1986**, *119* (3), 950–959. https://doi.org/10.1002/cber.19861190318.

(190) Ariel, S.; Trotter, J. 2-Cyclohexyl-1-(4-Methoxyphenyl)-2-Phenylethanone. *Acta Cryst. C* **1986**, *42* (4), 485–487. https://doi.org/10.1107/s0108270186095707.

(191) Bertolasi, V.; Ferretti, V.; Gilli, P.; Yao, X.; Li, C. J. Substituent Effects on Keto-Enol Tautomerization of β-Diketones from X-Ray Structural Data and DFT Calculations. *New J. Chem.* **2008**, *32* (4), 694–704. https://doi.org/10.1039/b714708b.

(192) Pascard-Billy, C. Structure Cristalline et Moléculaire d'un Dérivé Cyclobutanique de 5,6-Dihydrodiméthyluracile. *Acta Cryst. B* **1973**, *29* (3), 521–529. https://doi.org/10.1107/s0567740873002840.

(193) Matviiuk, T.; Rodriguez, F.; Saffon, N.; Mallet-Ladeira, S.; Gorichko, M.; De Jesus Lopes Ribeiro, A. L.; Pasca, M. R.; Lherbet, C.; Voitenko, Z.; Baltas, M. Design, Chemical Synthesis of 3-(9H-Fluoren-9-Yl)Pyrrolidine-2,5-Dione Derivatives and Biological Activity against Enoyl-ACP Reductase (InhA) and Mycobacterium Tuberculosis. *Eur. J. Med. Chem.* **2013**, *70*, 37–48. https://doi.org/10.1016/j.ejmech.2013.09.041.

(194) Liu, Y.; Zhou, S.; Li, G.; Yan, B.; Guo, S.; Zhou, Y.; Zhang, H.; Wang, P. G. Highly Efficient Brønsted Acid-Catalyzed Cycloisomerizations of Alkynes Bearing Bis(Acetoxy) Groups to Indenyl Ketones. *Adv. Synth. Catal.* **2008**, *350* (6), 797–801. https://doi.org/10.1002/adsc.200800037.

(195) Nienkemper, K.; Kehr, G.; Kehr, S.; Fröhlich, R.; Erker, G. (Amidomethyl)Pyridine Zirconium and Hafnium Complexes: Synthesis and Structural Characterization. *J. Organomet. Chem.* **2008**, *693* (8–9), 1572–1589. https://doi.org/10.1016/j.jorganchem.2007.12.004.

(196) Kostyanovsky, R. G.; Lyssenko, K. A.; Lenev, D. A.; El'natanov, Y. I.; Krutius, O. N.; Bronzova, I. A. 3,7-Dimethyl-3,7-Diazabicyclo[3.3.1]Nonane-2,6-Dione-1,5-Dicarboxylic Acid Derivatives: Synthesis, Structure and Resolution. *Mendeleev Commun.* **1999**, *9* (4), 151–153. https://doi.org/10.1070/mc1999v009n04abeh001067.

(197) Kostyanovsky, R. G.; Lyssenko, K. A.; El'natanov, Y. I.; Krutius, O. N.; Bronzova, I. A.; Strelenko, Y. A.; Kostyanovsky, V. R. 3,7-Diazabicyclo[3.3.1]Nonane-2,6-Diones: Building of Homo- and Heterochiral Crystals. *Mendeleev Commun.* **1999**, *9* (3), 106–108. https://doi.org/10.1070/mc1999v009n03abeh001058.

(198) Möricke, J.; Rehwinkel, F.; Danelzik, T.; Daniliuc, C. G.; Wibbeling, B.; Kehr, G.; Erker, G. Developing Carbon Lewis Base/Boron Lewis Acid Frustrated Lewis Pair Chemistry Derived from Conjugated Dienamines. *Tetrahedron* **2019**, *75* (5), 571–579. https://doi.org/10.1016/j.tet.2018.11.068.

(199) Feldman, K. S.; Simpson, R. E.; Parvez, M. Synthesis of Polyoxygenated Hydrocarbons via Radical-Mediated Oxygenation of Vinylcyclopropanes. *J. Am. Chem. Soc.* **1986**, *108* (6), 1328–1330. https://doi.org/10.1021/ja00266a051.

(200) Zhang, R.; Zhang, Z.; Zhou, Q.; Yu, L.; Wang, J. The Generation of Difluoroketenimine and Its Application in the Synthesis of α,α-Difluoro-β-Amino Amides. *Angew. Chem. Int. Ed.* **2019**, *58* (17), 5744–5748. https://doi.org/10.1002/anie.201901591.

(201) Reddy, B. V. S.; Babu, R. A.; Ramana Reddy, M.; Reddy, B. J. M.; Sridhar, B. Intramolecular C-O/C-S Bond Insertion of α-Diazoesters for the Synthesis of 2-Aryl-4H-Benzo[d][1,3]Oxazine and 2-Aryl-4H-Benzo[d][1,3]Thiazine Derivatives. *RSC Adv.* **2014**, *4* (84), 44629–44633. https://doi.org/10.1039/c4ra08208g.

(202) Karolak-Wojciechowska, J.; Trzeźwińska, H. B.; Alibert-Franco, S.; Santelli-Rouvier, C.; Barbe, J. The Crystal and Molecular Structures of 9,10-Dihydro-9, 10-Ethano- and Etheno-Anthracenes. *J. Chem. Crystallogr.* **1998**, *28* (12), 905–911. https://doi.org/10.1023/A:1022854604046.

(203) Savinov, S. N.; Austin, D. J. The Diastereoselective Cycloaddition of Vinyl Ethers with Isomunchnones. *Chem. Commun.* **1999**, No. 18, 1813–1814. https://doi.org/10.1039/a903381e.

(204) Kuran, B.; Kossakowski, J.; Cieślak, M.; Kazmierczak-Barańska, J.; Królewska, K.; Cyrański, M. K.; Stepień, D. K.; Krawiecka, M. Synthesis and Biological Activity of Novel Series of Heterocyclic Compounds Containing Succinimide Moiety. *Heterocycl. Commun.* **2013**, *19* (4), 287–296. https://doi.org/10.1515/hc-2013-0050.

(205) Krow, G. R.; Raghavachari, R.; Siatkowski, R.; Chodosh, D. F. Crystal Structure of 1-(Phenoxycarbonyl)-2-(p-Chlorophenyl)-4,5-Di-Methyl-1,2-Dihydropyridine. Insight into the Facial Selectivity of 1,2-Dihydropyridine Diels-Alder Cycloadditions. *J. Org. Chem.* **1986**, *51* (10), 1916–1918. https://doi.org/10.1021/jo00360a055.

(206) Eagan, R. L.; Ogliaruso, M. A.; Springer, J. P. An Efficient and Novel Approach to the Synthesis of 3,4,5,6-Tetraphenyl-2(1H)-Pyridinone. *J. Org. Chem.* **1986**, *51* (9), 1544–1547. https://doi.org/10.1021/jo00359a031.

(207) Heeg, M. J.; Subramanian, P.; Dryhurst, G. Structure of 1′,3,3′-Trimethyloxazolidine-5-Spiro-5′-Hydantoin-2,4-Dione. *Acta Cryst. C* **1986**, *42* (6), 747–750. https://doi.org/10.1107/s0108270186094696.

(208) Jäger, V.; Kuhn, W.; Schubert, U. The 4-Penten-4-Olide Group: A Novel, Multidirectional Participant in Cationic Olefin Cyclization. *Tetrahedron Lett.* **1986**, *27* (23), 2583–2586. https://doi.org/10.1016/S0040-4039(00)84590-6.

(209) Ibata, T.; Toyoda, J.; Sawada, M.; Tanaka, T. Formation and Reaction of Carbonyl Ylides. Structure of 2: 1-Cycloadducts of 1-Methoxy-2-Benzopyrylium-4-Olate with Isocyanates. *J. Chem. Soc. Chem. Commun.* **1986**, No. 16, 1266–1267. https://doi.org/10.1039/C39860001266.

(210) Matsuda-Sentou, W.; Shinmyozu, T. Multibridged [3(n)] Cyclophanes, 11(+): A Synthetic and Structural Study of 17,18-Dicyano[32](1,6)Cyclooctatetraeno-(1,4)Cyclophane Generated by Photolysis of [32](1,4)Barrelenophane. *Eur. J. Org. Chem.* **2000**, No. 18, 3195–3203. https://doi.org/10.1002/1099-0690(200009)2000:18<3195::aid-ejoc3195>3.0.co;2-1.

(211) Chen, L. M.; Kang, T. R. 3-(1-Naphthyl)-N-Phenyloxirane-2-Carboxamide. *Acta Cryst. E* **2009**, *65* (12), o3137–o3137. https://doi.org/10.1107/S1600536809048752.

(212) Lee, G. A.; Lee, H. Y.; Chen, W. C.; Lin, Y. H. Reactions of α,β-Unsaturated N-Benzenesulfonyl Imine - N-[(2E)-3-Phenyl-2-Propen-1-Ylidene]Benzenesulfonamide with Methyllithium. *J. Chinese Chem. Soc.* **2015**, *62* (7), 573–576. https://doi.org/10.1002/jccs.201500082.

(213) He, Y. T.; Wang, Q.; Zhao, J.; Liu, X. Y.; Xu, P. F.; Liang, Y. M. The Copper-Catalyzed Synthesis of β-Trifluoromethylated Acrylonitriles and Trifluoromethyl-Substituted 2H-Azirines. *Chem. Commun.* **2015**, *51* (67), 13209–13212. https://doi.org/10.1039/c5cc05066a.

(214) Novkovic, L.; Trmcic, M.; Rodic, M.; Bihelovic, F.; Zlatar, M.; Matovic, R.; Saicic, R. N. Synthesis of Endoperoxides by Domino Reactions of Ketones and Molecular Oxygen. *RSC Adv.* **2015**, *5* (120), 99577–99584. https://doi.org/10.1039/c5ra13476e.

(215) Hueso-Falcón, I.; Amesty, Á.; Martín, P.; López-Rodríguez, M.; Fernández-Pérez, L.; Estévez-Braun, A. Indium Catalyzed Solvent-Free Multicomponent Synthesis of Cytotoxic Dibenzo[a,h]Anthracenes from Aldehydes, 2-Hydroxy-1,4-Naphthoquinone, and 2-Naphthol. *Tetrahedron* **2014**, *70* (45), 8480–8487. https://doi.org/10.1016/j.tet.2014.09.076.

(216) Li, H. X.; Zhang, X. J.; Xu, L. Z. (Z)-N-{3-[1-(4-Chlorophenyl)Ethyl]Thiazolidin-2-Ylidene}cyanamide. *Acta Cryst. E* **2010**, *66* (8), o2171–o2171. https://doi.org/10.1107/S1600536810029879.

(217) Evans, S. V.; Garcia-Garibay, M.; Omkaram, N.; Scheffer, J. R.; Trotter, J.; Wireko, F. Use of Chiral Single Crystals To Convert Achiral Reactants to Chiral Products in High Optical Yield: Application to the Di-π-Methane and Norrish Type II Photorearrangements. *J. Am. Chem. Soc.* **1986**, *108* (18), 5648–5650. https://doi.org/10.1021/ja00278a060.

(218) Aversa, M. C.; Giannetto, P.; Ferlazzo, A.; Bruno, G. Formation and X-Ray Crystal Structure of 5, 6-Dihydro-1 -Hydroxy-5, 5-Dimethyl- 1 -Phenyl-3-Phenylamino-1H-Pyrrolo[1, 2-a][1, 5]Benzodiazepin-2(4H)-One. *J. Chem. Soc. Perkin Trans. 2* **1986**, No. 10, 1533–1535. https://doi.org/10.1039/P29860001533.

(219) Ullah, I.; Sher, M.; Khera, R. A.; Ali, A.; Ibad, M. F.; Villinger, A.; Fischer, C.; Langer, P. Chelation-Control in the

Formal [3+3] Cyclization of 1,3-Bis-(Silyloxy)-1,3-Butadienes with 1-Hydroxy-5-Silyloxy-Hex-4-En-3-Ones. One-Pot Synthesis of 3-Aryl-3,4-Dihydroisocoumarins. *Tetrahedron* **2010**, *66* (10), 1874–1884. https://doi.org/10.1016/j.tet.2010.01.019.

(220) Zhang, R.; Zhang, Y. Di; Wang, L. X.; Ge, C. H.; Ma, Z. Y.; Miao, J. P.; Zhang, X. D. Synthesis of a Series of Benzazaboroles as Selective Fluorescent Receptors for Iodide. *Inorg. Chem. Commun.* **2016**, *74*, 52–57. https://doi.org/10.1016/j.inoche.2016.10.033.

(221) Sieburth, S. M.; Fensterbank, L. An Intramolecular Diels-Alder Reaction of Vinylsilanes. *J. Org. Chem.* **1992**, *57* (20), 5279–5281. https://doi.org/10.1021/jo00046a002.

(222) Sieburth, S. M. N.; Joshi, P. V. Intramolecular [4 + 4] Photocycloadditions: Substituent-Mediated Product Control. *J. Org. Chem.* **1993**, *58* (7), 1661–1663. https://doi.org/10.1021/jo00059a010.

(223) Spanedda, M. V.; Ourévitch, M.; Crousse, B.; Bégué, J. P.; Bonnet-Delpon, D. Vinylogous Mannich Reactions. Additions of Trimethylsilyloxyfuran to Fluorinated Aldimines. *Tetrahedron Lett.* **2004**, *45* (26), 5023–5025. https://doi.org/10.1016/j.tetlet.2004.05.003.

(224) Darabantu, M.; Maiereanu, C.; Silaghi-Dumitrescu, I.; Toupet, L.; Condamine, E.; Ramondenc, Y.; Berghian, C.; Plé, G.; Plé, N. 3,7-Dioxa-1-Azabicyclo[3.3.0]Octanes Substituted at the C-5 Position - From Local to Global Stereochemistry. *Eur. J. Org. Chem.* **2004**, No. 12, 2644–2661. https://doi.org/10.1002/ejoc.200300584.

(225) Gharpure, S. J.; Shelke, Y. G. Cascade Radical Cyclization of N-Propargylindoles: Substituents Dictate Stereoselective Formation of N-Fused Indolines versus Indoles. *Org. Lett.* **2017**, *19* (19), 5022–5025. https://doi.org/10.1021/acs.orglett.7b02005.

(226) Pozharskii, A. F.; Degtyarev, A. V.; Ryabtsova, O. V.; Ozeryanskii, V. A.; Kletskii, M. E.; Starikova, Z. A.; Sobczyk, L.; Filarowski, A. 2-α-Hydroxyalkyl- and 2,7-Di(α-Hydroxyalkyl)-1,8- Bis(Dimethylamino)Naphthalenes: Stabilization of Nonconventional in/out Conformers of "Proton Sponges" via N···H-O Intramolecular Hydrogen Bonding. A Remarkable Kind of Tandem Nitrogen Inversion. *J. Org. Chem.* **2007**, *72* (8), 3006–3019. https://doi.org/10.1021/jo062667v.

(227) Raj, A. S. K.; Kale, B. S.; Mokar, B. D.; Liu, R. S. Gold-Catalyzed N, O-Functionalizations of 6-Allenyl-1-Ynes with n-Hydroxyanilines to Construct Benzo[b]-Azepin-4-One Cores. *Org. Lett.* **2017**, *19* (19), 5340–5343. https://doi.org/10.1021/acs.orglett.7b02629.

(228) Butti, P.; Rochat, R.; Sadow, A. D.; Togni, A. PalladIum-Catalyzed Enantioselective Allylic Phosphination. *Angew. Chem. Int. Ed.* **2008**, *47* (26), 4878–4881. https://doi.org/10.1002/anie.200801287.

(229) Sudhakar, G.; Bayya, S.; Kadam, V. D.; Nanubolu, J. B. Total Synthesis of Gonytolides C and G, Lachnone C, and Formal Synthesis of Blennolide C and Diversonol. *Org. Biomol. Chem.* **2014**, *12* (30), 5601–5610. https://doi.org/10.1039/c4ob00950a.

(230) Birbaum, F.; Neels, A.; Bochet, C. G. Photochemistry of Allenyl Salicylaldehydes. *Org. Lett.* **2008**, *10* (15), 3175–3178. https://doi.org/10.1021/ol800806a.

(231) Kiselev, V. D.; Anikin, O. V.; Kornilov, D. A.; Kolesnikova, A. O.; Shulyatev, A. A.; Sedov, I. A.; Gubaidullin, A. T. Pressure, Temperature, and Solvent Effects on the Rates of Reactions of 3,4-Dihydro-2H-Pyran with Tetracyanoethylene and 4-Phenyl-1,2,4-Triazoline-3,5-Dione. *Russ. Chem. Bull.* **2019**, *68* (2), 351–356. https://doi.org/10.1007/s11172-019-2392-8.

(232) Hou, H.; Zhu, S.; Pan, F.; Rueping, M. Visible-Light Photoredox-Catalyzed Synthesis of Nitrones: Unexpected Rate Acceleration by Water in the Synthesis of Isoxazolidines. *Org. Lett.* **2014**, *16* (11), 2872–2875. https://doi.org/10.1021/ol500893g.

(233) Liu, X.; Zhou, Y.; Song, Q. Metal-Free Cyclization of Unsaturated Hydrazones for the Divergent Assembly of Pyrazolones and Pyrazolines. *Chem. Commun.* **2019**, *55* (61), 8943–8946. https://doi.org/10.1039/c9cc04039k.

(234) Liu, W.; Li, H.; Cai, P. J.; Wang, Z.; Yu, Z. X.; Lei, X. Scalable Total Synthesis of Rac-Jungermannenones B and C. *Angew. Chem. Int. Ed.* **2016**, *55* (9), 3112–3116. https://doi.org/10.1002/anie.201511659.

(235) Vystorop, I. V.; Lyssenko, K. A.; Kostyanovsky, R. G. 2-Phenyl-3-Hydroxyimidazolidin-4-One: The Regioselective Synthesis, Structure and Enantiomerically Enriched Crystallization. *Mendeleev Commun.* **2003**, *13* (3), 116–118. https://doi.org/10.1070/MC2003v013n03ABEH001790.

(236) Barakat, A.; Al-Majid, A. M.; Ali, M.; Ghabbour, H. A.; Al-Marashdah, M. S.; Siddiqui, M. R. Crystal Structure of 6-Hydroxy-5-((2-Hydroxy-6-Oxocyclohex-1-En-1-Yl)(Phenyl)Methyl)-1,3-Dimethylpyrimidine-2,4(1H,3H)-Dione, C19H20N2O5. *Zeitschrift fur Krist. New Cryst. Struct.* **2016**, *231* (3), 849–851. https://doi.org/10.1515/ncrs-2015-0290.

(237) Kieć-Kononowicz, K.; Stadnicka, K.; Mitka, A.; Pekala, E.; Filipek, B.; Sapa, J.; Zygmunt, M. Synthesis, Structure

and Antiarrhythmic Properties Evaluation of New Basic Derivatives of 5,5-Diphenylhydantoin. *Eur. J. Med. Chem.* **2003**, *38* (6), 555–566. https://doi.org/10.1016/S0223-5234(03)00075-8.

(238) Alhadi, A. A.; Othman, R.; Yehye, W. A.; Rahman, N. A. Formation of 1,3,4-Oxadiazolines and 1,3,4-Oxadiazepines through Acetylation of Salicylic Hydrazones. *Tetrahedron Lett.* **2015**, *56* (4), 573–576. https://doi.org/10.1016/j.tetlet.2014.12.037.

(239) Ignatenko, V. A.; Deligonul, N.; Viswanathan, R. Branch-Selective Synthesis of Oxindole and Indene Scaffolds: Transition Metal-Controlled Intramolecular Aryl Amidation Leading to C3 Reverse-Prenylated Oxindoles. *Org. Lett.* **2010**, *12* (16), 3594–3597. https://doi.org/10.1021/ol1012372.

(240) Földi, Á. A.; Ludányi, K.; Bényei, A. C.; Mátyus, P. Tert -Amino Effect in Peri -Substituted Naphthalenes: Syntheses of Naphthazepine and Naphthazonine Ring Systems. *Synlett* **2010**, *2010* (14), 2109–2113. https://doi.org/10.1055/s-0030-1258536.

(241) Lim, W. T.; Cui, J. Z.; Suh, H. J.; Lee, H. S.; Heo, N. H.; Kim, S. H. Synthesis and X-Ray Structural Characterization of New Spiroxazine. *Dye. Pigment.* **2003**, *56* (1), 7–15. https://doi.org/10.1016/S0143-7208(02)00111-0.

(242) Takai, T.; Koike, T.; Nakamura, M.; Kajita, Y.; Yamashita, T.; Taya, N.; Tsukamoto, T.; Watanabe, T.; Murakami, K.; Igari, T.; Kamata, M. Discovery of Novel 5,6,7,8-Tetrahydro[1,2,4]Triazolo[4,3-a]Pyridine Derivatives as γ-Secretase Modulators (Part 2). *Bioorganic Med. Chem.* **2016**, *24* (14), 3192–3206. https://doi.org/10.1016/j.bmc.2016.05.040.

(243) Utz, D.; Kisslinger, S.; Heinemann, F. W.; Hampel, F.; Schindler, S. Syntheses, Characterization and Properties of Open-Chain Copper(I) Complexes. *Eur. J. Inorg. Chem.* **2011**, *2011* (2), 255–267. https://doi.org/10.1002/ejic.201000954.

(244) Nair, V.; Mathai, S.; Nair, S. M.; Rath, N. P. A Facile Three-Component Reaction of Dicarbomethoxycarbene, Aldehydes and o-Quinones: Synthesis of Novel Spiro-Dioxolanes. *Tetrahedron Lett.* **2003**, *44* (46), 8407–8409. https://doi.org/10.1016/j.tetlet.2003.09.102.

(245) Tona, V.; Ruider, S. A.; Berger, M.; Shaaban, S.; Padmanaban, M.; Xie, L. G.; González, L.; Maulide, N. Divergent Ynamide Reactivity in the Presence of Azides-an Experimental and Computational Study. *Chem. Sci.* **2016**, *7* (9), 6032–6040. https://doi.org/10.1039/c6sc01945e.

(246) DiMartino, G.; Hursthouse, M. B.; Light, M. E.; Percy, J. M.; Spencer, N. S.; Tolley, M. Syntheses of Selectively Fluorinated Cyclodecenones: The First Deployment of the Neutral Oxy-Cope Rearrangement in Organofluorine Chemistry. *Org. Biomol. Chem.* **2003**, *1* (24), 4423–4434. https://doi.org/10.1039/b311261f.

(247) Argay, G.; Kálmán, A.; Bernáth, G.; Gyarmati, Z. C. Cis-8-Azabicyclo[5.2.0]Nonan-9-One. *Acta Cryst. E* **2004**, *60* (2), o173–o175. https://doi.org/10.1107/S160053680302796X.

(248) Huo, F.; Yin, C.; Guo, W.; Xia, C. 2-[Hydroxy(2-Pyridyl)Methyl]-2-Cyclopenten-1-One: The Baylis-Hillman Adduct of 2-Pyridinecarboxaldehyde and 2-Cyclopenten-1-One. *Acta Cryst. E* **2004**, *60* (2), o204–o206. https://doi.org/10.1107/S1600536804000091.

(249) Obregón-Mendoza, M. A.; Estévez-Carmona, M. M.; Escobedo-Martínez, C.; Soriano-García, M.; Enríquez, R. G. Facile Preparation of 1,2-Diols from Chalcones: An NMR Spectroscopy and X-Ray Crystallography Study. *Int. J. Org. Chem.* **2015**, *05* (03), 137–146. https://doi.org/10.4236/ijoc.2015.53015.

(250) Egan, B. A.; Paradowski, M.; Thomas, L. H.; Marquez, R. Regiocontrolled Rearrangement of Isobenzofurans. *Org. Lett.* **2011**, *13* (8), 2086–2089. https://doi.org/10.1021/ol200498k.

(251) Ikuma, N.; Tamura, R.; Shimono, S.; Kawame, N.; Tamada, O.; Sakai, N.; Yamauchi, J.; Yamamoto, Y. Crystal Structure and Magnetic Properties of Novel Chiral Nitroxides Existing as Racemic Conglomerates. *Mendeleev Commun.* **2003**, *13* (3), 109–111. https://doi.org/10.1070/MC2003v013n03ABEH001787.

(252) Gourlay, M. D.; Kendrick, J.; Leusen, F. J. J. Predicting the Spontaneous Chiral Resolution by Crystallization of a Pair of Flexible Nitroxide Radicals. *Cryst. Growth Des.* **2008**, *8* (8), 2899–2905. https://doi.org/10.1021/cg701256e.

(253) Dannecker-Dörig, I.; Linden, A.; Heimgartner, H. Synthesis of Poly-Aib Oligopeptides and Aib-Containing Peptides via the “Azirine/Oxazolone Method”, and Their Crystal Structures. *Helv. Chim. Acta* **2011**, *94* (6), 993–1011. https://doi.org/10.1002/hlca.201100116.

(254) Gan, X. M.; Binyamin, I.; Rapko, B. M.; Fox, J.; Duesler, E. N.; Paine, R. T. Hydrogen Bonded Framework Structures Constructed from 2-(Pyridyl N-Oxide) Methylphosphonic Acid Ligands and Erbium(III). *Inorg. Chem.* **2004**, *43* (7), 2443–2448. https://doi.org/10.1021/ic0351850.

(255) Ovalles, S. R.; Hansen, J. H.; Davies, H. M. L. Thermally Induced Cycloadditions of Donor/Acceptor Carbenes. *Org. Lett.* **2011**, *13* (16), 4284–4287. https://doi.org/10.1021/ol201628d.

(256) Serguchev, Y. A.; Lourie, L. F.; Ponomarenko, M. V.; Rusanov, E. B.; Ignat'Ev, N. V. Fluorolactonization of Unsaturated Carboxylic Acids with F-TEDA-BF 4 in Ionic Liquids. *Tetrahedron Lett.* **2011**, *52* (40), 5166–5169. https://doi.org/10.1016/j.tetlet.2011.07.124.

(257) Jadhav, V. B.; Nayak, S. K.; Row, T. N. G.; Kulkarni, M. V. Synthesis, Structure and DNA Cleavage Studies of Coumarin Analogues of Tetrahydroisoquinoline and Protoberberine Alkaloids. *Eur. J. Med. Chem.* **2010**, *45* (9), 3575–3580. https://doi.org/10.1016/j.ejmech.2010.04.041.

(258) Sriramurthy, V.; Kwon, O. Diphosphine-Catalyzed Mixed Double-Michael Reaction: A Unified Synthesis of Lndolines, Dihydropyrrolopyridines, Benzimidazolines, Tetrahydroquinolines, Tetrahydroisoquinolines, Dihydrobenzo-1,4-Oxazines, and Dihydrobenzo-3,1- Oxazines. *Org. Lett.* **2010**, *12* (5), 1084–1087. https://doi.org/10.1021/ol100078w.

(259) Geraschenko, O. V.; Khodakovskiy, P. V.; Shivanyuk, O. N.; Shishkin, O. V.; Mykhailiuk, P. K.; Tolmachev, A. A. Easy Synthesis of Novel 4-Azolylpyridazin-3-Ones. *Synthesis* **2012**, *44* (8), 1263–1267. https://doi.org/10.1055/s-0031-1290808.

(260) Appel, R.; Kündgen, U.; Knoch, F. Niederkoordinierte Phosphorverbindungen, 30. 1-Chlor-2-phenyl-2-(Trimethylsilyl)-1-phosphaethen Als Synthese-baustein Neuer Phosphaalkene. *Chem. Ber.* **1985**, *118* (4), 1352–1370. https://doi.org/10.1002/cber.19851180407.

(261) Ishikawa, M.; Sugisawa, H.; Akitomo, H.; Matsusaki, K.; Kamitori, S.; Hirotsu, K.; Higuchi, T. Formation and Reactions of 1-Silacyclobut-2-En-1-Ylidene and Molecular Structures of Methanol and Diene Adducts. *Organometallics* **1986**, *5* (12), 2447–2451. https://doi.org/10.1021/om00143a007.

(262) Lee, G. A.; Chang, C. Y. Novel Ene Trimerization of 1-Phenylcyclopropene. *J. Org. Chem.* **2004**, *69* (25), 8949–8951. https://doi.org/10.1021/jo048889f.

(263) Rappoport, Z.; Gazit, A. Nucleophilic Attacks on Carbon-Carbon Double Bonds. 33. Approaching the Retention Region from the Stereoconvergence Region in Nucleophilic Substitution of (E)- and (Z)-Methyl p-Substituted a-Formyl- and α-(Fert-Butoxycarbonyl)-β-Halocinnamates. *J. Org. Chem.* **1986**, *51* (22), 4112–4131. https://doi.org/10.1021/jo00372a005.

(264) Ma, C.; Chen, J.; Xing, D.; Sheng, Y.; Hu, W. Iron Catalyzed Efficient Synthesis of Poly-Functional Primary Amines via the Direct Use of Ammonia. *Chem. Commun.* **2017**, *53* (19), 2854–2857. https://doi.org/10.1039/c7cc00003k.

(265) Fogh, R. H.; Larsen, S.; Dahl, O. Structure of 1-Dimethylamino-3-Methyl-3H-2,1λ5-Benzoxaphosphole 1-Sulfide at 105 K. *Acta Cryst. C* **1986**, *42* (11), 1635–1637. https://doi.org/10.1107/s0108270186091199.

(266) Wladislaw, B.; Bueno, M. A.; Marzorati, L.; Di Vitta, C.; Zukerman-Schpector, J. Phase Transfer Catalysis (PTC) Sulfanylation of Some 2-Methylsulfinyl- Cyclanones. *J. Org. Chem.* **2004**, *69* (26), 9296–9298. https://doi.org/10.1021/jo048751x.

(267) Şahin, Z. S.; Salgin-Gökşen, U.; Gökhan-Kelekçi, N.; Işik, Ş. Synthesis, Crystal Structures and DFT Studies of 1-[2-(5-Methyl-2- Benzoxazolinone-3-Yl)Acetyl]-3-Phenyl-5-(3,4-Dimethoxyphenyl)-4, 5-Dihydro-1H-Pyrazole and 1-[2-(5-Chloro-2-Benzoxazolinone-3-Yl)Acetyl]-3- Phenyl-5-(4-Methoxyphenyl)-4,5-Dihydro-1H-Pyrazo. *J. Mol. Struct.* **2011**, *1006* (1–3), 147–158. https://doi.org/10.1016/j.molstruc.2011.08.061.

(268) Haraguchi, R.; Takada, Y.; Matsubara, S. Rapid Preparation of Cycloheptane Ring from 1,2-Diketone and Bis(Iodozincio)Methane via Oxy-Cope Rearrangement Using Microflow System. *Chem. Lett.* **2012**, *41* (6), 628–629. https://doi.org/10.1246/cl.2012.628.

(269) Chan, C. K.; Hsueh, N. C.; Tsai, Y. L.; Chang, M. Y. Bi(OTf)3-Mediated Intramolecular Olefinic Cyclization: Synthesis of Substituted Aryl-Dihydronaphthalenes and Indenes. *J. Org. Chem.* **2017**, *82* (13), 7077–7084. https://doi.org/10.1021/acs.joc.7b01278.

(270) Valente, E. J.; Eggleston, D. S.; Schomaker, V. Structures of Five Trans-2-Hydroxy and Trans-2-Methoxy-2-Methyl-3,4-Dihydro-4-Aryl-2H,5H-Pyrano[3,2-c]Benzopyran-5-Ones. *Acta Cryst. C* **1986**, *42* (12), 1809–1813. https://doi.org/10.1107/s0108270186090455.

(271) Smith, D. T.; Vitaku, E.; Njardarson, J. T. Dearomatization Approach to 2-Trifluoromethylated Benzofuran and Dihydrobenzofuran Products. *Org. Lett.* **2017**, *19* (13), 3508–3511. https://doi.org/10.1021/acs.orglett.7b01479.

(272) Ter Wiel, M. K. J.; Vicario, J.; Davey, S. G.; Meetsma, A.; Feringa, B. L. New Procedure for the Preparation of Highly Sterically Hindered Alkenes Using a Hypervalent Iodine Reagent. *Org. Biomol. Chem.* **2005**, *3* (1), 28–30. https://doi.org/10.1039/b414959a.

(273) Györgydeák, Z.; Holzer, W.; Kunz, R. W.; Linden, A. 1,4-Diacyl-3-Acylamino-5-Aryl-4,5-Dihydro-1 H-1,2,4-

Triazoles: Ring Closure Products of Aromatic Carbaldehyde (Diaminomethylene) Hydrazones with Acylating Agents. *Monatshefte für Chemie Chem. Mon.* **1995**, *126* (6–7), 733–746. https://doi.org/10.1007/BF00807164.

(274) Francis, T.; Happer, D. A. R.; Robinson, W. T. 2-Phenylselanyl-3,5-Methano-2H-Cyclo-Penta[b]Furan-2-One. *Acta Cryst. E* **2005**, *61* (2), o453–o455. https://doi.org/10.1107/S160053680500231X.

(275) Cetina, M.; Nagl, A.; Prekupec, S.; Raić-Malić, S.; Mintas, M. Hydrogen-Bonding and C - H···π Interactions in 7-Hydroxy-3-Methoxy- 4-Methyl-5,6,7,8-Tetrahydropyrido[1,2-c]Pyrimidin-1 (9H)-One. *Acta Cryst. C* **2005**, *61* (3), o158–o160. https://doi.org/10.1107/S010827010500079X.

(276) Caine, D.; Mccloskey, C. J.; Atwood, J. L.; Bott, S. G.; Zhang, H. M.; VanDerveer, D. Synthesis and Base-Induced Methylation Reactions of Cis-7a-Hydroxy-3a-(Phenylsulfenyl)-3a,4,5,6,7,7a-Hexahydro-4-Indanone. *J. Org. Chem.* **1987**, *52* (7), 1280–1284. https://doi.org/10.1021/jo00383a020.

(277) Burstein, C.; Glorius, F. Organocatalyzed Conjugate Umpolung of α,β-Unsaturated Aldehydes for the Synthesis of γ-Butyrolactones. *Angew. Chem. Int. Ed.* **2004**, *43* (45), 6205–6208. https://doi.org/10.1002/anie.200461572.

(278) Jefford, C. W.; Favarger, F.; Ferro, S.; Chambaz, D.; Bringhen, A.; Bernardinelli, G.; Boukouvalas, J. The Formation of Bridged Bicyclic 1,2,4-Trioxanes by Intramolecular Capture of Β-Hydroperoxy Cations. *Helv. Chim. Acta* **1986**, *69* (7), 1778–1786. https://doi.org/10.1002/hlca.19860690735.

(279) Akasaka, T.; Ando, W. Stereospecific Oxygenation of 3-Adamantylidenetricyclo[3.2.1.02,4]Octane: Singlet Oxygen vs. Electron-Transfer Oxygenations. *J. Am. Chem. Soc.* **1987**, *109* (4), 1260–1262. https://doi.org/10.1021/ja00238a054.

(280) Lebed, P. S.; Fenneteau, J.; Wu, Y.; Cossy, J.; Mykhailiuk, P. K. Synthesis of N-Nitroso CHF2-Pyrazolines and Their Transformation into CHF2-Isoxazolines and -Pyrazoles. *Eur. J. Org. Chem.* **2017**, *2017* (41), 6114–6120. https://doi.org/10.1002/ejoc.201700803.

(281) Kitoh, S. I.; Kubota, A.; Kunimoto, K. K.; Kuwae, A.; Hanai, K. Conglomerate Formation and Crystal Structure of 4-Phenyl-1,3-Thiazolidin-2- One. *J. Mol. Struct.* **2005**, *737* (2–3), 277–282. https://doi.org/10.1016/j.molstruc.2004.10.064.

(282) Buttrus, N. H.; Cornforth, J.; Hitchcock, P. B.; Kumar, A.; Stuart, A. S. Synthesis of Substituted Dibenzophospholes. Part 5. Synthesis of Intermediates for 4-and 6-Aryl Substituents. *J. Chem. Soc. Perkin Trans. 1* **1987**, No. 0, 851. https://doi.org/10.1039/p19870000851.

(283) Noland, W. E.; Brown, C. D.; Zabronsky, A. E.; Tritch, K. J. Synthesis of 2-(9H-Carbazol-1-Yl)Anilines from 2,3′-Biindolyl and Ketones. *Tetrahedron* **2018**, *74* (19), 2391–2404. https://doi.org/10.1016/j.tet.2018.03.066.

(284) Qian, H.; Zhao, W.; Sung, H. H. Y.; Williams, I. D.; Sun, J. Stereoselective Synthesis of Aminoindanols via an Efficient Cascade Aza-Michael–Aldol Reaction. *Chem. Commun.* **2013**, *49* (39), 4361–4363. https://doi.org/10.1039/c2cc37102b.

(285) Moustafa, M. S.; Al-Mousawi, S. M.; Elnagdi, M. H. Simple Efficient Routes for the Preparation of Pyrazoleamines and Pyrazolopyrimidines: Regioselectivity of Pyrazoleamines Reactions with Bidentate Reagents. *Croat. Chem. Acta* **2016**, *89* (1), 71–79. https://doi.org/10.5562/cca2738.

(286) Basavaiah, D.; Lingam, H.; Babu, T. H. Baylis-Hillman Acetates in Organic Synthesis: A Simple Two-Step Strategy for Oxindole-Spiro-α-Arylidene-γ-Butyrolactone Framework. *Tetrahedron* **2018**, *74* (19), 2306–2313. https://doi.org/10.1016/j.tet.2018.03.035.

(287) Takamatsu, N.; Aiba, S.; Yamada, T.; Tokunaga, Y.; Kawasaki, T. Highly Stereoselective Strecker Synthesis Induced by a Slight Modification of Benzhydrylamine from Achiral to Chiral. *Chem. Eur. J.* **2018**, *24* (6), 1304–1310. https://doi.org/10.1002/chem.201704033.

(288) Zhang, H.; Lu, Z. Nickel/Copper Dual Catalysis for Sequential Nazarov Cyclization/Decarboxylative Aldol Reaction. *Org. Lett.* **2018**, *20* (18), 5709–5713. https://doi.org/10.1021/acs.orglett.8b02426.

(289) Duarah, G.; Kaishap, P. P.; Sarma, B.; Gogoi, S. Ruthenium(II)-Catalyzed Dearomatized C−H Activation and Annulation Reaction of Vinylnaphthols with Alkynes: Access to Spiro-Pentacyclic Naphthalenones. *Chem. Eur. J.* **2018**, *24* (40), 10196–10200. https://doi.org/10.1002/chem.201801537.

(290) Huang, J. W.; Shi, M. Carbolithiation of Gem-Aryl Disubstituted Methylenecyclopropanes. *Org. Biomol. Chem.* **2005**, *3* (3), 399–400. https://doi.org/10.1039/b417139j.

(291) Watanabe, K.; Hamada, T.; Moriyama, K. Ring-Contraction Reaction of Substituted Tetrahydropyrans via Dehydrogenative Dual Functionalization by Nitrite-Catalyzed Double Activation of Bromine. *Org. Lett.* **2018**, *20* (18), 5803–5807. https://doi.org/10.1021/acs.orglett.8b02488.

(292) Kálman, A.; Argay, G. Y.; Sohár, P.; Szabó, J.; Fodor, L.; Bernáth, G. 1H and 13C NMR and X-Ray Studies of 2- and

4-Phenyl-6,7-Dimethoxy-3methyl-3,4-Dihydro-2H-1,3-Benzothiazines [1]. *J. Mol. Struct.* **1986**, *145* (3–4), 341–354. https://doi.org/10.1016/0022-2860(86)85038-4.

(293) Degennaro, L.; Zenzola, M.; Trinchera, P.; Carroccia, L.; Giovine, A.; Romanazzi, G.; Falcicchio, A.; Luisi, R. Regioselective Functionalization of 2-Arylazetidines: Evaluating the Ortho-Directing Ability of the Azetidinyl Ring and the α-Directing Ability of the N-Substituent. *Chem. Commun.* **2014**, *50* (14), 1698–1700. https://doi.org/10.1039/c3cc48555b.

(294) Shchegol'Kov, E. V.; Ivanova, A. E.; Burgart, Y. V.; Saloutin, V. I. A Convenient Approach to 4,7-Dihydrotetrazolo [5,1-c][1,2,4]Triazine Synthesis. *J. Heterocycl. Chem.* **2013**, *50*, 80. https://doi.org/10.1002/jhet.1068.

(295) Hoffmann, K. L.; Maas, G.; Regitz, M. Oxepines from Pyrylium Salts and Diazo Esters. Cycloaddition Behavior toward 4-Phenyl-l,2,4-Triazoline-3,5-Dione. *J. Org. Chem.* **1987**, *52* (17), 3851–3857. https://doi.org/10.1021/jo00226a024.

(296) Noland, W. E.; Huisenga, M. P.; Herzig, R. J.; Rosenow, J. A.; Kim, H.; Kroll, N. J.; Nesmelov, A.; Johnson, B. T.; Duncan, N. S.; Ratanayanon, J.; Yue, R. A.; Xiong, K.; Ong, B. K.; Vo, D. T.; Klein, N. D.; Lang, S. B.; Riley, J. K.; Daniels, S. P.; Tritch, K. J. A Diels–Alder/Ene Cascade Leading to 5-(Pyrrolidin-3-Yl)Thieno[3,2-e]Isoindoles from Ketone-Derived 2-Vinylthiophenes and N-Phenylmaleimide. *J. Heterocycl. Chem.* **2018**, *55* (12), 2698–2714. https://doi.org/10.1002/jhet.3327.

(297) Norton Matos, M.; Afonso, C. A. M.; Batey, R. A. Synthesis of Substituted Pyrrolidines and Piperidines from Endocyclic Enamine Derivatives. Synthesis of (±)-Laburnamine. *Tetrahedron* **2005**, *61* (5), 1221–1244. https://doi.org/10.1016/j.tet.2004.11.035.

(298) Bredikhin, A. A.; Zakharychev, D. V.; Gubaidullin, A. T.; Fayzullin, R. R.; Pashagin, A. V.; Bredikhina, Z. A. Crystallization Features of the Chiral Drug Timolol Precursor: The Rare Case of Conglomerate with Partial Solid Solutions. *Cryst. Growth Des.* **2014**, *14* (4), 1676–1683. https://doi.org/10.1021/cg4017905.

(299) Wang, Q.; Hu, J.; Zheng, N. A Photocatalyzed Cascade Approach Toward the Tetracyclic Core of Akuammiline Alkaloids. *Org. Lett.* **2019**, *21* (3), 614–617. https://doi.org/10.1021/acs.orglett.8b03648.

(300) Talebizadeh, M.; Anary-Abbasinejad, M.; Darehkordi, A. A Simple One-Pot Three-Component Synthesis of Dihydrobenzo[4,5]Imidazo[2,1-b]Thiazol-3-Ols by Reaction of Acyl Chlorides, Isocyanides, and 2-Mercaptobenzimidazoles. *J. Heterocycl. Chem.* **2018**, *55* (12), 2737–2743. https://doi.org/10.1002/jhet.3335.

(301) He, Y.; Feng, T.; Fan, X. Synthesis of Functionalized Indole-1-Oxide Derivatives via Cascade Reactions of Allenynes and TBuONO. *Org. Lett.* **2019**, *21* (11), 3918–3922. https://doi.org/10.1021/acs.orglett.9b00968.

(302) Kandur, W. V.; Richert, K. J.; Rieder, C. J.; Thomas, A. M.; Hu, C.; Ziller, J. W.; Woerpel, K. A. Synthesis and Reactivity of 1,2-Dioxolanes from β,γ-Epoxy Ketones. *Org. Lett.* **2014**, *16* (10), 2650–2653. https://doi.org/10.1021/ol500835f.

(303) Jevric, M.; Taylor, D. K.; Greatrex, B. W.; Tiekink, E. R. T. DDQ Induced Oxidative Cyclisations of 1,2-Dihydronaptho[2,1-b]Furans. *Tetrahedron* **2005**, *61* (7), 1885–1891. https://doi.org/10.1016/j.tet.2004.12.010.

(304) Baert, F.; Muller, M.; Barbry, D.; Couturier, D. Structural Studies of 2-phenylthiazolidine. *Acta Cryst. B* **1987**, *43* (6), 538–543. https://doi.org/10.1107/S0108768187097350.

(305) Liu, K.; Teng, H. L.; Wang, C. J. Et3N-Catalyzed Tandem Formal [4 + 3] Annulation/Decarboxylation/Isomerization of Methyl Coumalate with Imine Esters: Access to Functionalized Azepine Derivatives. *Org. Lett.* **2014**, *16* (17), 4508–4511. https://doi.org/10.1021/ol5020569.

(306) Hijfte, L. Van; Little, R. D.; Petersen, J. L.; Moeller, K. D. Intramolecular 1,3-Diyl Trapping Reactions. Total Synthesis of (i)-Hypnophilin and (±)-Coriolin. Formation of The Trans-Fused Bicyclo[3. 3.0]Octane Ring System. *J. Org. Chem.* **1987**, *52* (21), 4647–4661. https://doi.org/10.1021/jo00230a001.

(307) Hart, D. J.; Hong, W. pyo; Hsu, L. yeh. Total Synthesis of (±)-Lythrancepine II and (±)-Lythrancepine III. *J. Org. Chem.* **1987**, *52* (21), 4665–4673. https://doi.org/10.1021/jo00230a003.

(308) Aravindhan, S.; Ponnuswamy, S.; Jamesh, M.; Ramesh, P.; Ponnuswamy, M. N. 1-Acetyl-c-3,t-3-Dimethyl-r-2,c-6-Diphenyl-Piperidin-4-One. *Acta Cryst. E* **2009**, *65* (8), o1974–o1974. https://doi.org/10.1107/S1600536809028049.

(309) FU, Y.; FU, H. gang; YE, F.; WEN, X. tian; JIANG, L. xue. Synthesis, Crystal Structure and Biological Activity of Novel Dichloroacetyl Oxazolidine Herbicide Safeners. *Chem. Res. Chinese Univ.* **2008**, *24* (3), 291–294. https://doi.org/10.1016/S1005-9040(08)60062-8.

(310) Dabroswki, Z.; Wróbel, J. T.; Andreetti, G. D. Structure and Stereochemistry of Lactames and Cyclic Imides. Part VII. Synthesis and Stereochemistry of 5,6-Distributed Piperidin-2-Ones. *J. Mol. Struct.* **1986**, *145* (3–4), 319–

330. https://doi.org/10.1016/0022-2860(86)85036-0.

(311) Li, B.; Wang, Z. X.; Xing, Z.; Chen, L. Z.; Han, G. F. Synthesis of Novel 2-Methyl and 2-Cyanomethyl-12-Aryl-8,12-Dihydro-9Hchromeno[3,2-e][1,2,4]Triazolo[1,5-c]Pyrimidin-11(10H)-One Derivatives. *J. Chem. Res.* **2015**, *39* (1), 30–35. https://doi.org/10.3184/174751915X14190040330104.

(312) Chu, S. S. C.; Napoleone, V.; Chu, T. L. Crystal Structures of 2,4-dimethylthioxanthene 10,10-dioxide, 2,4,9-trimethylthioxanthene 10,10-dioxide, and 2,4-dimethyl-9-isopropylthioxanthene 10,10-dioxide. *J. Heterocycl. Chem.* **1987**, *24* (1), 143–148. https://doi.org/10.1002/jhet.5570240127.

(313) Declercq, J. P.; Carretero, J. C.; Nemery, I. Structures of 9,9-Dimethoxy-1-Methyl-7-Phenylsulfonylbicyclo[4.3.0]Nonan-2-One (I) and 10,10-Dimethoxy-8-Phenylsulfonylbicyclo[5.3.0]Decan-2-One (II). *Acta Cryst. C* **1987**, *43* (11), 2146–2148. https://doi.org/10.1107/s0108270187088693.

(314) Paquette, L. A.; Schaefer, A. G.; Springer, J. P. Synthesis of (±)-14-Epiupial by Manganese(III) -γ-Lactone Annulation. *Tetrahedron* **1987**, *43* (23), 5567–5582. https://doi.org/10.1016/S0040-4020(01)87738-3.

(315) He, L.; Chen, L. M. Trimethyl 5-(2-Chloro-4-Fluoro-Phen-Yl)-2-Phenyl-Pyrrolidine-2,3,4- Tricarboxyl-Ate. *Acta Cryst. E* **2009**, *65* (11), o2927–o2927. https://doi.org/10.1107/S1600536809044274.

(316) Zhi-Guang, X.; Hai-Yang, L.; Guo-Bang, G.; Xuan, X.; Yun-Xiu, Z. Benzyl 2-Ethyl-Hexyl Sulfoxide. *Acta Cryst. E* **2009**, *65* (11), o2929–o2929. https://doi.org/10.1107/S1600536809044328.

(317) Cosstick, K. B.; Drew, M. G. B.; Gillbert, A. Specific Intramolecular Ortho Photocycloaddition of Substituted 4-Phenoxybut-1-Enes. *J. Chem. Soc. Chem. Commun.* **1987**, No. 24, 1867–1868. https://doi.org/10.1039/C39870001867.

(318) Olmstead, M. M.; Sampath, V.; Schore, N. E. Structure of an Oxatriquinane: Cis,Anti,Cis-7b-Methylperhydrodicyclopenta[b,d]Furan-1,6-Dione. *Acta Cryst. C* **1987**, *43* (10), 1939–1941. https://doi.org/10.1107/s0108270187089546.

(319) Barton, J. W.; Howard, J. A. K.; Shepherd, M. K.; Stringer, A. M. The Rearrangement of a Tetrahydrobiphenylene Derivative to a Bridged Benzocycloheptene. *J. Chem. Soc. Perkin Trans. 1* **1987**, *84* (2), 2443–2445. https://doi.org/10.1039/p19870002443.

(320) Dobbs, A. P.; Guesné, S. J. J.; Parker, R. J.; Skidmore, J.; Stephenson, R. A.; Hursthouse, M. B. A Detailed Investigation of the Aza-Prins Reaction. *Org. Biomol. Chem.* **2010**, *8* (5), 1064–1080. https://doi.org/10.1039/b915797b.

(321) Fan, Z.; Chen, L. Structures of 1-Phenyl-2,3,4-Tris(Trifluoromethyl)Pyrrolo[3,2-c]Quinoline (1) and 2-Fluoro-3-Pentafluoroethyl-1-Phenyl-2,3,4-Tris(Trifluoromethyl)-2,3-Dihydropyrrolo[3,2-c]Quinoline (2). *Acta Cryst. C* **1987**, *43* (11), 2206–2209. https://doi.org/10.1107/s0108270187088437.

(322) Rassadin, V. A.; Grosheva, D. S.; Tomashevskiy, A. A.; Sokolov, V. V.; Yufit, D. S.; Kozhushkov, S. I.; De Meijere, A. Bicyclic Sultams with a Nitrogen at the Bridgehead and a Sulfur Atom in the Apex Position: Facile Preparation and Conformational Properties. *Eur. J. Org. Chem.* **2010**, *2010* (18), 3481–3486. https://doi.org/10.1002/ejoc.201000345.

(323) Gandhamsetty, N.; Park, S.; Chang, S. Selective Silylative Reduction of Pyridines Leading to Structurally Diverse Azacyclic Compounds with the Formation of Sp3 C-Si Bonds. *J. Am. Chem. Soc.* **2015**, *137* (48), 15176–15184. https://doi.org/10.1021/jacs.5b09209.

(324) Huple, D. B.; Mokar, B. D.; Liu, R. S. Alkene-Directed N-Attack Chemoselectivity in the Gold-Catalyzed [2+2+1]-Annulations of 1,6-Enynes with N-Hydroxyanilines. *Angew. Chem. Int. Ed.* **2015**, *54* (49), 14924–14928. https://doi.org/10.1002/anie.201507946.

(325) Hansmann, M. M.; Melen, R. L.; Rudolph, M.; Rominger, F.; Wadepohl, H.; Stephan, D. W.; Hashmi, A. S. K. Cyclopropanation/Carboboration Reactions of Enynes with B(C6F5)3. *J. Am. Chem. Soc.* **2015**, *137* (49), 15469–15477. https://doi.org/10.1021/jacs.5b09311.

(326) Inhülsen, I.; Schmidt, K.; Margaretha, P. Photocycloaddition of Conjugated Cyclohex-2-Enones to 2,3-Dimethylbuta-1,3- Diene. *Helv. Chim. Acta* **2010**, *93* (6), 1052–1057. https://doi.org/10.1002/hlca.201000042.

(327) Yadav, J. S.; Borkar, P.; Pawan Chakravarthy, P.; Subba Reddy, B. V.; Sarma, A. V. S.; Basha, S. J.; Sridhar, B.; Grée, R. Versatile Intramolecular Aza-Prins and Prins Cyclization of Aryl Epoxides: A Facile Synthesis of Diaza-, Oxa-Aza-, and Dioxa-Bicycles. *J. Org. Chem.* **2010**, *75* (6), 2081–2084. https://doi.org/10.1021/jo902683p.

(328) Bermejo, E.; Castiñeiras, A.; West, D. X. Crystal and Molecular Structure of Δ6-5,6-Diphenyl-5-Methoxy-1,2,4-Triazacyclohexene-3-Thione and Δ6-4-Methyl-5,6-Diphenyl-5-Ethoxy-1,2, 4-Triazacyclohexene-3-Thione. *J. Mol. Struct.* **2003**, *650* (1–3), 93–97. https://doi.org/10.1016/S0022-2860(03)00076-0.

(329) Schneider, T. F.; Kaschel, J.; Awan, S. I.; Dittrich, B.; Werz, D. B. From Furan to Molecular Stairs: Syntheses,

Structural Properties, and Theoretical Investigations of Oligocyclic Oligoacetals. *Chem. Eur. J.* **2010**, *16* (37), 11276–11288. https://doi.org/10.1002/chem.201000468.

(330) Mauleón, P.; Núñez, A. A.; Alonso, I.; Carretero, J. C. Palladium-Catalyzed Cascade Reaction of α,β-Unsaturated Sulfones with Aryl Iodides. *Chem. Eur. J.* **2003**, *9* (7), 1511–1520. https://doi.org/10.1002/chem.200390173.

(331) Grillon, E.; Gallo, R.; Pierrot, M.; Boileau, J.; Wimmer, E. Isolation and X-Ray Structure of the Intermediate Dihydroxyimidazolidine(DHI) in the Synthesis of Glycoluril from Glyoxal and Urea. *Tetrahedron Lett.* **1988**, *29* (9), 1015–1016. https://doi.org/10.1016/0040-4039(88)85322-X.

(332) Kaga, A.; Peng, X.; Hirao, H.; Chiba, S. Diastereo-Divergent Synthesis of Saturated Azaheterocycles Enabled by TBuOK-Mediated Hydroamination of Alkenyl Hydrazones. *Chem. Eur. J.* **2015**, *21* (52), 19112–19118. https://doi.org/10.1002/chem.201504160.

(333) Öztürk, S.; Akkurt, M.; Tepe, E.; Heinemann, F. W.; Altundaş, A.; Kara, Y. 1-(12-Benzoyl-10-Oxa-12-Azatetracyclo-[6.3.1.02,7.0 9,11]Dodeca-2,4,6-Trien-1-Yl)Ethan-1-One. *Acta Cryst. E* **2003**, *59* (5), 635–637. https://doi.org/10.1107/S1600536803007827.

(334) Shtelzer, S.; Sheradsky, T.; Blum, J. The Chemistry of Polycyclic Arene Imines. VII. Reactions of Phenanthrene 9,10-imine with Aromatic Carboxaldehydes, Carboxylic Acids and Acetylenic Esters. *J. Heterocycl. Chem.* **1987**, *24* (6), 1581–1585. https://doi.org/10.1002/jhet.5570240615.

(335) Do Nascimento, J. P.; Santos, L. S.; Santos, R. H. A.; Tozzo, É.; Ferreira, J. G.; Do Carmo, M. C. L.; Brasil, D. S. B.; Alves, C. N. Synthesis, X-Ray Crystal Structure and Theoretical Calculations of Antileishmanial Neolignan Analogues. *J. Braz. Chem. Soc.* **2010**, *21* (10), 1825–1837. https://doi.org/10.1590/s0103-50532010001000006.

(336) Gazizov, A. S.; Smolobochkin, A. V.; Voronina, J. K.; Burilov, A. R.; Pudovik, M. A. Acid-Catalyzed Reaction of (4,4-Diethoxybutyl)Ureas with Phenols as a Novel Approach to the Synthesis of α-Arylpyrrolidines. *Synth. Commun.* **2015**, *45* (10), 1215–1221. https://doi.org/10.1080/00397911.2015.1011340.

(337) Van Berkom, L. W. A.; Kuster, G. J. T.; De Gelder, R.; Scheeren, H. W. Synthesis and Rearrangement of N-Organyloxy β-Lactams Derived from a (4+2)/(3+2) Sequential Cycloaddition Reaction Involving Enol Ethers and Nitro Alkenes. *Eur. J. Org. Chem.* **2004**, *2004* (21), 4397–4404. https://doi.org/10.1002/ejoc.200400371.

(338) Armstrong, A.; Edmonds, I. D.; Swarbrick, M. E.; Treweeke, N. R. Electrophilic Amination of Enolates with Oxaziridines: Effects of Oxaziridine Structure and Reaction Conditions. *Tetrahedron* **2005**, *61* (35), 8423–8442. https://doi.org/10.1016/j.tet.2005.06.085.

(339) Yamguchi, K.; Eto, M.; Higashi, K.; Yoshitake, Y.; Harano, K. DABCO-Triggered Mild Cascade Reaction of Electron-Deficient Cyclopentadienone: Facile and Efficient Synthesis of Condensed Carbocycles. *Tetrahedron Lett.* **2011**, *52* (46), 6082–6085. https://doi.org/10.1016/j.tetlet.2011.09.002.

(340) Cheng, X.; Zhu, L.; Lin, M.; Chen, J.; Huang, X. Rapid Access to Cyclopentadiene Derivatives through Gold-Catalyzed Cycloisomerization of Ynamides with Cyclopropenes by Preferential Activation of Alkenes over Alkynes. *Chem. Commun.* **2017**, *53* (26), 3745–3748. https://doi.org/10.1039/c7cc01368j.

(341) Olszewska, T.; Milewska, M. J.; Gdaniec, M.; Połoński, T. Spontaneous Generation of Chirality and Chiroptical Spectra of N-Nitroso-2,4-Diaryl-3-Azabicyclo[3.3.1]Nonanes. *Tetrahedron Asymmetry* **2012**, *23* (3–4), 278–283. https://doi.org/10.1016/j.tetasy.2012.02.012.

(342) Hartshorn, M. P.; Readman, J. M.; Robinson, W. T.; Sies, C. W.; Wright, G. J. The Nitration of 3, 4, 5, 6-Tetramethylbenzene- 1, 2-Dicarbonitrile, 2, 3, 5, 6-Tetramethylbenzonitrile, 1, 2, 3-Trimethyl-4, 6-Dinitrobenzene and 1, 2, 4, 5-Tetramethyl-3, 6-Dinitrobenzene. Methyl Migrations Following Ipso-Substitution. *Aust. J. Chem.* **1988**, *41* (3), 373–386. https://doi.org/10.1071/CH9880373.

(343) Yanai, H.; Sakiyama, T.; Oguchi, T.; Taguchi, T. Four Component Reaction of Aldehydes, Isocyanides, Me 3SiN 3, and Aliphatic Alcohols Catalyzed by Indium Triflate. *Tetrahedron Lett.* **2012**, *53* (25), 3161–3164. https://doi.org/10.1016/j.tetlet.2012.04.046.

(344) Logan, A. W. J.; Parker, J. S.; Hallside, M. S.; Burton, J. W. Manganese(III) Acetate Mediated Oxidative Radical Cyclizations. Toward Vicinal All-Carbon Quaternary Stereocenters. *Org. Lett.* **2012**, *14* (12), 2940–2943. https://doi.org/10.1021/ol300625u.

(345) Dong, B.; Wang, M.; Xu, C.; Feng, Q.; Wang, Y. Tuning Solid-State Fluorescence of a Twisted π-Conjugated Molecule by Regulating the Arrangement of Anthracene Fluorophores. *Cryst. Growth Des.* **2012**, *12* (12), 5986–5993. https://doi.org/10.1021/cg301055j.

(346) Kuznetsov, D. M.; Kutateladze, A. G. Step-Economical Photoassisted Diversity-Oriented Synthesis: Sustaining Cascade Photoreactions in Oxalyl Anilides to Access Complex Polyheterocyclic Molecular Architectures. *J. Am. Chem. Soc.* **2017**, *139* (46), 16584–16590. https://doi.org/10.1021/jacs.7b07598.

(347) Denmark, S. E.; Cottell, J. J. A Tandem, Nitroalkene Conjugate Addition/[3+2] Cycloaddition Approach to the Synthesis of the Pentacyclic Core of (±)-Scandine. *Adv. Synth. Catal.* **2006**, *348* (16–17), 2397–2402. https://doi.org/10.1002/adsc.200600301.

(348) Yagishita, F.; Ishikawa, H.; Onuki, T.; Hachiya, S.; Mino, T.; Sakamoto, M. Total Spontaneous Resolution by Deracemization of Isoindolinones. *Angew. Chem. Int. Ed.* **2012**, *51* (52), 13023–13025. https://doi.org/10.1002/anie.201205097.

(349) Okuma, K.; Hirano, K.; Shioga, C.; Nagahora, N.; Shioji, K. Novel Formation of Oxazepino[4,5-a]Quinolines by Tandem 1,3-Dipolar and Insertion Reaction of Quinoline N-Oxides with Arynes. *Bull. Chem. Soc. Jpn.* **2013**, *86* (5), 615–619. https://doi.org/10.1246/bcsj.20120325.

(350) Yaremenko, I. A.; Gomes, G. D. P.; Radulov, P. S.; Belyakova, Y. Y.; Vilikotskiy, A. E.; Vil, V. A.; Korlyukov, A. A.; Nikishin, G. I.; Alabugin, I. V.; Terent'Ev, A. O. Ozone-Free Synthesis of Ozonides: Assembling Bicyclic Structures from 1,5-Diketones and Hydrogen Peroxide. *J. Org. Chem.* **2018**, *83* (8), 4402–4426. https://doi.org/10.1021/acs.joc.8b00130.

(351) Malkhasian, A. Y. S.; Finch, M. E.; Nikolovski, B.; Menons, A.; Kucera, B. E.; Chavez, F. A. N,N′-Dimethylformamide-Derived Products from Catalytic Oxidation of 3-Hydroxyflavone. *Inorg. Chem.* **2007**, *46* (8), 2950–2952. https://doi.org/10.1021/ic062408o.

(352) Wang, B.; Li, M.; Xu, S.; Song, H.; Wang, B. Synthesis and Structures of 1,1′-Dinaphthopyrans. *Synthesis* **2007**, *2007* (9), 1304–1308. https://doi.org/10.1055/s-2007-965996.

(353) Jeklin, A. 済無No Title No Title No Title. *Zhurnal Strukt. Khimii* **2016**, *28* (July), 1–23.

(354) Obrech, J. -P; Schönholzer, P.; Jenny, C. J.; Prewo, R.; Heimgartner, H. The Reaction of 3-(Dimethylamino)-2H-azirines with 2,3-Pyridinedicarboximide. *Helv. Chim. Acta* **1988**, *71* (5), 1319–1327. https://doi.org/10.1002/hlca.19880710542.

(355) Dandia, A.; Jain, A. K.; Laxkar, A. K.; Bhati, D. S. A Highly Efficient Protocol for the Regio- and Stereo-Selective Synthesis of Spiro Pyrrolidine and Pyrrolizidine Derivatives by Multicomponent Reaction. *Tetrahedron Lett.* **2013**, *54* (24), 3180–3184. https://doi.org/10.1016/j.tetlet.2013.04.033.

(356) Yuan, J.; Ruan, X.; Yang, Y.; Huang, X. Study on the Electrophilic Tellurolactonization of 1,2-Allenic Phosphonates: A Facile and Efficient Synthesis of 4-(Phenyltelluro)-1,2- Oxaphosphol-3-Ene 2-Oxides. *Synlett* **2007**, *2007* (18), 2871–2874. https://doi.org/10.1055/s-2007-991082.

(357) Pailloux, S. L.; Rosario-Amorin, D.; Chakravarty, M.; Camus, J. M.; Smith, K. A.; Duesler, E. N.; Dickie, D. A.; Paine, R. T.; Klausmeyer, K. K.; Padron, D. A.; Hay, B. P.; Delmau, L. H. Synthesis and Properties of New (Phosphinoylmethyl)Pyridine N-Oxides. *Zeitschrift f?r Anorg. und Allg. Chemie* **2013**, *639* (7), 1101–1116. https://doi.org/10.1002/zaac.201300099.

(358) Minhas, H. K.; Riley, W.; Stuart, A. M.; Urbonaite, M. Activation of the Hypervalent Fluoroiodane Reagent by Hydrogen Bonding to Hexafluoroisopropanol. *Org. Biomol. Chem.* **2018**, *16* (39), 7170–7173. https://doi.org/10.1039/c8ob02236d.

(359) Hariss, L.; Barakat, Z.; Farès, F.; Roisnel, T.; Grée, R.; Hachem, A. Preparation of New Gem-Difluoro Heterocyclic-Fused 1,2,3-Triazole Derivatives. *Tetrahedron Lett.* **2019**, *60* (3), 292–296. https://doi.org/10.1016/j.tetlet.2018.12.032.

(360) Chang, M. Y.; Kung, Y. H.; Chen, S. T. BF3-OEt2/Et3SiH-Mediated Rearrangement of 4-Aryl-5,5-Diphenylazapan-4-Ols. *J. Chinese Chem. Soc.* **2007**, *54* (6), 1645–1649. https://doi.org/10.1002/jccs.200700232.

(361) Grant, P. S.; Brimble, M. A.; Furkert, D. P. Synthesis of the Bicyclic Lactone Core of Leonuketal, Enabled by a Telescoped Diels–Alder Reaction Sequence. *Chem. Asian J.* **2019**, *14* (8), 1128–1135. https://doi.org/10.1002/asia.201800903.

(362) Wales, S. M.; Rivinoja, D. J.; Gardiner, M. G.; Bird, M. J.; Meyer, A. G.; Ryan, J. H.; Hyland, C. J. T. Benzoazepine-Fused Isoindolines via Intramolecular (3 + 2)-Cycloadditions of Azomethine Ylides with Dinitroarenes. *Org. Lett.* **2019**, *21* (12), 4703–4708. https://doi.org/10.1021/acs.orglett.9b01580.

(363) Gopalaiah, K.; Tiwari, A.; Choudhary, R.; Mahiya, K. Straightforward Access to 3,4-Dihydro-2H-1,2,4-Benzothiadiazine 1,1-Dioxides and Quinazolines via Iron-Catalyzed Aerobic Oxidative Condensation of Amines. *ChemistrySelect* **2019**, *4* (18), 5200–5205. https://doi.org/10.1002/slct.201900850.

(364) Shaabani, A.; Soleimani, E.; Rezayan, A. H.; Sarvary, A.; Khavasi, H. R. Novel Isocyanide-Based Four-Component Reaction: A Facile Synthesis of Fully Substituted 3,4-Dihydrocoumarin Derivatives. *Org. Lett.* **2008**, *10* (12), 2581–2584. https://doi.org/10.1021/ol800856e.

(365) Sudhapriya, N.; Manikandan, A.; Kumar, M. R.; Perumal, P. T. Cu-Mediated Synthesis of Differentially

Substituted Diazepines as AChE Inhibitors; Validation through Molecular Docking and Lipinski's Filter to Develop Novel Anti-Neurodegenerative Drugs. *Bioorganic Med. Chem. Lett.* **2019**, *29* (11), 1308–1312. https://doi.org/10.1016/j.bmcl.2019.04.002.

(366) Tlegenov, R. T.; Pakarinen, J. M. H.; Oresmaa, L.; Ahlgrén, M.; Vainiotalo, P. Synthesis and Characterization of Some Derivatives of Lupinine and Aminolupinine. Collision Induced Dissociation Mass Spectrometry Studies of Protonated Molecules. *J. Heterocycl. Chem.* **2007**, *44* (6), 1339–1344. https://doi.org/10.1002/jhet.5570440616.

(367) Hou, H.; Tang, D.; Li, H.; Xu, Y.; Yan, C.; Shi, Y.; Chen, X.; Zhu, S. Visible-Light-Driven Chlorotrifluoromethylative and Chlorotrichloromethylative Cyclizations of Enynes. *J. Org. Chem.* **2019**, *84* (11), 7509–7517. https://doi.org/10.1021/acs.joc.9b00842.

(368) Gais, H. J.; Van Gumpel, M.; Raabe, G.; Müller, J.; Braun, S.; Lindner, H. J.; Rohs, S.; Runsink, J. Sulfonyl-Stabilized Allylic Norbornenyl and Norbornyl Carbanions: Structure and Stereoselectivity of Reaction with Electrophiles. *Eur. J. Org. Chem.* **1999**, *7* (7), 1627–1651. https://doi.org/10.1002/(SICI)1099-0690(199907)1999:7<1627::AID-EJOC1627>3.0.CO;2-9.

(369) Harmata, M.; Kahraman, M. Lewis Acid Mediated Reactions of N-Arylsulfonimidoyl Chlorides with Alkenes. Some Steric Effects of Alkene Substitution. *J. Org. Chem.* **1998**, *63* (20), 6845–6851. https://doi.org/10.1021/jo980503b.

(370) Hejda, M.; Lyčka, A.; Jambor, R.; Růžička, A.; Dostál, L. From C,N- and N,N-Chelated Chloroboranes to Substituted 1H-2,1-Benzazaboroles and 1H-Pyrrolo[1,2-c][1,3,2]Diazaborolidines: A Straightforward Route to Five-Membered Rings Containing the B-N or N-B-N Moiety. *Dalt. Trans.* **2014**, *43* (33), 12678–12688. https://doi.org/10.1039/c4dt01445f.

(371) Driess, M.; Rell, S.; Merz, K. Ungewöhnliche Reaktivität Der Silicium-Phosphor-Doppelbindung in Einem Silyliden(Fluorsilyl)Phosphan: Intramolekulare C,H-Inserierung Und Seine Umwandlung in Ein Neues Silyliden(Silyl)Phosphan. *Zeitschrift f?r Anorg. und Allg. Chemie* **1999**, *625* (7), 1119–1123. https://doi.org/10.1002/(sici)1521-3749(199907)625:7<1119::aid-zaac1119>3.3.co;2-t.

(372) Pan, B.; Liu, B.; Yue, E.; Liu, Q.; Yang, X.; Wang, Z.; Sun, W. H. A Ruthenium Catalyst with Unprecedented Effectiveness for the Coupling Cyclization of - Amino Alcohols and Secondary Alcohols. *ACS Catal.* **2016**, *6* (2), 1247–1253. https://doi.org/10.1021/acscatal.5b02638.

(373) Batey, R. A.; Simoncic, P. D.; Lin, D.; Smyj, R. P.; Lough, A. J. A Three-Component Coupling Protocol for the Synthesis of Substituted Hexahydropyrrolo[3,2-c]Quinolines. *Chem. Commun.* **1999**, No. 7, 651–652. https://doi.org/10.1039/a809614g.

(374) Cvengroš, J.; Schütte, J.; Schlörer, N.; Neudörfl, J.; Schmalz, H. G. Electrophilic Activation of Benzaldehydes through Ortho Palladation: One-Pot Synthesis of 3-Methylene-Indan-1-Ols through a Domino Allylstannylation/Heck Reaction under Neutral Conditions. *Angew. Chem. Int. Ed.* **2009**, *48* (33), 6148–6151. https://doi.org/10.1002/anie.200901837.

(375) Peraino, N. J.; Wheeler, K. A.; Kerrigan, N. J. Diastereoselective Synthesis of γ-Lactones through Reaction of Enediolates with α,β-Unsaturated Sulfoxonium Salts. *Org. Lett.* **2015**, *17* (7), 1735–1737. https://doi.org/10.1021/acs.orglett.5b00545.

(376) John, J.; Târcoveanu, E.; Jones, P. G.; Hopf, H. A Tandem Mannich Addition-Palladium Catalyzed Ring-Closing Route toward 4-Substituted-3(2H)-Furanones. *Beilstein J. Org. Chem.* **2014**, *10* (1), 1462–1470. https://doi.org/10.3762/bjoc.10.150.

(377) Auner, N.; Grasmann, M.; Herrschaft, B.; Hummer, M. Silaheterocycles 36 (1). Trichlorovinylsilane, Lithium-Tert-Butyl, and 1,3-Enynes: A Versatile Combination for the Competitive Formation of Silacyclobutanes and Silacyclobutenes. *Can. J. Chem.* **2000**, *78* (11), 1445–1458. https://doi.org/10.1139/v99-247.

(378) Li, Y.; Zhu, Y.; Qin, J.; Guo, H.; Li, L.; Wu, A. Synthesis of Self-Folded Molecular Rotors Controlled by Edge-to-Face CH/π Aromatic Interactions. *Synlett* **2009**, *2009* (12), 2028–2034. https://doi.org/10.1055/s-0029-1217528.

(379) Bromm, L. O.; Laaziri, H.; Lhermitte, F.; Harms, K.; Knochel, P. Highly Regio- and Stereoselective Thermal Migration of Organoboranes in Acyclic Molecules [7]. *Journal of the American Chemical Society*. American Chemical Society October 18, 2000, pp 10218–10219. https://doi.org/10.1021/ja001283y.

(380) Hu, Y.; Yu, C.; Ren, D.; Hu, Q.; Zhang, L.; Cheng, D. One-Step Synthesis of the Benzocyclo[Pentatoocta-]Isoindole Core. *Angew. Chem. Int. Ed.* **2009**, *48* (30), 5448–5451. https://doi.org/10.1002/anie.200901246.

(381) Hashmi, A. S. K.; Rudolph, M.; Huck, J.; Frey, W.; Bats, J. W.; Hamzić, M. Gold Catalysis: Switching the Pathway

of the Furan-Yne Cyclization. *Angew. Chem. Int. Ed.* **2009**, *48* (32), 5848–5852. https://doi.org/10.1002/anie.200900887.

(382) Rommel, M.; Fukuzumi, T.; Bode, J. W. Cyclic Ketimines as Superior Electrophiles for NHC-Catalyzed Homoenolate Additions with Broad ScoDe and Low Catalvst Loadinas. *J. Am. Chem. Soc.* **2008**, *130* (51), 17266–17267. https://doi.org/10.1021/ja807937m.

(383) Hsueh, M. L.; Huang, B. H.; Wu, J.; Lin, C. C. Synthesis, Characterization, and Catalytic Studies of Lithium Complexes: Efficient Initiators for Ring-Opening Polymerization of L-Lactide. *Macromolecules* **2005**, *38* (23), 9482–9487. https://doi.org/10.1021/ma050600o.

(384) Mathew, S.; Crandall, L. A.; Ziegler, C. J.; Hartley, C. S. Enhanced Helical Folding of Ortho-Phenylenes through the Control of Aromatic Stacking Interactions. *J. Am. Chem. Soc.* **2014**, *136* (47), 16666–16675. https://doi.org/10.1021/ja509902m.

(385) Yang, C. H.; Shen, H. J.; Wang, R. H.; Wang, J. C. 2,3,7-Triazabicyclo[3.3.0]Octenes Prepared by Tandem Cascade Reaction of Allyl Azides and Olefinic Dipolarophiles. *J. Chinese Chem. Soc.* **2002**, *49* (1), 95–102. https://doi.org/10.1002/jccs.200200016.

(386) Cui, H.; Akhmedov, N. G.; Petersen, J. L.; Wang, K. K. Synthesis of a Basket-Shaped C56H38 Hydrocarbon as a Precursor toward an End-Cap Template for Carbon [6,6]Nanotubes. *J. Org. Chem.* **2010**, *75* (6), 2050–2056. https://doi.org/10.1021/jo100132v.

(387) Shen, S. S.; Ji, S. J. Molecular Iodine Catalyzed One-Pot Aza-Diels-Alder Reaction under Solvent-Free Conditions. *Chinese J. Chem.* **2008**, *26* (5), 935–940. https://doi.org/10.1002/cjoc.200890171.

(388) Gibson, H. W.; Berg, M. A. G.; Price, T. L.; Niu, Z.; Lee, M.; Rouser, M. A.; Dickson, J. C.; Slebodnick, C. The Stereochemistry of Isoquinoline Reissert Compounds: A Unique Platform for Observation of Steric and Electronic Interactions. *Tetrahedron* **2012**, *68* (38), 8052–8067. https://doi.org/10.1016/j.tet.2012.06.008.

(389) Cheng, Z. L.; Chen, Q. Y. Difluorocarbene Chemistry: A Simple Transformation of 3,3-Gem-Difluorocyclopropenes to Cyclopropenones. *Chinese J. Chem.* **2006**, *24* (9), 1219–1224. https://doi.org/10.1002/cjoc.200690227.

(390) Villamena, F. A.; Rockenbauer, A.; Gallucci, J.; Velayutham, M.; Hadad, C. M.; Zweier, J. L. Spin Trapping by 5-Carbamoyl-5-Methyl-1-Pyrroline N-Oxide (AMPO): Theoretical and Experimental Studies. *J. Org. Chem.* **2004**, *69* (23), 7994–8004. https://doi.org/10.1021/jo049244i.

(391) Pokkuluri, P. R.; Scheffer, J. R.; Trotter, J. Crystal Structure and Photochemistry of Dimethyl 9,10-dichloro-9,10-dihydro-9,10-ethenoanthracene-11,12-dicarboxylate. *Acta Cryst. C* **1993**, *49* (11), 2014–2018. https://doi.org/10.1107/S0108270193004445.

(392) Muthukumaran, J.; Parthiban, A.; Rao, H. S. P.; Krishna, R. An X-Ray Crystallographic Study of N-Methyl-3-Nitro-4- (Nitromethyl)-4H- Chromen-2-Amine. *J. Chem. Crystallogr.* **2011**, *41* (12), 1927–1934. https://doi.org/10.1007/s10870-011-0201-9.

(393) Guerrand, H. D. S.; Adams, H.; Coldham, I. Cascade Cyclization, Dipolar Cycloaddition of Azomethine Imines for the Synthesis of Pyrazolidines. *Org. Biomol. Chem.* **2011**, *9* (22), 7921–7928. https://doi.org/10.1039/c1ob06122d.

(394) Zhu, X. Q.; Zhou, J.; Wang, C. H.; Li, X. T.; Jing, S. Actual Structure, Thermodynamic Driving Force, and Mechanism of Benzofuranone-Typical Compounds as Antioxidants in Solution. *J. Phys. Chem. B* **2011**, *115* (13), 3588–3603. https://doi.org/10.1021/jp200095g.

(395) Wang, P.; Wan, R.; Han, F.; Wang, Y. One Pot Synthesis of 1,3,4-Thiadiazole Thiazolidinone Derivatives. *J. Chem. Res.* **2009**, No. 11, 671–673. https://doi.org/10.3184/030823409X12559727242438.

(396) Chmielewski, M. K.; Tykarska, E.; Markiewicz, W. T.; Rypniewski, W. Engineering N-(2-Pyridyl)Aminoethyl Alcohols as Potential Precursors of Thermolabile Protecting Groups. *New J. Chem.* **2012**, *36* (3), 603–612. https://doi.org/10.1039/c1nj20584f.

(397) Zhou, B.; Li, L.; Zhu, X. Q.; Yan, J. Z.; Guo, Y. L.; Ye, L. W. Yttrium-Catalyzed Intramolecular Hydroalkoxylation/Claisen Rearrangement Sequence: Efficient Synthesis of Medium-Sized Lactams. *Angew. Chem. Int. Ed.* **2017**, *56* (14), 4015–4019. https://doi.org/10.1002/anie.201700596.

(398) Schmidt, K.; Margaretha, P. Photocyclodimers of "Made-to-Measure" Seven- and Six-Membered Cyclic Enones. *Helv. Chim. Acta* **2012**, *95* (3), 423–427. https://doi.org/10.1002/hlca.201100406.

(399) Duttwyler, S.; Lu, C.; Rheingold, A. L.; Bergman, R. G.; Ellman, J. A. Highly Diastereoselective Synthesis of Tetrahydropyridines by a C-H Activation-Cyclization-Reduction Cascade. *J. Am. Chem. Soc.* **2012**, *134* (9), 4064–4067. https://doi.org/10.1021/ja2119833.

(400) Zhou, T.; Li, S.; Huang, B.; Li, C.; Zhao, Y.; Chen, J.; Chen, A.; Xiao, Y.; Liu, L.; Zhang, J. Phosphine-Catalyzed Friedel-Crafts Reaction of Naphthols with: Para -Quinone Methides: Expedient Access to Triarylmethanes. *Org. Biomol. Chem.* **2017**, *15* (23), 4941–4945. https://doi.org/10.1039/c7ob00911a.

(401) Irving, A. Cl···π(Ar), Cl···Cl, and Other Intermolecular Interactions in the Crystal Structure of 8-Nitro-6-Methyl-2-Trichloromethyl-4-Dichloromethylene-1,3-Benzodioxin. *J. Crystallogr. Spectrosc. Res.* **1993**, *23* (9), 733–738. https://doi.org/10.1007/BF01187275.

(402) Calvert, J. L.; Hartshorn, M. P.; Robinson, W. T.; Wright, G. J. Nitration of 4-Chloro-2, 3, 6-Trimethylphenol, 3-Chloro-2, 4, 6-Trimethylphenol and 2, 3, 4, 6-Tetramethylphenol; 15N-Labeling Studies in the Reaction of 2, 3, 4, 6-Tetramethyl-4-Nitrocyclohexa-2, 5-Dienone With Nitrogen Dioxide. *Aust. J. Chem.* **1993**, *46* (11), 1447–1471. https://doi.org/10.1071/CH9931629.

(403) Lankri, D.; Mostinski, Y.; Tsvelikhovsky, D. Palladium-Catalyzed Cascade Assembly of Tricyclic Spiroethers from Diene-Alcohol Precursors. *J. Org. Chem.* **2017**, *82* (18), 9452–9463. https://doi.org/10.1021/acs.joc.7b01481.

(404) Mlostoń, G.; Petit, M.; Linden, A.; Heimgartner, H. Umsetzung von Di(Tert-butyl)- Und Diphenyldiazomethan Mit 1,3-Thiazol-5(4H)-thionen: Isolierung Und Kristallstruktur Des Primären Cycloadduktes. *Helv. Chim. Acta* **1994**, *77* (2), 435–444. https://doi.org/10.1002/hlca.19940770204.

(405) Yip, K. T.; Nimje, R. Y.; Leskinen, M. V.; Pihko, P. M. Palladium-Catalyzed Dehydrogenative B′-Arylation of β-Keto Esters under Aerobic Conditions: Interplay of Metal and Brønsted Acids. *Chem. Eur. J.* **2012**, *18* (40), 12590–12594. https://doi.org/10.1002/chem.201201988.

(406) Meng, J. -B; Du, D. -M; Xiong, G. -X; Wang, W. -G; Wang, Y. -M; Koshima, H.; Matsuura, T. A Dual Pathway in the Solid-state Photoreaction of Nitrobenzaldehydes with Indole. *J. Heterocycl. Chem.* **1994**, *31* (1), 121–124. https://doi.org/10.1002/jhet.5570310121.

(407) Duetsch, M.; Cidioni, S.; Stein, F.; Funke, F.; Noltemeyer, M.; De Meijere, A. 5-Methylene-2-Cyclopentenones as New Formal [2 + 2 + 1] Cycloaddcts from[2-(Dibenzylamino)Ethenyl]Carbenechromium Complexes and Alkynes. *J. Chem. Soc. Chem. Commun.* **1994**, No. 14, 1679–1680. https://doi.org/10.1039/C39940001679.

(408) Giacometti, A.; De Lucchi, O.; Dilillo, F.; Cossu, S.; Peters, K.; Peters, E. M.; von Schnering, H. G. Synthetic Equivalents to Substituted Acetylenes in Cycloaddition Reactions. Dienophilic Reactivity of 2-Methyl-, 2-Phenyl- and 2,3-Trimethylene-1,4-Benzodithiins-1,4-Tetroxides. *Tetrahedron* **1994**, *50* (26), 7913–7922. https://doi.org/10.1016/S0040-4020(01)85275-3.

(409) Chen, J.; Guo, H. M.; Zhao, Q. Q.; Chen, J. R.; Xiao, W. J. Visible Light-Driven Photocatalytic Generation of Sulfonamidyl Radicals for Alkene Hydroamination of Unsaturated Sulfonamides. *Chem. Commun.* **2018**, *54* (50), 6780–6783. https://doi.org/10.1039/c7cc09871e.

(410) Lee, Y. T.; Chiu, F. Y.; Barve, I. J.; Sun, C. M. Microwave-Assisted Synthesis of Benzimidazole-Linked Indoline and Indole Hybrids from C-2 Linked (o-Aminobenzyl)Benzimidazoles. *Adv. Synth. Catal.* **2018**, *360* (3), 502–512. https://doi.org/10.1002/adsc.201701140.

(411) Singh, V.; Yadav, G. P.; Maulik, P. R.; Batra, S. Studies toward the Construction of Substituted Piperidine-2-Ones and Pyridine-2-Ones from Baylis-Hillman Adducts: Discovery of a Facile Synthesis of 5-Methyl-4-Oxo-6-Aryl-3-Aza-Bicyclo[3.1.0]Hexane-1-Carboxylates. *Tetrahedron* **2006**, *62* (37), 8731–8739. https://doi.org/10.1016/j.tet.2006.06.099.

(412) Calvert, J.; Eberson, L.; Hartshorn, M.; Maclaga, n; Robinson, W. Photochemical Nitration by Tetranitromethane. XVII. The Regiochemistry of Adduct Formation in the Photochemical Reaction of 1-Methylnaphthalene and Tetranitromethane. *Aust. J. Chem.* **1994**, *47* (8), 1591. https://doi.org/10.1071/ch9941591.

(413) Briquet, A. A. S.; Hansen, H. -J. Thermal and Ru-catalyzed Reactions of Styryl-Substituted Azulenes with Dimethyl Acetylenedicarboxylate. *Helv. Chim. Acta* **1994**, *77* (7), 1940–1968. https://doi.org/10.1002/hlca.19940770723.

(414) Prinzbach, H.; Bingmann, H.; Beck, A.; Hunkler, D.; Sauter, H.; Hädicke, E. Cyclisch Gekreuzt-konjugierte Bindungssysteme, 381) Vierzehn-Elektronen-Elektrocyclisierung Des Vinylogen Sesquifulvalens – Phenazulen. *Chem. Ber.* **1981**, *114* (5), 1697–1722. https://doi.org/10.1002/cber.19811140511.

(415) Brown, R. S.; Christi, M.; Lough, A. J.; Ma, J.; Peters, E. M.; Peters, K.; Sammtleben, F.; Slebocka-Tilk, H.; Sung, K.; Tidwell, T. T. Addition of Bromine to Ketenes and Bisketenes: Electrophilic Attack at Carbonyl Carbon and Neighboring Group Participation. *J. Org. Chem.* **1998**, *63* (17), 6000–6006. https://doi.org/10.1021/jo980684h.

(416) Wu, K.; Du, Y.; Wei, Z.; Wang, T. Synthesis of Functionalized Pyrroloindolines: Via a Visible-Light-Induced Radical Cascade Reaction: Rapid Synthesis of (±)-Flustraminol B. *Chem. Commun.* **2018**, *54* (54), 7443–7446.

https://doi.org/10.1039/c8cc03575j.

(417) Aelvoet, K.; Batsanov, A. S.; Blatch, A. J.; Grosjean, C.; Patrick, L. G. F.; Smethurst, C. A.; Whiting, A. A Catalytic Aldol Reaction and Condensation through in Situ Boron "Ate" Complex Enolate Generation in Water. *Angew. Chem. Int. Ed.* **2008**, *47* (4), 768–770. https://doi.org/10.1002/anie.200704293.

(418) Mukherjee, M.; Mukherjee, A. K.; Das, S.; Mukherjee, D.; Helliwell, M. (±)-[6α(H)]-7,7,9β-Trimethyl-Cis-Tricyclo[6.3.1.01,6]Dodecan-2-One. *Acta Cryst. C* **1995**, *51* (2), 280–282. https://doi.org/10.1107/s0108270194008632.

(419) Kotha, S.; Cheekatla, S. R. Synthesis and Acid Catalyzed Rearrangement of Cage Propellanes. *ChemistrySelect* **2019**, *4* (46), 13440–13445. https://doi.org/10.1002/slct.201903441.

(420) Moricz, A.; Gassmann, E.; Bienz, S.; Hesse, M. Synthesis of (±)-Pyrenolide B. *Helv. Chim. Acta* **1995**, *78* (3), 663–669. https://doi.org/10.1002/hlca.19950780313.

(421) Kavala, V.; Murru, S.; Patel, B. K.; Das, G. Self-Assembled Superstructure of Xanthene Derivatives. *J. Chem. Crystallogr.* **2007**, *37* (8), 527–535. https://doi.org/10.1007/s10870-007-9201-1.

(422) Singh, R. R.; Skaria, M.; Chen, L. Y.; Cheng, M. J.; Liu, R. S. Gold-Catalyzed (4+3)-Annulations of 2-Alkenyl-1-Alkynylbenzenes with Anthranils with Alkyne-Dependent Chemoselectivity: Skeletal Rearrangement: Versus Non-Rearrangement. *Chem. Sci.* **2019**, *10* (4), 1201–1206. https://doi.org/10.1039/c8sc03619e.

(423) Yoshida, S.; Sugihara, Y.; Nakayama, J. Preparation and Properties of Nitrogen-Substituted Thiosulfinyl Compounds and Related New Heterocycles. *Tetrahedron Lett.* **2007**, *48* (46), 8116–8119. https://doi.org/10.1016/j.tetlet.2007.09.127.

(424) Ramesh, P.; Murugavel, S.; Pandi, A. S.; Murugan, R.; Narayanan, S. S. Ethyl 3-Phenyl-4-[(Z)-3-Phenyl-Acrylo-Yl] 5-(3,4,5-Trimethoxy-Phen-Yl) Pyrrolidine-2-Carboxyl-Ate. *Acta Cryst. E* **2007**, *63* (12), o4873–o4873. https://doi.org/10.1107/S1600536807059776.

(425) Bishop, R.; Downing, G. A.; Craig, D. C.; Scudder, M. L. Enantiomeric Self-Resolution through Dimethylsulfoxide Complexation. *J. Incl. Phenom. Mol. Recognit. Chem.* **1998**, *31* (2), 145–160. https://doi.org/10.1023/A:1007958031910.

(426) Kreft, A.; Lücht, A.; Grunenberg, J.; Jones, P. G.; Werz, D. B. Kinetic Studies of Donor–Acceptor Cyclopropanes: The Influence of Structural and Electronic Properties on the Reactivity. *Angew. Chem. Int. Ed.* **2019**, *58* (7), 1955–1959. https://doi.org/10.1002/anie.201812880.

(427) Talk, R. A.; El-Tunsi, A.; Robertson, C. C.; Coldham, I. Regioselective Lithiation and Electrophilic Quenching of N-Boc-3-Phenyltetrahydroisoquinoline. *Eur. J. Org. Chem.* **2019**, *2019* (31–32), 5294–5301. https://doi.org/10.1002/ejoc.201900238.

(428) Hart, H.; Chen, S. M.; Lee, S.; Ward, D. L.; Rung, W. J. H. Photorearrangements of Epoxy Ketones. Epoxides of Hexamethylbicyclo[3.2.0]Hepta-2, 5-Dienone, a Valence Tautomer of Hexamethyltropone. *J. Org. Chem.* **1980**, *45* (11), 2091–2096. https://doi.org/10.1021/jo01299a012.

(429) Gál, M.; Fehér, Ö.; Tihanyi, E.; Horváth, G.; Jerkovich, G.; Argay, G.; Kálmán, A. The Ring Closure and Rearrangement of 1-(2-Amino)-Benzoyl-1-Methylhydrazones of β-Dicarbonyl Compounds: On the Formation and Crystal Structure of 3a,9a-Dihydro- -1,3,3a,9a-Tetramethyl-4H-Pyrazolo[3,4-b]Quinolin-4-One. *Tetrahedron Lett.* **1980**, *21* (16), 1567–1570. https://doi.org/10.1016/S0040-4039(00)92777-1.

(430) Liu, Y. M.; Liu, H.; Hu, Y. Q. Synthesis and Crystal Structure of 3-Quinuclidinyl α-(Cyclopentyl-1- Ene)-α-Hydroxy-α-Phenylacetate. *J. Chem. Crystallogr.* **2008**, *38* (6), 491–494. https://doi.org/10.1007/s10870-008-9395-x.

(431) Freytag, M.; Grunenberg, J.; Jones, P. G.; Schmutzler, R. Two Sterically Encumbered 1,3,2-Dioxaphospholanes - Reactions, Comparison of Crystal Structures and Computational Explanations. *Zeitschrift f?r Anorg. und Allg. Chemie* **2008**, *634* (8), 1256–1266. https://doi.org/10.1002/zaac.200700514.

(432) Khlebnikov, A. F.; Konev, A. S.; Virtsev, A. A.; Yufit, D. S.; Mlostoń, G.; Heimgartner, H. Concerted vs. Non-Concerted 1,3-Dipolar Cycloadditions of Azomethine Ylides to Electron-Deficient Dialkyl 2,3-Dicyanobut-2-Enedioates. *Helv. Chim. Acta* **2014**, *97* (4), 453–470. https://doi.org/10.1002/hlca.201300405.

(433) Aouf, C.; Abed, D. El; Giorgi, M.; Santelli, M. Titanium-Mediated Addition of Diallylsilanes to Oxalyl Chloride: Formation of a Diquinane. *Tetrahedron Lett.* **2008**, *49* (24), 3862–3864. https://doi.org/10.1016/j.tetlet.2008.04.078.

(434) Chauveau, C.; Fouquay, S.; Michaud, G.; Simon, F.; Carpentier, J. F.; Guillaume, S. M. α,ω-Di(Vinylene Carbonate) Telechelic Polyolefins: Synthesis by Metathesis Reactions and Studies as Potential Precursors toward Hydroxy-Oxazolidone-Based Polyolefin NIPUs. *Eur. Polym. J.* **2019**, *116*, 144–157. https://doi.org/10.1016/j.eurpolymj.2019.03.052.

(435) Acosta, L. M.; Bahsas, A.; Palma, A.; Cobo, J.; Low, J. N.; Glidewell, C. Three Styryl-Substituted Tetrahydro-1,4-Epoxy-1-Benzazepines: Configurations, Conformations and Hydrogen-Bonded Chains. *Acta Cryst. C* **2008**, *64* (9), o514–o518. https://doi.org/10.1107/S0108270108025638.

(436) Gómez, S. L.; Raysth, W.; Palma, A.; Cobo, J.; Low, J. N.; Glidewell, C. Three Aryl-Substituted Tetrahydro-1,4-Epoxy-1-Benzazepines: Hydrogen-Bonded Structures in Two or Three Dimensions. *Acta Cryst. C* **2008**, *64* (9), o519–o523. https://doi.org/10.1107/S0108270108025961.

(437) Fusi, S.; Ponticelli, F.; Ventura, A.; Adamo, M. F. A. Rearrangement of 3,5-Dicyano-1,4-Dihydropyridines to Densely Functionalized Cyclopentadienes. *Tetrahedron Lett.* **2008**, *49* (40), 5820–5822. https://doi.org/10.1016/j.tetlet.2008.07.129.

(438) Huang, X.; Miao, M. Substrate-Controlled Selective Proximal and Distal C-C Bond Cleavage via Lewis Acid Mediated O-Acylation of 2-(Arylmethylene)Cyclopropylaldehyde: A Stereoselective Synthesis of Bifunctional Methylenecyclobutanes and 1,3-Conjugated Dienes. *J. Org. Chem.* **2008**, *73* (17), 6884–6887. https://doi.org/10.1021/jo801021f.

(439) Ungureanu, I.; Bologa, C.; Chayer, S.; Mann, A. Phenylaziridine as a 1,3-Dipole. Application to the Synthesis of Functionalized Pyrrolidines. *Tetrahedron Lett.* **1999**, *40* (29), 5315–5318. https://doi.org/10.1016/S0040-4039(99)01002-3.

(440) Miyahara, Y.; Ito, Y. N. AlCl3-Mediated Aldol Cyclocondensation of 1,6- and 1,7-Diones to Cyclopentene and Cyclohexene Derivatives. *J. Org. Chem.* **2014**, *79* (15), 6801–6807. https://doi.org/10.1021/jo5006137.

(441) Patil, D. V.; Park, H. S.; Koo, J.; Han, J. W.; Shin, S. Aerobic Oxygenative Cleavage of Electron Deficient C-C Triple Bonds in the Gold-Catalyzed Cyclization of 1,6-Enynes. *Chem. Commun.* **2014**, *50* (84), 12722–12725. https://doi.org/10.1039/c4cc04153d.

(442) Chen, Y.; Wang, D.; Duan, P.; Ben, R.; Dai, L.; Shao, X.; Hong, M.; Zhao, J.; Huang, Y. A Multitasking Functional Group Leads to Structural Diversity Using Designer C-H Activation Reaction Cascades. *Nat. Commun.* **2014**, *5* (1), 1–9. https://doi.org/10.1038/ncomms5610.

(443) Cabrera, A.; Le Lagadec, R.; Sharma, P.; Arias, J. L.; Toscano, R. A.; Velasco, L.; Gaviño, R.; Alvarez, C.; Salmón, M. Cyclo- and Hydrodimerization of α,β-Unsaturated Ketones Promoted by Samarium Diiodide. *J. Chem. Soc. Perkin Trans. 1* **1998**, No. 21, 3609–3617. https://doi.org/10.1039/a804269a.

(444) González-Rosende, M. E.; Sepúlveda-Arques, J.; Zaballos-Garcia, E.; Domingo, L. R.; Zaragozá, R. J.; Jennings, W. B.; Lawrence, S. E.; O'Leary, D. Domino Reaction between 2-Acylfurans and Diethyl Azodicarboxylate: A Combined Experimental, Theoretical, X-Ray and Dynamic NMR Study. *J. Chem. Soc. Perkin Trans. 2* **1999**, No. 1, 73–79. https://doi.org/10.1039/a806628k.

(445) Yan, X. Q.; Wang, Z. C.; Qi, P. fei; Li, G.; Zhu, H. L. Design, Synthesis and Biological Evaluation of 2-H Pyrazole Derivatives Containing Morpholine Moieties as Highly Potent Small Molecule Inhibitors of APC–Asef Interaction. *European Journal of Medicinal Chemistry*. Elsevier Masson SAS September 1, 2019, pp 425–447. https://doi.org/10.1016/j.ejmech.2019.05.056.

(446) Amutha, C.; Saravanan, S.; Muthusubramanian, S. Acetic Anhydride Induced Rearrangement and Grignard Addition on C-Phenyl-N-(1-Methyl-2-Aryl)Ethyl Nitrones. *Indian J. Chem. - Sect. B Org. Med. Chem.* **2013**, *52* (5), 646–653. https://doi.org/10.1002/chin.201335075.

(447) Bielenica, A.; Struga, M.; Mirosław, B.; Kozioł, A. E.; Kossakowski, J.; Sanna, G.; Colla, P. La; Giliberti, G. Synthesis and Biological Evaluation of N-Substituted Polycyclic Imides Derivatives. *Acta Pol. Pharm. - Drug Res.* **2013**, *70* (5), 809–822.

(448) Zhang, C.; Murarka, S.; Seidel, D. Facile Formation of Cyclic Aminals through a Brønsted Acid-Promoted Redox Process. *J. Org. Chem.* **2009**, *74* (1), 419–422. https://doi.org/10.1021/jo802325x.

(449) Robertson, J.; Naud, S. Synthesis of Spiroacetal Enol Ethers by Oxidative Activation of Furan Derivatives. *Org. Lett.* **2008**, *10* (23), 5445–5448. https://doi.org/10.1021/ol802138t.

(450) McQuaid, K. M.; Sames, D. C-H Bond Functionalization via Hydride Transfer: Lewis Acid Catalyzed Alkylation Reactions by Direct Interamolecular Coupling of Sp 3 C-H Bonds and Reactive Alkenyl Oxocarbenium Intermediates. *J. Am. Chem. Soc.* **2009**, *131* (2), 402–403. https://doi.org/10.1021/ja806068h.

(451) Fukuyama, K.; Tanaka, N.; Kakudo, M. Structure of 1,2,3,9b-Tetrahydro-9bβ-Hydroxy-2β-Methoxy-1α-Phenyl-5 H -Pyrrolo[2,1-α]Isoindol-5-One . *Acta Cryst. B* **1980**, *36* (8), 1965–1966. https://doi.org/10.1107/s0567740880007662.

(452) Ali Dondas, H.; Grigg, R.; Hadjisoteriou, M.; Markandu, J.; Kennewell, P.; Thornton-Pett, M. X = Y-ZH Systems as Potential 1,3-Dipoles. Part 51: Halogen-Induced Inter- and Intra-Molecular Formation of Nitrones from Oximes

and Alkenes. *Tetrahedron* **2001**, *57* (6), 1119–1128. https://doi.org/10.1016/S0040-4020(00)01084-X.

(453) Yang, C.; Zhang, X.; Zhang-Negrerie, D.; Du, Y.; Zhao, K. PhI(OCOCF3)2-Mediated Cyclization of o -(1-Alkynyl)Benzamides: Metal-Free Synthesis of 3-Hydroxy-2,3-Dihydroisoquinoline-1,4-Dione. *J. Org. Chem.* **2015**, *80* (10), 5320–5328. https://doi.org/10.1021/acs.joc.5b00576.

(454) Krishna, R.; Velmurugan, D.; Shanmugasundaram, M.; Raghunathan, R.; Sekar, K.; Shanmuga Sundara Raj, S.; Fun, H. K. Crystal Structure Analysis of 1′,2′,3′,4′-tetrahydro-1,3-diphenyl-4-chlorospiro[2-pyrazoline-5,2-napthalen]1′one - Krishna - 2002 - Crystal Research and Technology - Wiley Online Library. *Cryst. Res. Technol.* **2002**, *37* (1), 135–141.

(455) Mostinski, Y.; Valerio, V.; Lankri, D.; Tsvelikhovsky, D. Synthesis of Tricyclic Spiranoid Lactones via I2/Sm(II)- and I2/Pd(0)-Mediated Cyclizations of a Common Cycloalkylmethylene Precursor. *J. Org. Chem.* **2015**, *80* (21), 10464–10473. https://doi.org/10.1021/acs.joc.5b01278.

(456) Chang, M. Y.; Lin, C. H.; Chen, Y. L. Selenium Dioxide-Mediated Methoxyhydroxylation of Cyclic Arylolefin. *Tetrahedron Lett.* **2010**, *51* (10), 1430–1433. https://doi.org/10.1016/j.tetlet.2010.01.020.

(457) Mohammadizadeh, M. R.; Firoozi, N. A Novel, Convenient, and Efficient Procedure for the Synthesis of Spiroisoindoline-1,5′-Oxazolidine Derivatives. *Tetrahedron Lett.* **2010**, *51* (18), 2467–2469. https://doi.org/10.1016/j.tetlet.2010.02.163.

(458) Krow, G. R.; Lester, W. S.; Lin, G.; Fang, Y.; Carroll, P. J. Chlorosulfonyl Isocyanate Reactions with N-(Alkoxycarbonyl)-2-Azabicyclo[2.2.0]Hex5-Enes. Regiospecific Two-Atom Insertion Pathways. *J. Org. Chem.* **2003**, *68* (4), 1626–1629. https://doi.org/10.1021/jo020655d.

(459) Cui, H. F.; Dong, K. Y.; Nie, J.; Zheng, Y.; Ma, J. A. Lewis Acid-Catalyzed One-Pot Sequential Reaction for the Synthesis of α-Halogenated β-Keto Esters. *Tetrahedron Lett.* **2010**, *51* (17), 2374–2377. https://doi.org/10.1016/j.tetlet.2010.02.158.

(460) Demory, E.; Devaraj, K.; Orthaber, A.; Gates, P. J.; Pilarski, L. T. Boryl (Hetero)Aryne Precursors as Versatile Arylation Reagents: Synthesis through C-H Activation and Orthogonal Reactivity. *Angew. Chem. Int. Ed.* **2015**, *54* (40), 11765–11769. https://doi.org/10.1002/anie.201503152.

(461) Lu, D.; Wan, Y.; Kong, L.; Zhu, G. Copper-Catalyzed Cascade Annulation between α-Bromocarbonyls and Biaryl or (: Z)-Arylvinylacetylenes Enabling a Direct Synthesis of Dibenzocycloheptanes and Related Compounds. *Chem. Commun.* **2016**, *52* (97), 13971–13974. https://doi.org/10.1039/c6cc07727g.

(462) Chan, C. K.; Tsai, Y. L.; Chang, M. Y. Construction of Nitrated Benzo[3.3.1]Bicyclic Acetal/Ketal Core via Nitration of o-Carbonyl Allylbenzenes. *Org. Lett.* **2017**, *19* (6), 1358–1361. https://doi.org/10.1021/acs.orglett.7b00245.

(463) Lysenko, I. L.; Oh, H. S.; Jin, K. C. Diastereoselective Prins-Type Reaction of Cycloalkenylcyclopropanol Silyl Ethers and α,β-Unsaturated Aldehyde Acetals. *J. Org. Chem.* **2007**, *72* (21), 7903–7908. https://doi.org/10.1021/jo071272o.

(464) Kang, Y. Nickel-Catalyzed Double Silylation of a Variety of Carbonyl Compounds with 1,2-Bis(Dimethylsilyl)Carborane. *Organometallics* **2000**, *19* (24), 5026–5031. https://doi.org/10.1021/om000527k.

(465) Gais, H. J.; Van Gumpel, M.; Schleusner, M.; Raabe, G.; Runsink, J.; Vermeeren, C. Lithium Salts of Conformationally Constrained and Restricted Chiral Allylic α-Sulfonyl Carbanions - A Joint Study of Their Structures, Dynamics, and Stereoselectivities. *Eur. J. Org. Chem.* **2001**, No. 22, 4275–4303. https://doi.org/10.1002/1099-0690(200111)2001:22<4275::AID-EJOC4275>3.0.CO;2-1.

(466) Banerjee, R.; Mondal, R.; Howard, J. A. K.; Desiraju, G. R. Synthon Robustness and Solid-State Architecture in Substituted Gem-Alkynols. *Cryst. Growth Des.* **2006**, *6* (4), 999–1009. https://doi.org/10.1021/cg050598s.

(467) Yamaguchi, K.; Utsumi, K.; Yoshitake, Y.; Harano, K. Cyclization Reaction of Cyclopentadienone with Prop-2-Yn-1-Ol in Priority to Diels-Alder Reaction. *Tetrahedron Lett.* **2006**, *47* (25), 4235–4239. https://doi.org/10.1016/j.tetlet.2006.04.021.

(468) Witthaut, D.; Fröhlich, R.; Schäfer, H. J. Heterocycles through Domino Reactions with Trimethyl Aconitate, a Versatile Synthetic Building Block. *Angew. Chem. Int. Ed.* **2001**, *40* (22), 4212–4214. https://doi.org/10.1002/1521-3773(20011119)40:22<4207::AID-ANIE4207>3.0.CO;2-W.

(469) Xu, G.; Tong, C.; Cui, S.; Dai, L. A Silver Catalyzed Domino Reaction of: N -Cyanamide Alkenes and 1,3-Dicarbonyls for the Synthesis of Quinazolinones. *Org. Biomol. Chem.* **2018**, *16* (32), 5899–5906. https://doi.org/10.1039/c8ob01252k.

(470) Chowdhury, R.; Ghosh, S. K. An Organocatalyzed Asymmetric Michael Addition of Cyclic Ketones to 1,3-Diene-1,1-Dicarboxylates. *Eur. J. Org. Chem.* **2013**, *2013* (27), 6167–6174. https://doi.org/10.1002/ejoc.201300735.

(471) Singh, G.; Linden, A.; Abou-Hadeed, K.; Hansen, H. J. New Products from the Heptalene-Forming Reaction of

Azulenes and Acetylenedicarboxylates in Polar Media. *Helv. Chim. Acta* **2002**, *85* (1), 27–59. https://doi.org/10.1002/1522-2675(200201)85:1<27::AID-HLCA27>3.0.CO;2-F.

(472) Malpass, J. R.; Belkacemi, D.; Russell, D. R. Studies of Stereoselectivity in Cycloaddition of Cyclic Dienes to 2-Azabicyclo[2.2.2]Octene Derivatives; through-Space Effects on 15N NMR Shifts of Bicyclic Amines and Lactams. *Tetrahedron* **2002**, *58* (1), 197–204. https://doi.org/10.1016/S0040-4020(01)01125-5.

(473) García, E. G.; Mendoza, V. G.; Guzmán, J. A. B.; Maldonado Graniel, L. A.; Hernández-Ortega, S. 2-[3-Furyl(Hydroxy)Methyl]-2,3-Dimethylcyclohexanone. *Acta Cryst. C* **2002**, *58* (6), o336–o338. https://doi.org/10.1107/S0108270102007527.

(474) Sakharov, P. A.; Novikov, M. S.; Khlebnikov, A. F. 2-Diazoacetyl-2 H-Azirines: Source of a Variety of 2 H-Azirine Building Blocks with Orthogonal and Domino Reactivity. *J. Org. Chem.* **2018**, *83* (15), 8304–8314. https://doi.org/10.1021/acs.joc.8b01004.

(475) Das, P.; Omollo, A. O.; Sitole, L. J.; McClendon, E.; Valente, E. J.; Raucher, D.; Walker, L. R.; Hamme, A. T. Synthesis and Investigation of Novel Spiro-Isoxazolines as Anti-Cancer Agents. *Tetrahedron Lett.* **2015**, *56* (14), 1794–1797. https://doi.org/10.1016/j.tetlet.2015.02.059.

(476) Thenmozhi, S.; Govindan, E.; Gavaskar, D.; Raghunathan, R.; Subbiahpandi, A. 3′-(4-Chlorobenzoyl)-1′-Methyl-4′-[5-(2-Thienyl) -2-Thienyl]Spiro-[Acenaphthylene-1,2′-Pyrrolidin]-2(1H)-One. *Acta Cryst. E* **2011**, *67* (2), 148–155. https://doi.org/10.1107/S1600536810053870.

(477) Chen, T.; Li, Y. F.; An, Y.; Zhang, F. M. Iron-Catalyzed α-Arylation of Deoxybenzoins with Arenes through an Oxidative Dehydrogenative Approach. *Org. Lett.* **2016**, *18* (18), 4754–4757. https://doi.org/10.1021/acs.orglett.6b02516.

(478) Wei, Z. L.; Xiao, Y.; George, C.; Kellar, K. J.; Kozikowski, A. P. Functionalization of the Alicyclic Skeleton of Epibatidine: Synthesis and Nicotinic Acetylcholine Receptor Binding Affinities of Epibatidine Analogues. *Org. Biomol. Chem.* **2003**, *1* (22), 3878–3881. https://doi.org/10.1039/b308906a.

(479) Gupta, S.; Saluja, P.; Khurana, J. M. DBU Mediated Confluent Approach for the One Pot Synthesis of Novel 5-Hydroxy Pyrazolo[1,2-a][1,2,4]Triazoles and Their Dehydration to Novel Pyrazolo[1,2-a][1,2,4]Triazole Derivatives. *Tetrahedron* **2016**, *72* (27–28), 3986–3993. https://doi.org/10.1016/j.tet.2016.05.021.

(480) Lian, Z.; Guan, X. Y.; Shi, M. Phosphine-Mediated Annulation of N-Protected Imines with DEAD. *Tetrahedron* **2011**, *67* (11), 2018–2024. https://doi.org/10.1016/j.tet.2011.01.072.

(481) Palusiak, M.; Grabowski, S. J.; Epsztajn, J.; Kowalska, J. A. 8-Methoxy-4-(4-Methoxyphenyl)Isochroman-3-Ol. *Acta Cryst. E* **2003**, *59* (12), 2000–2002. https://doi.org/10.1107/S1600536803026102.

(482) Qin, Z.; Liu, W.; Wang, D.; He, Z. Phosphine-Catalyzed (4 + 1) Annulation of o-Hydroxyphenyl and o-Aminophenyl Ketones with Allylic Carbonates: Syntheses and Transformations of 3-Hydroxy-2,3-Disubstituted Dihydrobenzofurans and Indolines. *J. Org. Chem.* **2016**, *81* (11), 4690–4700. https://doi.org/10.1021/acs.joc.6b00596.

(483) Wang, Z.; Castellano, S.; Kinderman, S. S.; Argueta, C. E.; Beshir, A. B.; Fenteany, G.; Kwon, O. Diversity through a Branched Reaction Pathway: Generation of Multicyclic Scaffolds and Identification of Antimigratory Agents. *Chem. Eur. J.* **2011**, *17* (2), 649–654. https://doi.org/10.1002/chem.201002195.

(484) Cruz, D.; Wang, Z.; Kibbie, J.; Modlin, R.; Kwon, O. Diversity through Phosphine Catalysis Identifies Octahydro-1,6- Naphthyridin-4-Ones as Activators of Endothelium-Driven Immunity. *Proc. Natl. Acad. Sci.* **2011**, *108* (17), 6769–6774. https://doi.org/10.1073/pnas.1015254108.

(485) Da Silva Maia, A. F.; Siqueira, R. P.; De Oliveira, F. M.; Ferreira, J. G.; Da Silva, S. F.; Caiuby, C. A. D.; De Oliveira, L. L.; De Paula, S. O.; Souza, R. A. C.; Guilardi, S.; Bressan, G. C.; Teixeira, R. R. Synthesis, Molecular Properties Prediction and Cytotoxic Screening of 3-(2-Aryl-2-Oxoethyl)Isobenzofuran-1(3H)-Ones. *Bioorganic Med. Chem. Lett.* **2016**, *26* (12), 2810–2816. https://doi.org/10.1016/j.bmcl.2016.04.065.

(486) Lee, C. H.; Jin, G. F.; Lim, H. W.; Yang, E. H.; Lee, J. D.; Nakamura, H.; Ban, H. S.; Kang, S. O. Facile Synthesis of 4-Substituted 3,4-Dihydro-1H-2,1,3-Benzothiadiazine 2,2-Dioxides. *Heteroat. Chem.* **2011**, *22* (2), 192–197. https://doi.org/10.1002/hc.20670.

(487) Nallini, A.; Saraboji, K.; Ponnuswamy, M. N.; Venkatraj, M.; Jeyaraman, R. Crystal Structure and Conformation of a Pair of Piperidine Derivatives. *Mol. Cryst. Liq. Cryst.* **2003**, *403*, 57–65. https://doi.org/10.1080/15421400390234057.

(488) Shirrell, C. D.; Williams, D. E. The Crystal Structure of 2-Isopropylidene-1,1,7,7,9,9-Hexamethyl-3,5,10,11-Tetrathiadispiro[3,1,3,2]Undecane-8-Thione. *Acta Cryst. B* **1973**, *29* (10), 2128–2133. https://doi.org/10.1107/s0567740873006229.

(489) Dietz, J.; Martin, S. F. Novel Entry to the Tricyclic Core of Stemofoline and Didehydrostemofoline. *Tetrahedron Lett.* **2011**, *52* (17), 2048–2050. https://doi.org/10.1016/j.tetlet.2010.10.038.

(490) Bhanuchandra, M.; Kuram, M. R.; Sahoo, A. K. Silver(I)-Catalyzed Reaction between Pyrazole and Propargyl Acetates: Stereoselective Synthesis of the Scorpionate Ligands (E)-Allyl-Gem-Dipyrazoles (ADPs). *J. Org. Chem.* **2013**, *78* (23), 11824–11834. https://doi.org/10.1021/jo401867e.

(491) Wang, Q. F.; Hou, H.; Hui, L.; Yan, C. G. Diastereoselective Synthesis of Trans-2,3-Dihydrofurans with Pyridinium Ylide Assisted Tandem Reaction. *J. Org. Chem.* **2009**, *74* (19), 7403–7406. https://doi.org/10.1021/jo901379h.

(492) Junker, C. S.; Welker, M. E.; Day, C. S. Synthesis of 4-Aryl- and 4-Alkyl-2-Silyl-1,3-Butadienes and Their Diels-Alder/Cross-Coupling Reactions. *J. Org. Chem.* **2010**, *75* (23), 8155–8165. https://doi.org/10.1021/jo1017734.

(493) Ibad, M. F.; Abid, O. U. R.; Adeel, M.; Nawaz, M.; Wolf, V.; Villinger, A.; Langer, P. Synthesis of Highly Functionalized Biaryls by Condensation of 2-Fluoro-1,3-Bis(Silyloxy) 1,3-Dienes with 3-Cyanochromones and Subsequent Domino "Retro-Michael/Aldol/Fragmentation." *J. Org. Chem.* **2010**, *75* (23), 8315–8318. https://doi.org/10.1021/jo1018443.

(494) Liu, J.; Xu, X.; Li, J.; Liu, B.; Jiang, H.; Yin, B. Palladium-Catalyzed Dearomatizing 2,5-Alkoxyarylation of Furan Rings: Diastereospecific Access to Spirooxindoles. *Chem. Commun.* **2016**, *52* (61), 9550–9553. https://doi.org/10.1039/c6cc04298h.

(495) Galeta, J.; Man, S.; Bouillon, J. P.; Potáäček, M. Unexpected Heterocyclic Products from Cycloaddition Reactions of Nonsymmetrical Allenyl Aldoketazines with Substituted Alkynes. *Eur. J. Org. Chem.* **2011**, *2011* (2), 392–398. https://doi.org/10.1002/ejoc.201001044.

(496) Jiang, X.; Yang, J.; Zhang, F.; Yu, P.; Yi, P.; Sun, Y.; Wang, Y. Synthesis of Quaternary 3,3-Disubstituted 2-Oxindoles from 2-Substituted Indole Using Selectfluor. *Org. Lett.* **2016**, *18* (13), 3154–3157. https://doi.org/10.1021/acs.orglett.6b01367.

(497) Arde, P.; Anand, R. V. Expedient Access to Unsymmetrical Triarylmethanes through N-Heterocyclic Carbene Catalysed 1,6-Conjugate Addition of 2-Naphthols to: Para -Quinone Methides. *RSC Adv.* **2016**, *6* (81), 77111–77115. https://doi.org/10.1039/c6ra11116e.

(498) Kuznetsov, D. M.; Mukhina, O. A.; Kutateladze, A. G. Photoassisted Synthesis of Complex Molecular Architectures: Dearomatization of Benzenoid Arenes with Aza-o-Xylylenes via an Unprecedented [2+4] Reaction Topology. *Angew. Chem. Int. Ed.* **2016**, *55* (24), 6988–6991. https://doi.org/10.1002/anie.201602288.

(499) George, F.; Norberg, B.; Robeyns, K.; Wouters, J.; Leyssens, T. Peculiar Case of Levetiracetam and Etiracetam α-Ketoglutaric Acid Cocrystals: Obtaining a Stable Conglomerate of Etiracetam. *Cryst. Growth Des.* **2016**, *16* (9), 5273–5282. https://doi.org/10.1021/acs.cgd.6b00819.

(500) Palusiak, M.; Pfitzner, A.; Zabel, M.; Grabowski, S. J.; Epsztajn, J.; Kowalska, J. A. Isochroman Derivatives and Their Tendency to Crystallize in Chiral Space Groups. *Acta Cryst. C* **2004**, *60* (4), o239–o241. https://doi.org/10.1107/S0108270104003087.

(501) Schulza, M.; Wimmerb, K.; Gorlsb, H.; Westerhausen, M. Heterogeneously Catalyzed Diastereoselective Synthesis of 2-Nitro-1,3-Di(Pyridin-2-Yl)Propane-1,3-Diols. *Zeitschrift fur Naturforsch. - Sect. B J. Chem. Sci.* **2011**, *66* (6), 611–623. https://doi.org/10.1515/znb-2011-0609.

(502) Fleming, F. F.; Zhang, Z.; Wang, Q.; Steward, O. W. Oxonitriles: Multicomponent Grignard Addition-Alkylations. *Angew. Chem. Int. Ed.* **2004**, *43* (9), 1126–1129. https://doi.org/10.1002/anie.200352920.

(503) Fleming, F. F.; Zhang, Z.; Wei, G.; Steward, O. W. Metalated Nitriles: Electrophile-Dependent Alkylations. *Org. Lett.* **2005**, *7* (3), 447–449. https://doi.org/10.1021/ol047598q.

(504) Fleming, F. F.; Zhang, Z.; Wei, G.; Steward, O. W. C-Metalated Nitriles: Electrophile-Dependent Alkylations and Acylations. *J. Org. Chem.* **2006**, *71* (4), 1430–1435. https://doi.org/10.1021/jo052102j.

(505) Gao, H.; Wu, X.; Zhang, J. Gold(I)-Catalyzed, Highly Diastereoselective, Tandem Heterocyclizations/ [3+2] Cycloadditions: Synthesis of Highly Substituted Cyclopenta[c]Furans. *Chem. Eur. J.* **2011**, *17* (10), 2838–2841. https://doi.org/10.1002/chem.201003363.

(506) Xia, C.; White, A. J. P.; Hii, K. K. M. Synthesis of Isoindolinones by Pd-Catalyzed Coupling between N-Methoxybenzamide and Styrene Derivatives. *J. Org. Chem.* **2016**, *81* (17), 7931–7938. https://doi.org/10.1021/acs.joc.6b01696.

(507) Wang, M. A.; Zhang, N.; Lu, H. Z.; Wang, D. Q. Conformation of α,α,A'-Trisubstituted Cyclododecanone. *Chinese J. Chem.* **2007**, *25* (8), 1196–1201. https://doi.org/10.1002/cjoc.200790223.

(508) Krawczyk, E.; Owsianik, K.; Skowrońska, A.; Wieczorek, M.; Majzner, W. An Expedient, Stereoselective Synthesis of Highly Functionalized Cyclic Compounds. *New J. Chem.* **2002**, *26* (12), 1753–1767.

https://doi.org/10.1039/b207700k.

(509) Nečas, D.; Kotora, M.; Císařová, I. Iron-Catalyzed Transformations of 2-Chloro-1,6-Heptadienes. *Eur. J. Org. Chem.* **2004**, *2004* (6), 1280–1285. https://doi.org/10.1002/ejoc.200300660.

(510) Tanimoto, H.; Shitaoka, T.; Yokoyama, K.; Morimoto, T.; Nishiyama, Y.; Kakiuchi, K. Formal [3+2] Cycloaddition of Nitrosoallenes with Carbonyl and Nitrile Compounds to Form Functional Cyclic Nitrones. *J. Org. Chem.* **2016**, *81* (19), 8722–8735. https://doi.org/10.1021/acs.joc.6b00758.

(511) Pušavec Kirar, E.; Drev, M.; Mirnik, J.; Grošelj, U.; Golobič, A.; Dahmann, G.; Požgan, F.; Štefane, B.; Svete, J. Synthesis of 3D-Rich Heterocycles: Hexahydropyrazolo[1,5-A]Pyridin-2(1H)-Ones and Octahydro-2H-2a,2a1-Diazacyclopenta[Cd]Inden-2-Ones. *J. Org. Chem.* **2016**, *81* (19), 8920–8933. https://doi.org/10.1021/acs.joc.6b01608.

(512) Sonar, V. N.; Parkin, S.; Crooks, P. A. (Z)-2-(1-Phenylsulfonyl-1H-Indol3-Ylmethylene)-1-Azabicyclo[2.2.2] - Octan-3-One and (Z)-(S)-2-(1-Phenylsulfonyl-1H-Indol-3-Ylmethylene)-1-Azabicyclo[2.2.2] Octan-3-Ol. *Acta Cryst. C* **2004**, *60* (9), o659–o661. https://doi.org/10.1107/S0108270104015847.

(513) Nishibayashi, Y.; Yoshikawa, M.; Inada, Y.; Hidai, M.; Uemura, S. Ruthenium-Catalyzed Cycloaddition between Propargylic Alcohols and Cyclic 1,3-Dicarbonyl Compounds via an Allenylidene Intermediate. *J. Org. Chem.* **2004**, *69* (10), 3408–3412. https://doi.org/10.1021/jo0357465.

(514) Rulev, A. Y.; Romanov, A. R.; Kondrashov, E. V.; Ushakov, I. A.; Vashchenko, A. V.; Muzalevskiy, V. M.; Nenajdenko, V. G. Domino Assembly of Trifluoromethylated N,O-Heterocycles by the Reaction of Fluorinated α-Bromoenones with Amino Alcohols. *J. Org. Chem.* **2016**, *81* (20), 10029–10034. https://doi.org/10.1021/acs.joc.6b01927.

(515) Liu, Y.; Zhao, Q.; Zhang, Y. Intramolecular Cyclization and Disilylation of 1,1-Dicyano-2,2- Diarylethenes Promoted by Samarium/TMSCl in DMF: A New Approach to the Syntheses of Polysubstituted Indenes. *Tetrahedron Lett.* **2004**, *45* (23), 4571–4575. https://doi.org/10.1016/j.tetlet.2004.03.199.

(516) Liu, Y.; Zhang, F.; Qi, Y.; Zhang, Y.; Zhang, S. Transformation of Gem-Dicyanoethenes by Samarium: Direct Formation of Indenes or Direct Decyanation with in Situ Disilylation. *Eur. J. Org. Chem.* **2008**, *2008* (32), 5470–5476. https://doi.org/10.1002/ejoc.200800258.

(517) Kubicki, M.; Codding, P. W. The Anticonvulsant Sulfamates. 2. (1,4-Benzodioxin-2(3H)-Yl)- and (1,2,3,4-Tetrahydro-2-Naphthalenyl) Methyl Sulfamic Acid Esters. *J. Mol. Struct.* **2001**, *561* (1–3), 65–70. https://doi.org/10.1016/S0022-2860(00)00922-4.

(518) Lautens, M.; Schmid, G. A.; Chau, A. Remote Electronic Effects in the Rhodium-Catalyzed Nucleophilic Ring Opening of Oxabenzonorbornadienes. *J. Org. Chem.* **2002**, *67* (23), 8043–8053. https://doi.org/10.1021/jo025822o.

(519) Chornous, V. A.; Mel'Nik, O. Y.; Mel'Nik, D. A.; Rusanov, E. B.; Vovk, M. V. Polyfunctional Imidazoles: XI. Reaction of 1-Aryl-4-Chloro-5-(2-Nitrovinyl)-1H-Imidazoles with Nonstabilized Azomethine Ylides. Synthesis of (1-Aryl-4-Chloro-1H-Imidazol-5-Yl)-Substituted Nitropyrrolidines and Nitropyrrolizines. *Russ. J. Org. Chem.* **2015**, *51* (10), 1423–1429. https://doi.org/10.1134/S1070428015100115.

(520) Büyükgüngör, O. Structure of Endo,Exo-9,11-Dibromotricyclo[6.3.1.02,7]Dodeca-2(7),3,5-Trien-10-One. *Acta Cryst. C* **1989**, *45* (1), 75–77. https://doi.org/10.1107/s010827018800962x.

(521) Georges, G.; Norberg, B.; Evrard, G.; Durant, F. Structure Analysis of Anticonvulsant Drugs: 5-(p-Chlorophenylmethyl)-3-Methyl-2-Pyrrolidinone, Cis (I) and Trans (II) Isomers. *Acta Cryst. C* **1989**, *45* (3), 454–457. https://doi.org/10.1107/s010827018801176x.

(522) Chadwick, D. J.; Gilchrist, T. L.; Stretch, W. Structure of Ethyl 6-methyl-6-phenyl-5,6-dihydro-4H-1,2-oxazine-3-carboxylate. *Acta Cryst. C* **1989**, *45* (6), 976–978. https://doi.org/10.1107/S0108270188014878.

(523) Bellur, E.; Görls, H.; Langer, P. Regioselective Synthesis of Functionalized Furans by Cyclization of 1,3-Bis-Silyl Enol Ethers with 1-Chloro-2,2-Dimethoxyethane. *Eur. J. Org. Chem.* **2005**, *2005* (10), 2074–2090. https://doi.org/10.1002/ejoc.200400805.

(524) Zhi-Guang, X.; Hai-Yang, L.; Guo-Bang, G.; Xuan, X.; Yun-Xiu, Z. Benzyl 2-Ethyl-Hexyl Sulfoxide. *Acta Cryst. E* **2009**, *65* (11), 2929–2929. https://doi.org/10.1107/S1600536809044328.

(525) Evans, S. V.; Trotter, J. Reaction Pathways and Asymmetric Synthesis in the Solid-state Photochemistry of A-adamantylacetophenones. *Acta Cryst. B* **1989**, *45* (5), 500–505. https://doi.org/10.1107/S0108768189005690.

(526) Martelli, J.; Grée, D.; Kessabi, J.; Grée, R.; Toupet, L. Etude de La Diastéréosélectivité Dans Les Réactions de Diels-Alder Intramoléculaires à Partir d'alcools Diéniques; Syntheses Courtes de Lactones Polyfonctionnelles. *Tetrahedron* **1989**, *45* (13), 4213–4226. https://doi.org/10.1016/S0040-4020(01)81316-8.

(527) Begley, M. J.; Cheshire, D. R.; Harrison, T.; Hutchinson, J. H.; Myers, P. L.; Pattenden, G. A New Synthetic Route to (±)-Forskolin. *Tetrahedron* **1989**, *45* (16), 5215–5246. https://doi.org/10.1016/S0040-4020(01)81098-X.

(528) Wang, X. S.; Zhang, M. M.; Zeng, Z. Sen; Shi, D. Q.; Tu, S. J.; Wei, X. Y.; Zong, Z. M. A Simple and Clean Procedure for the Synthesis of Polyhydroacridine and Quinoline Derivatives: Reaction of Schiff Base with 1,3-Dicarbonyl Compounds in Aqueous Medium. *Tetrahedron Lett.* **2005**, *46* (42), 7169–7173. https://doi.org/10.1016/j.tetlet.2005.08.091.

(529) Fantauzzi, S.; Gallo, E.; Caselli, A.; Ragaini, F.; Macchi, P.; Casati, N.; Cenini, S. Origin of the Deactivation in Styrene Aziridination by Aryl Azides, Catalyzed by Ruthenium Porphyrin Complexes. Structural Characterization of a Δ2-1,2,3-Triazoline RuII(TPP)CO Complex. *Organometallics* **2005**, *24* (20), 4710–4713. https://doi.org/10.1021/om050244y.

(530) Horiguchi, Y.; Furukawa, T.; Kuwajima, I. A Highly Efficient Eight-Membered-Ring Cyclization for Construction of the Taxane Carbon Framework. *J. Am. Chem. Soc.* **1989**, *111* (21), 8277–8279. https://doi.org/10.1021/ja00203a039.

(531) Iddon, B.; Redhouse, A. D.; Yat, P. N. Synthesis and Reactions of 2,3,5,6-Tetrahydro-2,5-Ethano-3-Benzazocin-4(1H) -One and a Thieno-Extended Analogue: X-Ray Structure of 3-Methyl-2,3,5,6- Tetrahydro-2,5-Ethano[1]Benzothieno[3,2-d]Azocin-4(1H)-One. *J. Chem. Soc. Perkin Trans. 1* **1990**, No. 4, 1083–1090. https://doi.org/10.1039/p19900001083.

(532) Yamashita, M.; Dnyanoba, Y. N.; Nagahama, M.; Inaba, T.; Nishino, Y.; Miura, K.; Kosaka, S.; Fukao, J.; Kawasaki, I.; Ohta, S. Synthesis and Unambiguous Stereochemical Determination of 1-Exo- and 1-Endo-1-Aryl-1,2,2a,8b-Tetrahydro-3h-Benzo[b]Cyclobuta[d]Pyran-3-Ones. *Heterocycles* **2005**, *65* (10), 2411–2430. https://doi.org/10.3987/COM-05-10498.

(533) Bredikhin, A. A.; Bredikhina, Z. A.; Zakharychev, D. V.; Akhatova, F. S.; Krivolapov, D. B.; Litvinov, I. A. Solid-State Properties of 1,2-Epoxy-3-(2-Cyanophenoxy)Propane, a Conglomerate-Forming Chiral Drug Precursor. *Mendeleev Commun.* **2006**, *16* (5), 245–247. https://doi.org/10.1070/MC2006v016n05ABEH002388.

(534) Reich, H. J.; Holtan, R. C.; Bolm, C. Acylsilane Chemistry. Synthesis of Regio- and Stereoisomerically Defined Enol Silyl Ethers Using Acylsilanes1. *J. Am. Chem. Soc.* **1990**, *112* (14), 5609–5617. https://doi.org/10.1021/ja00170a026.

(535) Engman, L.; Törnroos, K. W. Formation of 2-Phenylselenenylenones and 2-Haloenones from Enones. Mechanistic and Synthetic Aspects, X-Ray Crystal Structures of Intermediates. *J. Organomet. Chem.* **1990**, *391* (2), 165–178. https://doi.org/10.1016/0022-328X(90)80171-U.

(536) Rivera-Becerril, E.; Pérez-Hernández, N.; Joseph-Nathan, P.; Morales-Ríos, M. S. Structural Characterization of Isomeric Methyl 2-(2-Oxo-3-Indolyl)Acetate and Methyl 1-(2-Oxo-4-Quinolyl)Formate. *Heterocycles* **2006**, *68* (7), 1459–1466. https://doi.org/10.3987/COM-06-10759.

(537) Tategami, S. ichi; Yamada, T.; Nishino, H.; Korp, J. D.; Kurosawa, K. Formation of 1,2-Dioxacyclohexanes by the Reaction of Alkenes with Tris(2,4-Pentanedionato)Manganese(III) or with β-Ketocarbonyl Compounds in the Presence of Manganese(III) Acetate. *Tetrahedron Lett.* **1990**, *31* (44), 6371–6374. https://doi.org/10.1016/S0040-4039(00)97067-9.

(538) Wang, Z.; Chen, L.; Yao, Y.; Liu, Z.; Gao, J. M.; She, X.; Zheng, H. Dearomatization of Indole via Intramolecular [3 + 2] Cycloaddition: Access to the Pentacyclic Skeleton of Strychons Alkaloids. *Org. Lett.* **2018**, *20* (15), 4439–4443. https://doi.org/10.1021/acs.orglett.8b01720.

(539) Holmes, R. R.; Swamy, K. C. K.; Holmes, J. M.; Day, R. O. Conformational Effects of Ring Fusion and Heteroatom Substitution in Six-Membered Rings of Spirocyclic Oxyphosphoranes. *Inorg. Chem.* **1991**, *30* (5), 1052–1062. https://doi.org/10.1021/ic00005a033.

(540) Kurahashi, T.; Kozhushkov, S. I.; Schill, H.; Meindl, K.; Rühl, S.; De Meijere, A. 1,1′-Linked Cyclopropane Derivatives: The Helical Conformation of Quinquecyclopropanol. *Angew. Chem. Int. Ed.* **2007**, *46* (34), 6545–6548. https://doi.org/10.1002/anie.200702013.

(541) Tinant, B.; Declercq, J. P. Structure of N-Substituted 1,2-Oxazines. II. Bicyclic Bridged Derivatives. *Acta Cryst. C* **1991**, *47* (6), 1266–1269. https://doi.org/10.1107/s0108270190011659.

(542) Katritzky, A. R.; Khashab, N. M.; Haase, D. N.; Yoshioka, M.; Ghiviriga, I.; Steel, P. J. C-Aminoimidoylation and C-Thiocarbamoylation of Esters, Sulfones, and Ketones. *J. Org. Chem.* **2007**, *72* (18), 6742–6748. https://doi.org/10.1021/jo070545c.

(543) Höpfl, H.; Farfán, N.; Castillo, D.; Santillan, R.; Gutierrez, A.; Daran, J. C. Study of Cyclic Borinates Obtained from Piperidine- and Piperazine Alcohols by Spectroscopic Methods and X-Ray Crystallography. *J. Organomet. Chem.*

1998, *553* (1–2), 221–239. https://doi.org/10.1016/S0022-328X(97)00636-0.

(544) Bottle, S.; Busfield, W. K.; Jenkins, I. D.; Skelton, B. W.; White, A. H.; Rizzardo, E.; Solomon, D. H. Unexpected Products from the Reaction of Tert-Butoxyl Radicals with Acetylenes in the Presence of an Aminoxyl Radical Scavenger. *J. Chem. Soc. Perkin Trans. 2* **1991**, No. 7, 1001–1007. https://doi.org/10.1039/p29910001001.

(545) Lambrecht, S.; Schäfer, H. J.; Fröhlich, R.; Grehl, M. A Highly Stereoselective Synthesis of a Tetrahydrofluorene via a Domino Claisen Rearrangement Hetero-Ene Reaction. *Synlett* **1996**, *1996* (3), 283–284. https://doi.org/10.1055/s-1996-5373.

(546) Zhou, L.; Zhang, M.; Li, W.; Zhang, J. Furan-Based o-Quinodimethanes by Gold-Catalyzed Dehydrogenative Heterocyclization of 2-(1-Alkynyl)-2-Alken-1-Ones: A Modular Entry to 2,3-Furan-Fused Carbocycles. *Angew. Chem. Int. Ed.* **2014**, *53* (25), 6542–6545. https://doi.org/10.1002/anie.201403709.

(547) Purohit, V. C.; Matla, A. S.; Romo, D. Concise Synthesis of Spirocyclic, Bridged γ-Butyrolactones via Stereospecific, Dyotropic Rearrangements of β-Lactones Involving 1,2-Acyl and δ-Lactone Migrations. *J. Am. Chem. Soc.* **2008**, *130* (32), 10478–10479. https://doi.org/10.1021/ja803579z.

(548) Liu, B.; Wei, E.; Lin, S.; Zhao, B.; Liang, F. Synthesis of Spiro[Isoquinolinone-4,2′-Oxiranes] and Isoindolinones via a Multicomponent Reaction of 2-Acetyl-Oxirane-2-Carboxamides, Arylaldehydes and Malononitrile. *Chem. Commun.* **2014**, *50* (53), 6995–6997. https://doi.org/10.1039/c4cc02141j.

(549) Thomson, J. E.; Kyle, A. F.; Gallagher, K. A.; Lenden, P.; Concellón, C.; Morrill, L. C.; Miller, A. J.; Joannesse, C.; Slawin, A. M. Z.; Smith, A. D. N-Heterocyclic Carbene Catalysed Oxygen-to-Carbon Carboxyl Transfer of Indolyl and Benzofuranyl Carbonates. *Synthesis* **2008**, *2008* (17), 2805–2818. https://doi.org/10.1055/s-2008-1077890.

(550) Padwa, A.; Austin, D. J.; Xu, S. L. Control of Chemoselectivity in the Rhodium(II)-Catalyzed Alkyne Insertion Reaction of α-Diazo Ketones. *J. Org. Chem.* **1992**, *57* (5), 1330–1331. https://doi.org/10.1021/jo00031a007.

(551) Calvert, J. L.; Gordon, J. L. M.; Hartshorn, M. P.; Robinson, W. T.; Wright, G. J. The Nitration of Tetrachlorocatechol and 3, 4, 6-Trichloro-5-Methylcatechol. The Formation of 1-Hydroxycyclopent-3-Enecarboxylic Acids. *Aust. J. Chem.* **1992**, *45* (4), 713–719. https://doi.org/10.1071/CH9920713.

(552) Griffiths, N.; Sawyer, L.; Taylor, P.; Muir, G.; Peesapati, V.; Wilson, N. H.; Blake, A. J. Cis-7-Oxo-8-oxabicyclo[4.3.0]Non-2-ene-5-cis-carboxylic Acid. *Acta Cryst. C* **1992**, *48* (7), 1320–1322. https://doi.org/10.1107/S0108270191014014.

(553) Sugimoto, A.; Hiraoka, R.; Inoue, H.; Adachi, T. New Photochemical Cyclisation of 9-(6-Anilinohexyl)Phenanthrene. *J. Chem. Soc. Perkin Trans. 1* **1992**, No. 13, 1559–1560. https://doi.org/10.1039/p19920001559.

(554) Kendi, E.; Saraç, S.; Yarim, M.; Ertan, M.; Läge, M.; Krebs, B. 4-(2-Methylphenyl)-1,2,3,4,5,6,7,8-Octahydroquinazoline-2,5-Dione (I) and 4-(4-Chlorophenyl)-1,2,3,4,5,6,7,8-Octahydroquinazoline-2,5-Dione (II). *Cryst. Res. Technol.* **1997**, *32* (6), 857–863. https://doi.org/10.1002/crat.2170320619.

(555) Borah, M.; Gogoi, P.; Indukuri, K.; Saikia, A. K. Diastereoselective Synthesis of Substituted Tetrahydrothiophenes and -Thiopyrans via Thia-Prins Cyclization Reaction. *J. Org. Chem.* **2015**, *80* (5), 2641–2648. https://doi.org/10.1021/jo502831w.

(556) Ledovskaya, M. S.; Stepakov, A. V.; Molchanov, A. P.; Kostikov, R. R. An Efficient Synthesis of Substituted Spiro[Isoxazolopyrroloisoquinolines] via Diastereoselective N-Acyliminium Ion Cyclization. *Tetrahedron* **2015**, *71* (40), 7562–7566. https://doi.org/10.1016/j.tet.2015.08.007.

(557) Suwunwong, T.; Chantrapromma, S.; Fun, H. K. Synthesis, Fluorescence, TGA and Crystal Structure of Thiazolyl-Pyrazolines Derived from Chalcones. *Opt. Spectrosc.* **2015**, *118* (4), 563–573. https://doi.org/10.1134/S0030400X15040219.

(558) Lazareva, M. I.; Kryschenko, Y. K.; Caple, R.; Young, V. G.; Smit, W. A. Highly Diastereoselective One-Pot Four-Component Coupling of p-TolSCl, 1-Methoxycycloalkene, Methyl Vinyl Ether and a Carbon Nucleophile Leading to the Synthesis of Polyfunctional Compounds. *Mendeleev Commun.* **1999**, *9* (1), 24–25. https://doi.org/10.1070/mc1999v009n01abeh001044.

(559) Arbuzov, B. A.; Zabotina, E. Y.; Dianova, E. N.; Litvinov, I. A.; Naumov, V. A.; Latypov, S. K.; Il'asov, A. V. 5-Methyl-2-phenyl-2H-1,2,3-diazaarsole in Reaction with Ethyl Diazoacetate. *Heteroat. Chem.* **1992**, *3* (2), 151–156. https://doi.org/10.1002/hc.520030211.

(560) Song, M. Z. N′-(2-Hydroxy-1,2-Diphenylethylidene)Benzohydrazide. *Acta Cryst. E* **2010**, *66* (10), o2692–o2692. https://doi.org/10.1107/S1600536810038365.

(561) Marchand, A. P.; Annapurna, P.; Reddy, S. P.; Watson, W. H.; Nagl, A. Lewis Acid Promoted Reactions of

Substituted Pentacyclo[5.4.0.02,603,10.05,9]Undecane-8,11-Diones with Ethyl Diazoacetate. *J. Org. Chem.* **1989**, *54* (1), 187–193. https://doi.org/10.1021/jo00262a041.

(562) Song, Z.; Ji, Q.; Sun, R.; Matsuura, T.; Meng, J. The Helical Structure of a Photochromic Spironaphthopyran. *J. Mol. Struct.* **2003**, *655* (1), 31–35. https://doi.org/10.1016/S0022-2860(03)00184-4.

(563) Fernandez, M.; Chang, S.; Ziller, J.; Doedens, R. J.; Moore, H. W. Zinc Chloride Catalyzed Rearrangements of l,2-Dialkynyl-l,2-Dihydroxy-3,6-Dimethoxy-3,5-Cyclohexadienes to Bicyclo[2: 2.2]Octadienones and Bicyclo[32.1]Octadienones. *J. Org. Chem.* **1989**, *54* (6), 1379–1383. https://doi.org/10.1021/jo00267a028.

(564) Cheng, Y. A.; Yu, W. Z.; Yeung, Y. Y. An Unexpected Bromolactamization of Olefinic Amides Using a Three-Component Co-Catalyst System. *J. Org. Chem.* **2016**, *81* (2), 545–552. https://doi.org/10.1021/acs.joc.5b02390.

(565) Chen, Z. G.; Wang, Y.; Wei, J. F.; Zhao, P. F.; Shi, X. Y. K3PO4-Catalyzed Regiospecific Aminobromination of Bnitrostyrene Derivatives with N-Bromoacetamide as Aminobtominating Agent. *J. Org. Chem.* **2010**, *75* (6), 2085–2088. https://doi.org/10.1021/jo9026879.

(566) Sucrow, W.; Ellermann, K. -H; Flörke, U.; Haupt, H. -J. Enhydrazine, 39. Einige Stabile 1,4,5,6-Tetrahydro-1-methylpyridazin-3,4,5,6-tetracarbonsäureester Mit Axialen Ester-Gruppen. *Chem. Ber.* **1988**, *121* (11), 2007–2012. https://doi.org/10.1002/cber.19881211117.

(567) Gottschling, S. E.; Grant, T. N.; Milnes, K. K.; Jennings, M. C.; Baines, K. M. Cyclopropyl Alkynes as Mechanistic Probes to Distinguish between Vinyl Radical and Ionic Intermediates. *J. Org. Chem.* **2005**, *70* (7), 2686–2695. https://doi.org/10.1021/jo047797n.

(568) Kvita, V.; Sauter, H.; Rihs, G. Eine Neue Azepinring-Synthese. *Helv. Chim. Acta* **1989**, *72* (3), 457–463. https://doi.org/10.1002/hlca.19890720306.

(569) Zhang, J.; Chen, Z.; Wu, H. H.; Zhang, J. Ni(ClO4)2-Catalysed Regio- and Diastereoselective [3+2] Cycloaddition of Indoles and Aryl Oxiranyl-Dicarboxylates/Diketones: A Facile Access to Furo[3,4-b]Indoles. *Chem. Commun.* **2012**, *48* (12), 1817–1819. https://doi.org/10.1039/c2cc16918e.

(570) Weber, E.; Meinhold, D.; Haase, R.; Seichter, W.; Rheinwald, G. Inclusion Compounds of Bulky Binaphthyl-Type Bis-Fluorenol Hosts. *Supramol. Chem.* **2005**, *17* (4), 303–314. https://doi.org/10.1080/10610270500076245.

(571) Ruggiero, G.; Valente, E. J.; Eggleston, D. S. Structures of (±)-Cis-2-Hydroxy-4-(2-Phenylethyl)- (I) and Cis-4-Phenyl-2-Methyl-3,4-Dihydro-2H,5H-Pyrano[3,2-c][1]Benzopyran-5-One (II). *Acta Cryst. C* **1989**, *45* (9), 1369–1372. https://doi.org/10.1107/s0108270189001034.

(572) Hori, M.; Tadashi, T.; Shimizu, H.; Imai, E.; Kawamura, N.; Iwata, N.; Kurono, K.; Nakano, K.; Masayasu, M.; Kido, M. Synthesis and Analgesic Activity of Novel Heterocycles, [1]Benzothiopyrano[3,4-b]Pyrrole Derivatives. *Chem. Pharm. Bull.* **1989**, *37* (5), 1282–1286. https://doi.org/10.1248/cpb.37.1282.

(573) Atienza, R. L.; Roth, H. S.; Scheidt, K. A. N-Heterocyclic Carbene-Catalyzed Rearrangements of Vinyl Sulfones. *Chem. Sci.* **2011**, *2* (9), 1772–1776. https://doi.org/10.1039/c1sc00194a.

(574) Kim, S. Y.; Park, Y.; Son, S.; Chung, Y. K. Tandem Platinum Dichloride Catalysis and Thermal Reaction of Enynes: Versatile Synthetic Platform Based on Bicyclo[4.1.0]Hept-2-Enes. *Adv. Synth. Catal.* **2012**, *354* (1), 179–186. https://doi.org/10.1002/adsc.201100694.

(575) Ramos, A.; Antiñolo, A.; Carrillo-Hermosilla, F.; Fernández-Galán, R.; Montero-Rama, M. D. P.; Villaseñor, E.; Rodríguez-Diéguez, A.; García-Vivó, D. Insertion Reactions of Small Unsaturated Molecules in the N-B Bonds of Boron Guanidinates. *Dalt. Trans.* **2017**, *46* (31), 10281–10299. https://doi.org/10.1039/c7dt02081c.

(576) Xie, X.; Yue, G.; Tang, S.; Huo, X.; Liang, Q.; She, X.; Pan, X. Highly Diastereoselective Formation of 1,2,3-Trisubstituted Cyclopropane Derivatives. *Org. Lett.* **2005**, *7* (18), 4057–4059. https://doi.org/10.1021/ol051653t.

(577) Wdowiak, J.; Gdaniec, M.; Kosturkiewicz, Z. Structure of 2-(p-Toluenesulfonyl)-1,2,3,4-Tetrahydro-1-Isoquinolinecarbonitrile. *Acta Cryst. C* **1990**, *46* (1), 66–68. https://doi.org/10.1107/s0108270189004701.

(578) Zimmerman, H. E.; Lamers, P. H. Photochemistry of Some Extended π-Systems: Type A and Aryl Rearrangements of Systems With Extended Conjugation Related To Cyclohexadienones and Cyclohexenones. Mechanistic and Exploratory Organic Photochemistry. *J. Org. Chem.* **1989**, *54* (24), 5788–5804. https://doi.org/10.1021/jo00285a028.

(579) Sanzone, J. R.; Hu, C. T.; Woerpel, K. A. Uncatalyzed Carboboration of Seven-Membered-Ring Trans-Alkenes: Formation of Air-Stable Trialkylboranes. *J. Am. Chem. Soc.* **2017**, *139* (25), 8404–8407. https://doi.org/10.1021/jacs.7b03986.

(580) Hartshorn, M. P.; Robinson, W. T.; Grant Waller, A.; Wright, G. J. The Nitration of P-Cymene with Nitrogen Dioxide in Acetic Anhydride. *Aust. J. Chem.* **1989**, *42* (12), 2143–2160. https://doi.org/10.1071/CH9892143.

(581) Liang, L.; Dong, X.; Huang, Y. Phosphine-Mediated Sequential Annulation Reaction: Access to Functionalized Benzofurans and 4,5-Dihydrobenzofurans. *Chem. Eur. J.* **2017**, *23* (33), 7882–7886. https://doi.org/10.1002/chem.201701026.

(582) Holzinger, D.; Kickelbick, G. Modified Cubic Spherosilicates as Macroinitiators for the Synthesis of Inorganic-Organic Starlike Polymers. *J. Polym. Sci. Part A Polym. Chem.* **2002**, *40* (21), 3858–3872. https://doi.org/10.1002/pola.10476.

(583) Bong, I. C. C.; Ung, A. T.; Craig, D. C.; Scudder, M. L.; Bishop, R. Ritter Reactions. V: Further Investigation of the 3-Azatricyclo[5.3, 1.04, 9]Undec-2-Ene System. *Aust. J. Chem.* **1989**, *42* (11), 1929–1937. https://doi.org/10.1071/CH9891929.

(584) Liu, S.; Lan, X. C.; Chen, K.; Hao, W. J.; Li, G.; Tu, S. J.; Jiang, B. Ag/Brønsted Acid Co-Catalyzed Spiroketalization of β-Alkynyl Ketones toward Spiro[Chromane-2,1′-Isochromene] Derivatives. *Org. Lett.* **2017**, *19* (14), 3831–3834. https://doi.org/10.1021/acs.orglett.7b01705.

(585) Purohit, V. C.; Richardson, R. D.; Smith, J. W.; Romo, D. Practical, Catalytic, Asymmetric Synthesis of β-Lactones via a Sequential Ketene Dimerization/Hydrogenation Process: Inhibitors of the Thioesterase Domain of Fatty Acid Synthase. *J. Org. Chem.* **2006**, *71* (12), 4549–4558. https://doi.org/10.1021/jo060392d.

(586) Cioc, R. C.; Estévez, V.; van der Niet, D. J.; Vande Velde, C. M. L.; Turrini, N. G.; Hall, M.; Faber, K.; Ruijter, E.; Orru, R. V. A. Stereoselective Synthesis of Functionalized Bicyclic Scaffolds by Passerini 3-Center-2-Component Reactions of Cyclic Ketoacids. *Eur. J. Org. Chem.* **2017**, *2017* (9), 1262–1271. https://doi.org/10.1002/ejoc.201601432.

(587) Ohkita, M.; Lehn, J. M.; Baum, G.; Fenske, D. Helicity Coding: Programmed Molecular Self-Organization of Achiral Nonbiological Strands into Multiturn Helical Superstructures: Synthesis and Characterization of Alternating Pyridine-Pyrimidine Oligomers. *Chem. Eur. J.* **1999**, *5* (12), 3471–3481. https://doi.org/10.1002/(SICI)1521-3765(19991203)5:12<3471::AID-CHEM3471>3.0.CO;2-5.

(588) Xu, Y.; Liu, X. Y.; Wang, Z. H.; Tang, L. F. Synthesis of 3-Acyl, Methylene and Epoxy Substituted Isoindolinone Derivatives via the Ortho-Lithiation/Cyclization Procedures of Aromatic Imines with Carbon Monoxide. *Tetrahedron* **2017**, *73* (52), 7245–7253. https://doi.org/10.1016/j.tet.2017.11.001.

(589) Barluenga, J.; Mendoza, A.; Rodríguez, F.; Fañanás, F. J. A Palladium(II)-Catalyzed Synthesis of Spiroacetals through a One-Pot Multicomponent Cascade Reaction. *Angew. Chem. Int. Ed.* **2009**, *48* (9), 1644–1647. https://doi.org/10.1002/anie.200805519.

(590) Beaud, R.; Guillot, R.; Kouklovsky, C.; Vincent, G. FeCl3-Mediated Friedel-Crafts Hydroarylation with Electrophilic N-Acetyl Indoles for the Synthesis of Benzofuroindolines. *Angew. Chem. Int. Ed.* **2012**, *51* (50), 12546–12550. https://doi.org/10.1002/anie.201206611.

(591) Gharpure, S. J.; Vishwakarma, D. S.; Nanda, S. K. Lewis Acid Mediated “Endo-Dig” Hydroalkoxylation-Reduction on Internal Alkynols for the Stereoselective Synthesis of Cyclic Ethers and 1,4-Oxazepanes. *Org. Lett.* **2017**, *19* (24), 6534–6537. https://doi.org/10.1021/acs.orglett.7b03241.

(592) Carré, F.; Cerveau, G.; Chuit, C.; Corriu, R. J. P.; Nayyar, N. K.; Reyé, C. Hexacoordination at Silicon: The Case of Silatranes. *Organometallics* **1990**, *9* (7), 1989–1991. https://doi.org/10.1021/om00157a001.

(593) Simonneau, A.; Harrak, Y.; Jeanne-Julien, L.; Lemière, G.; Mouriès-Mansuy, V.; Goddard, J. P.; Malacria, M.; Fensterbank, L. Ring Expansions Within the Gold-Catalyzed Cycloisomerization of O-Tethered 1,6-Enynes. Application to the Synthesis of Natural-Product-like Macrocycles. *ChemCatChem* **2013**, *5* (5), 1096–1099. https://doi.org/10.1002/cctc.201200484.

(594) Cameron, T. S.; Linden, A.; Jochem, K. Structures of Cis-1,2-Bis(4-Cyanophenyl)-1,2-Bis(4-Methoxyphenyl)Cyclopropane (I), 1,1-Bis(4-Cyanophenyl)-2,2-Diphenylcyclopropane (II) and Trans-1-(4-Cyanophenyl)-2-(4-Methoxyphenyl)-1,2-Diphenylcyclopropane (III). *Acta Cryst. C* **1990**, *46* (11), 2110–2115. https://doi.org/10.1107/s0108270190000622.

(595) Zaytsev, V. P.; Mertsalov, D. F.; Nadirova, M. A.; Dorovatovskii, P. V.; Khrustalev, V. N.; Sorokina, E. A.; Zubkov, F. I.; Varlamov, A. V. [3+2] Cycloaddition of o-Nitrophenyl Azide to 3a,6-Epoxyisoindoles. *Chem. Heterocycl. Compd.* **2017**, *53* (11), 1199–1206. https://doi.org/10.1007/s10593-018-2194-1.

(596) Smith, T. J.; Langdon, A. G.; Wilkins, A. L.; Wilcock, R. J.; Coddington, J. M. A Revised Structure for Compound ‘2’ and X-Ray Crystal Structure of Compound C, Components of Technical Chlordane. *Aust. J. Chem.* **1990**, *43* (9), 1581–1586. https://doi.org/10.1071/CH9901581.

(597) Hartshorn, M. P.; Hayman, K. A.; Martyn, R. J.; Robinson, W. T.; Vaughan, J.; Wells, B. A.; Wright, G. J. The Chlorination of 4-Chloro-2, 3, 6- Trimethylphenol. Some Addition Reactions of 4, 6-Dichlorocyclohexa-2, 4-

Dienones. *Aust. J. Chem.* **1990**, *43* (10), 1729–1744. https://doi.org/10.1071/CH9901729.

(598) Golushko, A. A.; Sandzhieva, M. A.; Ivanov, A. Y.; Boyarskaya, I. A.; Khoroshilova, O. V.; Barkov, A. Y.; Vasilyev, A. V. Reactions of 3,3,3-Trihalogeno-1-Nitropropenes with Arenes in the Superacid CF3SO3H: Synthesis of (Z)-3,3,3-Trihalogeno-1,2-Diarylpropan-1-One Oximes and Study on the Reaction Mechanism. *J. Org. Chem.* **2018**, *83* (17), 10142–10157. https://doi.org/10.1021/acs.joc.8b01406.

(599) Buffet, J. C.; Turner, Z. R.; O'Hare, D. Popcorn-Shaped Polyethylene Synthesised Using Highly Active Supported Permethylindenyl Metallocene Catalyst Systems. *Chem. Commun.* **2018**, *54* (78), 10970–10973. https://doi.org/10.1039/c8cc05350b.

(600) Pizzarello, S.; Groy, T. L. Molecular Asymmetry in Extraterrestrial Organic Chemistry: An Analytical Perspective. *Geochim. Cosmochim. Acta* **2011**, *75* (2), 645–656. https://doi.org/10.1016/j.gca.2010.10.025.

(601) Hart, D. J.; Magomedov, N. Spiroquinazoline Support Studies: New Cascade Reactions Based on the Morin Rearrangement. *J. Org. Chem.* **1999**, *64* (9), 2990–2991. https://doi.org/10.1021/jo990147c.

(602) Mohler, P.; Rippert, A. J.; Hansen, H. J. Synthesis of (P)- and (M)-6,7-Bis[(Diphenylphosphanyl)Methyl]-8,12-Diphenylbenzo[a]Heptalenes - Potential Ligands for Homogeneous Asymmetric Catalysis. *Helv. Chim. Acta* **2000**, *83* (1), 258–277. https://doi.org/10.1002/(SICI)1522-2675(20000119)83:1<258::AID-HLCA258>3.0.CO;2-A.

(603) Wang, S.; Chai, Z.; Zhou, S.; Wang, S.; Zhu, X.; Wei, Y. A Novel Lewis Acid Catalyzed [3 + 3]-Annulation Strategy for the Syntheses of Tetrahydro-β-Carbolines and Tetrahydroisoquinolines. *Org. Lett.* **2013**, *15* (11), 2628–2631. https://doi.org/10.1021/ol4008525.

(604) Xing, Y.; Zhao, H.; Shang, Q.; Wang, J.; Lu, P.; Wang, Y. Parallel Copper Catalysis: Diastereoselective Synthesis of Polyfunctionalized Azetidin-2-Imines. *Org. Lett.* **2013**, *15* (11), 2668–2671. https://doi.org/10.1021/ol4010323.

(605) Xie, L.; Zhang, J.; Hu, H.; Cui, C. Synthesis and Reactions of π-Conjugated Iminoboranes Stabilized by Intramolecular Imine Groups. *Organometallics* **2013**, *32* (23), 6875–6878. https://doi.org/10.1021/om4008407.

(606) Willmes, P.; Cowley, M. J.; Hartmann, M.; Zimmer, M.; Huch, V.; Scheschkewitz, D. From Disilene (Si=Si) to Phosphasilene (Si=P) and Phosphacumulene (P=C=N). *Angew. Chem. Int. Ed.* **2014**, *53* (8), 2216–2220. https://doi.org/10.1002/anie.201308525.

(607) Ibbeson, B. M.; Laraia, L.; Alza, E.; O'Connor, C. J.; Tan, Y. S.; Davies, H. M. L.; Mckenzie, G.; Venkitaraman, A. R.; Spring, D. R. Diversity-Oriented Synthesis as a Tool for Identifying New Modulators of Mitosis. *Nat. Commun.* **2014**, *5* (1), 1–8. https://doi.org/10.1038/ncomms4155.

(608) Mihova, T. R.; Trifonov, L. S.; Dimitrov, V. S.; Orahovats, A. S.; Linden, A.; Heimgartner, H. New Polycyclic Compounds from Photochemical Rearrangements of Some Substituted 2-Azatricyclo[5.2.2.01,5]Undeca-4,8,10-trien-3-ones. *Helv. Chim. Acta* **1991**, *74* (5), 1011–1026. https://doi.org/10.1002/hlca.19910740512.

(609) Hosomi, H.; Ohba, S.; Aoyama, H. 2-Isopropyl-4-Methyl-4-Phenyl-1,2,3,4-Tetrahydroisoquinoline-1-Thione. *Acta Cryst. C* **2000**, *56* (4), e155. https://doi.org/10.1107/S0108270100004054.

(610) Osano, Y. T.; Mitsuhashi, K.; Maeda, S.; Matsuzaki, T. Structures of Photochromic Spiroindolinobenzoxazines and a Spiroindolinobenzopyran. *Acta Cryst. C* **1991**, *47* (10), 2137–2141. https://doi.org/10.1107/s0108270191003499.

(611) Nishino, S.; Hirano, K.; Miura, M. Copper-Catalyzed Electrophilic Amination of Gem-Diborylalkanes with Hydroxylamines Providing α-Aminoboronic Acid Derivatives. *Org. Lett.* **2019**, *21* (12), 4759–4762. https://doi.org/10.1021/acs.orglett.9b01640.

(612) Simanis, J. A.; Law, C. M.; Woodall, E. L.; Hamaker, C. G.; Goodell, J. R.; Mitchell, T. A. Investigation of Oxidopyrylium–Alkene [5+2] Cycloaddition Conjugate Addition Cascade (C3) Sequences. *Chem. Commun.* **2014**, *50* (65), 9130–9133. https://doi.org/10.1039/c4cc04163a.

(613) Schuster, D. I.; Heibel, G. E.; Woning, J. The Mechanism of Interaction of Triplet 3-Methylcyclohex-2-en-1-one with Maleo- and Fumarodinitrile: Evidence for Direct Formation of Triplet 1,4-Biradicals in [2 + 2] Photocyclo-Additions without the Intermediacy of Exciplexes. *Angew. Chem. Int. Ed.* **1991**, *30* (10), 1345–1347. https://doi.org/10.1002/anie.199113451.

(614) Wang, Y. Y.; Bode, J. W. Olefin Amine (OLA) Reagents for the Synthesis of Bridged Bicyclic and Spirocyclic Saturated N-Heterocycles by Catalytic Hydrogen Atom Transfer (HAT) Reactions. *J. Am. Chem. Soc.* **2019**, *141* (24), 9739–9745. https://doi.org/10.1021/jacs.9b05074.

(615) Han, G.; Liu, Y.; Wang, Q. Copper-Catalyzed Intramolecular Trifluoromethylation of N -Benzylacrylamides

Coupled with Dearomatization: Access to CF 3-Containing 2-Azaspiro[4.5]Decanes. *Org. Lett.* **2014**, *16* (12), 3188–3191. https://doi.org/10.1021/ol501054c.

(616) Steendam, R. R. E.; Verkade, J. M. M.; Van Benthem, T. J. B.; Meekes, H.; Van Enckevort, W. J. P.; Raap, J.; Rutjes, F. P. J. T.; Vlieg, E. Emergence of Single-Molecular Chirality from Achiral Reactants. *Nat. Commun.* **2014**, *5* (5543), 1–5. https://doi.org/10.1038/ncomms6543.

(617) Shi, T.; Guo, X.; Teng, S.; Hu, W. Pd(Ii)-Catalyzed Formal [4+1] Cycloaddition Reactions of Diazoacetates and Aryl Propargyl Alcohols to Form 2,5-Dihydrofurans. *Chem. Commun.* **2015**, *51* (82), 15204–15207. https://doi.org/10.1039/c5cc05000f.

(618) Bagchi, V.; Paraskevopoulou, P.; Das, P.; Chi, L.; Wang, Q.; Choudhury, A.; Mathieson, J. S.; Cronin, L.; Pardue, D. B.; Cundari, T. R.; Mitrikas, G.; Sanakis, Y.; Stavropoulos, P. A Versatile Tripodal Cu(I) Reagent for C-N Bond Construction via Nitrene-Transfer Chemistry: Catalytic Perspectives and Mechanistic Insights on C-H Aminations/Amidinations and Olefin Aziridinations. *J. Am. Chem. Soc.* **2014**, *136* (32), 11362–11381. https://doi.org/10.1021/ja503869j.

(619) Quan, Y.; Qiu, Z.; Xie, Z. Transition-Metal-Mediated Three-Component Cascade Cyclization: Selective Cage B-C(Sp2) Coupling of Carborane with Aromatics and Synthesis of Carborane-Fused Tricyclics. *J. Am. Chem. Soc.* **2014**, *136* (21), 7599–7602. https://doi.org/10.1021/ja503489b.

(620) Jones, P. G.; Dölle, A.; Kirby, A. J.; Parker, J. K. Bond Length and Reactivity. The Effect of β-Fluorine. Structures of the 4-Nitrophenyl Ether and the 4-Nitrobenzoate Ester of 2-Fluoro-2-Phenylethanol. *Acta Cryst. C* **1992**, *48* (5), 835–837. https://doi.org/10.1107/s0108270191012179.

(621) Costa, V. E. U.; Alifantes, J.; Mascarenhas, Y. P.; De Paula E Silva, C. H. T.; Seidl, P. R. Structures of Half-Cage Pentacyclododecane Derivatives: Skeletal Deformations Due to Steric Compression-Decompression. *J. Mol. Struct.* **2000**, *519* (1–3), 37–40. https://doi.org/10.1016/S0022-2860(99)00233-1.

(622) Schmidt, R.; Oh, J. H.; Sun, Y. Sen; Deppisch, M.; Krause, A. M.; Radacki, K.; Braunschweig, H.; Könemann, M.; Erk, P.; Bao, Z.; Würthner, F. High-Performance Air-Stable n-Channel Organic Thin Film Transistors Based on Halogenated Perylene Bisimide Semiconductors. *J. Am. Chem. Soc.* **2009**, *131* (17), 6215–6228. https://doi.org/10.1021/ja901077a.

(623) Engler, T.; Sampath, U. S.; Vander Velde, D.; Takusagawa, F. Further Studies On Quinone Diels-Alder Reactions With L, 3, 3-Trimethyl-2-Vinylcyclohexenes: Regioselective Synthesis Of 12-Methyl-Podocarpane Diterpenes and Isolation of a Hetero Diels-Alder Product from 14-Benzoquinone. *Synth. Commun.* **1992**, *22* (14), 2031–2042. https://doi.org/10.1080/00397919208021337.

(624) Baum, K.; Archibald, T. G.; Tzeng, D.; Gilardi, R.; Flippen-Anderson, J. L.; George, C. Synthesis and Properties of 1,2-Difluorodinitroethylene. *J. Org. Chem.* **1991**, *56* (2), 537–539. https://doi.org/10.1021/jo00002a011.

(625) Acosta, L. M.; Palma, A.; Bahsas, A.; Cobo, J.; Glidewell, C. A Three-Dimensional Hydrogen-Bonded Framework in (2S*,4R*)-7- Fluoro-2-Exo-[(E)-Styr-Yl]-2,3,4,5-Tetra-Hydro-1H-1,4-Ep-Oxy-1-Benzazepine. *Acta Cryst. C* **2010**, *66* (4), o206–o208. https://doi.org/10.1107/S010827011000884X.

(626) Acosta, L. M.; Palma, A.; Bahsas, A.; Cobo, J.; Glidewell, C. Hydrogen-Bonded Dimers, Chains and Rings in Six Differently Substituted 2-Vinyl-Tetra-Hydro-1,4-Ep-Oxy-1-Benz-Azepines. *Acta Cryst. C* **2010**, *66* (4), o209–o214. https://doi.org/10.1107/S0108270110009017.

(627) Ourahou, S.; Chammache, M.; Zouihri, H.; Essassi, E. M.; Ng, S. W. 2-Hydr-Oxy-10-Propargylpyrrolo[2,1-c][1,4]Benzodiazepine-5,11-Dione Monohydrate. *Acta Cryst. E* **2010**, *66* (4), 11. https://doi.org/10.1107/S1600536810006896.

(628) Ourahou, S.; Chammache, M.; Zouihri, H.; Essassi, E. M.; Ng, S. W. Ethyl 2-Hydr-Oxy-5,11-Dioxopyrrolo[2,1-c][1,4]Benzodiazepine-10-Acetate. *Acta Cryst. E* **2010**, *66* (4), o733–o733. https://doi.org/10.1107/S1600536810006914.

(629) Patil, P.; Khoury, K.; Herdtweck, E.; Dömling, A. MCR Synthesis of a Tetracyclic Tetrazole Scaffold. *Bioorganic Med. Chem.* **2015**, *23* (11), 2699–2715. https://doi.org/10.1016/j.bmc.2014.12.021.

(630) Yehye, W. A.; Ariffin, A.; Rahman, N. A.; Ng, S. W. 2-[4-Acetyl-5-(Biphenyl-4-Yl)-4,5-Dihydro-1,3,4-Oxadiazol-2-Yl]Phenyl Acetate. *Acta Cryst. E* **2010**, *66* (4), 4115–4117. https://doi.org/10.1107/S1600536810009621.

(631) Sidduri, A.; Knochel, P. New Preparation of α-Methylene-γ-Butyrolactones Mediated by (Iodomethyi)Zinc Iodide. *J. Am. Chem. Soc.* **1992**, *114* (19), 7579–7581. https://doi.org/10.1021/ja00045a050.

(632) Baktharaman, S.; Afagh, N.; Vandersteen, A.; Yudin, A. K. Unprotected Vinyl Aziridines: Facile Synthesis and Cascade Transformations. *Org. Lett.* **2010**, *12* (2), 240–243. https://doi.org/10.1021/ol902550q.

(633) Liu, X. G.; Wei, Y.; Shi, M. Probing Phosphane-Mediated [2+1] Annulation Reactions. *Eur. J. Org. Chem.* **2010**,

2010 (10), 1977–1988. https://doi.org/10.1002/ejoc.200901235.

(634) Rousselin, Y.; Sok, N.; Boschetti, F.; Guilard, R.; Denat, F. Efficient Synthesis of New C-Functionalized Macrocyclic Polyamines. *Eur. J. Org. Chem.* **2010**, *2010* (9), 1688–1693. https://doi.org/10.1002/ejoc.200901183.

(635) Ganesan, A.; Kothandapani, J.; Nanubolu, J. B.; Ganesan, S. S. Oleic Acid: A Benign Brønsted Acidic Catalyst for Densely Substituted Indole Derivative Synthesis. *RSC Adv.* **2015**, *5* (36), 28597–28600. https://doi.org/10.1039/c5ra02906f.

(636) Miura, T.; Funakoshi, Y.; Murakami, M. Intramolecular Dearomatizing [3 + 2] Annulation of α-Imino Carbenoids with Aryl Rings Furnishing 3,4-Fused Indole Skeletons. *J. Am. Chem. Soc.* **2014**, *136* (6), 2272–2275. https://doi.org/10.1021/ja412663a.

(637) Kang, T. R. 3-(3-Cyanophenyl)-N-Phenyloxirane-2-Carboxamide. *Acta Cryst. E* **2010**, *66* (10), o2628–o2628. https://doi.org/10.1107/S1600536810037475.

(638) Takido, T.; Tamura, S.; Sato, K.; Kamijo, H.; Nakazawa, T.; Hata, T.; Seno, M. The Synthesis of Hexahydrooxoepithiopyridinedicarboximides by the Reaction of Thioamides with N-Substituted Maleimides. *J. Heterocycl. Chem.* **1998**, *35* (2), 437–443. https://doi.org/10.1002/jhet.5570350229.

(639) Quesada, E.; Stockley, M.; Ragot, J. P.; Prime, M. E.; Whitwood, A. C.; Taylor, R. J. K. A Versatile, Non-Biomimetic Route to the Preussomerins: Syntheses of (±)-Preussomerins F, K and L. *Org. Biomol. Chem.* **2004**, *2* (17), 2483–2495. https://doi.org/10.1039/b407895k.

(640) Brown, C. C.; Glotzbach, C.; Stephan, D. W. Ag(i) and Au(i) Complexes of Sterically Crowded Cyclic Phosphinimine Ligands. *Dalt. Trans.* **2010**, *39* (40), 9626–9632. https://doi.org/10.1039/c0dt00261e.

(641) Letcher, R. M.; Sin, D. W. M.; Cheung, K. K. Oxazolo[3,2-a]Indoles, Pyrrolo- and Azepino-[1,2-a]Indoles from 3H-Indole 1-Oxides and Acetylenecarboxylic Esters by Skeletal Rearrangements. *J. Chem. Soc. Perkin Trans. 1* **1993**, No. 8, 939–944. https://doi.org/10.1039/p19930000939.

(642) Roesky, H. W.; May, A.; Noltemeyer, M. Synthese von Heterocyclen Durch Verwendung von Bis(Trifluormethyl)Sulfin-Imiden. *J. Fluor. Chem.* **1993**, *62* (1), 77–99. https://doi.org/10.1016/S0022-1139(00)80083-4.

(643) Monbaliu, J. C.; Tinant, B.; Marchand-Brynaert, J. Cycloadditions of 1-Phosphono-1,3-Butadienes with Nitroso Heterodienophiles: A Versatile Synthetic Route for Polyfunctionalized Aminophosphonic Derivatives. *J. Org. Chem.* **2010**, *75* (16), 5478–5486. https://doi.org/10.1021/jo100230r.

(644) Ianelli, S.; Nardelli, M.; Belletti, D.; Jamart-Grégoire, B.; Brosse, N.; Caubère, P. Synthesis and Structure of Strained Polycyclic Cyclobutane-containing Derivatives. *Acta Cryst. C* **1993**, *49* (7), 1388–1392. https://doi.org/10.1107/S0108270193000319.

(645) Fox, D. J.; Morley, T. J.; Taylor, S.; Warren, S. Selective Five- and Six-Membered Cyclic Amine Syntheses via Capture of Episulfonium Ions. *Org. Biomol. Chem.* **2005**, *3* (8), 1369–1371. https://doi.org/10.1039/b503068b.

(646) Sterkhova, I. V.; Astakhova, V. V.; Shainyan, B. A. X-Ray, FTIR and DFT Study of New Iodine-Containing Derivatives of Trifluoroacetamide. *J. Mol. Struct.* **2017**, *1141*, 351–356. https://doi.org/10.1016/j.molstruc.2017.03.095.

(647) Yasuda, M.; Tanaka, S. Y.; Baba, A. Reductive Cross-Aldol Reaction Using Bromoaldehyde and an Aldehyde Mediated by Germanium(II): One-Pot, Large-Scale Protocol. *Org. Lett.* **2005**, *7* (9), 1845–1848. https://doi.org/10.1021/ol050533i.

(648) Moerdyk, J. P.; Bielawski, C. W. Diamidocarbenes as Versatile and Reversible [2+1] Cycloaddition Reagents. *Nat. Chem.* **2012**, *4* (4), 275–280. https://doi.org/10.1038/nchem.1267.

(649) Egorov, V. A.; Khasanova, L. S.; Gimalova, F. A.; Lobov, A. N.; Miftakhov, M. S. Straightforward Synthesis of Pyrrolizidines. *Mendeleev Commun.* **2017**, *27* (2), 163–165. https://doi.org/10.1016/j.mencom.2017.03.019.

(650) Tähtinen, P.; Sillanpää, R.; Stájer, G.; Szabó, A. E.; Pihlaja, K. NMR and X-Ray Structural Study of Saturated (p-Chlorophenyl)-Pyrrolo[1,2-a][3,1]Benzoxazin-1-Ones Prepared from Aroylisobutyric Acid and Cyclic Amino Alcohols. High Energy Barriers for Hindered Rotation of Bridgehead Phenyl Groups. *J. Chem. Soc. Perkin Trans. 2* **1999**, No. 10, 2011–2021. https://doi.org/10.1039/a905445f.

(651) Tang, Z.; Xia, Z.; Li, X. Stereoselective Synthesis, Crystal Structure and Fungicidal Activity of Trans-2,3,6(8)-Trisubstituted-1,3-Benzoxazines. *Chinese J. Org. Chem.* **2016**, *36* (3), 590–595. https://doi.org/10.6023/cjoc201508019.

(652) Szučová, L.; Trávníček, Z.; Zatloukal, M.; Popa, I. Novel Platinum(II) and Palladium(II) Complexes with Cyclin-Dependent Kinase Inhibitors: Synthesis, Characterization and Antitumour Activity. *Bioorganic Med. Chem.* **2006**, *14* (2), 479–491. https://doi.org/10.1016/j.bmc.2005.08.033.

(653) Rizzoli, C.; Sgarabotto, P.; Ugozzoli, F.; Carloni, P.; Damiani, E.; Greci, L.; Stipa, P. Conformational Study on Indoline Compounds. Structures of 2-phenyl-3-arylimino-3H-indole 1-Oxide, 1,2-dihydro-2-phenyl-2-benzyl- and 2-tert-Butyl-3-phenylimino-3H-indole 1-oxyls. *J. Heterocycl. Chem.* **1993**, *30* (3), 637–642. https://doi.org/10.1002/jhet.5570300310.

(654) Guideri, L.; Noschese, R.; Ponticelli, F. Tetrahydropyridinium Bromide: Useful Synthon to Functionalized Pyrrolidines. *J. Heterocycl. Chem.* **2012**, *49* (2), 297–302. https://doi.org/10.1002/jhet.823.

(655) Borisov, D. D.; Novikov, R. A.; Tomilov, Y. V. Highly Diastereoselective Formation of 3,7-Dioxabicyclo[3.3.0]Octan-2-Ones in Reaction of 2-Arylcyclopropanedicarboxylates with Aromatic Aldehydes Using 1,2-Zwitterionic Reactivity Type. *Tetrahedron Lett.* **2017**, *58* (38), 3712–3716. https://doi.org/10.1016/j.tetlet.2017.08.025.

(656) He, L. 2,2-Diethyl 3,4-Dimethyl 5-(4-Cyano-Phen-Yl)Pyrrolidine-2,2,3,4-Tetra- Carboxyl-Ate. *Acta Cryst. E* **2012**, *68* (8), 4–5. https://doi.org/10.1107/S1600536812029625.

(657) Ravichandran, K.; Sethuvasan, S.; Thirunavukarasu, K.; Ponnuswamy, S.; Ponnuswamy, M. N. 3-Isopropyl-2,6-Bis-(4-Meth-Oxy-Phen-Yl)-Piperidin-4-One. *Acta Cryst. E* **2012**, *68* (8), o2453–o2453. https://doi.org/10.1107/S1600536812030966.

(658) Baeg, J. O.; Alper, H. Novel Palladium(II)-Catalyzed Cyclization of Aziridines and Sulfur Diimides. *J. Am. Chem. Soc.* **1994**, *116* (4), 1220–1224. https://doi.org/10.1021/ja00083a007.

(659) Sephton, S. M.; Mu, L.; Schweizer, W. B.; Schibli, R.; Krämer, S. D.; Ametamey, S. M. Synthesis and Evaluation of Novel α-Fluorinated (E)-3-((6- Methylpyridin-2-Yl)Ethynyl)Cyclohex-2-Enone-O-Methyl Oxime (ABP688) Derivatives as Metabotropic Glutamate Receptor Subtype 5 PET Radiotracers. *J. Med. Chem.* **2012**, *55* (16), 7154–7162. https://doi.org/10.1021/jm300648b.

(660) Bieńko, D. C.; Michalska, D.; Borrell, J. I.; Teixidó, J.; Matallana, J. L.; Alvarez-Larena, A.; Piniella, J. F. Structure of 2-Cyano-4-Phenyl-Glutarimide Studied by X-Ray Diffraction, Vibrational Spectroscopy and Ab Initio Methods. *J. Mol. Struct.* **1998**, *471* (1–3), 49–56. https://doi.org/10.1016/S0022-2860(98)00382-2.

(661) Gorin, D. J.; Dubé, P.; Toste, F. D. Synthesis of Benzonorcaradienes by Gold(I)-Catalyzed [4+3] Annulation. *J. Am. Chem. Soc.* **2006**, *128* (45), 14480–14481. https://doi.org/10.1021/ja066694e.

(662) Hili, R.; Yudin, A. K. Readily Available Unprotected Amino Aldehydes. *J. Am. Chem. Soc.* **2006**, *128* (46), 14772–14773. https://doi.org/10.1021/ja065898s.

(663) Schmidt, K.; Kopf, J.; Margaretha, P. Light-Induced Cycloaddition of 2,3-Dihydro-2,2-Dimethyl-4H-Thiopyran-4-One (a 4-Thiacyclohex-2-Enone) to Alkenes and Dienes. *Helv. Chim. Acta* **2005**, *88* (7), 1922–1930. https://doi.org/10.1002/hlca.200590147.

(664) Guo, H. M.; Jian, F. F.; Xiao, H. L.; Sun, X. Z.; Li, Y. F. 3-(4-Bromo-Phen-Yl)-5-(4-Chloro-Phen-Yl)-1-Phenyl-2-Pyrazoline. *Acta Cryst. E* **2007**, *63* (3), o1435–o1436. https://doi.org/10.1107/S160053680700102X.

(665) Kirschke, K.; Baumann, H.; Costisella, B.; Ramm, M. Lithium-selektive Chromoionophore Vom Arylazobutenoat-Typ. *Liebigs Ann. der Chemie* **1994**, *1994* (3), 265–268. https://doi.org/10.1002/jlac.199419940307.

(666) Główka, M. L. Crystal Structure of Enantiomeric 3-Amino-6,7-Dihydro-6-Hydroxy-5 H-1,2,4-Triazolo [3,4-b] 1,3-Thiazine. *J. Chem. Crystallogr.* **1994**, *24* (4), 273–276. https://doi.org/10.1007/BF01670356.

(667) Renou, L.; Morelli, T.; Coste, S.; Petit, M. N.; Berton, B.; Malandain, J. J.; Coquerel, G. Chiral Discrimination at the Solid State of Methyl 2- (Diphenylmethylsulfinyl)Acetate. In *Crystal Growth and Design*; American Chemical Society , 2007; Vol. 7, pp 1599–1607. https://doi.org/10.1021/cg070075f.

(668) Bajtos, B.; Yu, M.; Zhao, H.; Pagenkopf, B. L. C-2/C-3 Annulation and C-2 Alkylation of Indoles with 2-Alkoxycyclopropanoate Esters. *J. Am. Chem. Soc.* **2007**, *129* (31), 9631–9634. https://doi.org/10.1021/ja067821+.

(669) Halli, J.; Kramer, P.; Grimmer, J.; Bolte, M.; Manolikakes, G. Bi(OTf)3-Catalyzed Diastereoselective One-Pot Synthesis of 1,3-Diamines with Three Continuous Stereogenic Centers. *J. Org. Chem.* **2018**, *83* (19), 12007–12022. https://doi.org/10.1021/acs.joc.8b01925.

(670) Kamimura, D.; Urabe, D.; Nagatomo, M.; Inoue, M. Et3B-Mediated Radical-Polar Crossover Reaction for Single-Step Coupling of O,Te-Acetal, α,β-Unsaturated Ketones, and Aldehydes/Ketones. *Org. Lett.* **2013**, *15* (19), 5122–5125. https://doi.org/10.1021/ol402563v.

(671) Kumar, R.; Chaudhary, S.; Kumar, R.; Upadhyay, P.; Sahal, D.; Sharma, U. Catalyst and Additive-Free Diastereoselective 1,3-Dipolar Cycloaddition of Quinolinium Imides with Olefins, Maleimides, and Benzynes: Direct Access to Fused N,N′-Heterocycles with Promising Activity against a Drug-Resistant Malaria Parasite. *J. Org. Chem.* **2018**, *83* (19), 11552–11570. https://doi.org/10.1021/acs.joc.8b01520.

(672) Shen, Y. M.; Duan, W. L.; Shi, M. Chemical Fixation of Carbon Dioxide Co-Catalyzed by a Combination of Schiff Bases or Phenols and Organic Bases. *Eur. J. Org. Chem.* **2004**, *2004* (14), 3080–3089. https://doi.org/10.1002/ejoc.200400083.

(673) Chen, J.; Tian, J.; Liu, F.; Liu, Y.; Zhao, G.; Yuan, W.; Zhao, B. Intramolecular Umpolung Synthesis of Exocyclic β-Amino Alcohols through Decarboxylative Amination. *ACS Omega* **2018**, *3* (11), 14671–14679. https://doi.org/10.1021/acsomega.8b02324.

(674) Sun, J.; Xie, Y. J.; Yan, C. G. Construction of Dispirocyclopentanebisoxindoles via Self-Domino Michael-Aldol Reactions of 3-Phenacylideneoxindoles. *J. Org. Chem.* **2013**, *78* (17), 8354–8365. https://doi.org/10.1021/jo4010603.

(675) Winner, L.; Ewing, W. C.; Geetharani, K.; Dellermann, T.; Jouppi, B.; Kupfer, T.; Schäfer, M.; Braunschweig, H. Spontaneous Metal-Free Transfer Hydrogenation of Iminoboranes with Ammonia Borane and Amine Boranes. *Angew. Chem. Int. Ed.* **2018**, *57* (38), 12275–12279. https://doi.org/10.1002/anie.201807435.

(676) Aslam, N. A.; Babu, S. A.; Sudha, A. J.; Yasuda, M.; Baba, A. Chelation-Controlled Diastereoselective Construction of N-Aryl-, N-Acyl/Tosylhydrazono β-Substituted Aspartate Derivatives via Barbier-Type Reaction. *Tetrahedron* **2013**, *69* (32), 6598–6611. https://doi.org/10.1016/j.tet.2013.05.130.

(677) Tran, Y. S.; Kwon, O. Phosphine-Catalyzed [4 + 2] Annulation: Synthesis of Cyclohexenes. *J. Am. Chem. Soc.* **2007**, *129* (42), 12632–12633. https://doi.org/10.1021/ja0752181.

(678) Chagarovskiy, A. O.; Strel'tsova, E. D.; Rybakov, V. B.; Levina, I. I.; Trushkov, I. V. Synthesis of 2,3-Diaryl-2,3,4,4a-Tetrahydro-5H-Indeno[1,2-c]Pyridazin-5-Ones. *Chem. Heterocycl. Compd.* **2019**, *55* (3), 240–245. https://doi.org/10.1007/s10593-019-02448-y.

(679) Yaremenko, F.; Beryozkina, T.; Khvat, A.; Svidlo, I.; Shishkin, O.; Shishkina, S.; Orlov, V. 5,7-Substituted Thiazolo[2,3-a]Pyrimidines: Synthesis, Stereochemistry and Crystal Structure. *J. Mol. Struct.* **2008**, *874* (1–3), 57–63. https://doi.org/10.1016/j.molstruc.2007.03.031.

(680) Mori, K.; Kurihara, K.; Akiyama, T. Expeditious Synthesis of 1-Aminoindane Derivatives Achieved by [1,4]-Hydride Shift Mediated C(Sp3)–H Bond Functionalization. *Chem. Commun.* **2014**, *50* (28), 3729–3731. https://doi.org/10.1039/c4cc00894d.

(681) Bornhöft, J.; Siegwarth, J.; Näther, C.; Herges, R. Synthesis of Annulenes via Multiple Wittig Reactions; in-Situ Dimerization of 1,2;5,6-Dibenzocyclooctatetraene. *Eur. J. Org. Chem.* **2008**, *2008* (9), 1619–1624. https://doi.org/10.1002/ejoc.200701174.

(682) Carroccia, L.; Degennaro, L.; Romanazzi, G.; Cuocci, C.; Pisano, L.; Luisi, R. Straightforward Access to 4-Membered Sulfurated Heterocycles: Introducing a Strategy for the Single and Double Functionalization of Thietane 1-Oxide. *Org. Biomol. Chem.* **2014**, *12* (14), 2180–2184. https://doi.org/10.1039/c4ob00173g.

(683) Suresh, A.; Baiju, T. V.; Kumar, T.; Namboothiri, I. N. N. Synthesis of Spiro- and Fused Heterocycles via (4+4) Annulation of Sulfonylphthalide with o-Hydroxystyrenyl Derivatives. *J. Org. Chem.* **2019**, *84* (6), 3158–3168. https://doi.org/10.1021/acs.joc.8b03039.

(684) Maison, W.; Lützen, A.; Kosten, M.; Sclilcmminger, I.; Westcrhoff, O.; Saak, W.; Martens, J. Multicomponent Synthesis of Tripeptides Containing Pipecolic Acid Derivatives: Selective Induction of Cis- And Tmns-Imide Bonds into Peptide Backbones. *J. Chem. Soc. Perkin Trans. 1* **2000**, No. 12, 1867–1871. https://doi.org/10.1039/b002258f.

(685) Roversi, E.; Monnat, F.; Schenk, K.; Vogel, P.; Braña, P.; Sordo, J. A. 17O NMR Spectroscopy of Sulfolenes (2,5-Dihydrothiophene-1,1-Dioxides) and Sultines (3,6-Dihydro-1,2-Oxathiin-2-Oxides) - Experiment and Quantum Calculations: Synthesis of 4,9-Dioxo-1,2-Oxathiacyclodecane-2-Oxide, a New Heterocycle. *Chem. Eur. J.* **2000**, *6* (10), 1858–1864. https://doi.org/10.1002/(sici)1521-3765(20000515)6:10<1858::aid-chem1858>3.0.co;2-u.

(686) Zubkov, F. I.; Nikitina, E. V.; Galeev, T. R.; Zaytsev, V. P.; Khrustalev, V. N.; Novikov, R. A.; Orlova, D. N.; Varlamov, A. V. General Synthetic Approach towards Annelated 3a,6-Epoxyisoindoles by Tandem Acylation/IMDAF Reaction of Furylazaheterocycles. Scope and Limitations. *Tetrahedron* **2014**, *70* (8), 1659–1690. https://doi.org/10.1016/j.tet.2014.01.008.

(687) Rawling, M. J.; Rowley, J. H.; Campbell, M.; Kennedy, A. R.; Parkinson, J. A.; Tomkinson, N. C. O. Mechanistic Insights into the Malonoyl Peroxide Syn-Dihydroxylation of Alkenes. *Chem. Sci.* **2014**, *5* (5), 1777–1785. https://doi.org/10.1039/c3sc53256a.

(688) Yang, Y.; Schießl, J.; Zallouz, S.; Göker, V.; Gross, J.; Rudolph, M.; Rominger, F.; Hashmi, A. S. K. Gold-Catalyzed C(Sp2)−C(Sp) Coupling by Alkynylation through Oxidative Addition of Bromoalkynes. *Chem. Eur. J.* **2019**, *25* (41), 9624–9628. https://doi.org/10.1002/chem.201902213.

(689) Raj, A. S. K.; Liu, R. S. Gold-Catalyzed Bicyclic Annulations of 2-Alkynylbenzaldehydes with Vinyldiazo Carbonyls That Serve as Five-Atom Building Units. *Angew. Chem. Int. Ed.* **2019**, *58* (32), 10980–10984. https://doi.org/10.1002/anie.201905350.

(690) Annaka, T.; Nakata, N.; Ishii, A. A Reversible and Turn-on Type Fluorescence Behaviour of Hydrogen Sulfide via a Redox Cycle between Selenoxide and Selenide. *New J. Chem.* **2019**, *43* (29), 11643–11652. https://doi.org/10.1039/c9nj02813g.

(691) Lan, P.; Jackson, C. J.; Banwell, M. G.; Willis, A. C. Synthesis of a D-Ring Isomer of Galanthamine via a Radical-Based Smiles Rearrangement Reaction. *J. Org. Chem.* **2014**, *79* (14), 6759–6764. https://doi.org/10.1021/jo501255c.

(692) Zhou, X.; Xu, H.; Yang, Q.; Chen, H.; Wang, S.; Zhao, H. Co(II)/Cu(II)-Cocatalyzed Oxidative C-H/N-H Functionalization of Benzamides with Ketones: A Facile Route to Isoindolin-1-Ones. *Chem. Commun.* **2019**, *55* (59), 8603–8606. https://doi.org/10.1039/c9cc02661d.

(693) Paul, M.; Neudörfl, J. M.; Berkessel, A. Breslow Intermediates from a Thiazolin-2-Ylidene and Fluorinated Aldehydes: XRD and Solution-Phase NMR Spectroscopic Characterization. *Angew. Chem. Int. Ed.* **2019**, *58* (31), 10596–10600. https://doi.org/10.1002/anie.201904308.

(694) Shi, M.; Jiang, J. K.; Feng, Y. S. Titanium(IV) Chloride and the Amine-Promoted Baylis-Hillman Reaction. *Org. Lett.* **2000**, *2* (16), 2397–2400. https://doi.org/10.1021/ol000046x.

(695) Von Zezschwitz, P.; Voigt, K.; Noltemeyer, M.; De Meijere, A. A New Methodology for Ring Enlargements by the Oxy-Cope Rearrangement: Ready Access to Highly Functionalized Medium-Size Rings. *Synthesis*. Georg Thieme Verlag December 31, 2000, pp 1327–1340. https://doi.org/10.1055/s-2000-6427.

(696) Malassene, R.; Toupet, L.; Hurvois, J. P.; Moinet, C. Synthesis of 1-Azaspiroundecane Ring System via Thorpe-Ziegler Annulation of 2-Cyano-2-(4-Cyano-Tethered) Arylpiperidines. *Synlett* **2002**, *2002* (6), 895–898. https://doi.org/10.1055/s-2002-31904.

(697) Gayen, B.; Banerji, A. Simple and Efficient Routes to Substituted Oxazolidine and Spiro-Oxindole Systems by One-Pot Synthetic Strategies. *Monatshefte fur Chemie* **2014**, *145* (12), 1953–1965. https://doi.org/10.1007/s00706-014-1269-7.

(698) Von Seebach, M.; Grigg, R.; De Meijere, A. Multicomponent Queuing Cascades of Bicyclopropylidene, Carbon Monoxide and Aryl Iodides or Aryl Thiols. *Eur. J. Org. Chem.* **2002**, *2002* (19), 3268–3275. https://doi.org/10.1002/1099-0690(200210)2002:19<3268::AID-EJOC3268>3.0.CO;2-H.

(699) Bower, J.; Box, M. R.; Czyzewski, M.; Goeta, A. E.; Steel, P. G. Acyl Polysilanes: New Acyl Anion Equivalents for Additions to Electron-Deficient Alkenes. *Org. Lett.* **2009**, *11* (13), 2744–2747. https://doi.org/10.1021/ol900813z.

(700) Qin, T.; Cheng, L.; Zhang, S. X. A.; Liao, W. Stereoselective Synthesis of Organosulfur Compounds Incorporating N-Aromatic Heterocyclic Motifs and Quaternary Carbon Centers via a Sulfa-Michael Triggered Tandem Reaction. *Chem. Commun.* **2015**, *51* (47), 9714–9717. https://doi.org/10.1039/c5cc01875g.

(701) Hossain, S.; Gupta, S. K.; Murugavel, R. 2,6-Dimethylphenol Derived H-Phosphonate and α-Hydroxyphosphonate: Facile Synthesis, Crystal Chemistry, Supramolecular Association and Metal Complexation. *CrystEngComm* **2015**, *17* (23), 4355–4366. https://doi.org/10.1039/c5ce00675a.

(702) Khadem, S.; Udachin, K. A.; Enright, G. D.; Prakesch, M.; Arya, P. One-Pot Construction of Isoindolo[2,1-a]Quinoline System. *Tetrahedron Lett.* **2009**, *50* (48), 6661–6664. https://doi.org/10.1016/j.tetlet.2009.09.075.

(703) Honda, M.; Nishizawa, T.; Nishii, Y.; Fujinami, S.; Segi, M. Reaction Behavior of Cyclopropylmethyl Cations Derived 1-Phenylselenocyclopropylmethanols with Acids. *Tetrahedron* **2009**, *65* (45), 9403–9411. https://doi.org/10.1016/j.tet.2009.08.082.

(704) Deligny, M.; Carreaux, F.; Carboni, B.; Toupet, L.; Dujardin, G. A Novel Diastereoselective Route to α-Hydroxyalkyl Dihydropyrans Using a Hetero Diels-Alder/Allylboration Sequence. *Chem. Commun.* **2003**, No. 2, 276–277. https://doi.org/10.1039/b208572k.

(705) Castillo, J. C.; Quiroga, J.; Abonia, R.; Rodriguez, J.; Coquerel, Y. Pseudo-Multicomponent Reactions of Arynes with N-Aryl Imines. *J. Org. Chem.* **2015**, *80* (19), 9767–9773. https://doi.org/10.1021/acs.joc.5b01564.

(706) Horvat, M.; Görner, H.; Warzecha, K. D.; Neudörfl, J.; Griesbeck, A. G.; Mlinarić-Majerski, K.; Basarić, N. Photoinitiated Domino Reactions: N-(Adamantyl)Phthalimides and N-(Adamantylalkyl)Phthalimides. *J. Org. Chem.* **2009**, *74* (21), 8219–8231. https://doi.org/10.1021/jo901753z.

(707) Hosoya, T.; Ohhara, T.; Uekusa, H.; Ohashi, Y. Crystalline-State Photoisomerization of α,β-Unsaturated Thioamide Analyzed by x-Rays. *Bull. Chem. Soc. Jpn.* **2002**, *75* (10), 2147–2151.

https://doi.org/10.1246/bcsj.75.2147.

(708) Jones, N. D.; Meessen, P.; Smith, M. B.; Losehand, U.; Rettig, S. J.; Patrick, B. O.; James, B. R. Bisphosphine Ligands Containing Two O-N,N-Dimethylanilinyl Substituents at Each Phosphorus Atom. *Can. J. Chem.* **2002**, *80* (11), 1600–1606. https://doi.org/10.1139/v02-127.

(709) Tənase, C. I.; Drəghici, C.; Shova, S.; Cojocaru, A.; Maganu, M.; Munteanu, C. V. A.; Cocu, F. Regioselective Reactions on a 1,3-Disubstituted Dihydroxymethyl or Dicarboxyl Hexahydropentalene Skeleton. *Tetrahedron* **2015**, *71* (38), 6852–6859. https://doi.org/10.1016/j.tet.2015.07.021.

(710) Buchmeiser, M. R.; Lubbad, S.; Mayr, M.; Wurst, K. Access to Silica- and Monolithic Polymer Supported C-C-Coupling Catalysts via ROMP: Applications in High-Throughput Screening, Reactor Technology and Biphasic Catalysis. *Inorganica Chim. Acta* **2003**, *345*, 145–153. https://doi.org/10.1016/S0020-1693(02)01291-4.

(711) Ramachandiran, K.; Karthikeyan, K.; Muralidharan, D.; Perumal, P. T. Synthesis of Isoxazolobenzoxepanes via Michael Addition of Indoles to Nitroalkenes and Sequential Intramolecular Nitrile Oxide Cycloaddition. *Tetrahedron Lett.* **2010**, *51* (22), 3006–3009. https://doi.org/10.1016/j.tetlet.2010.04.001.

(712) Chen, Z.; Zhang, Y. X.; Wang, Y. H.; Zhu, L. L.; Liu, H.; Li, X. X.; Guo, L. Gold Catalyzed Diastereoselective Cascade Allylation/Enyne Cycloisomerization to Construct Densely Functionalized Oxygen Hetereocycles. *Org. Lett.* **2010**, *12* (15), 3468–3471. https://doi.org/10.1021/ol1012923.

(713) Yang, G.; Raptis, R. G. Synthesis, Characterization and Crystal Structures of Two 2-Naphthyl Substituted Pyrazoles. *J. Heterocycl. Chem.* **2003**, *40* (4), 659–664. https://doi.org/10.1002/jhet.5570400416.

(714) Madelaine, C.; Buriez, O.; Crousse, B.; Florent, I.; Grellier, P.; Retailleau, P.; Six, Y. Aminocyclopropanes as Precursors of Endoperoxides with Antimalarial Activity. *Org. Biomol. Chem.* **2010**, *8* (24), 5591–5601. https://doi.org/10.1039/c0ob00308e.

(715) Bremner, J. B.; Russell, H. F.; Skelton, B. W.; White, A. H. Novel Indole-Fused, Medium-Sized Ring Heterocycles via Chloroacetamide Photochemistry. *Heterocycles* **2000**, *53* (2), 277–290. https://doi.org/10.3987/com-99-8737.

(716) Barton, B.; Caira, M. R.; McCleland, C. W.; Taljaard, B. Synthesis of N,N′-Bis(9-Phenylxanthen-9-Yl)Ethylenediamine and an Investigation of Its Host-Guest Inclusion Potential. *J. Chem. Soc. Perkin Trans. 2* **2000**, No. 4, 865–869. https://doi.org/10.1039/a907232b.

(717) Winkler, M.; Martínková, L.; Knall, A. C.; Krahulec, S.; Klempier, N. Synthesis and Microbial Transformation of β-Amino Nitriles. *Tetrahedron* **2005**, *61* (17), 4249–4260. https://doi.org/10.1016/j.tet.2005.02.057.

(718) Zhang, B. S.; Hua, H. L.; Gao, L. Y.; Liu, C.; Qiu, Y. F.; Zhou, P. X.; Zhou, Z. Z.; Zhao, J. H.; Liang, Y. M. Palladium-Catalyzed Arene C-H Activation/Ketone C-H Functionalization Reaction: Route to Spirodihydroindenones. *Org. Chem. Front.* **2017**, *4* (7), 1376–1379. https://doi.org/10.1039/c7qo00164a.

(719) Carta, M.; Bezzu, C. G.; Vile, J.; Kariuki, B. M.; McKeown, N. B. Polymers of Intrinsic Microporosity Derived from a Carbocyclic Analogue of Tröger's Base. *Polymer* **2017**, *126*, 324–329. https://doi.org/10.1016/j.polymer.2017.03.037.

(720) Marchand, A. P.; Namboothiri, I. N. N.; Ganguly, B.; Watson, W. H.; Bodige, S. G. Generation and Trapping of an Unsymmetrical, Caged Pyramidalized Alkene. *Tetrahedron Lett.* **1999**, *40* (28), 5105–5109. https://doi.org/10.1016/S0040-4039(99)00888-6.

(721) Vary, M. W.; McBride, J. M. Using Spiropyran Fragment Superposition to Assign the EPR Spectrum of Localized Triplet Excitation on a Distorted Aromatic Nitro Group. *Cryst. Growth Des.* **2005**, *5* (6), 2036–2042. https://doi.org/10.1021/cg0503907.

(722) Yoshitake, Y.; Yamaguchi, K.; Harano, K. Formation Pathway of Novel Cycloadduct Obtained by Reaction of 3,5-Disubstituted 4-Oxo-4H-Pyrazole 1,2-Dioxide with Dimethyl Acetylenedicarboxylate. *Heterocycles* **2009**, *78* (11), 2777–2785. https://doi.org/10.3987/COM-09-11780.

(723) Li, Y.; Zhang, Q.; Wang, H.; Cheng, B.; Zhai, H. Bioinspired Total Synthesis of (±)-Chaetophenol C Enabled by a Pd-Catalyzed Cascade Cyclization. *Org. Lett.* **2017**, *19* (16), 4387–4390. https://doi.org/10.1021/acs.orglett.7b02124.

(724) Dinçer, M.; Özdemir, N.; Çukurovali, A.; Yilmaz, I.; Büyükgüngör, O. Benzoin 4-Ethylthiosemicarbazone. *Acta Cryst. C* **2006**, *62* (1), o13–o15. https://doi.org/10.1107/S0108270105037728.

(725) Ahmed, F. R. Three Crystal Structures of 1β-(p -Methoxybenzyl)-9 α,10β-Dihydroxydecahydroisoquinoline and 1β-(p -Methoxybenzyl)-9β,10α-Dihydroxydecahydroisoquinoline, C 17 H 25 NO 3 . *Acta Cryst. B* **1978**, *34* (8), 2589–2594. https://doi.org/10.1107/s056774087800864x.

(726) Davidson, A.; Murray, I. E. P.; Preston, P. N.; King, T. J. Reaction of Benzazole Derivatives with Dimethyl

Acetylenedicarboxylate. Crystal and Molecular Structures of [1,4]Thiazino[4,3-a]Benzimidazole Derivatives. *J. Chem. Soc. Perkin Trans. 1* **1979**, No. 0, 1239–1245. https://doi.org/10.1039/p19790001239.

(727) Hisano, T.; Ichikawa, M.; Matsuoka, T.; Hagiwara, H.; Muraoka, K.; Komori, T.; Harano, K.; Ida, Y.; Christensen, A. Reaction of Aromatic N-Oxides with Dipolarophiles. III. Cycloadditions of Substituted Phenyl Isocyanates to 3,5-Dimethyl and 3,5-Dibromopyridine N-Oxides and X-Ray Crystal Structures of the Isomeric Cycloadducts. *Chem. Pharm. Bull.* **1979**, *27* (10), 2261–2272. https://doi.org/10.1248/cpb.27.2261.

(728) Smith, A. E.; Kalish, R.; Smutny, E. J. The Crystal and Molecular Structure of 2 H -Thiopyran p -Bromobenzyl Ester, C 20 H 22 O 4 NS 2 Br . *Acta Cryst. B* **1972**, *28* (12), 3494–3501. https://doi.org/10.1107/s0567740872008246.

(729) Guo, X. H.; Qi, X. X.; Chang, J. B. 5-(1H-Indol-3-Yl)-3-(4-Methylphenyl)-4,5-Dihydroisoxazoline. *Acta Cryst. E* **2006**, *62* (1), o374–o375. https://doi.org/10.1107/S1600536805040092.

(730) Palani, K.; Amaladass, P.; Mohanakrishnan, A. K.; Ponnuswamy, M. N. 3-(Anthracen-9-Yl)-3H-Isobenzofuran-1-One. *Acta Cryst. E* **2006**, *62* (1), o55–o57. https://doi.org/10.1107/S1600536805039553.

(731) Bocelli, G.; Cantoni, A.; Righi, L. Crystal Structures of Two Substituted Biphenyl Derivatives: 5,5′-Di t-Butyl-2-2′-Biphenyldiol and 5,5′-Dimethyl-2,2′-Biphenyldiol. *J. Chem. Crystallogr.* **1999**, *29* (2), 157–161. https://doi.org/10.1023/A:1009509808535.

(732) Subbiahpandi, A.; Velmurugan, D.; Ravikumar, K.; Durga, R. R.; Raghunathan, R. 3-Acetyl-4-Furyl-1-Methylspiro[Pyrrolidine-2,2′-Indol] -2′(3′H)-One. *Acta Cryst. E* **2006**, *62* (5), o1964–o1965. https://doi.org/10.1107/S1600536806013353.

(733) Ware, R. W.; King, S. B. N-Phosphinoylnitroso Compounds: New Asymmetric N-O Heterodienophiles and Nitroxyl Delivery Agents [12]. *Journal of the American Chemical Society*. American Chemical Society July 21, 1999, pp 6769–6770. https://doi.org/10.1021/ja9908016.

(734) Singh, R. P.; Kirchmeier, R. L.; Shreeve, J. M. TBAF-Catalyzed Direct Nucleophilic Trifluoromethylation of α-Keto Amides with Trimethyl(Trifluoromethyl)Silane. *J. Org. Chem.* **1999**, *64* (7), 2579–2581. https://doi.org/10.1021/jo982297d.

(735) Kendhale, A.; Gonnade, R.; Rajamohanan, P. R.; Sanjayan, G. J. Isotactic N-Alkyl Acrylamide Oligomers Assume Self-Assembled Sheet Structure: First Unequivocal Evidence from Crystal Structures. *Chem. Commun.* **2006**, No. 26, 2756–2758. https://doi.org/10.1039/b601317a.

(736) Yang, L.; Xie, P.; Li, E.; Li, X.; Huang, Y.; Chen, R. Phosphine-Catalyzed Domino Reaction: An Efficient Method for the Synthesis of Highly Functionalized Spirooxazolines. *Org. Biomol. Chem.* **2012**, *10* (37), 7628–7634. https://doi.org/10.1039/c2ob26338f.

(737) Xu, Y.; Rudler, H.; Denise, B.; Parlier, A.; Chaquin, P.; Herson, P. Lactones from Lactones: Regio and Diastereoselective Double Dinucleophilic Additions of Bis(OTMS) Ketene Acetals to Pyridines. *Tetrahedron Lett.* **2006**, *47* (27), 4541–4544. https://doi.org/10.1016/j.tetlet.2006.05.004.

(738) Mamedov, V. A.; Gubaidullin, A. T.; Litvinov, I. A.; Tsuboi, S. An Unexpected Reaction of 3-Phenyl-3-Chloro-2-Oxopropanoic Acid Derivatives with Sodium Azide. A Novel Synthesis of Highly Functionalized Oxazolines. *Heterocycles* **2000**, *52* (3), 1385–1391. https://doi.org/10.3987/com-99-s128.

(739) Crespin, L. N. S.; Greb, A.; Blakemore, D. C.; Ley, S. V. Visible-Light-Mediated Annulation of Electron-Rich Alkenes and Nitrogen-Centered Radicals from N-Sulfonylallylamines: Construction of Chloromethylated Pyrrolidine Derivatives. *J. Org. Chem.* **2017**, *82* (24), 13093–13108. https://doi.org/10.1021/acs.joc.7b02146.

(740) Hsueh, N. C.; Chen, H. Y.; Chang, M. Y. Construction of Sulfonyl Oxabenzo[3.3.1]Bicyclic Core via Cyclocondensation of β-Ketosulfones and o-Formyl Allylbenzenes. *J. Org. Chem.* **2017**, *82* (24), 13324–13332. https://doi.org/10.1021/acs.joc.7b02425.

(741) Abbott, P. J.; Acheson, R. M.; Eisner, U.; Watkin, D. J.; Carruthers, J. R. Addition Reactions of Heterocyclic Compounds. Part LXIII. New Structures for Some 2:1 Molar Adducts from Dimethyl Acetylenedicarboxylate with Thiazoles and Benzo-Imidazoles, -Oxazoles, and -Thiazoles Formed by Novel Rearrangement. Crystal and Molecular St. *J. Chem. Soc. Perkin Trans. 1* **1976**, No. 12, 1269–1278. https://doi.org/10.1039/P19760001269.

(742) Singh, V.; Madapa, S.; Yadav, G. P.; Maulik, P. R.; Batra, S. Interesting Results of Catalytic Hydrogenation of 3-(2-Nitrophenyl) Isoxazoles and 3- (Nitrophenyl)-4,5-Dihydroisoxazoles. *Synthesis* **2006**, *2006* (12), 1995–2004. https://doi.org/10.1055/s-2006-942388.

(743) Akkurt, M.; Kennedy, A. R.; Mohamed, S. K.; Younes, S. H. H.; Miller, G. J. 3-Amino-1-Phenyl-1H-Benzo[f]Chromene-2-Carbonitrile. *Acta Cryst. E* **2013**, *69* (3), o401–o401. https://doi.org/10.1107/S1600536813004376.

(744) Aboussafy, C. L.; Clive, D. L. J. A Dieckmann Cyclization Route to Piperazine-2,5-Diones. *J. Org. Chem.* **2012**, *77* (11), 5125–5131. https://doi.org/10.1021/jo3007144.

(745) Liu, Y.; Zhu, J.; Qian, J.; Xu, Z. Gold-Catalyzed Simultaneous Formation of C-C, C=O, and C-F Bonds in the Presence of Selectfluor: A Synthesis of Fluoroindenes from Allene Esters. *J. Org. Chem.* **2012**, *77* (12), 5411–5417. https://doi.org/10.1021/jo3006528.

(746) Krabbendam, H.; Spek, A. L. 7,11-Bis(5-Methyl-2-Furyl)Spiro[5.5]Undecane-1,5,9-Trione. *Acta Cryst. B* **1979**, *35* (8), 1913–1915. https://doi.org/10.1107/s0567740879008098.

(747) Ishida, S.; Iwamoto, T.; Kabuto, C.; Kira, M. Unexpected Reactions of an Isolable Dialkylsilylene with Haloalkanes. *Chem. Lett.* **2001**, No. 11, 1102–1103. https://doi.org/10.1246/cl.2001.1102.

(748) Molchanov, A. P.; Sipkin, D. I.; Koptelov, Y. B.; Kostikov, R. R. Double Addition of Diphenylcyclopropenone to Azomethine Imines Generated from 6-Aryl-1,5-Diazabicyclo[3.1.0]Hexanes. *Eur. J. Org. Chem.* **2002**, No. 3, 453–456. https://doi.org/10.1002/1099-0690(20022)2002:3<453::AID-EJOC453>3.0.CO;2-R.

(749) Hansen, L. K.; Bayer, A.; Gautun, O. R. Benzyl (1 R *,3 S *)-3,6-Dihydro-3-Methyl-1λ 4 ,2-Thiazine-2-Carboxylate 1-Oxide . *Acta Cryst. E* **2002**, *58* (2), o165–o166. https://doi.org/10.1107/s1600536802000880.

(750) Liang, X.; Huang, X.; Xiong, M.; Shen, K.; Pan, Y. Copper(i)-Catalyzed N-H Olefination of Sulfonamides for: N - Sulfonyl Enaminone Synthesis. *Chem. Commun.* **2018**, *54* (60), 8403–8406. https://doi.org/10.1039/c8cc04385j.

(751) Kaaz, M.; Bender, J.; Förster, D.; Frey, W.; Nieger, M.; Gudat, D. Phosphines with N-Heterocyclic Boranyl Substituents. *Dalt. Trans.* **2014**, *43* (2), 680–689. https://doi.org/10.1039/c3dt52441h.

(752) Garner, S. A.; Krische, M. J. Rhodium-Catalyzed Reductive Mannich Coupling of Vinyl Ketones to N-Sulfonylimines Mediated by Hydrogen. *J. Org. Chem.* **2007**, *72* (15), 5843–5846. https://doi.org/10.1021/jo070779w.

(753) Kou, Y. Da; Zhao, Z. N.; Yang, X.; Kalita, S. J.; Chen, X. J.; Xie, Z. Z.; Zhao, Y.; Huang, Y. Y. Stereospecific Synthesis of Fluorinated Pyrazolidinones and Isoxazolidines via a Catalyst-Free 1,3-Dipolar Cycloaddition of β-Fluoroalkylated α,β-Unsaturated 2-Pyridylsulfones. *Asian J. Org. Chem.* **2018**, *7* (9), 1830–1834. https://doi.org/10.1002/ajoc.201800435.

(754) Lebrasseur, N.; Gagnepain, J.; Ozanne-Beaudenon, A.; Léger, J. M.; Quideau, S. Efficient Access to Orthoquinols and Their [4 + 2] Cyclodimers via SIBX-Mediated Hydroxylative Phenol Dearomatization. *J. Org. Chem.* **2007**, *72* (16), 6280–6283. https://doi.org/10.1021/jo0708893.

(755) Cheng, L. Q.; Cheng, Y. Reaction of β-Lactam Carbenes with Alkyl Isonitriles for a Ready Approach to 4-Cyano and 4-Carbamoyl Substituted β-Lactams. *Tetrahedron* **2007**, *63* (38), 9359–9364. https://doi.org/10.1016/j.tet.2007.06.111.

(756) Pleschke, A.; Geier, J.; Keller, M.; Wörth, J.; Knothe, L.; Prinzbach, H. In Pursuit of Cis,Cis,Cis-Cyclonona-2,5,8-Triene-1,4,7-Trione - An Adventure in Medium-Sized Ring Chemistry. *Eur. J. Org. Chem.* **2007**, *2007* (29), 4867–4880. https://doi.org/10.1002/ejoc.200700397.

(757) Tian, G. Q.; Shi, M. Gold(I)-Catalyzed Three-Component Additions of 2-(Arylmethylene) Cyclopropylcarbinols, Terminal Arynes, and Alcohols: An Efficient Access to 3-Oxabicyclo[3.1.0]Hexanes. *Org. Lett.* **2007**, *9* (23), 4917–4920. https://doi.org/10.1021/ol702341a.

(758) Clayden, J.; Purewal, S.; Helliwell, M.; Mantell, S. J. Dearomatizing Disrotatory Electrocyclic Ring Closure of Lithiated N-Benzoyloxazolidines. *Angew. Chem. Int. Ed.* **2002**, *41* (6), 1049–1051. https://doi.org/10.1002/1521-3773(20020315)41:6<1049::AID-ANIE1049>3.0.CO;2-7.

(759) Palacios, F.; Herrán, E.; Rubiales, G.; Ezpeleta, J. M. Cycloaddition Reaction of 2-Azadienes Derived from β-Amino Acids with Electron-Rich and Electron-Deficient Alkenes and Carbonyl Compounds. Synthesis of Pyridine and 1,3-Oxazine Derivatives. *J. Org. Chem.* **2002**, *67* (7), 2131–2135. https://doi.org/10.1021/jo016273+.

(760) Bojase, G.; Payne, A. D.; Willis, A. C.; Sherburn, M. S. One-Step Synthesis and Exploratory Chemistry of [5]Dendralene. *Angew. Chem. Int. Ed.* **2008**, *47* (5), 910–912. https://doi.org/10.1002/anie.200704470.

(761) Thornton, A. R.; Blakey, S. B. Catalytic Metallonitrene/Alkyne Metathesis: A Powerful Cascade Process for the Synthesis of Nitrogen-Containing Molecules. *J. Am. Chem. Soc.* **2008**, *130* (15), 5020–5021. https://doi.org/10.1021/ja7111788.

(762) Ray, J. K.; Singha, R.; Ray, D.; Ray, P.; Rao, D. Y.; Anoop, A. Palladium-Catalyzed Expedient Heck Annulations in 1-Bromo-1,5-Dien-3-Ols: Exceptional Formation of Fused Bicycles. *Tetrahedron Lett.* **2019**, *60* (13), 931–935. https://doi.org/10.1016/j.tetlet.2019.02.043.

(763) Garduno-Alva, A.; Xu, Y.; Gualo-Soberanes, N.; Lopez-Cortes, J.; Rudler, H.; Parlier, A.; Ortega-Alfaro, M. C.;

Alvarez-Toledano, C.; Toscano, R. A. Synthesis of New Polycyclic γ- and δ-Lactones upon Activation of, and Nucleophilic Additions to, Diazines: Influence of the Activating Agents. *Eur. J. Org. Chem.* **2008**, *2008* (21), 3714–3723. https://doi.org/10.1002/ejoc.200800338.

(764) Serguchev, Y. A.; Lourie, L. F.; Polishchuk, G. V.; Chernega, A. N. Fluorocyclization of Norbornenecarboxylic Acids with F-TEDA-BF4. *Mendeleev Commun.* **2002**, *12* (3), 115–117. https://doi.org/10.1070/MC2002v012n03ABEH001564.

(765) Dong, K. Y.; Qin, H. T.; Liu, F.; Zhu, C. Oxime-Mediated Oxychlorination and Oxybromination of Unactivated Olefins. *Eur. J. Org. Chem.* **2015**, *2015* (7), 1419–1422. https://doi.org/10.1002/ejoc.201403538.

(766) Bentabed-Ababsa, G.; Derdour, A.; Roisnel, T.; Sáez, J. A.; Pérez, P.; Chamorro, E.; Domingo, L. R.; Mongin, F. A Combined Experimental and Theoretical Study of the Polar [3 + 2] Cyeloaddition of Electrophilically Activated Carbonyl Ylides with Aldehydes and Imines. *J. Org. Chem.* **2009**, *74* (5), 2120–2133. https://doi.org/10.1021/jo8027104.

(767) Pérez-Ruiz, R.; Hinze, O.; Neudörfl, J. M.; Blunk, D.; Görner, H.; Griesbeck, A. G. Photochemistry of Allyloxybenzophenones: A Pseudo-Paternò-Büchi Rearrangement Accompanied by Hydrogen Transfer Induced 1,5-Cyclization. *Photochem. Photobiol. Sci.* **2008**, *7* (7), 782–788. https://doi.org/10.1039/b807889k.

(768) Yamashita, M.; Okuyama, K.; Kawajiri, T.; Takada, A.; Inagaki, Y.; Nakano, H.; Tomiyama, M.; Ohnaka, A.; Terayama, I.; Kawasaki, I.; Ohta, S. A Novel Tandem Reaction of 3-Substituted Coumarins with Two Equivalents of Dimethylsulfoxonium Ylide to 2-Substituted Cyclopenta[b]Benzofuran-3-Ol Derivatives. *Tetrahedron* **2002**, *58* (8), 1497–1505. https://doi.org/10.1016/S0040-4020(02)00014-5.

(769) McCallum, P. A.; Irving, H. M. N. H.; Hutton, A. T.; Nassimbeni, L. R. The Structures of Two Condensation Products of Dithizone. 2-Methyl-3-Phenyl-5-Phenylazo-1,3,4-Thiadiazoline and 5,6-Dihydro-4-Phenyl-2-Phenylazo-4H-1,3,4-Thiadiazine. *Acta Cryst. B* **1980**, *36* (7), 1626–1630. https://doi.org/10.1107/s0567740880006723.

(770) He, L. 3-(4-Nitro-Phen-Yl)-N-Phenyl-Oxirane-2-Carboxamide. *Acta Cryst. E* **2009**, *65* (8), o2052–o2052. https://doi.org/10.1107/S1600536809029699.

(771) Ferraz, H. M. C.; Longo, L. S.; Zukerman-Schpector, J. Thallium Trinitrate Mediated Oxidation of 3-Alkenols: Ring Contraction vs Cyclization. *J. Org. Chem.* **2002**, *67* (10), 3518–3521. https://doi.org/10.1021/jo011178m.

(772) Wang, C.; Zhang, L.; Chen, C.; Han, J.; Yao, Y.; Zhao, Y. Oxalyl Amide Assisted Palladium-Catalyzed Synthesis of Pyrrolidones via Carbonylation of γ-C(Sp3)-H Bonds of Aliphatic Amine Substrates. *Chem. Sci.* **2015**, *6* (8), 4610–4614. https://doi.org/10.1039/c5sc00519a.

(773) Mohamed, S. K.; Horton, P. N.; Akkurt, M.; Younes, S. H. H.; Albayati, M. R. Crystal Structure of 2-Amino-4-Phenyl-4H-Benzo[h]Chromene-3-Carbonitrile. *Acta Cryst. E* **2015**, *71* (7), o516–o517. https://doi.org/10.1107/S2056989015011536.

(774) Coulthard, G.; Unsworth, W. P.; Taylor, R. J. K. Propylphosphonic Anhydride (T3P) Mediated Synthesis of β-Lactams from Imines and Aryl-Substituted Acetic Acids. *Tetrahedron Lett.* **2015**, *56* (23), 3113–3116. https://doi.org/10.1016/j.tetlet.2015.01.009.

(775) Quintana, L.; Peña, D.; Pérez, D.; Guitián, E. Generation and Reactivity of 1,2-Cyclohexadiene under Mild Reaction Conditions. *Eur. J. Org. Chem.* **2009**, *2009* (32), 5519–5524. https://doi.org/10.1002/ejoc.200900631.

(776) Rahman, A. N. M. M.; Bishop, R.; Craig, D. C.; Scudder, M. L. Crystal Engineering Involving C-H···N Weak Hydrogen Bonds: Penannular Enclosure of Organic Guests by a Diquinoline Host. *Eur. J. Org. Chem.* **2003**, No. 1, 72–81. https://doi.org/10.1002/1099-0690(200301)2003:1<72::AID-EJOC72>3.0.CO;2-Z.

(777) Banwell, M. G.; Jones, M. T.; Loong, D. T. J.; Lupton, D. W.; Pinkerton, D. M.; Ray, J. K.; Willis, A. C. A Pd[0]-Catalyzed Ullmann Cross-Coupling/Reductive Cyclization Approach to C-3 Mono-Alkylated Oxindoles and Related Compounds. *Tetrahedron* **2010**, *66* (47), 9252–9262. https://doi.org/10.1016/j.tet.2010.09.042.

(778) Skarżyński, T.; Brozozowski, A. M.; Derewenda, Z.; Mlostoń, G. C-4,t-5-Bis(p-Methoxyphenyl)-t-3-Methyl-r-2-Phenyl-1,3-Oxazolidine, C24H25NO3. *Acta Cryst. C* **1983**, *39* (8), 1051–1053. https://doi.org/10.1107/s0108270183007337.

(779) Wilson, S. R.; Phillips, L. R.; Pelister, Y.; Huffman, J. C. Cyclobutene Derivatives as Isoprene Equivalents in Terpene Synthesis. 3. Bicyclo[2.2.0]Hexanes. *J. Am. Chem. Soc.* **1979**, *101* (24), 7373–7379. https://doi.org/10.1021/ja00518a040.

(780) Zimmerman, H. E.; Hoffacker, K. D. Novel Radical Cation Reactions of Bichromophoric Systems. Transannular Aryl Migrations; Mechanistic and Exploratory Organic Photochemistry. *J. Org. Chem.* **1996**, *61* (19), 6526–6534. https://doi.org/10.1021/jo960467l.

(781) Weng, S. S.; Zhang, J. W. N-Oxyl-Radical-Catalyzed Intermolecular Aminooxygenation of Styrenes and Inter/Intramolecular Aminoalkoxylation of Homoallylic Alcohols. *ChemCatChem* **2016**, *8* (24), 3720–3724. https://doi.org/10.1002/cctc.201601183.

(782) García, M. D.; Caamaño, O.; Fernández, F.; López, C.; De Clercq, E. Synthesis of Purinyl Homo-Carbonucleoside Derivatives of 2-Benzylcyclopenta[c]Pyrazol. *Synthesis* **2005**, *2005* (6), 925–932. https://doi.org/10.1055/s-2005-861831.

(783) Li, S.; Cao, W. J.; Ma, J. A. Thieme Chemistry Journals Awardees - Where Are They Now?Stereoselective Cycloaddition of 2,2,2-Trifluorodiazoethane with α-Methylene-β-Lactams: Facile Synthesis of Trifluoromethyl-Substituted Spirocyclic β-Lactams. *Synlett* **2017**, *28* (6), 673–678. https://doi.org/10.1055/s-0036-1588363.

(784) Ivanova, O. A.; Budynina, E. M.; Chagarovskiy, A. O.; Trushkov, I. V.; Melnikov, M. Y. (3 + 3)-Cyclodimerization of Donor-Acceptor Cyclopropanes. Three Routes To Six-Membered Rings. *J. Org. Chem.* **2011**, *76* (21), 8852–8868. https://doi.org/10.1021/jo201612w.

(785) Ghalib, R. M.; Hashim, R.; Alshahateet, S. F.; Mehdi, S. H.; Sulaiman, O.; Chan, K. L.; Murugaiyah, V.; Jawad, A. Synthesis, Antimicrobial and Cholinesterase Enzymes Inhibitory Activities of Indeno Imidazoles and X-Ray Crystal Structure of 3a,8a-Dihydroxy-1,3- Diphenyl-1,3,3a,8a-Tetrahydro-Indeno [1,2-d]Imidazole-2,8-Dione. *J. Chem. Crystallogr.* **2012**, *42* (8), 783–789. https://doi.org/10.1007/s10870-012-0288-7.

(786) Atarashi, S.; Choi, J. K.; Ha, D. C.; Hart, D. J.; Kuzmich, D.; Lee, C. S.; Ramesh, S.; Wu, S. C. Free Radical Cyclizations in Alkaloid Total Synthesis: (±)-21- Oxogelsemine and (±)-Gelsemine. *J. Am. Chem. Soc.* **1997**, *119* (27), 6226–6241. https://doi.org/10.1021/ja970089h.

(787) Hou, J. Y.; Wang, D. Z.; Li, F.; Yan, Z. Y.; Liang, Y. M.; Liu, Y. Q. Synthesis of 5-Azaspiro[2.4]Heptan and Penta-Substituted Pyrrole Derivatives via Pd-Catalyzed Intramolecular Cyclization Reaction of Alkynyl Carboxamides. *Synth. Commun.* **2012**, *42* (7), 1070–1084. https://doi.org/10.1080/00397911.2010.535944.

(788) Mao, R.; Yuan, Z.; Li, Y.; Wu, J. N-Radical-Initiated Cyclization through Insertion of Sulfur Dioxide under Photoinduced Catalyst-Free Conditions. *Chem. Eur. J.* **2017**, *23* (34), 8176–8179. https://doi.org/10.1002/chem.201702040.

(789) Dindulkar, S. D.; Puranik, V. G.; Jeong, Y. T. Supported Copper Triflate as an Efficient Catalytic System for the Synthesis of Highly Functionalized 2-Naphthol Mannich Bases under Solvent Free Condition. *Tetrahedron Lett.* **2012**, *53* (33), 4376–4380. https://doi.org/10.1016/j.tetlet.2012.06.022.

(790) Sugimoto, A.; Hayashi, C.; Omoto, Y.; Mizuno, K. Synthesis of Tricyclic Lactones Containing 2-Azabicyclo[3.3.1]Nonane Skeleton via Tandem Intramolecular Photocyclization of 2-(1-Naphthyl)Ethyl ω-Anilinoalkanoate. *Tetrahedron Lett.* **1997**, *38* (18), 3239–3242. https://doi.org/10.1016/S0040-4039(97)00573-X.

(791) Aust, N. C.; Fröhlich, R.; Hecht, J.; Würthwein, E. U. Unsaturated Hetero Chains, VI: ω-Alkoxy- and ω-Amino-Substituted Oligonitriles - Synthesis, Structures, and Reactivity. *Liebigs Ann.* **1997**, *1997* (7), 1593–1602. https://doi.org/10.1002/jlac.199719970742.

(792) Wu, Y.; Yuan, C.; Wang, C.; Mao, B.; Jia, H.; Gao, X.; Liao, J.; Jiang, F.; Zhou, L.; Wang, Q.; Guo, H. Palladium-Catalyzed [5 + 2] Cycloaddition of Vinyloxiranes with Sulfamate-Derived Cyclic Imines to Construct 1,3-Oxazepine Heterocycles. *Org. Lett.* **2017**, *19* (23), 6268–6271. https://doi.org/10.1021/acs.orglett.7b02704.

(793) Fritz, S. P.; Matlock, J. V.; McGarrigle, E. M.; Aggarwal, V. K. Efficient Synthesis of Cyclopropane-Fused Heterocycles with Bromoethylsulfonium Salt. *Chem. Eur. J.* **2013**, *19* (33), 10827–10831. https://doi.org/10.1002/chem.201302081.

(794) Odabaşoğlu, M.; Büyükgüngör, O. 3-(4-Acetylanilino)Isobenzofuran-1(3H)-One. *Acta Cryst. E* **2006**, *62* (9), o4145–o4147. https://doi.org/10.1107/S1600536806032570.

(795) Kostyanovsky, R. G.; Lyssenko, K. A.; Kravchenko, A. N.; Lebedev, O. V.; Kadorkina, G. K.; Kostyanovsky, V. R. Crystal Properties of N-Alkyl-Substituted Glycolurils as the Precursors of Chiral Drugs. *Mendeleev Commun.* **2001**, *11* (4), 134–136. https://doi.org/10.1070/MC2001v011n04ABEH001469.

(796) Miao, M.; Luo, Y.; Xu, H.; Jin, M.; Chen, Z.; Xu, J.; Ren, H. Strain-Promoted Nitration of 3-Cyclopropylideneprop-2-En-1-Ones and the Application for the Synthesis of Pyrroles. *J. Org. Chem.* **2017**, *82* (23), 12224–12237. https://doi.org/10.1021/acs.joc.7b02087.

(797) Zheng, G.; Sun, J.; Liu, Y.; Yang, S.; Li, Y.; Sun, H.; Zhang, Q. Copper-Catalyzed Azidative Multifunctionalization of Alkynes. *J. Org. Chem.* **2017**, *82* (23), 12813–12820. https://doi.org/10.1021/acs.joc.7b02148.

(798) Riedel, S.; Maichle-Mössmer, C.; Maier, M. E. Intramolecular Diels-Alder Reactions of Tethered Enoate Substituted Furans Induced by Dialkylaluminum Chloride. *J. Org. Chem.* **2017**, *82* (23), 12798–12805.

https://doi.org/10.1021/acs.joc.7b02117.

(799) Barluenga, J.; Tomás, M.; Bieger, K.; García-Granda, S.; Santiago-García, R. Reactivity of Dihydrodiazaphosphinines towards Unsaturated Substrates: Addition, [5 + 2] Cycloaddition and Rearrangement Processes. *J. Organomet. Chem.* **1997**, *529* (1–2), 233–241. https://doi.org/10.1016/S0022-328X(96)06618-1.

(800) Huang, H. M.; Procter, D. J. Radical Heterocyclization and Heterocyclization Cascades Triggered by Electron Transfer to Amide-Type Carbonyl Compounds. *Angew. Chem. Int. Ed.* **2017**, *56* (45), 14262–14266. https://doi.org/10.1002/anie.201708354.

(801) Egli, D. H.; Linden, A.; Heimgartner, H. Reactions of α,β-Unsaturated Thioamides with Diazo Compounds. *Helv. Chim. Acta* **2006**, *89* (11), 2815–2824. https://doi.org/10.1002/hlca.200690252.

(802) Okuma, K.; Yasuda, T.; Takeshita, I.; Shioji, K.; Yokomori, Y. Novel Formation of Indoles and 3,1-Benzoxazines from o-Alkenylanilides and Dimethyl(Methylthio)Sulfonium Trifluoromethanesulfonate. *Tetrahedron* **2007**, *63* (34), 8250–8254. https://doi.org/10.1016/j.tet.2007.05.111.

(803) Schelwies, M.; Dempwolff, A. L.; Rominger, F.; Helmchen, G. Gold-Catalyzed Intermolecular Addition of Carbonyl Compounds to 1,6-Enynes. *Angew. Chem. Int. Ed.* **2007**, *46* (29), 5598–5601. https://doi.org/10.1002/anie.200701378.

(804) Coppola, A.; Sánchez-Alonso, P.; Sucunza, D.; Burgos, C.; Alajarín, R.; Alvarez-Builla, J.; Mosquera, M. E. G.; Vaquero, J. J. Remote Aryl Cyanation via Isocyanide-Cyanide Rearrangement on Tosylmethyl Isocyanide Derivatives. *Org. Lett.* **2013**, *15* (13), 3388–3391. https://doi.org/10.1021/ol401433x.

(805) Fraser, R. R.; Faibish, N. C.; Kong, F.; Bednarski, K. Stereodependence of the Effect of α Substituants on the Face Reactivity of Conformationally Rigid Ketones toward Metal Hydrides. Is There an Antiperiplanar (Cieplak) Effect? *J. Org. Chem.* **1997**, *62* (18), 6164–6176. https://doi.org/10.1021/jo970428p.

(806) Ye, L. W.; Sun, X. L.; Li, C. Y.; Tang, Y. Tetrahydrothiophene-Catalyzed Synthesis of Benzo[n.1.0] Bicycloalkanes. *J. Org. Chem.* **2007**, *72* (4), 1335–1340. https://doi.org/10.1021/jo062209m.

(807) Fu, T. Y.; Scheffer, J. R.; Trotter, J.; Yang, J. Structure and Photochemistry of Four Adamantylacetophenones. *Acta Cryst. C* **1998**, *54* (4), 491–496. https://doi.org/10.1107/S0108270197015862.

(808) Zheng, C.; Wang, L.; Li, J.; Wang, L.; Wang, D. Z. Ortho-Dearomatization of Phenols Creating All-Carbon Spiro-Bicycles. *Org. Lett.* **2013**, *15* (16), 4046–4049. https://doi.org/10.1021/ol401863k.

(809) Rajca, A.; Takahashi, M.; Pink, M.; Spagnol, G.; Rajca, S. Conformationally Constrained, Stable, Triplet Ground State (S = 1) Nitroxide Diradicals. Antiferromagnetic Chains of S = 1 Diradicals. *J. Am. Chem. Soc.* **2007**, *129* (33), 10159–10170. https://doi.org/10.1021/ja0712017.

(810) Vroemans, R.; Bamba, F.; Winters, J.; Thomas, J.; Jacobs, J.; Van Meervelt, L.; John, J.; Dehaen, W. Sequential Ugi Reaction/Base-Induced Ring Closing/IAAC Protocol toward Triazolobenzodiazepine-Fused Diketopiperazines and Hydantoins. *Beilstein J. Org. Chem.* **2018**, *14* (1), 626–633. https://doi.org/10.3762/bjoc.14.49.

(811) Ghorai, J.; Chaitanya, M.; Anbarasan, P. Cp*Co(Iii)-Catalysed Selective Alkylation of C-H Bonds of Arenes and Heteroarenes with α-Diazocarbonyl Compounds. *Org. Biomol. Chem.* **2018**, *16* (40), 7346–7350. https://doi.org/10.1039/c8ob02111b.

(812) Valente, E. J.; Fuller, J. F.; Ball, J. D. Pseudoacids. I. 4- and 5-Oxoacids. *Acta Cryst. B* **1998**, *54* (2), 162–173. https://doi.org/10.1107/S010876819701149X.

(813) Prieto, O.; Lam, H. W. Cobalt-Catalyzed Reductive Mannich Reactions of 4-Acryloylmorpholine with N-Tosyl Aldimines. *Org. Biomol. Chem.* **2008**, *6* (1), 55–57. https://doi.org/10.1039/b715839d.

(814) Liu, X. Y.; Cheng, B. Q.; Guo, Y. C.; Chu, X. Q.; Li, Y. X.; Loh, T. P.; Shen, Z. L. Bismuth-Mediated Diastereoselective Allylation Reaction of Carbonyl Compounds with Cyclic Allylic Halides or Cinnamyl Halide. *Adv. Synth. Catal.* **2019**, *361* (3), 542–549. https://doi.org/10.1002/adsc.201801297.

(815) Yang, W. L.; Zuo, Z. Q.; Li, M. Ethyl 3′-Cyano-1′-Methyl-2-Oxo-4′-Phenylspiro- [Acenaphthene-1,2′-Pyrrolidine]-3′-Carboxylate. *Acta Cryst. E* **2008**, *64* (2), 543–548. https://doi.org/10.1107/S1600536807067530.

(816) Li, X.; Wang, S.; Li, S.; Li, K.; Mo, X.; Liu, L.; Chang, W.; Li, J. Temperature-Controlled Divergent Hydroamination Cyclization [2+2]-Cycloaddition Cascade Reactions of Homopropargylic Amines with 2-Butynedioates: Direct Access to Pyrrolo- b-Cyclobutene and Dihydro-1 H-Azepines. *J. Org. Chem.* **2019**, *84* (3), 1288–1298. https://doi.org/10.1021/acs.joc.8b02730.

(817) Belyy, A. Y.; Levina, A. A.; Platonov, D. N.; Salikov, R. F.; Medvedev, M. G.; Tomilov, Y. V. Synthesis of Diazanorcaradienes and 1,2-Diazepines via the Tandem [4+2]-Cycloaddition/Retro-[4+2]-Cycloaddition

Reaction between Methoxycarbonylcyclopropenes and Dimethoxycarbonyltetrazine. *Eur. J. Org. Chem.* **2019**, *2019* (26), 4133–4138. https://doi.org/10.1002/ejoc.201801861.

(818) Wu, S. Y.; Chen, W. L.; Ma, X. P.; Liang, C.; Su, G. F.; Mo, D. L. Copper-Catalyzed [3+2] Cycloaddition and Interrupted Fischer Indolization to Prepare Polycyclic Furo[2,3-b]Indolines from N-Aryl Isatin Nitrones and Methylenecyclopropanes. *Adv. Synth. Catal.* **2019**, *361* (5), 965–970. https://doi.org/10.1002/adsc.201801327.

(819) Schmidt, O.; Fuchs, A.; Gudat, D.; Nieger, M.; Hoffbauer, W.; Niecke, E.; Schoeller, W. W. Valence Isomerization in the Solid State: From 1,3-Diphosphacyclobutane-2,4-Diyl to 1,2-Dihydro-1,2-Diphosphete. *Angew. Chem. Int. Ed.* **1998**, *37* (7), 949–952. https://doi.org/10.1002/(SICI)1521-3773(19980420)37:7<949::AID-ANIE949>3.0.CO;2-H.

(820) Merz, A.; Gromann, L.; Karl, A.; Parkanyi, L.; Schneider, O. Conformers and Rotamers of (±)-Trans-2,3-Bis(2-Naphthyl)-15-Crown-5 and -18-Crown-6 and Their Alkali Metal Complexes. *Eur. J. Org. Chem.* **1998**, No. 2, 403–408. https://doi.org/10.1002/(sici)1099-0690(199802)1998:2<403::aid-ejoc403>3.0.co;2-q.

(821) Mishima, N.; Ogawa, T.; Tanabe, G.; Muraoka, O.; Wasada, H.; Hatae, N.; Yoshimatsu, M. Synthesis of Thiazinoimidazoles by Lewis Acid–Catalyzed [3+3] Cycloaddition Reactions of Propargyl Alcohols with 2-Mercaptoimidazoles. *Eur. J. Org. Chem.* **2019**, *2019* (20), 3117–3121. https://doi.org/10.1002/ejoc.201900367.

(822) Maguire, C. J.; Carlson, G. J.; Ford, J. W.; Strecker, T. E.; Hamel, E.; Trawick, M. L.; Pinney, K. G. Synthesis and Biological Evaluation of Structurally Diverse α-Conformationally Restricted Chalcones and Related Analogues. *MedChemComm* **2019**, *10* (8), 1445–1456. https://doi.org/10.1039/c9md00127a.

(823) Liu, K.; Wang, G.; Cheng, S. J.; Jiang, W. F.; He, C.; Ye, Z. S. Phosphine-Catalyzed Dearomative [3+2] Annulation of 3-Nitroindoles and Allenoates. *Tetrahedron Lett.* **2019**, *60* (29), 1885–1890. https://doi.org/10.1016/j.tetlet.2019.06.016.

(824) Wang, F.; Qi, X.; Liang, Z.; Chen, P.; Liu, G. Copper-Catalyzed Intermolecular Trifluoromethylazidation of Alkenes: Convenient Access to CF3-Containing Alkyl Azides. *Angew. Chem. Int. Ed.* **2014**, *53* (7), 1881–1886. https://doi.org/10.1002/anie.201309991.

(825) Chu, X. Q.; Meng, H.; Zi, Y.; Xu, X. P.; Ji, S. J. Metal-Free Oxidative Direct C(Sp3)–H Bond Functionalization of Ethers with α,α-Diaryl Allylic Alcohols. *Chem. Commun.* **2014**, *50* (68), 9718–9721. https://doi.org/10.1039/c4cc04282d.

(826) Zhang, J.; Chan, H. S.; Xie, Z. Reaction of 13-Vertex Carboranes with Nucleophiles: Unprecedented Cage-Carbon Extrusion and Formation of Monocarba-Closo-Dodecaborate Anions. *Angew. Chem. Int. Ed.* **2008**, *47* (49), 9447–9449. https://doi.org/10.1002/anie.200804249.

(827) DeTar, M. B.; Sieloff, R. F.; VanDerveer, D.; House, H. O. Enones with Strained Double Bonds. 5. The 2-Methylbicyclo[3.3.1]Non-1-En-3-One System. *J. Org. Chem.* **1980**, *45* (18), 3545–3549. https://doi.org/10.1021/jo01306a001.

(828) Webber, P.; Krische, M. J. Concise Stereocontrolled Formal Synthesis of (±)-Quinine and Total Synthesis of (±)-7-Hydroxyquinine via Merged Morita-Baylis-Hillman-Tsuji- Trost Cyclization. *J. Org. Chem.* **2008**, *73* (23), 9379–9387. https://doi.org/10.1021/jo802165k.

(829) Dadwal, M.; Kesharwani, M. K.; Danayak, V.; Ganguly, B.; Mobin, S. M.; Muruganantham, R.; Namboothiri, I. N. N. Synthetic and Theoretical Investigations on the Construction of Oxanorbornenes by a Michael Addition and Intramolecular Diels-Alder Furan Reaction. *Eur. J. Org. Chem.* **2008**, *2008* (36), 6106–6118. https://doi.org/10.1002/ejoc.200800681.

(830) Wang, H. Y.; Xie, M. H.; Luo, S. N.; He, Y. J.; Zou, P. 4-(4-Ethoxy-Benz-Yl)-1,3-Oxazolidin-2-One. *Acta Cryst. E* **2009**, *65* (4), o805–o805. https://doi.org/10.1107/S160053680900957X.

(831) Valente, E. J.; Martin, S. B.; Sullivan, L. D. Pseudoacids. II. 2-Acylbenzoic Acid Derivatives. *Acta Cryst. B* **1998**, *54* (3), 264–276. https://doi.org/10.1107/S0108768197012020.

(832) Feng, X.; Wang, J. J.; Xun, Z.; Zhang, J. J.; Huang, Z. Bin; Shi, D. Q. Highly Selective Synthesis of Functionalized Polyhydroisoquinoline Derivatives via a Three-Component Domino Reaction. *Chem. Commun.* **2015**, *51* (8), 1528–1531. https://doi.org/10.1039/c4cc08900f.

(833) Zheng, F.; Xie, Z. Reaction of N-Heterocyclic Carbenes with 13-Vertex Closo-Carboranes: Synthesis and Structural Characterization of Zwitterionic Salts of 13-Vertex Nido-Carboranes. *Org. Chem. Front.* **2015**, *2* (1), 55–59. https://doi.org/10.1039/c4qo00287c.

(834) Allous, I.; Comesse, S.; Berkeš, D.; Alkyat, A.; Daïch, A. Toward the Improvement of the Tandem Halide Displacement/Amide Coupling Spiro-Cyclization as a New Route to γ-Lactam and Pyrroloisoquinoline Templates. *Tetrahedron Lett.* **2009**, *50* (31), 4411–4415. https://doi.org/10.1016/j.tetlet.2009.02.114.

(835) Hashmi, A. S. K.; Wagner, S.; Rominger, F. Gold Catalysis: Chemoselective Indolin Synthesis in the Presence of Acrylate Units. *Aust. J. Chem.* **2009**, *62* (7), 657–666. https://doi.org/10.1071/CH09174.

(836) Mamedov, V. A.; Murtazina, A. M.; Gubaidullin, A. T.; Hafizova, E. A.; Rizvanov, I. K. Efficient Synthesis of 2-(Pyrazol-3-Yl)Benzimidazoles from 3-Arylacylidene-3,4-Dihydroquinoxalin-2(1H)-Ones and Hydrazine Hydrate via a Novel Rearrangement. *Tetrahedron Lett.* **2009**, *50* (37), 5186–5189. https://doi.org/10.1016/j.tetlet.2009.05.116.

(837) Mohamed, S. K.; Mague, J. T.; Akkurt, M.; Abdelhamid, A. A.; Albayati, M. R. Crystal Structure of 1-{3-Acetyl-2-(4-Chlorophenyl)-6-Hydroxy-4-[(2-Hydroxypropyl)Amino]-6-Methylcyclohex-3-En-1-Yl}ethanone. *Acta Cryst. E* **2015**, *71* (5), o369–o370. https://doi.org/10.1107/S2056989015008191.

(838) Chang, M. Y.; Lu, Y. J.; Cheng, Y. C. M-CPBA-Mediated Stereoselective Synthesis of Sulfonyl Tetrahydropyrans. *Tetrahedron* **2015**, *71* (8), 1192–1201. https://doi.org/10.1016/j.tet.2015.01.016.

(839) Nguyen, V. T.; Chan, I. Y. H.; Bishop, R.; Craig, D. C.; Scudder, M. L. Crystallisation of C2-Symmetric Endo,Endo-Bicyclo[3.3.1]Nonane- 2-6-Diols: Supramolecular Synthons and Concomitant Degrees of Enantiomer Separation. *New J. Chem.* **2009**, *33* (8), 1736–1741. https://doi.org/10.1039/b900463g.

(840) Gökhan-Kelekçi, N.; Şimşek, Ö. Ö.; Ercan, A.; Yelekçi, K.; Şahin, Z. S.; Işik, Ş.; Uçar, G.; Bilgin, A. A. Synthesis and Molecular Modeling of Some Novel Hexahydroindazole Derivatives as Potent Monoamine Oxidase Inhibitors. *Bioorganic Med. Chem.* **2009**, *17* (18), 6761–6772. https://doi.org/10.1016/j.bmc.2009.07.033.

(841) Casalone, G.; Pilatc, T.; Binello, A. Five 3,4-Dihydro-2H,5H-Pyrano[3,2-c][1]-Benzopyran-5-One Derivatives. *Acta Cryst. C* **1998**, *54* (7), 1042–1047. https://doi.org/10.1107/S0108270198000249.

(842) Tiekink, E. R. T.; Macedo, A.; Wendler, E. P.; Dos Santos, A. A.; Zukerman-Schpector, J. 2-Cyclohexyl-4-Methyltetrahydropyran-4-Ol. *Acta Cryst. E* **2010**, *66* (5), o1223–o1223. https://doi.org/10.1107/S1600536810015333.

(843) Macedo, A.; Wendler, E. P.; Dos Santos, A. A.; Zukerman-Schpector, J.; Tiekink, E. R. T. Solvent-Free Catalysed Synthesis of Tetrahydropyran Odorants: The Role of SiO2·p-TSA Catalyst on the Prins-Cyclization Reaction. *J. Braz. Chem. Soc.* **2010**, *21* (8), 1563–1571. https://doi.org/10.1590/S0103-50532010000800023.

(844) Sephton, M. A.; Emerson, C. R.; Zakharov, L. N.; Blakemore, P. R. Spontaneous Symmetry Breaking during Interrupted Crystallization of an Axially Chiral Amino Acid Derivative. *Chem. Commun.* **2010**, *46* (12), 2094–2096. https://doi.org/10.1039/b922028c.

(845) Kägi, M.; Linden, A.; Mlostoń, G.; Heimgartner, H. 1,3-Oxathiole and Thiirane Derivatives from the Reactions of Azibenzil and α-Diazo Amides with Thiocarbonyl Compounds. *Helv. Chim. Acta* **1998**, *81* (2), 285–302. https://doi.org/10.1002/hlca.19980810209.

(846) Mak, T. C. W.; Patrick, B. O.; Rettig, S. J.; Scheffer, J. R.; Trotter, J.; Ukpabi, P.; Wu, B. M.; Yee, V. C. A-Naphthyl Phenyl Pinacols. *Acta Cryst. C* **1998**, *54* (8), 1148–1151. https://doi.org/10.1107/S0108270198002777.

(847) Shen, R.; Chen, L.; Huang, X. Facile Synthesis of Polycyclic Fluorene Derivatives via a Palladium-Catalyzed Coupling, Propargyl-Allenyl Isomerization and Schmittel Cyclization Sequence. *Adv. Synth. Catal.* **2009**, *351* (17), 2833–2838. https://doi.org/10.1002/adsc.200900600.

(848) Tsuge, O.; Hatta, T.; Kakura, Y.; Tashiro, H.; Maeda, H.; Kakehi, A. A New and General Route to N-Unsubstituted Azomethine Ylides from N-(Silylmethyl)Thioureas: Cycloaddition of Synthetic Equivalents of Nonstabilized Aminonitrile Ylides. *Chem. Lett.* **1997**, *26* (9), 945–946. https://doi.org/10.1246/cl.1997.945.

(849) Li, W.; Xiao, Y.; Zhang, J. Alkynyl Group as Activating Group: Base-Catalyzed Diastereoselective Domino Reactions of Electron-Deficient Enynes. *Adv. Synth. Catal.* **2009**, *351* (18), 3083–3088. https://doi.org/10.1002/adsc.200900633.

(850) Kwon, Y.; Schatz, D. J.; West, F. G. 1,4-Diketones from Cross-Conjugated Dienones: Potassium Permanganate-Interrupted Nazarov Reaction. *Angew. Chem. Int. Ed.* **2015**, *54* (34), 9940–9943. https://doi.org/10.1002/anie.201503696.

(851) Karmazin, L.; Mazzanti, M.; Bezombes, J. P.; Gateau, C.; Pécaut, J. Comparative Structural Studies of Iodide Complexes of Uranium(III) and Lanthanide(III) with Hexadentate Tetrapodal Neutral N-Donor Ligands. *Inorg. Chem.* **2004**, *43* (16), 5147–5158. https://doi.org/10.1021/ic049538m.

(852) Yang, J. Tert-Butyl 6-Oxo-2,7-Diazaspiro[4.4]Nonane-2-Carboxylate. *Acta Cryst. E* **2011**, *67* (12), o3492–o3492. https://doi.org/10.1107/S160053681105046X.

(853) Rodríguez, M.; Ochoa, M. E.; Rodríguez, C.; Santillan, R.; Barba, V.; Farfán, N. Imino Diels-Alder Reaction of Boronates. Preparation and Characterization of New 3,4-Dihydroquinoline and 1,2,3,6-Tetrahydropyridine Derivatives. *J. Organomet. Chem.* **2007**, *692* (12), 2425–2435.

https://doi.org/10.1016/j.jorganchem.2007.02.012.

(854) Yadav, V. K.; Senthil, G.; Babu, K. G.; Parvez, M.; Reid, J. L. Heteroatom Influence on the π-Facial Selectivity of Diels-Alder Cycloadditions to 1-Oxa-4-Thia-6-Vinylspiro[4.5]Dec-6-Ene, 3-Methoxy-3-Methyl-2-Vinylcyclohexene, and 3-Methoxy-2-Vinylcyclohexene. *J. Org. Chem.* **2002**, *67* (4), 1109–1117. https://doi.org/10.1021/jo0106400.

(855) Qiu, L.; Guo, X.; Zhou, J.; Liu, S.; Yang, L.; Wu, X.; Hu, W. A Stereoselective Synthesis of Fully Substituted Tetrahydrofurans through 1,3-Dipolar Cycloaddition with Cinnamaldehydes: An Easy Access to Chroman Derivatives. *RSC Adv.* **2013**, *3* (43), 20065–20070. https://doi.org/10.1039/c3ra44123g.

(856) Fan, X.; Wang, Q.; Wei, Y.; Shi, M. Catalyst-Free Geminal Aminofluorination of Ortho-Sulfonamide-Tethered Alkylidenecyclopropanes via a Wagner-Meerwein Rearrangement. *Chem. Commun.* **2018**, *54* (74), 10503–10506. https://doi.org/10.1039/C8CC05634J.

(857) Hong, F. J.; Chong, K. W.; Low, Y. Y.; Thomas, N. F.; Kam, T. S. Transformations of Ferric Chloride-Generated Stilbene Cation Radicals. the Effect of Aromatic Substitution and a Comparison with Anodic Oxidation. *Chem. Asian J.* **2015**, *10* (10), 2207–2220. https://doi.org/10.1002/asia.201500488.

(858) Vemula, N.; Pagenkopf, B. L. Synthesis of Tetrahydro-1,2-Oxazines and Pyrrolidines via Cycloadditions of Donor-Acceptor Cyclobutanes and Nitrosoarenes. *Eur. J. Org. Chem.* **2015**, *2015* (22), 4900–4906. https://doi.org/10.1002/ejoc.201500542.

(859) Mlostoń, G.; Urbaniak, K.; Linden, A.; Heimgartner, H. Unexpected Formation of Dimethylthioketene Cycloadducts in the Reaction of 1,3-Diphenylaziridine-2,2-Dicarboxylate with Cyclobutanethione Derivatives. *Helv. Chim. Acta* **2002**, *85* (9), 2644–2656. https://doi.org/10.1002/1522-2675(200209)85:9<2644::AID-HLCA2644>3.0.CO;2-V.

(860) Shi, M.; Xu, Y. M.; Zhao, G. L.; Wu, X. F. Lewis Base Effects in the Baylis-Hillman Reaction of Arenecarbaldehydes and N-Arylidene-4-Methylbenzenesulfonamides with α,β-Unsaturated Cyclic Ketones. *Eur. J. Org. Chem.* **2002**, No. 21, 3666–3679. https://doi.org/10.1002/1099-0690(200211)2002:21<3666::AID-EJOC3666>3.0.CO;2-9.

(861) Weller, T.; Seebach, D.; Davis, R. E.; Laird, B. B. 3-Hydroxy-4-nitro-cyclohexanone Aus Ketonen Und 4-Nitrobuttersäurechlorid. Eine Ringerweiternde Fünfringanellierung. *Helv. Chim. Acta* **1981**, *64* (3), 736–760. https://doi.org/10.1002/hlca.19810640314.

(862) Chang, M. Y.; Cheng, Y. C.; Lu, Y. J. Synthesis of Substituted Benzenes via Bi(OTf)3-Mediated Intramolecular Carbonyl Allylation of α-Prenyl or α-Geranyl β-Arylketosulfones. *Org. Lett.* **2015**, *17* (12), 3142–3145. https://doi.org/10.1021/acs.orglett.5b01461.

(863) Bergman, J.; Abrahamsson, S.; Dahlén, B. An Unexpected Spirocyclic Product from the Reaction of 3-(3-Methyl-2-Oxobutyl)-3-Hydroxyoxindole with Thionyl Chloride. *Tetrahedron* **1971**, *27* (24), 6143–6149. https://doi.org/10.1016/S0040-4020(01)91779-X.

(864) Umamatheswari, S.; Kabilan, S. Spectral Characterization and Crystal Structure of 5-Spiro-(3-Methyl-2,6-Diphenyltetrahydropyran-4-Yl)-4,5-Dihydro-[1,3,4]Thiadiazole. *J. Mol. Struct.* **2009**, *938* (1–3), 142–149. https://doi.org/10.1016/j.molstruc.2009.09.016.

(865) Malassene, R.; Sanchez-Bajo, L.; Toupet, L.; Hurvois, J. P.; Moinet, C. Stereoselective Approach to the Pyrroloquinoline Core of Martinelline. *Synlett* **2002**, *2002* (9), 1500–1504. https://doi.org/10.1055/s-2002-33515.

(866) Tenora, L.; Buchlovič, M.; Man, S.; Potáček, M. A New and Facile Synthesis of Methyl 3-Amino-4,5,6,7-Tetrahydrobenzo[b] Thiophene-2-Carboxylate. *Tetrahedron Lett.* **2011**, *52* (3), 401–403. https://doi.org/10.1016/j.tetlet.2010.11.076.

(867) Chandrasekhar, S.; Seenaiah, M.; Kumar, A.; Reddy, C. R.; Mamidyala, S. K.; Kumar, C. G.; Balasubramanian, S. Intramolecular Copper(I)-Catalyzed 1,3-Dipolar Cycloaddition of Azido-Alkynes: Synthesis of Triazolo-Benzoxazepine Derivatives and Their Biological Evaluation. *Tetrahedron Lett.* **2011**, *52* (7), 806–808. https://doi.org/10.1016/j.tetlet.2010.12.040.

(868) Nicolaou, K. C.; Ortiz, A.; Zhang, H.; Guella, G. Total Synthesis and Structural Revision of Vannusals A and B: Synthesis of the True Structures of Vannusals A and B. *J. Am. Chem. Soc.* **2010**, *132* (20), 7153–7176. https://doi.org/10.1021/ja100742b.

(869) Ranjith Kumar, G.; Kiran Kumar, Y.; Kant, R.; Sridhar Reddy, M. Synthesis of Benzofuranyl and Indolyl Methyl Azides by Tandem Silver-Catalyzed Cyclization and Azidation. *Org. Biomol. Chem.* **2016**, *14* (17), 4077–4088. https://doi.org/10.1039/c6ob00191b.

(870) Zimmerman, H. E.; Wang, P. An Unusual Abnormal Wolff Rearrangement. *Can. J. Chem.* **2003**, *81* (6), 517–524.

https://doi.org/10.1139/v03-039.

(871) Meng, J.; Light, M. E.; Kilburn, J. D.; Dixon, S. Samarium Diiodide Mediated Intramolecular Cyclisation of Mixed Enone-Enoate Systems: A Simple Preparation of Spirocyclic Ethers. *Tetrahedron Lett.* **2011**, *52* (8), 928–931. https://doi.org/10.1016/j.tetlet.2010.12.077.

(872) Goldring, W. P. D.; Bouazzaoui, S.; Malone, J. F. A Rearrangement-Cycloaddition Approach to Spiro-Fused Indanones. *Tetrahedron Lett.* **2011**, *52* (9), 960–963. https://doi.org/10.1016/j.tetlet.2010.12.086.

(873) Griesbeck, A. G.; Höinck, L. O.; Neudörfl, J. M. Synthesis of Spiroannulated and 3-Arylated 1,2,4-Trioxanes from Mesitylol and Methyl 4-Hydroxytiglate by Photooxygenation and Peroxyacetalization. *Beilstein J. Org. Chem.* **2010**, *6* (1), 0–0. https://doi.org/10.3762/bjoc.6.61.

(874) Ghorai, M. K.; Halder, S.; Das, R. K. Domino Imino-Aldol-Aza-Michael Reaction: One-Pot Diastereo- and Enantioselective Synthesis of Piperidines. *J. Org. Chem.* **2010**, *75* (21), 7061–7072. https://doi.org/10.1021/jo101680f.

(875) Aginagalde, M.; Bello, T.; Masdeu, C.; Vara, Y.; Arrieta, A.; Cossío, F. P. Formation of γ-Oxoacids and 1 H-Pyrrol-2(5 H)-Ones from α,β-Unsaturated Ketones and Ethyl Nitroacetate. *J. Org. Chem.* **2010**, *75* (21), 7435–7438. https://doi.org/10.1021/jo101388x.

(876) Banik, S. M.; Medley, J. W.; Jacobsen, E. N. Catalytic, Diastereoselective 1,2-Difluorination of Alkenes. *J. Am. Chem. Soc.* **2016**, *138* (15), 5000–5003. https://doi.org/10.1021/jacs.6b02391.

(877) Sun, Y.; Yu, B.; Wang, X.; Tang, S.; She, X.; Pan, X. Stereoselective Syntheses of Four Diastereomers of 3,9,12-Trihydroxycalamenene via a Benzobicyclo[3.3.1] Intermediate. *J. Org. Chem.* **2010**, *75* (12), 4224–4229. https://doi.org/10.1021/jo1008349.

(878) Aydln, A.; Bilginer, S.; Gul, H. I.; Akkurt, M.; Mete, E. Crystal Structure of 4-[5-(4-Fluorophenyl)-3-(4-Hydroxyphenyl)-4,5-Dihydropyrazol-1-Yl] Benzenesulfonamide, C21H18FN3O3S. *Zeitschrift fur Krist. New Cryst. Struct.* **2016**, *231* (1), 81–83. https://doi.org/10.1515/ncrs-2015-0034.

(879) Sakamoto, M.; Yagishita, F.; Kanehiro, M.; Kasashima, Y.; Mino, T.; Fujita, T. Exclusive Photodimerization Reactions of Chromone-2-Carboxylic Esters Depending on Reaction Media. *Org. Lett.* **2010**, *12* (20), 4435–4437. https://doi.org/10.1021/ol101734k.

(880) Zhang, H.; Hay, E. Ben; Geib, S. J.; Curran, D. P. Fates of Imine Intermediates in Radical Cyclizations of N-Sulfonylindoles and Ene-Sulfonamides. *Beilstein J. Org. Chem.* **2015**, *11* (1), 1649–1655. https://doi.org/10.3762/bjoc.11.181.

(881) Singh, M. K.; Akula, H. K.; Satishkumar, S.; Stahl, L.; Lakshman, M. K. Ruthenium-Catalyzed C-H Bond Activation Approach to Azolyl Aminals and Hemiaminal Ethers, Mechanistic Evaluations, and Isomer Interconversion. *ACS Catal.* **2016**, *6* (3), 1921–1928. https://doi.org/10.1021/acscatal.5b02603.

(882) Nicholls, T. P.; Constable, G. E.; Robertson, J. C.; Gardiner, M. G.; Bissember, A. C. Brønsted Acid Cocatalysis in Copper(I)-Photocatalyzed α-Amino C-H Bond Functionalization. *ACS Catal.* **2016**, *6* (1), 451–457. https://doi.org/10.1021/acscatal.5b02014.

(883) Tollefson, E. J.; Dawson, D. D.; Osborne, C. A.; Jarvo, E. R. Stereospecific Cross-Coupling Reactions of Aryl-Substituted Tetrahydrofurans, Tetrahydropyrans, and Lactones. *J. Am. Chem. Soc.* **2014**, *136* (42), 14951–14958. https://doi.org/10.1021/ja5076426.

(884) Mondal, S.; Chakraborty, S.; Bhowmick, S.; Das, N. Synthesis of Triptycene-Based Organosoluble, Thermally Stable, and Fluorescent Polymers: Efficient Host-Guest Complexation with Fullerene. *Macromolecules* **2013**, *46* (17), 6824–6831. https://doi.org/10.1021/ma401421k.

(885) Srinivas, V.; Sajna, K. V.; Kumara Swamy, K. C. To Stay as Allene or Go Further? Synthesis of Novel Phosphono-Heterocycles and Polycyclics via Propargyl Alcohols. *Chem. Commun.* **2011**, *47* (19), 5629–5631. https://doi.org/10.1039/c1cc10230c.

(886) Kumari, R.; Varghese, A.; George, L. Synthesis, Crystal Structure and Photophysical Properties of (E)-4-(4-(2-Hydroxybenzylideneamino)Benzyl)Oxazolidin-2-One. *J. Lumin.* **2016**, *179*, 518–526. https://doi.org/10.1016/j.jlumin.2016.07.022.

(887) Perlovich, G. L.; Volkova, T. V.; Proshin, A. N.; Sergeev, D. Y.; Bui, C. T.; Petrova, L. N.; Bachurin, S. O. Synthesis, Pharmacology, Crystal Properties, and Quantitative Solvation Studies from a Drug Transport Perspective for Three New 1,2,4-Thiadiazoles. *J. Pharm. Sci.* **2010**, *99* (9), 3754–3768. https://doi.org/10.1002/jps.22143.

(888) Christensen, K. E.; Csatayová, K.; Davies, S. G.; Lee, J. A.; Roberts, P. M.; Thompson, A. L.; Thomson, J. E. Stereochemical Assignment of Substituted 2-Aminobicyclo[3.1.0]Hexane and 2-Aminobicyclo[5.1.0]Octane Derivatives via Single Crystal x-Ray Diffraction. *J. Chem. Crystallogr.* **2011**, *41* (7), 1007–1012.

https://doi.org/10.1007/s10870-011-0034-6.

(889) Mück, F. M.; Baus, J. A.; Bertermann, R.; Tacke, R. SO2Activation by the Bis(Guanidinato)Silylene [IPrNC(NiPr2)NiPr]2Si: Formation of Neutral Six-Coordinate Silicon(IV) Complexes with a Chelating Sulfito or Dithionito Ligand. *Eur. J. Inorg. Chem.* **2016**, *2016* (20), 3240–3245. https://doi.org/10.1002/ejic.201600294.

(890) Xu, W.; Chen, M.; Sun, N.; Liu, Y. Gold-Catalyzed Cyclization of 1,6-Diynyl Dithioacetals: Via 1,7-Carbene Transfer and Aromatic C-H Functionalization. *Chem. Commun.* **2016**, *52* (73), 11000–11003. https://doi.org/10.1039/c6cc05302e.

(891) Wu, X. L.; Liu, F. M.; Zhou, Y. L. Synthesis and Structure Characterization of New [1,2,4]Triazolo[5,4-d][1,5] Benzothiazepine Derivatives through 1,3-Dipolar Cycloaddition Reaction. *J. Heterocycl. Chem.* **2011**, *48* (2), 368–372. https://doi.org/10.1002/jhet.587.

(892) Noto, N.; Koike, T.; Akita, M. Diastereoselective Synthesis of CF3- and CF2H-Substituted Spiroethers from Aryl-Fused Cycloalkenylalkanols by Photoredox Catalysis. *J. Org. Chem.* **2016**, *81* (16), 7064–7071. https://doi.org/10.1021/acs.joc.6b00953.

(893) Khan, A. T.; Musawwer Khan, M. Sequential Three-Component Reactions: Synthesis, Regioselectivity and Application of Functionalized Dihydropyridines (DHPs) for the Creation of Fused Naphthyridines. *Tetrahedron Lett.* **2011**, *52* (27), 3455–3459. https://doi.org/10.1016/j.tetlet.2011.04.098.

(894) Terrasson, V.; Planas, J. G.; Viñas, C.; Teixidor, F.; Prim, D.; Light, M. E.; Hursthouse, M. B. Closo - O - Carboranylmethylamine-Pyridine Associations: Synthesis, Characterization, and First Complexation Studies. *Organometallics* **2010**, *29* (18), 4130–4134. https://doi.org/10.1021/om1006374.

(895) Mukovoz, P. P.; Gorbunova, A. V.; Koz'minykh, V. O.; Slepukhin, P. A.; Ganebnykh, I. N.; El'tsov, O. S.; Koz'minykh, E. N. Two Directions of the Reaction of 3,4-Dihydroxy-6-Oxoalka-2,4-Dienoic Acid Esters with Anthranilic Acid Hydrazide. *Russ. J. Org. Chem.* **2016**, *52* (7), 993–999. https://doi.org/10.1134/S1070428016070125.

(896) Li, J.; Wang, M.; Zhang, Y.; Fan, Z.; Zhang, W.; Sun, F.; Ma, N. Dearomatizing Naphthol Mannich Bases toward Spiro Thiazolidinethiones Catalyzed by Recyclable Reduced Graphene Oxide with Air as Oxidant. *ACS Sustain. Chem. Eng.* **2016**, *4* (6), 3189–3195. https://doi.org/10.1021/acssuschemeng.6b00208.

(897) Schaumann, E.; Grabley, S.; Adiwidjaja, G. Cycloadditionsreaktionen von Heterokumulenen, XXI. [2: 1]- Und [3: 1]-Addukte Aus Isocyanaten Und 3-Dimethyl-amino-2H-azirinen. *Liebigs Ann. der Chemie* **1981**, *1981* (2), 264–276. https://doi.org/10.1002/jlac.198119810211.

(898) Le, T. N.; Diter, P.; Pégot, B.; Bournaud, C.; Toffano, M.; Guillot, R.; Vo-Thanh, G.; Magnier, E. S-Trifluoromethyl Sulfoximine as a Directing Group in Ortho-Lithiation Reaction toward Structural Complexity. *Org. Lett.* **2016**, *18* (19), 5102–5105. https://doi.org/10.1021/acs.orglett.6b02548.

(899) Huang, Y. W.; Frontier, A. J. Nazarov Cyclization/Internal Redox Cyclization Sequence for the Synthesis of N-Heterocyclic Bridged Ring Systems. *Org. Lett.* **2016**, *18* (19), 4896–4899. https://doi.org/10.1021/acs.orglett.6b02369.

(900) Kholodnyak, S. V.; Voskoboynik, O. Y.; Kovalenko, S. I.; Sergeieva, T. Y.; Okovytyy, S. I.; Shishkina, S. V. 5,6-Dihydro-[1,2,4]Triazolo[1,5-c]Quinazolines. Message 3. Synthesis of 2-Aryl-5-Trichloromethyl-5,6-Dihydro[1,2,4]Triazolo[1,5-c]Quinazolines and Their Reactivity towards n-Nucleophiles. *J. Org. Pharm. Chem.* **2016**, *14* (2(54)), 47–52. https://doi.org/10.24959/ophcj.16.879.

(901) Zhu, M. K.; Zhao, J. F.; Loh, T. P. Palladium-Catalyzed Oxime Assisted Intramolecular Dioxygenation of Alkenes with 1 Atm of Air as the Sole Oxidant. *J. Am. Chem. Soc.* **2010**, *132* (18), 6284–6285. https://doi.org/10.1021/ja100716x.

(902) Mohacsi, E.; O'Brien, J. P.; Todaro, L. J. Synthesis, Stereochemistry and Pharmacological Activity of Rac.-cis-tetrahydro-6-hydroxy-7-(4-methoxyphenyl)-1,4-thiazepin-5(2H)-ones. *J. Heterocycl. Chem.* **1992**, *29* (1), 193–197. https://doi.org/10.1002/jhet.5570290135.

(903) Han, D.; He, Q.; Fan, R. Aniline Dearomatization and Silver-Catalyzed [3+3] Dipolar Cycloaddition: Efficient Construction of Oxocino[4,3,2-Cd]Indoles from 2-Alkynylanilines and 2-Alkynylbenzaldoximes. *Angew. Chem. Int. Ed.* **2015**, *54* (47), 14013–14016. https://doi.org/10.1002/anie.201507277.

(904) Duke, N. E. C.; Codding, P. W. Molecular Modeling and Crystallographic Studies of 4-Amino-N-Phenylbenzamide Anticonvulsants. *J. Med. Chem.* **1992**, *35* (10), 1806–1812. https://doi.org/10.1021/jm00088a016.

(905) Hatakeyama, S.; Sugawara, K.; Takano, S. Diastereofacial Selectivity in Diels-Alder Reactions of Buta-1,3-Dienes Having Stereogenic Allylic Heteroatom Substituents at the C-2 Position. *J. Chem. Soc. Chem. Commun.* **1992**, *3* (13), 953–955. https://doi.org/10.1039/C39920000953.

(906) Kokurkina, G. V.; Dutov, M. D.; Shevelev, S. A.; Kachala, V. V.; Chizhov, A. O.; Nelyubina, Y. V. Unexpected Aspect of the Fischer Indolization of Propiophenone (5-Chloro-2-Methoxyphenyl)Hydrazone. *Mendeleev Commun.* **2011**, *21* (6), 337–338. https://doi.org/10.1016/j.mencom.2011.11.015.

(907) Hegmann, J.; Ditterich, E.; Hüttner, G.; Christl, M.; Peters, E. -M; Peters, K.; Von Schnering, H. G. Cycloadditionen von 6H-1,3,4-Oxadiazin-6-onen (4,5-Diazaα-pyronen), 11. Δ-Chlor-δ-lactone Aus Γ-Oxoketenen. *Chem. Ber.* **1992**, *125* (8), 1913–1918. https://doi.org/10.1002/cber.19921250819.

(908) St. Denis, J. D.; Liew, S. K.; Scully, C. C. G.; Yudin, A. K. Activation of Alkynylzinc Reagents by a Hemiaminal-Driven Catalytic Microenvironment. *Eur. J. Org. Chem.* **2017**, *2017* (2), 419–423. https://doi.org/10.1002/ejoc.201601554.

(909) Kirillov, N. F.; Slepukhin, P. A.; Gorbunov, A. A.; Gavrilov, A. G.; Vakhrin, M. I. Reaction of Methyl 1-Bromocyclopentanecarboxylate with Zinc and 1-Aryl-5-Phenylpenta-1,4-Dien-3-Ones. *Russ. J. Org. Chem.* **2012**, *48* (8), 1090–1093. https://doi.org/10.1134/S107042801208009X.

(910) Bredikhin, A. A.; Gubaidullin, A. T.; Bredikhina, Z. A.; Krivolapov, D. B.; Pashagin, A. V.; Litvinov, I. A. Absolute Configuration and Crystal Packing for Three Chiral Drugs Prone to Spontaneous Resolution: Guaifenesin, Methocarbamol and Mephenesin. *J. Mol. Struct.* **2009**, *920* (1–3), 377–382. https://doi.org/10.1016/j.molstruc.2008.11.037.

(911) Bellur, E.; Görls, H.; Langer, P. Synthesis of 2-Alkylidenepyrrolidines, Pyrroles, and Indoles by Condensation of Silyl Enol Ethers and 1,3-Bis-Silyl Enol Ethers with 1-Azido-2,2-Dimethoxyethane and Subsequent Reductive Cyclization. *J. Org. Chem.* **2005**, *70* (12), 4751–4761. https://doi.org/10.1021/jo047856x.

(912) Irngartinger, H.; Gries, S.; Klaus, P.; Gleiter, R. Substituenteneffekte Auf Die Struktur von Spiropentan. *Chem. Ber.* **1992**, *125* (11), 2503–2512. https://doi.org/10.1002/cber.19921251124.

(913) Zhang, J.; Tang, Y.; Wei, W.; Wu, Y.; Li, Y.; Zhang, J.; Zheng, Y.; Xu, S. Organocatalytic Cloke-Wilson Rearrangement: DABCO-Catalyzed Ring Expansion of Cyclopropyl Ketones to 2,3-Dihydrofurans. *Org. Lett.* **2017**, *19* (12), 3043–3046. https://doi.org/10.1021/acs.orglett.7b00805.

(914) Yao, T.; Ren, B.; Wang, B.; Zhao, Y. Highly Selective Synthesis of Dihydrobenzo[d]Isoxazoles and Dihydrobenzo[d]Oxazoles from Oximes and Arynes via in Situ Generation of Nitrones. *Org. Lett.* **2017**, *19* (12), 3135–3138. https://doi.org/10.1021/acs.orglett.7b01260.

(915) Kesternich, V.; Nelson-González, R.; Pérez-Fehrmann, M.; Cárdenas, A.; Brito, I. 1-(2,4-Difluorophenyl)-2-(1H-1,2,4-Triazol-1-Yl)Ethanol. *Acta Cryst. E* **2012**, *68* (6), o1727–o1727. https://doi.org/10.1107/S1600536812020661.

(916) Yang, S. H.; Zhai, Z. W. Dimethyl[(E)-(2-Nitromethylidene-1,3-Dithiolan-4-Yl)Methyl]Amine. *Acta Cryst. E* **2012**, *68* (6), o1749–o1749. https://doi.org/10.1107/S1600536812021307.

(917) Ogawa, N.; Yamaoka, Y.; Yamada, K. I.; Takasu, K. Synthesis of π-Extended Fluoranthenes via a KHMDS-Promoted Anionic-Radical Reaction Cascade. *Org. Lett.* **2017**, *19* (12), 3327–3330. https://doi.org/10.1021/acs.orglett.7b01538.

(918) Kirfel, A.; Schwabenländer, F.; Herdeis, C.; Telser, J. Crystal Structure of 2-Benzyl-7-(Anti)-Hydroxy-7-(Syn)-Methyl-2-Aza-Bicyclo[2.2.2]Oct-5-Ene-3-One, C15H17NO2. *Zeitschrift fur Krist. New Cryst. Struct.* **1997**, *212* (1), 445–446. https://doi.org/10.1524/ncrs.1997.212.1.445.

(919) Abdel-Wahab, B. F.; Mohamed, H. A.; Ng, S. W.; Tiekink, E. R. T. 4-{1-[4-(4-Bromophenyl)-1,3-Thiazol-2-Yl]-5-(4-Fluorophenyl)-4, 5-Dihydro-1H-Pyrazol-3-Yl}-5-Methyl-1-(4-Methylphenyl)-1H-1,2,3-Triazole. *Acta Cryst. E* **2012**, *68* (6), o1956–o1957. https://doi.org/10.1107/S1600536812024257.

(920) Abdel-Wahab, B. F.; Abdel-Latif, E.; Mohamed, H. A.; Awad, G. E. A. Design and Synthesis of New 4-Pyrazolin-3-Yl-1,2,3-Triazoles and 1,2,3-Triazol-4-Yl-Pyrazolin-1-Ylthiazoles as Potential Antimicrobial Agents. *Eur. J. Med. Chem.* **2012**, *52*, 263–268. https://doi.org/10.1016/j.ejmech.2012.03.023.

(921) Zhong, Y.; Wu, B. (E)-3-(2-Ethoxyphenyl)-1-{4-[(2-Fluorophenyl)(4-Fluorophenyl)Methyl] Piperazin-1-Yl}prop-2-En-1-One. *Acta Cryst. E* **2012**, *68* (6), o1975–o1975. https://doi.org/10.1107/S1600536812024130.

(922) Darabi, H. R.; Khatamifar, E.; Sharifi, A.; Aghapoor, K.; Jadidi, K. A Divergent McMurry Coupling of the Bis-Salisaldehyde with Methylene Linker: The Characterization of a Key by-Product with the Possible Pathway Formation. *Synth. Commun.* **2017**, *47* (5), 428–434. https://doi.org/10.1080/00397911.2016.1265984.

(923) Bu, X. Bin; Yu, Y.; Li, B.; Zhang, L.; Chen, J. J.; Zhao, Y. L. Copper-Catalyzed Cascade Cyclization Reactions of Isocyanides with α-Diazocarbonyls as N-Terminal Electrophiles: Efficient Synthesis of 2-Imidazolines and 1,1′-Biimidazoles. *Adv. Synth. Catal.* **2017**, *359* (2), 351–356. https://doi.org/10.1002/adsc.201600574.

(924) Dziewulska-Kułaczkowska, A.; Bartyzel, A. Structural and Physicochemical Properties of 3-(3-

Carboxyphenylaminomethylene)-2-Methoxychroman-4-One. *J. Mol. Struct.* **2013**, *1033*, 67–74. https://doi.org/10.1016/j.molstruc.2012.08.013.

(925) Bates, R. W.; Sridhar, S. A Synthesis of (±)-Stemoamide Using the Intramolecular Propargylic Barbier Reaction. *Synlett* **2009**, *2009* (12), 1979–1981. https://doi.org/10.1055/s-0029-1217540.

(926) Melis, N.; Luridiana, A.; Guillot, R.; Secci, F.; Frongia, A.; Boddaert, T.; Aitken, D. J. Stereoselective and Regioselective Pinacol-Type Rearrangement of a Fused Bicyclic Oxetanol Scaffold. *Eur. J. Org. Chem.* **2017**, *2017* (39), 5896–5902. https://doi.org/10.1002/ejoc.201701214.

(927) Chandrasekhar, S.; Gorla, S. K. Novel Cis-Trans Enantiomeric Conglomerates: Triage and Absolute Configurations via Anomalous X-Ray Scattering. A Photochemical Second Order Asymmetric Transformation. *Tetrahedron Asymmetry* **2006**, *17* (15), 2247–2251. https://doi.org/10.1016/j.tetasy.2006.07.033.

(928) Song, D.; McDonald, R.; West, F. G. Diastereoselective [4 + 4]-Photocycloaddition Reactions of Pyran-2-Ones: Rapid Access to Functionalized 5-8-5 Skeletons. *Org. Lett.* **2006**, *8* (18), 4075–4078. https://doi.org/10.1021/ol061576h.

(929) Hejda, M.; Lyčka, A.; Jambor, R.; Růžička, A.; Dostál, L. Reactivity of C,N-Chelated Organoboron Compounds with Lithium Anilides-Formation of Unexpected 1,2,3-Trisubstituted 1H-2,1-Benzazaboroles. *Dalt. Trans.* **2013**, *42* (18), 6417–6428. https://doi.org/10.1039/c3dt32850c.

(930) Manta, E.; Scarone, L.; Hernández, G.; Mariezcurrena, R.; Suescun, L.; Brito, I.; Brouard, I.; González, M. C.; Pérez, R.; Martín, J. D. A Facile Synthesis of an Oxatricyclic Trans-Syn-Trans-Substituted Oxepanyl Framework. *Tetrahedron Lett.* **1997**, *38* (33), 5853–5856. https://doi.org/10.1016/S0040-4039(97)01302-6.

(931) Egli, D. H.; Linden, A.; Heimgartner, H. 1,5-Dipolar Electrocyclizations in Reactions of α-Thioxo Ketones and α-Thioxo Thioamides with Diazo Compounds. *Helv. Chim. Acta* **2006**, *89* (9), 1910–1926. https://doi.org/10.1002/hlca.200690182.

(932) Duff, T.; James, J. P.; Müller-Bunz, H. Synthesis and X-Ray Crystal Structure Determinations of Pyrrolidine-2,4-Diones 2-Imnopyrrolidin-5-Ones and 1,3-Oxazine-2,4-Diones Derived from Acetoacetanilides. *Heterocycles* **2006**, *68* (3), 465–474. https://doi.org/10.3987/COM-05-10568.

(933) Xiao, T.; Lin, Y.; Zhang, X. Q.; Chen, J.; Wang, J. T. (E)-2-[4-(2-Chlorophenyl)-1,3-Dithiolan-2-Ylidene]-2-(Imidazol-1-Yl) Acetonitrile. *Acta Cryst. E* **2006**, *62* (11), o5052–o5053. https://doi.org/10.1107/S1600536806040736.

(934) Butts, C. P.; Calvert, J. L.; Eberson, L.; Hartshorn, M. P.; Robinson, W. T. Formation of a 1,3-Dipolar Nitro Addition Product from the Photochemical Reaction of 1,2-Dimethylnaphthalene and Tetranitromethane. *J. Chem. Soc. Chem. Commun.* **1993**, No. 19, 1513–1514. https://doi.org/10.1039/C39930001513.

(935) Arshad, M. N.; Birinji, A. S.; Khalid, M.; Asiri, A. M.; Al-Amry, K. A.; Aqlan, F. M. S.; Braga, A. A. C. Synthesis, Spectroscopic, Single Crystal Diffraction and Potential Nonlinear Optical Properties of Novel Pyrazoline Derivatives: Interplay of Experimental and Computational Analyses. *Spectrochim. Acta - Part A Mol. Biomol. Spectrosc.* **2018**, *202*, 146–158. https://doi.org/10.1016/j.saa.2018.04.069.

(936) Ngamsomprasert, N.; Yoshida, Y.; Yakiyama, Y.; Ikuma, N.; Sakurai, H. Nucleophilic Substitution at the Internal Carbon of Sumanene Framework with Inversion of Configuration. *Chem. Lett.* **2018**, *47* (7), 878–880. https://doi.org/10.1246/cl.180270.

(937) Iwasawa, N.; Watanabe, S.; Ario, A.; Sogo, H. Re(I)-Catalyzed Hydropropargylation of Silyl Enol Ethers Utilizing Dynamic Interconversion of Vinylidene-Alkenylmetal Intermediates via 1,5-Hydride Transfer. *J. Am. Chem. Soc.* **2018**, *140* (25), 7769–7772. https://doi.org/10.1021/jacs.8b02903.

(938) Gujral, J.; Reddy, T. P.; Gorachand, B.; Ramachary, D. B. An Aldehyde-Azomethine Imine [3+2]-Cycloaddition: High-Yielding Regioselective Synthesis of Substituted N,N-Bicyclic Pyrazolidinones. *ChemistrySelect* **2018**, *3* (27), 7900–7905. https://doi.org/10.1002/slct.201801328.

(939) Eberson, L.; Hartshorn, M. P.; Radner, F.; Robinson, W. T.; Hutters, M. B.; Spanget-Larsen, J.; MIlanova, R. K.; Nakata, H.; Nasiri, A.; Okada, Y. Photochemical Nitration by Tetranitromethane. VIII. Isolation, X-Ray Structural Analysis and Chemical Properties of a Vicinal Nitro/Trinitromethyl Adduct from Fluoranthene. *Acta Chem. Scand.* **1993**, *47*, 410–415. https://doi.org/10.3891/acta.chem.scand.47-0410.

(940) Mirnik, J.; Grošelj, U.; Novak, A.; Dahmann, G.; Golobič, A.; Kasunič, M.; Stanovnik, B.; Svete, J. A Novel Synthesis of Tetrahydropyrazolo[1,5-c]Pyrimidine-2,7(1 H,3 H)-Diones. *Synthesis* **2013**, *45* (24), 3404–3412. https://doi.org/10.1055/s-0033-1339977.

(941) Fahey, K.; Coyle, R.; McArdle, P.; Aldabbagh, F. Bu3SnH-Mediated Cyclopropyl Radical Cyclizations onto Indole-3-Carbaldehyde. *Arkivoc* **2013**, *2013* (3), 401–412. https://doi.org/10.3998/ark.5550190.p008.208.

(942) Liu, W.; Wang, B. Synthesis of (±)-Merrilactone A by a Desymmetrization Strategy. *Chem. Eur. J.* **2018**, *24* (62),

16511–16515. https://doi.org/10.1002/chem.201804195.

(943) Tamura, R.; Susuki, S.; Azuma, N.; Matsumoto, A.; Toda, F.; Kamimura, A.; Hori, K. Preparation of Chiral Nitroxide Radicals and Spontaneous Optical Resolution by Recrystallization. *Angew. Chem. Int. Ed.* **1994**, *33* (8), 878–879. https://doi.org/10.1002/anie.199408781.

(944) Prakash, G. K. S.; Zhang, Z.; Wang, F.; Rahm, M.; Ni, C.; Iuliucci, M.; Haiges, R.; Olah, G. A. Stereoselective Synthesis of Fluoroalkenoates and Fluorinated Isoxazolidinones: N-Substituents Governing the Dual Reactivity of Nitrones. *Chem. Eur. J.* **2014**, *20* (3), 831–838. https://doi.org/10.1002/chem.201303509.

(945) Wang, Y.; Wang, J.; Xiong, Y.; Liu, Z. Q. I2O5-Mediated Bromohydroxylation and Dibromination of Olefins Using KBr in Water. *Tetrahedron Lett.* **2014**, *55* (16), 2734–2737. https://doi.org/10.1016/j.tetlet.2014.03.064.

(946) Lee, G. A.; Chen, K. C. The General Synthesis and Trapping of 3-Substituted 1 -Chlorocyclopropenes. *Org. Lett.* **2008**, *10* (9), 1843–1845. https://doi.org/10.1021/ol8004479.

(947) Huang, C.; Qian, H.; Zhang, W.; Ma, S. Hydroxy Group-Enabled Highly Regio- and Stereo-Selective Hydrocarboxylation of Alkynes. *Chem. Sci.* **2019**, *10* (21), 5505–5512. https://doi.org/10.1039/c8sc05743e.

(948) Zhu, X.; Chiba, S. TEMPO-Mediated Allylic C-H Amination with Hydrazones. *Org. Biomol. Chem.* **2014**, *12* (26), 4567–4570. https://doi.org/10.1039/c4ob00839a.

(949) Marchand, A. P.; Sorokin, V. D.; Rajagopal, D.; Bott, S. G. Reactions of Some Sulfur(II)- and Iodine(III)-Containing Electrophiles with Endo-Tricyclo[6.2.1.02,7]Undeca-4,9-Diene-3,6-Dione. *Tetrahedron* **1994**, *50* (33), 9933–9942. https://doi.org/10.1016/S0040-4020(01)89608-3.

(950) Georgieva, A.; Stanoeva, E.; Karamfilova, K.; Spassov, S.; Angelova, O.; Haimova, M.; De Kimpe, N.; Boelens, M. Synthesis of 11H-4b, 10b-Dihydro [1] Benzopyrano [4,3-c] Isoquinoline-6,11 (5H)-Diones and 13H-6c, 12b-Dihydronaphtho [1′,2′:5,6]-Pyrano [4-3c] Isoquinoline-8,13 (7H)-Dione from Homophthalic Anhydride and N-(2-Hydroxyarylidene) Alkylamines. *Tetrahedron* **1994**, *50* (31), 9399–9410. https://doi.org/10.1016/S0040-4020(01)85515-0.

(951) M. Aleksandrov, A.; A. Bourne, S.; Krawiec, M.; J.Pehk, T.; E. Petrenko, A.; H. Watson, W. New Route to 4,4,6,7-Tetrasubstituted Pentacyclo[6.3.0.02,6.03.10.05,9]Undecanes. *Tetrahedron* **1994**, *50* (29), 8597–8602. https://doi.org/10.1016/S0040-4020(01)85334-5.

(952) Maria, T. M. R.; Marins, F. A.; Costa, J. B. S.; Silva, M. R.; Carrilho, R. M. B.; Monteiro, C. J. P.; Pereira, M. M.; Eusébio, M. E. S. Solid State Investigation of BINOL and BINOL Derivatives: A Contribution to Enantioselective Symmetry Breaking by Crystallization. *Thermochim. Acta* **2017**, *648*, 32–43. https://doi.org/10.1016/j.tca.2016.12.008.

(953) Seibold, S.; Schäfer, A.; Lohstroh, W.; Walter, O.; Döring, M. Phosphorus-Containing Terephthaldialdehyde Adducts-Structure Determination and Their Application as Flame Retardants in Epoxy Resins. *J. Appl. Polym. Sci.* **2008**, *108* (1), 264–271. https://doi.org/10.1002/app.27550.

(954) Hu, X. Y.; Yang, X. Y.; Cheng, M.; Zhang, Q.; Wei, W.; Ji, J. X. Efficient Synthesis of α, β-Diamino Acid Derivatives by in(III)-Catalyzed Regioselective Addition of Amines to Aziridines. *Synth. Commun.* **2012**, *42* (23), 3403–3412. https://doi.org/10.1080/00397911.2011.582977.

(955) Pawar, S. K.; Vasu, D.; Liu, R. S. Gold- and Silver-Catalyzed [4+2]Cycloadditions of Ynamides with Oxetanes and Azetidines. *Adv. Synth. Catal.* **2014**, *356* (11–12), 2411–2416. https://doi.org/10.1002/adsc.201400024.

(956) Tang, J.; Grampp, G.; Liu, Y.; Wang, B. X.; Tao, F. F.; Wang, L. J.; Liang, X. Z.; Xiao, H. Q.; Shen, Y. M. Visible Light Mediated Cyclization of Tertiary Anilines with Maleimides Using Nickel(II) Oxide Surface-Modified Titanium Dioxide Catalyst. *J. Org. Chem.* **2015**, *80* (5), 2724–2732. https://doi.org/10.1021/jo502901h.

(957) Cernak, T. A.; Lambert, T. H. Multicatalytic Synthesis of α-Pyrrolidinyl Ketones via a Tandem Palladium(II)/Indium(III)-Catalyzed Aminochlorocarbonylation/Friedel-Crafts Acylation Reaction. *J. Am. Chem. Soc.* **2009**, *131* (9), 3124–3125. https://doi.org/10.1021/ja809897f.

(958) Barton, T. J.; Wulff, W. D.; Arnold, E. V.; Clardy, J. Silicon-Carbon Double-Bond Formation via 1, 5-Sigmatropic Migration of Trimethylsilyl from Silicon to Carbon. *J. Am. Chem. Soc.* **1979**, *101* (10), 2733–2735. https://doi.org/10.1021/ja00504a042.

(959) Wilson, R. M.; Rekers, J. W.; Packard, A. B.; Elder, R. C. Intra- and Intermolecular Cyclization of Olefinic Tosylhydrazones Under Acidic Conditions. A Facile Synthesis of Bicyclic Azoalkanes. *J. Am. Chem. Soc.* **1980**, *102* (5), 1633–1641. https://doi.org/10.1021/ja00525a029.

(960) Bakthadoss, M.; Vinayagam, V. A Novel Protocol for the Facile Construction of Tetrahydroquinoline Fused Tricyclic Frameworks via an Intramolecular 1,3-Dipolar Nitrile Oxide Cycloaddition Reaction. *Org. Biomol. Chem.* **2015**, *13* (39), 10007–10014. https://doi.org/10.1039/c5ob01060h.

(961) Veera Reddy, P.; Manisekaran, T.; Bhat, S. V. Novel Synthesis of Trioxatetracyclo[5.3.2.0.4,9.04,11]Dodecane and Bibenzyl Skeletons. *Tetrahedron Lett.* **1998**, *39* (12), 1629–1632. https://doi.org/10.1016/S0040-4039(97)10858-9.

(962) Shih, Y. C.; Tsai, P. H.; Hsu, C. C.; Chang, C. W.; Jhong, Y.; Chen, Y. C.; Chien, T. C. Biomimetic Approach toward the Total Synthesis of Rac-2-(Acylmethylene)Pyrrolidine Alkaloids. *J. Org. Chem.* **2015**, *80* (13), 6669–6678. https://doi.org/10.1021/acs.joc.5b00836.

(963) Brookes, P. C.; Murphy, P. J.; Sommer, K.; Hibbs, D. E.; Hursthouse, M. B.; Malik, K. M. A. Disparity in the Tandem Epoxide-Allylic Alcohol-[1,2]/[2,3]-Wittig Rearrangement of Cis- and Trans-1-Benzyloxy-3,4-Epoxycyclopentane. *J. Chem. Soc. Perkin Trans. 1* **1998**, No. 12, 1899–1901. https://doi.org/10.1039/a801598h.

(964) Iwama, T.; Matsumoto, H.; Shimizu, H.; Kataoka, T.; Muraoka, O.; Tanabe, G. Pummerer Reaction of 2-Vinylcyclopropyl Sulfoxides: Generation and Reactions of Butadienylthionium Ion Intermediates. *J. Chem. Soc. Perkin Trans. 1* **1998**, No. 9, 1569–1576. https://doi.org/10.1039/a708724a.

(965) Ghosh, P.; Deka, M. J.; Saikia, A. K. Lewis Acid Mediated Intramolecular C-O Bond Formation of Alkanol-Epoxide Leading to Substituted Morpholine and 1,4-Oxazepane Derivatives: Total Synthesis of (±)-Viloxazine. *Tetrahedron* **2016**, *72* (5), 690–698. https://doi.org/10.1016/j.tet.2015.12.015.

(966) Eipert, M.; Maichle-Mössmer, C.; Maier, M. E. Acid-Induced Rearrangement Reactions of Reduced Benzoquinone Cyclopentadiene Cycloadducts. *J. Org. Chem.* **2002**, *67* (24), 8692–8695. https://doi.org/10.1021/jo026238i.

(967) Zhang, H.; Chan, W. H.; Lee, A. W. M.; Wong, W. Y. 1,3-Dipolar Cycloadditions of Prop-1-Ene-1,3-Sultone with Nitrile Oxides/Nitrones. *Tetrahedron Lett.* **2003**, *44* (2), 395–397. https://doi.org/10.1016/S0040-4039(02)02502-9.

(968) Bland, D.; Chambournier, G.; Dragan, V.; Hart, D. J. Intramolecular Conjugate Addition Reactions of Amines and Carbamates to 2,5-Cyclohexadien-1-Ones: Stereoselective Synthesis of Perhydroindoles. *Tetrahedron* **1999**, *55* (29), 8953–8966. https://doi.org/10.1016/S0040-4020(99)00455-X.

(969) Kroulík, J.; Čejka, J.; Sedmera, P.; Jegorov, A.; Kratochvíl, B.; Kuthan, J. Rearrangement of Substituted 2,4,4,6-Tetraaryl-4H-Thiopyrans to Triaryl-3aH-Benzo[3,4]Cyclopenta[1,2-b]Thiophene. *Collect. Czechoslov. Chem. Commun.* **2004**, *69* (8), 1631–1642. https://doi.org/10.1135/cccc20041631.

(970) Gudimalla, N.; Fröhlich, R.; Hoppe, D. Stereoselective Synthesis of Baylis-Hillman-Type Adducts via Allenolates Generated by Acyl Migration. *Org. Lett.* **2004**, *6* (22), 4005–4008. https://doi.org/10.1021/ol0483908.

(971) Silva, L. F.; Craveiro, M. V.; Gambardella, M. T. P. Synthesis of Polyalkylated Indoles Using a Thallium(III)-Mediated Ring-Contraction Reaction. *Synthesis* **2007**, *2007* (24), 3851–3857. https://doi.org/10.1055/s-2007-990907.

(972) Patil, N. T.; Lakshmi, P. G. V. V.; Sridhar, B.; Patra, S.; Pal Bhadra, M.; Patra, C. R. New Linearly and Angularly Fused Quinazolinones: Synthesis through Gold(I)-Catalyzed Cascade Reactions and Anticancer Activities. *Eur. J. Org. Chem.* **2012**, *2012* (9), 1790–1799. https://doi.org/10.1002/ejoc.201101822.

(973) Kawade, R. K.; Liu, R. S. Copper-Catalyzed Three-Component Annulations of Alkenes, Nitrosoarenes, and N-Hydroxyallylamines To Form Fused Oxazinane/Isoxazolidine Heterocycles. *Angew. Chem. Int. Ed.* **2017**, *56* (8), 2035–2039. https://doi.org/10.1002/anie.201611388.

(974) Liu, W.; Du, S. T.; Wang, S. Y.; Liao, W. W. Controllable Diastereodivergent Synthesis of Pyrrolo[2,1-a]Isoquinolines via Catalytic Intramolecular Acylsulfenylation of Activated Alkenes. *J. Org. Chem.* **2017**, *82* (9), 4829–4839. https://doi.org/10.1021/acs.joc.7b00551.

(975) Zbancioc, G.; Florea, O.; Jones, P. G.; Mangalagiu, I. I. An Efficient and Selective Way to New Highly Functionalized Coronands or Spiro Derivatives Using Ultrasonic Irradiation. *Ultrason. Sonochem.* **2012**, *19* (3), 399–403. https://doi.org/10.1016/j.ultsonch.2011.08.001.

(976) Knobloch, K.; Koch, J.; Keller, M.; Eberbach, W. The Dipolar Route to Azepin-3-One Derivatives by Heterocyclization of Linear and Monocyclic Enallenyl Nitrones as the Key Step. *Eur. J. Org. Chem.* **2005**, *2005* (13), 2715–2733. https://doi.org/10.1002/ejoc.200400922.

(977) Mirzayans, P. M.; Pouwer, R. H.; Williams, C. M.; Bernhardt, P. V. Stereocontrolled Synthesis of the Cis-Hydroxydecalin System: Towards Biologically Active 19-nor-Clerodanes. *Eur. J. Org. Chem.* **2012**, No. 8, 1633–1638. https://doi.org/10.1002/ejoc.201101807.

(978) Adamski, A.; Kruszka, D.; Dutkiewicz, Z.; Kubicki, M.; Gorczyński, A.; Patroniak, V. Novel Family of Fused Tricyclic [1,4]Diazepines: Design, Synthesis, Crystal Structures and Molecular Docking Studies. *Tetrahedron* **2017**, *73* (24), 3377–3386. https://doi.org/10.1016/j.tet.2017.05.015.

(979) Mukai, C.; Inagaki, F.; Yoshida, T.; Yoshitani, K.; Hara, Y.; Kitagaki, S. Rh(I)-Catalyzed Pauson-Khand Reaction and Cycloisomerization of Allenynes: Selective Preparation of Monocyclic, Bicyclo[m.3.0], and Bicyclo[5.2.0] Ring Systems. *J. Org. Chem.* **2005**, *70* (18), 7159–7171. https://doi.org/10.1021/jo050770z.

(980) Usman, R.; Khan, A.; Wang, M. Study of H-Bonded Assemblies of the Solvates of Anthracene Derivatives: Guest Effect on the Crystal Symmetry and Spectroscopic Properties. *Supramol. Chem.* **2017**, *29* (7), 497–505. https://doi.org/10.1080/10610278.2017.1284324.

(981) Magnus, P.; Stent, M. A. H. Stereospecific Synthesis of (±)-1,2-Anhydro Methyl Rocaglate. *Org. Lett.* **2005**, *7* (18), 3853–3855. https://doi.org/10.1021/ol0513793.

(982) Lu, Y.; Lemal, D. M.; Jasinski, J. P. Bond Alternation in Azulenes. *J. Am. Chem. Soc.* **2000**, *122* (11), 2440–2445. https://doi.org/10.1021/ja984113i.

(983) Boisbrun, M.; Jeannin, L.; Toupet, L.; Laronze, J. Y. A Convenient Synthesis of Indole-Substituted 2-Pyrrolidones and Their Cyclized Derivatives. *Eur. J. Org. Chem.* **2000**, No. 17, 3051–3057. https://doi.org/10.1002/1099-0690(200009)2000:17<3051::AID-EJOC3051>3.0.CO;2-3.

(984) Enders, D.; Kroll, M.; Raabe, G.; Runsink, J. Zirconocene Mediated Diastereo- and Enantioselective Synthesis of 4,5,5-Trisubstituted 2-Alkoxytetrahydrofurans. *Mol. Online* **1998**, *2* (8), 109–113. https://doi.org/10.1007/s007830050065.

(985) Hong, B. C.; Shen, I. C.; Liao, J. H. Unprecedented Sequential Oxidative Dimerization and Cycloaddition of 1,3-Diketones to Fulvenes. A Facile Synthesis of the Cyclopenta[b]Chromenes. *Tetrahedron Lett.* **2001**, *42* (5), 935–938. https://doi.org/10.1016/S0040-4039(00)02139-0.

(986) Paul, A.; Thimmegowda, N. R.; Galani Cruz, T.; Seidel, D. Decarboxylative Annulation of α-Amino Acids with β-Ketoaldehydes. *Org. Lett.* **2018**, *20* (3), 602–604. https://doi.org/10.1021/acs.orglett.7b03721.

(987) Kamikawa, K.; Shimizu, Y.; Takemoto, S.; Matsuzaka, H. Nickel-Catalyzed [3+1+1] Cycloaddition Reactions of Alkenyl Fischer Carbene Complexes with Methylenecyclopropanes. *Org. Lett.* **2006**, *8* (18), 4011–4014. https://doi.org/10.1021/ol061559s.

(988) Ganesh Kumar, R.; Gayathri, D.; Velmurugan, D.; Ravikumar, K.; Poornachandran, M. 1′-Methyl-4′-Phenyl-1H-Indole-3-Spiro-2′-Pyrrolidine- 3′-Spiro-1″-Cyclooctane-2(3H),2″-Dione. *Acta Cryst. E* **2006**, *62* (11), o4821–o4823. https://doi.org/10.1107/S1600536806039481.

(989) Zhang, K.; Cai, L.; Yang, Z.; Houk, K. N.; Kwon, O. Bridged [2.2.1] Bicyclic Phosphine Oxide Facilitates Catalytic γ-Umpolung Addition-Wittig Olefination. *Chem. Sci.* **2018**, *9* (7), 1867–1872. https://doi.org/10.1039/c7sc04381c.

(990) Salvati, A. E.; Law, J. A.; Liriano, J.; Frederich, J. H. Modular Access to Functionalized 5-8-5 Fused Ring Systems: Via a Photoinduced Cycloisomerization Reaction. *Chem. Sci.* **2018**, *9* (24), 5389–5393. https://doi.org/10.1039/c8sc00999f.

(991) Gao, F.; Zhang, G.; Zhang, S.; Cheng, Y.; Shi, Z.; Li, Y.; Gao, J. Different N-C-N Formation Reactions of Aromatic Aldehydes and Thiohydantoins Controlled by Lewis Acid Promoters. *Tetrahedron* **2007**, *63* (19), 3973–3981. https://doi.org/10.1016/j.tet.2007.03.002.

(992) Yücel, B.; Arve, L.; De Meijere, A. A Two-Step Four-Component Queuing Cascade Involving a Heck Coupling, π-Allylpalladium Trapping and Diels-Alder Reaction. *Tetrahedron* **2005**, *61* (48), 11355–11373. https://doi.org/10.1016/j.tet.2005.09.014.

(993) García-López, J. A.; Oliva-Madrid, M. J.; Saura-Llamas, I.; Bautista, D.; Vicente, J. Reactivity toward CO of Eight-Membered Palladacycles Derived from the Insertion of Alkenes into the Pd-C Bond of Cyclopalladated Primary Arylalkylamines of Pharmaceutical Interest. Synthesis of Tetrahydrobenzazocinones, Ortho-Functionalized Phenethylamine. *Organometallics* **2012**, *31* (17), 6351–6364. https://doi.org/10.1021/om300593x.

(994) Mangion, D.; Arnold, D. R.; Cameron, S. S.; Robertson, K. N. The Electron Transfer Photochemistry of Allenes with Cyanoarenes. Photochemical Nucleophile-Olefin Combination, Aromatic Substitution (Photo-NOCAS) and Related Reactions. *J. Chem. Soc. Perkin Trans. 2* **2001**, No. 1, 48–60. https://doi.org/10.1039/b007205m.

(995) Crisma, M.; Possenti, M.; De Lucchi, O.; Lazzari, D.; Soverini, M. Crystal Structure of 2α-(1,2-Dibromoisopropyl)-5α-Nitro-5β-Methyl-1,3-Dioxane, C8H13NO4Br2. *Zeitschrift fur Krist. New Cryst. Struct.* **2001**, *216* (1–4), 103–104. https://doi.org/10.1524/ncrs.2001.216.14.103.

(996) Akpınar, D. E.; Özgür (Nee Yakut), M.; Aslan, H.; Alagöz, O.; Öktemer, A.; Dal, H.; Hökelek, T.; Loğoğlu, E. Synthesis, Characterization, and Investigations of Antimicrobial Activity of Benzopyrans, Benzofurans and Spiro[4.5]Decanes. *Synth. Commun.* **2018**, *48* (19), 2510–2521. https://doi.org/10.1080/00397911.2018.1509094.

(997) Campbell, E. L.; Skepper, C. K.; Sankar, K.; Duncan, K. K.; Boger, D. L. Transannular Diels-Alder/1,3-Dipolar Cycloaddition Cascade of 1,3,4-Oxadiazoles: Total Synthesis of a Unique Set of Vinblastine Analogues. *Org. Lett.* **2013**, *15* (20), 5306–5309. https://doi.org/10.1021/ol402549n.

(998) Saitoh, H.; Ijuin, H. K.; Watanabe, N.; Matsumoto, M. Novel Intramolecular Cyclization of 2-(Buta-1,3-Dienyl)Benzyl Anions to 6,7(9)-Dihydro-5H-Benzocycloheptenyl Anions Leading to Successive Formation of 1,2-Dihydrocyclopropa[a]Naphthalenes. *Helv. Chim. Acta* **2013**, *96* (9), 1704–1713. https://doi.org/10.1002/hlca.201200653.

(999) Guo, Z. W.; Huang, X.; Mao, J. M.; Zhu, W. D.; Xie, J. W. Diastereoselective Synthesis of Polysubstituted Δ1-Pyrroline Derivatives from in Situ Generated Nitrile Ylides. *RSC Adv.* **2013**, *3* (47), 25103–25109. https://doi.org/10.1039/c3ra44736g.

(1000) Macomber, R. S.; Guttadauro, M.; Pinhas, A. R.; Bauer, J. K. Reactions of 1,2-Oxaphospholenes. 9. Attempted Deprotonation at C5. *J. Org. Chem.* **2001**, *66* (4), 1480–1483. https://doi.org/10.1021/jo000751j.

(1001) Kamimura, A.; Omata, Y.; Mitsudera, H.; Kakehi, A. A Simple Preparation of Syn-NH-Amide Aldols and Amide-Baylis-Hillman Adducts via a Michael-Aldol Tandem Process. *J. Chem. Soc. Perkin Trans. 1* **2000**, No. 24, 4499–4504. https://doi.org/10.1039/b004721j.

(1002) Morisaki, Y.; Kondo, T.; Mitsudo, T. A. A New Route to Cyclopentenones via Ruthenium-Catalyzed Carbonylative Cyclization of Allylic Carbonates with Alkenes. *Org. Lett.* **2000**, *2* (7), 949–952. https://doi.org/10.1021/ol0000206.

(1003) Guerrero, S. A.; Sanabría, C. M.; Palma, A.; Cobo, J.; Glidewell, C. Four Related Benzazepine Derivatives in a Reaction Pathway Leading to a Benzazepine Carboxylic Acid: Hydrogen-Bonded Assembly in Zero, One, Two and Three Dimensions. *Acta Crystallogr. Sect. C Struct. Chem.* **2014**, *70* (4), 408–415. https://doi.org/10.1107/S2053229614006007.

(1004) Bringmann, G.; Hinrichs, J.; Pabst, T.; Henschel, P.; Peters, K.; Peters, E. M. From Dynamic to Non-Dynamic Kinetic Resolution of Lactone-Bridged Biaryls: Synthesis of Mastigophorene B. *Synthesis* **2001**, No. 1, 155–167. https://doi.org/10.1055/s-2001-9760.

(1005) Douglas, N.; Neef, C. J.; Rogers, R. A.; Stanley, J. A.; Armitage, J.; Martin, B.; Hudnall, T. W.; Brittain, W. J. Reactivity of Tetrahydrochromeno[2,3-b]Indoles: Chromic Indicators of Cyanide. *J. Phys. Org. Chem.* **2013**, *26* (8), 688–695. https://doi.org/10.1002/poc.3158.

(1006) Buchlovič, M.; Man, S.; Potáček, M. Allenyloxime-a New Source of Heterocyclizations to Stable Cyclic Nitrones. *Tetrahedron* **2008**, *64* (42), 9953–9961. https://doi.org/10.1016/j.tet.2008.07.113.

(1007) Buchlovič, M.; Hebanová, S.; Potáček, M. 1,3-Dipolar Cycloadditions of New 2,5-Bifunctionalized Five-Membered Cyclic Nitrones. *Tetrahedron* **2012**, *68* (14), 3117–3122. https://doi.org/10.1016/j.tet.2012.01.043.

(1008) Lu, J. M.; Zhu, Z. Bin; Shi, M. Lewis Acid or Brønsted Acid Catalyzed Reactions of Vinylidene Cyclopropanes with Activated Carbon-Nitrogen, Nitrogen-Nitrogen, and Iodine-Nitrogen Double-Bond-Containing Compounds. *Chem. Eur. J.* **2009**, *15* (4), 963–971. https://doi.org/10.1002/chem.200801785.

(1009) Huang, P. J. J.; Stanley Cameron, T.; Jha, A. Novel Synthesis of 2,2-Dialkyl-3-Dialkylamino-2,3-Dihydro-1H-Naphtho[2,1-b]Pyrans. *Tetrahedron Lett.* **2009**, *50* (1), 51–54. https://doi.org/10.1016/j.tetlet.2008.10.083.

(1010) Shen, Z.; Dong, V. M. Benzofurans Prepared by C-H Bond Functionalization with Acylsilanes. *Angew. Chem. Int. Ed.* **2009**, *48* (4), 784–786. https://doi.org/10.1002/anie.200804854.

(1011) Miesch, L.; Welsch, T.; Rietsch, V.; Miesch, M. Intramolecular Alkynylogous Mukaiyama Aldol Reaction Starting from Bicyclic Alkanones Tethered to Alkynyl Esters: Formal Total Synthesis Of (±)-Hamigeran B. *Chem. Eur. J.* **2009**, *15* (17), 4394–4401. https://doi.org/10.1002/chem.200802309.

(1012) Tang, H. T.; Xiong, K.; Li, R. H.; Ding, Z. C.; Zhan, Z. P. Synthesis of 5,6-Dihydropyrazolo[1,5- c]Quinazolines through Gold-Catalyzed Chemoselective Bicyclization of N -Propargylic Sulfonylhydrazones. *Org. Lett.* **2015**, *17* (2), 326–329. https://doi.org/10.1021/ol503437n.

(1013) Wagner, T.; Lange, J.; Grote, D.; Sander, W.; Schaumann, E.; Adiwidjaja, G.; Adam, A.; Kopf, J. Organylthio(Silyl)Carbenes. *Eur. J. Org. Chem.* **2009**, *2009* (30), 5198–5207. https://doi.org/10.1002/ejoc.200900482.

(1014) Peng, Y.; Duan, S. M.; Wang, Y. W. Concise Synthesis of the DEFG Ring System in Rubriflordilactone B. *Tetrahedron Lett.* **2015**, *56* (30), 4509–4511. https://doi.org/10.1016/j.tetlet.2015.05.117.

(1015) Foces-Foces, C.; Jagerovic, N.; Elguero, J. Crystal Structure of 1,3,5-Triphenyl-2-Pyrazoline: A Case of Spontaneous Resolution. *Zeitschrift fur Krist.* **2001**, *216* (4), 240–244. https://doi.org/10.1524/zkri.216.4.240.23253.

(1016) Hansen, L. K.; Bayer, A.; Gautun, O. R. Exo -3,6-Dihydro-3-Methyl-2-(Toluene-4-Sulfonyl)-1λ 4 ,2-Thiazine 1-Oxide . *Acta Cryst. E* **2001**, *57* (11), o1109–o1110. https://doi.org/10.1107/s1600536801017901.

(1017) Nguyen, V. T.; Ahn, P. D.; Bishop, R.; Scudder, M. L.; Craig, D. C. Molecular Solids Formed by the Self-Organisation of Dialcohols into Hydrogen-Bonded Ladders. *Eur. J. Org. Chem.* **2001**, No. 23, 4489–4499. https://doi.org/10.1002/1099-0690(200112)2001:23<4489::AID-EJOC4489>3.0.CO;2-Z.

(1018) Yang, X. L.; Peng, X. X.; Chen, F.; Han, B. TEMPO-Mediated Aza-Diels-Alder Reaction: Synthesis of Tetrahydropyridazines Using Ketohydrazones and Olefins. *Org. Lett.* **2016**, *18* (9), 2070–2073. https://doi.org/10.1021/acs.orglett.6b00702.

(1019) Barakat, A.; Islam, M. S.; Al-Majid, A. M.; Soliman, S. M.; Mabkhot, Y. N.; Al-Othman, Z. A.; Ghabbour, H. A.; Fun, H. K. Synthesis of Novel 5-Monoalkylbarbiturate Derivatives: New Access to 1,2-Oxazepines. *Tetrahedron Lett.* **2015**, *56* (50), 6984–6987. https://doi.org/10.1016/j.tetlet.2015.10.108.

(1020) Padwa, A.; Krumpe, K. E.; Weingarten, M. D. An Unusual Example of a 6-Endo-Dig Addition to an Unactivated Carbon-Carbon Triple Bond. *J. Org. Chem.* **1995**, *60* (17), 5595–5603. https://doi.org/10.1021/jo00122a047.

(1021) Padwa, A.; Hertzog, D. L.; Nadler, W. R. Intramolecular Cycloaddition of Isomünchnone Dipoles to Heteroaromatic π-Systems. *J. Org. Chem.* **1994**, *59* (23), 7072–7084. https://doi.org/10.1021/jo00102a037.

(1022) Quast, H.; Becker, C.; Peters, E. M.; Peters, K.; Von Schnering, H. G. A Novel Synthesis and the Autoxidation of 2,4,6,8-Tetraphenylbarbaralane. *Liebigs Ann.* **1997**, No. 4, 685–698. https://doi.org/10.1002/jlac.199719970409.

(1023) Marzouk, A. A.; Abdelhamid, A. A.; Mohamed, S. K.; Simpson, J. Morpholinium Hydrogen Sulfate (MHS) Ionic Liquid as an Efficient Catalyst for the Synthesis of Bioactive Multi-Substituted Imidazoles (MSI) under Solvent-Free Conditions. *Zeitschrift fur Naturforsch. - Sect. B J. Chem. Sci.* **2017**, *72* (1), 23–33. https://doi.org/10.1515/znb-2016-0121.

(1024) Kojima, R.; Sawamoto, S.; Okamura, A.; Takahashi, H.; Tsunoi, S.; Shibata, I. Synthesis of 4-Hydroxy-2-Oxazolidinones Catalyzed by Tin Alkoxides. *Eur. J. Org. Chem.* **2011**, *2011* (36), 7255–7258. https://doi.org/10.1002/ejoc.201101465.

(1025) Chen, J.; Ni, S.; Ma, S. Tuning of Regioselectivity in Inorganic Iodide-Catalyzed Alkylation of 2-Methoxyfurans via Electronic and Steric Effects. *Adv. Synth. Catal.* **2012**, *354* (6), 1114–1128. https://doi.org/10.1002/adsc.201100337.

(1026) Shintani, R.; Ito, T.; Hayashi, T. Palladium-Catalyzed Synthesis of 4-Oxaspiro[2.4]Heptanes via Central Attack of Oxygen Nucleophiles to π-Allylpalladium Intermediates. *Org. Lett.* **2012**, *14* (9), 2410–2413. https://doi.org/10.1021/ol300852v.

(1027) Noji, M.; Kadowaki, H.; Kubota, Y.; Yoshida, T.; Saito, N.; Yamaguchi, S.; Ohata, R.; Ishii, K.; Takanami, T. Diastereoselective Synthesis of 2,4-Substituted Tetrahydroquinolines via Hf(Otf)4-Catalyzed Substitution/Cyclization of 2-Aminobenzyl Alcohols with Styrenes. *Heterocycles* **2017**, *95* (2), 1041–1073. https://doi.org/10.3987/COM-16-S(S)76.

(1028) Helliwell, M.; Fengas, D.; Knight, C. K.; Parker, J.; Quayle, P.; Raftery, J.; Richards, S. N. Bifurcate, Tandem ATRC Reactions: Towards 2-Oxabicyclo[4.3.0]Nonane Core of Eunicellins. *Tetrahedron Lett.* **2005**, *46* (42), 7129–7134. https://doi.org/10.1016/j.tetlet.2005.08.104.

(1029) Santini, A.; Benedetti, E.; Pedone, C.; Caliendo, G.; Santagada, V.; Grieco, P.; Perissutti, E. Molecular Structures of Quinuclidinic Neurokinin Antagonists: 2-(2-Phenylbenzylidene)-3-(2-X-Benzylamino) Derivatives. *Struct. Chem.* **1996**, *7* (3), 173–181. https://doi.org/10.1007/BF02281228.

(1030) Heinisch, G.; Holzer, W.; Mereiter, K.; Strobl, B.; Zheng, C. Formation of Spiro-Lactols upon Reaction of Ninhydrin with Methyldiazines. *Heterocycles* **1996**, *43* (8), 1665–1679. https://doi.org/10.3987/com-96-7488.

(1031) Zhang, D.; Zhang, R.; Liang, Y.; Dong, D. Formal [4+1] Annulation of Cyclopropyl Amides and Water Mediated by Lewis Acid: A Novel Entry to γ-Butyrolactones. *Synthesis* **2012**, *44* (11), 1679–1685. https://doi.org/10.1055/s-0031-1290971.

(1032) Suárez-Castillo, O. R.; Sánchez-Zavala, M.; Meléndez-Rodríguez, M.; Castelán-Duarte, L. E.; Morales-Ríos, M. S.; Joseph-Nathan, P. Preparation of 3-Hydroxyoxindoles with Dimethyldioxirane and Their Use for the Synthesis of Natural Products. *Tetrahedron* **2006**, *62* (13), 3040–3051. https://doi.org/10.1016/j.tet.2006.01.036.

(1033) Ishii, A.; Ohishi, M.; Matsumoto, K.; Takayanagi, T. Synthesis and Properties of a Dithiirane Trans-1,2-Dioxide, a Three-Membered Vic-Disulfoxide. *Org. Lett.* **2006**, *8* (1), 91–94. https://doi.org/10.1021/ol052570f.

(1034) Kajiyama, K.; Kojima, S.; Akiba, K. Y. Synthesis and Characterization of Intra- and Intermolecular Hydrogen Bonding Isomers of P-H (Apical) Phosphoranes Bearing a Hydroxyl Group and Their Thermal Cyclization.

Tetrahedron Lett. **1996**, *37* (46), 8409–8412. https://doi.org/10.1016/0040-4039(96)01923-5.

(1035) Meng, F.; Zhang, H.; Guo, K.; Lu, J. D. A. M.; Zhu, Y. Access to Cyano-Containing Isoxazolines via Copper-Catalyzed Domino Cyclization/Cyanation of Alkenyl Oximes. *J. Org. Chem.* **2017**, *82* (19), 10742–10747. https://doi.org/10.1021/acs.joc.7b02133.

(1036) Shao, P. L.; Li, Z. R.; Wang, Z. P.; Zhou, M. H.; Wu, Q.; Hu, P.; He, Y. [3 + 2] Cycloaddition of Azaoxyallyl Cations with Cyclic Ketones: Access to Spiro-4-Oxazolidinones. *J. Org. Chem.* **2017**, *82* (19), 10680–10686. https://doi.org/10.1021/acs.joc.7b01728.

(1037) Aggarwal, V. K.; Boccardo, G.; Worrall, J. M.; Adams, H.; Alexander, R. 2-Halogeno-1,3-Dithiane 1,3-Dioxide: A Diastereoselective Carbonyl Anion Equivalent in Reactions with Aldehydes. *J. Chem. Soc. Perkin Trans. 1* **1997**, No. 1, 11–19. https://doi.org/10.1039/a603416k.

(1038) Yeh, M. C. P.; Liang, C. J.; Liu, C. Y.; Shih, Y. F.; Lee, I. C.; Liu, H. F.; Wang, J. L. Diastereoselective Synthesis of Vicinal Cis-Dihydroxyheterospirocycles by One-Pot Epoxidation/Spirocyclization of C(3)-Functionalized Cyclohex-2-En-1-Ols. *Org. Biomol. Chem.* **2013**, *11* (20), 3393–3399. https://doi.org/10.1039/c3ob27352k.

(1039) Feng, Q.; Wang, M.; Dong, B.; Xu, C.; Zhao, J.; Zhang, H. Tuning Solid-State Fluorescence of Pyrene Derivatives via a Cocrystal Strategy. *CrystEngComm* **2013**, *15* (18), 3623–3629. https://doi.org/10.1039/c3ce27102a.

(1040) Zhu, Y.; Guo, J.; Jin, S.; Guo, J.; Bai, X.; Wang, Q.; Bu, Z. Construction of Bridged Cyclic: N, O -Ketal Spirooxindoles through a Michael Addition/ N, O -Ketalization Sequence. *Org. Biomol. Chem.* **2018**, *16* (10), 1751–1759. https://doi.org/10.1039/c8ob00306h.

(1041) Deredas, D.; Albrecht; Maniukiewicz, W.; Wojciechowski, J.; Wolf, W. M.; Paluch, P.; Janecki, T.; Rózalski, M.; Krajewska, U.; Janecka, A.; Krawczyk, H. Three-Component Reaction of 3-(Diethoxyphosphoryl)Coumarin, Enolizable Ketones and Primary Amines: Simple, Stereoselective Synthesis of Benzo[1,3]Oxazocine Skeletons. *RSC Adv.* **2013**, *3* (19), 6821–6832. https://doi.org/10.1039/c3ra40184g.

(1042) Muthusamy, S.; Krishnamurthi, J.; Suresh, E. Highly Regio- and Chemoselective Ring Opening of Oxa-Bridged Piperidinones toward Functionalized Furanones and Piperidines. *Org. Lett.* **2006**, *8* (22), 5101–5104. https://doi.org/10.1021/ol0620038.

(1043) Mahieu, A.; Miquel, Y.; Igau, A.; Donnadieu, B.; Majoral, J. P. Versatile Behavior of a New Class of Bicyclic Compounds: Zirconacyclopentadiene Phosphiranes. *Organometallics* **1997**, *16* (14), 3086–3088. https://doi.org/10.1021/om970216i.

(1044) Švorc, Ľ.; Vrábel, V.; Kožíšek, J.; Marchalín, Š.; Šafář, P. Dimethyl 2-[(E)-(Hydroxyimino)Methyl]-6-Methyl-4-(2-Thienyl)-1,4- Dihydropyridine-3,5-Dicarboxylate. *Acta Cryst. E* **2007**, *63* (5), o2240–o2242. https://doi.org/10.1107/S1600536807014122.

(1045) Ader, T. A.; Champey, C. A.; Kuznetsova, L. V.; Li, T.; Lim, Y. H.; Rucando, D.; Sieburth, S. M. N. Polyquinanes by [4 + 4] Cycloaddition - Transannular Cyclization. *Org. Lett.* **2001**, *3* (14), 2165–2167. https://doi.org/10.1021/ol016076x.

(1046) Białońska, A.; Ciunik, Z. Spontaneous Racemic Resolution-towards Control of Molecular Recognition Nature. *CrystEngComm* **2013**, *15* (33), 6448–6452. https://doi.org/10.1039/c3ce40778k.

(1047) Rodríguez, J. F.; Marchese, A. D.; Lautens, M. Palladium-Catalyzed Synthesis of Dihydrobenzoindolones via C-H Bond Activation and Alkyne Insertion. *Org. Lett.* **2018**, *20* (14), 4367–4370. https://doi.org/10.1021/acs.orglett.8b01856.

(1048) Hintz, S.; Fröhlich, R.; Mattay, J. PET-Oxidative Cyclization of Unsaturated Silyl Enol Ethers. Regioselective Control by Solvent Effects. *Tetrahedron Lett.* **1996**, *37* (41), 7349–7352. https://doi.org/10.1016/0040-4039(96)01708-X.

(1049) Kozikowski, A. P.; Araldi, G. L.; Ball, R. G. Dipolar Cycloaddition Route to Diverse Analogues of Cocaine: The 6- and 7-Substituted 3-Phenyltropanes. *J. Org. Chem.* **1997**, *62* (3), 503–509. https://doi.org/10.1021/jo961957g.

(1050) Chen, Y. C.; Yang, D. Y. Visible Light-Mediated Synthesis of Quinazolines from 1,2- Dihydroquinazoline 3-Oxides. *Tetrahedron* **2013**, *69* (48), 10438–10444. https://doi.org/10.1016/j.tet.2013.09.089.

(1051) Garve, L. K. B.; Jones, P. G.; Werz, D. B. Ring-Opening 1-Amino-3-Aminomethylation of Donor–Acceptor Cyclopropanes via 1,3-Diazepanes. *Angew. Chem. Int. Ed.* **2017**, *56* (31), 9226–9230. https://doi.org/10.1002/anie.201704619.

(1052) Well, M.; Schmutzler, R. Addition von Dimethylphosphinoxid Bzw. -Sulfid an Carbonylverbindungen; Darstellung von α-Hydroxyphosphinoxiden Bzw. -Sulfiden. *Phosphorus, Sulfur, Silicon Relat. Elem.* **1992**, *72* (1–4), 171–187. https://doi.org/10.1080/10426509208031550.

(1053) Bosc, J. J.; Jarry, C.; Léger, J. M.; Carpy, A. NMR and Crystallographic Evidence for Polymorphism of the N-

Phenyl-N'-[1-(3-(Phenyl-4-Piperazinyl)Propan-2-Ol)]Urea. *J. Chem. Crystallogr.* **1996**, *26* (12), 807–814. https://doi.org/10.1007/BF01670313.

(1054) Dalling, A. G.; Yamauchi, T.; McCreanor, N. G.; Cox, L.; Bower, J. F. Carbonylative C–C Bond Activation of Electron-Poor Cyclopropanes: Rhodium-Catalyzed (3+1+2) Cycloadditions of Cyclopropylamides. *Angew. Chem. Int. Ed.* **2019**, *58* (1), 221–225. https://doi.org/10.1002/anie.201811460.

(1055) Schick, G.; Loew, A.; Nieger, M.; Niecke, E. On the Reaction of Amino Bis(Diorganylamino)Phosphanes with Halogen Phosphanes-P-Hydrogeno(Iminophosphorany1)-Halogenophosphanes and P-Hydrogen-(Iminophosphoranyl)-L-Iminophosphanes. *Heteroat. Chem.* **1996**, *7* (6), 427–435. https://doi.org/10.1002/(SICI)1098-1071(199611)7:6.

(1056) Roßbach, J.; Harms, K.; Koert, U. Regioselective Passerini and Passerini-Knoevenagel Reactions with Vic-Diketo Amides. *Eur. J. Org. Chem.* **2014**, *2014* (5), 993–1006. https://doi.org/10.1002/ejoc.201301548.

(1057) Islam, M.; Kariuki, B. M.; Shafiq, Z.; Wirth, T.; Ahmed, N. Efficient Electrosynthesis of Thiazolidin-2-Imines via Oxysulfurization of Thiourea-Tethered Terminal Alkenes Using the Flow Microreactor. *Eur. J. Org. Chem.* **2019**, *2019* (6), 1371–1376. https://doi.org/10.1002/ejoc.201801688.

(1058) Ashmore, J.; Bishop, R.; Craig, D. C.; Scudder, M. L. 5b,6,7,13b,14,15-Hexahydro-Acridino[4,3-c]Acridine. *Acta Cryst. E* **2008**, *64* (6), o1136–o1136. https://doi.org/10.1107/S1600536808014803.

(1059) Mori, K.; Kurihara, K.; Yabe, S.; Yamanaka, M.; Akiyama, T. Double C(Sp3)-H Bond Functionalization Mediated by Sequential Hydride Shift/Cyclization Process: Diastereoselective Construction of Polyheterocycles. *J. Am. Chem. Soc.* **2014**, *136* (10), 3744–3747. https://doi.org/10.1021/ja412706d.

(1060) He, Z. C.; Mellerup, S. K.; Liu, L.; Wang, X.; Dao, C.; Wang, S. Reversible Photoisomerization from Borepin to Boratanorcaradiene and Double Aryl Migration from Boron to Carbon. *Angew. Chem. Int. Ed.* **2019**, *58* (20), 6683–6687. https://doi.org/10.1002/anie.201902231.

(1061) Perevoshchikova, A. N.; Eroshenko, D. V.; Dmitriev, M. V.; Grishko, V. V.; Shklyaev, Y. V. Synthesis and Prediction of the Ubiquinol-Cytochrome c Reductase Inhibitory Activity of 3,4-Dihydroisoquinolines and 2-Azaspiro[4.5]Decanes (Spiropyrrolines). *J. Heterocycl. Chem.* **2019**, *56* (5), 1634–1645. https://doi.org/10.1002/jhet.3551.

(1062) Gasiecki, A. F.; Cross, J. L.; Henry, R. F.; Gracias, V.; Djuric, S. W. A Flexible Synthesis of Privileged Structural Motifs Using the Ollis-Sweeney Ammonium Ylid Rearrangement. *Tetrahedron Lett.* **2008**, *49* (44), 6286–6288. https://doi.org/10.1016/j.tetlet.2008.08.055.

(1063) Lowry, R. J.; Veige, M. K.; Clément, O.; Abboud, K. A.; Ghiviriga, I.; Veige, A. S. New Constrained-Geometry C2-Symmetric Di-N-Heterocyclic Carbene Ligands and Their Mono- and Dinuclear Rhodium(I) Complexes: Design, Synthesis, and Structural Analysis. *Organometallics* **2008**, *27* (20), 5184–5195. https://doi.org/10.1021/om800471m.

(1064) Wang, X. Z. Crystal Structure of 6-(Hydroxy(Phenyl)Methyl)-6H-Dibenzo[c,e][1,2]-Oxaphosphinine 6-Oxide, C19H15O3P. *Zeitschrift fur Krist. New Cryst. Struct.* **2014**, *229* (3), 227–228. https://doi.org/10.1515/ncrs-2014-0114.

(1065) Sahani, R. L.; Liu, R. S. Gold-Catalyzed Oxidative Arylations of 3-Butyn-1-Ols and 2-Propyn-1-Ols with Nitrones to Yield Distinct Fused Indoles Bearing a Heterocyclic Ring. *ACS Catal.* **2019**, *9* (7), 5890–5896. https://doi.org/10.1021/acscatal.9b01491.

(1066) Sudhir, U.; Rath, N. P.; Nair, M. S. Synthesis of Novel Tetra- and Pentacyclic Aza-Cage Systems. *Tetrahedron* **2001**, *57* (36), 7749–7753. https://doi.org/10.1016/S0040-4020(01)00741-4.

(1067) Pfeiffer, W. D.; Roßberg, H.; Kelzhanova, N.; Saginayev, A. T.; Villinger, A.; Langer, P. Synthesis and Reactions of 1,3,4-Selenadiazines. *Heterocycles* **2014**, *8* (2), 1397–1431. https://doi.org/10.3987/COM-13-S(S)109.

(1068) Devleshova, N. A.; Lozovskiy, S. V.; Vasilyev, A. V. Reactions of Alkyl 4-Hydroxybut-2-Ynoates with Arenes under Superelectrophilic Activation with Triflic Acid or HUSY Zeolite: Alternative Propargylation or Allenylation of Arenes, and Synthesis of Furan-2-Ones. *Tetrahedron* **2019**, *75* (39), 130517. https://doi.org/10.1016/j.tet.2019.130517.

(1069) Haut, F. L.; Habiger, C.; Speck, K.; Wurst, K.; Mayer, P.; Korber, J. N.; Müller, T.; Magauer, T. Synthetic Entry to Polyfunctionalized Molecules through the [3+2]-Cycloaddition of Thiocarbonyl Ylides. *J. Am. Chem. Soc.* **2019**, *141* (34), 13352–13357. https://doi.org/10.1021/jacs.9b07729.

(1070) Li, J.; Wei, J.; Zhu, B.; Wang, T.; Jiao, N. Cu-Catalyzed Oxygenation of Alkene-Tethered Amides with O2: Via Unactivated CC Bond Cleavage: A Direct Approach to Cyclic Imides. *Chem. Sci.* **2019**, *10* (39), 9099–9103. https://doi.org/10.1039/c9sc03175h.

(1071) Coelho, J. A. S.; Trindade, A. F.; André, V.; Teresa Duarte, M.; Veiros, L. F.; Afonso, C. A. M. Trienamines Derived from 5-Substituted Furfurals: Remote ε-Functionalization of 2,4-Dienals. *Org. Biomol. Chem.* **2014**, *12* (46), 9324–9328. https://doi.org/10.1039/c4ob01759e.

(1072) Chen, T. Y.; Tsutsumi, R.; Montgomery, T. P.; Volchkov, I.; Krische, M. J. Ruthenium-Catalyzed C-C Coupling of Amino Alcohols with Dienes via Transfer Hydrogenation: Redox-Triggered Imine Addition and Related Hydroaminoalkylations. *J. Am. Chem. Soc.* **2015**, *137* (5), 1798–1801. https://doi.org/10.1021/ja5130258.

(1073) Martin, S. E.; Streeter, M. D.; Jones, L. L.; Klepfer, M. S.; Atmatzidis, K.; Wille, K. D.; Harrison, S. A.; Hoegg, E. D.; Sheridan, H. M.; Kramer, S.; Parrish, D. A.; Amick, A. W. A Three-Step Process to Facilitate the Annulation of Polycyclic Aromatic Hydrocarbons. *J. Org. Chem.* **2014**, *79* (17), 8324–8330. https://doi.org/10.1021/jo501576e.

(1074) He, L.; Qin, H. M.; Chen, L. M. 3-(2-Bromophenyl)-N-Phenyloxirane-2-Carboxamide. *Acta Cryst. E* **2009**, *65* (12), o2999–o2999. https://doi.org/10.1107/S1600536809045929.

(1075) Shi, H. 4-(4-Methoxyphenyl)-7,7-Dimethyl-5-Oxo-5,6,7,8-Tetrahydrochromene-2,5-Dione. *Acta Cryst. E* **2009**, *65* (12), o3216–o3216. https://doi.org/10.1107/S160053680904971X.

(1076) Quikert, G.; Scherer, S.; Reichert, D.; Nestler, H. P.; Wennemers, H.; Ebel, A.; Urbahns, K.; Wagner, K.; Michaelis, K. P.; Wiech, G.; Prescher, G.; Bronstert, B.; Freitag, B. J.; Wicke, I.; Lisch, D.; Belik, P.; Crecelius, T.; Hörstermann, D.; Zimmermann, G.; Bats, J. W.; Dürner, G.; Rehm, D. 120. Stereoselective Ring Opening of Electronically Excited Cyclohexa-2,4-Dienones: Cause and Effect. *Helv. Chim. Acta* **1997**, *80* (6), 1683–1772. https://doi.org/10.1002/hlca.19970800602.

(1077) Olszewska, T.; Gdaniec, M.; Połoński, T. Molecular Geometry and Optical Activity of N-Nitroso-2,2,6,6-Tetramethylpiperidines Generated by Spontaneous Crystallization and Inclusion Complexation with Optically Active Diols. *Tetrahedron Asymmetry* **2009**, *20* (11), 1308–1313. https://doi.org/10.1016/j.tetasy.2009.05.008.

(1078) Guspanová, J.; Knecht, R.; Laganà, M.; Weymuth, C.; Hansen, H.-J. Synthesis of Benzo[a]Heptalene . *Helv. Chim. Acta* **1997**, *80* (5), 1375–1407. https://doi.org/10.1002/hlca.19970800506.

(1079) Mackewitz, T. W.; Peters, C.; Bergsträsser, U.; Leininger, S.; Regitz, M. Phosphaalkenes as Building Blocks in Ene Reactions: Synthesis and Reactivity of 3-Amino-1,2-Dihydro-1,2-Diphosphetes. *J. Org. Chem.* **1997**, *62* (22), 7605–7613. https://doi.org/10.1021/jo9707186.

(1080) Kavuru, P.; Aboarayes, D.; Arora, K. K.; Clarke, H. D.; Kennedy, A.; Marshall, L.; Ong, T. T.; Perman, J.; Pujari, T.; Wojtas, Ł.; Zaworotko, M. J. Hierarchy of Supramolecular Synthons: Persistent Hydrogen Bonds between Carboxylates and Weakly Acidic Hydroxyl Moieties in Cocrystals of Zwitterions. *Cryst. Growth Des.* **2010**, *10* (8), 3568–3584. https://doi.org/10.1021/cg100484a.

(1081) Mackay, M. F.; Banwell, M. G.; Pallich, S.; Phyland, J. R. Novel Products from the Rearrangement of Some [n.3.1]Propellanes. *Acta Cryst. C* **1997**, *53* (12), 2000–2002. https://doi.org/10.1107/S0108270197010044.

(1082) Zhao, H. W.; Chen, X. Q.; Yang, Z.; Tian, T.; Li, B.; Meng, W.; Song, X. Q.; Pang, H. L. Highly Diastereoselective Synthesis of Imidazolidine-Dispirooxindoles via Three-Component [3 + 2] Cycloadditions of Isatins, 2-(Aminomethyl)Pyridine and Isatin-Based Imines. *RSC Adv.* **2015**, *5* (125), 103116–103122. https://doi.org/10.1039/c5ra21995g.

(1083) Liu, D. L.; Li, C.; Tian, X.; Li, S.; Xiao, T. 1-(4-Fluorophenyl)-2-(1H-Imidazol-1-Yl)-Ethanol. *Acta Cryst. E* **2012**, *68* (1), o152–o152. https://doi.org/10.1107/S1600536811053505.

(1084) Yao, T.; Liu, T.; Zhang, C. Palladium-Catalyzed Domino Heck/Intermolecular Cross-Coupling: Efficient Synthesis of 4-Alkylated Isoquinoline Derivatives. *Chem. Commun.* **2017**, *53* (15), 2386–2389. https://doi.org/10.1039/c6cc10075a.

(1085) Yeh, M. C. P.; Jou, C. F.; Yeh, W. T.; Chiu, D. Y.; Reddy, N. R. K. Intramolecular 1,3-Dipolar Cycloaddition of Cyclo-1,3-Diene-Tethered Nitrile Oxides. *Tetrahedron* **2005**, *61* (2), 493–500. https://doi.org/10.1016/j.tet.2004.10.078.

(1086) Serelis, A. K.; Solomon, D. H.; Steel, P. J. Stereospecificity in the Geminate Recombination of 1, 3-Diphenylpropyl Radical Pairs. *Aust. J. Chem.* **1989**, *42* (3), 395–404. https://doi.org/10.1071/CH9890395.

(1087) Hans, J. K.; Defoin, R.; Gollnick, K.; Krüger, C.; Yi-Hung, T.; Ling-Kang, L.; Betz, P. Sensitized Photocyclodimerization of Aβ-Unsaturated Cyclic Sulfones. Crystal Structural Analyses of the Photodimers of 2-Sulfolene and Thia-2-Cyclohexene-1,1-Dioxide. *Tetrahedron* **1989**, *45* (6), 1667–1677. https://doi.org/10.1016/S0040-4020(01)80031-4.

(1088) Guo, H.; Gao, Y. Bin; Han, J.; Meng, J. Ben. 8-Bromo-6-Chloro-1′,3′,3′, 5′-Tetramethyl-Spiro[2H-1-Benzopyran-2,2′-Indoline]. *Acta Cryst. E* **2005**, *61* (5), o1461–o1462. https://doi.org/10.1107/S1600536805011864.

(1089) Galeta, J.; Man, S.; Valoušková, A.; Potáček, M. Homoallenyl Azines in Criss-Cross Cycloaddition Reactions. *Monatshefte fur Chemie* **2013**, *144* (2), 205–216. https://doi.org/10.1007/s00706-012-0865-7.

(1090) Shi, D.; Rong, L.; Shi, C.; Zhuang, Q.; Wang, X.; Tu, S.; Hu, H. Low-Valent Titanium Reagent-Promoted Intramolecular Reductive Coupling Reactions of Ketomalononitriles: A Facile Synthesis of Benzo[4,5]Indene, Acridine and Quinoline Derivatives. *Synthesis* **2005**, *2005* (5), 717–724. https://doi.org/10.1055/s-2005-861820.

(1091) Lu, B. L.; Shi, M. Synthesis of Functionalized Polycyclic Compounds: Rhodium(I)-Catalyzed Intramolecular Cycloaddition of Yne and Ene Vinylidenecyclopropanes. *Angew. Chem. Int. Ed.* **2011**, *50* (50), 12027–12031. https://doi.org/10.1002/anie.201105292.

(1092) Gao, F.; Stamp, C. T. M.; Thornton, P. D.; Cameron, T. S.; Doyle, L. E.; Miller, D. O.; Burnell, D. J. Selective Formation of Angular Tricyclic Compounds by Ruthenium-Mediated Ring-Rearrangement Metathesis. *Chem. Commun.* **2012**, *48* (2), 233–235. https://doi.org/10.1039/c1cc15452d.

(1093) Yadav, V. K.; Senthil, G.; Singh, L.; Parvez, M. On the Reaction of 1-Oxa-4-Thiaspiro[4.5]Decan-7-One with PhLi. A Reinvestigation. *J. Org. Chem.* **2004**, *69* (23), 8131–8132. https://doi.org/10.1021/jo0489364.

(1094) Zimmerman, H. E.; St Clair, J. D. Electronic And Steric Control of α- Versus β-Naphthyl Migratory Aptitudes In Enone Photochemistry. Mechanistic and Exploratory Organic Photochemistry. *J. Org. Chem.* **1989**, *54* (9), 2125–2137. https://doi.org/10.1021/jo00270a022.

(1095) Yeh, M. C. P.; Fang, C. W.; Lin, H. H. Facile Synthesis of Azaspirocycles via Iron Trichloride-Promoted Cyclization/Chlorination of Cyclic 8-Aryl-5-Aza-5-Tosyl-2-En-7-Yn-1-Ols. *Org. Lett.* **2012**, *14* (7), 1830–1833. https://doi.org/10.1021/ol300434m.

(1096) Gouygou, M.; Bellan, J.; Escudie, J.; Couret, C.; Dubourg, A.; Declercq, J. P.; Koenig, M. From Unsymmetrical Diphosphene to New 1,3-Diphosphapropenes. *J. Chem. Soc. Chem. Commun.* **1989**, No. 9, 593–595. https://doi.org/10.1039/C39890000593.

(1097) Xu, X.; Hu, W. H.; Zavalij, P. Y.; Doyle, M. P. Divergent Outcomes of Carbene Transfer Reactions from Dirhodium- and Copper-Based Catalysts Separately or in Combination. *Angew. Chem. Int. Ed.* **2011**, *50* (47), 11152–11155. https://doi.org/10.1002/anie.201105557.

(1098) Shen, C. C.; Paquette, L. A. Impact of a Basal Nitro Group on the Density Characteristics of Select [4]Peristylane Derivatives. *J. Org. Chem.* **1989**, *54* (14), 3324–3328. https://doi.org/10.1021/jo00275a016.

(1099) Kwon, B. M.; Foote, C. S.; Khan, S. I. Chemistry of Singlet Oxygen. 52. Reaction with Trans-Stilbene. *J. Org. Chem.* **1989**, *54* (14), 3378–3382. https://doi.org/10.1021/jo00275a024.

(1100) Li, B.; Zhang, M.; Huang, X.; Gu, Z. Synthesis of 2,3-Dihydro-1: H -Phosphindole-1-Oxides via the t -BuLi-Mediated Rearrangement of Vinyl Bromides and Phosphine Oxides. *Org. Chem. Front.* **2017**, *4* (9), 1854–1857. https://doi.org/10.1039/c7qo00310b.

(1101) Harwood, L. M.; Jones, G.; Pickard, J.; Thomas, R. M.; Watkin, D. Stereochemical Studies on the Intramolecular Diels Alder Reaction of Furans with Doubly Activated Dienophiles. *Tetrahedron Lett.* **1988**, *29* (45), 5825–5828. https://doi.org/10.1016/S0040-4039(00)82203-0.

(1102) Semmelhack, M. F.; Zhang, N. Stereoselective Formation of Tetrahydrofuran Rings via Intramolecular Alkoxycarbonylation of Hydroxyalkenes. *J. Org. Chem.* **1989**, *54* (19), 4483–4485. https://doi.org/10.1021/jo00280a003.

(1103) Zimmerman, H. E.; Zuraw, M. J. Photochemistry in a Box. Photochemical Reactions of Molecules Entrapped in Crystal Lattices: Mechanistic and Exploratory Organic Photochemistry. *J. Am. Chem. Soc.* **1989**, *111* (20), 7974–7989. https://doi.org/10.1021/ja00202a044.

(1104) Krishnakumar, K.; Franklin, S.; Prabhu, G. V.; Balasubramanian, T. 1,5-Dimethyl-2-Phenyl-4-[Phenyl(Pyridin-2-Ylamino)Methyl]-1H-Pyrazol-3(2H) -One. *Acta Cryst. E* **2012**, *68* (11), o3193–o3193. https://doi.org/10.1107/S1600536812042936.

(1105) Carloni, P.; Greci, L.; Stipa, P.; Alberti, A.; Rizzoli, C.; Sgarabotto, P.; Ugozzoli, F. Competition between Nucleophilic Attack and Electron Transfer in the Reaction of Indoledione Mine N-Oxides with Primary Aromatic Amines. *J. Chem. Soc. Perkin Trans. 2* **1990**, *2* (1), 185–193. https://doi.org/10.1039/p29900000185.

(1106) Kamimura, A.; Takeuchi, R.; Ikeda, K.; Moriyama, T.; Sumimoto, M. Stereoselective Synthesis of 1-Nitrobicyclo[3.1.0]Hexanes and Fused Isoxazoline-N-Oxides from Primary Nitro Compounds. *J. Org. Chem.* **2012**, *77* (5), 2236–2245. https://doi.org/10.1021/jo202489v.

(1107) Fan, X. W.; Cheng, Y. NHC Brønsted Base-Catalyzed Transformations of Isochromene Derivatives: Regulation of Products by the Structures of Carbene Catalysts. *Org. Biomol. Chem.* **2012**, *10* (45), 9079–9084.

https://doi.org/10.1039/c2ob26622a.

(1108) Billings, S. B.; Woerpel, K. A. Nucleophilic Substitution Reactions of Sulfur-Substituted Cyclohexanone Acetals: An Analysis of the Factors Controlling Stereoselectivity. *J. Org. Chem.* **2006**, *71* (14), 5171–5178. https://doi.org/10.1021/jo060077r.

(1109) Karai, O.; Aouine, Y.; Faraj, H.; Alami, A.; El Hallaoui, A.; Zouihri, H. Methyl (1-Benzamido-2-Methoxy-2-Oxoethyl)Tryptophanate. *IUCrData* **2017**, *2* (10), x171547-. https://doi.org/10.1107/s2414314617015474.

(1110) Farndon, J. J.; Ma, X.; Bower, J. F. Transition Metal Free C-N Bond Forming Dearomatizations and Aryl C-H Aminations by in Situ Release of a Hydroxylamine-Based Aminating Agent. *J. Am. Chem. Soc.* **2017**, *139* (40), 14005–14008. https://doi.org/10.1021/jacs.7b07830.

(1111) Sun, H.; Han, J.; Kattamuri, P. V.; Pan, Y.; Li, G. Approach to Vicinal T-Boc-Amino Dibromides via Catalytic Aminobromination of Nitrostyrenes without Using Chromatography and Recrystallization. *J. Org. Chem.* **2013**, *78* (3), 1171–1175. https://doi.org/10.1021/jo302727v.

(1112) Yin, Z. G.; Xu, P. Y.; Qian, H. Y.; Zhou, N.; Liu, S. M. 5-(2-Furyl)-3-Methyl-1-(4-Nitrophenyl)-2-Pyrazoline. *Acta Cryst. E* **2006**, *62* (12), o5508–o5509. https://doi.org/10.1107/S1600536806046782.

(1113) Regitz, M.; Michels, G. Ein Cyclobutadiencarboxylat Als Hetero-1,3-dien Bei Diels-Alder-Reaktionen Mit 2H-Azirinen. *Chem. Ber.* **1990**, *123* (4), 927–933. https://doi.org/10.1002/cber.19901230444.

(1114) Parmar, N. J.; Teraiya, S. B.; Barad, H. A.; Sharma, D.; Gupta, V. K. Efficient One-Pot Synthesis of Precursors of Some Novel Aminochromene Annulated Heterocycles via Domino Knoevenagel-Hetero-Diels-Alder Reaction. *Synth. Commun.* **2013**, *43* (11), 1577–1586. https://doi.org/10.1080/00397911.2011.652755.

(1115) Kraakman, P. A.; Valk, J. M.; Niederländer, H. A. G.; Brouwer, D. B. E.; Matthias Bickelhaupt, F.; de Wolf, W. H.; Bickelhaupt, F.; Stam, C. H. Unusual Reactivity of Small Cyclophanes: Nucleophilic Attack on 11-Chloro- and 8, 11-Dichloro[5]Metacyclophane. *J. Am. Chem. Soc.* **1990**, *112* (18), 6638–6646. https://doi.org/10.1021/ja00174a028.

(1116) Sun, Z.; Xiang, K.; Tao, H.; Guo, L.; Li, Y. Synthesis of 2-Substituted 3-Chlorobenzofurans via TMSCl-Mediated Nucleophilic Annulation of Isatin-Derived Propargylic Alcohols. *Org. Biomol. Chem.* **2018**, *16* (33), 6133–6139. https://doi.org/10.1039/c8ob01731j.

(1117) Pasechnik, M. P.; Matveeva, A. G.; Lyssenko, K. A.; Aysin, R. R.; Smol'yakov, A. F.; Zubavichus, Y. V.; Godovikov, I. A.; Goryunov, E. I. Competing Intramolecular vs. Intermolecular Hydrogen Bonding in Phosphoryl-Containing Secondary Alkanols: A Structural, Spectroscopic and DFT Study. *J. Mol. Struct.* **2019**, *1175*, 874–881. https://doi.org/10.1016/j.molstruc.2018.08.009.

(1118) Muthusamy, S.; Gangadurai, C. "On Water" Cascade Synthesis of Benzopyranopyrazoles and Their Macrocycles. *Tetrahedron Lett.* **2018**, *59* (15), 1501–1505. https://doi.org/10.1016/j.tetlet.2018.03.013.

(1119) Wagner, B. J.; Doi, J. T.; Musker, W. K. Hydrolysis Rates of Saturated Acyclic and Cyclic Sulfinamides: X-Ray Crystal Structures of an Acyclic Sulfinamide and γ-Ammoniopropanesulfinate. *J. Org. Chem.* **1990**, *55* (24), 5940–5945. https://doi.org/10.1021/jo00311a010.

(1120) Hatano, K.; Takeuchi, I.; Hamada, Y.; Yashiro, T.; Kurono, Y. Conformation of 1-Methyl-3,4-Benzo-7-Thia-2-Azabicyclo[3.3.1]Non-3-Ene 7-Oxide. *Bull. Chem. Soc. Jpn.* **1990**, *63* (10), 3013–3015. https://doi.org/10.1246/bcsj.63.3013.

(1121) Główka, M. L.; Gilli, G.; Bertolasi, V. Structure of 5-Benzoylamino-5-Methyl-4-Oxo-1,3-Dioxane. *J. Crystallogr. Spectrosc. Res.* **1986**, *16* (2), 227–232. https://doi.org/10.1007/BF01161110.

(1122) Müller, J. F. K.; Neuburger, M.; Zehnder, M. Structure and Reactivity of a Sulfoximine-Stabilized Chiral Dilithiocarbanion. *Helv. Chim. Acta* **1997**, *80* (7), 2182–2190. https://doi.org/10.1002/hlca.19970800717.

(1123) Wang, P. Z.; Yu, X. Y.; Li, C. Y.; He, B. Q.; Chen, J. R.; Xiao, W. J. A Photocatalytic Iminyl Radical-Mediated C-C Bond Cleavage/Addition/Cyclization Cascade for the Synthesis of 1,2,3,4-Tetrahydrophenanthrenes. *Chem. Commun.* **2018**, *54* (71), 9925–9928. https://doi.org/10.1039/c8cc06145a.

(1124) Rajesh, M.; Singam, M. K. R.; Puri, S.; Balasubramanian, S.; Sridhar Reddy, M. Nickel Catalyzed Syn-Selective Aryl Nickelation and Cyclization of Aldehyde/Enone-Tethered Terminal Alkynes with Arylboronic Acids. *J. Org. Chem.* **2018**, *83* (24), 15361–15371. https://doi.org/10.1021/acs.joc.8b02618.

(1125) Křižan, M.; Vinklárek, J.; Erben, M.; Růžičková, Z.; Honzíček, J. Iron(II) Complex with Modified Bispidine Ligand: Synthesis and Catalytic Alkyd Drying. *Inorganica Chim. Acta* **2019**, *486*, 636–641. https://doi.org/10.1016/j.ica.2018.11.035.

(1126) Draux, M.; Bernal, I.; Fuchs, R. Conglomerate Crystallization in Organic Compounds II. The Conformation, Configuration, and Spontaneous Resolution of 1-Phenyl-2-Cyano-Cyclopropane. *Struct. Chem.* **1991**, *2* (2), 127–

131. https://doi.org/10.1007/BF00676623.

(1127) Das, P.; Dutta, A.; Bhaumik, A.; Mukhopadhyay, C. Heterogeneous Ditopic ZnFe2O4 Catalyzed Synthesis of 4H-Pyrans: Further Conversion to 1,4-DHPs and Report of Functional Group Interconversion from Amide to Ester. *Green Chem.* **2014**, *16* (3), 1426–1435. https://doi.org/10.1039/c3gc42095g.

(1128) Tan, B.; Bai, L.; Ding, P.; Liu, J.; Wang, Y.; Luan, X. Palladium-Catalyzed Intermolecular [4+1] Spiroannulation by C(Sp 3)−H Activation and Naphthol Dearomatization. *Angew. Chem. Int. Ed.* **2019**, *58* (5), 1474–1478. https://doi.org/10.1002/anie.201813202.

(1129) Cermola, F.; Guaragna, A.; Iesce, M. R.; Palumbo, G.; Purcaro, R.; Rubino, M.; Tuzi, A. New Insight into the Reaction of Singlet Oxygen with Sulfur-Containing Cyclic Alkenes: Dye-Sensitized Photooxygenation of 5,6-Dihydro-1,4-Dithiins. *J. Org. Chem.* **2007**, *72* (26), 10075–10080. https://doi.org/10.1021/jo701983v.

(1130) Day, R. O.; Swamy, K. C. K.; Fairchild, L.; Holmes, J. M.; Holmes, R. R. Influence of Hydrogen Bonding on the Formation of Boat and Chair Conformations of Six-Membered Rings in Spirocyclic Tetraoxyphosphoranes. *J. Am. Chem. Soc.* **1991**, *113* (5), 1627–1635. https://doi.org/10.1021/ja00005a028.

(1131) Chang, M. Y.; Chen, H. Y.; Tsai, Y. L. Intramolecular Benzannulation of 3-Sulfonyl-2-Benzylchromen-4-Ones: Synthesis of Sulfonyl Dibenzooxabicyclo[3.3.1]Nonanes. *J. Org. Chem.* **2019**, *84* (1), 443–449. https://doi.org/10.1021/acs.joc.8b02726.

(1132) Rozek, T.; Tiekink, E. R. T.; Taylor, D. K.; Bowie, J. H. Syntheses of Angucyclinones Related to Ochromycinpne. II.* Regio- and Stereo-Selective Reduction of a Tetrahydroangucyclinone. *Aust. J. Chem.* **1998**, *51* (11), 1057–1060. https://doi.org/10.1071/C98094.

(1133) Cook, S. D.; Hamor, T. A.; Jennings, W. B.; Tebbutt, A. A.; Watson, S. P.; Boyd, D. R. Structure and Bonding in 3,3-Dialkyl-2-Phosphinoyloxaziridines by X-Ray Diffraction and Nuclear Magnetic Resonance Spectroscopy. *J. Chem. Soc. Perkin Trans. 2* **1991**, No. 8, 1281–1285. https://doi.org/10.1039/p29910001281.

(1134) Claeys, D. D.; Moonen, K.; Roman, B. I.; Nemykin, V. N.; Zhdankin, V. V.; Waroquier, M.; Van Speybroeck, V.; Stevens, C. V. Synthesis of Tricyclic Phosphonopyrrolidines via IMDAF: Experimental and Theoretical Investigation of the Observed Stereoselectivity. *J. Org. Chem.* **2008**, *73* (20), 7921–7927. https://doi.org/10.1021/jo801138s.

(1135) Ahmed, S.; Baker, L. A.; Grainger, R. S.; Innocenti, P.; Quevedo, C. E. Thermal Elimination of Diethyldithiocarbamates and Application in the Synthesis of (±)-Ferrugine. *J. Org. Chem.* **2008**, *73* (20), 8116–8119. https://doi.org/10.1021/jo801652x.

(1136) Ohshlta, J.; Ohsaki, H.; Ishikawa, M.; Tachibana, A.; Kurosaki, Y.; Yamabe, T.; Tsukihara, T.; Takahashi, K.; Kiso, Y. Silicon-Carbon Unsaturated Compounds. 29. Photochemical Behavior of 2,6- and 2,7-Bis(Pentamethyldisilanyl)Naphthalene. *Organometallics* **1991**, *10* (8), 2685–2695. https://doi.org/10.1021/om00054a033.

(1137) Donnelly, B. L.; Elliott, L. D.; Willis, C. L.; Booker-Milburn, K. I. Sequential Photochemical and Prins Reactions for the Diastereoselective Synthesis of Tricyclic Scaffolds. *Angew. Chem. Int. Ed.* **2019**, *58* (27), 9095–9098. https://doi.org/10.1002/anie.201903488.

(1138) Jaafar, A.; Alilou, E. H.; Réglier, M.; Waegell, B. Unexpected [2+2+2] MIMIRC Annulation between a Lithium Dienolate and Methyl Acrylate. *Tetrahedron Lett.* **1991**, *32* (40), 5531–5534. https://doi.org/10.1016/0040-4039(91)80076-I.

(1139) Lázár, L.; Fülöp, F.; Bernáth, G.; Kálmán, A.; Argay, G. Synthesis and Stereochemistry of Stereoisomeric 1,3-benzoxazino-1,3- and -3,1-benzoxazines. *J. Heterocycl. Chem.* **1991**, *28* (5), 1213–1218. https://doi.org/10.1002/jhet.5570280510.

(1140) Harmata, M.; Kahraman, M. Lewis Acid Mediated Reactions of N-Arylsulfonimidoyl Chlorides with Alkenes. Some Steric Effects of Alkene Substitution. *J. Org. Chem.* **1998**, *63* (20), 6845–6851. https://doi.org/10.1021/jo980503b.

(1141) George, J.; Ward, J. S.; Sherburn, M. S. A General Synthesis of Dendralenes. *Chem. Sci.* **2019**, *10* (43), 9969–9973. https://doi.org/10.1039/c9sc03976g.

(1142) Wang, H.; Dong, B.; Wang, Y.; Li, J.; Shi, Y. A Palladium-Catalyzed Regioselective Hydroesterification of Alkenylphenols to Lactones with Phenyl Formate as CO Source. *Org. Lett.* **2014**, *16* (1), 186–189. https://doi.org/10.1021/ol403171p.

(1143) Bredikhin, A. A.; Zakharychev, D. V.; Bredikhina, Z. A.; Gubaidullin, A. T.; Fayzullin, R. R. Crystal Structure and Phase Behavior of the Tolyl Glycerol Ethers. from the Conglomerate Former to the Chirality-Driven Nanogelator. *CrystEngComm* **2012**, *14* (1), 211–222. https://doi.org/10.1039/c1ce05637a.

(1144) James, M. N. G.; Matsushima, M. N -Succinopyridine . *Acta Cryst. B* **1976**, *32* (3), 959–961. https://doi.org/10.1107/s0567740876004329.

(1145) Nandi, S.; Singha, R.; Ray, J. K. Palladium Catalyzed Intramolecular Cascade Type Cyclizations: Interesting Approach towards Naphthoquinone Derivatives Having an O-Containing Heterocyclic Skeleton. *Tetrahedron* **2015**, *71* (4), 669–675. https://doi.org/10.1016/j.tet.2014.12.016.

(1146) Devillard, M.; Brousses, R.; Miqueu, K.; Bouhadir, G.; Bourissou, D. A Stable but Highly Reactive Phosphine-Coordinated Borenium: Metal-Free Dihydrogen Activation and Alkyne 1,2-Carboboration. *Angew. Chem. Int. Ed.* **2015**, *54* (19), 5722–5726. https://doi.org/10.1002/anie.201500959.

(1147) Mukhina, O. A.; Kumar, N. N. B.; Cowger, T. M.; Kutateladze, A. G. Photoassisted Diversity-Oriented Synthesis: Accessing 2,6-Epoxyazocane (Oxamorphan) Cores. *J. Org. Chem.* **2014**, *79* (22), 10956–10971. https://doi.org/10.1021/jo5019848.

(1148) Qin, Y.; Zhang, L.; Lv, J.; Luo, S.; Cheng, J. P. Bioinspired Organocatalytic Aerobic C-H Oxidation of Amines with an Ortho -Quinone Catalyst. *Org. Lett.* **2015**, *17* (6), 1469–1472. https://doi.org/10.1021/acs.orglett.5b00351.

(1149) Tu, X. J.; Hao, W. J.; Ye, Q.; Wang, S. S.; Jiang, B.; Li, G.; Tu, S. J. Four-Component Bicyclization Approaches to Skeletally Diverse Pyrazolo[3,4- b]Pyridine Derivatives. *J. Org. Chem.* **2014**, *79* (22), 11110–11118. https://doi.org/10.1021/jo502096t.

(1150) Pati, K.; Gomes, G. D. P.; Harris, T.; Hughes, A.; Phan, H.; Banerjee, T.; Hanson, K.; Alabugin, I. V. Traceless Directing Groups in Radical Cascades: From Oligoalkynes to Fused Helicenes without Tethered Initiators. *J. Am. Chem. Soc.* **2015**, *137* (3), 1165–1180. https://doi.org/10.1021/ja510563d.

(1151) O'Halloran, N.; James, J. P.; Downey, C. A.; O'Malley, P.; Duff, T.; Bertrand, S. Inter-and Intra-Molecular Cyclisation Reactions of Azoacetates Derived from Aryl Hydrazones of Ethyl Acetoacetate and Acetoacetanilides. *Heterocycles* **2008**, *75* (11), 2681–2701. https://doi.org/10.3987/COM-08-11433.

(1152) Lee, K. S.; Zhugralin, A. R.; Hoveyda, A. H. Efficient C-B Bond Formation Promoted by N-Heterocyclic Carbenes: Synthesis of Tertiary and Quaternary B-Substituted Carbons through Metal-Free Catalytic Boron Conjugate Additions to Cyclic and Acyclic α,β-Unsaturated Carbonyls. *J. Am. Chem. Soc.* **2009**, *131* (21), 7253–7255. https://doi.org/10.1021/ja902889s.

(1153) Hayamizu, T.; Maeda, H.; Mizuno, K. Diastereoselective Protonation on Radical Anions of Electron-Deficient Alkenes via Photoinduced Electron Transfer. *J. Org. Chem.* **2004**, *69* (15), 4997–5004. https://doi.org/10.1021/jo0496953.

(1154) Khlebnikov, A. F.; Novikov, M. S.; Pakalnis, V. V.; Yufit, D. S. Nonconcerted Cycloaddition of 2H-Azirines to Acylketenes: A Route to N-Bridgehead Heterocycles. *J. Org. Chem.* **2011**, *76* (22), 9344–9352. https://doi.org/10.1021/jo201563b.

(1155) Zhao, D.; Zhang, J.; Xie, Z. Dearomative [2 + 2] Cycloaddition and Formal C-H Insertion Reaction of o-Carboryne with Indoles: Synthesis of Carborane-Functionalized Heterocycles. *J. Am. Chem. Soc.* **2015**, *137* (29), 9423–9428. https://doi.org/10.1021/jacs.5b05426.

(1156) Chang, M. Y.; Lin, C. H.; Chen, Y. L.; Hsu, R. T.; Chang, C. Y. Pd2(Dba)3-Promoted Synthesis of 3-N-Substituted 4-Aryl-1,2,3,6-Tetrahydropyridine. *Tetrahedron Lett.* **2010**, *51* (37), 4886–4889. https://doi.org/10.1016/j.tetlet.2010.07.043.

(1157) Barsu, N.; Sen, M.; Premkumar, J. R.; Sundararaju, B. Cobalt(Iii) Catalyzed C-8 Selective C-H and C-O Coupling of Quinoline N-Oxide with Internal Alkynes via C-H Activation and Oxygen Atom Transfer. *Chem. Commun.* **2016**, *52* (7), 1338–1341. https://doi.org/10.1039/c5cc08736h.

(1158) Wang, Y.; Zhu, S.; Zhu, G.; Huang, Q. An Efficient Synthesis of 3-Trifluoromethylated 8-Oxabicyclo[3.2.1]Octa-2,6-Dienes. *Tetrahedron* **2001**, *57* (34), 7337–7342. https://doi.org/10.1016/S0040-4020(01)00709-8.

(1159) Yuan, X. L.; Liu, W. Y.; Huo, P.; Mei, G. Q. (Diphenylphosphoryl)(2-Nitrophenyl)Methanol. *Acta Cryst. E* **2010**, *66* (9), o2331–o2331. https://doi.org/10.1107/S160053681003196X.

(1160) Wang, Y.; Cheng, F. X.; Yuan, X. L.; Tang, W. J.; Shi, J. B.; Liao, C. Z.; Liu, X. H. Dihydropyrazole Derivatives as Telomerase Inhibitors: Structure-Based Design, Synthesis, SAR and Anticancer Evaluation in Vitro and in Vivo. *Eur. J. Med. Chem.* **2016**, *112*, 231–251. https://doi.org/10.1016/j.ejmech.2016.02.009.

(1161) Gabbutt, C. D.; Heron, B. M.; Kilner, C.; Kolla, S. B. The Influence of a 1,1-Diarylvinyl Moiety on the Photochromism of Naphthopyrans. *Org. Biomol. Chem.* **2010**, *8* (21), 4874–4883. https://doi.org/10.1039/c0ob00141d.

(1162) Krishna, R.; Selvanayagam, S.; Yogavel, M.; Velmurugan, D.; Shanmugasundaram, M.; Raghunathan, R.; Shanmuga Sundara Raj, S.; Fun, H. K. 1,2,3,4,4a,12a-Hexahydro-2,5,5-Trimethyl-1H-[2]Benzopyrano[3,2-

c]Coumarin. *Acta Cryst. E* **2003**, *59* (6), o808–o809. https://doi.org/10.1107/S1600536803009978.

(1163) Garve, L. K. B.; Petzold, M.; Jones, P. G.; Werz, D. B. [3+3]-Cycloaddition of Donor-Acceptor Cyclopropanes with Nitrile Imines Generated in Situ: Access to Tetrahydropyridazines. *Org. Lett.* **2016**, *18* (3), 564–567. https://doi.org/10.1021/acs.orglett.5b03598.

(1164) Chamas, Z. E. A.; Dietz, O.; Aubert, E.; Fort, Y.; Mamane, V. Synthesis of New Pentacyclic Chromophores through a Highly Regio- and Diastereoselective Cascade Process. *Org. Biomol. Chem.* **2010**, *8* (21), 4815–4818. https://doi.org/10.1039/c0ob00390e.

(1165) Chang, M. Y.; Cheng, Y. C. Synthesis of Substituted Tetralins and Benzosuberans via BF3·OEt2-Mediated Formal (4 + 2) and (5 + 2) Stereocontrolled Cycloaddition of 4-Alkenols with Veratrol. *Org. Lett.* **2016**, *18* (3), 608–611. https://doi.org/10.1021/acs.orglett.5b03696.

(1166) Garcia, J. G.; Reza-Garduño, R. G.; Fronczek, F. R.; McLaughlin, M. L. The Diels–Alder Diadduct of Hexachlorocyclopentadiene with Cyclooctatetraene. *Acta Cryst. C* **1991**, *47* (8), 1771–1774. https://doi.org/10.1107/S0108270191002366.

(1167) Alwarsh, S.; Xu, Y.; Qian, S. Y.; McIntosh, M. C. Radical [1,3] Rearrangements of Breslow Intermediates. *Angew. Chem. Int. Ed.* **2016**, *55* (1), 355–358. https://doi.org/10.1002/anie.201508368.

(1168) Maslak, P.; Narvaez, J. N.; Parvez, M. X-Ray Studies of Sterically Congested Diphenylethane Derivatives. Substituent Effect on Carbon-Carbon Bond Length. *J. Org. Chem.* **1991**, *56* (2), 602–607. https://doi.org/10.1021/jo00002a022.

(1169) Abe, H.; Morishita, T.; Yoshie, T.; Long, K.; Kobayashi, T.; Ito, H. The Total Synthesis of (±)-Naupliolide: A Tetracyclic Sesquiterpene Lactone. *Angew. Chem. Int. Ed.* **2016**, *55* (11), 3795–3798. https://doi.org/10.1002/anie.201600055.

(1170) Cheng, H.; Zeng, F. H.; Yang, X.; Meng, Y. J.; Xu, L.; Wang, F. P. Collective Total Syntheses of Atisane-Type Diterpenes and Atisine-Type Diterpenoid Alkaloids: (±)-Spiramilactone B, (±)-Spiraminol, (±)-Dihydroajaconine, and (±)-Spiramines C and D. *Angew. Chem. Int. Ed.* **2016**, *55* (1), 392–396. https://doi.org/10.1002/anie.201508996.

(1171) Amt, H.; Kliegel, W.; Rettig, S. J.; Trotter, J. Structural Studies of Organoboron Compounds XL. 4,7-Dicyclohexyl-2,2,5,6-Tetraphenyl- 1,3-Dioxa-4-Aza-7-Azonia-2-Boratacycloheptane. *Can. J. Chem.* **1990**, *68* (10), 1797–1802. https://doi.org/10.1139/v90-280.

(1172) Li, S.; Jia, Z.; Nakajima, K.; Kanno, K. I.; Takahashi, T. Dehydro Side Coupling of Substituted Pentacene Derivatives. *J. Org. Chem.* **2011**, *76* (24), 9983–9987. https://doi.org/10.1021/jo201659q.

(1173) He, J. Crystal Structure of 2-Amino-4-(2,3-Dichlorophenyl)-5-Oxo-5,6,7,8-Tetrahydro-4H-Chromene-3-Carbonitrile, C16H12Cl2N2O2. *Zeitschrift fur Krist. New Cryst. Struct.* **2017**, *232* (2), 237–238. https://doi.org/10.1515/ncrs-2016-0221.

(1174) Xu, S.; Chen, R.; Qin, Z.; Wu, G.; He, Z. Divergent Amine-Catalyzed [4+2] Annulation of Morita-Baylis-Hillman Allylic Acetates with Electron-Deficient Alkenes. *Org. Lett.* **2012**, *14* (4), 996–999. https://doi.org/10.1021/ol2032569.

(1175) Schnapp, K. A.; Motz, P. L.; Stoeckel, S. M.; Wilson, R. M.; Krause Bauer, J. A.; Bohne, C. Sequential Multiple-Photon Photochemistry of Sterically Congested Enones. *Tetrahedron Lett.* **1996**, *37* (14), 2317–2320. https://doi.org/10.1016/0040-4039(96)00284-5.

(1176) Camps, P.; Lozano, D.; Guitián, E.; Peña, D.; Pérez, D.; Font-Bardia, M.; Llamas-Saíz, A. L. Straightforward Synthesis of a Vicinal Double-Bridgehead Iodo Trimethylsilyl Octacycle: Unprecedented Lack of Reactivity of the Silyl Group in the Presence of Fluoride Anions. *Eur. J. Org. Chem.* **2017**, *2017* (12), 1594–1603. https://doi.org/10.1002/ejoc.201601618.

(1177) Zhu, S.; Xing, C.; Zhu, S. Stereoselective Preparation of Trifluoromethyl Containing 1,4-Oxathiolane Derivatives through Ring Expansion Reaction of 1,3-Oxathiolanes. *Tetrahedron* **2006**, *62* (5), 829–832. https://doi.org/10.1016/j.tet.2005.10.050.

(1178) Padwa, A.; Battisti, A. Photochemical Transformations in the 9-Heterobicyclo[3.3.1]Nonenone System. *J. Am. Chem. Soc.* **1972**, *94* (2), 521–529. https://doi.org/10.1021/ja00757a033.

(1179) Gümüş, M. K.; Gorobets, N. Y.; Sedash, Y. V.; Shishkina, S. V.; Desenko, S. M. Rapid Formation of Chemical Complexity via a Modified Biginelli Reaction Leading to Dihydrofuran-2(3H)-One Spiro-Derivatives of Triazolo[1,5-a]Pyrimidine. *Tetrahedron Lett.* **2017**, *58* (35), 3446–3448. https://doi.org/10.1016/j.tetlet.2017.07.071.

(1180) Sapi, J.; Dridi, S.; Laronze, J.; Sigaut, F.; Patigny, D.; Laronze, J. Y.; Lévy, J.; Toupet, L. Indole as a Tool in Synthesis.

Indolenine Approach to 4,5-Epoxy-10-Normorphinans. *Tetrahedron* **1996**, *52* (24), 8209–8222. https://doi.org/10.1016/0040-4020(96)00378-X.

(1181) Odabaşoğlu, M.; Büyükgüngör, O. 3-(3-Pyridylamino)Isobenzofuran-1(3H)-One. *Acta Cryst. E* **2006**, *62* (5), o2088–o2089. https://doi.org/10.1107/S1600536806013390.

(1182) Balaraman, K.; Ding, R.; Wolf, C. Stereoselective Synthesis of 3,3'-Bisindolines by Organocatalytic Michael Additions of Fluorooxindole Enolates to Isatylidene Malononitriles in Aqueous Solution. *Adv. Synth. Catal.* **2017**, *359* (23), 4165–4169. https://doi.org/10.1002/adsc.201701107.

(1183) Tornus, I.; Schaumann, E.; Adiwidjaja, G. Novel Reactions of N-Sulfonylamines with 3-Dimethylamino-2H-Azirines. Competitive Formation of 1,2,5-Thiadiazoles, 1,2,3-Oxathiazoles and Acrylamidines. X-Ray Molecular Structure of N-(4-Dimethylamino-5-Methyl-2-Oxo-5-Phenyl-5H-1,2λ 6,3-Oxathiazol-2-Ylide. *J. Chem. Soc. Perkin Trans. 1* **1996**, No. 13, 1629–1633. https://doi.org/10.1039/P19960001629.

(1184) Langer, J.; Gärtner, M.; Görls, H.; Walther, D. Five- and Six-Membered Nickelacyclic Carboxylates as Reagents for the Facile Synthesis of δ-Ketocarboxylic Acids, Isocoumarins, and 1,3-Dicarbonyl Derivatives of Benzoic Acid. *Synthesis* **2006**, *2006* (16), 2697–2706. https://doi.org/10.1055/s-2006-942507.

(1185) Singh, R. S.; Yadav, M.; Gupta, R. K.; Pandey, R.; Pandey, D. S. Luminescent N,O-Chelated Chroman-BF2 Complexes: Structural Variants of BODIPY. *Dalt. Trans.* **2013**, *42* (5), 1696–1707. https://doi.org/10.1039/c2dt31820b.

(1186) Bott, S. G.; Marchand, A. P.; Bolin, J. C.; Wang, Y.; Prasad, A. D.; Xing, D.; Talafuse, L. K.; Watson, W. H.; Bourne, S. A.; Carlson, T. F. Synthesis and Base Promoted Intramolecular Nucleophilic Reaction of 8-Hydroxy-1,2,3,4-Tetrachloro-9,9-Dimethoxy-1α,4α,4aα,6,7,8β,8aα-Hexahydro-1,4-Methanonaphthalene-5(1 H)-One. *J. Chem. Crystallogr.* **1995**, *25* (10), 641–651. https://doi.org/10.1007/BF01665970.

(1187) Quast, H.; Becker, C.; Witzel, M.; Peters, E. M.; Peters, K.; Von Schnering, H. G. Syntheses and Structures of 2,6-Substituted Barbaralanes. *Liebigs Ann.* **1996**, *1996* (6), 985–997. https://doi.org/10.1002/jlac.199619960619.

(1188) Karthikeyan, S. V.; Perumal, S. A Facile Tandem Protocol for the Regioselective Synthesis of Novel Thienobenzothiazepines. *Tetrahedron Lett.* **2007**, *48* (13), 2261–2265. https://doi.org/10.1016/j.tetlet.2007.01.168.

(1189) Gökşen, U. S.; Alpaslan, Y. B.; Kelekçi, N. G.; Işik, Ş.; Ekizoğlu, M. Synthesis, Crystal Structures and Theoretical Calculations of New 1-[2-(5-Chloro-2-Benzoxazolinone-3-Yl)Acetyl]-3,5-Diphenyl-4,5-Dihydro-(1H) -Pyrazoles. *J. Mol. Struct.* **2013**, *1039*, 71–83. https://doi.org/10.1016/j.molstruc.2013.01.066.

(1190) Cameron, A. F.; Cameron, I. R.; Duncanson, F. D. The Structures of the Products of Three Condensation Reactions. Crystal and Molecular Structures of 5,6-Dihydrothiazolo[2,3-c][1,2,4]-Thiadiazol-3-One, 6β-(1-Ethyl-1-Hydroxypropyl)-5α-Phenyl-2,3,5,6-Tetrahydro-Imidazo[2, 1-b]Thiazole, and 5,5-Diphenyl-2,3. *J. Chem. Soc. Perkin Trans. 2* **1981**, No. 5, 789–793. https://doi.org/10.1039/P29810000789.

(1191) Guillaume, S.; Ephritikhine, M. Synthesis and X-Ray Crystal Structure of [U(?-C8H8)(HMPA)3][BPh4], the First Cationic Monocyclooctatetraenyl Compound of Uranium(III), and Its Neodymium Homologue. *Eur. J. Inorg. Chem.* **2001**, 1841–1846. https://doi.org/10.1002/1099-0682.

(1192) Klunder, A. J. H.; Zwanenburg, B.; Liu, Z. Y. A Stereospecific Formal Synthesis of Clavulones from Tricyclo[5.2.1.02,6]Decadienone Epoxides. *Tetrahedron Lett.* **1991**, *32* (26), 3131–3132. https://doi.org/10.1016/0040-4039(91)80708-E.

(1193) Zhu, J.; Klunder, A. J. H.; Smits, J. M. M.; Beurskens, P. T.; Beurskens, G. Crystal and Molecular Structure of Heptacyclo [9,6,1,14,7,114,17,02,10,03,8,0 1,13]-Eicosa-3(8),5,12,15-Tetrene-9, 18-Dione. *J. Chem. Crystallogr.* **1996**, *26* (8), 559–562. https://doi.org/10.1007/BF01668415.

(1194) Dobson, A. J.; Gerkin, R. E. 2-Acetylbenzoic Acid: Phthalide Form. *Acta Cryst. C* **1996**, *52* (12), 3078–3081. https://doi.org/10.1107/S0108270196010414.

(1195) Safaei, E.; Weyhermüller, T.; Bothe, E.; Wieghardt, K.; Chaudhuri, P. A Magnetostructural and Electrochemical Study of CuIII and FeIII Complexes Containing a Tetradentate Aminebis(Phenolate) Ligand with a Pendent Tetrahydrofuran Group. *Eur. J. Inorg. Chem.* **2007**, *2007* (16), 2334–2344. https://doi.org/10.1002/ejic.200700095.

(1196) Petrov, V.; Marshall, W. Hexafluorothioacetone Based Synthesis of Fluorinated Heterocycles. *J. Fluor. Chem.* **2007**, *128* (7), 729–735. https://doi.org/10.1016/j.jfluchem.2007.02.009.

(1197) Nikitin, K.; Müller-Bunz, H.; Ortin, Y.; McGlinchey, M. J. Joining the Rings: The Preparation of 2- and 3-Indenyl-Triptycenes, and Curious Related Processes. *Org. Biomol. Chem.* **2007**, *5* (12), 1952–1960. https://doi.org/10.1039/b703437g.

(1198) Chen, J. Q.; Mi, Y.; Shi, Z. F.; Cao, X. P. Construction of the Tetracyclic Core of (±)-Cycloclavine and 4-Amino Uhle's Ketone. *Org. Biomol. Chem.* **2018**, *16* (20), 3801–3808. https://doi.org/10.1039/c7ob03067c.

(1199) Yeh, M. C. P.; Liang, C. J.; Fan, C. W.; Chiu, W. H.; Lo, J. Y. Synthesis of 2-Azaspiro[4.6]Undec-7-Enes from N - Tosyl- N -(3-Arylpropargyl)-Tethered 3-Methylcyclohex-2-En-1-Ols. *J. Org. Chem.* **2012**, *77* (21), 9707–9717. https://doi.org/10.1021/jo301764g.

(1200) Kerr, W. J.; McLaughlin, M.; Morrison, A. J.; Pauson, P. L. Formal Total Synthesis of (±)-α- and β-Cedrene by Preparation of Cedrone. Construction of the Tricyclic Carbon Skeleton by the Use of a Highly Efficient Intramolecular Khand Annulation. *Org. Lett.* **2001**, *3* (19), 2945–2947. https://doi.org/10.1021/ol016054a.

(1201) Kennedy, A. R.; Kerr, W. J.; McLaughlin, M.; Pauson, P. L. Key Tricyclic Synthetic Intermediates for the Preparation of the Sesquiter-Penes α- and β-Cedrene. *Acta Cryst. C* **2001**, *57* (11), 1316–1318. https://doi.org/10.1107/S0108270101013336.

(1202) Ramirez, T. A.; Wang, Q.; Zhu, Y.; Zheng, H.; Peng, X.; Cornwall, R. G.; Shi, Y. Pd(0)-Catalyzed Sequential C-N Bond Formation via Allylic and Aromatic C-H Amination of α-Methylstyrenes with Diaziridinone. *Org. Lett.* **2013**, *15* (16), 4210–4213. https://doi.org/10.1021/ol401935c.

(1203) Jolivet, S.; Toupet, L.; Texier-Boullet, F.; Hamelin, J. Unusual and Stereoselective Ring Closure of Unsaturated Monohydrazones with β-Ketoesters in a Solvent-Free Reaction. *Tetrahedron* **1996**, *52* (16), 5819–5832. https://doi.org/10.1016/0040-4020(96)00177-9.

(1204) Kashyap, R. P.; Deshpande, M. N.; Rajapaksa, D.; Marchand, A. P.; Watson, W. H. Strained Tridecane Cage Systems. *J. Chem. Crystallogr.* **1995**, *25* (9), 573–578. https://doi.org/10.1007/BF01667026.

(1205) Hilmey, D. G.; Davis, B. D.; Gallucci, J. C.; Brodbelt, J. S.; Paquette, L. A. X-Ray Crystallographic and Mass Spectrometric Probing of the Conformational and Ionophoric Properties of Stereoisomeric Hexatetrahydrofuranylhexane Segments. *J. Org. Chem.* **2007**, *72* (24), 9088–9101. https://doi.org/10.1021/jo7016229.

(1206) Gu, Z.; Ma, S. Studies on Thermal Reactivity of β-(1,2-Allenyl)Butenolides and 2-Allyl-3-Allenylcyclohex-2-Enones. *Chem. Eur. J.* **2008**, *14* (8), 2453–2464. https://doi.org/10.1002/chem.200701171.

(1207) Moorthy, J. N.; Mandal, S.; Mukhopadhyay, A.; Samanta, S. Helicity as a Steric Force: Stabilization and Helicity-Dependent Reversion of Colored o-Quinonoid Intermediates of Helical Chromenes. *J. Am. Chem. Soc.* **2013**, *135* (18), 6872–6884. https://doi.org/10.1021/ja312027c.

(1208) An, X.; Zha, Q.; Wu, Y. Perhydrolysis in Ethereal H 2 O 2 Mediated by MoO 2 (Acac) 2 : Distinct Chemoselectivity between Ketones, Ketals, and Epoxides. *Org. Lett.* **2019**, *21* (5), 1542–1546. https://doi.org/10.1021/acs.orglett.9b00425.

(1209) Rádai, Z.; Kiss, N. Z.; Czugler, M.; Karaghiosoff, K.; Keglevich, G. The Typical Crystal Structures of a Few Representative α-Aryl-α-Hydroxyphosphonates. *Acta Crystallogr. Sect. C Struct. Chem.* **2019**, *75* (3), 283–293. https://doi.org/10.1107/S2053229619001839.

(1210) Satyamurthy, N.; Sivakumar, R.; Ramalingam, K. R.; Berlin, K. D.; Loghry, R. A.; van der Helm, D. Kinetics of Acetylation of Substituted 4-Thianols. Single-Crystal Analysis of Cis-2, Trans-6-Diphenyl-Cis-3-Ethylthian-r-4-Ol and 2,2,6,6-Tetramethyl-4(e)-Phenylthian-4(a)-Ol. *J. Org. Chem.* **1980**, *45* (2), 349–355. https://doi.org/10.1021/jo01290a032.

(1211) Chen, C. N.; Liu, R. S. Gold-Catalyzed [4+2] Annulations of Dienes with Nitrosoarenes as 4 π Donors: Nitroso-Povarov Reactions. *Angew. Chem. Int. Ed.* **2019**, *58* (29), 9831–9835. https://doi.org/10.1002/anie.201903615.

(1212) Kumar, S.; Balkrishna, S. J.; Hodage, A. S.; Kumar, S.; Panini, P. Sensitive and Regenerable Organochalcogen Probes for the Colorimetric Detection of Thiols. *RSC Adv.* **2014**, *4* (23), 11535–11538. https://doi.org/10.1039/c4ra00381k.

(1213) White, J. M.; Riches, A. G.; Cameron, D. W.; Griffiths, P. G. (1A,4Aβ,9Aβ)-4a-Acetoxy-9a-Chloro-1-Methoxy-3-Trimethylsiloxy-1,4,4a,9,9a,10-Hexahydroanthracene-9,10-Dione. *Acta Cryst. C* **1997**, *53* (3), 386–387. https://doi.org/10.1107/S0108270196013595.

(1214) Korzhenko, K. S.; Osipov, D. V.; Osyanin, V. A.; Klimochkin, Y. N. Divergent Pathways for Reactions of 3-Formylchromone with Cyclic Secondary Amines in Alcoholic Media. *SynOpen* **2019**, *3* (4), 164–168. https://doi.org/10.1055/s-0039-1690339.

(1215) Carloni, P.; Greci, L.; Stipa, P.; Cauzzi, D.; Rizzoli, C.; Sgarabotto, P. Nitrogen Configuration Determined by X-Ray Analysis on an Homogeneous Series of 3-Indolinones. *J. Heterocycl. Chem.* **1996**, *33* (1), 81–85. https://doi.org/10.1002/jhet.5570330114.

(1216) Chen, J. R.; Wong, J. B.; Kuo, P. Y.; Yang, D. Y. Synthesis and Characterization of Coumarin-Based Spiropyran

Photochromic Colorants. *Org. Lett.* **2008**, *10* (21), 4823–4826. https://doi.org/10.1021/ol8018902.

(1217) Lévesque, F.; Bélanger, G. A Versatile Cascade of Intramolecular Vilsmeier-Haack and Azomethine Ylide 1,3-Dipolar Cycloaddition toward Tricyclic Cores of Alkaloids. *Org. Lett.* **2008**, *10* (21), 4939–4942. https://doi.org/10.1021/ol802010n.

(1218) Karpiuk, J.; Gawryś, P.; Karpiuk, E.; Suwińska, K. Electron Transfer across a Spiro Link: Extreme Solvatofluorochromism of a Compact Spiro-Bridged: N, N -Dimethylaniline-Phthalide Dyad. *Chem. Commun.* **2019**, *55* (58), 8414–8417. https://doi.org/10.1039/c9cc02933h.

(1219) Čejka, J.; Kubík, R.; Kratochvíl, B.; Kuthan, J. Sterically Crowded Heterocycles. VI. Relative Configuration of Diastereoisomeric (Z)-1-3-Diphenyl-3-(5-Methyl-2-Phenylimidazo[1,2-α]Pyridin-3-Yl)Prop-2-En- 1-Ols. *Collect. Czechoslov. Chem. Commun.* **1996**, *61* (9), 1380–1385. https://doi.org/10.1135/cccc19961380.

(1220) Müller, M.; Förster, W. R.; Holst, A.; Kingma, A. J.; Schaumann, E.; Adiwidjaja, G. Synthesis of 4-Silylcyclobut-2-Enethiones and Their Use in Cyclobutadiene Generation. *Chem. Eur. J.* **1996**, *2* (8), 949–956. https://doi.org/10.1002/chem.19960020809.

(1221) Singh, V.; Das, B.; Jarhad, D. B.; Mobin, S. M. Molecular Diversity from Aromatics. Cycloaddition of Cyclohexa-2,4-Dienones, Ring-Closing Metathesis and Sigmatropic Shifts: A General and Stereoselective Route to Novel Spirocarbocyclics. *Tetrahedron* **2015**, *71* (4), 560–576. https://doi.org/10.1016/j.tet.2014.12.033.

(1222) Ganguly, R.; Sally; Chan, P. W. H.; Stoeckli-Evans, H. Crystal Structure of (Z)-1-Phenyl-3-Styrylundeca-2-En-4, 10-Diyn-1-Ol. *Acta Cryst. E* **2015**, *71* (1), o64. https://doi.org/10.1107/S205698901402742X.

(1223) Higginbotham, M. C. M.; Kennedy, L.; Lindsay, A. G.; Troester, A.; Bebbington, M. W. P. Gold(I)-Catalysed Synthesis of Cyclic Sulfamidates: Current Scope, Stereochemistry and Competing Ene-Allene Cycloisomerisation. *Tetrahedron* **2015**, *71* (4), 727–737. https://doi.org/10.1016/j.tet.2014.11.058.

(1224) Bachi, M. D.; Bar-Ner, N.; Melman, A. Stereoselective Synthesis of (±)-α-Kainic Acid Using Free Radical Key Reactions. *J. Org. Chem.* **1996**, *61* (20), 7116–7124. https://doi.org/10.1021/jo9607875.

(1225) Hoather, H. A.; Raftery, J.; Yalavac, I.; Thomas, E. J. On the Stereoselectivities of Some Hindered Diels-Alder Reactions. *Tetrahedron* **2015**, *71* (24), 4124–4131. https://doi.org/10.1016/j.tet.2015.04.101.

(1226) Lourie, L. F.; Serguchev, Y. A.; Bentya, A. V.; Ponomarenko, M. V.; Rusanov, E. B.; Vovk, M. V.; Fokin, A. A.; Ignat'ev, N. V. Metal Free Electrophilic Fluoro-Cyclization of Unsaturated N-Hydroxy- and N-Acetoxyamides with N-F Reagents. *J. Fluor. Chem.* **2015**, *179*, 42–47. https://doi.org/10.1016/j.jfluchem.2015.04.011.

(1227) Yu, F. C.; Hao, X. P.; Lin, X. R.; Yan, S. J.; Lin, J. Synthesis of Fused Polyhalogeno-7a-Hydroxy-[1,2-a]Indol-5-One Derivatives. *Tetrahedron* **2015**, *71* (24), 4084–4089. https://doi.org/10.1016/j.tet.2015.04.113.

(1228) Black, R. S.; Billing, D. G.; Bartyzel, A.; Cukrowska, E. M. N,N′-Bis[(2-Hydroxy-Phen-Yl)(Phen-Yl)Methyl-Idene]Propane-1,2-Diam Ine. *Acta Cryst. E* **2010**, *66* (6), o1256–o1257. https://doi.org/10.1107/S1600536810015291.

(1229) Samshuddin, S.; Narayana, B.; Yathirajan, H. S.; Safwan, A. P.; Tiekink, E. R. T. 3,5-Bis(4-Bromophenyl)-1-Phenyl-4,5-Dihydro-1H-Pyrazole. *Acta Cryst. E* **2010**, *66* (6), o1279–o1280. https://doi.org/10.1107/S1600536810015795.

(1230) Samshuddin, S.; Narayana, B.; Sarojini, B. K.; Khan, M. T. H.; Yathirajan, H. S.; Raj, C. G. D.; Raghavendra, R. Antimicrobial, Analgesic, DPPH Scavenging Activities and Molecular Docking Study of Some 1,3,5-Triaryl-2-Pyrazolines. *Med. Chem. Res.* **2012**, *21* (8), 2012–2022. https://doi.org/10.1007/s00044-011-9735-9.

(1231) Yan, Y. G.; Tu, G. G.; Wang, L. D.; Liu, J.; Li, S. H. 4-Chloro-N-[3-Methyl-1-(5-Thioxo-4,5-Dihydro-1,3,4-Oxadiazol-2-Yl)but-Yl] Benzamide. *Acta Cryst. E* **2010**, *66* (6), o1381–o1381. https://doi.org/10.1107/S1600536810017368.

(1232) Asaruddin, M. R.; Wahab, H. A.; Mohamed, N.; Goh, J. H.; Fun, H. K. 4-(3,4-Dimethyl-5-Phenyl-1,3-Oxazolidin-2-Yl)-2-Methoxyphenol. *Acta Cryst. E* **2010**, *66* (6), o1452–o1453. https://doi.org/10.1107/S1600536810018891.

(1233) Wu, L.; Shi, M.; Li, Y. BF3·OEt2-Catalyzed Intermolecular Reactions of Yinylidenecyclopropanes with Bis(p-Alkoxyphenyl)Methanols: A Novel Cationic 1,4-Aryl-Migration Process. *Chem. Eur. J.* **2010**, *16* (17), 5163–5172. https://doi.org/10.1002/chem.200903131.

(1234) Rajachan, O. A.; Paul, M.; Yatham, V. R.; Neudörfl, J. M.; Kanokmedhakul, K.; Kanokmedhakul, S.; Berkessel, A. N-Heterocyclic Carbene Catalyzed Tail-to-Tail Oligomerization of N,N-Dimethylacrylamide (DMAA) and the Search for the Stetter Reaction of DMAA with Benzaldehyde. *Tetrahedron Lett.* **2015**, *56* (47), 6537–6540. https://doi.org/10.1016/j.tetlet.2015.09.104.

(1235) Howard, R. H.; Theobald, N.; Bochmann, M.; Wright, J. A. 1-[2-(2,6-Diisopropyl-Anilino)-1-Naph-Th-

Yl]Isoquinoline. *Acta Cryst. C* **2010**, *66* (6), o310–o312. https://doi.org/10.1107/S0108270110018433.

(1236) Yu, X.; Qiu, G.; Liu, J.; Wu, J. Synthesis of 2,3,4,11b-Tetrahydro-1 H-Pyridazino[6,1-a]Isoquinolines via the Three-Component Reaction of 2-Alkynylbenzaldehydes, a Sulfonohydrazide and Dimethyl Cyclopropane-1,1-Dicarboxylate. *Synthesis* **2011**, *2011* (14), 2268–2274. https://doi.org/10.1055/s-0030-1260063.

(1237) Heras, M.; Ventura, M.; Linden, A.; Villalgordo, J. M. Reaction of α-Iminomethylene Amino Esters with Mono- and Bidentate Nucleophiles: A Straightforward Route to 2-Amino-1H-5-Imidazolones. *Tetrahedron* **2001**, *57* (20), 4371–4388. https://doi.org/10.1016/S0040-4020(01)00330-1.

(1238) Stepakov, A. V.; Molchanov, A. P.; Magull, J.; Vidović, D.; Starova, G. L.; Kopf, J.; Kostikov, R. R. The Methoxycarbonylcarbene Insertion into 1,3-Dithiolane and 1,3-Oxathiolane Rings. *Tetrahedron* **2006**, *62* (15), 3610–3618. https://doi.org/10.1016/j.tet.2006.01.100.

(1239) Xie, P.; Wang, L.; Yang, L.; Li, E.; Ma, J.; Huang, Y.; Chen, R. Domino Reaction for the Chemo- and Stereoselective Synthesis of Trans-2,3-Dihydrobenzofurans from N-Thiophosphinyl Imines and Sulfur Ylides. *J. Org. Chem.* **2011**, *76* (19), 7699–7705. https://doi.org/10.1021/jo2008737.

(1240) Zhou, L.; Chen, J.; Zhou, J.; Yeung, Y. Y. N-Bromosuccinimide Promoted One-Pot Synthesis of Guanidine: Scope and Mechanism. *Org. Lett.* **2011**, *13* (21), 5804–5807. https://doi.org/10.1021/ol202402y.

(1241) Escalante, L.; González-Rodríguez, C.; Varela, J. A.; Saá, C. Tandem Brønsted Acid Promoted and Nazarov Carbocyclizations of Enyne Acetals to Hydroazulenones. *Angew. Chem. Int. Ed.* **2012**, *51* (49), 12316–12320. https://doi.org/10.1002/anie.201205823.

(1242) Hergueta, A. R.; López, C.; García-Mera, X.; Fernández, F. Anionic Ring Opening of Norbornenes Fused to Heterocycles. *Tetrahedron* **2004**, *60* (45), 10343–10352. https://doi.org/10.1016/j.tet.2004.08.038.

(1243) Alonso, F.; Meléndez, J.; Yus, M. A Novel Route towards the Synthesis of Spirocyclic Bislactones. *Synlett* **2008**, *2008* (11), 1627–1630. https://doi.org/10.1055/s-2008-1078489.

(1244) Lee, J.; Ko, K. M.; Kim, S. G. Ni(ClO4)2-Catalyzed Friedel–Crafts Reaction of Coumarin-Fused Donor–Acceptor Cyclopropanes with Indoles: Stereoselective Synthesis of Trans-3,4-Disubstituted-3,4-Dihydrocoumarins. *Eur. J. Org. Chem.* **2018**, *2018* (30), 4166–4170. https://doi.org/10.1002/ejoc.201800669.

(1245) Light, M. E.; Murphy, P. J.; Hancock, P.; Hursthouse, M. B. Ethyl 3-(Diphenylphosphinoyl)-3-Phenylpropionate. *Acta Cryst. E* **2002**, *58* (9), o990–o991. https://doi.org/10.1107/s1600536802014642.

(1246) Tsao, J. F.; Thompson, H. W.; Lalancette, R. A. 2,6-Dioxa-3,7-Dioxotricyclo[3.3.3.01,5]Undecane, a Propellanoid Dilactone Representing a New Tricyclic Ring System. *Struct. Chem.* **2002**, *13* (5–6), 455–458. https://doi.org/10.1023/A:1020513504492.

(1247) Lambert, T. H.; MacMillan, D. W. C. Development of a New Lewis Acid-Catalyzed [3,3]-Sigmatropic Rearrangement: The Allenoate-Claisen Rearrangement. *J. Am. Chem. Soc.* **2002**, *124* (46), 13646–13647. https://doi.org/10.1021/ja028090q.

(1248) Liu, B.; Thalji, R. K.; Adams, P. D.; Fronczek, F. R.; McLaughlin, M. L.; Barkley, M. D. Fluorescence of Cis-1-Amino-2-(3-Indolyl)Cyclohexane-1-Carboxylic Acid: A Single Tryptophan X1 Rotamer Model. *J. Am. Chem. Soc.* **2002**, *124* (44), 13329–13338. https://doi.org/10.1021/ja016542d.

(1249) Surov, A. O.; Solanko, K. A.; Bond, A. D.; Bauer-Brandl, A.; Perlovich, G. L. Diversity of Felodipine Solvates: Structure and Physicochemical Properties. *CrystEngComm* **2015**, *17* (22), 4089–4097. https://doi.org/10.1039/c5ce00350d.

(1250) Egli, D. H.; Linden, A.; Heimgartner, H. 1,5-Dipolar Electrocyclizations of Thiocarbonyl Ylides Bearing C=N Groups: Reactions of N-[(Dimethylamino)Methylene]Thiobenzamide and 2-(Dimethylhydrazono) -1-Phenylethane-1-Thione with Diazo Compounds. *Helv. Chim. Acta* **2007**, *90* (1), 86–100. https://doi.org/10.1002/hlca.200790025.

(1251) McCarty, Z. R.; Lastovickova, D. N.; Bielawski, C. W. A Cyclic (Alkyl)(Amido)Carbene: Synthesis, Study and Utility as a Desulfurization Reagent. *Chem. Commun.* **2016**, *52* (31), 5447–5450. https://doi.org/10.1039/c6cc01376g.

(1252) Yoon, U. C.; Jin, Y. X.; Oh, S. W.; Park, C. H.; Park, J. H.; Campana, C. F.; Cai, X.; Duesler, E. N.; Mariano, P. S. A Synthetic Strategy for the Preparation of Cyclic Peptide Mimetics Based on SET-Promoted Photocyclization Processes. *J. Am. Chem. Soc.* **2003**, *125* (35), 10664–10671. https://doi.org/10.1021/ja030297b.

(1253) Chao, W.; Waldman, J. H.; Weinreb, S. M. Studies of Intramolecular Cyclizations of N-Acyliminium Ions Derived from Acyclic Ketones: Unanticipated Stereochemical and Structural Results. *Org. Lett.* **2003**, *5* (16), 2915–2918. https://doi.org/10.1021/ol035046m.

(1254) Shtamburg, V. G.; Shtamburg, V. V.; Anishchenko, A. A.; Zubatyuk, R. I.; Mazepa, A. V.; Klotz, E. A.; Kravchenko, S. V.; Kostyanovsky, R. G. Single-Stage Synthesis of 3-Hydroxy- and 3-Alkoxy-5-Arylimidazolidine-2,4-Diones by

Reaction of Arylglyoxal Hydrates with N-Hydroxy- and N-Alkoxyureas. *Chem. Heterocycl. Compd.* **2015**, *51* (6), 553–559. https://doi.org/10.1007/s10593-015-1735-0.

(1255) Li, Y.; Liu, B.; Song, R. J.; Wang, Q. A.; Li, J. H. Visible Light-Initiated C(Sp3)Br/C(Sp3)H Functionalization of α-Carbonyl Alkyl Bromides through Hydride Radical Shift. *Adv. Synth. Catal.* **2016**, *358* (8), 1219–1228. https://doi.org/10.1002/adsc.201501134.

(1256) Mutoh, K.; Kobayashi, Y.; Hirao, Y.; Kubo, T.; Abe, J. Stealth Fast Photoswitching of Negative Photochromic Naphthalene-Bridged Phenoxyl-Imidazolyl Radical Complexes. *Chem. Commun.* **2016**, *52* (41), 6797–6800. https://doi.org/10.1039/c6cc01534d.

(1257) Selvanayagam, S.; Sridhar, B.; Ravikumar, K.; Saravanan, P.; Raghunathan, R. 1′-Methyl-4′-(1-Naphth-Yl)-1″,2″,3″, 4″-Tetrahydroindane-2-Spiro-2′-Pyrrolidine-3′-Spiro-2″- Naphthalene-1,3,1″-Trione. *Acta Cryst. E* **2011**, *67* (3), o629–o629. https://doi.org/10.1107/S1600536811004880.

(1258) Csatayová, K.; Davies, S. G.; Lee, J. A.; Ling, K. B.; Roberts, P. M.; Russell, A. J.; Thomson, J. E. Syntheses of Trans -SCH-A and Cis -SCH-A via a Stereodivergent Cyclopropanation Protocol. *Org. Lett.* **2010**, *12* (14), 3152–3155. https://doi.org/10.1021/ol101295t.

(1259) Hartung, J.; Kneuer, R.; Laug, S.; Schmidt, P.; Špehar, K.; Svoboda, I.; Fuess, H. A Radical Version of the Bromo- and the Iodocyclization of Bis(Homoallylic) Alcohols - The Synthesis of Halogenated Tetrahydrofurans by Stereoselective Alkoxyl Radical Ring Closures. *Eur. J. Org. Chem.* **2003**, *2003* (20), 4033–4052. https://doi.org/10.1002/ejoc.200300107.

(1260) Kamimura, D.; Nagatomo, M.; Urabe, D.; Inoue, M. Expanding the Scope of Et3B/O2-Mediated Coupling Reactions of O,Te-Acetal. *Tetrahedron* **2016**, *72* (48), 7839–7848. https://doi.org/10.1016/j.tet.2016.04.023.

(1261) Hökelek, T.; Uludağ, N.; Patir, S. N-(2-Benzyloxyethyl)-4,7-Dimethyl-6-(1,3-Dithiolan-2-Yl)-1,2,3,4,5, 6-Hexahydro-1,5-Methano-2-Azocino[4,3-6]-Indol-2-One. *Acta Cryst. E* **2004**, *60* (1), o25–o27. https://doi.org/10.1107/S1600536803027041.

(1262) Liu, D.; Chen, J.; Ai, L.; Zhang, H.; Liu, J. Synthesis of the Putative Structure of (±)-Amarbellisine. *Org. Lett.* **2013**, *15* (2), 410–413. https://doi.org/10.1021/ol3034093.

(1263) Valkonen, A.; Kolehmainen, E.; Zakrzewska, A.; Skotnicka, A.; Gawinecki, R. 2-Methyl-4-Phenyl-3,4-Dihydroquinazoline. *Acta Cryst. E* **2011**, *67* (4), o923–o924. https://doi.org/10.1107/S1600536811009664.

(1264) Powner, M. W.; Sutherland, J. D.; Szostak, J. W. Chemoselective Multicomponent One-Pot Assembly of Purine Precursors in Water. *J. Am. Chem. Soc.* **2010**, *132* (46), 16677–16688. https://doi.org/10.1021/ja108197s.

(1265) Jarhad, D. B.; Singh, V. Π4s + Π2s Cycloaddition of Spiroepoxycyclohexa-2,4-Dienone, Radical Cyclization, and Oxidation-Aldol-Oxidation Cascade: Synthesis of BCDE Ring of Atropurpuran. *J. Org. Chem.* **2016**, *81* (10), 4304–4309. https://doi.org/10.1021/acs.joc.6b00728.

(1266) Uncuţa, C.; Bartha, E.; Gherase, D.; Loas, I. A.; Teodorescu, F.; Varga, R. A.; Vanthuyne, N.; Roussel, C.; Berg, U. Chiral Bicyclo[3.3.1]-3,7-Dioxanonane Derivatives: Study of Crystallization Mode and Conformational Dynamics in Solution. *J. Mol. Struct.* **2011**, *989* (1–3), 20–30. https://doi.org/10.1016/j.molstruc.2010.12.028.

(1267) Ledovskaya, M. S.; Molchanov, A. P.; Kostikov, R. R.; Panikorovsky, T. L.; Gurzhiy, V. V.; Ryazantsev, M. N.; Boitsov, V. M.; Stepakov, A. V. Anthracene-Fused Isoxazolopyrrolo[2,1-a]Isoquinolines via an Endocyclic N-Acyliminium Ion Cyclization: A Joint Experimental and Theoretical Study. *Tetrahedron* **2016**, *72* (32), 4827–4834. https://doi.org/10.1016/j.tet.2016.06.048.

(1268) Fun, H. K.; Hemamalini, M.; Ragavan, R. V.; Vijayakumar, V.; Venkatesh, M. 1,1′-Bis(4-Fluorophenyl)-3,3′-Diisobutyl-4,4′-Diphenoxy- 1H,1′H-4,4′-Bipyrazole-5,5′(4H,4′H)-Dione. *Acta Cryst. E* **2011**, *67* (5), o1036–o1036. https://doi.org/10.1107/S1600536811011664.

(1269) Demircan, A.; Şahin, E.; Beyazova, G.; Karaaslan, M.; Hökelek, T. (3aR,4S,7R,7aS)-2-Phenyl-4-Propyl-3a,4,7,7a-Tetrahydro-1H-4, 7-Epithioisoindole-1,3-Dione 8-Oxide. *Acta Cryst. E* **2011**, *67* (5), o1085–o1086. https://doi.org/10.1107/S1600536811012876.

(1270) Peng, X. X.; Wei, D.; Han, W. J.; Chen, F.; Yu, W.; Han, B. Dioxygen Activation via Cu-Catalyzed Cascade Radical Reaction: An Approach to Isoxazoline/Cyclic Nitrone-Featured α-Ketols. *ACS Catal.* **2017**, *7* (11), 7830–7834. https://doi.org/10.1021/acscatal.7b03078.

(1271) Sánchez-Larios, E.; Holmes, J. M.; Daschner, C. L.; Gravel, M. NHC-Catalyzed Spiro Bis-Indane Formation via Domino Stetter - Aldol - Michael and Stetter - Aldol - Aldol Reactions. *Org. Lett.* **2010**, *12* (24), 5772–5775. https://doi.org/10.1021/ol102685u.

(1272) Sharada, D. S.; Shinde, A. H.; Patel, S. M.; Vidyacharan, S. Scaffold Diversity through a Branching Double-Annulation Cascade Strategy: Iminium-Induced One-Pot Synthesis of Diverse Fused Tetrahydroisoquinoline

Scaffolds. *J. Org. Chem.* **2016**, *81* (15), 6463–6471. https://doi.org/10.1021/acs.joc.6b01096.

(1273) Villuri, B. K.; Kotipalli, T.; Kavala, V.; Ichake, S. S.; Bandi, V.; Kuo, C. W.; Yao, C. F. Synthesis of Spiro Isoindolinone-Indolines and 1,2-Disubstituted Indoles from 2-Iodobenzamide Derivatives. *RSC Adv.* **2016**, *6* (78), 74845–74858. https://doi.org/10.1039/c6ra15002k.

(1274) Cera, G.; Crispino, P.; Monari, M.; Bandini, M. Stereoselective Synthesis of Tetracyclic Indolines via Gold-Catalyzed Cascade Cyclization Reactions. *Chem. Commun.* **2011**, *47* (27), 7803–7805. https://doi.org/10.1039/c1cc12328a.

(1275) Suzuki, H.; Aoyagi, S. Total Synthesis of (±)-Chamobtusin A. *Chem. Commun.* **2011**, *47* (27), 7878–7879. https://doi.org/10.1039/c1cc12267c.

(1276) Zhang, J.; Chan, H. S.; Xie, Z. Selective Cage Boron/Carbon Extrusion Reaction of 13-Vertex Carborane μ-1,2-(CH2)3-1,2-C2B11H 11: Formation of Nido-CB10, Closo-CB10, and Closo-C2B10 Species. *Chem. Commun.* **2011**, *47* (28), 8082–8084. https://doi.org/10.1039/c1cc12023a.

(1277) Turcheniuk, K. V.; Rozhenko, A. B.; Shevchenko, I. V. Synthesis and Some Chemical Properties of a 1, 2λ3σ3-Thiaphosphirane. *Eur. J. Inorg. Chem.* **2011**, *2011* (11), 1762–1767. https://doi.org/10.1002/ejic.201001192.

(1278) Beingessner, R. L.; Farand, J. A.; Barriault, L. Progress toward the Total Synthesis of (±)-Havellockate. *J. Org. Chem.* **2010**, *75* (19), 6337–6346. https://doi.org/10.1021/jo101279z.

(1279) Donadío, L. G.; Galetti, M. A.; Giorgi, G.; Rasparini, M.; Comin, M. J. Anti-Selective Organocatalytic Michael Addition between Phenylacetaldehyde and Nitrostyrene. *J. Org. Chem.* **2016**, *81* (17), 7952–7957. https://doi.org/10.1021/acs.joc.6b01061.

(1280) Al-Jaroudi, Z.; Mohapatra, P. P.; Cameron, T. S.; Jha, A. Expedient and Diastereoselective Synthesis of Substituted 6,6a-Dihydroisoindolo[2,1-a]Quinolin-11(5H)-Ones. *Synthesis* **2016**, *48* (24), 4477–4488. https://doi.org/10.1055/s-0035-1562616.

(1281) El-Hiti, G. A.; Abdel-Wahab, B. F.; Ajarim, M. D.; Alobaid, A. M.; Ghabbour, H. A. Crystal Structure of 2-(3-(Benzofuran-2-Yl)-5-Phenyl-4,5-Dihydro-1H-Pyrazol-1-Yl)-4-Phenylthiazole, C26H19N3OS. *Zeitschrift fur Krist. New Cryst. Struct.* **2016**, *231* (3), 935–936. https://doi.org/10.1515/ncrs-2016-0012.

(1282) Wang, L.; Ma, H. Highly Active Magnesium Initiators for Ring-Opening Polymerization of Rac-Lactide. *Macromolecules* **2010**, *43* (16), 6535–6537. https://doi.org/10.1021/ma101263g.

(1283) Zhang, Y.; Lee, J. H.; Danishefsky, S. J. Antarafacial Mediation of Oxygen Delivery by a Phenylsulfinyl Group in the Epoxidation of Proximal Double Bonds: Intramolecular Trapping of an Early Pummerer Intermediate with Stereoelectronic Control. *J. Am. Chem. Soc.* **2011**, *133* (4), 752–755. https://doi.org/10.1021/ja1107707.

(1284) Khan, A. T.; Das, D. K.; Khan, M. M. Ferric Sulfate [Fe2(SO4)3·xH 2O]: An Efficient Heterogeneous Catalyst for the Synthesis of Tetrahydroquinoline Derivatives Using Povarov Reaction. *Tetrahedron Lett.* **2011**, *52* (35), 4539–4542. https://doi.org/10.1016/j.tetlet.2011.06.080.

(1285) Devi, R. B.; Henrot, M.; De Paolis, M.; Maddaluno, J. Domino Alkylation/Oxa-Michael of 1,3-Cyclohexanediones: Steering the C/O-Chemoselectivity to Reach Tetrahydrobenzofuranones. *Org. Biomol. Chem.* **2011**, *9* (19), 6509–6512. https://doi.org/10.1039/c1ob05923h.

(1286) Yoshida, K.; Nakajima, S.; Ban, Y.; Shibasaki, M.; Ohnuma, T.; Aoe, K.; Date, T. Synthetic Approaches Toward Mitomycins: Construction of p-Quinone Moiety on 1-Benzazocine Derivative. *J. Org. Chem.* **1988**, *53* (22), 5355–5359. https://doi.org/10.1021/jo00257a031.

(1287) Höpfl, H.; Sánchez, M.; Barba, V.; Farfán, N.; Rojas, S.; Santillan, R. Synthesis and Study of Monomeric and Dimeric Boronates by Spectroscopic Methods and X-Ray Crystallography. *Inorg. Chem.* **1998**, *37* (8), 1679–1692. https://doi.org/10.1021/ic970886g.

(1288) Lim, R. K. V.; Lin, Q. Azirine Ligation: Fast and Selective Protein Conjugation via Photoinduced Azirine-Alkene Cycloaddition. *Chem. Commun.* **2010**, *46* (42), 7993–7995. https://doi.org/10.1039/c0cc02863k.

(1289) Jardim, G. A. M.; Cruz, E. H. G.; Valença, W. O.; Resende, J. M.; Rodrigues, B. L.; Ramos, D. F.; Oliveira, R. N.; Silva, P. E. A.; Da Silva Júnior, E. N. On the Search for Potential Antimycobacterial Drugs: Synthesis of Naphthoquinoidal, Phenazinic and 1,2,3-Triazolic Compounds and Evaluation against Mycobacterium Tuberculosis. *J. Braz. Chem. Soc.* **2015**, *26* (5), 1013–1027. https://doi.org/10.5935/0103-5053.20150067.

(1290) Pottie, I. R.; Nandaluru, P. R.; Benoit, W. L.; Miller, D. O.; Dawe, L. N.; Bodwell, G. J. Synthesis of 6 H-Dibenzo[b, d]Pyran-6-Ones Using the Inverse Electron Demand Diels-Alder Reaction. *J. Org. Chem.* **2011**, *76* (21), 9015–9030. https://doi.org/10.1021/jo201775e.

(1291) Chang, M. Y.; Lin, C. H.; Lee, T. W. Regioselective Synthesis of 2-Substituted 3-Diarylmethylenylpiperidines. *Tetrahedron Lett.* **2012**, *53* (6), 627–631. https://doi.org/10.1016/j.tetlet.2011.11.105.

(1292) Puranik, D. B.; Johnson, M. P.; Fink, M. J. The Unusual Dimerization of 1-Mesityl-2,3,4-Tri-t-Butyl-1-Silacyclobutadiene: A Solid State Structure. *J. Chem. Soc. Chem. Commun.* **1989**, No. 11, 706–708. https://doi.org/10.1039/C39890000706.

(1293) Piotrowski, D. W.; Rolph, M.; Wei, L. Substituted Azabicyclo[2.2.1]Heptanes via Nitrenium Ion Rearrangement. *Tetrahedron Lett.* **2012**, *53* (9), 1009–1012. https://doi.org/10.1016/j.tetlet.2011.12.065.

(1294) Bonacorso, H. G.; Cavinatto, S.; Campos, P. T.; Porte, L. M. F.; Navarini, J.; Paim, G. R.; Martins, M. A. P.; Zanatta, N.; Stuker, C. Z. New Trifluoromethyl-Containing (E)-N′-Arylidene-[3-Alkyl(Aryl/ Heteroaryl)-4,5-Dihydro-1H-Pyrazol-1-Yl]Carbohydrazides: Synthesis, Crystal Structure and Antimicrobial/Antioxidant Activity. *J. Fluor. Chem.* **2012**, *135*, 303–314. https://doi.org/10.1016/j.jfluchem.2011.12.010.

(1295) Karpov, V. M.; Platonov, V. E.; Rybalova, T. V.; Gatilov, Y. V.; Shakirov, M. M. Fluorinated Dihydroindeno[2,1-c][1,2,6]Thiadiazines: The First Synthesis, Structural Characterization and Reactivity. *J. Fluor. Chem.* **2012**, *135*, 254–260. https://doi.org/10.1016/j.jfluchem.2011.12.005.

(1296) Lim, C. W.; Tissot, O.; Mattison, A.; Hooper, M. W.; Brown, J. M.; Cowley, A. R.; Hulmes, D. I.; Blacker, A. J. Practical Preparation and Resolution of 1-(2′-Diphenylphosphino-1′-Naphthyl)Isoquinoline: A Useful Ligand for Catalytic Asymmetric Synthesis. *Org. Process Res. Dev.* **2003**, *7* (3), 379–384. https://doi.org/10.1021/op034007n.

(1297) Dandia, A.; Khan, S.; Soni, P.; Indora, A.; Mahawar, D. K.; Pandya, P.; Chauhan, C. S. Diversity-Oriented Sustainable Synthesis of Antimicrobial Spiropyrrolidine/Thiapyrrolizidine Oxindole Derivatives: New Ligands for a Metallo-β-Lactamase from Klebsiella Pneumonia. *Bioorganic Med. Chem. Lett.* **2017**, *27* (13), 2873–2880. https://doi.org/10.1016/j.bmcl.2017.04.083.

(1298) Huang, H. M.; Procter, D. J. Dearomatizing Radical Cyclizations and Cyclization Cascades Triggered by Electron-Transfer Reduction of Amide-Type Carbonyls. *J. Am. Chem. Soc.* **2017**, *139* (4), 1661–1667. https://doi.org/10.1021/jacs.6b12077.

(1299) Hartshorn, M. P.; Robinson, W. T.; Wright, G. J.; Yong, C. L. 15N-Labelling Study of the Rearrangement of r-2, 4-Dichloro-t-6-Hydroxy-3, 6-Dimethyl-2, t-5-Dinitrocyclohex-3-Enone to 4-Chloro-c-6-Hydroxy-3, 6-Dimethyl-r-5-Nitro-Cyclohex-3-Ene-1, 2-Dione. *Aust. J. Chem.* **1989**, *42* (9), 1569–1578. https://doi.org/10.1071/CH9891569.

(1300) Hwang, S. H.; Olmstead, M. M.; Kurth, M. J. Solid-Phase Synthesis of an Isoxazolinopyrrole Library. *J. Comb. Chem.* **2004**, *6* (1), 142–148. https://doi.org/10.1021/cc034023w.

(1301) Takeda, T.; Yoshida, S.; Nishimura, T.; Tsubouchi, A. Highly Diastereoselective Addition of Allyltitanocenes to α-Chiral Ketones. *Tetrahedron Lett.* **2012**, *53* (30), 3930–3933. https://doi.org/10.1016/j.tetlet.2012.05.085.

(1302) Chelghoum, M.; Bahnous, M.; Bouraiou, A.; Bouacida, S.; Belfaitah, A. An Efficient and Rapid Intramolecular Aza-Michael Addition of 2′-Aminochalcones Using Ionic Liquids as Recyclable Reaction Media. *Tetrahedron Lett.* **2012**, *53* (32), 4059–4061. https://doi.org/10.1016/j.tetlet.2012.05.097.

(1303) Chang, M. Y.; Wu, M. H.; Chen, Y. L. Synthesis of Dihydrobenzoimidazo[2,1-a]Isoquinolines. *Tetrahedron Lett.* **2012**, *53* (32), 4156–4160. https://doi.org/10.1016/j.tetlet.2012.05.132.

(1304) Lahiri, S.; Yadav, S.; Chanda, M.; Chakraborty, I.; Chowdhury, K.; Mukherjee, M.; Choudhury, A. R.; Row, T. N. G. Importance of Steric Factors in Face-Selective Cycloadditions: 1,6-Annulated Cyclohexa-1,3-Dienes. *Tetrahedron Lett.* **2005**, *46* (47), 8133–8136. https://doi.org/10.1016/j.tetlet.2005.09.129.

(1305) Yang, J.; Zhang, M.; Wang, P.; Liu, W.; Shen, R. 2-Amino Homopropargyl Alcohols from the Highly Regioselective Ag2CO3-Catalyzed Nucleophilic Openings of Alkynyl Epoxides by Amines. *Chinese Chem. Lett.* **2018**, *29* (3), 524–526. https://doi.org/10.1016/j.cclet.2017.09.001.

(1306) Hartshorn, M. P.; Robinson, W. T.; Grant Waller, A.; Wright, G. J. Reactions of 6-Benzyl-2-t-Butyl-4, 6-Dimethyl-Cyclohexa-2, 4-Dienone and 6-Benzyl-4-t-Butyl-2, 6-Dimethylcyclohexa-2, 4-Dienone with Nitrogen Dioxide. *Aust. J. Chem.* **1989**, *42* (9), 1547–1568. https://doi.org/10.1071/CH9891547.

(1307) Sadeghi-Khomami, A.; Blake, A. J.; Wilson, C.; Thomas, N. R. Synthesis of a Carbasugar Analogue of a Putative Intermediate in the UDP-Galp-Mutase Catalyzed Isomerization. *Org. Lett.* **2005**, *7* (22), 4891–4894. https://doi.org/10.1021/ol0517877.

(1308) Niecke, E.; Streubel, R.; Nieger, M.; Stalke, D. Synthesis and Structure of a 1H-Diphosphirene. *Angew. Chem. Int. Ed.* **1989**, *28* (12), 1673–1674. https://doi.org/10.1002/anie.198916731.

(1309) Abdel-Wahab, B. F.; Abdel-Latif, E.; Mohamed, H. A.; Awad, G. E. A. Design and Synthesis of New 4-Pyrazolin-3-Yl-1,2,3-Triazoles and 1,2,3-Triazol-4-Yl-Pyrazolin-1-Ylthiazoles as Potential Antimicrobial Agents. *Eur. J. Med. Chem.* **2012**, *52*, 263–268. https://doi.org/10.1016/j.ejmech.2012.03.023.

(1310) Sedash, Y. V.; Gorobets, N. Y.; Chebanov, V. A.; Konovalova, I. S.; Shishkin, O. V.; Desenko, S. M. Dotting the i's

in Three-Component Biginelli-like Condensations Using 3-Amino-1,2,4-Triazole as a 1,3-Binucleophile. *RSC Adv.* **2012**, *2* (17), 6719–6728. https://doi.org/10.1039/c2ra20195j.

(1311) Surya Prakash Rao, H.; Vijjapu, S. Chemistry of 9,10-Phenanthrenequinone Revisited: Iron(Iii) Chloride Catalyzed Reactions of 9,10-Phenanthrenequinone with Acyclic and Cyclic Ketones Provide Furan Annulated Products. *RSC Adv.* **2012**, *2* (17), 6773–6783. https://doi.org/10.1039/c2ra20499a.

(1312) Xu, X. S.; Li, Z. W.; Zhang, Y. J.; Peng, X. S.; Wong, H. N. C. Total Synthesis of (±)-Pallambins C and D. *Chem. Commun.* **2012**, *48* (68), 8517–8519. https://doi.org/10.1039/c2cc34310j.

(1313) Giri, S. S.; Liu, R. S. Gold-Catalyzed [4+2]- and [3+3]-Annulations of Ynamides with 1-Yn-3-Ols to Access Six-Membered Carbocycles and Oxacycles via Three Distinct Cyclizations. *Adv. Synth. Catal.* **2017**, *359* (19), 3311–3318. https://doi.org/10.1002/adsc.201700784.

(1314) Slama, J. T.; Satsangi, R. K.; Simmons, A.; Lynch, V.; Bolger, R. E.; Suttie, J. An Approach to Trapping γ-Glutamyl Radical Intermediates Proposed for Vitamin K Dependent Carboxylase: α,β-Methyleneglutamic Acid. *J. Med. Chem.* **1990**, *33* (2), 824–832. https://doi.org/10.1021/jm00164a056.

(1315) Sharma, V.; Gupta, V.; Anthal, S.; Saxena, A. K.; Ishar, M. P. S. Photochemical Formation and Decomposition of 8-[β-Arylethenyl]-2,2,6- Trimethyl-7,9,10-Trioxa-Tricyclo[6.2.2.0 1,6]Dodec-11-Ene to Novel 6-Hydroxy-1,7,7-Trimethyl-2-Oxa-Bicyclo[4.4.0]Dec-4-En-3-One in the Presence of Oxygen. *Tetrahedron Lett.* **2012**, *53* (42), 5649–5651. https://doi.org/10.1016/j.tetlet.2012.08.028.

(1316) Grigg, R.; Duffy, L. M.; Dorrity, M. J.; Malone, J. F.; Rajviroongit, S.; Thornton-Pett, M. X = Y-ZH Systems as Potential 1,3-Dipoles. Part 27 Intramolecular Cycloaddition Reactions of Imines of Cyclic Secondary α-Amino Esters. Dipole and Cycloaddition Stereochemistry. *Tetrahedron* **1990**, *46* (6), 2213–2230. https://doi.org/10.1016/S0040-4020(01)89786-6.

(1317) Ye, F.; Li, J. J.; Xu, Z. Z.; Fu, Y. Crystal Structure of 2-(8a-Methyl-5-Oxo-Hexahydroimidazo [1,2-A]Pyridin-1(5H)-Yl)-2-Oxoethyl Acetate, C12H18N2O4. *Zeitschrift fur Krist. New Cryst. Struct.* **2017**, *232* (5), 791–793. https://doi.org/10.1515/ncrs-2017-0041.

(1318) Döpp, D.; Kruse, C.; Flörke, U.; Henkel, G. Rel-(1 R,4R,9R)-1-Acetyl-9-(1-Piperidinyl)-1,4-Dihydro-1,4-Ethanonaphthalene-9-Carbonitrile. *Acta Cryst. E* **2006**, *62* (9), o3904–o3906. https://doi.org/10.1107/S160053680603203X.

(1319) Yeung, Y. Y.; Gao, X.; Corey, E. J. A General Process for the Haloamidation of Olefins. Scope and Mechanism. *J. Am. Chem. Soc.* **2006**, *128* (30), 9644–9645. https://doi.org/10.1021/ja063675w.

(1320) Bakulina, O.; Ivanov, A.; Suslonov, V.; Darin, D.; Krasavin, M. A Speedy Route to Sterically Encumbered, Benzene-Fused Derivatives of Privileged, Naturally Occurring Hexahydropyrrolo[1,2-b]Isoquinoline. *Beilstein J. Org. Chem.* **2017**, *13* (1), 1413–1424. https://doi.org/10.3762/bjoc.13.138.

(1321) Kostyanovsky, R. G.; Lyssenko, K. A.; Kadorkina, G. K.; Prokopenko, O. R. The First Conglomerate in Fluorinated Asymmetric Nitrogen Compounds. *Mendeleev Commun.* **2006**, *16* (3), 139–141. https://doi.org/10.1070/MC2006v016n03ABEH002366.

(1322) Denmark, S. E.; Dappen, M. S.; Sear, N. L.; Jacobs, R. T. The Vinylogous Anomeric Effect in 3-Alkyl-2-Chlorocyclohexanone Oximes and Oxime Ethers. *J. Am. Chem. Soc.* **1990**, *112* (9), 3466–3474. https://doi.org/10.1021/ja00165a034.

(1323) Zimmerman, H. E.; Nesterov, E. E. Crystal Lattice Photochemistry Often Proceeds in Discrete Stages. Mechanistic and Exploratory Organic Photochemistry. *Org. Lett.* **2000**, *2* (8), 1169–1170. https://doi.org/10.1021/ol0057838.

(1324) Ashmore, J.; Bishop, R.; Craig, D. C.; Scudder, M. L. Inclusion of Polyhalomethanes by a Tetrahalogenated Diquinoline Host. *CrystEngComm* **2006**, *8* (12), 923–930. https://doi.org/10.1039/b613537d.

(1325) Lu, B. L.; Wei, Y.; Shi, M. Rhodium(I)-Catalyzed Pauson-Khand-Type [3 + 2 + 1] Cycloaddition Reaction of Ene-Vinylidenecyclopropanes and CO: A Highly Regio- and Stereoselective Synthetic Approach for the Preparation of Aza- and Oxa-Bicyclic Compounds. *Organometallics* **2012**, *31* (12), 4601–4609. https://doi.org/10.1021/om3004288.

(1326) Lopes, L. D.; Bortoluzzi, A. J.; Prampolini, G.; dos Santos, F. P.; Livotto, P. R.; Merlo, A. A. Structural and Morphological Aspects of Small 3,5-Disubstituted Isoxazoles. *J. Fluor. Chem.* **2018**, *211*, 24–36. https://doi.org/10.1016/j.jfluchem.2018.04.007.

(1327) Gupta, A. K.; Ahamad, S.; Vaishanv, N. K.; Kant, R.; Mohanan, K. Base-Mediated 1,6-Conjugate Addition of the Seyferth-Gilbert Reagent to: Para -Quinone Methides. *Org. Biomol. Chem.* **2018**, *16* (25), 4623–4627. https://doi.org/10.1039/c8ob01017j.

(1328) Rodier, F.; Parrain, J. L.; Chouraqui, G.; Commeiras, L. First Studies Directed towards the Diastereoselective Synthesis of the BCD Tricyclic Core of Brownin F. *Org. Biomol. Chem.* **2013**, *11* (25), 4178–4185. https://doi.org/10.1039/c3ob40363g.

(1329) Odabasoğlu, M.; Büyükgüngör, O. 3-(Dimethylamino)Isobenzofuran-1(3H)-One. *Acta Cryst. E* **2007**, *63* (4), o1560–o1561. https://doi.org/10.1107/S1600536807009415.

(1330) Aulenta, F.; Berndt, M.; Brüdgam, I.; Hartl, H.; Sörgel, S.; Reißig, H. U. A New, Efficient and Stereoselective Synthesis of Tricyclic and Tetracyclic Compounds by Samarium Diiodide Induced Cyclisations of Naphthyl-Substituted Arylketones-an Easy Access to Steroid-like Skeletons. *Chem. Eur. J.* **2007**, *13* (21), 6047–6062. https://doi.org/10.1002/chem.200700057.

(1331) Pirovano, V.; Dell'Acqua, M.; Facoetti, D.; Nava, D.; Rizzato, S.; Abbiati, G.; Rossi, E. Cycloaddition versus Alkylation Reactions of 2-Vinylindoles with α,β-Unsaturated Carbonyl Compounds under Gold Catalysis. *Eur. J. Org. Chem.* **2013**, *2013* (28), 6267–6279. https://doi.org/10.1002/ejoc.201300725.

(1332) Craig, D.; Fischer, D. A.; Kemal, Ö.; Marsh, A.; Plessner, T.; Slawin, A. M. Z.; Williams, D. J. Intramolecular Diels-Alder Reactions of Sulphonyl-Substituted Trienes. *Tetrahedron* **1991**, *47* (18–19), 3095–3128. https://doi.org/10.1016/S0040-4020(01)96038-7.

(1333) Grunewald, G. L.; Palanki, M. S.; Takusagawa, F. Structures of Two Conformationally Defined Phenylethanolamines: Exo-1,4-Epoxy-2-Formamido-1,2,3,4-Tetrahydro-8-Trifluoromethylnaphthale Ne and Exo-1,4-Epoxy-2-Formamido-1,2,3,4-Tetrahydro-6-Trifluoromethylnaphthale Ne. *Acta Crystallogr. C.* **1991**, *47 (Pt 4)* (4), 771–775. https://doi.org/10.1107/s0108270190007284.

(1334) Mukovoz, P. P.; Slepukhin, P. A.; Danilova, E. A.; Aysuvakova, O. P.; Glinushkin, A. P. Synthesis, Structure, and Biological Activity of Products of Reactions of 3,4-Dioxohexane-1,6-Dioic Acid Esters with 2-Aminophenol. *Russ. J. Gen. Chem.* **2018**, *88* (7), 1363–1368. https://doi.org/10.1134/S1070363218070022.

(1335) Vakhula, A. R.; Horak, Y. I.; Lytvyn, R. Z.; Lesyuk, A. I.; Kinzhybalo, V.; Zubkov, F. I.; Obushak, M. D. 5-Aryl-2-Furaldehydes in the Synthesis of Tetrahydropyrimidinones by Biginelli Reaction. *Chem. Heterocycl. Compd.* **2018**, *54* (5), 545–549. https://doi.org/10.1007/s10593-018-2301-3.

(1336) Peng, L. jun; Liu, Z. quan; Wang, J. tao; Wu, L. min. N-Nitrosation of (E)-2-(Benzylidene-Amino)Ethanols. *Tetrahedron Lett.* **2007**, *48* (41), 7418–7421. https://doi.org/10.1016/j.tetlet.2007.05.178.

(1337) Eipert, M.; Maichle-Mössmer, C.; Maier, M. E. Use of Epoxidation and Epoxide Opening Reactions for the Synthesis of Highly Functionalized 1-Oxaspiro[4.5]Decan-2-Ones and Related Compounds. *Tetrahedron* **2003**, *59* (40), 7949–7960. https://doi.org/10.1016/j.tet.2003.08.010.

(1338) Ruider, S. A.; Müller, S.; Carreira, E. M. Ring Expansion of 3-Oxetanone-Derived Spirocycles: Facile Synthesis of Saturated Nitrogen Heterocycles. *Angew. Chem. Int. Ed.* **2013**, *52* (45), 11908–11911. https://doi.org/10.1002/anie.201306563.

(1339) Peng, Y.; Luo, Z. B.; Zhang, J. J.; Luo, L.; Wang, Y. W. Collective Synthesis of Several 2,7'-Cyclolignans and Their Correlation by Chemical Transformations. *Org. Biomol. Chem.* **2013**, *11* (43), 7574–7586. https://doi.org/10.1039/c3ob41672k.

(1340) Zhao, B.; Yuan, W.; Du, H.; Shi, Y. Cu(I)-Catalyzed Intermodular Diamination of Activated Terminal Olefins. *Org. Lett.* **2007**, *9* (24), 4943–4945. https://doi.org/10.1021/ol702061s.

(1341) Su, S.; Porco, J. A. 1,2-Dihydroisoquinolines as Templates for Cascade Reactions to Access Isoquinoline Alkaloid Frameworks. *Org. Lett.* **2007**, *9* (24), 4983–4986. https://doi.org/10.1021/ol702176h.

(1342) Calatayud, D. G.; Escolar, F. J.; López-Torres, E.; Mendiola, M. A. Facile and Selective Synthesis of 4-Methyl- and 4-Phenylthiosemicarbazide (= N-Methyl- and N-Phenylhydrazinecarbothioamide) Derivatives of Benzil (= 1,2-Diphenylethane-1,2-Dione). *Helv. Chim. Acta* **2007**, *90* (11), 2201–2216. https://doi.org/10.1002/hlca.200790228.

(1343) Boobalan, R.; Santhoshkumar, R.; Cheng, C. H. Co(III)-Catalyzed [4+1] Annulation of Amides with Allenes via C−H Activation. *Adv. Synth. Catal.* **2019**, *361* (5), 1140–1145. https://doi.org/10.1002/adsc.201801335.

(1344) Gianatassio, R.; Kadish, D. Direct Alkylation of 1-Azabicyclo[1.1.0]Butanes. *Org. Lett.* **2019**, *21* (7), 2060–2063. https://doi.org/10.1021/acs.orglett.9b00321.

(1345) Yang, Y. Y.; Yang, G.; Cheng, C.; Li, Y. X.; Zhang, J. Q.; Feng, W.; Zhao, Y. L.; Tang, L. Catalyst-Free Cleavage of Amide and C-O Double Bond for the Diastereoselective Synthesis of Trifluoromethyl-Containing Dihydrooxazole Derivatives. *Org. Lett.* **2019**, *21* (7), 2236–2240. https://doi.org/10.1021/acs.orglett.9b00522.

(1346) Gammill, R. B.; Nash, S. A.; Bell, L. T.; Watt, W.; Mizsak, S. A.; Scahill, T. A.; Sobieray, D. Mechanistic and Stereochemical Aspects of the 1,2-3,4 Hydride Reduction of Enones. *Tetrahedron Lett.* **1990**, *31* (37), 5303–

5306. https://doi.org/10.1016/S0040-4039(00)98056-0.

(1347) Pestana, D. C.; Power, P. P. Nature of the Boron-Phosphorus Bond in Monomeric Phosphinoboranes and Related Compounds. *J. Am. Chem. Soc.* **1991**, *113* (22), 8426–8437. https://doi.org/10.1021/ja00022a034.

(1348) Kliegel, W.; Lubkowitz, G.; Rettig, S. J.; Trotter, J. Structural Studies of Organoboron Compounds. XLIX. 4,6-Bis(1-Phenyl-2-Nitroethyl)-2-(4-Methoxyphenyl)-1,3-Dioxa-4,6-Diaza-2-Boracyclohexane. *Can. J. Chem.* **1991**, *69* (8), 1227–1232. https://doi.org/10.1139/v91-183.

(1349) Nesi, R.; Giomi, D.; Papaleo, S.; Turchi, S.; Dapporto, P.; Paoli, P. Reaction of 1-Diethylaminopropyne with Difunctionalized 4-Nitroisoxazoles: A Single Step Access to the Novel Isoxazolo[4,5-c]Isoxazole System. *Tetrahedron Lett.* **1991**, *32* (43), 6223–6226. https://doi.org/10.1016/0040-4039(91)80794-7.

(1350) Kumaraswamy, G.; Gangadhar, M.; Ramesh, V.; Ankamma, K.; Sridhar, B. Cationic Pd(IV)-Induced Highly Diastereoselective Arylative Cascade Cyclization of Allene-Tethered Cyclohexadienones Leading to Oxygenated Bicyclic Motifs. *Org. Lett.* **2019**, *21* (16), 6300–6304. https://doi.org/10.1021/acs.orglett.9b02180.

(1351) Grigg, R.; Santhakumar, V.; Sridharan, V.; Stevenson, P.; Teasdale, A.; Thornton-Pett, M.; Worakun, T. The Synthesis of Bridged-Ring Carbo- and Hetero-Cycles via Palladium Catalysed Regiospecific Cyclisation Reactions. *Tetrahedron* **1991**, *47* (46), 9703–9720. https://doi.org/10.1016/S0040-4020(01)91034-8.

(1352) Wang, M. Z.; Zhou, C. Y.; Che, C. M. A Silver-Promoted Auto-Tandem Catalysis for the Synthesis of Multiply Substituted Tetrahydrocarbazoles. *Chem. Commun.* **2011**, *47* (4), 1312–1314. https://doi.org/10.1039/c0cc04383d.

(1353) Chang, Z.; Guillot, R.; Boddaert, T.; Aitken, D. J. Stereocontrolled Preparation of Diversely Trifunctionalized Cyclobutanes. *J. Org. Chem.* **2019**, *84* (16), 10518–10525. https://doi.org/10.1021/acs.joc.9b01463.

(1354) Apeland, I. M.; Kählig, H.; Lorbeer, E.; Brinker, U. H. Probing the Nature and Extent of Stabilization within Foiled Carbenes: Homoallylic Participation by a Neighboring Cyclopropane Ring. *J. Org. Chem.* **2013**, *78* (10), 4879–4885. https://doi.org/10.1021/jo4004579.

(1355) Harmata, M.; Fletcher, V. R.; Claassen, R. J. Alkoxyvinyl Thionium Ions in Intramolecular 4 + 3 Cycloaddition Reactions. *J. Am. Chem. Soc.* **1991**, *113* (26), 9861–9862. https://doi.org/10.1021/ja00026a028.

(1356) Tietze, L. F.; Wünsch, J. R.; Noltemeyer, M. Stereoselective Sequential Photochemical Cycloaddition - Iminium Ion - Propargylsilane Cyclization. Synthesis of Quinolizidines and Pyrido [1,2-a]Azepines. *Tetrahedron* **1992**, *48* (11), 2081–2099. https://doi.org/10.1016/S0040-4020(01)88876-1.

(1357) Magnus, P.; Rodriguez-López, J.; Mulholland, K.; Matthews, I. Biomimetic Synthesis of the Pentacyclic Alkaloid (±)-Nirurine and Possible Biogenetic Rearrangement of a Precursor into (±)-Norsecurinine. *J. Am. Chem. Soc.* **1992**, *114* (1), 382–383. https://doi.org/10.1021/ja00027a070.

(1358) Eggleston, D. S.; Baures, P. W.; Grabowska, U.; Marson, C. M.; Walsgrove, T. Structures of Three Tricyclic γ-Lactams. *Acta Cryst. C* **1992**, *48* (12), 2177–2181. https://doi.org/10.1107/s0108270192003111.

(1359) Olivato, P. R.; Domingues, N. L. C.; Reis, A. K. C. A.; Vinhato, E.; Mondino, M. G.; Zukerman-Schpector, J.; Rittner, R.; Colle, M. D. Spectroscopic and Theoretical Studies of Some N-Methoxy-N-Methyl-2-[(4′-Substituted) Phenylsulfonyl]Propanamides. *J. Mol. Struct.* **2009**, *935* (1–3), 60–68. https://doi.org/10.1016/j.molstruc.2009.06.038.

(1360) Maity, S.; Kundu, A.; Pramanik, A. Synthesis of Biologically Important, Fluorescence Active 5-Hydroxy Benzo[g]Indoles through Four-Component Domino Condensations and Their Fluorescence "Turn-off" Sensing of Fe(Iii) Ions. *RSC Adv.* **2015**, *5* (65), 52852–52865. https://doi.org/10.1039/c5ra05780a.

(1361) Zhao, L. M.; Zhang, A. L.; Gao, H. S.; Zhang, J. H. Synthesis of Furo[3,2-c]Benzopyrans via an Intramolecular [4 + 2] Cycloaddition Reaction of o-Quinonemethides. *J. Org. Chem.* **2015**, *80* (20), 10353–10358. https://doi.org/10.1021/acs.joc.5b01641.

(1362) Zhao, Y.; Wang, H.; Li, X.; Wang, D.; Xin, X.; Wan, B. Selective Synthesis of Functionalized Pyrroles from 3-Aza-1,5-Enynes. *Org. Biomol. Chem.* **2016**, *14* (2), 526–541. https://doi.org/10.1039/c5ob01887k.

(1363) Ghorai, M. K.; Halder, S.; Das, S. Domino Michael-Michael and Aldol-Aldol Reactions: Diastereoselective Synthesis of Functionalized Cyclohexanone Derivatives Containing Quaternary Carbon Center. *J. Org. Chem.* **2015**, *80* (19), 9700–9712. https://doi.org/10.1021/acs.joc.5b01768.

(1364) Milde, B.; Leibeling, M.; Hecht, A.; Jones, P. G.; Visscher, A.; Stalke, D.; Grunenberg, J.; Werz, D. B. Oligoene-Based π-Helicenes or Dispiranes? Winding up Oligoyne Chains by a Multiple Carbopalladation/Stille/(Electrocyclization) Cascade. *Chem. Eur. J.* **2015**, *21* (45), 16136–16146. https://doi.org/10.1002/chem.201501797.

(1365) Al-Rashida, M.; Nagra, S. A.; Khan, I. U.; Kostakis, G.; Abbas, G. 3-(6-Fluoro-4-Oxo-4H-Chromen-3-Yl)-3,4-

Dihydro-2H-1,2,4-Benzothia-Diazine- 1,1-Dione. *Acta Cryst. E* **2010**, *66* (11), o2707–o2707. https://doi.org/10.1107/S1600536810038274.

(1366) Artem'ev, A. V.; Malysheva, S. F.; Gusarova, N. K.; Belogorlova, N. A.; Sukhov, B. G.; Sutyrina, A. O.; Matveeva, E. A.; Vasilevsky, S. F.; Govdi, A. I.; Gatilov, Y. V.; Albanov, A. I.; Trofimov, B. A. Reaction of Elemental Phosphorus with α-Methylstyrenes: One-Pot Synthesis of Secondary and Tertiary Phosphines, Prospective Bulky Ligands for Pd(II) Catalysts. *Tetrahedron* **2016**, *72* (4), 443–450. https://doi.org/10.1016/j.tet.2015.11.009.

(1367) Saglam, M. F.; Alborzi, A. R.; Payne, A. D.; Willis, A. C.; Paddon-Row, M. N.; Sherburn, M. S. Synthesis and Diels-Alder Reactivity of Substituted [4]Dendralenes. *J. Org. Chem.* **2016**, *81* (4), 1461–1475. https://doi.org/10.1021/acs.joc.5b02583.

(1368) Al-Masri, H. T.; Sieler, J.; Lönnecke, P.; Junk, P. C.; Hey-Hawkins, E. Synthesis, Characterization, and Crystal Structures of Novel Intramolecularly Base-Stabilized Borane Derivatives with Six- and Seven-Membered Chelate Rings. *Inorg. Chem.* **2004**, *43* (22), 7162–7169. https://doi.org/10.1021/ic049352n.

(1369) Wu, B.; Hua, R. Palladium-Catalyzed [3+2+1] Cyclocarbonylative Coupling of 1,3-Cyclohexanediones, Alkynes, and Carbon Monoxide: An Atom-Economic Route to Chromene-2,5-Dione Derivatives. *Tetrahedron Lett.* **2010**, *51* (49), 6433–6435. https://doi.org/10.1016/j.tetlet.2010.09.132.

(1370) Wang, C. Y.; Pan, G. H.; Chen, F.; Li, J. H. Oxidative Cyclization of 2-Allenyl-1,1′-Biphenyls with α-Carbonyl Alkyl Bromides: Facile Access to Functionalized Phenanthrenes. *Chem. Commun.* **2017**, *53* (34), 4730–4733. https://doi.org/10.1039/c7cc00483d.

(1371) Chung, M. C.; Chan, Y. H.; Chang, W. J.; Hou, D. R. Synthesis of 2,3-Dihydro-1: H -Pyrroles by Intramolecular Cyclization of N -(3-Butynyl)-Sulfonamides. *Org. Biomol. Chem.* **2017**, *15* (17), 3783–3790. https://doi.org/10.1039/c7ob00528h.

(1372) Sahoo, S. C.; Nath, U.; Pan, S. C. Direct Aerobic Oxidative Reactions of 2-Hydroxyacetophenones. *Eur. J. Org. Chem.* **2017**, *2017* (30), 4434–4438. https://doi.org/10.1002/ejoc.201700909.

(1373) Kudo, K.; Hashimoto, Y.; Saigo, K. Highly Stereoselective Cationic Cyclization Assisted by a Sulfenyl Group. *Tetrahedron Lett.* **1993**, *34* (44), 7063–7066. https://doi.org/10.1016/S0040-4039(00)61599-X.

(1374) Dennis, G. D.; Edwards-Davis, D.; Field, L. D.; Masters, A. F.; Maschmeyer, T.; Ward, A. J.; Buys, I. E.; Turner, P. Fused Supracyclopentadienyl Ligand Precursors. Synthesis, Structure, and Some Reactions of 1,3-Diphenylcyclopenta[l]Phenanthrene-2-One, 1,2,3-Triphenylcyclopenta[l]Phenanthrene-2-Ol, 1-Chloro-1,2,3-Triphenylcyclopenta[l]Phenanthrene, 1-Bromo-1,2,3-Triphen. *Aust. J. Chem.* **2006**, *59* (2), 135–146. https://doi.org/10.1071/CH05172.

(1375) Pokkuluri, P. R.; Scheffer, J. R.; Trotter, J. Dimethyl 9-Phenyl-1,4-Dihydro-1,4-Ethenoanthracene-11,12-Dicarboxylate. *Acta Cryst. C* **1994**, *50* (3), 415–417. https://doi.org/10.1107/s0108270193010054.

(1376) Fredericks, E. J.; Gindling, M. J.; Kroll, L. C.; Storhoff, B. N. Phosphine-Nitrile Ligands: The Molecular Structure of Cis-2-Diphenylphosphino-1-Cyanocyclopentane and Studies of the Donor/Acceptor Properties of This and Related Ligands. *J. Organomet. Chem.* **1994**, *465* (1–2), 289–296. https://doi.org/10.1016/0022-328X(94)87068-3.

(1377) Kavitha, C. V.; Lakshmi, S.; Basappa; Mantelingu, K.; Sridhar, M. A.; Shashidhara Prasad, J.; Rangappa, K. S. Synthesis and Molecular Structure Analysis of Venlafaxine Intermediate and Its Analog. *J. Chem. Crystallogr.* **2005**, *35* (12), 957–963. https://doi.org/10.1007/s10870-005-5249-y.

(1378) Link, M.; Niecke, E.; Nieger, M. Untersuchungen Am System Phosphirenimin/Iminophosphan — Insertionsreaktionen Und Isomerisierung. *Chem. Ber.* **1994**, *127* (2), 313–319. https://doi.org/10.1002/cber.19941270206.

(1379) Rana, S.; Brown, M.; Mukhopadhyay, C. FeCl3 Catalysed Multicomponent Divergent Synthesis of a Library of Indeno-Fused Heterocycles. *RSC Adv.* **2013**, *3* (10), 3291–3303. https://doi.org/10.1039/c2ra23332k.

(1380) Cooke, M. P.; Gopal, D. Tandem Metal-Halogen Exchange-Initiated Conjugate Addition Reactions of Conjugated Acetylenic Esters. *J. Org. Chem.* **1994**, *59* (1), 260–263. https://doi.org/10.1021/jo00080a048.

(1381) Aldeco-Pérez, E.; Rudler, H.; Parlier, A.; Alvarez, C.; Apan, M. T.; Herson, P.; Toscano, A. A Simple Synthesis of Cytotoxic Endoperoxide Lactones. *Tetrahedron Lett.* **2006**, *47* (51), 9053–9056. https://doi.org/10.1016/j.tetlet.2006.10.093.

(1382) Moon, D. J.; Al-Amin, M.; Lewis, R. S.; Arnold, K. M.; Yap, G. P. A.; Sims-Mourtada, J.; Chain, W. J. A Strategy toward Icetexane Natural Products. *Eur. J. Org. Chem.* **2018**, *2018* (25), 3348–3351. https://doi.org/10.1002/ejoc.201800707.

(1383) Dey, R.; Kumar, P.; Banerjee, P. Lewis Acid Catalyzed Annulation of Cyclopropane Carbaldehydes and Aryl

Hydrazines: Construction of Tetrahydropyridazines and Application Toward a One-Pot Synthesis of Hexahydropyrrolo[1,2- b]Pyridazines. *J. Org. Chem.* **2018**, *83* (10), 5438–5449. https://doi.org/10.1021/acs.joc.8b00332.

(1384) Niu, H.; Mangan, R. J.; Protchenko, A. V.; Phillips, N.; Unkrig, W.; Friedmann, C.; Kolychev, E. L.; Tirfoin, R.; Hicks, J.; Aldridge, S. Experimental and Quantum Chemical Studies of Anionic Analogues of N-Heterocyclic Carbenes. *Dalt. Trans.* **2018**, *47* (22), 7445–7455. https://doi.org/10.1039/c8dt01661e.

(1385) Eto, M.; Yoshitake, Y.; Harano, K.; Hisano, T. Cycloaddition Behaviour of Pyrazol-4-One N,N-Dioxides toward Unsaturated Compounds. Stereochemical and Mechanistic Aspect. *J. Chem. Soc. Perkin Trans. 2* **1994**, *2* (6), 1337–1345. https://doi.org/10.1039/p29940001337.

(1386) Hieu, T. H.; Anh, L. T.; Soldatenkov, A. T.; Vasil'ev, V. G.; Kotsuba, V. E.; Khrustalev, V. N. Unexpected Formation of [(Δ3-Piperideino)Pyrimidino]-14-Crown-4 Ethers in a Petrenko-Kritschenko Type Condensation. *Macroheterocycles* **2013**, *6* (4), 379–382. https://doi.org/10.6060/mhc131264h.

(1387) Michael, J. P.; Billing, D. G.; Maqutu, T. L. Crystal and Molecular Structure of 6-Exo-Methyl-6-Endo-Nitro-2-Exo-Phenylbicyclo[2.2.1]Heptan-2-Endo-Ol. *J. Chem. Crystallogr.* **1994**, *24* (5), 311–314. https://doi.org/10.1007/BF01670206.

(1388) Basu, P.; Sikdar, R.; Kumar, T.; Namboothiri, I. N. N. Synthesis of Functionalized Arenopyrans and Arenylsulfanes by Reacting Nitroallylic Acetates with Arenols and Arenethiols. *Eur. J. Org. Chem.* **2018**, *2018* (41), 5735–5743. https://doi.org/10.1002/ejoc.201801132.

(1389) Kelleher, S.; Muldoon, J.; Müller-Bunz, H.; Evans, P. Studies Concerning the Double Reduction of Diels-Alder Derived Bicylic Sulfonamides. *Tetrahedron Lett.* **2007**, *48* (27), 4733–4736. https://doi.org/10.1016/j.tetlet.2007.05.015.

(1390) Belyaeva, K. V.; Nikitina, L. P.; Afonin, A. V.; Vashchenko, A. V.; Muzalevskiy, V. M.; Nenajdenko, V. G.; Trofimov, B. A. Catalyst-Free 1:2 Annulation of Quinolines with Trifluoroacetylacetylenes: An Access to Functionalized Oxazinoquinolines. *Org. Biomol. Chem.* **2018**, *16* (43), 8038–8041. https://doi.org/10.1039/c8ob02379d.

(1391) Spek, A. L.; van der Steen, F. H.; Jastrzebski, J. T. B. H.; van Koten, G. Trans-3-Amino-1-Methyl-4-Phenyl-2-Azetidinone, C10H12N2O. *Acta Cryst. C* **1994**, *50* (12), 1933–1935. https://doi.org/10.1107/s0108270194007675.

(1392) Wu, J.; Yu, H.; Wang, Y.; Xing, X.; Dai, W. M. Unexpected Epimerization and Stereochemistry Revision of IMDA Adducts from Sorbate-Related 1,3,8-Nonatrienes. *Tetrahedron Lett.* **2007**, *48* (37), 6543–6547. https://doi.org/10.1016/j.tetlet.2007.07.046.

(1393) Mahadevegowda, S. H.; Khan, F. A. Grob-Type Fragmentation of 5-Oxabicyclo[2.1.1]Hexane System: A Strategy for Synthesis of Annulated and 2,2,5-Trisubstituted Tetrahydrofurans. *Tetrahedron* **2013**, *69* (39), 8494–8504. https://doi.org/10.1016/j.tet.2013.07.005.

(1394) Bakthadoss, M.; Kannan, D.; Selvakumar, R. A Multicomponent Cascade Reaction for the Synthesis of Novel Chromenopyranpyrazole Scaffolds. *Chem. Commun.* **2013**, *49* (93), 10947–10949. https://doi.org/10.1039/c3cc45502e.

(1395) Zhang, S.; Zhan, M.; Wang, Q.; Wang, C.; Zhang, W. X.; Xi, Z. Synthesis of Semibullvalene Derivatives via Co2(CO)8-Mediated Cyclodimerization of 1,4-Dilithio-1,3-Butadienes. *Org. Chem. Front.* **2014**, *1* (2), 130–134. https://doi.org/10.1039/c3qo00019b.

(1396) Wakchaure, P. B.; Easwar, S.; Puranik, V. G.; Argade, N. P. Facile Air-Oxidation of N-Homopiperonyl-5,6-Dimethoxyhomophthalimide: Simple and Efficient Access to Nuevamine. *Tetrahedron* **2008**, *64* (8), 1786–1791. https://doi.org/10.1016/j.tet.2007.11.104.

(1397) Felton, G. A. N. Electrocatalytic Reactions: Anion Radical Cyclobutanation Reactions and Electrogenerated Base Reactions. *Tetrahedron Lett.* **2008**, *49* (5), 884–887. https://doi.org/10.1016/j.tetlet.2007.11.171.

(1398) Bhunia, S.; Wang, K. C.; Liu, R. S. PtII-Catalyzed Synthesis of 9-Oxabicyclo[3.3.1]Nona-2,6-Dienes from 2-Alkynyl-1-Carbonylbenzenes and Allylsilanes by an Allylation/Annulation Cascade. *Angew. Chem. Int. Ed.* **2008**, *47* (27), 5063–5066. https://doi.org/10.1002/anie.200800826.

(1399) Gzella, A.; Wrzeciono, U.; Pöppel, W. Azole. 44. Uber Morpholinonitroimidazolderivate. *Acta Crystallogr. Sect. C Cryst. Struct. Commun.* **2000**, *56* (9), 1161–1163. https://doi.org/10.1107/S0108270100008635.

(1400) Poomathi, N.; Kamalraja, J.; Mayakrishnan, S.; Muralidharan, D.; Perumal, P. T. Indium Trichloride Catalysed Domino Reactions of Isatin: A Facile Access to the Synthesis of Spiro(Indoline-3,4′-Pyrano[2,3- c]Pyrazol)-2-One Derivatives. *Synlett* **2014**, *25* (5), 708–712. https://doi.org/10.1055/s-0033-1340666.

(1401) Szkop, K. M.; Geeson, M. B.; Stephan, D. W.; Cummins, C. C. Synthesis of Acyl(Chloro)Phosphines Enabled by

Phosphinidene Transfer. *Chem. Sci.* **2019**, *10* (12), 3627–3631. https://doi.org/10.1039/C8SC05657A.

(1402) Koenig, J. J.; Arndt, T.; Gildemeister, N.; Neudörfl, J. M.; Breugst, M. Iodine-Catalyzed Nazarov Cyclizations. *J. Org. Chem.* **2019**, *84* (12), 7587–7605. https://doi.org/10.1021/acs.joc.9b01083.

(1403) Miroslaw, B.; Koziol, A. E.; Bielenica, A.; Dziuba, K.; Struga, M. Substituent Effect on Supramolecular Motifs in Series of Succinimide Polycyclic Keto Derivatives - Spectroscopic, Theoretical and Crystallographic Studies. *J. Mol. Struct.* **2014**, *1074*, 695–702. https://doi.org/10.1016/j.molstruc.2014.05.029.

(1404) Ko, H. M.; Dong, G. Cooperative Activation of Cyclobutanones and Olefins Leads to Bridged Ring Systems by a Catalytic [4 + 2] Coupling. *Nat. Chem.* **2014**, *6* (8), 739–744. https://doi.org/10.1038/nchem.1989.

(1405) Bairy, G.; Nandi, A.; Manna, K.; Jana, R. Ruthenium(II)-Catalyzed Migratory C-H Allylation/Hydroamination Cascade for the Synthesis of Rutaecarpine Analogues. *Synthesis* **2019**, *51* (12), 2523–2531. https://doi.org/10.1055/s-0037-1611525.

(1406) Ramírez-Quirós, Y.; Balderas, M.; Escalante, J.; Quintana, D.; Gallardo, I.; Madrigal, D.; Molins, E.; Juaristi, E. X-Ray Crystallographic Study of Substituted Perhydropyrimidinones. Extreme Changes in Ring Conformation. *J. Org. Chem.* **1999**, *64* (23), 8668–8680. https://doi.org/10.1021/jo991297q.

(1407) Bhuvan Kumar, N. N.; Kumara Swamy, K. C. The Reaction of Allenes with Phosphorus(III) Compounds Bearing a P-NH-(t-Bu) Group: Isolation of Both Enantiomers in Crystalline Form from an Achiral System. *Tetrahedron Lett.* **2008**, *49* (50), 7135–7138. https://doi.org/10.1016/j.tetlet.2008.09.153.

(1408) Millán-Ortiz, A.; López-Valdez, G.; Cortez-Guzmán, F.; Miranda, L. D. A Novel Carbamoyl Radical Based Dearomatizing Spiroacylation Process. *Chem. Commun.* **2015**, *51* (39), 8345–8348. https://doi.org/10.1039/c4cc06192f.

(1409) Peters, K.; Peters, E. M.; Ach, M.; Quast, H. Crystal Structure of 4,5,2′,3′-Tetrahydro-4-(1,1-Dimethylethyl)-1,1′,3′- Trimethylspiro-{[1H][1,2,3]-Triazole-5,2′-[1H]-Benzimidazole}, [C6H4N2(CH3)2]C[CHN 3(CH3)(C4H9)]. *Zeitschrift fur Krist. New Cryst. Struct.* **2000**, *215* (2), 297–298. https://doi.org/10.1515/ncrs-2000-0252.

(1410) Irngartinger, H.; Altreuther, A.; Sommerfeld, T.; Stojanik, T. Pyramidalization in Derivatives of Bicyclo[5.1.0]Oct-1(7)-Enes and 2,2,5,5-Tetramethylbicyclo[4.1.0]Hept-1(6)-Enes. *Eur. J. Org. Chem.* **2000**, *2000* (24), 4059–4070. https://doi.org/10.1002/1099-0690(200012)2000:24<4059::aid-ejoc4059>3.0.co;2-v.

(1411) Rickard, C. E. F.; Brimble, M. A.; Pavia, G.; Stevenson, R. J. Trans -2-(2-Naphthyl)-3-(Phenylselenyl)Tetrahydropyran . *Acta Cryst. E* **2002**, *58* (8), o931–o932. https://doi.org/10.1107/s1600536802013260.

(1412) Toshimitsu, A.; Saeki, T.; Tamao, K. Enhanced Leaving Ability of Methoxy Group and Retarded Deprotonation on the Carbon Atom Linked to the 1-Position of 8-Phosphino-or 8-Amino-Naphthalene. *Chem. Lett.* **2002**, No. 3, 278–279. https://doi.org/10.1246/cl.2002.278.

(1413) Field, J. D.; Turner, P.; Harding, M. M.; Hatzikominos, T.; Kim, L. Carbocyclic Molecular Clefts Incorporating Hydrogen Bonding Features. *New J. Chem.* **2002**, *26* (6), 720–725. https://doi.org/10.1039/b109055k.

(1414) Ye, F.; Liu, C. G.; Wang, X. M.; Fu, Y.; Gao, S. A Convenient One-Pot Synthesis and Bioactivity of N-Dichloroacetyl-5-Aryl- 1,3-Oxazolidines. *Heterocycl. Commun.* **2013**, *19* (3), 201–205. https://doi.org/10.1515/hc-2013-0042.

(1415) Zhang, L.; Xu, X.; Tan, J.; Pan, L.; Xia, W.; Liu, Q. Tandem Michael Addition/Intramolecular Isocyanide [3 + 2] Cycloaddition: Highly Diastereoselective One Pot Synthesis of Fused Oxazolines. *Chem. Commun.* **2010**, *46* (19), 3357–3359. https://doi.org/10.1039/c001617a.

(1416) Itoh, K.; Kanemasa, S. A New Method for Enol Lactone Synthesis by a Michael Addition/Cyclization Sequence. *Tetrahedron Lett.* **2003**, *44* (9), 1799–1802. https://doi.org/10.1016/S0040-4039(03)00147-3.

(1417) Angle, S. R.; Belanger, D. S. Stereoselective Synthesis of 3-Hydroxyproline Benzyl Esters from N-Protected β-Aminoaldehydes and Benzyl Diazoacetate. *J. Org. Chem.* **2004**, *69* (13), 4361–4368. https://doi.org/10.1021/jo030360f.

(1418) Pèpe, G.; Lamarque, L.; Méou, A.; Brun, P. Crystal Structure of (1β,6β,7α)-7-Carbomethoxy-9-Oxabicyclo[4.3.0]Non-2-En-8-One, C10H12O4. *Zeitschrift fur Krist. New Cryst. Struct.* **2000**, *215* (1), 61–62. https://doi.org/10.1515/ncrs-2000-0134.

(1419) Lazar, S.; Soukri, M.; Leger, J. M.; Jarry, C.; Akssira, M.; Chirita, R.; Grig-Alexa, I. C.; Finaru, A.; Guillaumet, G. Efficient Synthesis of 2- and 3-Substituted-2,3-Dihydro [1,4]Dioxino[2,3-b]Pyridine Derivatives. *Tetrahedron* **2004**, *60* (31), 6461–6473. https://doi.org/10.1016/j.tet.2004.06.041.

(1420) Dang, M.; Hou, L.; Tong, X. RhI-Catalyzed Cyclizative Addition Reaction of 1,6-Enyne and Sulfonyl Chloride by Carbophilic Activation. *Chem. Eur. J.* **2016**, *22* (23), 7734–7738. https://doi.org/10.1002/chem.201601098.

(1421) Katritzky, A. R.; Manju, K.; Gromova, A. V.; Steel, P. J. β-Acylvinyl Anion and Dianion Equivalents: Lithiation of 1-[(2EZ)-3-Chloroprop-2-Enyl]-1H-1,2,3-Benzotriazole: Preparation and Elaboration of 1-(2-Oxiranylvinyl)-1H-Benzotriazoles. *J. Org. Chem.* **2004**, *69* (18), 6018–6023. https://doi.org/10.1021/jo049818j.

(1422) Jiang, B.; Yi, M. S.; Shi, F.; Tu, S. J.; Pindi, S.; Mc Dowell, P.; Li, G. A Multi-Component Domino Reaction for the Direct Access to Polyfunctionalized Indoles via Intermolecular Allylic Esterification and Indolation. *Chem. Commun.* **2012**, *48* (6), 808–810. https://doi.org/10.1039/c1cc15913e.

(1423) Gerhardt, V.; Tutughamiarso, M.; Bolte, M. Conformational Studies of Hydantoin-5-Acetic Acid and Orotic Acid. *Acta Cryst. C* **2012**, *68* (2), o92–o98. https://doi.org/10.1107/S0108270112001151.

(1424) Griesbeck, A. G.; Oelgemoller, M.; Lex, J. Photodecarboxylative Additions of α-Keto Carboxylates to Phthalimides Alkylation, Acylation and Ring Expansion. *Synlett* **2000**, No. 10, 1455–1457. https://doi.org/10.1055/s-2000-7623.

(1425) Steinwascher, J.; Lex, J.; Griesbeck, A. G. Crystal Structures of Cis- and Trans-2-Azido-Indan-1-Ol, C9H9N3O. *Zeitschrift fur Krist. New Cryst. Struct.* **2000**, *215* (4), 627–629. https://doi.org/10.1515/ncrs-2000-0473.

(1426) Zhang, D. H.; Wei, Y.; Shi, M. Gold(I)-Catalyzed Cycloisomerization of Nitrogen- and Oxygen-Tethered Alkylidenecyclopropanes to Tricyclic Compounds. *Chem. Eur. J.* **2012**, *18* (23), 7026–7029. https://doi.org/10.1002/chem.201200978.

(1427) Ishii, A.; Annaka, T.; Nakata, N. Convenient Syntheses and Photophysical Properties of 1-Thio- and 1-Seleno-1,3-Butadiene Fluorophores in Rigid Dibenzobarrelene and Benzobarrelene Skeletons. *Chem. Eur. J.* **2012**, *18* (21), 6428–6432. https://doi.org/10.1002/chem.201200761.

(1428) An, Y.; Wu, J. Synthesis of Tetrahydropyridine Derivatives through a Reaction of 1,6-Enynes, Sulfur Dioxide, and Aryldiazonium Tetrafluoroborates. *Org. Lett.* **2017**, *19* (21), 6028–6031. https://doi.org/10.1021/acs.orglett.7b03195.

(1429) Duff, T.; James, J. P.; Müller-Bunz, H. Synthesis and X-Ray Crystal Structure Determinations of Pyrrolidine-2,4-Diones 2-Imnopyrrolidin-5-Ones and 1,3-Oxazine-2,4-Diones Derived from Acetoacetanilides. *Heterocycles* **2006**, *68* (3), 465–474. https://doi.org/10.3987/COM-05-10568.

(1430) Penkett, C. S.; Sims, R. O.; Byrne, P. W.; Berritt, S.; Pennicott, L. E.; Rushton, S. P.; Avent, A. G.; Hitchcock, P. B. Palladium-Mediated Fragmentation Reactions of Meta Photocycloadducts to Afford Arylated or Oxidatively Cyclised Products. *Tetrahedron* **2006**, *62* (40), 9403–9409. https://doi.org/10.1016/j.tet.2006.07.060.

(1431) Nair, V.; Mathew, B.; Radhakrishnan, K. V.; Rath, N. P. Hetero Diels-Alder Reaction of o-Benzoquinones with Tetracyclone: An Efficient Synthesis of Benzodioxinone Derivatives. *Tetrahedron* **1999**, *55* (36), 11017–11026. https://doi.org/10.1016/S0040-4020(99)00609-2.

(1432) Qin, G.; Wang, Y.; Huang, H. Copper-Catalyzed Dehydrogenative Formal [4 + 2] and [3 + 2] Cycloadditions of Methylnaphthalenes and Electron-Deficient Alkenes. *Org. Lett.* **2017**, *19* (23), 6352–6355. https://doi.org/10.1021/acs.orglett.7b03194.

(1433) Stomberg, R.; Langer, V.; Lundquist, K. 2,3-Diaryl-3-Hydroxypropionic Acid Intermediates in the Synthesis of Threo Forms of 1,2-Diaryl-1,3-Propanediols. *Acta Cryst. C* **2006**, *62* (12), o684–o686. https://doi.org/10.1107/S0108270106046026.

(1434) Morgans, G. L.; Fernandes, M. A.; van Otterlo, W. A. L.; Michael, J. P. Base-Mediated Cyclization of 3-[2-(2-Oxo-2-Phenylethyl)-1-Pyrrolidinyl]Propanenitrile to 7-Phenyl-1,2,3,7,8,8a-Hexahydroindolizine-6-Carbonitrile: What Lies Between? *Eur. J. Org. Chem.* **2018**, *2018* (16), 1902–1909. https://doi.org/10.1002/ejoc.201701714.

(1435) Diab, S.; Noël-Duchesneau, L.; Sanselme, M.; Kondo, Y.; De Paolis, M.; Chataigner, I. High Pressure Elicits Unexpected Transformations of Plain Nitroaromatics with 4-(Cyclohex-1-En-1-Yl)Morpholine. *Eur. J. Org. Chem.* **2018**, *2018* (18), 2048–2052. https://doi.org/10.1002/ejoc.201800316.

(1436) Brinkworth, C.; Rozek, T.; Bowie, J. H.; Skelton, B. W.; White, A. H. Angucyclinones Related to Ochromycinone. IV. The Structures and Reactions of Unusual Diels-Alder Adducts Formed from Maleic Anhydride and Racemic 5,5-Dimethyl-3-Vinylcyclohex-2-En-1-Ol. *Aust. J. Chem.* **2000**, *53* (5), 403. https://doi.org/10.1071/CH00034.

(1437) Lozhkin, B. V.; Sigachev, A. S.; Kravchenko, A. N.; Lyssenko, K. A.; Kolotyrkina, N. G.; Makhova, N. N. The First Conglomerate in the Series of 2,4,6,8,10-Pentaazatricyclo[5.3.1.03.11]Undecane-1,5-Diones. *Mendeleev Commun.* **2007**, *17* (2), 85–87. https://doi.org/10.1016/j.mencom.2007.03.010.

(1438) Mandal, M.; Balamurugan, R. Triflic Acid-Mediated Expedient Synthesis of Benzo[a]Fluorenes and Fluorescent Benzo[a]Fluorenones. *Adv. Synth. Catal.* **2018**, *360* (7), 1453–1465. https://doi.org/10.1002/adsc.201701516.

(1439) Lumby, R. J. R.; Joensuu, P. M.; Lam, H. W. Diastereoselective Intermolecular Cobalt-Catalyzed Reductive Aldol Reactions of α,β-Unsaturated Amides with Ketones. *Org. Lett.* **2007**, *9* (21), 4367–4370.

https://doi.org/10.1021/ol701980e.

(1440) Lumby, R. J. R.; Joensuu, P. M.; Lam, H. W. Racemic and Asymmetric Cobalt-Catalysed Reductive Aldol Couplings of α,β-Unsaturated Amides with Ketones. *Tetrahedron* **2008**, *64* (33), 7729–7740. https://doi.org/10.1016/j.tet.2008.06.022.

(1441) Reddy, J. S.; Xu, B. H.; Mahdi, T.; Fröhlich, R.; Kehr, G.; Stephan, D. W.; Erker, G. Alkenylborane-Derived Frustrated Lewis Pairs: Metal-Free Catalytic Hydrogenation Reactions of Electron-Deficient Alkenes. *Organometallics* **2012**, *31* (15), 5638–5649. https://doi.org/10.1021/om3006068.

(1442) Bresien, J.; Schulz, A.; Thomas, M.; Villinger, A. A Bismuth–Arene σ-Complex – On the Edge of Menshutkin-Type Complexes. *Eur. J. Inorg. Chem.* **2019**, *2019* (9), 1279–1287. https://doi.org/10.1002/ejic.201900003.

(1443) Kreft, A.; Lücht, A.; Grunenberg, J.; Jones, P. G.; Werz, D. B. Kinetic Studies of Donor–Acceptor Cyclopropanes: The Influence of Structural and Electronic Properties on the Reactivity. *Angew. Chem. Int. Ed.* **2019**, *58* (7), 1955–1959. https://doi.org/10.1002/anie.201812880.

(1444) Nair, V.; Rajan, R.; Rath, N. P. A CAN-Induced Cyclodimerization - Ritter Trapping Strategy for the One-Pot Synthesis of 1-Amino-4-Aryltetralins from Styrenes. *Org. Lett.* **2002**, *4* (9), 1575–1577. https://doi.org/10.1021/ol0257934.

(1445) Griesbeck, A. G.; Gudipati, M. S.; Hirt, J.; Lex, J.; Oelgemoller, M.; Schmickler, H.; Schouren, F. Photoinduced Electron-Transfer Reactions with Quinolinic and Trimellitic Acid Imides: Experiments and Spin Density Calculations. *J. Org. Chem.* **2000**, *65* (21), 7151–7157. https://doi.org/10.1021/jo001070r.

(1446) Griesbeck, A. G.; Oelgemöller, M.; Lex, J. Photochemistry of MTM- and MTE-Esters of Ωphthalimido Carboxyhc Acids: Macrocyclization versus Deprotection. *J. Org. Chem.* **2000**, *65* (26), 9028–9032. https://doi.org/10.1021/jo001089u.

(1447) Batsanov, A. S.; Brooke, G. M.; Kenwright, A.; Wood, J. L. Pyrolysis Reactions of 4-Methyl-Tetrafluorophenyl and Pentafluorophenyl Prop-2-Enyl Ethers: Isomeric Tetrahydroinden-1-Ones from Both Intra-Molecular Diels-Alder Adducts of the Claisen Rearrangement Reaction from the 4-Me Derivative - Mechanistic Implicat. *J. Fluor. Chem.* **2002**, *113* (1), 123–131. https://doi.org/10.1016/S0022-1139(01)00503-6.

(1448) Yoshitake, Y.; Yamaguchi, K.; Kai, C.; Akiyama, T.; Handa, C.; Jikyo, T.; Harano, K. Cyclization of Electron-Deficient Cyclopentadienone with 2-Alkenyl and 2-Alkynylamines via Sequential Pericyclic Reaction Pathway. *J. Org. Chem.* **2001**, *66* (26), 8902–8911. https://doi.org/10.1021/jo015941o.

(1449) Greatrex, B. W.; Kimber, M. C.; Taylor, D. K.; Fallon, G.; Tiekink, E. R. T. 1,2-Dioxines as Masked Cis γ-Hydroxy Enones and Their Versatility in the Synthesis of Highly Substituted γ-Lactones. *J. Org. Chem.* **2002**, *67* (15), 5307–5314. https://doi.org/10.1021/jo0200421.

(1450) He, Z. L.; Wang, C. J. Ag(i)-Catalyzed Tandem [6+3] Annulation/Isomerization of Isocyanoacetates with Fulvenes: An Expedient Approach to Synthesize Fused Dihydropyridines. *Chem. Commun.* **2015**, *51* (3), 534–536. https://doi.org/10.1039/c4cc08382b.

(1451) Ponnuswamy, S.; Mohanraj, V.; Ilango, S. S.; Thenmozhi, M.; Ponnuswamy, M. N. Growth, Thermal, Mechanical, Structural and Optical Properties of Organic NLO Crystals of Novel Cis-2,6-Bis(2-Chlorophenyl)-3,3-Dimethylpiperidin-4-One. *J. Mol. Struct.* **2015**, *1081*, 449–456. https://doi.org/10.1016/j.molstruc.2014.10.063.

(1452) Zheng, H.; Zhu, Y.; Shi, Y. Palladium(0)-Catalyzed Heck Reaction/C-H Activation/Amination Sequence with Diaziridinone: A Facile Approach to Indolines. *Angew. Chem. Int. Ed.* **2014**, *53* (42), 11280–11284. https://doi.org/10.1002/anie.201405365.

(1453) Chang, T. W.; Ho, P. Y.; Mao, K. C.; Hong, F. E. Developing Five-Membered Heterocycle Substituted Phosphinous Acids as Ligands for Palladium-Catalyzed Suzuki-Miyaura and Catellani Reactions. *Dalt. Trans.* **2015**, *44* (39), 17129–17142. https://doi.org/10.1039/c5dt02611c.

(1454) Chang, M. Y.; Chen, Y. H.; Cheng, Y. C. Fe(OTf)3-Mediated Synthesis of Sulfonyl Dihydropyrans. *Tetrahedron* **2016**, *72* (4), 518–524. https://doi.org/10.1016/j.tet.2015.12.007.

(1455) Yogavel, M.; Velmurugan, D.; Sekar, K.; Schenk, H.; Fraanje, J.; Peschar, R.; Srinivasan, S.; Athappan, P. R.; Rafi, Z. A. 3-Benzoyl-2-Hydroxy-2-Methylchromene. *Acta Cryst. E* **2003**, *59* (3), o266–o268. https://doi.org/10.1107/S1600536803002411.

(1456) Li, X.; Danishefsky, S. J. Cyclobutenone as a Highly Reactive Dienophile: Expanding upon Diels-Alder Paradigms. *J. Am. Chem. Soc.* **2010**, *132* (32), 11004–11005. https://doi.org/10.1021/ja1056888.

(1457) Cai, X.; Zhai, Y.; Ghiviriga, I.; Abboud, K. A.; Dolbier, W. R. Reactions of TFDA with Ketones. Synthesis of Difluoromethyl 2,2-Difluorocyclopropyl Ethers. *J. Org. Chem.* **2004**, *69* (12), 4210–4215.

https://doi.org/10.1021/jo049570y.

(1458) Ismiyev, A. I.; Maharramov, A. M.; Rashidov, B. A.; Mammadova, G. Z.; Askerov, R. K. Rac-Ethyl 6-Hydroxy-6-Methyl-3-Oxo-4-Phenyl-1,3,4,5,6,7-Hexahydrobenzo[c] [1,2]Oxazole-5-Carboxylate. *Acta Cryst. E* **2011**, *67* (11), o3018–o3018. https://doi.org/10.1107/S1600536811042395.

(1459) Eisenbeis, S. A.; Phillips, J. R.; Rescek, D.; Oyola-Cintron, Y. Synthesis of Azepino[3,4b]Indoles via the Plancher Rearrangement. *Tetrahedron Lett.* **2010**, *51* (33), 4303–4305. https://doi.org/10.1016/j.tetlet.2010.06.029.

(1460) Mirzaei, P.; Amanpour, T.; Naderi, S.; Soorki, A. A. Nef-Isocyanide -Based One-Pot Two-Step Three Component Dihydrobenzo[4,5]Imidazo[2,1-b]Thiazoles Synthesis. *J. Heterocycl. Chem.* **2016**, *53* (6), 1783–1786. https://doi.org/10.1002/jhet.2484.

(1461) Mothe, S. R.; Kothandaraman, P.; Rao, W.; Chan, P. W. H. Rapid Access to Halohydrofurans via Brønsted Acid-Catalyzed Hydroxylation/Halocyclization of Cyclopropyl Methanols with Water and Electrophilic Halides. *J. Org. Chem.* **2011**, *76* (8), 2521–2531. https://doi.org/10.1021/JO102374Z.

(1462) Pinto, O.; Sardinha, J.; Vaz, P. D.; Piedade, F.; Calhorda, M. J.; Abramovitch, R.; Nazareth, N.; Pinto, M.; Nascimento, M. S. J.; Rauter, A. P. Synthesis of Tetrahydronaphthalene Lignan Esters by Intramolecular Cyclization of Ethyl P-Azidophenyl-2-Phenylalkanoates and Evaluation of the Growth Inhibition of Human Tumor Cell Lines. *J. Med. Chem.* **2011**, *54* (9), 3175–3187. https://doi.org/10.1021/jm101182s.

(1463) Sun, H.; Cui, B.; Duan, L.; Li, Y. M. Intramolecular Aminoalkoxylation of Unfunctionalized Olefins via Intramolecular Iodoamination and Aziridinium Ion Ring-Opening Sequence. *Org. Lett.* **2017**, *19* (7), 1520–1523. https://doi.org/10.1021/acs.orglett.7b00284.

(1464) Zhang, Y.; Yu, A.; Jia, J.; Ma, S.; Li, K.; Wei, Y.; Meng, X. NaH Promoted [4+3] Annulation of Crotonate-Derived Sulfur Ylides with Thioaurones: Synthesis of 2,5-Dihydrobenzo[4,5]Thieno[3,2-: B] Oxepines. *Chem. Commun.* **2017**, *53* (77), 10672–10675. https://doi.org/10.1039/c7cc04466f.

(1465) Roe, S. J.; Hughes, D. L.; Aggarwal, P.; Stockman, R. A. Investigation of a Unified Strategy for the Synthesis of Anatoxin Analogues: Scope and Limitations. *Synthesis* **2009**, No. 22, 3775–3784. https://doi.org/10.1055/s-0029-1217021.

(1466) Basavaiah, D.; Thamizharasi, P. Baylis–Hillman Reaction: In Situ Generated Isoquinolinium Species as Excellent Electrophiles for Coupling with Alkyl Acrylates and Acrylonitrile. *Eur. J. Org. Chem.* **2017**, *2017* (34), 5135–5140. https://doi.org/10.1002/ejoc.201700743.

(1467) Clavier, H.; Giordano, L.; Tenaglia, A. Palladium-Mediated Phosphine-Dependent Chemoselective Bisallylic Alkylation Leading to Spirocarbocycles. *Angew. Chem. Int. Ed.* **2012**, *51* (34), 8648–8651. https://doi.org/10.1002/anie.201204629.

(1468) Yang, C. H.; Lee, L. T.; Yang, J. H.; Wang, Y.; Lee, G. H. Spiropyrazolines from Tandem Reaction of Azides and Alkyl Vinyl Ketones. *Tetrahedron* **1994**, *50* (42), 12133–12142. https://doi.org/10.1016/S0040-4020(01)89566-1.

(1469) Hartrampf, N.; Winter, N.; Pupo, G.; Stoltz, B. M.; Trauner, D. Total Synthesis of the Norhasubanan Alkaloid Stephadiamine. *J. Am. Chem. Soc.* **2018**, *140* (28), 8675–8680. https://doi.org/10.1021/jacs.8b01918.

(1470) Jourdan, J. P.; Rochais, C.; Legay, R.; Sopkova De Oliveira Santos, J.; Dallemagne, P. An Unusual Boron Tribromide-Mediated, One-Pot Bromination/Cyclization Reaction. Application to the Synthesis of a Highly Strained Cyclopenta[1,3] Cyclopropa[1,2-b]Pyrrolizin-8-One. *Tetrahedron Lett.* **2013**, *54* (9), 1133–1136. https://doi.org/10.1016/j.tetlet.2012.12.056.

(1471) Burak, K.; Ciunik, Z.; Głowiak, T. Crystal Structure of Two Oximes in Cis-Pinane Series. *J. Chem. Crystallogr.* **1994**, *24* (8), 503–506. https://doi.org/10.1007/BF01666727.

(1472) Furusawa, T.; Kawano, M.; Fujita, M. The Confined Cavity of a Coordination Cage Suppresses the Photocleavage of α-Diketones to Give Cyclization Products through Kinetically Unfavorable Pathways. *Angew. Chem. Int. Ed.* **2007**, *46* (30), 5717–5719. https://doi.org/10.1002/anie.200701250.

(1473) Gorbunov, E. B.; Novikova, R. K.; Plekhanov, P. V.; Slepukhin, P. A. 2-AZIDO-5-NITROPYRIMIDINE : SYNTHESIS , MOLECULAR STRUCTURE , AND REACTIONS. *Khimiya Geterotsiklicheskikh Soedin.* **2013**, *49* (5), 766–775. https://doi.org/10.1007/s10593-013-1308-z.

(1474) Zhang, Y. Q.; Zhu, X. Q.; Chen, Y. B.; Tan, T. De; Yang, M. Y.; Ye, L. W. Synthesis of Isothiochroman-3-Ones via Metal-Free Oxidative Cyclization of Alkynyl Thioethers. *Org. Lett.* **2018**, *20* (23), 7721–7725. https://doi.org/10.1021/acs.orglett.8b03462.

(1475) Van Eupen, J. T. H.; Elffrink, W. W. J.; Keltjens, R.; Bennema, P.; De Gelder, R.; Smits, J. M. M.; Van Eck, E. R. H.; Kentgens, A. P. M.; Deij, M. A.; Meekes, H.; Vlieg, E. Polymorphism and Migratory Chiral Resolution of the Free

Base of Venlafaxine. A Remarkable Topotactical Solid State Transition from a Racemate to a Racemic Conglomerate. *Cryst. Growth Des.* **2008**, *8* (1), 71–79. https://doi.org/10.1021/cg700831z.

(1476) Aulenta, F.; Wefelscheid, U. K.; Brüdgam, I.; Reißig, H. U. Nitrogen-Containing Tricyclic and Tetracyclic Compounds by Stereoselective Samarium Diiodide Promoted Cyclizations of Quinolyl-Substituted Ketones - A New Access to Azasteroids. *Eur. J. Org. Chem.* **2008**, No. 13, 2325–2335. https://doi.org/10.1002/ejoc.200800019.

(1477) Naseema, K.; Rao, V.; Sujith, K. V. A Phase-Matchable Nonlinear Optical Crystal. *October* **2009**, *73* (4), 719–729.

(1478) Fun, H. K.; Sujith, K. V.; Patil, P. S.; Kalluraya, B.; Chantrapromma, S. 4-Amino-3-{1-[4-(2-Methyl-Prop-Yl)Phen-Yl]Eth-Yl}-1H-1,2,4-Triazole-5(4H) -Thione. *Acta Cryst. E* **2008**, *64* (8), o1590–o1591. https://doi.org/10.1107/S1600536808022794.

(1479) Xu, H.; Zhu, Y.; Xie, L.; Wang, X. Design, Synthesis and Bioactivity of Novel 2-Aroyl-5-Alkylcyclopentanone Derivatives. *Chinese J. Org. Chem.* **2013**, *33* (1), 143–147. https://doi.org/10.6023/cjoc201208029.

(1480) Rashid, S. O.; Almadhhi, S. S.; Berrisford, D. J.; Raftery, J.; Vitorica-Yrezabal, I.; Whitehead, G.; Quayle, P. Radical Truce-Smiles Reactions on an Isoxazole Template: Scope and Limitations. *Tetrahedron* **2019**, *75* (16), 2413–2430. https://doi.org/10.1016/j.tet.2019.03.015.

(1481) Nikolaev, V. V.; Schulze, B.; Heimgartner, H.; Nikolaev, V. A. Spirocyclic and Fused Derivatives of Maleimide Based on Intra- and Intermolecular Reactions of Carbonyl Ylides from Diazocarbonyl Compounds. *Heterocycles* **2007**, *73* (C), 433–449. https://doi.org/10.3987/COM-07-S(U)13.

(1482) Low, Y. Y.; Gan, C. Y.; Kam, T. S. Andransinine: An Unusual Case of Spontaneous Resolution in an Indole Alkaloid Derivative. *J. Nat. Prod.* **2014**, *77* (6), 1532–1535. https://doi.org/10.1021/np500289t.

(1483) Parmar, B.; Patel, P.; Pillai, R. S.; Tak, R. K.; Kureshy, R. I.; Khan, N. U. H.; Suresh, E. Cycloaddition of CO2 with an Epoxide-Bearing Oxindole Scaffold by a Metal-Organic Framework-Based Heterogeneous Catalyst under Ambient Conditions. *Inorg. Chem.* **2019**, *58* (15), 10084–10096. https://doi.org/10.1021/acs.inorgchem.9b01234.

(1484) Castillo-Contreras, E. B.; Stahl, A. M.; Dake, G. R. Annulated Isoxazoles via [3 + 2] Cycloaddition of Alkenyl Bromides and Oximoyl Chlorides and Ag(I) Promoted Elimination. *J. Org. Chem.* **2014**, *79* (15), 7250–7255. https://doi.org/10.1021/jo501376q.

(1485) Lewis, R. T.; MacLeod, A. M.; Merchant, K. J.; Kelleher, F.; Sanderson, I.; Herbert, R. H.; Cascieri, M. A.; Sadowski, S.; Ball, R. G.; Hoogsteen, K. Tryptophan-Derived NK1 Antagonists: Conformationally Constrained Heterocyclic Bioisosteres of the Ester Linkage. *J. Med. Chem.* **1995**, *38* (6), 923–933. https://doi.org/10.1021/jm00006a011.

(1486) El Bouakher, A.; Allouchi, H.; Abrunhosa-Thomas, I.; Troin, Y.; Guillaumet, G. Suzuki-Miyaura Reactions of Halospirooxindole Derivatives. *Eur. J. Org. Chem.* **2015**, *2015* (16), 3450–3461. https://doi.org/10.1002/ejoc.201500268.

(1487) Li, H.; Pang, M.; Wu, B.; Meng, J. Synthesis, Crystal Structure and Photochromism of a Novel Spiro[Indoline-Naphthaline]Oxazine Derivative. *J. Mol. Struct.* **2015**, *1087*, 23–29. https://doi.org/10.1016/j.molstruc.2015.01.050.

(1488) Gómez, S. L.; Sanabria, C. M.; Palma, A.; Bahsas, A.; Cobo, J.; Glidewell, C. Four Differently Substituted 2-Aryl-2,3,4,5-Tetra-Hydro-1H-1,4-Ep-Oxy-1- Benzazepines: Hydrogen-Bonded Structures in One, Two and Three Dimensions. *Acta Cryst. C* **2009**, *65* (9), o465–o469. https://doi.org/10.1107/S0108270109030339.

(1489) Bew, S.; Carrington, R.; Hughes, D.; Liddle, J.; Pesce, P. An Organocatalytic Synthesis of Cis-N-Alkyl- and N-Arylaziridine Carboxylates. *Adv. Synth. Catal.* **2009**, *351* (16), 2579–2588. https://doi.org/10.1002/adsc.200900474.

(1490) Štverková, S.; Žák, Z.; Jonas, J. 1,3-dipolar Cycloaddition of Diphenylnitrilimine to Substituted Dihydro-3-methylene-2(3H)-furanones. *Liebigs Ann.* **1995**, *1995* (3), 477–480. https://doi.org/10.1002/jlac.199519950365.

(1491) Urtiaga, M. K.; Arriortua, M. I.; Badía, D.; Domínguez, E.; González-Cameno, A. M.; Ochando, L.; Reventos, M.; Debaerdemaeker, T. The Effect of Substituents on the Crystal Structure of β-Amino Alcohols. 2-[(3,4-Dimethoxybenzyl)(Methyl)Amino]-2-Phenylethanol. *Acta Cryst. C* **1995**, *51* (6), 1175–1177. https://doi.org/10.1107/s0108270194013855.

(1492) Presset, M.; Coquerel, Y.; Rodriguez, J. Microwave-Assisted Domino and Multicomponent Reactions with Cyclic Acylketenes: Expeditious Syntheses of Oxazinones and Oxazindiones. *Org. Lett.* **2009**, *11* (24), 5706–5709. https://doi.org/10.1021/ol9024056.

(1493) Sugimoto, A.; Kimoto, S.; Adachi, T.; Inoue, H. Photocleavage of the C-O Bond of 9-Phenanthrylmethyl ω-

Anilinoalkyl Ethers via Photo-Induced Intramolecular Electron Transfer. *J. Chem. Soc. Perkin Trans. 1* **1995**, No. 11, 1459–1466. https://doi.org/10.1039/p19950001459.

(1494) Bamatraf, M. M. M.; Vernon, J. M.; Wilson, G. D. Ring Expansion Reactions of Isoquinoline Derivatives to 2-Benzazocine and 3-Benzazecine Derivatives. *J. Chem. Soc. Perkin Trans. 1* **1995**, No. 13, 1647–1648. https://doi.org/10.1039/p19950001647.

(1495) Huang, S.; Shao, Y.; Zhang, L.; Zhou, X. Cycloamidination of Aminoalkenes with Nitriles: Synthesis of Substituted 2-Imidazolines and Tetrahydropyrimidines. *Angew. Chem. Int. Ed.* **2015**, *54* (48), 14452–14456. https://doi.org/10.1002/anie.201508442.

(1496) Tiwari, V. K.; Kamal, N.; Kapur, M. One Substrate, Two Modes of C-H Functionalization: A Metal-Controlled Site-Selectivity Switch in C-H Arylation Reactions. *Org. Lett.* **2017**, *19* (1), 262–265. https://doi.org/10.1021/acs.orglett.6b03558.

(1497) Vicente, J.; Abad, J. A.; Gil-Rubio, J.; Jones, P. G. Palladium-Assisted Formation of Carbon-Carbon Bonds. 3.1 Study of Reactions of Bis{η2-(2,3,4-Trimethoxy-6-Acetylphenyl-C,O)}bis(μ-Chloro)Dipalladium(II) with Symmetrical and Unsymmetrical Internal Alkynes. A Stoichiometric Route to Highly Functionalized S. *Organometallics* **1995**, *14* (6), 2677–2688. https://doi.org/10.1021/om00006a014.

(1498) Schaper, L. A.; öfele, K.; Kadyrov, R.; Bechlars, B.; Drees, M.; Cokoja, M.; Herrmann, W. A.; Kühn, F. E. N-Heterocyclic Carbenes via Abstraction of Ammonia: 'Normal' Carbenes with 'Abnormal' Character. *Chem. Commun.* **2012**, *48* (32), 3857–3859. https://doi.org/10.1039/c2cc30611e.

(1499) Wilson, R. M.; Patterson, W. S.; Austen, S. C.; Ho, D. M.; Krause Bauer, J. A. Orbital Symmetry Governed Reactions under High-Intensity Argon Laser-Jet Conditions: The Involvement of a [1, 5S] Sigmatropic Shift in the Photocyclization of an o-Alkenylbenzaldehyde to a Benzocyclobutenone. *J. Am. Chem. Soc.* **1995**, *117* (29), 7820–7821. https://doi.org/10.1021/ja00134a033.

(1500) Boukraa, M.; Jouini, T.; Barkallah, S.; Ben Akacha, A.; Zantour, H.; Baccar, B. Diphenyl(2-Oxocyclohexyl)Phosphine Sulfide. *Acta Cryst. C* **1995**, *51* (9), 1851–1853. https://doi.org/10.1107/s0108270195001557.

(1501) Sukumar, N.; Ponnuswamy, M. N.; Thenmozhiyal, J. C.; Jeyaraman, R. Structural Investigation of 2,6-Di-2-Furyl-3,5-Dimethyl-4-Piperidinone and Its N-Nitroso Derivative in Solution and the Solid State-Influence of the Nitroso Moiety on the Conformation of the Piperidine Ring and Orientation of Its Substituents. *Bull. Chem. Soc. Jpn.* **1994**, *67* (4), 1069–1073. https://doi.org/10.1246/bcsj.67.1069.

(1502) Podsiadło, M.; Patyk, E.; Katrusiak, A. Chiral Aggregation Hierarchy in High-Pressure Resolved 2-Butanol and 2,3-Butanediol. *CrystEngComm* **2012**, *14* (20), 6419–6423. https://doi.org/10.1039/c2ce25372k.

(1503) Hashikawa, Y.; Murata, M.; Wakamiya, A.; Murata, Y. Palladium-Catalyzed Cyclization: Regioselectivity and Structure of Arene-Fused C60 Derivatives. *J. Am. Chem. Soc.* **2017**, *139* (45), 16350–16358. https://doi.org/10.1021/jacs.7b09459.

(1504) Zhou, Y.; Qian, L.; Liu, M.; Wu, G.; Gao, W.; Ding, J.; Huang, X.; Wu, H. The Influence of Different N-Substituted Groups on the Mechanochromic Properties of 1,4-Dihydropyridine Derivatives with Simple Structures. *RSC Adv.* **2017**, *7* (81), 51444–51451. https://doi.org/10.1039/c7ra09515e.

(1505) Marquardt, C.; Kahoun, T.; Baumann, J.; Timoshkin, A. Y.; Scheer, M. Coordination of Boron-Centered Lewis Acids by Organo-Substituted Phosphanylboranes. *Zeitschrift f?r Anorg. und Allg. Chemie* **2017**, *643* (21), 1326–1330. https://doi.org/10.1002/zaac.201700219.

(1506) Hübner, A.; Diefenbach, M.; Bolte, M.; Lerner, H. W.; Holthausen, M. C.; Wagner, M. Confirmation of an Early Postulate: B-C-B Two-Electron-Three-Center Bonding in Organo(Hydro)Boranes. *Angew. Chem. Int. Ed.* **2012**, *51* (50), 12514–12518. https://doi.org/10.1002/anie.201207335.

(1507) Sengoden, M.; Punniyamurthy, T. "On Water": Efficient Iron-Catalyzed Cycloaddition of Aziridines with Heterocumulenes. *Angew. Chem. Int. Ed.* **2013**, *52* (2), 572–575. https://doi.org/10.1002/anie.201207746.

(1508) Dahiya, A.; Ali, W.; Patel, B. K. Catalyst and Solvent Free Domino Ring Opening Cyclization: A Greener and Atom Economic Route to 2-Iminothiazolidines. *ACS Sustain. Chem. Eng.* **2018**, *6* (3), 4272–4281. https://doi.org/10.1021/acssuschemeng.7b04723.

(1509) Cao, D. K.; Lu, Y. H.; Zheng, T.; Zhang, Y. H.; Li, Y. Z.; Zheng, L. M. Reaction of an Anthracene-Based Cyclic Phosphonate Ester with Trimethylsilyl Bromide Unexpectedly Generating Two Phosphonates: Syntheses, Crystal Structures and Fluorescent Properties. *RSC Adv.* **2013**, *3* (12), 4001–4007. https://doi.org/10.1039/c3ra22863k.

(1510) Schneider, R.; Gerardin, P.; Loubinoux, B.; Rihs, G. Reaction of β-Nitroenones with Thiophenol Synthesis of 5-

Hydroxy-4-(Phenylthio)-2-Isoxazoline 2-Oxides. *Tetrahedron* **1995**, *51* (17), 4997–5010. https://doi.org/10.1016/0040-4020(95)98697-G.

(1511) Connor, D. A.; Arnold, D. R.; Bakshi, P. K.; Cameron, T. S. Photochemical Nucleophile–Olefin Combination, Aromatic Substitution (Photo-NOCAS) Reaction. Part 9: Methanol-2,6-Dimethyl-1,6-Heptadiene, and 1,4-Dicyanobenzene. *Can. J. Chem.* **1995**, *73* (6), 762–771. https://doi.org/10.1139/v95-096.

(1512) Okada, K.; Suzuki, R.; Oda, M. Novel Boron-Nitrogen Containing Compounds from the Reaction of Organolithiums with Complexes between Dimesitylfluoroborane and Six- or Five-Membered Aza Aromatic Compounds. *J. Chem. Soc. Chem. Commun.* **1995**, No. 20, 2069–2070. https://doi.org/10.1039/C39950002069.

(1513) Thiruvalluvar, A.; Sankar Raja Raj, K.; Krishna Pillay, M.; Venkatasubramanian, K. N-Benzoyl-2-Phenyldecahydroquinolin-4-One, C22H23NO2. *Acta Cryst. C* **1995**, *51* (12), 2641–2643. https://doi.org/10.1107/s0108270195003702.

(1514) Yakoubi, A.; Kallel, A.; Bizid, A.; Baklouti, A. Trans-2-Fluorocyclohexyl p-Toluenesulfonate and Trans-2-Fluorocycloheptyl p-Toluenesulfonate. *Acta Cryst. C* **1995**, *51* (12), 2705–2707. https://doi.org/10.1107/s0108270195008080.

(1515) Peng, C.; Zhang, J.; Xue, J.; Li, S.; Wang, X. N.; Chang, J. Lewis Acids Catalyzed Annulations of Ynamides with Acyl Chlorides for Constructing 4-Amino-2-Naphthol Derivatives and 3-Aminocyclobutenones. *J. Org. Chem.* **2018**, *83* (16), 9256–9266. https://doi.org/10.1021/acs.joc.8b01255.

(1516) Mousser, A.; Badeche, S.; Merazig, H.; Benhaoua, H.; Sidhoum, D. A. N-Benzyl-3,4-Diphenyl-2-Azetidinone. *Acta Cryst. C* **1996**, *52* (1), 201–203. https://doi.org/10.1107/S0108270195004598.

(1517) Ianelli, S.; Nardelli, M.; Belletti, D.; Jamart-Grégoire, B.; Mercier-Girardot, S.; Caubère, P. Structural Aspects of the Nucleophilic Attack of Ketone Enolate on Dehydrodihydropyran. *Acta Cryst. C* **1996**, *52* (1), 237–244. https://doi.org/10.1107/S0108270195010134.

(1518) Zhang, X.; Wang, M.; Li, P.; Wang, L. N-Bu4NI/TBHP-Catalyzed Direct Amination of Allylic and Benzylic C(Sp3)–H with Anilines under Metal-Free Conditions. *Chem. Commun.* **2014**, *50* (59), 8006–8009. https://doi.org/10.1039/c4cc01189a.

(1519) Endo, K.; Nakano, T.; Fujinami, S.; Ukaji, Y. Chemoselective Carbozincation of Cyclopropene for C-C Bond Formation and Cleavage in a Single Operation. *Eur. J. Org. Chem.* **2013**, *2013* (29), 6514–6518. https://doi.org/10.1002/ejoc.201301026.

(1520) Maity, S.; Pathak, S.; Pramanik, A. Substituted Benzo[a]Carbazoles and Indoleacetic Acids from Arylglyoxals and Enamines through Domino Condensation, Thermal Cyclization, and Aromatization. *Eur. J. Org. Chem.* **2014**, *2014* (21), 4651–4662. https://doi.org/10.1002/ejoc.201402085.

(1521) Almansour, A. I.; Suresh Kumar, R.; Arumugam, N.; Bianchini, G.; Menéndez, J. C.; Al-thamili, D. M.; Periyasami, G.; Altaf, M. Design and Synthesis of A- and D Ring-Modified Analogues of Luotonin A with Reduced Planarity. *Tetrahedron Lett.* **2019**, *60* (23), 1514–1517. https://doi.org/10.1016/j.tetlet.2019.05.010.

(1522) Dai, P.; Tan, X.; Luo, Q.; Yu, X.; Zhang, S.; Liu, F.; Zhang, W. H. Synthesis of 3-Acyl-Isoxazoles and Δ2-Isoxazolines from Methyl Ketones, Alkynes or Alkenes, and Tert-Butyl Nitrite via a Csp3-H Radical Functionalization/Cycloaddition Cascade. *Org. Lett.* **2019**, *21* (13), 5096–5100. https://doi.org/10.1021/acs.orglett.9b01683.

(1523) Liu, L.; Sun, H. [HCo(CO)4] -Catalyzed Three-Component Cycloaddition of Epoxides, Imines, and Carbon Monoxide: Facile Construction of 1,3-Oxazinan-4-Ones. *Angew. Chem. Int. Ed.* **2014**, *53* (37), 9865–9869. https://doi.org/10.1002/anie.201403998.

(1524) Szabó, A. E.; Stájer, G.; Sohár, P.; Sillanpää, R.; Bernáth, G.; Seddon, K. R.; Bao-Sheng, L. Preparation and Structure of Saturated 1-Oxopyrrolo[2,1-b]Quinazoline and 1-Oxopyrrolo[1,2-a]Quinazoline. *Acta Chem. Scand.* **1995**, *49*, 751–754. https://doi.org/10.3891/acta.chem.scand.49-0751.

(1525) Yamada, S. Effects of C(O)-N Bond Rotation on the 13C, 15N, and 17O NMR Chemical Shifts, and Infrared Carbonyl Absorption in a Series of Twisted Amides. *J. Org. Chem.* **1996**, *61* (3), 941–946. https://doi.org/10.1021/jo9516953.

(1526) Zhou, D.; Li, Z. H.; Li, J.; Li, S. H.; Wang, M. W.; Luo, X. L.; Ding, G. L.; Sheng, R. L.; Fu, M. J.; Tang, S. Copper-Catalysed Alkylarylation of Activated Alkenes Using AIBN and beyond: An Access to Cyano-Containing Oxindoles. *Eur. J. Org. Chem.* **2015**, *2015* (7), 1606–1612. https://doi.org/10.1002/ejoc.201403499.

(1527) Hellier, D. G.; Motevalli, M. Cis,Trans-4,5-Diphenyl-1,3,2-Dioxathiolane 2-Oxide, Cis,Cis-4,5-Diphenyl-1,3,2-Dioxathiolane 2-Oxide and 4,4-Diphenyl-1,3,2-Dioxathiolane 2-Oxide. *Acta Cryst. C* **1996**, *52* (3), 739–743. https://doi.org/10.1107/S0108270195012984.

(1528) Mori, Y.; Maeda, K. Photochemical Reactions of Anthracene-Naphthalene Bichromophoric Systems Linked by a Three-Carbon Chain. *J. Chem. Soc. Perkin Trans. 2* **1996**, *1* (1), 113–119. https://doi.org/10.1039/P29960000113.

(1529) Butts, C. P.; Eberson, L.; Hartshorn, M. P.; Robinson, W. T. Photochemical Nitration by Tetranitromethane. Part XXVII. Adduct Formation in the Photochemical Reaction of 4-Methylanisole. Solvent and Temperature Effects on the Regiochemistry of Reaction of the Radical Cation of 4-Methylanisole. *Acta Chem. Scand.* **1996**, *50* (2), 122–131. https://doi.org/10.3891/acta.chem.scand.50-0122.

(1530) André, C.; Luger, P.; Lotz, S.; Fehlhammer, W. P. The Crystal Packing Modes of Three Sterically Overcrowded Imidazole Derivatives. *Acta Cryst. B* **1996**, *52* (2), 369–375. https://doi.org/10.1107/S0108768195011293.

(1531) Block, E.; Thiruvazhi, M.; Toscano, P. J.; Bayer, T.; Grisoni, S.; Zhao Shu-Hai. Allium Chemistry: Structure, Synthesis, Natural Occurrence in Onion (Allium Cepa), and Reactions of 2,3-Dimethyl-5,6-Dithiabicyclo[2.1.1]Hexane S-Oxides. *J. Am. Chem. Soc.* **1996**, *118* (12), 2790–2798. https://doi.org/10.1021/ja951134t.

(1532) Kavala, M.; Boča, R.; Dlháň, L.; Brezová, V.; Breza, M.; Kožíšek, J.; Fronc, M.; Herich, P.; Švorc, L.; Szolcsányi, P. Preparation and Spectroscopic, Magnetic, and Electrochemical Studies of Mono-/Biradical TEMPO Derivatives. *J. Org. Chem.* **2013**, *78* (13), 6558–6569. https://doi.org/10.1021/jo400845m.

(1533) Verma, A. K.; Choudhary, D.; Saunthwal, R. K.; Rustagi, V.; Patel, M.; Tiwari, R. K. On Water: Silver-Catalyzed Domino Approach for the Synthesis of Benzoxazine/Oxazine-Fused Isoquinolines and Naphthyridines from o-Alkynyl Aldehydes. *J. Org. Chem.* **2013**, *78* (13), 6657–6669. https://doi.org/10.1021/jo4009639.

(1534) Lane, T. M.; Rodriguez, O. P.; Tasz, M. K.; Sommese, A. G.; Cremer, S. E.; Bennett, D. W.; Fanwick, P. E. X-Ray Crystallographic Structures of Two Polymorphic Forms of Cis6-Carboxy-2, 10-Dioxa-l-Phosphabicyclo-[4. 4.0]Decane 1-Oxide. *Phosphorus, Sulfur, Silicon Relat. Elem.* **1995**, *102* (1–4), 115–125. https://doi.org/10.1080/10426509508042549.

(1535) Pordanjani, H. M.; Faderl, C.; Wang, J.; Motti, C. A.; Junk, P. C.; Oelgemöller, M. Photodecarboxylative Benzylations of N-Methoxyphthalimide under Batch and Continuous-Flow Conditions. *Aust. J. Chem.* **2015**, *68* (11), 1662–1667. https://doi.org/10.1071/CH15356.

(1536) Basak, A.; Sekhar, S.; Anjali, P.; Bertolasi, V.; Kumar, A. Molecular Recognition in B-Lactams. *J. Chem. Res.* **2004**, 318–321.

(1537) Aubry, A.; Marroud, M.; Protas, J.; Neel, J. Determination de La Structure Cristalline Da N-Acetyl-Methionyl-Dimethylamide. *C. R. Acad. Sci. Ser. C* **1971**, *273*, 959.

(1538) Bredikhin, A. A.; Zakharychev, D. V.; Gubaidullin, A. T.; Samigullina, A. I.; Bredikhina, Z. A. Crystal Landscape of Chiral Drug Chlorphenesin and Its Structural Analogues: Polymorphism of Racemic and Enantiopure Samples, Metastable and Stable Racemic Conglomerates, Diverse in Unity Crystal Motifs. *Cryst. Growth Des.* **2021**, *21* (6), 3211–3224. https://doi.org/10.1021/acs.cgd.0c01570.

(1539) Patil, N. T.; Patil, M. T.; Sarkar, N.; Gonnade, R. G.; Shashidhar, M. S. Access to Enantiomeric Organic Compounds with Potential for Synthesis via Racemic Conglomerates: Inositol Derivatives as a Case in Point. *Cryst. Growth Des.* **2021**, *21* (7), 3786–3797. https://doi.org/10.1021/acs.cgd.1c00126.

(1540) Siemeling, U.; Scheppelmann, I.; Neumann, B.; Stammler, A.; Stammler, H. G.; Frelek, J. Spontaneous Chiral Resolution of a Coordination Polymer with Distorted Helical Structure Consisting of Achiral Building Blocks. *Chem. Commun.* **2003**, *3* (17), 2236–2237. https://doi.org/10.1039/b307161h.

(1541) Yang, P.; Wang, J.; Jia, C.; Yang, X. J.; Wu, B. Dinuclear Chloride-Binding Foldamers Based on Fluorescent Oligoureas. *Eur. J. Org. Chem.* **2013**, *2013* (17), 3446–3454. https://doi.org/10.1002/ejoc.201300043.

(1542) Yoshizawa, K.; Toyota, S.; Toda, F. A Novel Transformation of a 1:1:1 Racemic Complex of 2,2′-Dihydroxy- 1,1′-Binaphthyl, Me4N+ Cl- and MeOH into a Conglomerate in the Solid State by Heating or Contact with MeOH Vapour. *Chem. Commun.* **2004**, *60* (16), 1844–1845. https://doi.org/10.1039/b404297b.

(1543) Yoshizawa, K.; Toyota, S.; Toda, F. Enantiomer Separation of Rac-2,2′-Dihydroxy-1,1′-Binaphthyl (BNO) by Inclusion Complexation with Racemic or Achiral Ammonium Salts and a Novel Transformation of a 1:1:1 Racemic Complex of BNO, Me4N +·Cl- and MeOH into a Conglomerate Complex in the Solid S. *Tetrahedron* **2004**, *60* (35), 7767–7774. https://doi.org/10.1016/j.tet.2004.05.122.

(1544) Lightfoot, M. P.; Mair, F. S.; Pritchard, R. G.; Warren, J. E. New Supramolecular Packing Motifs: π-Stacked Rods Encased in Triply-Helical Hydrogen Bonded Amide Strands. *Chem. Commun.* **1999**, No. 19, 1945–1946. https://doi.org/10.1039/a905245c.

(1545) Mbodji, A.; Gbabode, G.; Sanselme, M.; Couvrat, N.; Leeman, M.; Dupray, V.; Kellogg, R. M.; Coquerel, G. Family

of Conglomerate-Forming Systems Composed of Chlocyphos and Alkyl-Amine. Assessment of Their Resolution Performances by Using Various Modes of Preferential Crystallization. *Cryst. Growth Des.* **2019**, *19* (9), 5173–5183. https://doi.org/10.1021/acs.cgd.9b00568.

(1546) Chau, F. H. V.; Corey, E. J. Short and Simple Synthesis of Chelating Bis-Ethers and Bis-Amines in the Bicyclo[3.3.1]Nonane Series. *Tetrahedron Lett.* **2006**, *47* (15), 2581–2583. https://doi.org/10.1016/j.tetlet.2006.02.038.

(1547) Hager, O.; Llamas-Saiz, A. L.; Foces-Foces, C.; Claramunt, R. M.; López, C.; Elguero, J. Complexes between 1,1'-Binaphthyl-2,2'-Dicarboxylic Acid and Pyrazoles: A Case of Manual Sorting of Conglomerate Crystals (Triage). *Helv. Chim. Acta* **1999**, *82* (12), 2213–2230. https://doi.org/10.1002/(SICI)1522-2675(19991215)82:12<2213::AID-HLCA2213>3.0.CO;2-R.

(1548) Washio, A.; Hosaka, M.; Uemura, N.; Yoshida, Y.; Mino, T.; Kasashima, Y.; Sakamoto, M. Asymmetric Anisoin Synthesis Involving Benzoin Condensation Followed by Deracemization. *Cryst. Growth Des.* **2021**, *21* (4), 2423–2428. https://doi.org/10.1021/acs.cgd.1c00036.

(1549) Altona, C.; Knobler, C.; Romers, C. Investigations into the Conformation of Non-aromatic Ring-compounds X: The Crystal Structure of Trans-2,3-dibromo-1,4-dioxan at −100°. *Recl. des Trav. Chim. des Pays-Bas* **1963**, *82* (11), 1089–1098. https://doi.org/10.1002/recl.19630821112.

(1550) Le Bihan, M.-T.; Pérucaud, M.-C. Structures Atomiques Du Trans -1,2-Dichloroacénaphtène et Du Trans -1,2-Dibromoacénaphtène Antipodes. *Acta Cryst. B* **1972**, *28* (2), 629–634. https://doi.org/10.1107/s0567740872002870.

(1551) Karle, I. L.; Ottenheym, H. C. J.; Witkop, B. On the Conformation and Synthesis of Diketopiperazines. 3, 4-Dehydroproline Anhydride. *J. Am. Chem. Soc.* **1974**, *96* (2), 539–543. https://doi.org/10.1021/ja00809a033.

(1552) Upadhyay, G. M.; Mande, H. M.; Pithadia, D. K.; Maradiya, R. H.; Bedekar, A. V. Effect of the Position of the Cyano Group on Molecular Recognition, Supramolecular Superhelix Architecture, and Spontaneous Resolution of Aza[7]Helicenes. *Cryst. Growth Des.* **2019**, *19* (9), 5354–5361. https://doi.org/10.1021/acs.cgd.9b00829.

(1553) Bates, G. W.; Gale, P. A.; Light, M. E. Isophthalamides and 2,6-Dicarboxamidopyridines with Pendant Indole Groups: A "twisted" Binding Mode for Selective Fluoride Recognition. *Chem. Commun.* **2007**, No. 21, 2121–2123. https://doi.org/10.1039/b703905k.

(1554) Kravchenko, A. N.; Kadorkina, G. K.; Sigachev, A. S.; Maksareva, E. Y.; Lyssenko, K. A.; Belyakov, P. A.; Lebedev, O. V.; Kharybin, O. N.; Makhova, N. N.; Kostyanovsky, R. G. Spontaneous Resolution in the Imidazolidin-2-One Series. *Mendeleev Commun.* **2003**, *13* (3), 114–116. https://doi.org/10.1070/MC2003v013n03ABEH001736.

(1555) Valenti, G.; Tinnemans, P.; Baglai, I.; Noorduin, W. L.; Kaptein, B.; Leeman, M.; ter Horst, J. H.; Kellogg, R. M. Combining Incompatible Processes for Deracemization of a Praziquantel Derivative under Flow Conditions. *Angew. Chem. Int. Ed.* **2021**, *60* (10), 5279–5282. https://doi.org/10.1002/anie.202013502.

(1556) Yathirajan, H. S.; Nagaraj, B.; Nagaraja, P.; Bolte, M. Pioglitazone Hydrochloride. *Acta Cryst. E* **2005**, *61* (1), o154–o155. https://doi.org/10.1107/S1600536804033100.

(1557) Zhang, C.; Matzger, A. J. A Newly Discovered Racemic Compound of Pioglitazone Hydrochloride Is More Stable than the Commercial Conglomerate. *Cryst. Growth Des.* **2017**, *17* (2), 414–417. https://doi.org/10.1021/acs.cgd.6b01638.

(1558) Engwerda, A. H. J.; Koning, N.; Tinnemans, P.; Meekes, H.; Bickelhaupt, F. M.; Rutjes, F. P. J. T.; Vlieg, E. Deracemization of a Racemic Allylic Sulfoxide Using Viedma Ripening. *Cryst. Growth Des.* **2017**, *17* (8), 4454–4457. https://doi.org/10.1021/acs.cgd.7b00828.

(1559) Yagishita, F.; Okamoto, K.; Kamataki, N.; Kanno, S.; Mino, T.; Kasashima, Y.; Sakamoto, M. Chiral Symmetry Breaking of Axially Chiral Nicotinamide by Crystallization from the Melt. *Chem. Lett.* **2013**, *42* (12), 1508–1510. https://doi.org/10.1246/cl.130796.

(1560) Saigo, K.; Kimoto, H.; Nohira, H.; Yanagi, K.; Hasegawa, M. Molecular Recognition in the Formation of Conglomerate Crystal. The Role of Cinnamic Acid in the Conglomerate Crystals of 1-Phenylethylamine and 1-(4-Isopropylphenyl)Ethylamine Salts. *Bull. Chem. Soc. Jpn.* **1987**, *60* (10), 3655–3658. https://doi.org/10.1246/bcsj.60.3655.

(1561) Davey, R. J.; Sadiq, G.; Back, K.; Wilkinson, L.; Seaton, C. C. The Isolation of a Metastable Conglomerate Using a Combined Computational and Controlled Crystallization Approach. *Chem. Commun.* **2012**, *48* (14), 1976–1978. https://doi.org/10.1039/c1cc16173c.

(1562) Gilks, S. E.; Davey, R. J.; Mughal, R. K.; Sadiq, G.; Black, L. Crystallization of 2-Chloromandelic Acid: Solubility, Formation of the Metastable Conglomerate, and Use of a Nonaqueous Emulsion to Prepare an Enantiomerically

Enriched Product. *Cryst. Growth Des.* **2013**, *13* (10), 4323–4329. https://doi.org/10.1021/cg400667z.

(1563) Sun, Q.; Bai, Y.; He, G.; Duan, C.; Lin, Z.; Meng, Q. Spontaneous Resolution of Silver Double Helicates Consisting of Achiral Ligands with Several Aromatic Rings. *Chem. Commun.* **2006**, No. 26, 2777–2779. https://doi.org/10.1039/b604066g.

(1564) Neurohr, C.; Marchivie, M.; Lecomte, S.; Cartigny, Y.; Couvrat, N.; Sanselme, M.; Subra-Paternault, P. Naproxen-Nicotinamide Cocrystals: Racemic and Conglomerate Structures Generated by CO2 Antisolvent Crystallization. *Cryst. Growth Des.* **2015**, *15* (9), 4616–4626. https://doi.org/10.1021/acs.cgd.5b00876.

(1565) Ando, S.; Kikuchi, J.; Fujimura, Y.; Ida, Y.; Higashi, K.; Moribe, K.; Yamamoto, K. Physicochemical Characterization and Structural Evaluation of a Specific 2:1 Cocrystal of Naproxen–Nicotinamide. *J. Pharm. Sci.* **2012**, *101* (9), 3214–3221. https://doi.org/10.1002/JPS.23158.

(1566) Wermester, N.; Aubin, E.; Pauchet, M.; Coste, S.; Coquerel, G. Preferential Crystallization in an Unusual Case of Conglomerate with Partial Solid Solutions. *Tetrahedron: Asymmetry* **2007**, *18* (7), 821–831. https://doi.org/10.1016/j.tetasy.2007.03.011.

(1567) Mahieux, J.; Sanselme, M.; Harthong, S.; Melan, C.; Aronica, C.; Guy, L.; Coquerel, G. Preparative Resolution of (±)-Bis-Tetralone by Means of Autoseeded Preferential Crystallization Induced by Solvent Evaporation (ASPreCISE). *Cryst. Growth Des.* **2013**, *13* (8), 3621–3631. https://doi.org/10.1021/cg400589u.

(1568) Engwerda, A. H. J.; Maassen, R.; Tinnemans, P.; Meekes, H.; Rutjes, F. P. J. T.; Vlieg, E. Attrition-Enhanced Deracemization of the Antimalaria Drug Mefloquine. *Angew. Chem. Int. Ed.* **2019**, *58* (6), 1670–1673. https://doi.org/10.1002/anie.201811289.

(1569) Kostyanovsky, R. G.; Lyssenko, K. A.; Krutius, O. N.; Kostyanovsky, V. R. Isomorphism of Chiral Ammonium Salts Ph(All)N+Et(Me)X-·CHCl3. *Mendeleev Commun.* **2009**, *19* (1), 19–20. https://doi.org/10.1016/j.mencom.2009.01.008.

(1570) Lennartson, A.; Olsson, S.; Sundberg, J.; Håkansson, M. A Different Approach to Enantioselective Organic Synthesis: Absolute Asymmetric Synthesis of Organometallic Reagents. *Angew. Chem. Int. Ed.* **2009**, *48* (17), 3137–3140. https://doi.org/10.1002/anie.200806222.

(1571) Kennedy, A. R.; Morrison, C. A.; Briggs, N. E. B.; Arbuckle, W. Density and Stability Differences between Enantiopure and Racemic Salts: Construction and Structural Analysis of a Systematic Series of Crystalline Salt Forms of Methylephedrine. *Cryst. Growth Des.* **2011**, *11* (5), 1821–1834. https://doi.org/10.1021/cg200030s.

(1572) Kopf, J.; Morf, M.; Zimmer, B.; Bischoff, M.; Köll, P. The Crystal and Molecular Structures of the Hexitol Hexa-Acetates. *Carbohydr. Res.* **1992**, *229* (1), 17–32. https://doi.org/10.1016/S0008-6215(00)90477-4.

(1573) Ezuhara, T.; Endo, K.; Aoyama, Y. Helical Coordination Polymers from Achiral Components in Crystals. Homochiral Crystallization, Homochiral Helix Winding in the Solid State, and Chirality Control by Seeding. *J. Am. Chem. Soc.* **1999**, *121* (14), 3279–3283. https://doi.org/10.1021/ja9819918.

(1574) Huang, W.; Ogawa, T. Spontaneous Resolution of Δ and Λ Enantiomeric Pair of [Ru(Phen)(Bpy)2](PF6)2 (Phen = 1,10-Phenanthroline, Bpy = 2,2′-Bipyridine) by Conglomerate Crystallization. *Polyhedron* **2006**, *25* (6), 1379–1385. https://doi.org/10.1016/j.poly.2005.09.033.

(1575) Khandavilli, U. B. R.; Gavin, D. P.; Maguire, A. R.; Nolan, M.; Lawrence, S. E. Exploring the Crystal Landscape of 3-Methyl-2-Phenylbutyramide: Crystallization of Metastable Racemic Forms from the Stable Conglomerate. *Cryst. Growth Des.* **2018**, *18* (6), 3549–3557. https://doi.org/10.1021/acs.cgd.8b00348.

(1576) Shoemaker, D. P.; Donohue, J.; Schomaker, V.; Corey, R. B. The Crystal Structure of Ls-Threonine. *J. Am. Chem. Soc.* **1950**, *72* (6), 2328–2349. https://doi.org/10.1021/ja01162a002.

(1577) Uemura, N.; Hosaka, M.; Washio, A.; Yoshida, Y.; Mino, T.; Sakamoto, M. Chiral Symmetry Breaking of Thiohydantoins by Attrition-Enhanced Deracemization. *Cryst. Growth Des.* **2020**, *20* (8), 4898–4903. https://doi.org/10.1021/acs.cgd.0c00829.

(1578) Sakamoto, M.; Yagishita, F.; Ando, M.; Sasahara, Y.; Kamataki, N.; Ohta, M.; Mino, T.; Kasashima, Y.; Fujita, T. Generation and Amplification of Optical Activity of Axially Chiral N-(1-Naphthyl)-2(1H)-Pyrimidinethione by Crystallization. *Org. Biomol. Chem.* **2010**, *8* (23), 5418–5422. https://doi.org/10.1039/c0ob00262c.

(1579) Engwerda, A. H. J.; Van Schayik, P.; Jagtenberg, H.; Meekes, H.; Rutjes, F. P. J. T.; Vlieg, E. Solid Phase Deracemization of an Atropisomer. *Cryst. Growth Des.* **2017**, *17* (10), 5583–5585. https://doi.org/10.1021/acs.cgd.7b01180.

(1580) Höhne, E.; Lohs, K. H. Die Kristallstruktur Des Trichlorphons C4H8O4PCl3. *Zeitschrift fur Naturforschung B* **1969**, *244*, 1071–1074.

(1581) Yao, Q. X.; Xuan, W. M.; Zhang, H.; Tu, C. Y.; Zhang, J. The Formation of a Hydrated Homochiral Helix from an

Achiral Zwitterionic Salt, Spontaneous Chiral Symmetry Breaking and Redox Chromism of Crystals. *Chem. Commun.* **2009**, No. 1, 59–61. https://doi.org/10.1039/b815456b.

(1582) Wudl, F.; Lightner, D. A.; Cram, D. J. Methanesulfinic Acid and Its Properties. *J. Am. Chem. Soc.* **1967**, *89* (16), 4099–4101. https://doi.org/10.1021/ja00992a026.

(1583) Seff, K.; Heidner, E. G.; Meyers, M.; Trueblood, K. N. The Crystal and Molecular Structure of Methanesulfinic Acid. *Acta Cryst. Sect. B* **1969**, *25* (2), 350–354. https://doi.org/10.1107/S0567740869002214.

(1584) Byrkjedal, A.; Mostad, A.; Rømming, C. The Crystal and Molecular Structure of 3-Hydroxyphenylalanine (m-Tyrosine). *Acta Chem. Scand. B* **1974**, *28*, 750–756. https://doi.org/10.3891/acta.chem.scand.28b-0750.

(1585) Bredikhin, A. A.; Gubaidullin, A. T.; Bredikhina, Z. A. Absolute Configuration and Crystal Packing Chirality for Three Conglomerate-Forming Ortho-Halogen Substituted Phenyl Glycerol Ethers. *J. Mol. Struct.* **2010**, *975* (1–3), 323–329. https://doi.org/10.1016/j.molstruc.2010.04.045.

(1586) Shemchuk, O.; Song, L.; Tumanov, N.; Wouters, J.; Braga, D.; Grepioni, F.; Leyssens, T. Chiral Resolution of RS-Oxiracetam upon Cocrystallization with Pharmaceutically Acceptable Inorganic Salts. *Cryst. Growth Des.* **2020**, *20* (4), 2602–2607. https://doi.org/10.1021/acs.cgd.9b01725.

(1587) Sanada, K.; Washio, A.; Nishihata, K.; Yagishita, F.; Yoshida, Y.; Mino, T.; Suzuki, S.; Kasashima, Y.; Sakamoto, M. Chiral Symmetry Breaking of Racemic 3-Phenylsuccinimides via Crystallization-Induced Dynamic Deracemization. *Cryst. Growth Des.* **2021**, *21* (11), 6051–6055. https://doi.org/10.1021/acs.cgd.1c01010.

(1588) Baglai, I.; Leeman, M.; Wurst, K.; Kaptein, B.; Kellogg, R. M.; Noorduin, W. L. The Strecker Reaction Coupled to Viedma Ripening: A Simple Route to Highly Hindered Enantiomerically Pure Amino Acids. *Chem. Commun.* **2018**, *54* (77), 10832–10834. https://doi.org/10.1039/c8cc06658b.

(1589) Chen, H. F.; Guo, G. C.; Wang, M. S.; Xu, G.; Zou, W. Q.; Guo, S. P.; Wu, M. F.; Huang, J. S. Spontaneous Chiral Resolution, Nonlinear Optical and Luminescence of Eight-Coordinate Lanthanide(III) Complexes. *Dalt. Trans.* **2009**, No. 46, 10166–10168. https://doi.org/10.1039/b917559h.

(1590) Lorenz, H.; Von Langermann, J.; Sadiq, G.; Seaon, C. C.; Davey, R. J.; Seidel-Morgenstern, A. The Phase Behavior and Crystallization of 2-Chloromandelic Acid: The Crystal Structure of the Pure Enantiomer and the Behavior of Its Metastable Conglomerate. *Cryst. Growth Des.* **2011**, *11* (5), 1549–1556. https://doi.org/10.1021/cg1015077.

(1591) Kikkawa, S.; Maeno, I.; Katagiri, K.; Murayama, Y.; Nozawa, M.; Hikawa, H.; Azumaya, I. High Proportion of Chiral Crystallization of Achiral Indolyl Sulfonamides: Effect of Intermolecular Interactions. *Cryst. Growth Des.* **2021**, *21* (8), 4380–4389. https://doi.org/10.1021/acs.cgd.1c00305.

(1592) Vávra, J.; Severa, L.; Åvec, P.; Císařová, I.; Koval, D.; Sázelová, P.; Kašička, V.; Teplý, F. Preferential Crystallization of a Helicene-Viologen Hybrid - An Efficient Method to Resolve [5]Helquat Enantiomers on a 20 g Scale. *Eur. J. Org. Chem.* **2012**, *2012* (3), 489–499. https://doi.org/10.1002/ejoc.201101367.

(1593) Krämer, R.; Lehn, J. -M; De Cian, A.; Fischer, J. Self-Assembly, Structure, and Spontaneous Resolution of a Trinuclear Triple Helix from an Oligobipyridine Ligand and NiII Ions. *Angew. Chem. Int. Ed.* **1993**, *32* (5), 703–706. https://doi.org/10.1002/anie.199307031.

(1594) Navare, P. S.; MacDonald, J. C. Investigation of Stability and Structure in Three Homochiral and Heterochiral Crystalline Forms of 3-Phenyllactic Acid. *Cryst. Growth Des.* **2011**, *11* (6), 2422–2428. https://doi.org/10.1021/cg200171r.

(1595) Ibuka, T.; Inubushi, Y.; Saji, I.; Tanaka, K.; Masaki, N. Total Synthesis of Dl-Pumiliotoxin c Hydrochloride and Its Crystal Structure. *Tetrahedron Lett.* **1975**, *16* (5), 323–326. https://doi.org/10.1016/S0040-4039(00)71854-5.

(1596) Akashi, M.; Sato, Y.; Mori, M. Synthesis of Pumiliotoxine C from Molecular Nitrogen as a Nitrogen Source. *J. Org. Chem.* **2001**, *66* (23), 7873–7874. https://doi.org/10.1021/JO0104072/SUPPL_FILE/JO0104072_S.PDF.

(1597) Kostyanovsky, R. G.; Kostyanovsky, V. R.; Kadorkina, G. K.; Lyssenko, K. A. Wedekind–Fock–Havinga Salt Me(Et)N+(All)PhI−·CHCl3 as Historically the First Object for Absolute Asymmetric Synthesis: Spontaneous Resolution, Structure and Absolute Configuration. *Mendeleev Commun.* **2001**, *11* (1), 1–5. https://doi.org/10.1070/MC2001v011n01ABEH001420.

(1598) Havinga, E. Spotaneous Formation of Optically Acitve Substances. *Biochim. Biophys. Acta* **1954**, *13*, 171–174.

(1599) Ihlefeldt, F. S.; Pettersen, F. B.; Von Bonin, A.; Zawadzka, M.; Gorbitz, C. H. The Polymorphs of L-Phenylalanine. *Angew. Chem. Int. Ed.* **2014**, *53* (49), 13600–13604. https://doi.org/10.1002/anie.201406886.

(1600) Canceill, J.; Jacques, J.; Perucaud-Brianso, M. C. No Title. *Bull. Soc. Chim. Fr.* **1974**, 2833.

(1601) Isoda, K.; Haga, H.; Kamebuchi, H.; Tadokoro, M. Crystallization-Induced Planar Chirality by Asymmetric Ferrocene-Appended Tetraazanaphthacene. *Cryst. Growth Des.* **2020**, *20* (11), 7081–7086.

https://doi.org/10.1021/acs.cgd.0c00934.

(1602) Shimizu, W.; Uemura, N.; Yoshida, Y.; Mino, T.; Kasashima, Y.; Sakamoto, M. Attrition-Enhanced Deracemization and Absolute Asymmetric Synthesis of Flavanones from Prochiral Precursors. *Cryst. Growth Des.* **2020**, *20* (9), 5676–5681. https://doi.org/10.1021/acs.cgd.0c00955.

(1603) Black, S. N.; Williams, L. J.; Davey, R. J.; Moffatt, F.; McEwan, D. M.; Sadler, D. E.; Docherty, R.; Williams, D. J. Crystal Chemistry of 1-(4-Chlorophenyl)-4,4-Dimethyl-2-(1H-1,2,4-Triazol-1-Yl)Pentan-3-One, a Paclobutrazol Intermediate. *J. Phys. Chem.* **1990**, *94* (7), 3223–3226. https://doi.org/10.1021/j100370a087.

(1604) Moutin, M.; Rassat, A.; Bordeaux, D.; Lajerowicz-Bonnetau, J. Nitroxydes. LXXIV: Mise En Evidence de Deux Oxazolidines Nitroxydes Derivées Du Norcamphre. *J. Mol. Struct.* **1976**, *31* (2), 275–282. https://doi.org/10.1016/0022-2860(76)80007-5.

(1605) Kawasaki, T.; Takamatsu, N.; Aiba, S.; Tokunaga, Y. Spontaneous Formation and Amplification of an Enantioenriched α-Amino Nitrile: A Chiral Precursor for Strecker Amino Acid Synthesis. *Chem. Commun.* **2015**, *51* (76), 14377–14380. https://doi.org/10.1039/c5cc05848a.

(1606) Hassel, O.; Lund, E. W. The Crystal Structure of Tetrachlorocyclohexane, M. P. 174° C. *Acta Crystallogr.* **1949**, *2* (5), 309–311. https://doi.org/10.1107/s0365110x49000795.

(1607) Vávra, J.; Severa, L.; Císařová, I.; Klepetářová, B.; Šaman, D.; Koval, D.; Kašička, V.; Teplý, F. Search for Conglomerate in Set of [7]Helquat Salts: Multigram Resolution of Helicene-Viologen Hybrid by Preferential Crystallization. *J. Org. Chem.* **2013**, *78* (4), 1329–1342. https://doi.org/10.1021/jo301615k.

(1608) Shemchuk, O.; Spoletti, E.; Braga, D.; Grepioni, F. Solvent Effect on the Preparation of Ionic Cocrystals of Dl - Amino Acids with Lithium Chloride: Conglomerate versus Racemate Formation. *Cryst. Growth Des.* **2021**, *21* (6), 3438–3448. https://doi.org/10.1021/acs.cgd.1c00216.

(1609) Sakamoto, M.; Uemura, N.; Saito, R.; Shimobayashi, H.; Yoshida, Y.; Mino, T.; Omatsu, T. Chirogenesis and Amplification of Molecular Chirality Using Optical Vortices. *Angew. Chem. Int. Ed.* **2021**, *60* (23), 12819–12823. https://doi.org/10.1002/anie.202103382.

(1610) Custelcean, R.; Ward, M. D. Chiral Discrimination in Low-Density Hydrogen-Bonded Frameworks. *Cryst. Growth Des.* **2005**, *5* (6), 2277–2287. https://doi.org/10.1021/cg050118x.

(1611) Bredikhin, A. A.; Zakharychev, D. V.; Bredikhina, Z. A.; Kurenkov, A. V.; Krivolapov, D. B.; Gubaidullin, A. T. Spontaneous Resolution of Chiral 3-(2,3-Dimethylphenoxy)Propane-1,2-Diol under the Circumstances of an Unusual Diversity of Racemic Crystalline Modifications. *Cryst. Growth Des.* **2017**, *17* (8), 4196–4206. https://doi.org/10.1021/acs.cgd.7b00510.

(1612) Bredikhin, A. A.; Zakharychev, D. V.; Gubaidullin, A. T.; Fayzullin, R. R.; Samigullina, A. I.; Bredikhina, Z. A. Crystallization of Chiral Para-n-Alkylphenyl Glycerol Ethers: Phase Diversity and Impressive Predominance of Homochiral Guaifenesin-Like Supramolecular Motif. *Cryst. Growth Des.* **2018**, *18* (7), 3980–3987. https://doi.org/10.1021/acs.cgd.8b00321.

(1613) Uemura, N.; Toyoda, S.; Ishikawa, H.; Yoshida, Y.; Mino, T.; Kasashima, Y.; Sakamoto, M. Asymmetric Diels-Alder Reaction Involving Dynamic Enantioselective Crystallization. *J. Org. Chem.* **2018**, *83* (16), 9300–9304. https://doi.org/10.1021/acs.joc.8b01273.

(1614) Uemura, N.; Toyoda, S.; Shimizu, W.; Yoshida, Y.; Mino, T.; Sakamoto, M. Absolute Asymmetric Synthesis Involving Chiral Symmetry Breaking in Diels–Alder Reaction. *Symmetry* **2020**, *12* (6), 910. https://doi.org/10.3390/sym12060910.

(1615) Hamilton, V.; Andrusenko, I.; Potticary, J.; Hall, C.; Stenner, R.; Mugnaioli, E.; Lanza, A. E.; Gemmi, M.; Hall, S. R. Racemic Conglomerate Formation via Crystallization of Metaxalone from Volatile Deep Eutectic Solvents. *Cryst. Growth Des.* **2020**, *20* (7), 4731–4739. https://doi.org/10.1021/acs.cgd.0c00497.

(1616) Kinbara, K.; Hashimoto, Y.; Sukegawa, M.; Nohira, H.; Saigo, K. Crystal Structures of the Salts of Chiral Primary Amines with Achiral Carboxylic Acids: Recognition of the Commonly-Occurring Supramolecular Assemblies of Hydrogen-Bond Networks and Their Role in the Formation of Conglomerates. *J. Am. Chem. Soc.* **1996**, *118* (14), 3441–3449. https://doi.org/10.1021/ja9539960.

(1617) Halmöy, E.; Hassel, O. Note on the Molecular Structure of 1,2,4,5-Tetrabromocyclohexane (m. p. 185°). *J. Am. Chem. Soc.* **1939**, *61* (6), 1601–1602. https://doi.org/10.1021/ja01875a505.

(1618) Mion, L.; Casadevall, A.; Casadevall, E. No Title. *Bull. Soc. Chim. Fr.* **1968**, 2950.

(1619) Hardy, A. D. U.; MacNicol, D. D.; McKendrick, J. J.; Wilson, D. R. A Study of the Role of Hydrogen Bonding in Clathrate Formation. *Tetrahedron Lett.* **1975**, *16* (52), 4711–4712. https://doi.org/10.1016/S0040-4039(00)91059-1.

(1620) Penot, J. P.; Jacques, J.; Billard, J. Pour Reduire La Part de Hasard Dans La Recherche Des Dedoublements Spontanes (II) (1) : Un Micro-Diagnostic de l'activite Optique et Son Application. *Tetrahedron Lett.* **1968**, *9* (37), 4013–4016. https://doi.org/10.1016/S0040-4039(00)76307-6.

(1621) Hardy, A. D. U.; McKendrick, J. J.; MacNicol, D. D. Variation of Inclusion Properties by Structural Modification of a Clathrate Host. *J. Chem. Soc. Chem. Commun.* **1974**, No. 23, 972–973. https://doi.org/10.1039/C39740000972.

(1622) Janson, S. E.; Pope, W. J. The Symmetrical Spiro -Heptanediamine and Its Resolution into Optically Active Components . *Proc. R. Soc. London. Ser. A - Math. Phys. Sci.* **1936**, *154* (881), 53–60. https://doi.org/10.1098/rspa.1936.0035.

(1623) Sohail, M.; Wang, Y. F.; Wu, S. X.; Zeng, W.; Guo, J. Y.; Chen, F. X. Non-Superimposable Mirror Image Crystals of Enantiomers by Spontaneous Resolution and the Chiral Discrimination Mechanism. *Chinese Chem. Lett.* **2013**, *24* (8), 695–698. https://doi.org/10.1016/j.cclet.2013.04.045.

(1624) Jacques, J.; Collet, A.; Wilen, S. H. *Enantiomers, Racemates, and Resolutions*; Wiley: New York, 1994.

(1625) Bellies, S.; Cardinael, P.; Ndzié, E.; Petit, S.; Coquerel, G. Preferential Crystallisation and Comparative Crystal Growth Study between Pure Enantiomer and Racemic Mixture of a Chiral Molecule: 5-Ethyl-5-Methylhydantoin. *Chem. Eng. Sci.* **2001**, *56* (7), 2281–2294. https://doi.org/10.1016/S0009-2509(00)00442-5.

(1626) Dilman, A. D.; Belyakov, P. A.; Struchkova, M. I.; Arkhipov, D. E.; Korlyukov, A. A.; Tartakovsky, V. A. Fluorocyanation of Enamines. *J. Org. Chem.* **2010**, *75* (15), 5367–5370. https://doi.org/10.1021/jo1008993.

(1627) Murata, Y.; Suzuki, M.; Rubin, Y.; Komatsu, K. Structure of the Hydration Product of the C 60-Di(2-Pyridyl)-1,2,4,5-Tetrazine Adduct. *Bull. Chem. Soc. Jpn.* **2003**, *76* (8), 1669–1672. https://doi.org/10.1246/bcsj.76.1669.

(1628) Majewski, M. W.; Miller, P. A.; Oliver, A. G.; Miller, M. J. Alternate "Drug" Delivery Utilizing β-Lactam Cores: Syntheses and Biological Evaluation of β-Lactams Bearing Isocyanate Precursors. *J. Org. Chem.* **2017**, *82* (1), 737–744. https://doi.org/10.1021/acs.joc.6b02272.

(1629) Reding, A.; Jones, P. G.; Werz, D. B. Intramolecular Trans-Carbocarbonation of Internal Alkynes by a Cascade of Formal Anti-Carbopalladation/Cyclopropanol Opening. *Org. Lett.* **2018**, *20* (22), 7266–7269. https://doi.org/10.1021/acs.orglett.8b03179.

(1630) Meng, Y.; Hu, W.; Wu, K. 4-Methyl-2-Phenyl-1-Tosylpiperazine. *Acta Cryst. E* **2007**, *63* (5), o2630–o2630. https://doi.org/10.1107/S160053680701906X.

(1631) Jangra, H.; Chen, Q.; Fuks, E.; Zenz, I.; Mayer, P.; Ofial, A. R.; Zipse, H.; Mayr, H. Nucleophilicity and Electrophilicity Parameters for Predicting Absolute Rate Constants of Highly Asynchronous 1,3-Dipolar Cycloadditions of Aryldiazomethanes. *J. Am. Chem. Soc.* **2018**, *140* (48), 16758–16772. https://doi.org/10.1021/jacs.8b09995.

(1632) PENKOVA, A.; RETAILLEAU, P.; MANOLOV, I. Crystal Structure of a 1'-Ethanoyl-5'-Methyl-3'-Phenyl-1',3'-Dihydrospiro[Chroman-3,2'-Pyrrole]-2,4-Dione. *X-ray Struct. Anal. Online* **2009**, *25* (12), 125–126. https://doi.org/10.2116/xraystruct.25.125.

(1633) Koar, B.; Karaarslan, M.; Demircan, A.; Büyükgüngör, O. 3a-Bromo-Perhydro-2,6a-Epoxy-Oxireno[e][2]Benzofuran. *Acta Cryst. E* **2007**, *63* (9), o3691–o3691. https://doi.org/10.1107/S1600536807037233.

(1634) Gómez, S. L.; Palma, A.; Cobo, J.; Glidewell, C. Five 2-Aryl-Substituted Tetra-Hydro-1,4-Ep-Oxy-1-Benzazepines: Isolated Mol-Ecules and Hydrogen-Bonded Chains and Sheets. *Acta Cryst. C* **2010**, *66* (4), o233–o240. https://doi.org/10.1107/S0108270110009662.

(1635) Yeap, C. S.; Hemamalini, M.; Fun, H. K. N,N'-Bis(2,6-Dichloro-Benzyl-Idene)Propane-1,2-Diamine. *Acta Cryst. E* **2010**, *66* (4), 753. https://doi.org/10.1107/S160053681000766X.

(1636) Fun, H. K.; Quah, C. K.; Priya, S.; Narayana, B.; Sarojini, B. K. 1-[5-(4-Chlorophenyl)-3-(4-Hydroxyphenyl)-4,5-Dihydro-1H-Pyrazol-1-Yl] -Ethanone. *Acta Cryst. E* **2012**, *68* (3), o818–o818. https://doi.org/10.1107/S1600536812006885.

(1637) Daszkiewicz, M.; Puszyńska-Tuszkanow, M.; Staszak, Z.; Chojnacka, I.; Fałtynowicz, H.; Cieślak-Golonka, M. Single Crystal-to-Single Crystal Transformations Induced by Ammonia-Water Equilibrium Changes. *CrystEngComm* **2018**, *20* (21), 2907–2911. https://doi.org/10.1039/c8ce00401c.

(1638) Ishida, S.; Hirakawa, F.; Iwamoto, T. Reactions of a Stable Phosphinyl Radical with Stable Aminoxyl Radicals. *Chem. Lett.* **2015**, *44* (1), 94–96. https://doi.org/10.1246/cl.140885.

(1639) Ji, S.; Qiao, X.; Li, Z. W.; Wang, Y. R.; Yu, S. W.; Liang, W. F.; Lin, X. H.; Ye, M. Enantiomeric 3-Arylcoumarins and 2-Arylcoumarones from the Roots of Glycyrrhiza Uralensis as Protein Tyrosine Phosphatase 1B (PTP1B)

Inhibitors. *RSC Adv.* **2015**, *5* (56), 45258–45265. https://doi.org/10.1039/c5ra06452j.

(1640) Basvani, K. R.; Kindermann, M. K.; Frauendorf, H.; Schulzke, C.; Jones, P. G.; Heinicke, J. W. 3-Phenylphosphaprolines – Synthesis, Structure and Properties of Heterocyclic α-Phosphanyl Amino Acids. *Polyhedron* **2017**, *130*, 195–204. https://doi.org/10.1016/j.poly.2017.04.014.

(1641) Carlton, J. B.; Levin, R. H.; Clardy, J. 1,6-Methano[10]Annulen-11-Ylidene. *J. Am. Chem. Soc.* **1976**, *98* (19), 6068–6070. https://doi.org/10.1021/ja00435a065.

(1642) Nagarajan, K.; Rao, S.; Shridhara, K.; Nayak, S.; Thomas, S.; Row, G.; Pari, K.; Jithendranath, S. Structural Studies on Nitroimidazooxazoles with Antitubercular and Antileishmanial Activities. *Indian J. Chem. -Section B* **2017**, *56* (02), 145–151.

(1643) Ermolovich, Y.; Barysevich, M. V.; Adamson, J.; Rogova, O.; Kaabel, S.; Järving, I.; Gathergood, N.; Snieckus, V.; Kananovich, D. G. Site-Selective and Stereoselective C-H Functionalization of N-Cyclopropylamides via a Directed Remote Metalation Strategy. *Org. Lett.* **2019**, *21* (4), 969–973. https://doi.org/10.1021/acs.orglett.8b03955.

(1644) Elkin, I.; Maris, T.; Melkoumov, A.; Hildgen, P.; Banquy, X.; Leclair, G.; Barrett, C. Crystal Structure of 2-Oxopyrrolidin-3-Yl 4-(2-Phenyldiazen-1-Yl)Benzoate. *Acta Crystallogr. Sect. E Crystallogr. Commun.* **2018**, *74* (4), 458–460. https://doi.org/10.1107/S205698901800333X.

(1645) Koşar, B.; Demircan, A.; Karaarslan, M.; Büyükgüngör, O. Terf-Butyl N-(2-Bromocyclohex-2-Enyl)-N-(2-Furylmethyl)Carbamate. *Acta Cryst. E* **2006**, *62* (2), o765–o767. https://doi.org/10.1107/S1600536806002224.

(1646) Chen, M.; Shen, W.; Bao, L.; Cai, W.; Xie, Y.; Akasaka, T.; Lu, X. Regioselective Thermal Reaction between Triethylamine and C60 Revisited: X-Ray Confirmation of the Pentane-Fused Adduct and in Situ Mechanism Study. *Eur. J. Org. Chem.* **2015**, *2015* (26), 5742–5746. https://doi.org/10.1002/ejoc.201500814.

(1647) Yoo, C. L.; Fettinger, J. C.; Kurth, M. J. Stannous Chloride in Alcohol: A One-Pot Conversion of 2-Nitro-N-Arylbenzamides to 2,3-Dihydro-1H-Quinazoline-4-Ones. *J. Org. Chem.* **2005**, *70* (17), 6941–6943. https://doi.org/10.1021/jo050450f.

(1648) Zuo, Y.; He, X.; Ning, Y.; Wu, Y.; Shang, Y. Rh(III)-Catalyzed C-H Activation/Intramolecular Cyclization: Access to N-Acyl-2,3-Dihydro-1H-Carbazol-4(9H)-Ones from Cyclic 2-Diazo-1,3-Diketones and N-Arylamides. *ACS Omega* **2017**, *2* (11), 8507–8516. https://doi.org/10.1021/acsomega.7b01637.

(1649) Clegg, W.; Harrington, R. W.; North, M.; Villuendas, P. A Bimetallic Aluminum(Salen) Complex for the Synthesis of 1,3-Oxathiolane-2-Thiones and 1,3-Dithiolane-2-Thiones. *J. Org. Chem.* **2010**, *75* (18), 6201–6207. https://doi.org/10.1021/jo101121h.

(1650) North, H.; Wutoh, K.; Odoom, M. K.; Karla, P.; Scott, K. R.; Butcher, R. J. 3-(4-Chloroanilino)-2,5-Dimethylcyclohex-2-En-1-One. *Acta Cryst. E* **2011**, *67* (5), o1283–o1284. https://doi.org/10.1107/S1600536811005678.

(1651) Akkurt, M.; Horton, P. N.; Mohamed, S. K.; Younes, S. H. H.; Albayati, M. R. Crystal Structure of 3-Amino-1-(4-Chlorophenyl)-1H-Benzo[f]Chromene-2-Carbonitrile. *Acta Cryst. E* **2015**, *71* (7), o481–o482. https://doi.org/10.1107/S2056989015011159.

(1652) Gayathri, A.; Rajeswari, K.; Vidhyasagar, T.; Selvanayagam, S. Crystal Structures of Salen-Type Ligands 2-[(1 E)-({1-(3-Chlorophenyl)-2-[(E)-(2-Hydroxybenzylidene)Amino]Propyl}imino)Methyl]Phenol and 2-[(1 E)-({1-(4-Chlorophenyl)-2-[(E)-(2-Hydroxybenzylidene)Amino]Propyl}imino)Methyl]Phenol. *Acta Cryst. E* **2017**, *73* (12), 1878–1881. https://doi.org/10.1107/S2056989017016292.

(1653) Liu, W. Y.; Huo, P. (2-Chlorophenyl)(Diphenylphosphoryl)Methanol. *Acta Cryst. E* **2008**, *64* (1), o233–o233. https://doi.org/10.1107/S1600536807057674.

(1654) Jenson, H. Unknown Title. *Chem. Abstr.* **1970**, *73*, 77222.

(1655) Ohata, K.; Fukumi, H.; Ishiwata, H.; Yajima, M. Unknown Title. *Chem. Abstr.* **1976**, *85*, 159702.

(1656) Shiraiwa, T.; Kiyoe, R. Optical Resolution by Preferential Crystallization of (1RS,3RS)-1,2,3,4- Tetrahydro-6,7-Dihydroxy-1-Methyl-3-Isoquinolinecarboxylic Acid. *Chem. Pharm. Bull.* **2005**, *53* (9), 1197–1199. https://doi.org/10.1248/cpb.53.1197.

(1657) British Patent No. 1,241,405.

(1658) Hein, J. E.; Cao, B. H.; Van Der Meijden, M. W.; Leeman, M.; Kellogg, R. M. Resolution of Omeprazole Using Coupled Preferential Crystallization: Efficient Separation of a Nonracemizable Conglomerate Salt under near-Equilibrium Conditions. *Org. Process Res. Dev.* **2013**, *17* (6), 946–950. https://doi.org/10.1021/op400081c.

(1659) Brandel, C.; Amharar, Y.; Rollinger, J. M.; Griesser, U. J.; Cartigny, Y.; Petit, S.; Coquerel, G. Impact of Molecular

Flexibility on Double Polymorphism, Solid Solutions and Chiral Discrimination during Crystallization of Diprophylline Enantiomers. *Mol. Pharm.* **2013**, *10* (10), 3850–3861. https://doi.org/10.1021/mp400308u.

(1660) Velluz, L.; Amiard, G. Unknown Title. *Bull. Soc. Chim. Fr.* **1953**, *20*, 903.

(1661) Amiard, G. Unknown Title. *Bull. Soc. Chim. Fr.* **1956**, *20*, 903.

(1662) Reinhold, D. F.; Firestone, R. A.; Gaines, W. A.; Chemerda, J. M.; Sletzinger, M. Synthesis of L-.Alpha.-Methyldopa from Asymmetric Intermediates. *J. Org. Chem.* **1968**, *33* (3), 1209–1213. https://doi.org/10.1021/jo01267a059.

(1663) Harfouche, L. C.; Brandel, C.; Cartigny, Y.; Ter Horst, J. H.; Coquerel, G.; Petit, S. Enabling Direct Preferential Crystallization in a Stable Racemic Compound System. *Mol. Pharm.* **2019**, *16* (11), 4670–4676. https://doi.org/10.1021/acs.molpharmaceut.9b00805.

(1664) Menozzi, A.; Appiani, G. Unknown Title. *Atti R. Accad. Lincei* **1893**, *5* (2), 415.

(1665) Menozzi, A.; Appiani, G. Unknown Title. *Chem. Zentralbl.* **1894**, *I* (65), 463.

(1666) Harada, K. The Optical Resolution of DL-Aspartic Acid, DL-Glutamic Acid, DL-Asparagine and DL-Glutamine by Preferential Crystallization. *Bull. Chem. Soc. Jpn.* **1965**, *38* (9), 1552–1555. https://doi.org/10.1246/bcsj.38.1552.

(1667) Brienne, M.-J.; Jacques, J. Unknown Title. *Bull. Soc. Chim. Fr.* **1974**, 2647.

(1668) Wermester, N.; Lambert, O.; Coquerel, G. Preferential Crystallization (AS3PC Mode) of Modafinic Acid: An Example of Productivity Enhancement by Addition of a Non-Chiral Base. *CrystEngComm* **2008**, *10* (6), 724–733. https://doi.org/10.1039/b715975g.

(1669) British Patent No. 1,150,851, 1969.

(1670) Hongo, C.; Shibazaki, M.; Yamada, S.; Chibata, I. Preparation of Optically Active Proline. Optical Resolution of N-Acyl-DL-Proline by Preferential Crystallization Procedure. *J. Agric. Food Chem.* **1976**, *24* (5), 903–906. https://doi.org/10.1021/jf60207a049.

(1671) Bison, G.; Jansen, P.; R., S. French Patent No. 2,163,740, 1973.

(1672) Galan, K.; Eicke, M. J.; Elsner, M. P.; Lorenz, H.; Seidel-Morgenstern, A. Continuous Preferential Crystallization of Chiral Molecules in Single and Coupled Mixed-Suspension Mixed-Product-Removal Crystallizers. *Cryst. Growth Des.* **2015**, *15* (4), 1808–1818. https://doi.org/10.1021/cg501854g.

(1673) Dutch Patent No. 6,514,950, 1966.

(1674) Petruševska-Seebach, K.; Seidel-Morgenstern, A.; Elsner, M. P. Preferential Crystallization of l -Asparagine in Water. *Cryst. Growth Des.* **2011**, *11* (6), 2149–2163. https://doi.org/10.1021/cg101408e.

(1675) AKASHI, T. Racemic Modifications of the Derivatives of Glutamic Acid and Their Optical Resolution by Means of Inoculation Method. *Nippon kagaku zassi* **1962**, *83* (5), 532-536,A34. https://doi.org/10.1246/nikkashi1948.83.5_532.

(1676) Fouquey, S.; Jacques, J. Unknown Title. *Bull. Soc. Chim. Fr.* **1966**, 165.

(1677) Collet, A.; Brienne, M.-J.; Jacques, J. Unknown Title. *Bull. Soc. Chim. Fr.* **1972**, 127.

(1678) Gendron, F. X.; Mahieux, J.; Sanselme, M.; Coquerel, G. Resolution of Baclofenium Hydrogenomaleate by Using Preferential Crystallization. A First Case of Complete Solid Solution at High Temperature and a Large Miscibility Gap in the Solid State. *Cryst. Growth Des.* **2019**, *19* (8), 4793–4801. https://doi.org/10.1021/acs.cgd.9b00665.

(1679) Santhanam, J. British Patent No. 1,210,495, 1967.

(1680) Collet, A.; Brienne, M.-J.; Jacques, J. Unknown Title. *Bull. Soc. Chim. Fr.* **1977**, 494.

(1681) Shiraiwa, T.; Sado, Y.; Komure, M.; Kurokawa, H. Optical Resolution by Preferential Crystallization of DL-Thiazolidine-4-Carboxylic Acid. *Bull. Chem. Soc. Jpn.* **1987**, *60* (9), 3277–3283. https://doi.org/10.1246/bcsj.60.3277.

(1682) INAGAKI, M.; KUNIYOSHI, I.; NABETA, S. The Optical Resolution of DL-O-Acetylpantolactone by Seeded Crystallization in the Binary System. *YAKUGAKU ZASSHI* **1976**, *96* (1), 71–74. https://doi.org/10.1248/yakushi1947.96.1_71.

(1683) Hongo, C.; Tohyama, M.; Yoshioka, R.; Yamada, S.; Chibata, I. Asymmetric Transformation of DL- p -Hydroxyphenylglycine by a Combination of Preferential Crystallization and Simultaneous Racemization of the o -Toluenesulfonate . *Bull. Chem. Soc. Jpn.* **1985**, *58* (2), 433–436. https://doi.org/10.1246/bcsj.58.433.

(1684) Shibazaki, M. Japanese Patent No. 1576, 1965.

(1685) Courvoisier, L.; Ndzié, E.; Petit, M. N.; Hedtmann, U.; Sprengard, U.; Coquerel, G. Influence of the Process on

the Mechanisms and the Performances of the Preferential Crystallization: Example with (±)-5-(4-Bromophenyl)-5-Methylhydantoin. *Chem. Lett.* **2001**, *30* (4), 364–365. https://doi.org/10.1246/cl.2001.364.

(1686) Scheibler, H. Über Die Gegenseitigen Genetischen Beziehungen Der Optisch-aktiven Formen von β,B′-Iminodibuttersäure Und B-Aminobuttersäure. *Berichte der Dtsch. Chem. Gesellschaft* **1912**, *45* (2), 2272–2297. https://doi.org/10.1002/cber.191204502110.

(1687) Zaugg, H. E. A Mechanical Resolution of Dl-Methadone Base. *J. Am. Chem. Soc.* **1955**, *77* (10), 2910. https://doi.org/10.1021/ja01615a084.

(1688) Vogler, K.; Baumgartner, H. Spaltung VonDL-3,4-Dioxyphenylalanin in Die Optischen Antipoden. *Helv. Chim. Acta* **1952**, *35* (5), 1776–1780. https://doi.org/10.1002/hlca.19520350544.

(1689) Pallavicini, M.; Bolchi, C.; Binda, M.; Ferrara, R.; Fumagalli, L.; Piccolo, O.; Valoti, E. Entrainment Resolution of Carnitinamide Chloride. *Tetrahedron: Asymmetry* **2008**, *19* (14), 1637–1640. https://doi.org/10.1016/j.tetasy.2008.06.011.

(1690) Galland, A.; Dupray, V.; Lafontaine, A.; Berton, B.; Sanselme, M.; Atmani, H.; Coquerel, G. Preparative Resolution of (±)-Trans-1,2-Diaminocyclohexane by Means of Preferential Crystallization of Its Citrate Monohydrate. *Tetrahedron Asymmetry* **2010**, *21* (18), 2212–2217. https://doi.org/10.1016/j.tetasy.2010.07.019.

(1691) Velluz, L.; Amiard, G.; Joly, R. Unknown Title. *Bull. Soc. Chim. Fr.* **1953**, *35*, 1777.

(1692) Pallavicini, M.; Bolchi, C.; Di Pumpo, R.; Fumagalli, L.; Moroni, B.; Valoti, E.; Demartin, F. Resolution of 5-Hydroxymethyl-2-Oxazolidinone by Preferential Crystallization and Investigations on the Nature of the Racemates of Some 2-Oxazolidinone Derivatives. *Tetrahedron Asymmetry* **2004**, *15* (10), 1659–1665. https://doi.org/10.1016/j.tetasy.2004.03.038.

(1693) Hein, J. E.; Huynh Cao, B.; Viedma, C.; Kellogg, R. M.; Blackmond, D. G. Pasteur's Tweezers Revisited: On the Mechanism of Attrition-Enhanced Deracemization and Resolution of Chiral Conglomerate Solids. *J. Am. Chem. Soc.* **2012**, *134* (30), 12629–12636. https://doi.org/10.1021/ja303566g.

(1694) Damois, E. Disymmetrie Moleculaire. *Compt. Rend. Acad. Sci. Paris* **1953**, *237*, 124–126.

(1695) Bolchi, C.; Pallavicini, M.; Fumagalli, L.; Rusconi, C.; Binda, M.; Valoti, E. Resolution of 2-Substituted 1,4-Benzodioxanes by Entrainment. *Tetrahedron: Asymmetry* **2007**, *18* (9), 1038–1041. https://doi.org/10.1016/j.tetasy.2007.04.026.

(1696) Sato, N.; Uzuki, T.; Toi, K.; Akashi, T. Direct Resolution of DL-Lysine-3, 5-Dinitrobenzoate. *Agric. Biol. Chem.* **1969**, *33* (7), 1107–1108. https://doi.org/10.1271/bbb1961.33.1107.

(1697) Leeman, M.; Querniard, F.; Vries, T. R.; Kaptein, B.; Kellogg, R. M. The Resolution of 2-Hydroxy-5,5-Dimethyl-4-Phenyl-1,3,2-Dioxaphosphorinan 2-Oxide (Phencyphos) by Preferential Crystallization. *Org. Process Res. Dev.* **2009**, *13* (6), 1379–1381. https://doi.org/10.1021/op900171k.

(1698) Potter, G. A.; Garcia, C.; McCague, R.; Adger, B.; Collet, A. Oscillating Crystallization of (+) and (–) Enantiomers during Resolution by Entrainment of 2-Azabicyclo[2.2.1]Hept-5-En-3-One. *Angew. Chem. Int. Ed.* **1996**, *35* (15), 1666–1668. https://doi.org/10.1002/ANIE.199616661.

(1699) Dunn, A. S.; Svoboda, V.; Sefcik, J.; Ter Horst, J. H. Resolution Control in a Continuous Preferential Crystallization Process. *Org. Process Res. Dev.* **2019**, *23* (9), 2031–2041. https://doi.org/10.1021/acs.oprd.9b00275.

(1700) Srivastava, R. P.; Zjawiony, J. K.; Peterson, J. R.; McChesney, J. D. N-Benzoyl-(2R,3S)-3-Phenylisoserine Methyl Ester; a Facile and Convenient Synthesis and Resolution by Entrainment. *Tetrahedron: Asymmetry* **1994**, *5* (9), 1683–1688. https://doi.org/10.1016/0957-4166(94)80079-0.

(1701) Karady, S.; Ly, M. G.; Pines, S. H.; Sletzinger, M. Synthesis of D- and L-.Alpha.-(3,4-Dihydroxybenzyl)-.Alpha.-Hydrazinopropionic Acid via Resolution. *J. Org. Chem.* **1971**, *36* (14), 1946–1948. https://doi.org/10.1021/jo00813a023.

(1702) Wang, X. J.; Ching, C. B. A Systematic Approach for Preferential Crystallization of 4-Hydroxy-2-Pyrrolidone: Thermodynamics, Kinetics, Optimal Operation and in-Situ Monitoring Aspects. *Chem. Eng. Sci.* **2006**, *61* (8), 2406–2417. https://doi.org/10.1016/j.ces.2005.11.008.

(1703) Ushio, T.; Tamura, R.; Takahashi, H.; Azuma, N.; Yamamoto, K. Unusual Enantiomeric Resolution Phenomenon Observed upon Recrystallization of a Racemic Compound. *Angew. Chem. Int. Ed.* **1996**, *35* (20), 2372–2374. https://doi.org/10.1002/ANIE.199623721.

(1704) Lipp, P. Über Isocamphan. *Justus Liebigs Ann. Chem.* **1911**, *382* (3), 265–305. https://doi.org/10.1002/JLAC.19113820303.

(1705) Miyazaki, H.; Morita, H.; Shiraiwa, T.; Kurokawa, H. Optical Resolution by Preferential Crystallization and Replacing Crystallization of DL-Allothreonine. *Bull. Chem. Soc. Jpn.* **1994**, *67* (7), 1899–1903. https://doi.org/10.1246/bcsj.67.1899.

(1706) Sanfilippo, C.; Forni, A.; Patti, A. Characterization of a Conglomerate-Forming Derivative of (±)-Milnacipran and Its Enantiomeric Resolution by Preferential Crystallization. *RSC Adv.* **2016**, *6* (55), 49876–49882. https://doi.org/10.1039/c6ra07745e.

(1707) Long, L. M. U.S. Patent No. 2,767,213, 1966.

(1708) Shiraiwa, T.; Kubo, M.; Watanabe, M.; Nakatani, H.; Ohkubo, M.; Kurokawa, H. Optical Resolution by Preferential Crystallization of (RS)-2-Amino-3-(2-Earboxyethylthio)Propanoic Acid. *Biosci. Biotechnol. Biochem.* **1998**, *62* (4), 818–820. https://doi.org/10.1271/bbb.62.818.

(1709) Ros, F.; Molina, M. T. A New Racemic Conglomerate and Its Characterization by the Melting Point Diagram – Formation of the Metastable Racemic Compound. *Eur. J. Org. Chem.* **1999**, *1999* (11), 3179–3183. https://doi.org/10.1002/(SICI)1099-0690(199911)1999:11<3179::AID-EJOC3179>3.0.CO;2-0.

(1710) Reid, J. A.; Turner, E. E. 709. Partial Asymmetric Synthesis with Keto-Esters. Part I. *J. Chem. Soc.* **1951**, 3219–3223. https://doi.org/10.1039/JR9510003219.

(1711) Cascella, F.; Temmel, E.; Seidel-Morgenstern, A.; Lorenz, H. Efficient Resolution of Racemic Guaifenesin via Batch-Preferential Crystallization Processes. *Org. Process Res. Dev.* **2020**, *24* (1), 50–58. https://doi.org/10.1021/acs.oprd.9b00413.

(1712) Temmel, E.; Eicke, M. J.; Cascella, F.; Seidel-Morgenstern, A.; Lorenz, H. Resolution of Racemic Guaifenesin Applying a Coupled Preferential Crystallization-Selective Dissolution Process: Rational Process Development. *Cryst. Growth Des.* **2019**, *19* (6), 3148–3157. https://doi.org/10.1021/acs.cgd.8b01660.

(1713) Kostyanovsky, R. G.; Lakhvich, F. A.; Philipchenko, P. M.; Lenev, D. A.; Torbeev, V. Y.; Lyssenko, K. A. (±)-Trans-1,2-Diaminocyclohexane Crystallises As a Conglomerate. *Mendeleev Commun.* **2002**, *12* (4), 147–148. https://doi.org/10.1070/mc2002v012n04abeh001598.

(1714) Vizitiu, D.; Iona, R.; Lacoste, J.-E.; Simion, D. US Patent No. 7,501,514, 2009.

(1715) Marcuccio, S. M.; Epa, R.; White, J. M.; Deadman, J. J. A New Process for Synthesis of Apricitabine, 2-(R)-Hydroxymethyl-4-(R)-(Cytosin-1′-Yl)-1,3-Oxathiolane, an Anti-HIV NRTI. *Org. Process Res. Dev.* **2011**, *15* (4), 763–773. https://doi.org/10.1021/op2000332.

(1716) Aubin, E.; Petit, M. N.; Coquerel, G. Resolution of the Ethanolamine Salt of (±)Mandelic Acid by Using the AS3PC Method: Principle, Application and Results. *J. Phys. IV JP* **2004**, *122*, 157–162. https://doi.org/10.1051/jp4:2004122024.

(1717) Collet, A.; Brienne, M.-J.; Jacques, J. Optical Resolution by Direct Crystallization of Enantiomer Mixtures. *Chem. Rev.* **1980**, *80* (3), 215–230. https://doi.org/10.1021/cr60325a001.
